# *Helicobacter pylori*–induced PPFIA4 orchestrates immune network–promoting gastritis and gastric bacterial colonization

**DOI:** 10.1172/JCI193848

**Published:** 2026-05-01

**Authors:** Pan Wang, Nan You, Yong-Sheng Teng, Yi-Pin Lv, Wen-Qing Tian, Jing-Yu Xu, Rui Xie, Jiang-Bo Wu, Geng-Yu Yue, Ping Cheng, Jin-Yu Zhang, Liu-Sheng Peng, Fang-Yuan Mao, Shou-Lu Luo, Shi-Ming Yang, Yong-Liang Zhao, Hong Zhou, Weisan Chen, Bin Wang, Yuan Zhuang

**Affiliations:** 1 Department of Gastroenterology, The 940 Hospital of Joint Logistic Support Force of PLA, Lanzhou, China.; 2 Collaborative Innovation Center of Tissue Damage Repair and Regeneration Medicine, Zunyi Medical University, Zunyi, China.; 3 National Engineering Research Center of Immunological Products, Department of Microbiology and Biochemical Pharmacy, College of Pharmacy and Laboratory Medicine, and; 4 Department of Hepatobiliary Surgery, XinQiao Hospital, Third Military Medical University, Chongqing, China.; 5 Department of Gastroenterology, Chongqing General Hospital, Chongqing University, Chongqing, China.; 6 Department of Infectious Disease, The General Hospital of Western Theater Command, Chengdu, China.; 7 Department of Gastroenterology, Chongqing University Cancer Hospital, Chongqing, China.; 8 Department of Endoscopy and Digestive System, Guizhou Provincial People’s Hospital, Guiyang, China.; 9 Department of Gastroenterology, XinQiao Hospital, and; 10 Department of General Surgery and Center of Minimal Invasive Gastrointestinal Surgery, Southwest Hospital, Third Military Medical University, Chongqing, China.; 11 Department of Cell Biology, School of Life Sciences, Anhui Medical University, Hefei, China.; 12 La Trobe Institute of Molecular Science, La Trobe University, Bundoora, Victoria, Australia.; 13 Jinfeng Laboratory, Chongqing, China.; 14 Department of Gastroenterology, Chongqing Key Laboratory of Digestive Malignancies, Daping Hospital, Third Military Medical University, Chongqing, China.

**Keywords:** Gastroenterology, Infectious disease, Bacterial infections

## Abstract

Bacteria-modulated gastric epithelial cells (GECs) play key roles in *Helicobacter pylori*–associated pathology. Here, we demonstrate both procolonization and proinflammation roles of GEC-derived PPFIA4 in *H*. *pylori* infection. PPFIA4 was elevated in GECs from gastric mucosa of *H*. *pylori*–infected patients and mice. PPFIA4 could be synergistically induced by *H*. *pylori* and IL-33 via the CagA/AP1 pathway. Human gastric PPFIA4 correlated with *H*. *pylori* colonization and the severity of gastritis, and *H*. *pylori* colonization and inflammation were attenuated in *Ppfia4*^ΔGEC^ mice. Mechanistically, PPFIA4’s SAM1 domain bound domains from CaMK to the first L27 of CASK and subsequently formed a PPFIA4/CASK/AKT1 complex to activate AKT1, resulting in NF-κB activation and MMP1/CXCL3 secretion. This not only led to decreased E-cadherin and ZO-1 by MMP1, thereby promoting gastric mucosal damage to foster *H*. *pylori* colonization, but also resulted in increased gastric influx of G-MDSCs via CXCL3-dependent migration, thereby promoting gastritis and impairing *H*. *pylori*–specific IFN-γ–producing CD4^+^ T cell responses to foster *H*. *pylori* colonization. Furthermore, we identified a PPFIA4 inhibitor, kira6, which effectively inhibited GEC’s MMP1/CXCL3 production and ameliorated gastric *H*. *pylori* colonization and gastritis. Overall, PPFIA4 could be a promising therapeutic target, as it collectively ensures *H*. *pylori* persistence and promotes gastritis.

## Abstract 2

PPFIA4 could potentially serve as a promising therapeutic target as it collectively ensures *H. pylori* persistence and promotes gastritis.

## Graphical abstract

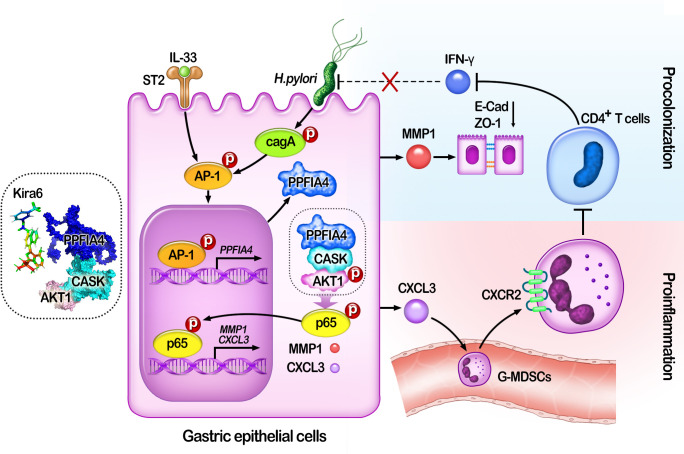

## Introduction

*Helicobacter pylori* is a Gram-negative stomach-colonized pathogen that infects more than half of the world’s population (1). Persistent *H*. *pylori* infection induces chronic gastritis, which can lead to gastric ulcers and gastric malignancy (2). The first detectable host response to the pathogen is an increase in submucosal and intraepithelial inflammatory immune cells, which is characterized by the activation of CD4^+^ T cells that play a decisive role in controlling gastric *H*. *pylori* load via secretion of IFN-γ (3). However, such T cells do not efficiently eradicate this pathogen (4), suggesting that progressive immune network regulations create an environment favorable for gastric *H*. *pylori* colonization along with chronic gastritis development (5). Hence, insight into mechanisms by which *H*. *pylori* regulates the immune network may help in the prevention of *H*. *pylori* persistence and chronic gastritis.

Upon infection, *H*. *pylori* colonization takes place in the gastric mucus and eventually involves bacterial adherence to gastric epithelial cells (GECs). GECs are known to be not only the first contacted cells but also the major effector cells modulated by *H*. *pylori* in gastric mucosa (6). Although the persistent colonization of *H*. *pylori* and the development of *H*. *pylori*–associated gastritis remain poorly understood, it is believed that, in gastric mucosa, the modulated GECs by *H*. *pylori* are key contributors (7). *H*. *pylori* can modulate GECs by extracting lipid from them, which abrogates phagocytosis of *H*. *pylori* and subsequent T cell activation (8), suggesting that altered GECs are involved in the subsequent immune evasion or response during *H*. *pylori* infection. Yet, efforts to identify GEC-derived factors that regulate the immune network, foster gastritis, and benefit bacterial colonization during *H*. *pylori* infection have so far failed.

There is increasing interest in the interplay between neuronal signals and the tissue immune network in infectious diseases (9). However, it is currently unknown whether such factors, that were originally identified in the nervous system, are induced in GECs in response to *H*. *pylori* infection, how they are influenced by immune molecules, and how immune cells in turn might be affected during *H*. *pylori* infection. Here, we identified a factor, protein tyrosine phosphatase receptor type F polypeptide interacting protein alpha 4 (PPFIA4), originally identified in neurons of the nervous system (10), that is elevated in GECs of gastric mucosa from *H*. *pylori*–infected patients and mice. Normally, PPFIA4 acts as a scaffold protein for regulating neurotransmitter release at the active zone by interacting with other proteins (11). The human *PPFIA4* gene was identified in 2003 (12), and PPFIA4 can be induced under pathological conditions such as carcinogenesis (13). In this study, we show that PPFIA4 can be induced in GECs following *H*. *pylori* infection dependent on the bacterial virulence factor CagA and can be synergistically induced by the immune molecule IL-33. Importantly, specifically knocking out GEC-derived PPFIA4 in *Gif*-*Cre* *Ppfia4*^fl/fl^ (*Ppfia4*^ΔGEC^) mice attenuated both *H*. *pylori* colonization and inflammation. In *H*. *pylori*–infected GECs, PPFIA4 binds to calcium/calmodulin-dependent serine protein kinase (CASK) and subsequently forms a PPFIA4/CASK/AKT1 complex to activate AKT1, which activates NF-κB to induce MMP1/CXCL3 secretion. MMP1 decreases E-cadherin and zonula occludens-1 (ZO-1) proteins to promote gastric mucosal damage, and CXCL3 increases gastric influx of granulocytic myeloid-derived suppressor cells (G-MDSCs), promoting gastritis and impairing *H*. *pylori*–specific IFN-γ–producing CD4^+^ T cell response to foster *H*. *pylori* colonization. More importantly, a PPFIA4 inhibitor, kira6, that effectively inhibits GEC’s MMP1/CXCL3 production in vitro and ameliorates gastric *H*. *pylori* colonization and gastritis in vivo has been identified.

Together, we have systematically evaluated the expression, regulation, and function of PPFIA4 in *H*. *pylori* infection and elucidated the mechanisms underlying the role of the PPFIA4-involved immune network in persistent *H*. *pylori* infection–induced clinical gastritis, showing PPFIA4 as a potential therapeutic target in *H*. *pylori* infection.

## Results

### PPFIA4 is increased in GECs from gastric mucosa of H. pylori–infected patients and mice.

**Figure 1:**
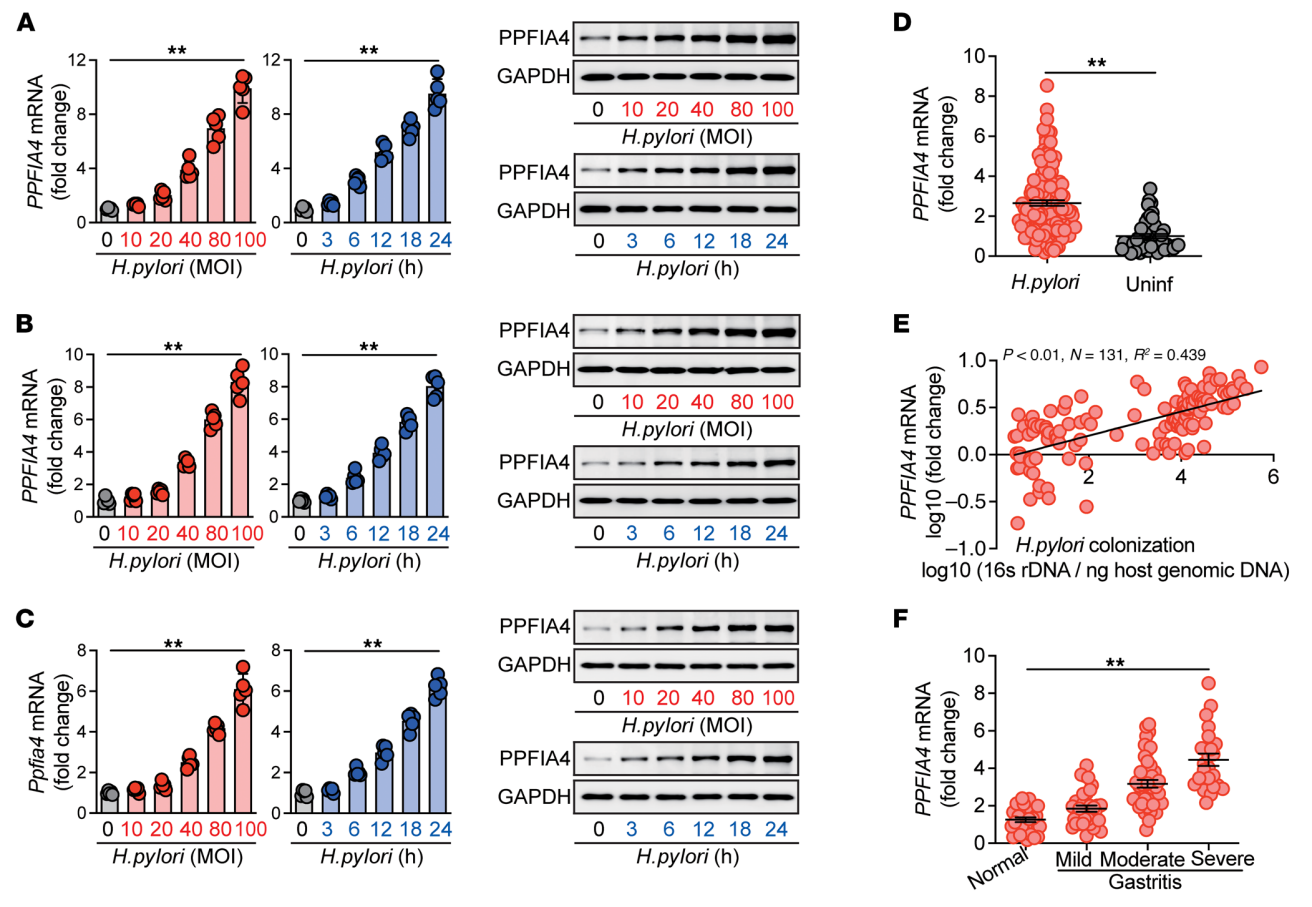
PPFIA4 is increased in GECs from gastric mucosa of *H*. *pylori*–infected patients and mice. (**A**–**C**) *PPFIA4/Ppfia4* expression and PPFIA4 protein in *H*. *pylori*–infected AGS cells (**A**), and human (**B**) and mouse (**C**) primary GECs infected with different MOIs (24 hours) or at different time points (MOI = 100) were analyzed by real-time PCR and Western blotting (*n* = 5). (**D**) *PPFIA4* expression in gastric mucosa of *H*. *pylori*–infected patients (*n* = 131) and uninfected donors (*n* = 50) was compared. (**E**) The correlation between *PPFIA4* expression and *H*. *pylori* colonization in gastric mucosa of *H*. *pylori*–infected patients was analyzed. (**F**) *PPFIA4* expression in gastric mucosa of *H*. *pylori*–infected patients with mild (*n* = 34), moderate (*n* = 45), or severe inflammation (*n* = 24) and with normal gastric histopathology (*n* = 28) was compared. Data are presented as mean ± SEM. Statistics: 1-way ANOVA (**A**–**C** and **F**), unpaired 2-tailed *t* test (**D**), and 2-tailed Pearson’s correlation test (**E**). **P < 0.01 for groups connected by horizontal lines.

To evaluate the involvement of GEC-derived PPFIA4 in *H*. *pylori* infection and *H*. *pylori*–associated gastritis, we first performed thiol(SH)-linked alkylation for the metabolic sequencing of RNA (SLAM-seq) and microarray analysis of *H*. *pylori*–infected and uninfected AGS cells that have been used to investigate the effects of *H*. *pylori* infection on GECs (14) and obtained 300 top significantly upregulated differentially expressed genes (DEGs). We identified 9 significantly upregulated DEGs by overlapping analysis, and finally identified 4 significantly upregulated DEGs with unknown function in the stomach (Supplemental Figure 1A; supplemental material available online with this article; https://doi.org/10.1172/JCI193848DS1). Second, we analyzed scRNA-seq data of 18 patients pathologically diagnosed with gastric lesions from the Gene Expression Omnibus (GEO) (GSE249874) and identified 5 clusters of GECs. SCENIC (Single-Cell Regulatory Network Inference and Clustering; https://github.com/aertslab/SCENIC; commit ID 7a74341) analysis of these GEC clusters found 3 key regulons that are mostly associated with *H*. *pylori*–associated gastritis, and protein–protein interaction analysis identified that *PPFIA4* has the closest relation with these 3 regulons (Supplemental Figure 1A). By overlapping the DEGs enriched from the data of SLAM-seq and microarray as well as the key genes enriched from scRNA-seq data, *PPFIA4* was the only candidate (Supplemental Figure 1A), suggesting a potential role of GEC-derived PPFIA4 induced by *H*. *pylori* infection in *H*. *pylori*–associated gastritis. Furthermore, *H*. *pylori* infection induced AGS cells to express PPFIA4 via bacterium-cell contact (Supplemental Figure 1, B and C), and *H*. *pylori*–infected AGS cells (Figure 1A) as well as human (Figure 1B) or mouse (Figure 1C) primary GECs increased PPFIA4 in both infection dose- and time-dependent manners. Analysis of *PPFIA4* expression in human primary gastric mucosa of 131 *H*. *pylori*–infected patients and 50 uninfected individuals confirmed that, compared with uninfected donors, *PPFIA4* expression was higher in gastric mucosa of *H*. *pylori*–infected patients (Figure 1D), whose expression was positively correlated with *H*. *pylori* colonization (Figure 1E). Also, higher *PPFIA4* expression was strongly associated with more severe gastritis (Figure 1F). We also confirmed that *H*. *pylori* infection induced human and mouse primary gastric mucosa to increase PPFIA4 in both infection dose- and time-dependent manners (Supplemental Figure 1, D and E). Collectively, these data demonstrate that PPFIA4 is increased in GECs from gastric mucosa of *H*. *pylori*–infected patients and mice.

### H. pylori induces GECs to express PPFIA4 via the CagA/AP1 pathway.

**Figure 2:**
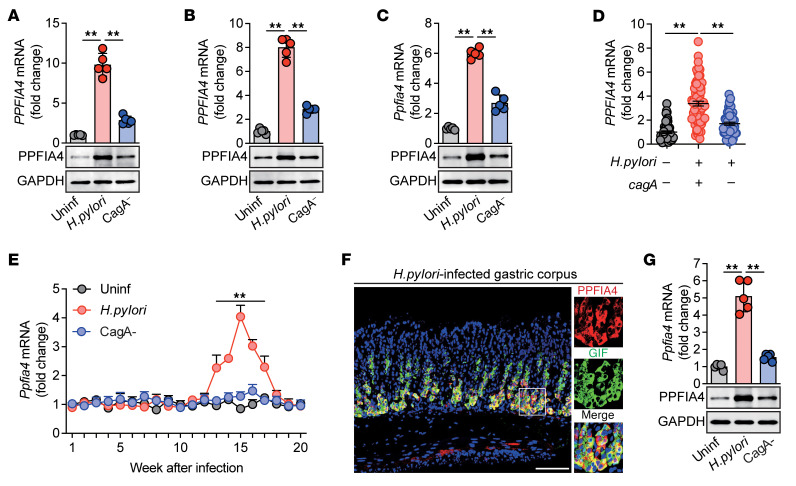
*H*. *pylori* induces GECs to express PPFIA4 via CagA. (**A**–**C**) *PPFIA4/Ppfia4* expression and PPFIA4 protein in *H*. *pylori*–infected, CagA^–^ *H*. *pylori*–infected, and uninfected AGS cells (**A**), and human (**B**) and mouse (**C**) primary GECs (MOI = 100, 24 hours) were analyzed by real-time PCR and Western blotting (*n* = 5). (**D**) *PPFIA4* expression in gastric mucosa of *cagA*^+^ *H*. *pylori*–infected (*n* = 74), *cagA*^–^ *H*. *pylori*–infected (*n* = 57), and uninfected donors (*n* = 50) was compared. (**E**) Dynamic changes of *Ppfia4* expression in gastric mucosa of *H*. *pylori*–infected, CagA^–^ *H*. *pylori*–infected, and uninfected mice (*n* = 5 per group per time point). **P < 0.01 for *H*. *pylori*–infected mice compared with CagA^–^ *H*. *pylori*–infected mice. (**F**) Immunofluorescence analysis showing PPFIA4-expressing GIF^+^ cells in gastric corpus of *H*. *pylori*–infected WT mice 15 weeks p.i. Scale bar: 100 μm. (**G**) *Ppfia4* expression and PPFIA4 protein in *H*. *pylori*–infected, CagA^–^ *H*. *pylori*–infected, and uninfected mouse gastric organoids (MOI = 100, 24 hours) were analyzed by real-time PCR and Western blotting (*n* = 5). Data are presented as mean ± SEM. Statistics: unpaired 2-tailed *t* test (**A**–**D** and **G**) and 2-way ANOVA with multiple comparisons (**E**). **P < 0.01 for groups connected by horizontal lines.

Virulence factor CagA is strongly linked to *H*. *pylori*–associated pathology (15). Notably, compared with *cagA*-knockout mutant *H*. *pylori* (CagA^–^ *H*. *pylori*)–infected counterparts, *H*. *pylori*–infected AGS cells (Figure 2A) and human (Figure 2B) or mouse (Figure 2C) primary GECs showed significantly increased PPFIA4. Similar observations were made when using other human GEC lines (Supplemental Figure 2, A–C). Furthermore, *PPFIA4* expression was significantly higher in *cagA*^+^ patients than that in *cagA*^–^ individuals (Figure 2D). Consistent with our findings in humans, *Ppfia4* expression was also detected in *H*. *pylori*– but not CagA^–^ *H*. *pylori*–infected mice, reaching a peak 15 weeks post-infection (p.i.) (Figure 2E). Next, *H*. *pylori* infection induced human and mouse primary gastric mucosa to increase PPFIA4 in a CagA-dependent manner (Supplemental Figure 2, D and E), and PPFIA4 was higher in gastric mucosa of *cagA*^+^ *H*. *pylori*–infected patients and mice, compared with that in *cagA*^–^ patients and CagA^–^ *H*. *pylori*–infected counterparts, respectively (Supplemental Figure 2, F and G). Notably, PPFIA4 was only detected in gastric intrinsic factor (GIF^+^) chief cells in the gastric corpus of *H*. *pylori*–infected mice 15 weeks p.i. (Figure 2F and Supplemental Figure 2, H and I). Importantly, in established mouse gastric organoids from gastric corpus (Supplemental Figure 2J), *H*. *pylori* infection induced PPFIA4 in a CagA-dependent manner, and PPFIA4 was only expressed in GIF^+^ cells in these infected organoids (Figure 2G and Supplemental Figure 2K). Similar observations were made using human gastric organoids infected with *H*. *pylori* (Supplemental Figure 2, L–N).

**Figure 3:**
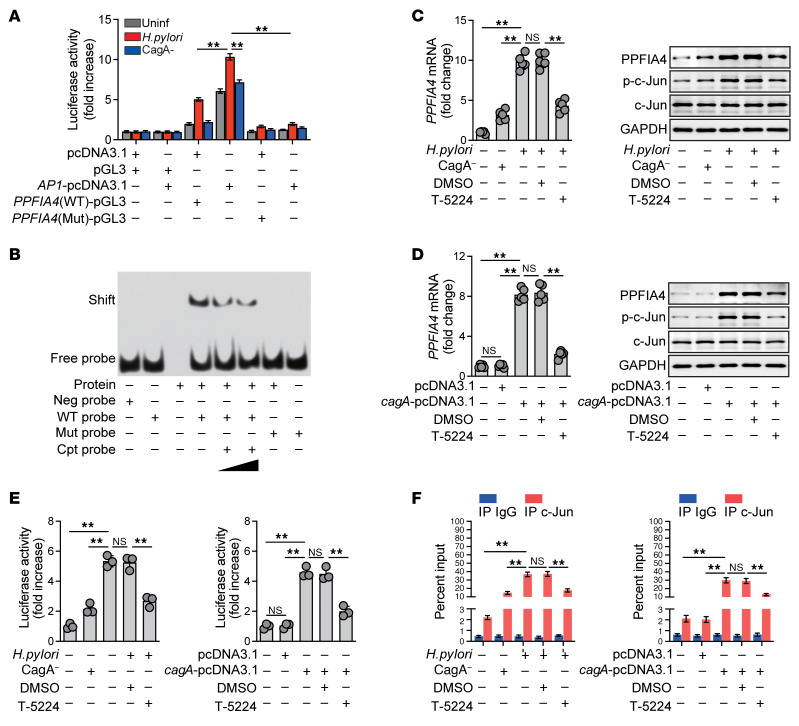
*H*. *pylori* induces GECs to express PPFIA4 via the CagA/AP1 pathway. (**A**) AGS cells were cotransfected with luciferase reporter constructs containing the *PPFIA4*-luc promoter with WT or mutant AP1 binding site [*PPFIA4*(WT)-pGL3 or *PPFIA4*(Mut)-pGL3] or pGL3 and/or constructs expressing AP1 (*AP1*-pcDNA3.1) or pcDNA3.1 for 24 hours. Luciferase activity was measured to assess *PPFIA4* promoter activity after *H*. *pylori* or CagA^–^ *H*. *pylori* infection (MOI = 100) for 24 hours (*n* = 3). (**B**) EMSA for AP1 binding to the AP1 binding site of the *PPFIA4* promoter was performed. Neg, negative; Mut, mutant; Cpt, competitor. (**C** and **D**) AGS cells were pretreated with or without AP1 inhibitor T-5224 and infected with *H*. *pylori* or CagA^–^ *H*. *pylori* (MOI = 100) for 24 hours (**C**) or transfected with plasmid pcDNA3.1 or *cagA*-pcDNA3.1 for 48 hours (**D**). *PPFIA4* expression and PPFIA4, c-Jun, and p-c-Jun proteins were analyzed by real-time PCR and Western blotting (*n* = 5). (**E** and **F**) Luciferase reporter assay (**E**) and ChIP assay (**F**) of AGS cells infected with *H*. *pylori* (pretreated with or without AP1 inhibitor T-5224) or CagA^–^ *H*. *pylori* or AGS cells transfected with plasmid *cagA*-pcDNA3.1 (pretreated with or without AP1 inhibitor T-5224) or pcDNA3.1 (*n* = 3). Data are presented as mean ± SEM. Statistics: unpaired 2-tailed *t* test (**A** and **C**–**F**). **P < 0.01, n.s. *P* > 0.05 for groups connected by horizontal lines.

To explore the underlying mechanism of PPFIA4 induction in GECs by *H*. *pylori*, we analyzed *PPFIA4*/*Ppfia4* promoters (–2,000/1,000) and identified 16 overlapping transcription factors in human and mouse (Supplemental Figure 3A). Subsequent luciferase reporter experiments identified only 1 transcription factor, activator protein-1 (AP1), that mediated *PPFIA4* gene transcription in response to *H*. *pylori* infection in a CagA-dependent manner (Supplemental Figure 3B). To investigate the binding of AP1 to the *PPFIA4* promoter, we generated a series of *PPFIA4*-luc promoter constructs of varying lengths and performed luciferase reporter experiments. It was clear that the *PPFIA4* promoter (–1,750/–1,501) region mediated transcription in response to *H*. *pylori* infection in a CagA-dependent manner (Supplemental Figure 3C). Further analysis showed that both human and mouse *PPFIA4*/*Ppfia4* promoters contained a conserved AP1 binding site (Supplemental Figure 3D and Supplemental Table 8). Subsequent luciferase reporter experiments (Figure 3A) and EMSA (Figure 3B) showed that, compared with CagA^–^ *H*. *pylori* infection, *H*. *pylori* infection significantly increased AP1 binding to the *PPFIA4* promoter in AGS cells via this conserved AP1 binding site. Furthermore, PPFIA4 and c-Jun, a subunit of AP1, were predominantly increased and phosphorylated in AGS cells after being infected with *H*. *pylori*, which was abolished when pretreated with the AP1 inhibitor T-5224 (Figure 3C) or with the CagA EPIYA motif phosphorylation inhibitor PP2 (16) (Supplemental Figure 3E). Additionally, increased PPFIA4 and c-Jun phosphorylation was found in AGS cells transfected with *cagA*-pcDNA3.1 compared with those transfected with the vector (pcDNA3.1) (Figure 3D). Subsequently, luciferase reporter experiments (Figure 3E) and ChIP assays (Figure 3F and Supplemental Figure 3F) showed that, compared with CagA^–^ *H*. *pylori* infection or pcDNA3.1 transfection, *H*. *pylori* infection and *cagA*-pcDNA3.1 transfection significantly increased AP1 binding to the *PPFIA4* promoter in AGS cells, which was abolished when pretreated with T-5224. Collectively, these findings demonstrate that CagA-mediated AP1 activation induces PPFIA4 in *H*. *pylori*–infected GECs.

### H. pylori and IL-33 induce PPFIA4 synergistically.

**Figure 4:**
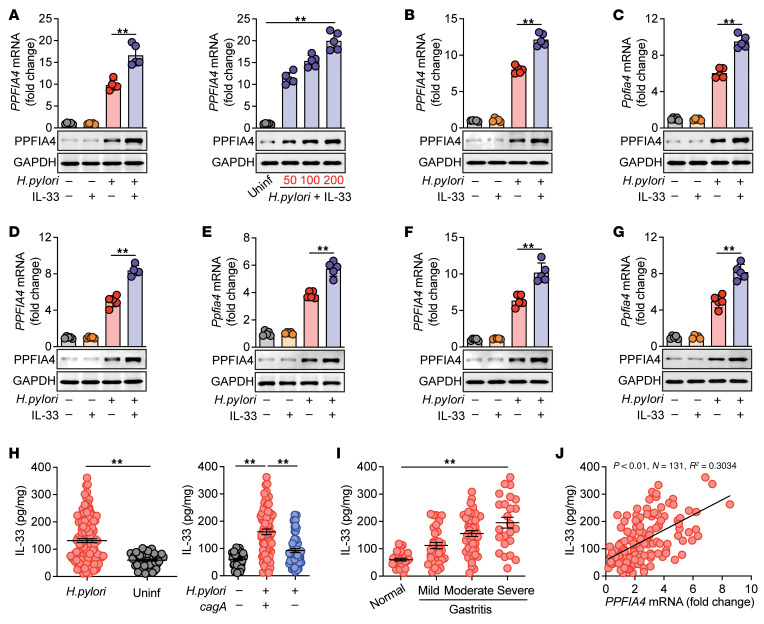
*H*. *pylori* and IL-33 induce PPFIA4 synergistically. (**A**) *PPFIA4* expression and PPFIA4 protein in AGS cells infected with *H*. *pylori* (MOI = 100) in the presence or absence of IL-33 (100 ng/mL) (24 hours) or in AGS cells infected with *H*. *pylori* (MOI = 100) in the presence of IL-33 (50, 100, and 200 ng/mL) (24 hours) were analyzed by real-time PCR and Western blotting (*n* = 5). (**B**–**G**) *PPFIA4/Ppfia4* expression and PPFIA4 protein in human (**B**) and mouse (**C**) primary GECs, in human (**D**) and mouse (**E**) primary gastric mucosa, or in human (**F**) and mouse (**G**) gastric organoids infected with *H*. *pylori* (MOI = 100) in the presence or absence of IL-33 (100 ng/mL) (24 hours) were analyzed by real-time PCR and Western blotting (*n* = 5). (**H**) IL-33 protein in gastric mucosa of *H*. *pylori*–infected (*n* = 131) and uninfected donors (*n* = 50) or in gastric mucosa of *cagA*^+^ *H*. *pylori*–infected (*n* = 74), *cagA*^–^ *H*. *pylori*–infected (*n* = 57), and uninfected donors (*n* = 50) was compared. (**I**) IL-33 protein in gastric mucosa of *H*. *pylori*–infected patients with mild (*n* = 34), moderate (*n* = 45), or severe inflammation (*n* = 24) and with normal gastric histopathology (*n* = 28) was compared. (**J**) The correlation between *PPFIA4* expression and IL-33 protein in gastric mucosa of *H*. *pylori*–infected patients was analyzed. Data are representative of 2 independent experiments. Data are presented as mean ± SEM. Statistics: unpaired 2-tailed *t* test (**A**–**H**), 1-way ANOVA (**I**), and 2-tailed Pearson’s correlation test (**J**). **P < 0.01 for groups connected by horizontal lines.

**Figure 5:**
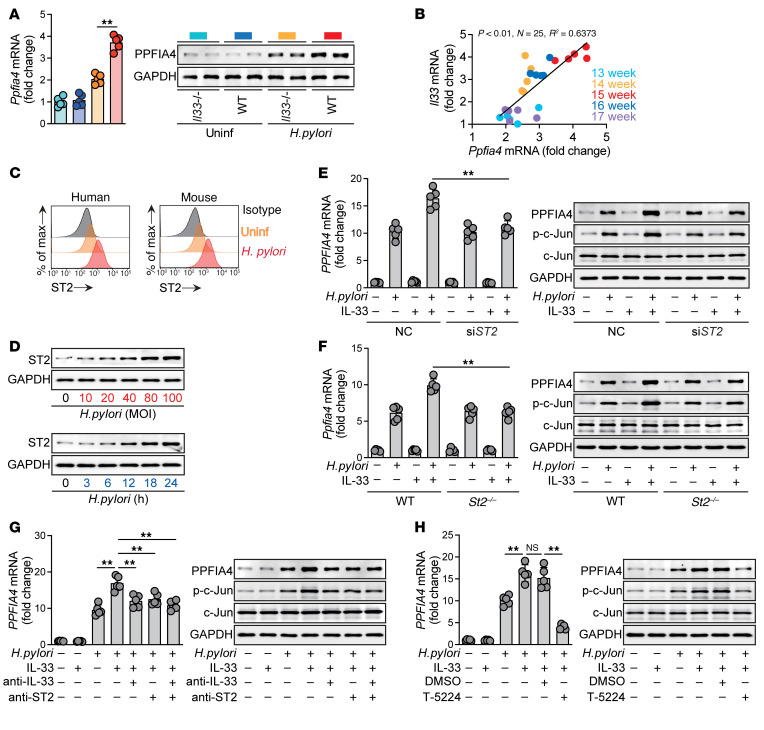
*H*. *pylori* and IL-33 induce PPFIA4 synergistically. (**A**) *Ppfia4* expression and PPFIA4 protein in gastric mucosa of *H*. *pylori*–infected WT and *Il33*^–/–^ mice 15 weeks p.i. were compared (*n* = 5). (**B**) The correlation between *Ppfia4* expression and *Il33* expression in gastric mucosa of *H*. *pylori*–infected mice 13, 14, 15, 16, and 17 weeks p.i. was analyzed. (**C**) ST2 in *H*. *pylori*–infected human or mouse primary GECs (MOI = 100, 24 hours) was analyzed by flow cytometry. (**D**) ST2 in *H*. *pylori*–infected AGS cells at different time points (MOI = 100) or with different MOIs (24 hours) was analyzed by Western blotting. (**E**–**H**) *PPFIA4/Ppfia4* expression and PPFIA4, c-Jun, and p-c-Jun proteins in si*ST2* or siNC pretreated AGS cells (**E**), primary GECs from uninfected WT and *St2*^–/–^ mice (**F**), AGS cells pretreated with anti–IL-33 and/or anti-ST2 Abs (**G**), or AGS cells pretreated with or without AP1 inhibitor T-5224 (**H**) infected with *H*. *pylori* (MOI = 100) in the presence or absence of IL-33 (100 ng/mL) (24 hours) were analyzed by real-time PCR and Western blotting (*n* = 5). Data are presented as mean ± SEM. Statistics: unpaired 2-tailed *t* test (**A** and **E**–**H**) and 2-tailed Pearson’s correlation test (**B**). **P < 0.01, n.s. *P* > 0.05 for groups connected by horizontal lines.

Immune molecules, including cytokines with proinflammatory properties, play critical roles in *H*. *pylori*–associated diseases (17). Screening analysis revealed that only IL-33 exerted a synergistic effect on PPFIA4 induction in a dose-dependent manner (Figure 4A and Supplemental Figure 4A). Similar observations were made using human (Figure 4B) and mouse (Figure 4C) primary GECs, human (Figure 4D) and mouse (Figure 4E) primary gastric mucosa, as well as human (Figure 4F) and mouse (Figure 4G) gastric organoids infected with *H*. *pylori* in the presence or absence of IL-33. However, IL-33 had no synergistic effect on PPFIA4 induction in AGS cells infected with CagA^–^ *H*. *pylori* (Supplemental Figure 4B). Furthermore, IL-33 was higher in gastric mucosa of *cagA*^+^ *H*. *pylori*–infected patients (Figure 4H), and higher IL-33 was strongly associated with more severe gastritis (Figure 4I). Importantly, IL-33 positively correlated with *PPFIA4* expression in gastric mucosa of 131 *H*. *pylori*–infected patients (Figure 4J). Similar observations were made when analyzing *IL33* expression in these samples (Supplemental Figure 4, C–E). Most importantly, this observation was confirmed in vivo in gastric mucosa of *H*. *pylori*–infected WT and *Il33*^–/–^ mice, as we found decreased PPFIA4 in *Il33*^–/–^ mice in the context of *H*. *pylori* infection (Figure 5A) but not in the context of CagA^–^ *H*. *pylori* infection (Supplemental Figure 4F). *Ppfia4* expression was found to be positively correlated with *Il33* expression in gastric mucosa of *H*. *pylori*–infected mice (Figure 5B). Together, these results demonstrate that *H*. *pylori* and IL-33 synergistically induce PPFIA4 even though PPFIA4 expression is not IL-33 dependent.

Given the critical importance of the ST2 receptor in IL-33 signaling (18), we next analyzed the effect of increased ST2 expression on human and mouse primary GECs infected with *H*. *pylori* (Figure 5C and Supplemental Figure 4G) and found that *H*. *pylori*–infected AGS cells increased ST2 in both infection dose- and time-dependent manners (Figure 5D and Supplemental Figure 4H). Furthermore, the induction of PPFIA4 and phosphorylation of c-Jun by *H*. *pylori* and IL-33 were attenuated when the IL-33–ST2 interaction was abolished in either *ST2* siRNA-treated AGS cells (Figure 5E) or mouse primary GECs from *St2^–/–^* mice (Figure 5F) or when the IL-33–ST2 interaction was blocked with anti–IL-33 and/or anti-ST2 antibodies (Figure 5G). Additionally, increased PPFIA4 and c-Jun phosphorylation in AGS cells induced by *H*. *pylori* and IL-33 could be abolished when pretreated with the AP1 inhibitor T-5224 (Figure 5H). Collectively, these results demonstrate that *H*. *pylori* infection induces ST2 expression on GECs, which augments the synergistic effects of *H*. *pylori* and IL-33 on PPFIA4 induction via activating AP1.

### PPFIA4 increases gastritis by promoting G-MDSC accumulation via CXCL3 during H. pylori infection.

**Figure 6:**
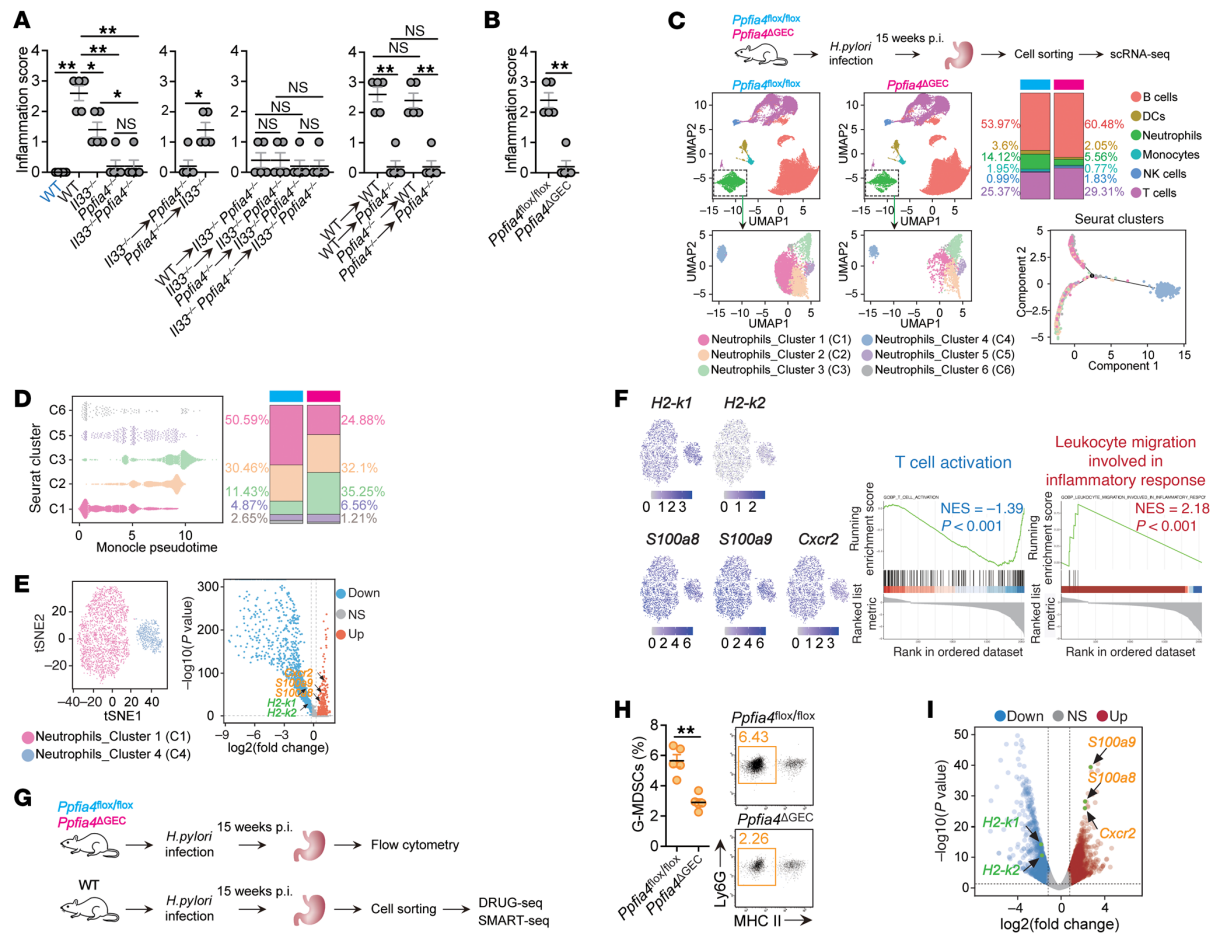
PPFIA4 increases gastritis by promoting G-MDSC accumulation during *H*. *pylori* infection. (**A** and **B**) The histological scores of inflammation in gastric mucosa of uninfected WT mice (blue); *H*. *pylori*–infected WT, *Il33*^–/–^, *Ppfia4*^–/–^, and *Il33*^–/–^*Ppfia4*^–/–^ mice; and BM chimera mice (**A**) or in gastric mucosa of *H*. *pylori*–infected *Ppfia4*^ΔGEC^ mice and *Ppfia4*^fl/fl^ littermates (**B**) 15 weeks p.i. were compared (*n* = 5). (**C**) Schematic showing the protocol for scRNA-seq; UMAP plots showing the annotation and color codes for immune cell types among CD45^+^ cells from gastric mucosa of *H*. *pylori*–infected *Ppfia4*^ΔGEC^ mice and *Ppfia4*^fl/fl^ littermates 15 weeks p.i.; immune cell level was compared (*n* = 3). UMAP plots showing that neutrophils were separated into 6 distinct clusters; pseudotime trajectory analysis showing potential trajectories of neutrophil clusters (C1–C6) during *H*. *pylori* infection. (**D**) Pseudotime analysis of neutrophil clusters; neutrophil cluster level was compared (*n* = 3). (**E**) t-SNE visualization of 2 distinct neutrophil clusters (C1 and C4); volcano plot showing DEGs in C1 versus C4. (**F**) t-SNE plots showing the expression of the indicated genes in C1 versus C4; GSEA of enriched pathways in C1 versus C4. (**G**) Schematic showing the protocol for flow cytometry, DRUG-seq, and SMART-seq. (**H**) Statistical analysis and representative data of G-MDSCs (G-MDSCs in CD45^+^ cells) in gastric mucosa of *H*. *pylori*–infected *Ppfia4*^ΔGEC^ mice and *Ppfia4*^fl/fl^ littermates 15 weeks p.i. (*n* = 5). (**I**) Volcano plot showing DEGs between G-MDSCs and MHCII^+^ neutrophils in gastric mucosa of *H*. *pylori*–infected WT mice 15 weeks p.i. by DRUG-seq (*n* = 3). Data are presented as mean ± SEM. Statistics: Mann-Whitney *U* test (**A** and **B**) and unpaired 2-tailed *t* test (**H**). *P < 0.05, **P < 0.01, n.s. *P* > 0.05 for groups connected by horizontal lines.

To evaluate the effects of PPFIA4 in *H*. *pylori*–associated gastritis, we compared gastric inflammation among WT, *Il33*^–/–^, *Ppfia4*^–/–^, and *Il33*^–/–^*Ppfia4*^–/–^ mice 15 weeks p.i. and found that knocking out *Il33* and/or *Ppfia4* effectively reduced gastric inflammation when compared with that in WT mice; this was more pronounced when PPFIA4 was knocked out in *Ppfia4*^–/–^ and *Il33*^–/–^*Ppfia4*^–/–^ mice compared with in *Il33*^–/–^ mice (Figure 6A and Supplemental Figure 5A). To exclude the possibility that immune cells themselves possibly express PPFIA4 that contributes to *H*. *pylori*–associated gastritis, we generated BM chimera mice to determine the contribution of BM-derived or non-BM-derived PPFIA4 to gastric inflammation. First, *Il33*^–/–^ BM into *Ppfia4*^–/–^ mice showed significantly reduced inflammation when compared with that in *Ppfia4*^–/–^ BM into *Il33*^–/–^ mice, suggesting that BM-derived IL-33 and non-BM-derived PPFIA4 contribute to the increased inflammation (Figure 6A). Next, to formally exclude the possibility that BM-derived and non-BM-derived cells may also contribute PPFIA4 and IL-33 in gastric mucosa, respectively, we generated other BM chimera mouse groups (*Il33*^–/–^ BM, *Ppfia4*^–/–^ BM, and *Il33*^–/–^*Ppfia4*^–/–^ BM into *Il33*^–/–^*Ppfia4*^–/–^ mice). We did not detect a role of potential BM-derived IL-33 (*Ppfia4*^–/–^ BM into *Il33*^–/–^*Ppfia4*^–/–^ mice) or BM-derived PPFIA4 (*Il33*^–/–^ BM into *Il33*^–/–^*Ppfia4*^–/–^ mice) in gastric inflammation in our scenarios (Figure 6A). Finally, compared with *Ppfia4*^–/–^ BM into WT mice, WT BM into *Ppfia4*^–/–^ mice was not able to correct the increased gastric inflammation, again indicating that the defect is associated with non-BM-derived PPFIA4 (Figure 6A). Collectively, using these BM chimera mice, we concluded that non-BM-derived PPFIA4 was largely responsible for gastric inflammation during *H*. *pylori* infection (Figure 6A and Supplemental Figure 5A). To confirm the contribution of GEC-derived PPFIA4 to *H*. *pylori*–associated gastritis, as PPFIA4 was almost exclusively detected in GIF^+^ chief cells (Figure 2F), we generated a *Ppfia4* GEC-specific knockout mouse (*Ppfia4*^ΔGEC^) with normal gastric mucosa and GEC profiles and found that, during *H*. *pylori* infection, gastric inflammation was significantly decreased in *Ppfia4*^ΔGEC^ mice (Figure 6B and Supplemental Figure 5, B–D). Collectively, our data demonstrate that GEC-derived PPFIA4 promotes *H*. *pylori*–associated gastritis.

To investigate *H*. *pylori*–associated gastritis regulated by GEC-derived PPFIA4, we performed scRNA-seq. After annotating all cells based on known markers (Supplemental Figure 6A), lack of PPFIA4 in GECs led to maximum decreased gastric neutrophils, from 14.12% of all CD45^+^ cells in *Ppfia4*^fl/fl^ littermates to 5.56% of all CD45^+^ cells in *Ppfia4*^ΔGEC^ mice (Figure 6C). Neutrophils were analyzed and divided into 6 clusters (Figure 6C and Supplemental Figure 6B), and cell pseudotime trajectory analysis revealed a key node in the differentiation process of neutrophils that was closely related to the conversion of neutrophil identity in *H*. *pylori*–associated gastritis (Figure 6C). No changes in Neutrophils_Cluster 4 (C4), a group with high-differentiation phenotype independent from other Neutrophils_Clusters, were found between *Ppfia4*^ΔGEC^ mice and *Ppfia4*^fl/fl^ littermates (Supplemental Figure 6C). Further pseudotime analysis of the other Neutrophils_Clusters revealed that Neutrophils_Cluster 1 (C1) was mostly associated with *H*. *pylori*–associated gastritis with maximum decrease from 50.59% of all neutrophils in *Ppfia4*^fl/fl^ littermates to 24.88% of all neutrophils in *Ppfia4*^ΔGEC^ mice (Figure 6D). Compared with C4, genes associated with antigen presentation and T cell activation, including *H2-k1* and *H2-k2*, significantly decreased in C1; however, genes associated with neutrophil chemotaxis and inflammatory response, including *Cxcr2*, *S100a8*, and *S100a9*, significantly increased in C1 (Figure 6, E and F, and Supplemental Figure 6, D–F).

To confirm scRNA-seq findings and to analyze myeloid cells precisely, we performed multiple-color flow cytometry (Figure 6G). Interestingly, *Ppfia4*^ΔGEC^ mice only showed decreased G-MDSCs in gastric mucosa (Figure 6H and Supplemental Figure 7) but not BM, blood, or spleen (Supplemental Figure 8) G-MDSCs. These results were confirmed by BM chimera experiments in which non-BM-derived PPFIA4 was largely responsible for the increased gastric G-MDSCs during *H*. *pylori* infection (Supplemental Figure 9, A–C). Immunofluorescence microscopy showed lower gastric LyG^+^MHCII^–^ neutrophil infiltration in *Ppfia4*^ΔGEC^ mice (Supplemental Figure 9D). To investigate the nature of these G-MDSCs, we sorted gastric G-MDSCs from WT mice to perform digital RNA with perturbation of genes sequencing (DRUG-seq) and switching mechanism at the 5′ end of RNA template sequencing (SMART-seq) (Figure 6G). Compared with MHCII^+^ neutrophils, genes associated with antigen presentation and T cell activation, including *H2-k1* and *H2-k2*, significantly decreased in G-MDSCs; however, genes associated with neutrophil chemotaxis and inflammatory response, including *Cxcr2*, *S100a8*, and *S100a9*, significantly increased in these G-MDSCs (Figure 6I and Supplemental Figure 10), suggesting that, similar to C1 in scRNA-seq data, such G-MDSCs with potential functions of promoting inflammation and inhibiting T cell activation accumulate in gastric mucosa during *H*. *pylori* infection.

**Figure 7:**
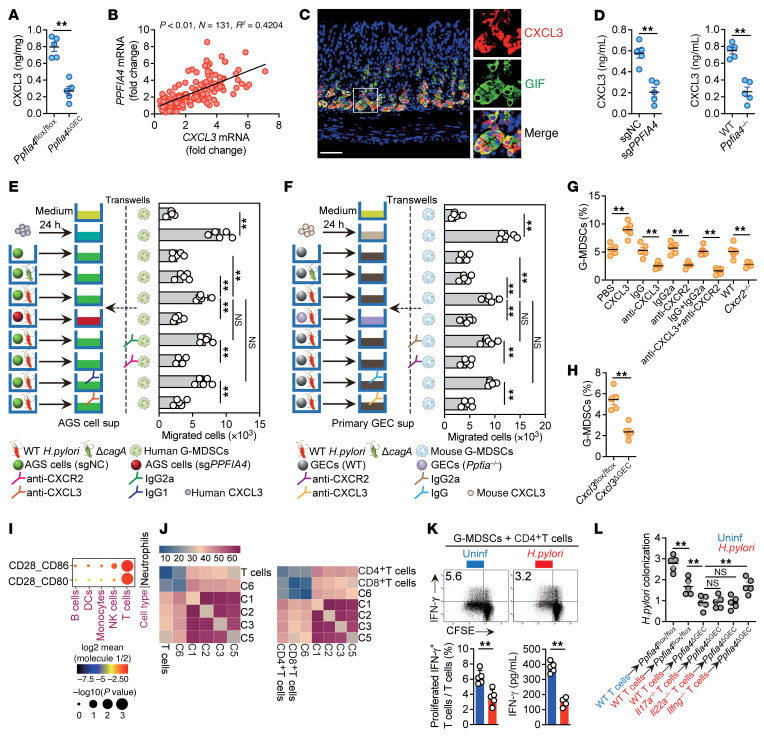
PPFIA4 increases gastritis by promoting G-MDSC accumulation via CXCL3 during *H*. *pylori* infection. (**A**) CXCL3 in gastric mucosa of *H*. *pylori*–infected *Ppfia4*^ΔGEC^ mice and *Ppfia4*^fl/fl^ littermates 15 weeks p.i. was compared (*n* = 5). (**B**) The correlation between *PPFIA4* and *CXCL3* expression in gastric mucosa of *H*. *pylori*–infected patients was analyzed. (**C**) Immunofluorescence analysis showed CXCL3-expressing GIF^+^ GECs in gastric mucosa of *H*. *pylori*–infected WT mice 15 weeks p.i. Scale bar: 100 μm. (**D**) CXCL3 in sg*PPFIA4*-modified or sgNC-modified AGS cells and primary GECs from uninfected WT and *Ppfia4*^–/–^ mice stimulated with *H*. *pylori* (MOI = 100, 24 hours) was analyzed by ELISA (*n* = 5). (**E** and **F**) Human (**E**) and mouse (**F**) G-MDSC migrations were assessed by transwell assays and statistically analyzed (*n* = 5). sup, supernatant. (**G** and **H**) The G-MDSC level (G-MDSCs in CD45^+^ cells) in gastric mucosa of *H*. *pylori*–infected WT mice injected with CXCL3 or anti-CXCL3/CXCR2 Abs, *H*. *pylori*–infected WT and *Cxcr2*^–/–^ mice 15 weeks p.i. (**G**), or *H*. *pylori*–infected *Cxcl3*^ΔGEC^ mice and *Cxcl3*^fl/fl^ littermates 15 weeks p.i. (**H**) was compared (*n* = 5). (**I**) Bubble plot representing pathways associated with T cell activation between neutrophils and other immune cell types. (**J**) Spatial proximity enrichment showing relationships between neutrophil clusters and T cells or CD4^+^ T cells/CD8^+^ T cells. (**K**) G-MDSC/T cell cultures were performed and statistically analyzed (*n* = 5). (**L**) T cell adoptive transfers were performed and statistically analyzed (*n* = 5). The bacteria colonization is shown as log_10_(the number of bacterial genomes per nanogram of host genomic DNA) by measuring *H*. *pylori*–specific 16s rDNA. Data are presented as mean ± SEM. Statistics: unpaired 2-tailed *t* test (**A**, **D**–**H**, **K**, and **L**) and 2-tailed Pearson’s correlation test (**B**). **P < 0.01, n.s. *P* > 0.05 for groups connected by horizontal lines.

Chemotaxis plays important roles in myeloid cell migration (19), and analysis revealed that only CXCL3 was decreased in *Ppfia4*^ΔGEC^ mice (Figure 7A and Supplemental Figure 11A). These results were confirmed by BM chimera experiments in which non-BM-derived PPFIA4 was largely responsible for the increased gastric CXCL3 during *H*. *pylori* infection (Supplemental Figure 11, B and C). Furthermore, CXCL3 was higher in gastric mucosa of *cagA*^+^ *H*. *pylori*–infected patients (Supplemental Figure 11, D and F), and higher CXCL3 was strongly associated with more severe gastritis (Supplemental Figure 11, E and G). Importantly, CXCL3 positively correlated with *PPFIA4* expression in gastric mucosa of 131 *H*. *pylori*–infected patients (Figure 7B and Supplemental Figure 11H). Notably, CXCL3 was almost exclusively expressed in GIF^+^ chief cells (Figure 7C), and CXCL3 production from AGS cells and mouse primary GECs was regulated in a PPFIA4-dependent manner (Figure 7D and Supplemental Figure 11I). Also, G-MDSCs expressed high CXCR2, the chemokine receptor for CXCL3 (Supplemental Figure 11, J and K), which was consistent with scRNA-seq and DRUG-seq data (Figure 6, E and I). Chemotaxis assays demonstrated that culture supernatants from *H*. *pylori*–stimulated AGS cells pretreated with nonspecific control sgRNA (sgNC) induced significantly more G-MDSC migration than those from *H*. *pylori*–infected AGS cells pretreated with sg*PPFIA4* or those from CagA^–^ *H*. *pylori*–infected AGS cells pretreated with sgNC. This effect was lost upon pretreatment with neutralizing antibodies against CXCL3 or CXCR2 (Figure 7E). Similarly, culture supernatant collected from *H*. *pylori*–stimulated primary GECs of WT mice also induced significantly more mouse G-MDSC migration than that from *H*. *pylori*–infected primary GECs of *Ppfia4*^–/–^ mice or that from CagA^–^ *H*. *pylori*–infected primary GECs of WT mice. This effect was also lost upon pretreatment with neutralizing antibodies against CXCL3 or CXCR2 (Figure 7F). Furthermore, CXCL3 administration significantly increased G-MDSC accumulation; conversely, neutralizing CXCL3 and/or CXCR2, or CXCR2 knockout in *Cxcr2*^–/–^ mice significantly reduced G-MDSC accumulation (Figure 7G and Supplemental Figure 11L). To confirm the contribution of GEC-derived CXCL3 to gastric G-MDSC accumulation in *H*. *pylori* infection, we generated the *Cxcl3* GEC-specific knockout mouse (*Gif*-*Cre* *Cxcl3*^fl/fl^, hereafter called *Cxcl3*^ΔGEC^) and found that gastric G-MDSC accumulation significantly decreased in *Cxcl3*^ΔGEC^ mice (Figure 7H and Supplemental Figure 11, M and N). Collectively, our data demonstrate that GEC-derived PPFIA4 plays an essential role in attracting gastric G-MDSCs via CXCL3, contributing to *H*. *pylori*–associated gastritis.

To evaluate the function of G-MDSCs during *H*. *pylori* infection, we reanalyzed scRNA-seq data and found that pathways associated with T cell activation were mostly enhanced in neutrophils and T cells (Figure 7I). We found that, among all the Neutrophils_Clusters, C1 had the best associations with T cells, especially CD4^+^ T cells (Figure 7J), together suggesting that these neighboring cell populations (C1 and CD4^+^ T cells) most likely interacted with each other. Therefore, G-MDSCs with similar C1 profiles and CD4^+^ T cells were sorted. The subsequent G-MDSC/CD4^+^ T cell cocultures showed that gastric G-MDSCs from *H*. *pylori*–infected mice induced fewer CD4^+^ T cells in *H*. *pylori*–infected mice to proliferate and produce IFN-γ than those of uninfected counterparts (Figure 7K). These results suggest an inhibiting effect of G-MDSCs on *H*. *pylori*–specific IFN-γ–producing CD4^+^ T cells.

Next, we conducted in vivo adoptive transfer experiments and evaluated bacterial colonization in gastric mucosa 15 weeks p.i. First, transferring CD4^+^ T cells from *H*. *pylori*–infected WT donors (15 weeks p.i.) into *Ppfia4*^fl/fl^ recipients effectively reduced *H*. *pylori* colonization when compared with *Ppfia4*^fl/fl^ recipients receiving CD4^+^ T cells from uninfected WT donors, suggesting *H*. *pylori*–specific CD4^+^ T cells contribute to reduced bacterial colonization. Next, transferring CD4^+^ T cells from *H*. *pylori*–infected WT donors (15 weeks p.i.) into *Ppfia4*^ΔGEC^ recipients effectively reduced *H*. *pylori* colonization when compared with *Ppfia4*^fl/fl^ recipients receiving the same CD4^+^ T cells, suggesting GEC-derived PPFIA4-mediated inhibition of *H*. *pylori*–specific CD4^+^ T cells, leading to increased bacterial colonization. Finally, transferring CD4^+^ T cells from *H*. *pylori*–infected WT donors (15 weeks p.i.) into *Ppfia4*^ΔGEC^ recipients effectively reduced *H*. *pylori* colonization when compared with *Ppfia4*^ΔGEC^ recipients receiving CD4^+^ T cells from *H*. *pylori*–infected *Ifng*^–/–^ donors but not *Il17a*^–/–^ or *Il22*^–/–^ donors, suggesting GEC-derived PPFIA4-mediated inhibition of IFN-γ production in *H*. *pylori*–specific CD4^+^ T cells, leading to increased bacterial colonization (Figure 7L and Supplemental Figure 11O). Overall, these results indicate that GEC-derived PPFIA4 promotes gastric *H*. *pylori* colonization through inhibiting *H*. *pylori*–specific IFN-γ–producing CD4^+^ T cells.

### PPFIA4 promotes MMP1 expression, leading to increased bacterial burden and gastric mucosal damage during H. pylori infection.

**Figure 8:**
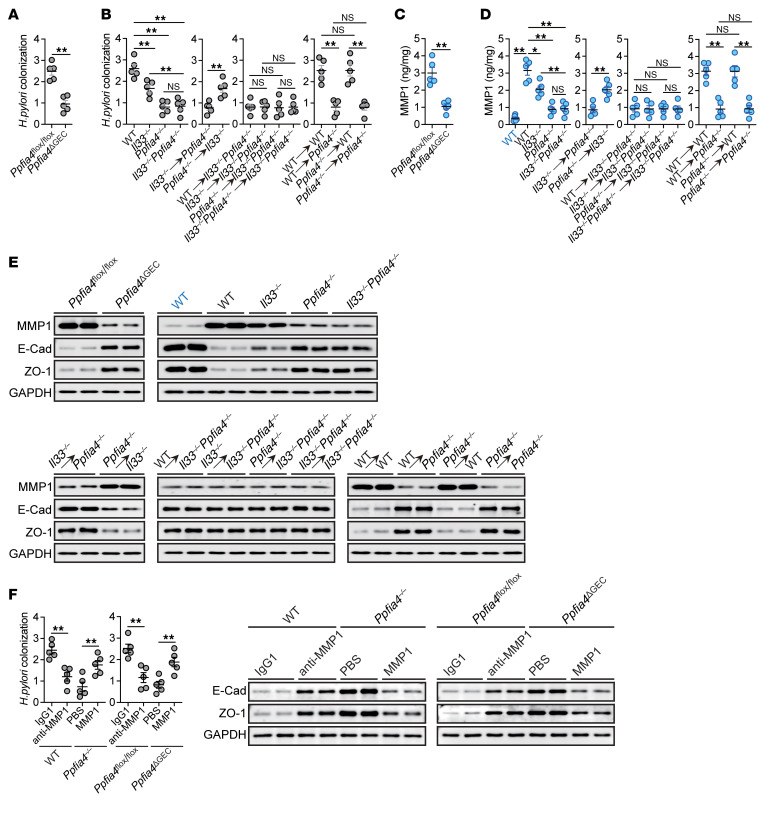
PPFIA4 promotes MMP1 expression leading to increased bacterial burden during *H*. *pylori* infection. (**A** and **B**) The bacteria colonization in gastric mucosa of *H*. *pylori*–infected *Ppfia4*^ΔGEC^ mice and *Ppfia4*^fl/fl^ littermates (**A**) or *H*. *pylori*–infected WT, *Il33*^–/–^, *Ppfia4*^–/–^, and *Il33*^–/–^*Ppfia4*^–/–^ mice and BM chimera mice (**B**) 15 weeks p.i. was analyzed (*n* = 5). (**C**–**E**) MMP1, E-cadherin, and ZO-1 proteins in gastric mucosa of *H*. *pylori*–infected *Ppfia4*^ΔGEC^ mice and *Ppfia4*^fl/fl^ littermates, uninfected WT mice (blue), or *H*. *pylori*–infected WT, *Il33*^–/–^, *Ppfia4*^–/–^, and *Il33*^–/–^*Ppfia4*^–/–^ mice and BM chimera mice 15 weeks p.i. were analyzed (*n* = 5). (**F**) The bacteria colonization and E-cadherin and ZO-1 proteins in gastric mucosa of *H*. *pylori*–infected WT mice or *Ppfia4*^fl/fl^ littermates injected with anti-MMP1 Abs or *H*. *pylori*–infected *Ppfia4*^–/–^ mice or *Ppfia4*^ΔGEC^ mice injected with MMP1 15 weeks p.i. were analyzed (*n* = 5). The bacteria colonization in **A**, **B**, and **F** is shown as log_10_(the number of bacterial genomes per nanogram of host genomic DNA) by measuring *H*. *pylori*–specific 16s rDNA. Data are presented as mean ± SEM. Statistics: unpaired 2-tailed *t* test (**A**–**D** and **F**). **P < 0.01, n.s. *P* > 0.05 for groups connected by horizontal lines.

To confirm the promoting effects of GEC-derived PPFIA4 on *H*. *pylori* colonization, we compared gastric bacterial colonization between *Ppfia4*^ΔGEC^ mice and *Ppfia4*^fl/fl^ littermates 15 weeks p.i. and found that *Ppfia4*^ΔGEC^ mice showed reduced gastric *H*. *pylori* colonization (Figure 8A and Supplemental Figure 12A). These results were confirmed by BM chimera experiments in which non-BM-derived PPFIA4 was largely responsible for the increased *H*. *pylori* colonization (Figure 8B and Supplemental Figure 12B). These data suggest that GEC-derived PPFIA4 increases bacterial burden.

By screening MMPs that contribute to tissue damage as well as β-defensins and Reg3 proteins that contribute to host defense, we found that *Ppfia4*^ΔGEC^ mice only showed decreased MMP1 (Figure 8C and Supplemental Figure 12C), which was confirmed by BM chimera experiments in which non-BM-derived PPFIA4 was largely responsible for the increased gastric MMP1 during *H*. *pylori* infection (Figure 8D and Supplemental Figure 12D). Additionally, *Ppfia4*^ΔGEC^ mice showed decreased E-cadherin and ZO-1 proteins that contribute to both integrity and stability of gastric mucosa, which was confirmed by BM chimera experiments in which non-BM-derived PPFIA4 was largely responsible for these decreases during *H*. *pylori* infection (Figure 8E). Next, neutralizing MMP1 significantly reduced gastric bacterial colonization of WT mice or *Ppfia4*^fl/fl^ littermates; conversely, MMP1 administration significantly increased gastric bacterial colonization of *Ppfia4*^–/–^ or *Ppfia4*^ΔGEC^ mice (Figure 8F and Supplemental Figure 12E). The changes in E-cadherin and ZO-1 protein levels showed an inverse relationship with MM1 in these samples (Figure 8F). Furthermore, MMP1 was higher in gastric mucosa of *cagA*^+^ *H*. *pylori*–infected patients (Supplemental Figure 12F), and MMP1 positively correlated with *PPFIA4* expression and *H*. *pylori* colonization (Supplemental Figure 12G) in gastric mucosa of 131 *H*. *pylori*–infected patients. Collectively, our data demonstrate that GEC-derived PPFIA4 plays an essential role in promoting MMP1 in gastric mucosa during *H*. *pylori* infection, which contribute to bacterial persistence.

**Figure 9:**
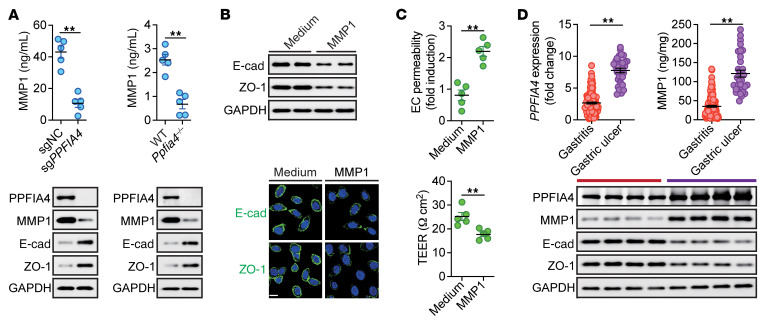
PPFIA4 promotes MMP1 expression, leading to gastric mucosal damage during *H*. *pylori* infection. (**A**) sg*PPFIA4*-modified or sgNC-modified AGS cells and primary GECs from uninfected WT or *Ppfia4*^–/–^ mice were stimulated with *H*. *pylori* (MOI = 100) for 24 hours. MMP1, E-cadherin, and ZO-1 proteins were analyzed by ELISA and Western blotting (*n* = 5). (**B**) E-cadherin and ZO-1 proteins in AGS treated with MMP1 (1 μg/mL, 24 hours) were analyzed by Western blotting and immunofluorescence. Scale bars: 10 μm. (**C**) AGS monolayers were treated with MMP1 (1 μg/mL) for 24 hours. FITC-dextran permeability was determined. TEER measurements were then performed, and TEER values were calculated. (**D**) *PPFIA4* expression and PPFIA4, MMP1, E-cadherin, and ZO-1 proteins in gastric mucosa of patients with gastritis (*n* = 103) or with gastric ulcer (*n* = 32) were analyzed. Data are shown as mean ± SEM. Statistics: unpaired 2-tailed *t* test. **P < 0.01 for groups connected by horizontal lines.

Using in vitro experiments, we showed that PPFIA4 upregulates MMP1, but downregulates E-cadherin and ZO-1, in both AGS cells and mouse primary GECs (Figure 9A and Supplemental Figure 12H). Furthermore, Western blotting and immunofluorescence analysis showed that MMP1 effectively and directly decreased E-cadherin and ZO-1 in AGS cells (Figure 9B and Supplemental Figure 12, I–K). In addition, GEC permeability assay and transepithelial electrical resistance (TEER) measurements showed increased cell permeability and decreased TEER values in MMP1-stimulated AGS cells (Figure 9C). Notably, the expression of PPFIA4 and MMP1 in patients with gastric ulcers was significantly higher than that in patients with gastritis (Figure 9D). Collectively, our data demonstrate that GEC-derived PPFIA4 promotes MMP1 expression, leading to increased bacterial burden and gastric mucosal damage during *H*. *pylori* infection.

### PPFIA4’s SAM1 binds domains of CaMK to the first L27 of CASK during H. pylori infection.

**Figure 10:**
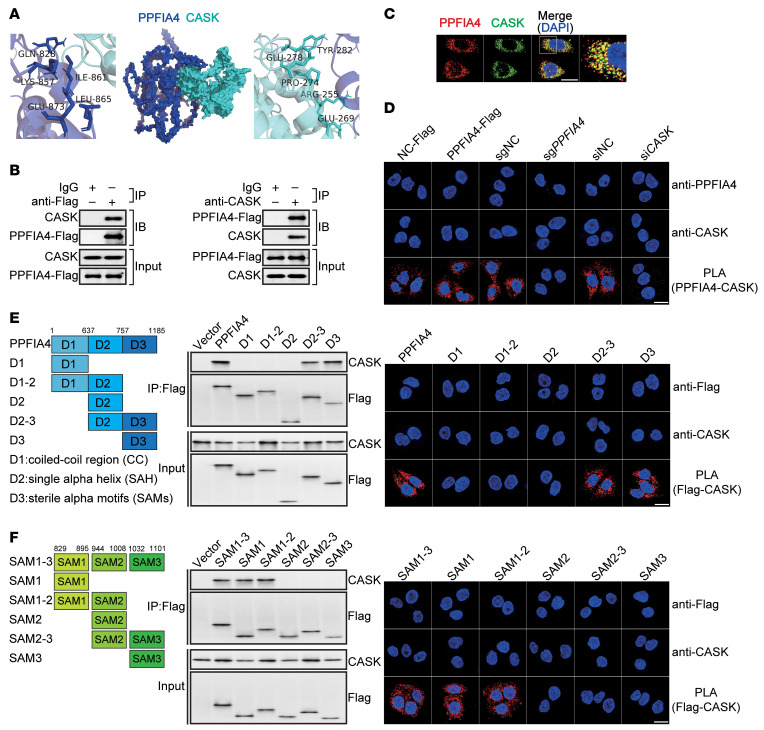
PPFIA4’s SAM1 binds to CASK during *H*. *pylori* infection. (**A**) The optimally predicted protein–protein complex obtained from HADDOCK’s easy interface (https://wenmr.science.uu.nl/haddock2.4/): middle, an overarching schematic of PPFIA4/CASK complex; left, active residues crucial for PPFIA4 binding with CASK; right, active residues crucial for CASK binding with PPFIA4. (**B**) AGS cells expressing PPFIA4-Flag were lysed and immunoprecipitated with anti-Flag or anti-CASK Abs. The IP samples were analyzed by Western blotting. (**C**) Immunofluorescence showed the PPFIA4/CASK colocalization in AGS cells expressing PPFIA4-Flag. Scale bar: 10 μm. (**D**) AGS cells expressing PPFIA4-Flag or NC-Flag were cultured. sg*PPFIA4*- or sgNC-modified AGS cells, and si*CASK* or siNC pretreated AGS cells were stimulated with *H*. *pylori* (MOI = 100) for 24 hours. Proximity ligation assay in AGS cells was performed by using anti-PPFIA4 and anti-CASK Abs. Single Ab only was used as negative control. Red dots represent close relationship between the 2 proteins. Scale bars: 10 μm. (**E** and **F**) Flag-tagged PPFIA4, D1, D1-2, D2, D2-3, and D3 were individually overexpressed in AGS cells (**E**), and Flag-tagged SAM1-3, SAM1, SAM1-2, SAM2, SAM2-3, and SAM3 were individually overexpressed in AGS cells (**F**), then anti-Flag Abs were used for IP. The IP samples were analyzed by Western blotting. Proximity ligation assay was performed using anti-Flag and anti-CASK Abs. Single Ab only was used as negative control. Red dots represent close relationship between the 2 proteins. Scale bar: 10 μm.

PPFIA4’s structure helps it act as a molecular scaffold with multiple binding sites (20). We next performed immunoprecipitation (IP) followed by mass spectrometry (MS) analysis to identify potential PPFIA4 interacting partners using established AGS cells stably expressing the Flag-tagged PPFIA4 (PPFIA4-Flag) (Supplemental Figure 13A). Compared with the control AGS cells, protein network analysis of the MS results identified that CASK was the potential candidate in AGS cells expressing PPFIA4-Flag by overlapping several Gene Ontology (GO) terms (Supplemental Figure 13B), and normalized intensity ratios yielded from MS spectral counts revealed that CASK was an abundant protein (Supplemental Figure 13C). Next, in silico prediction showed several residues between PPFIA4 and CASK likely involved in the interaction of the 2 molecules (Figure 10A and Supplemental Figure 13D). Importantly, we validated the predicted PPFIA4 and CASK interaction as an endogenous PPFIA4/CASK complex by co-IP experiments (Figure 10B). Immunofluorescence microscopy showed that PPFIA4 and CASK were colocalized (Figure 10C and Supplemental Figure 13E), which was supported by the proximity ligation assays showing the physical proximity of PPFIA4 and CASK (Figure 10D and Supplemental Figure 13F).

PPFIA4 contains 3 domains, including the coiled-coil region, single alpha helix, and sterile α motifs (SAMs). To elucidate the binding sites between PPFIA4 and CASK, we first generated 6 truncated PPFIA4 mutants to determine which PPFIA4 domain interacts with CASK. Co-IP experiments showed that only the WT and D3 domain of PPFIA4 bound to CASK and that mutants that do not contain the D3 domain lost the ability to interact with CASK (Figure 10E), which was supported by the proximity ligation assays showing the physical proximity of the D3 domain of PPFIA4 and CASK (Figure 10E and Supplemental Figure 14A). The D3 domain of PPFIA4 contains 3 C-terminal SAMs, which may play key roles during PPFIA4 binding (20). Thus, we generated 6 truncated mutants of PPFIA4 SAMs to determine which SAM of PPFIA4 interacts with CASK. Co-IP experiments showed that only the WT and SAM1 of PPFIA4 bound to CASK and that mutants that do not contain SAM1 lost the ability to interact with CASK (Figure 10F), which was supported by the proximity ligation assays showing the physical proximity of SAM1 of PPFIA4 and CASK (Figure 10F and Supplemental Figure 14B).

**Figure 11:**
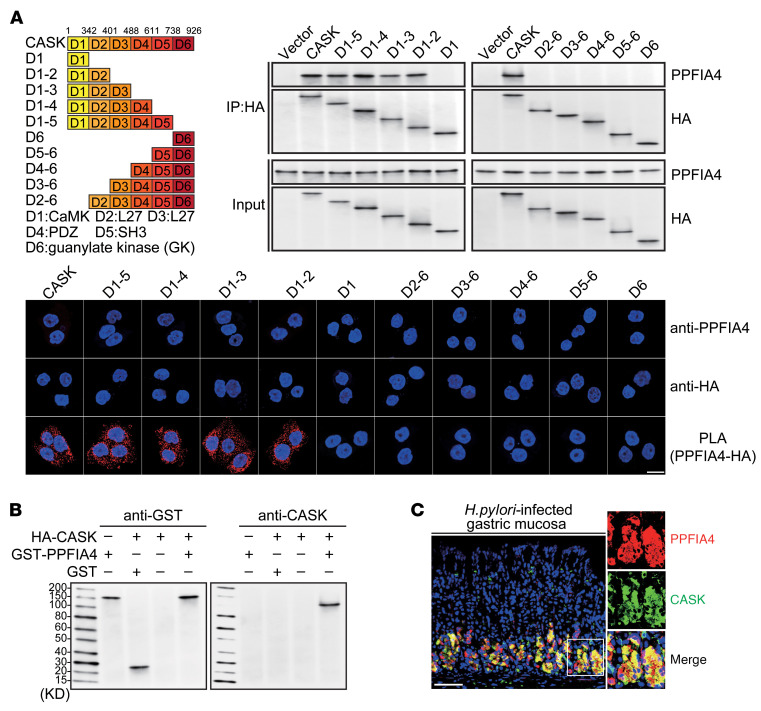
PPFIA4 binds to domains of CaMK to the first L27 of CASK during *H*. *pylori* infection. (**A**) HA-tagged CASK, D1, D1-2, D1-3, D1-4, D1-5, D6, D5-6, D4-6, D3-6, and D2-6 were individually overexpressed in AGS cells, and anti-HA Abs were used for IP. The IP samples were analyzed by Western blotting. Proximity ligation assay was performed using anti-HA and anti-PPFIA4 Abs. Scale bar: 10 μm. (**B**) In vitro binding between HA-CASK and GST-PPFIA4 was analyzed by GST pull-down assays. (**C**) Immunofluorescence showed PPFIA4^+^CASK^+^ cells in gastric mucosa of *H*. *pylori*–infected WT mice 15 weeks p.i. Scale bar: 100 μm.

CASK contains 6 domains, including CaM kinase (CaMK), 2 L27 domains, Postsynaptic density-95/Discs large/Zonula occludens-1, Src homology 3, and guanylate kinase. To determine which of these domains interacts with PPFIA4, we generated 11 truncated CASK mutants. Co-IP experiments showed that only the WT and D1 to D2 domains (domains of CaMK at the first L27) of CASK bound to PPFIA4 and that mutants that do not contain D1 to D2 domains lost the ability to interact with PPFIA4 (Figure 11A), which was supported by the proximity ligation assays showing the physical proximity of D1 to D2 domains of CASK and PPFIA4 (Figure 11A and Supplemental Figure 15). Finally, a robust combination between exogenous GST-PPFIA4 and HA-CASK was observed in the GST pull-down assay in vitro (Figure 11B), and a colocalization of PPFIA4 and CASK was found in *H*. *pylori*–infected gastric mucosa in vivo (Figure 11C). Collectively, our data demonstrate that PPFIA4’s SAM1 binds domains of CaMK to the first L27 of CASK in GECs during *H*. *pylori* infection.

### PPFIA4/CASK promotes NF-κB phosphorylation via interaction with and activation of AKT1 during H. pylori infection.

**Figure 12:**
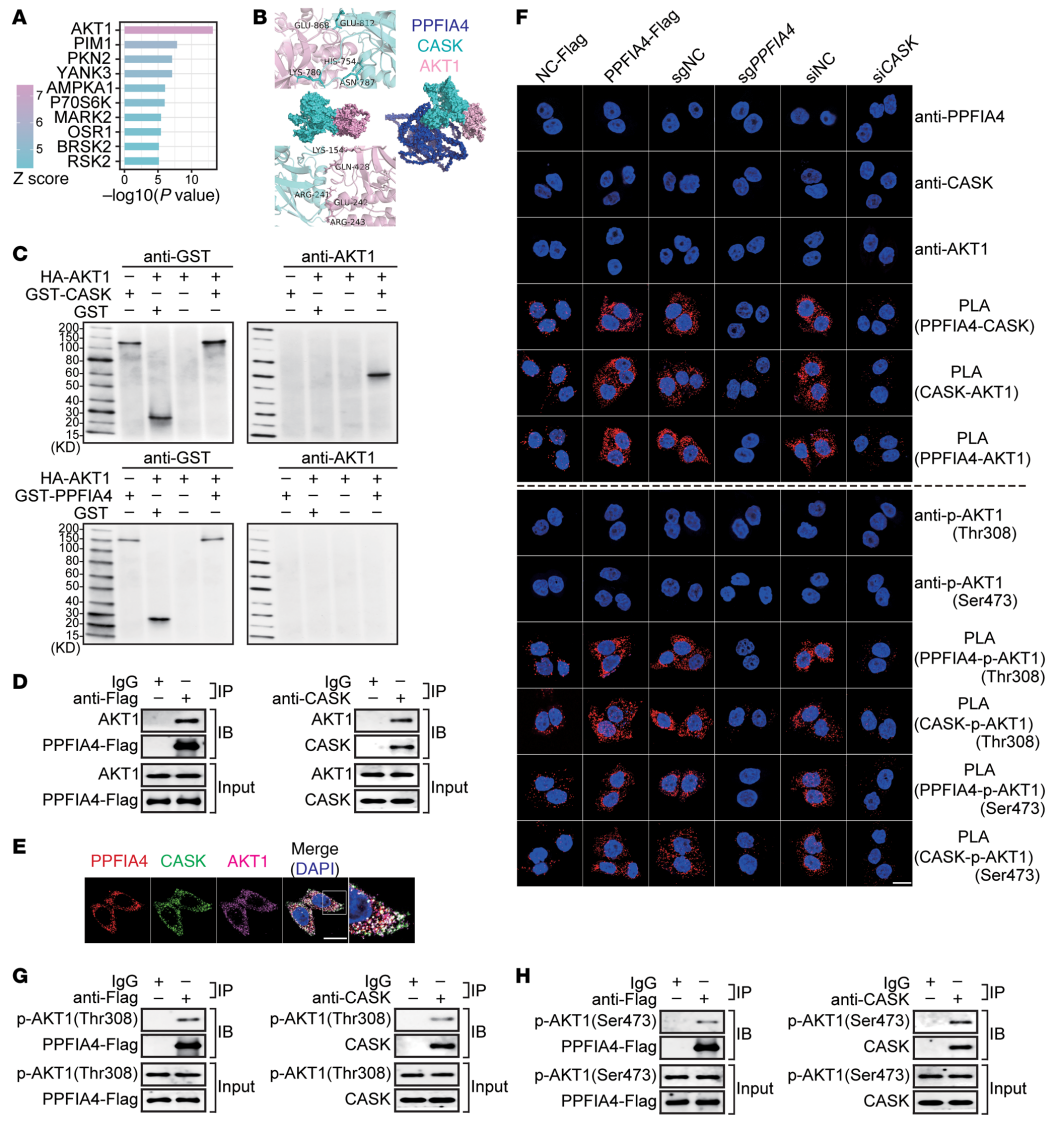
PPFIA4/CASK interacts with and activates AKT1 during *H*. *pylori* infection. (**A**) Compared with AGS cells expressing NC-Flag, the top 10 significantly upregulated kinases are shown in AGS cells expressing PPFIA4-Flag. (**B**) The optimally predicted protein–protein complex obtained from HADDOCK’s easy interface: middle left, an overarching schematic of CASK/AKT1 complex; upper left, active residues crucial for CASK binding with AKT1; lower left, active residues crucial for AKT1 binding with CASK; right, an overarching schematic of PPFIA4/CASK/AKT1 complex. (**C**) In vitro binding between HA-AKT1 and GST-CASK or between HA-AKT1 and GST-PPFIA4 was analyzed by GST pull-down assays. (**D**, **G**, and **H**) AGS cells expressing PPFIA4-Flag were lysed and IP with anti-Flag or anti-CASK Abs. The IP samples were analyzed by Western blotting. (**E**) Immunofluorescence showing the PPFIA4/CASK/AKT1 colocalization in AGS cells expressing PPFIA4-Flag. Scale bar: 10 μm. (**F**) AGS cells expressing PPFIA4-Flag or NC-Flag were cultured. sg*PPFIA4*- or sgNC-modified AGS cells and si*CASK* or siNC pretreated AGS cells were stimulated with *H*. *pylori* (MOI = 100) for 24 hours. Proximity ligation assay was performed using anti-PPFIA4, anti-CASK, anti-AKT1, anti-AKT1(Thr308), and anti-AKT1(Ser473) Abs. Single Ab only was used as negative control. Red dots represent close relationship between the 2 proteins. Scale bar: 10 μm.

To explore the function of the PPFIA4/CASK complex in GECs, we conducted tandem mass tag combined with liquid chromatography–tandem mass spectrometry (TMT-LC-MS/MS) and quantitative phosphoproteome analysis of AGS cells expressing PPFIA4-Flag. Compared with the control AGS cells, AKT1 was the top significant upregulated kinase in AGS cells expressing PPFIA4-Flag (Figure 12A and Supplemental Figure 16A). Enrichment analyses of GO terms and the Kyoto Encyclopedia of Genes and Genomes (KEGG) along with GSEA revealed that protein phosphorylation in AGS cells expressing PPFIA4-Flag was mainly involved in AKT1 signaling activation and PPFIA4’s SAM domain binding (Supplemental Figure 16, B–D). Next, in silico prediction showed several residues between AKT1 and CASK likely involved in the interaction of AKT1 with CASK as well as in the formation of the PPFIA4/CASK/AKT1 complex (Figure 12B and Supplemental Figure 17A). Furthermore, a direct interaction between exogenous GST-CASK and HA-AKT1, but not between exogenous GST-PPFIA4 and HA-AKT1, was observed in the GST pull-down assays in vitro (Figure 12C), and an endogenous PPFIA4/CASK/AKT1 complex was observed in co-IP experiments (Figure 12D), suggesting that PPFIA4/CASK can interact with AKT1 via CASK. These findings were supported by immunofluorescence microscopy showing that PPFIA4, CASK, and AKT1 were colocalized (Figure 12E and Supplemental Figure 18A) and by the proximity ligation assays showing the physical proximity of PPFIA4, CASK, and AKT1 (Figure 12F and Supplemental Figure 18B). Collectively, our data demonstrate that PPFIA4/CASK can interact with AKT1 in GECs.

**Figure 13:**
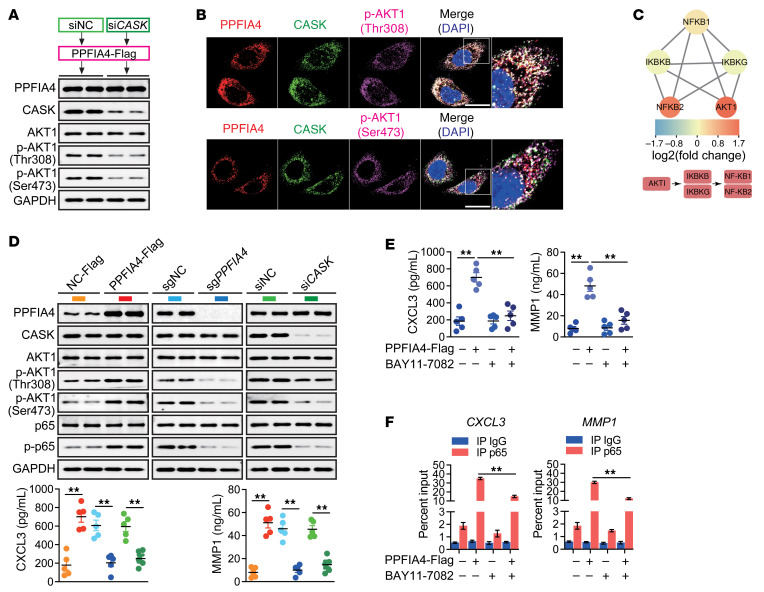
PPFIA4/CASK promotes NF-κB phosphorylation via interaction with and activation of AKT1 during *H*. *pylori* infection. (**A**) sg*PPFIA4*-modified AGS cells were transfected with si*CASK* or siNC and then with *PPFIA4*-Flag-pLVX. PPFIA4, CASK, AKT1, p-AKT1(Thr308), and p-AKT1(Ser473) proteins were analyzed by Western blotting. (**B**) Immunofluorescence showed the PPFIA4/CASK/p-AKT1(Thr308) or PPFIA4/CASK/p-AKT1(Ser473) colocalization in AGS cells expressing PPFIA4-Flag. Scale bars: 10 μm. (**C**) The network and the pathway of significantly upregulated AKT1/NF-κB signaling cascades are shown in AGS cells expressing PPFIA4-Flag compared with AGS cells expressing NC-Flag. (**D**) AGS cells expressing PPFIA4-Flag or NC-Flag were cultured. sg*PPFIA4*- or sgNC-modified AGS cells and si*CASK* or siNC pretreated AGS cells were stimulated with *H*. *pylori* (MOI = 100) for 24 hours. PPFIA4, CASK, AKT1, p-AKT1(Thr308), p-AKT1(Ser473), p65, and p-p65 proteins were analyzed by Western blotting. CXCL3 and MMP1 production was analyzed by ELISA (*n* = 5). (**E** and **F**) AGS cells expressing PPFIA4-Flag were pretreated with or without BAY 11-7082 and cultured. CXCL3 and MMP1 production was analyzed by ELISA (*n* = 5). ChIP assay was performed by PCR with primers designed for NF-κB binding sites of *CXCL3* and *MMP1* promoter regions (*n* = 3). Data are presented as mean ± SEM. Statistics: unpaired 2-tailed *t* test (**D**–**F**). **P < 0.01 for groups connected by horizontal lines.

Enrichment analysis of GO terms revealing protein kinase phosphorylation in AGS cells expressing PPFIA4-Flag implied that PPFIA4/CASK might induce AKT1 phosphorylation following interaction with AKT1 in GECs (Supplemental Figure 17, B and C). Importantly, we validated the phosphorylated AKT1 at Thr308 and Ser473 sites in an endogenous PPFIA4/CASK/AKT1 complex by co-IP experiments (Figure 12, G and H). These findings were supported by the proximity ligation assays showing the physical proximity of PPFIA4, CASK, and phosphorylated AKT1 (Figure 12F and Supplemental Figure 19A). To demonstrate that CASK connects PPFIA4 and AKT1 phosphorylation, we knocked down CASK in sg*PPFIA4*-modified AGS cells and overexpressed PPFIA4 in these cells, observing significantly decreased phosphorylation of AKT1 (Figure 13A). Immunofluorescence analysis showed that PPFIA4, CASK, and phosphorylated AKT1 were colocalized (Figure 13B and Supplemental Figure 19B). Collectively, our data demonstrate that PPFIA4/CASK can interact with AKT1 and activate AKT1 in GECs.

To explore the function of the PPFIA4/CASK/AKT1 complex in GECs, we reanalyzed data from TMT-LC-MS/MS to find that PPFIA4 activated the NF-κB pathway in GECs by mapping the pathway and network of signaling cascades (Figure 13C and Supplemental Figure 20A). Similar to the patterns in *Ppfia4*^ΔGEC^ mice, whole-transcriptome sequencing showed that sg*PPFIA4*-modified AGS cells exerted decreased expression of *CXCL3* and *MMP1* (Supplemental Figure 20B). More importantly, KEGG and GO analyses revealed that the NF-κB pathway was involved in PPFIA4-associated signaling (Supplemental Figure 20, C and D). Furthermore, CXCL3, MMP1, and the phosphorylation of p65, a direct NF-κB pathway downstream substrate, from/in AGS cells were regulated in a PPFIA4/CASK-dependent manner (Figure 13D and Supplemental Figure 20E). Finally, pretreatment with the NF-κB pathway inhibitor BAY 11-7082 effectively decreased CXCL3 and MMP1 (Figure 13E and Supplemental Figure 20F), and ChIP assays showed that PPFIA4 overexpression significantly increased NF-κB binding to the promoters of *CXCL3* and *MMP1*, which was abolished when pretreated with BAY 11-7082 (Figure 13F, Supplemental Figure 20G, and Supplemental Tables 9 and 10). Collectively, our data demonstrate that PPFIA4/CASK promotes NF-κB phosphorylation via interaction with and activation of AKT1 in GECs during *H*. *pylori* infection.

### Kira6 is a molecular inhibitor of PPFIA4 that ameliorates H. pylori persistence and H. pylori–induced gastritis.

**Figure 14:**
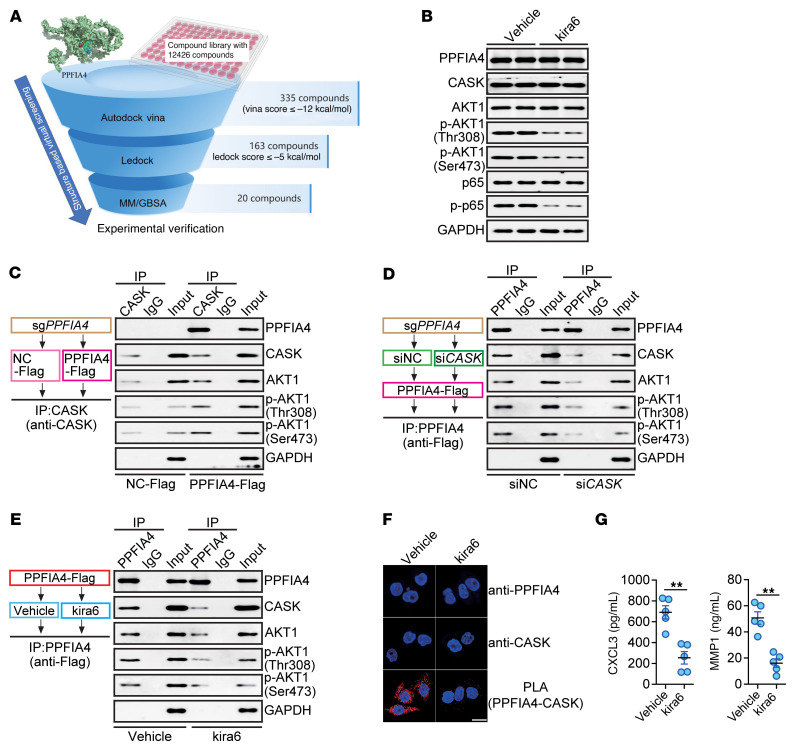
Kira6 is a molecular inhibitor of PPFIA4. (**A**) Schematic showing the protocol for compound selection. (**B**) AGS cells expressing PPFIA4-Flag were treated with kira6 (100 nM) for 2 hours. PPFIA4, CASK, AKT1, p-AKT1(Thr308), p-AKT1(Ser473), p65, and p-p65 proteins were analyzed by Western blotting. (**C**) sg*PPFIA4*-modified AGS cells were transfected with *PPFIA4*-Flag-pLVX or NC-Flag-pLVX for 24 hours and then lysed and immunoprecipitated with anti-CASK Abs. The IP samples were analyzed by Western blotting. (**D**) sg*PPFIA4*-modified AGS cells were transfected with si*CASK* or siNC (40 nM) for 24 hours and transfected with *PPFIA4*-Flag-pLVX for another 24 hours before being lysed and immunoprecipitated with anti-Flag Abs. The IP samples were analyzed by Western blotting. (**E**) AGS cells expressing PPFIA4-Flag were treated with kira6 (100 nM) for 2 hours and then lysed and immunoprecipitated with anti-Flag Abs. The IP samples were analyzed by Western blotting. (**F** and **G**) AGS cells expressing PPFIA4-Flag were treated with kira6 (100 nM) for 2 hours. Proximity ligation assay was performed using anti-PPFIA4 and anti-CASK Abs. Single Ab only was used as negative control. Red dots represent close relationship between the 2 proteins. Scale bar: 10 μm. CXCL3 and MMP1 production was analyzed by ELISA (*n* = 5). Data are presented as mean ± SEM. Statistics: unpaired 2-tailed *t* test (**G**). **P < 0.01 for groups connected by horizontal lines.

Given that PPFIA4 substantially promotes gastric *H*. *pylori* colonization and gastritis, we next explored the potential molecular inhibitors of PPFIA4. After screening, 20 compounds were selected for further study based on the lowest docking scores (Figure 14A and Supplemental Table 11). Next, we treated AGS cells expressing PPFIA4-Flag with these compounds individually and found that only kira6 significantly inhibited the expression of *CXCL3* and *MMP1* without affecting the cell viability and proliferation (Supplemental Figure 21, A and B). However, kira6 had no inhibitory effects on the expression of *CXCL3* and *MMP1* in sg*PPFIA4*-modified AGS cells (Supplemental Figure 21C). Furthermore, phosphorylated AKT1 and p65 were decreased in AGS cells expressing PPFIA4-Flag treated with kira6 (Figure 14B). Molecular dynamics simulations illustrated the stability of the specific interaction between PPFIA4 and kira6 (Supplemental Figure 22). Finally, compared with CASK, AKT1, and p65, PPFIA4 achieved the lowest docking score and binding free energy with kira6 (Supplemental Tables 12 and 13). Collectively, our data demonstrate that kira6 is a specific molecular inhibitor of PPFIA4 in GECs.

To explore the effect of kira6 on the PPFIA4/CASK/AKT1 complex in GECs, we conducted in vitro co-IP experiments and evaluated the phosphorylation of AKT1. First, compared with the cells transfected with NC-Flag-pLVX, sg*PPFIA4*-modified AGS cells transfected with *PPFIA4*-Flag-pLVX followed by IP with anti-CASK showed both increased AKT1 and p-AKT1, suggesting that overexpression of PPFIA4 enhances the binding of CASK to AKT1 and the AKT1 phosphorylation in the PPFIA4/CASK/AKT1 complex (Figure 14C). Next, compared with the cells transfected with NC siRNA, sg*PPFIA4*-modified AGS cells transfected with *CASK* siRNA followed by *PPFIA4*-Flag-pLVX transfection and subsequent IP with anti-Flag showed both decreased AKT1 and p-AKT1, suggesting that PPFIA4/CASK binds to AKT1 to form a PPFIA4/CASK/AKT1 complex and activate AKT1 via CASK (Figure 14D). Finally, kira6 treatment effectively decreased CASK, AKT1, and p-AKT1 in AGS cells expressing PPFIA4-Flag followed by IP with anti-Flag, suggesting that kira6 inhibits PPFIA4/CASK/AKT1 complex formation and AKT1 phosphorylation in the PPFIA4/CASK/AKT1 complex (Figure 14E), which was supported by decreased fluorescence in the proximity ligation assays of PPFIA4 and CASK (Figure 14F and Supplemental Figure 23A) and decreased CXCL3 and MMP1 by ELISA (Figure 14G) in AGS cells expressing PPFIA4-Flag treated with kira6. Collectively, our data demonstrate that kira6 is an effective molecular inhibitor of PPFIA4 in GECs.

**Figure 15:**
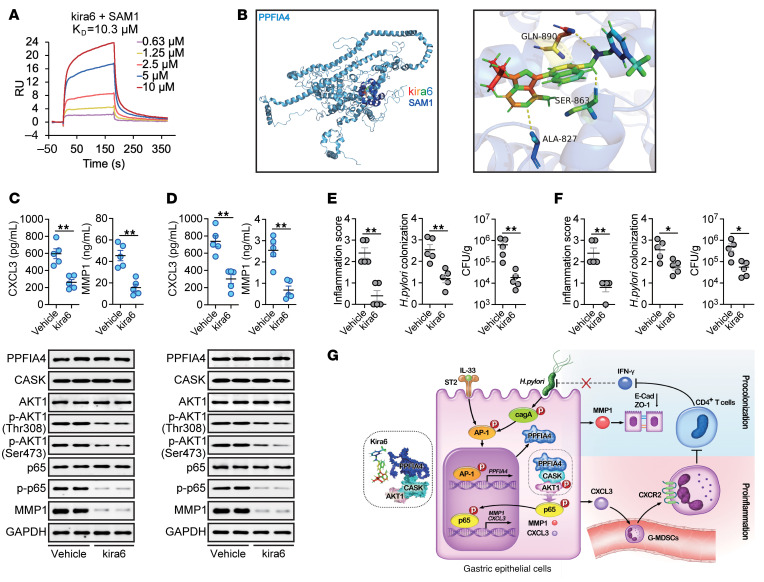
Kira6 is a molecular inhibitor of PPFIA4 that ameliorates *H*. *pylori* persistence and *H*. *pylori*–induced gastritis. (**A**) Surface plasmon resonance experiments measuring the binding ability of the SAM1 domain of PPFIA4 with kira6. Colored curves represent kira6 concentration with serial 1:2 dilutions. RU, response unit. (**B**) Autodock predicted molecular docking of PPFIA4 with kira6. (**C** and **D**) sgNC-modified AGS cells (**C**) and primary GECs from uninfected WT mice (**D**) were treated with kira6 (100 nM) for 2 hours, then stimulated with *H*. *pylori* (MOI = 100) for 24 hours. PPFIA4, CASK, AKT1, p-AKT1(Thr308), p-AKT1(Ser473), p65, p-p65, and MMP1 proteins were analyzed by Western blotting. CXCL3 and MMP1 production was analyzed by ELISA (*n* = 5). (**E** and **F**) The bacteria colonization and the histological scores of inflammation in gastric mucosa of *H*. *pylori*–infected *Ppfia4*^fl/fl^ littermates injected with kira6 15 (**E**) or 18 (**F**) weeks p.i. were compared (*n* = 5). The bacteria colonization is shown as log_10_(the number of bacterial genomes per nanogram of host genomic DNA) by measuring *H*. *pylori*–specific 16s rDNA or as CFU per gram of stomach tissue by bacterial reisolation and quantitative culture. (**G**) A proposed model of crosstalk among *H*. *pylori*, IL-33, GECs, PPFIA4, CASK, AKT1, MMP1, CXCL3, G-MDSCs, and CD4^+^ T cells leading to PPFIA4-mediated procolonization and proinflammation in gastric mucosa during *H*. *pylori* infection. Data are presented as mean ± SEM. Statistics: Mann-Whitney *U* test (**E** and **F**), and unpaired 2-tailed *t* test (**C**–**F**). *P < 0.05, **P < 0.01 for groups connected by horizontal lines.

To elucidate the binding sites of kira6 on PPFIA4, we performed a surface plasmon resonance assay. The results showed that SAM1 and SAM1-3 domains exhibited similar binding affinities to kira6, which was much higher than that of SAM2-3 domain binding to kira6, suggesting that kira6 specifically binds to the SAM1 domain of PPFIA4 (Figure 15, A and B, and Supplemental Figure 23, B–D).

To explore the effect of kira6 on the function of GEC-derived PPFIA4 during *H*. *pylori* infection, AGS cells and mouse primary GECs were in vitro treated with kira6 and infected with *H*. *pylori*. CXCL3 and MMP1 as well as phosphorylated AKT1 and p65 from/in AGS cells (Figure 15C) and mouse primary GECs (Figure 15D) were significantly decreased by kira6 treatment, which was abolished in sg*PPFIA4*-modified AGS cells (Supplemental Figure 24A) or in mouse primary GECs from *Ppfia4*^–/–^ mice (Supplemental Figure 24B). Furthermore, in the in vivo prevention models (administered soon after *H*. *pylori* infection), the levels of G-MDSCs, CXCL3, MMP1, bacteria colonization, and inflammation were significantly decreased in gastric mucosa of *Ppfia4*^fl/fl^ littermates injected with kira6 15 weeks p.i. (Figure 15E and Supplemental Figure 25, A–C), which was abolished in *Ppfia4*^ΔGEC^ mice (Supplemental Figure 25D). In the in vivo therapy models (administered when PPFIA4 increased during *H*. *pylori* infection), the levels of G-MDSCs, CXCL3, MMP1, bacteria colonization, and inflammation were significantly decreased in gastric mucosa of *Ppfia4*^fl/fl^ littermates injected with kira6 18 weeks p.i. (Figure 15F and Supplemental Figure 25, E–G), which was abolished in *Ppfia4*^ΔGEC^ mice (Supplemental Figure 25H). As kira6 was originally found to target inositol-requiring enzyme 1α (IRE1α) as an endoplasmic reticulum stress inhibitor (21), we investigated its potential off-target impact and found that it did not affect IRE1α phosphorylation in GECs during *H*. *pylori* infection in vitro (Supplemental Figure 24, C and D) and that no significant histological changes were detected in other tissues where IRE1α is reported to play critical roles in these in vivo prevention and therapy models (Supplemental Figure 26). Similar observations were made when infected with antibiotic-resistant *H*. *pylori* in these in vivo prevention and therapy models (Supplemental Figure 27). Collectively, our data demonstrate that kira6 is a molecular inhibitor of PPFIA4 capable of ameliorating bacterial persistence and gastritis during *H*. *pylori* infection.

## Discussion

Recent evidence has revealed the pathological roles of PPFIA4 in the progression of cancers (22). However, the functional roles of PPFIA4 in infectious diseases remain largely unknown. In the present study from both in vitro and in vivo gain- and loss-of-function experiments, we provide an example of an immune network regulated by digestive system–derived PPFIA4 induced by bacterial pathogens. This immune network does not contribute to *H*. *pylori* clearance; rather, it efficiently promotes gastric *H*. *pylori* persistence as well as *H*. *pylori* infection–driven gastritis. Thus, during *H*. *pylori* infection, PPFIA4 is induced in GECs in a CagA-dependent and IL-33–enhanced fashion and leads to activating PPFIA4/CASK/AKT1/NF-κB signaling cascades and inducing CXCL3/MMP1 production, which progressively modulates the immune network within the infected gastric mucosa, facilitating gastric *H*. *pylori* persistence and gastritis (Figure 15G). We also investigated the kinetics of gastric *Il33* expression, *Mmp1* expression, *Cxcl3* expression, G-MDSC levels, inflammation, and *H*. *pylori* colonization in *H*. *pylori*–infected mouse models (Supplemental Figure 28) that have been used in our previous studies on *H*. *pylori*–associated gastritis (6, 23) in vivo, which may emphasize the potential importance of *H*. *pylori*–induced PPFIA4 during the developing stage of gastritis.

To study the mechanism of PPFIA4 induction in GECs by *H*. *pylori*, we defined the proximal promoter of *PPFIA4* and confirmed that AP1 as a transcription factor upregulates and induces PPFIA4 in *H*. *pylori*–infected GECs. It has been reported that PPFIA4 is upregulated under hypoxic conditions and is directly regulated by HIF-1α in renal cell carcinoma (13). In our case, we identified a PPFIA4-regulating immune molecule, IL-33, during *H*. *pylori* infection that exerts a synergistic effect on PPFIA4 induction by activating AP1. *H*. *pylori* infection predisposes individuals to lifelong chronic gastric inflammation, significantly increasing the risk for gastric cancer. Immune suppressor cells dampen the active inflammatory process, leading to immune evasion or response during *H*. *pylori* infection (24). Here, we identified a neutrophil subset, G-MDSCs, with inflammatory features as well as suppressing potential on T cell activation by scRNA-seq, flow cytometry, DRUG-seq, and SMART-seq, whose accumulation is regulated by GEC-derived PPFIA4 during *H*. *pylori* infection. It was previously reported that a subset of myeloid cells is recruited to the gastric epithelium during *H*. *felis* infection and polarized into MDSCs (25), which inhibits T cells to promote *H*. *felis*–induced spasmolytic polypeptide-expressing metaplasia (26). This resembles our data showing that gastric G-MDSCs inhibit *H*. *pylori*–specific IFN-γ–producing CD4^+^ T cells. Spasmolytic polypeptide-expressing metaplasia could evolve from chief cells in models of *H*. *felis* infection (27), which would be different from our model focusing on the pathological process of *H*. *pylori*–associated gastritis. Notably, using our model, we mechanistically demonstrated that *H*. *pylori*–induced PPFIA4 in GECs leads to impaired host *H*. *pylori*–specific IFN-γ–producing CD4^+^ T cell response, resulting in increased bacterial colonization. Additionally, our observations showing *H*. *pylori* colonization by GEC-derived PPFIA4 was accompanied by increased MMP1 in vivo. Together, these data support the concept that PPFIA4 acts through a 2-pronged mechanism, involving both suppressing host defense and promoting gastric mucosal damage, which progressively contributes to *H*. *pylori* persistence and gastritis.

We have not only demonstrated that GECs express PPFIA4 but also elucidated the underlying molecular mechanisms for its binding partners and function. CASK has several domains, including the CaMK domain, that participate in the host’s immune response regulation during bacterial infection via activating downstream signaling cascades (28, 29). Our results identify that PPFIA4’s SAM1 domain directly binds domains of CaMK to the first L27 of CASK, which subsequently binds to AKT1 via CASK, leading to AKT1 phosphorylation most likely via the kinase activity of CaMK. We postulate that the combination of phosphorylated Thr308 and Ser473 sites in the activated AKT1 further phosphorylates p65 of NF-κB to induce CXCL3/MMP1 secretion, which regulates the local immune network during *H*. *pylori* infection.

The antiinflammation role of molecular compounds in *H*. *pylori* persistence has garnered increasing attention recently. Here, we demonstrated that kira6 is a molecular inhibitor of PPFIA4 to ameliorate bacterial persistence and gastritis during *H*. *pylori* infection. Kira6 was originally found to target IRE1α as an endoplasmic reticulum stress inhibitor (21), which has been reported to have antiinflammatory effects (30). Our study confirmed that kira6 has antiinflammatory activity that minimizes *H*. *pylori*–associated gastritis by inhibiting the PPFIA4/CXCL3 pathway in vitro and the CXCL3/G-MDSC axis in vivo. In this regard, our data may emphasize a key role of PPFIA4 in mediating pathological factors in the development of *H*. *pylori*–associated gastritis, and the in vivo data from both prevention and therapy models also emphasize kira6’s antiinflammatory activity in *H*. *pylori* infection–induced gastritis.

In summary, our findings demonstrate that PPFIA4 plays a critical role in *H*. *pylori* persistence and *H*. *pylori*–induced gastritis, which could have profound clinical implications (Supplemental Figure 29). The PPFIA4/CASK/AKT1/NF-κB signaling cascade promotes GEC’s proinflammatory properties and enables bacterial persistence. Although eradication therapy for *H*. *pylori* by oral antibiotics has progressed in recent years (31), it is noteworthy that *H*. *pylori* colonization commonly persists because of increased antimicrobial resistance and impaired host defense. Subsequent to the increasing antibiotic resistance, a substantial drop in *H*. *pylori* treatment efficacy has been noted (32). In this regard, our findings suggest a possible therapeutic target, PPFIA4, as well as its molecular inhibitor, kira6, in *H*. *pylori* infection. Targeting GEC-derived PPFIA4 could provide an opportunity to treat persistent *H*. *pylori* infection–induced clinical gastritis, especially in patients with resistance to antibiotics.

## Methods

### Sex as a biological variable.

Gene/protein expression analyses were performed using gastric biopsy specimens from 50 uninfected donors, 131 *H*. *pylori*–infected patients, and 32 gastric ulcer patients. No sex-related differences were observed. Sex was not considered as a biological variable in the mouse experiments. Infection experiments were conducted in female mice. Female mice were used to experimentally model infection to ensure consistency with previous studies (6, 23). Sex-based differences in immune responses to *H*. *pylori* infection have been reported, with males typically showing more impaired endothelial function (33) and more severe pathology (34, 35). Experiments not involving infection and involving in vitro studies were conducted in male mice to utilize available counterparts, in line with ethical principles for animal use.

For further details, see Supplemental Methods.

### Statistics.

Data are representative of 2 independent experiments. Data are expressed as the mean ± SEM. Comparisons between 2 groups were performed using Student’s *t* test or Mann-Whitney *U* test. Multiple comparisons were performed using 1- or 2-way ANOVA. Correlations between parameters were assessed using Pearson’s correlation analysis and linear regression analysis. SPSS statistical software (version 13.0) was used for all statistical analysis. All data were analyzed using 2-tailed tests, and *P* < 0.05 was considered statistically significant.

### Study approval.

All breeding and experiments were approved by the Animal Ethical and Experimental Committee of Third Military Medical University (AMUWEC20218020). The experiments involving human samples were approved by the Ethics Committee of XinQiao Hospital (2021-148-01) and Southwest Hospital (KY202220) of Third Military Medical University. Written informed consent was obtained from each subject.

### Data availability.

Values for all data points in graphs can be found in the Supporting Data Values file. The bulk RNA-seq data generated in this study have been deposited in the NCBI GEO under accession code GSE272270. The microarray data generated in this study have been deposited in the NCBI GEO under accession code GSE264263. The SLAM-seq, DRUG-seq, SMART-seq, and scRNA-seq data generated in this study have been deposited in the NCBI Sequence Read Archive under accession numbers PRJNA1228176, PRJNA1227754, PRJNA1227108, and PRJNA1229657, respectively. The publicly available human gastric tissue data used in this study are available in the NCBI GEO database under accession code GSE249874.

## Author contributions

All authors participated meaningfully in the study, and they have read and approved the submission of this manuscript. YZ designed the research. YZ, BW, PW, NY, YST, YPL, and WQT participated in performing the research, analyzing the data, and writing the original draft of the article. BW, NY, YST, YPL, WQT, and WC revised the manuscript. JYX, RX, JBW, GYY, PC, JYZ, LSP, FYM, and SLL participated in performing the research and collecting the data. SMY, YLZ, and HZ contributed reagents, mice, and human clinical samples.

## Conflict of interest

The authors have declared that no conflict of interest exists.

## Funding support

- National Natural Science Foundation of China (82470595 and 82070578).
- Chongqing Natural Science Fund for Distinguished Young Scholars (cstc2019jcyjjqX0003).
- Collaborative Innovation Center of Chinese Ministry of Education (2020-39).
- Sichuan Science and Technology Program (2024NSFSC1684).
- National Key Research and Development Program of China (2022YFA1105300).

## Supplementary Material

Supplemental data

Supporting data values

Unedited blot and gel images

## Version 1 05/01/2026

Electronic publication

## Version 2 08/17/2026

Corrections to supplemental figures and supporting data values per corrigendum
