# Corrigendum to *Helicobacter pylori*–induced PPFIA4 orchestrates immune network–promoting gastritis and gastric bacterial colonization

**DOI:** 10.1172/JCI210814

**Published:** 2026-08-17

**Authors:** Pan Wang, Nan You, Yong-Sheng Teng, Yi-Pin Lv, Wen-Qing Tian, Jing-Yu Xu, Rui Xie, Jiang-Bo Wu, Geng-Yu Yue, Ping Cheng, Jin-Yu Zhang, Liu-Sheng Peng, Fang-Yuan Mao, Shou-Lu Luo, Shi-Ming Yang, Yong-Liang Zhao, Hong Zhou, Weisan Chen, Bin Wang, Yuan Zhuang

Original citation: *J Clin Invest*. 2026;136(9):e193848. https://doi.org/10.1172/JCI193848

Citation for this corrigendum: *J Clin Invest*. 2026;136(16):e210814. https://doi.org/10.1172/JCI210814

Following the publication of this article, the corresponding author’s institution required all researchers to conduct a self-inspection of publications from the past 3 years. During this process, the authors identified errors in Supplemental Figure 8B, Supplemental Figure 25H, and Supplemental Figure 28 due to errors in referencing calculation formulas or mistakes in pasting the data. The authors have corrected the figures and supporting data values online. The authors haves stated that these errors do not alter the conclusions of the study.

The authors regret the errors.

## Version 1 08/17/2026

Electronic publication

