# Supplementary material for: Helicobacter pylori–induced PPFIA4 orchestrates immune network–promoting gastritis and gastric bacterial colonization
Source: J Clin Invest. 2026 May 1;136(9):e193848. doi: 10.1172/JCI193848 (PMC13132368; doi:10.1172/JCI193848)

Full unedited gel for Figure 1A(up)
PPFIA4 (from lane 1 to lane 6)

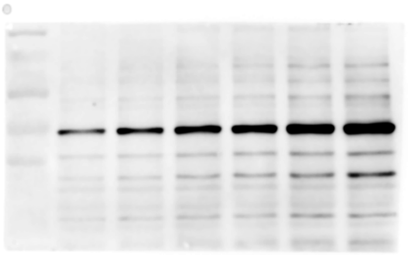

GAPDH (from lane 1 to lane 6)

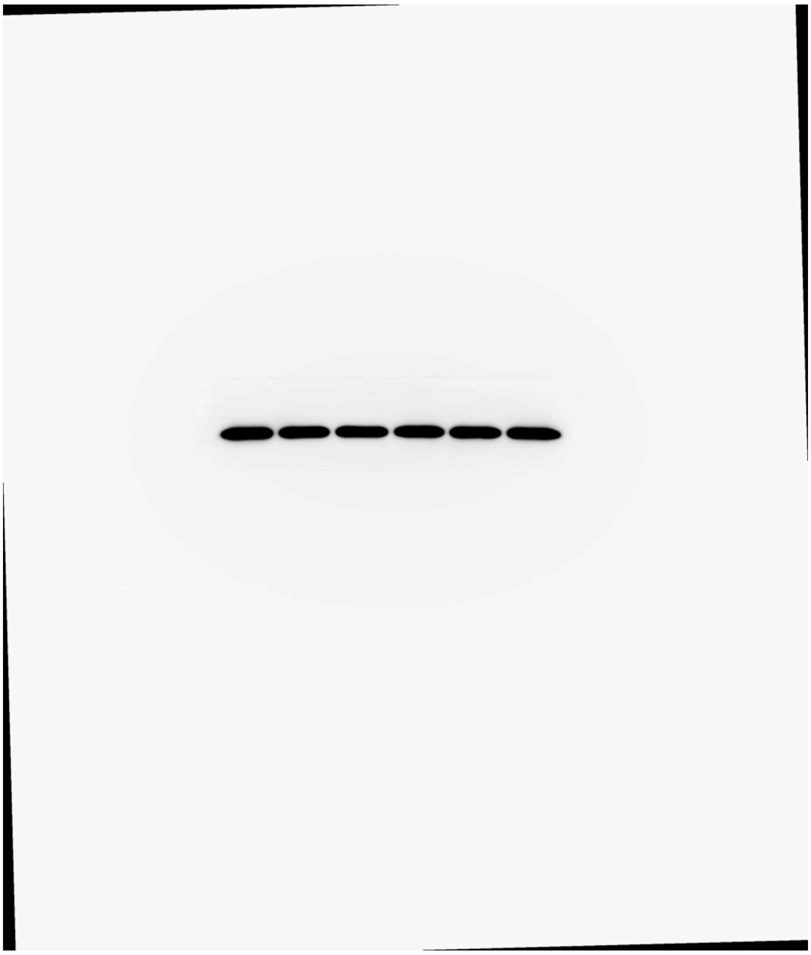

Full unedited gel for Figure 1A(down)
PPFIA4 (from lane 1 to lane 6)

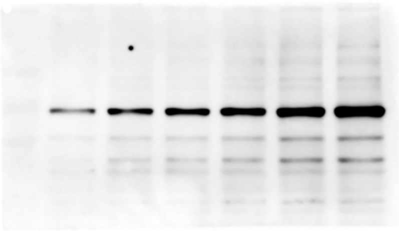

GAPDH (from lane 1 to lane 6)

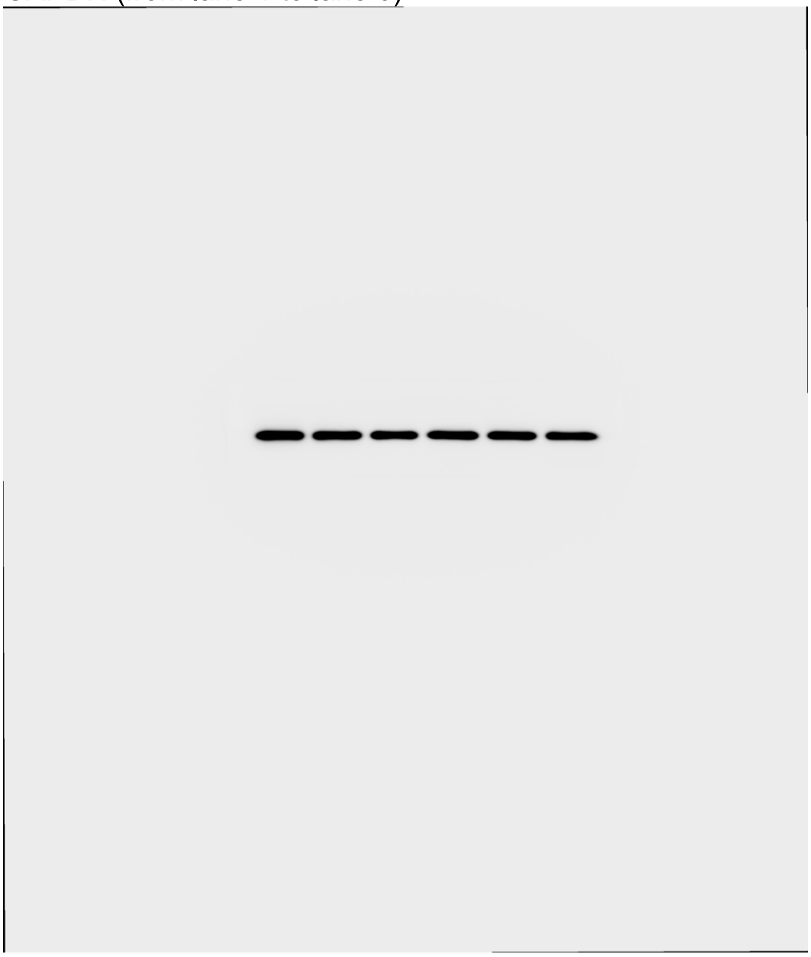

Full unedited gel for Figure 1B(up)

PPFIA4 (from lane 1 to lane 6)

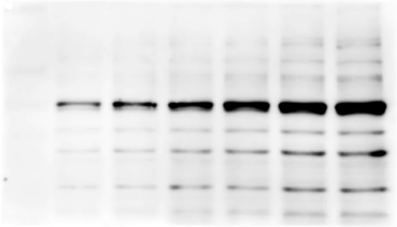

GAPDH (from lane 1 to lane 6)

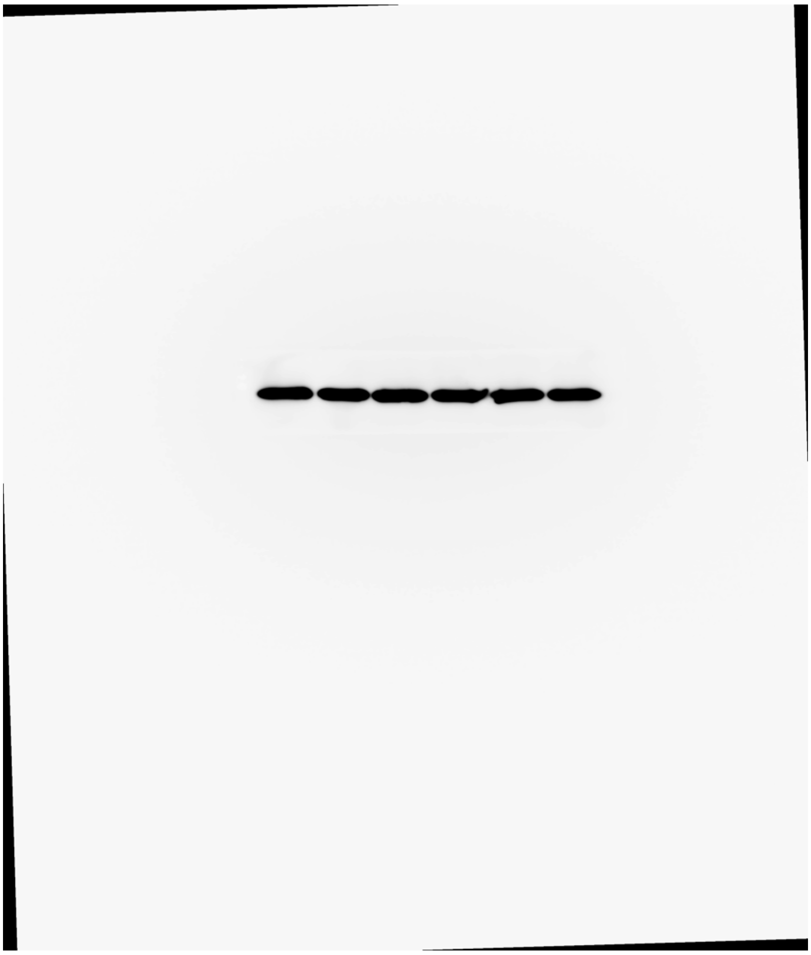

Full unedited gel for Figure 1B(down)
PPFIA4 (from lane 1 to lane 6)

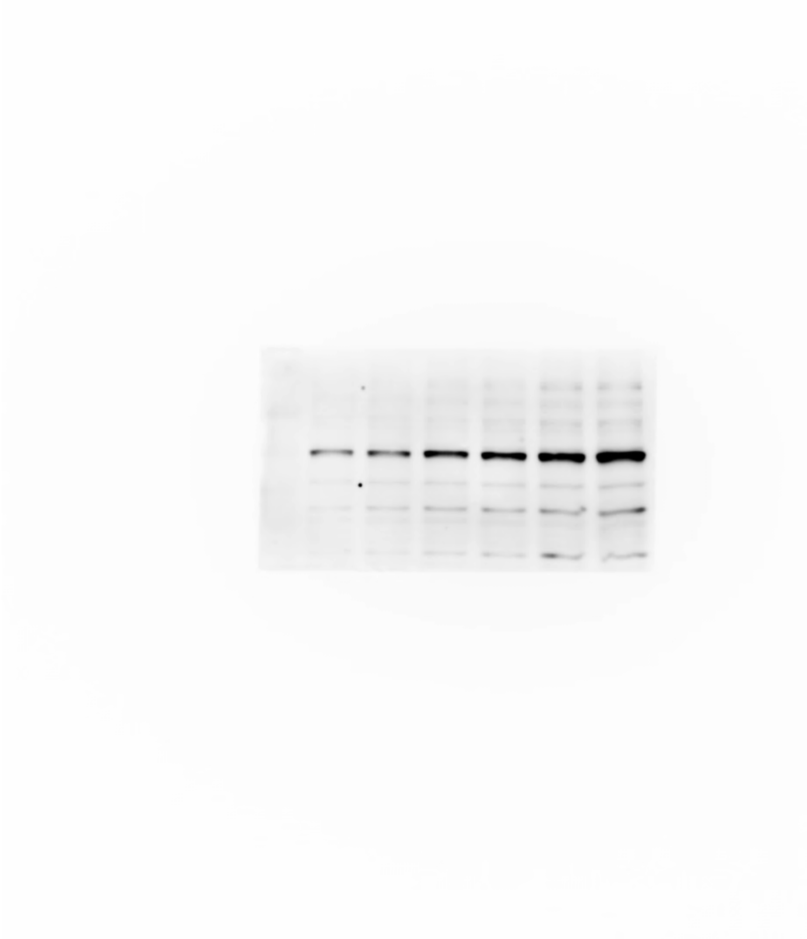

GAPDH (from lane 1 to lane 6)

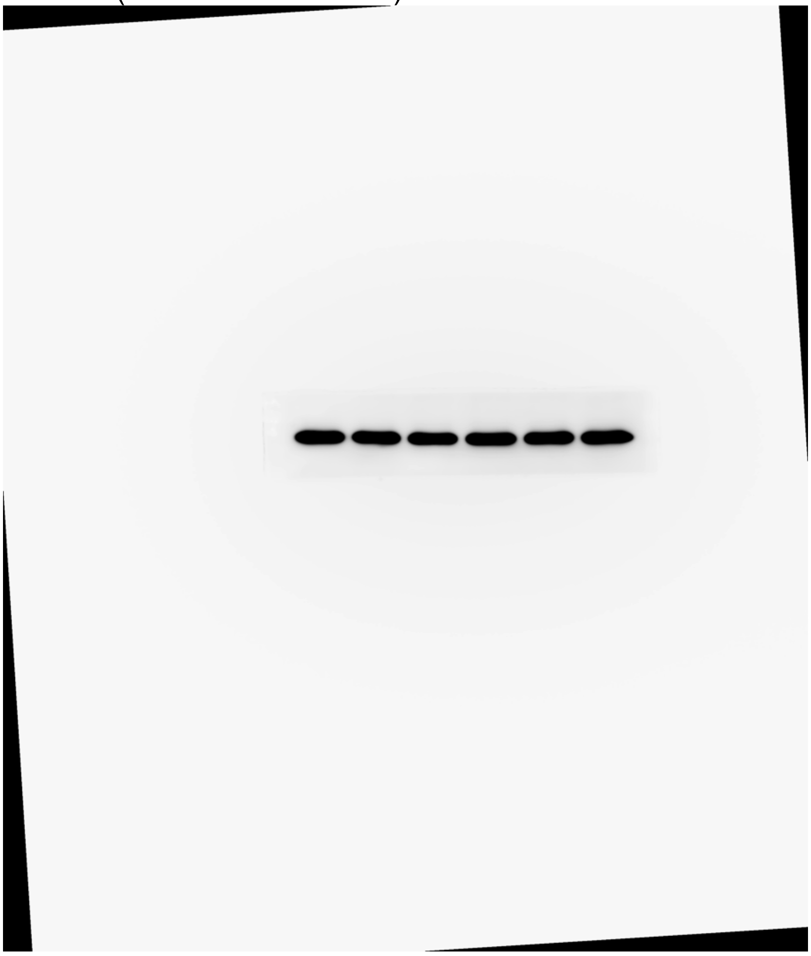

Full unedited gel for Figure 1C(up)
PPFIA4 (from lane 1 to lane 6)

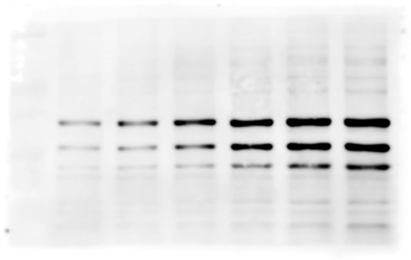

GAPDH (from lane 1 to lane 6)

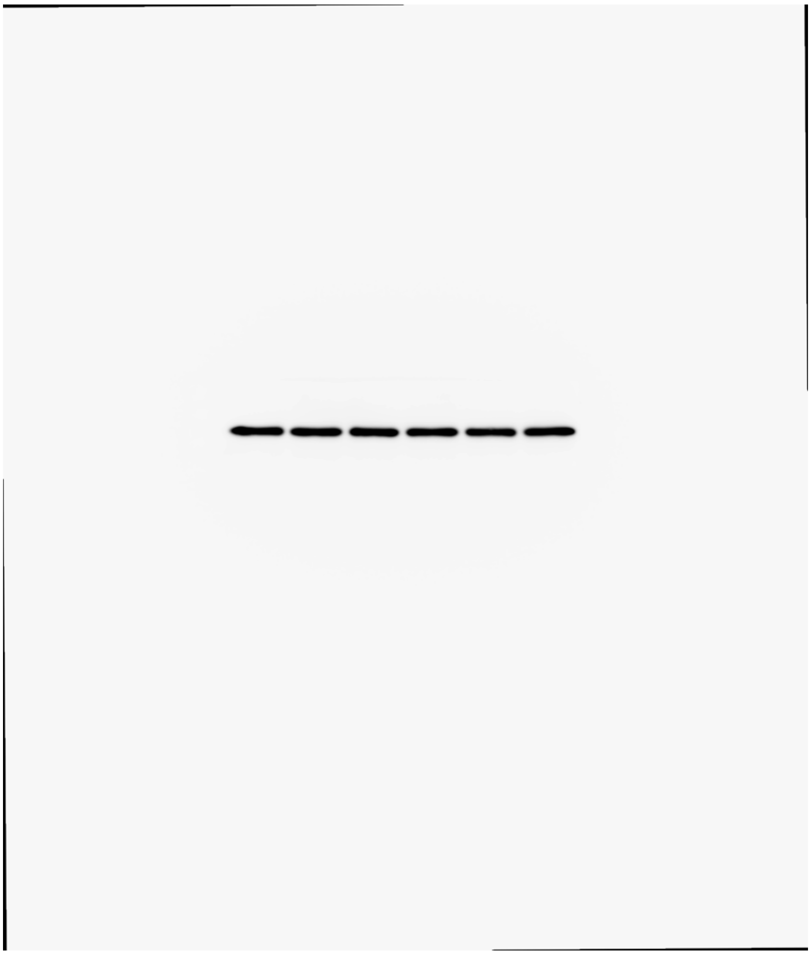

Full unedited gel for Figure 1C(down)

PPFIA4 (from lane 1 to lane 6)

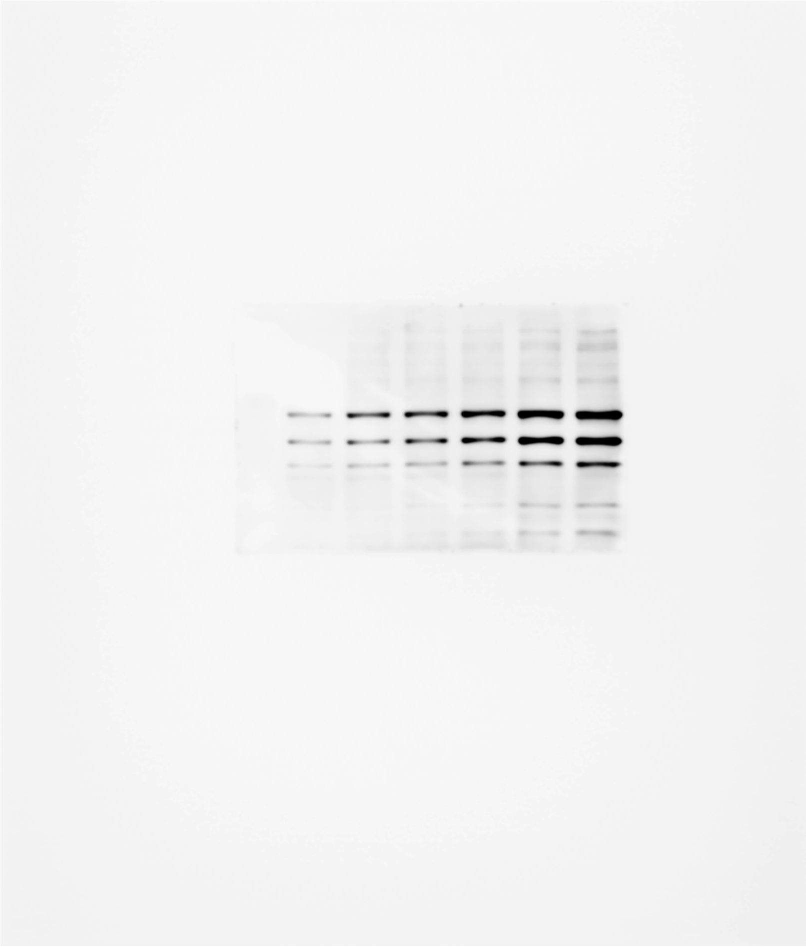

GAPDH (from lane 1 to lane 6)

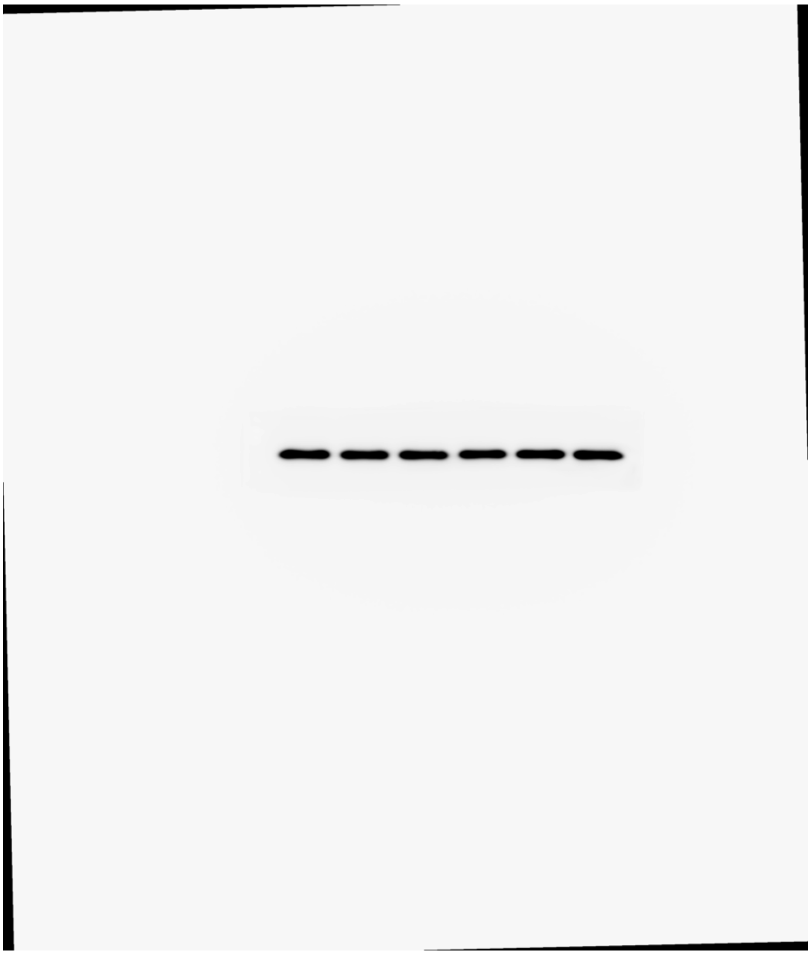

Full unedited gel for Figure 2A
PPFIA4 (from lane 1 to lane 3)

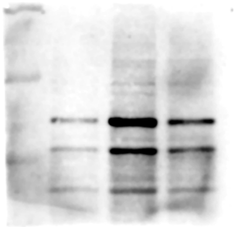

GAPDH (from lane 1 to lane 3)

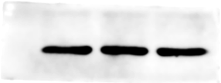

Full unedited gel for Figure 2B
PPFIA4 (from lane 1 to lane 3)

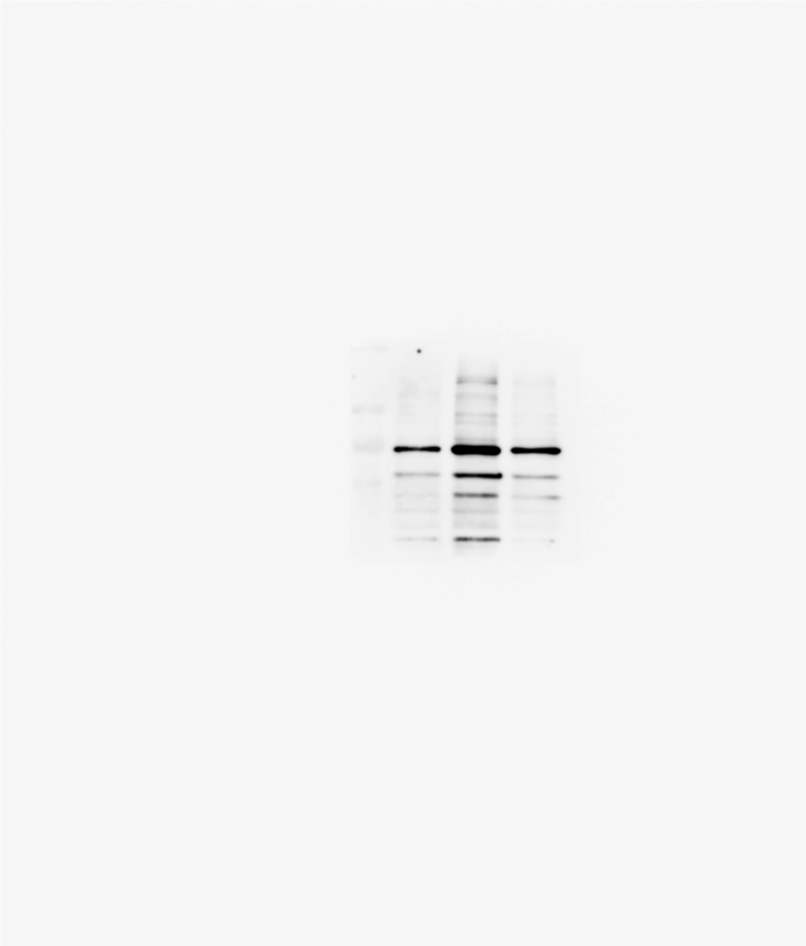

GAPDH (from lane 1 to lane 3)

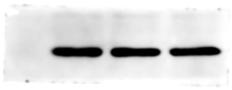

Full unedited gel for Figure 2C
PPFIA4 (from lane 1 to lane 3)

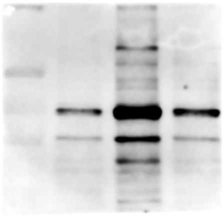

GAPDH (from lane 1 to lane 3)

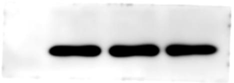

Full unedited gel for Figure 2G

PPFIA4 (from lane 1 to lane 3)

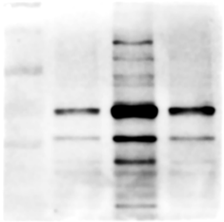

GAPDH (from lane 1 to lane 3)

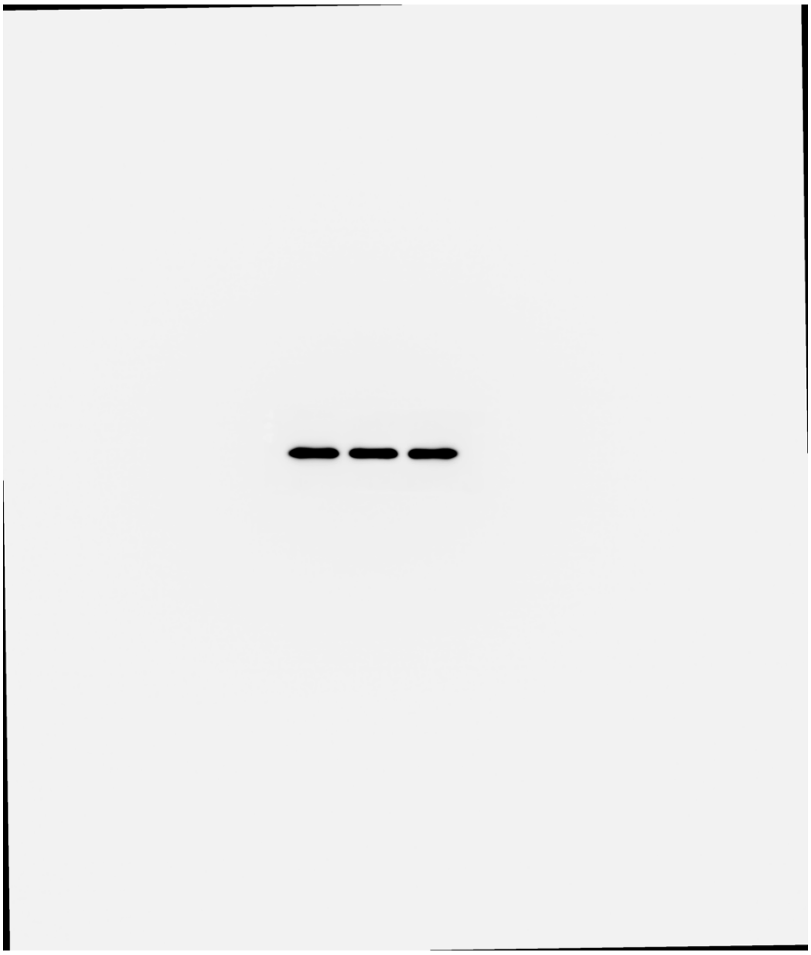

Full unedited gel for Figure 3B

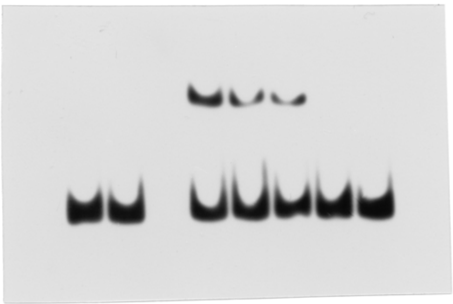

Full unedited gel for Figure 3C

PPFIA4 (from lane 1 to lane 5)

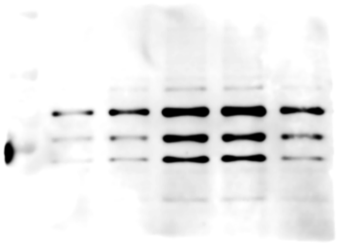

p-c-Jun (from lane 1 to lane 5)

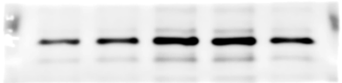

c-Jun (from lane 1 to lane 5)

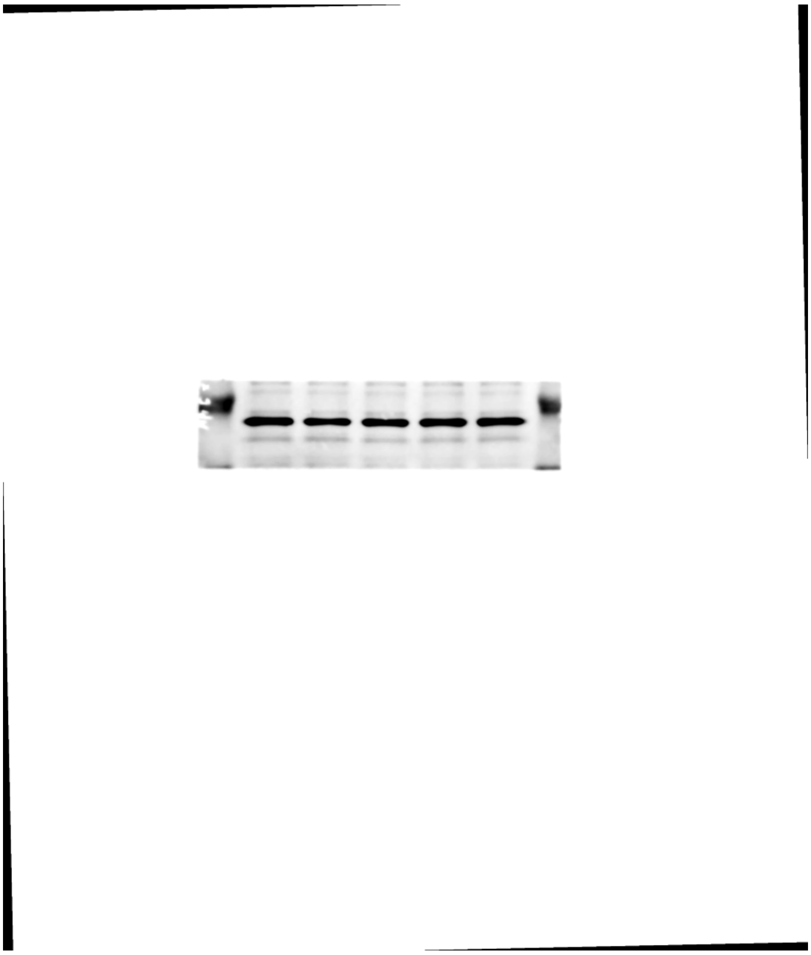

GAPDH (from lane 1 to lane 5)

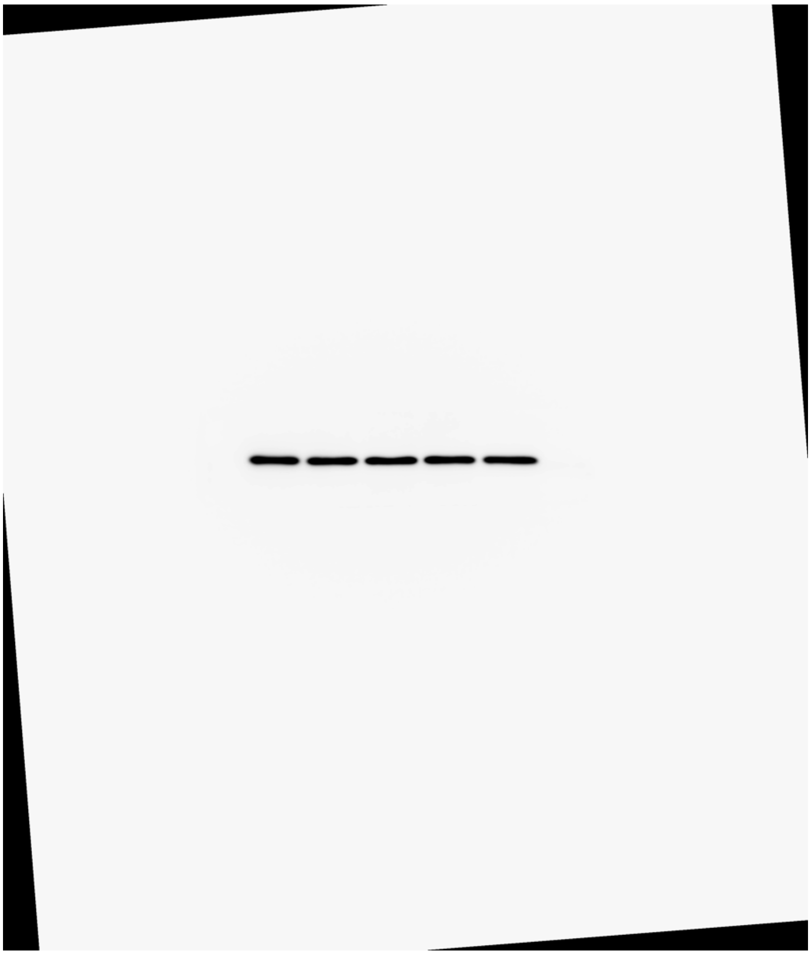

Full unedited gel for Figure 3D

PPFIA4 (from lane 1 to lane 5)

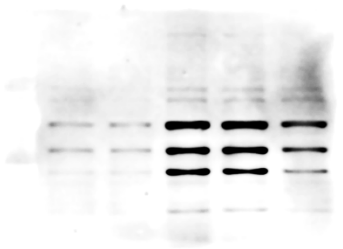

p-c-Jun (from lane 1 to lane 5)

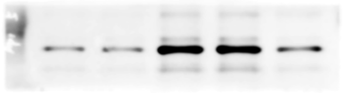

c-Jun (from lane 1 to lane 5)

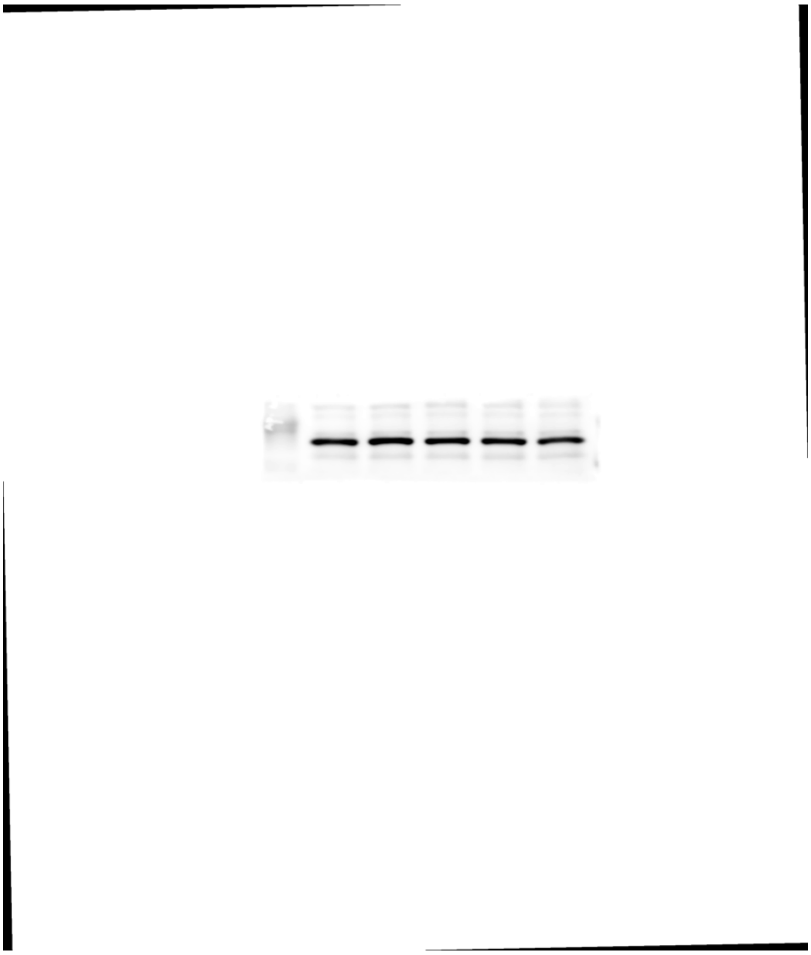

GAPDH (from lane 1 to lane 5)

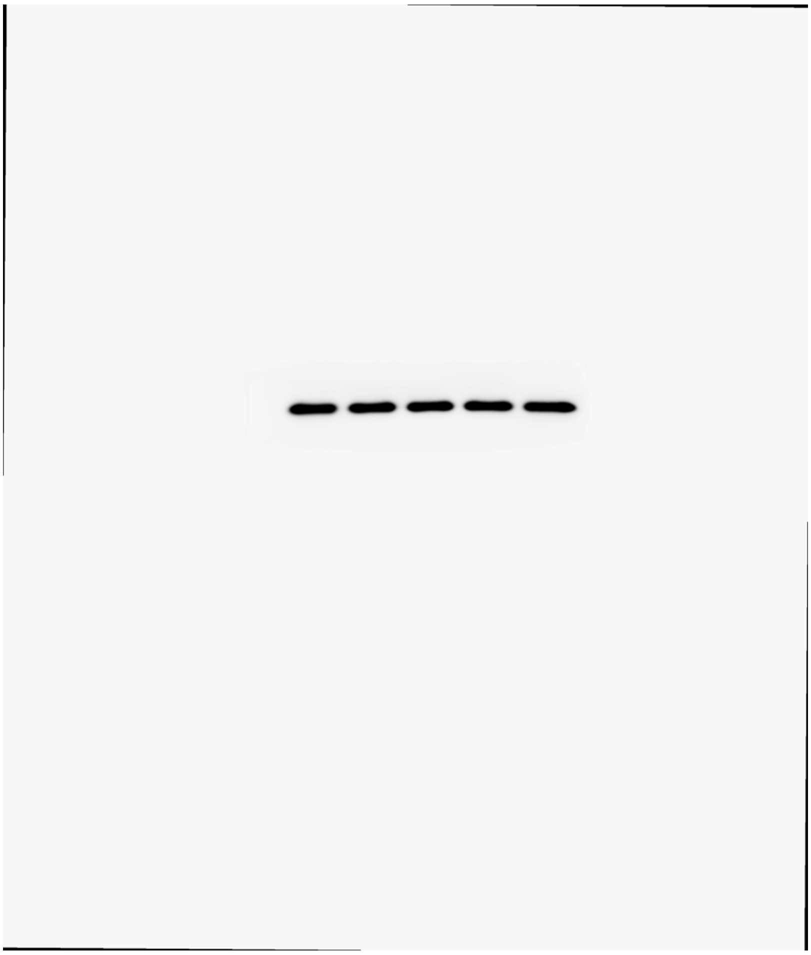

Full unedited gel for Figure 4A(left)
PPFIA4 (from lane 1 to lane 4)

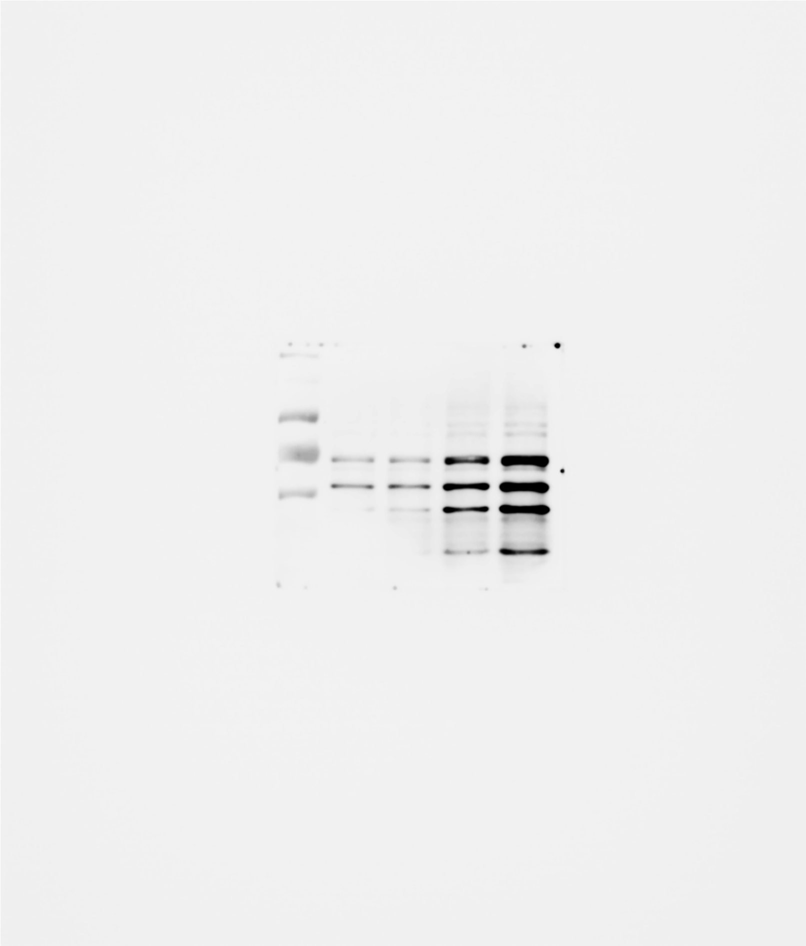

GAPDH (from lane 1 to lane 4)

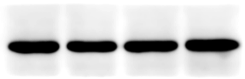

Full unedited gel for Figure 4A(right)

PPFIA4 (from lane 1 to lane 4)

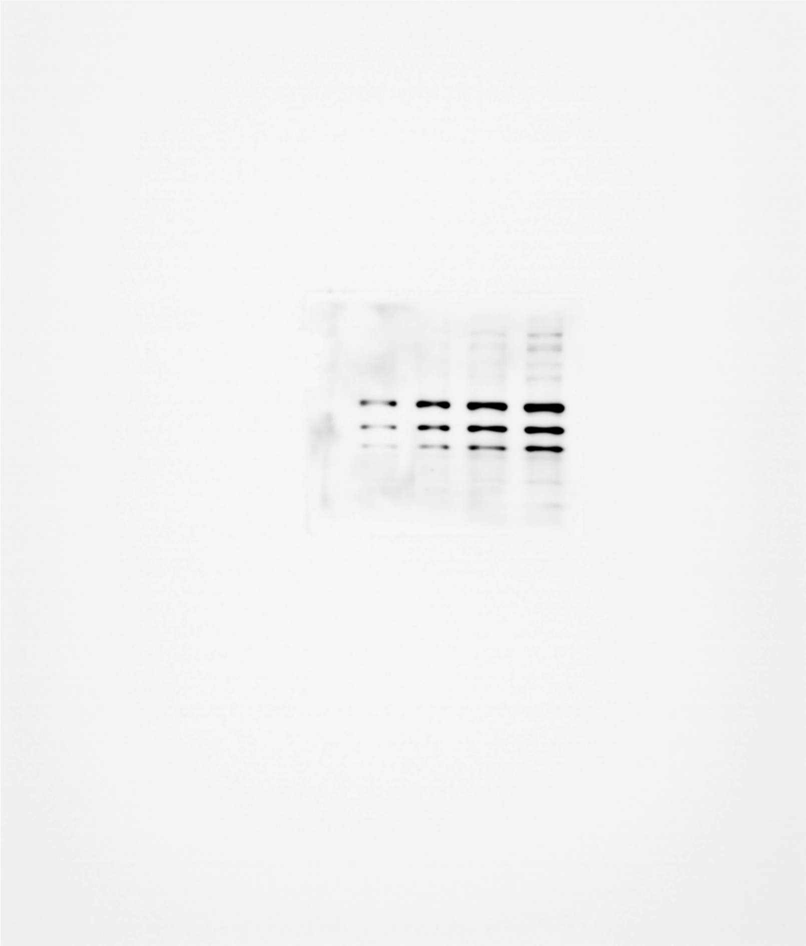

GAPDH (from lane 1 to lane 4)

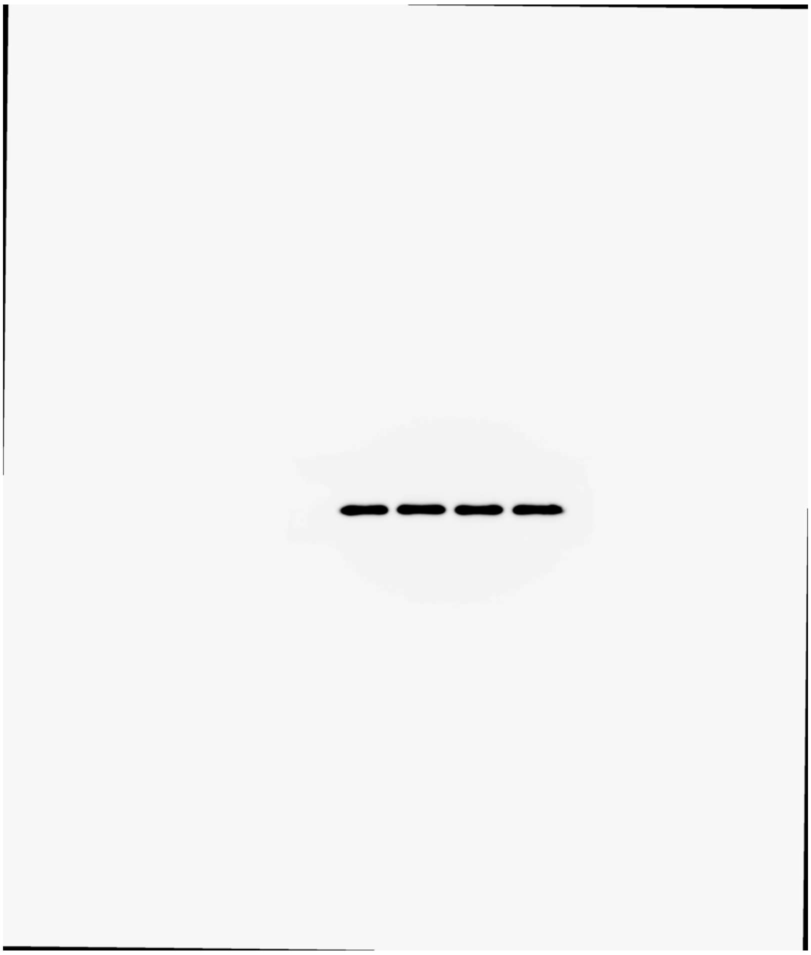

Full unedited gel for Figure 4B

PPFIA4 (from lane 1 to lane 4)

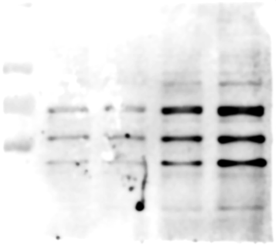

GAPDH (from lane 1 to lane 4)

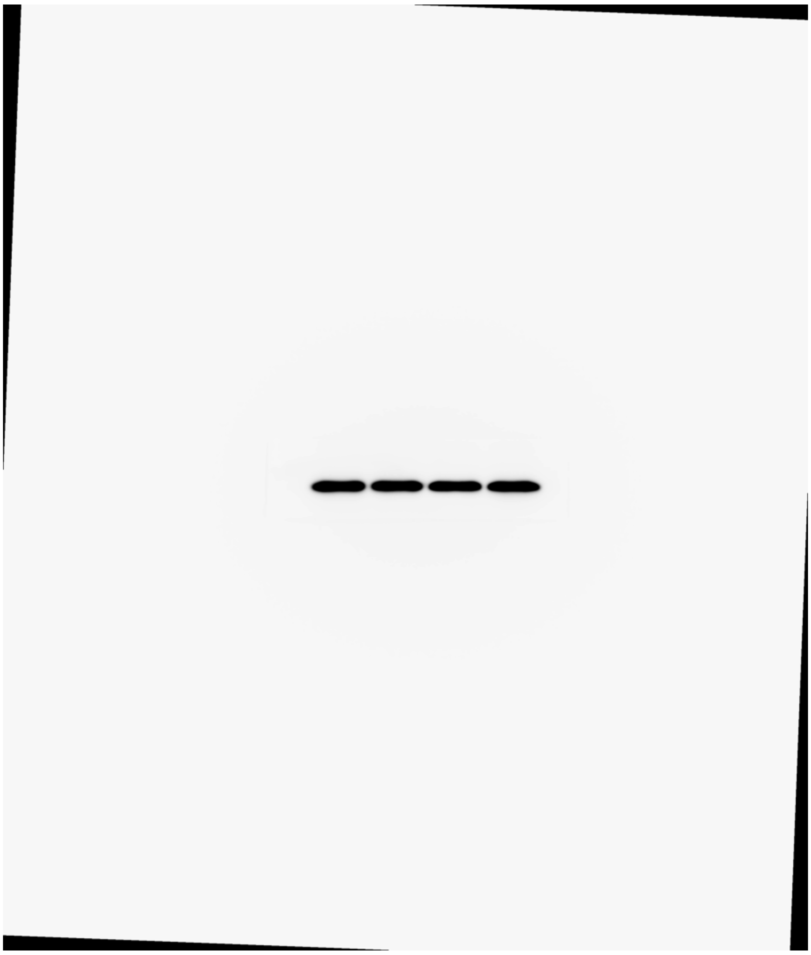

Full unedited gel for Figure 4C
PPFIA4 (from lane 1 to lane 4)

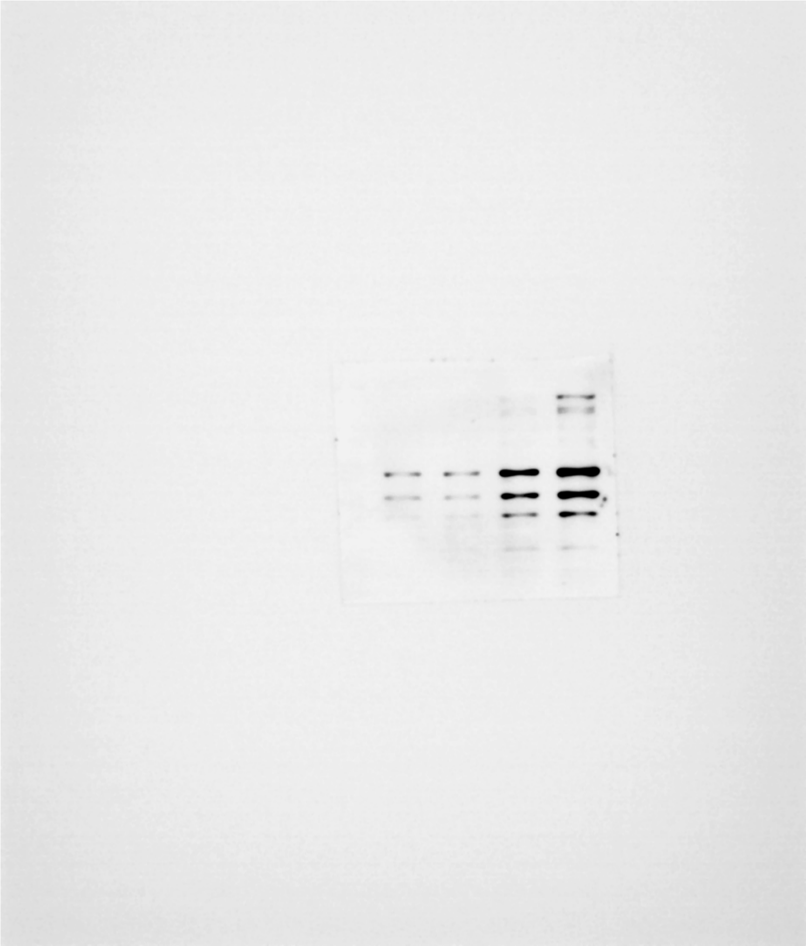

GAPDH (from lane 1 to lane 4)

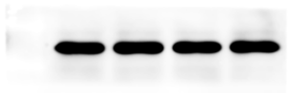

Full unedited gel for Figure 4D

PPFIA4 (from lane 1 to lane 4)

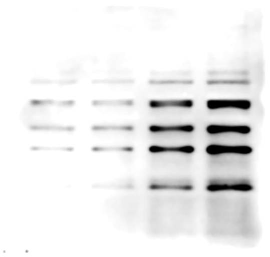

GAPDH (from lane 1 to lane 4)

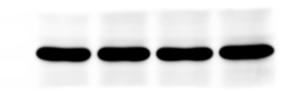

Full unedited gel for Figure 4E

PPFIA4 (from lane 1 to lane 4)

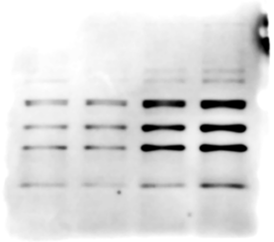

GAPDH (from lane 1 to lane 4)

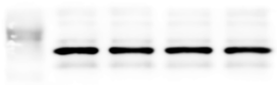

Full unedited gel for Figure 4F

PPFIA4 (from lane 1 to lane 4)

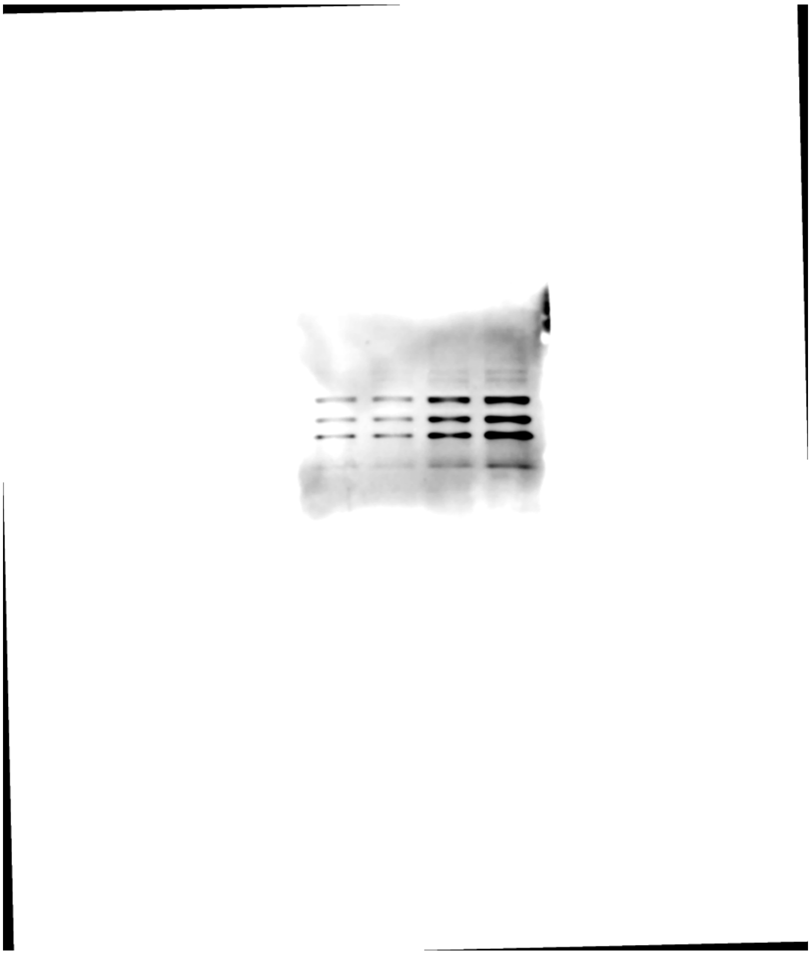

GAPDH (from lane 1 to lane 4)

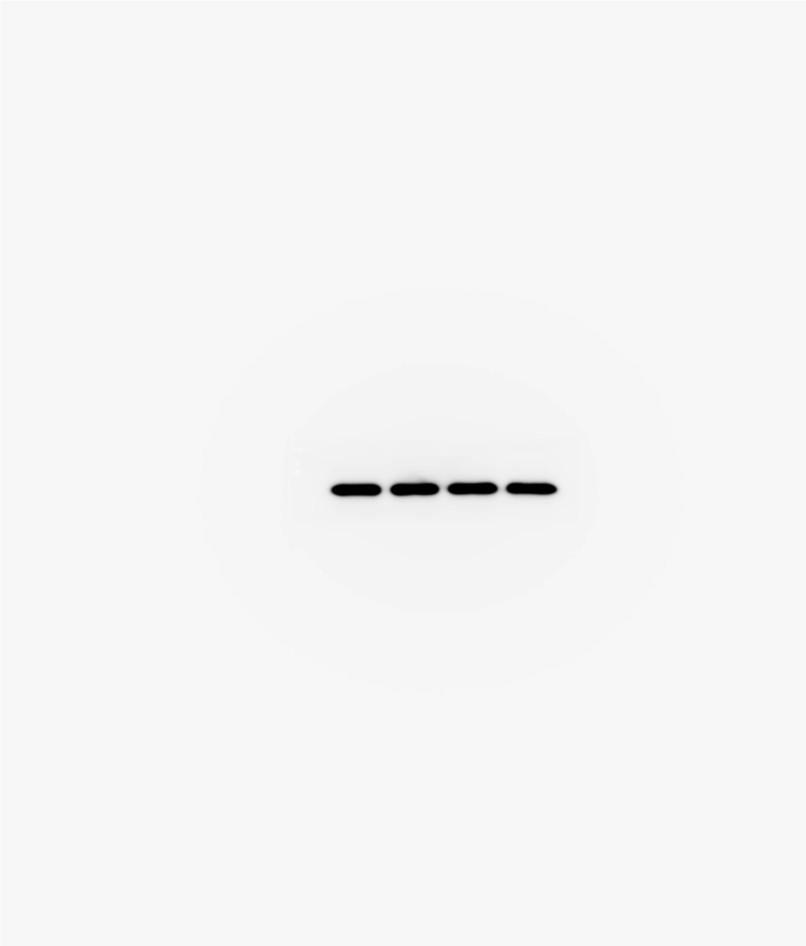

Full unedited gel for Figure 4G

PPFIA4 (from lane 1 to lane 4)

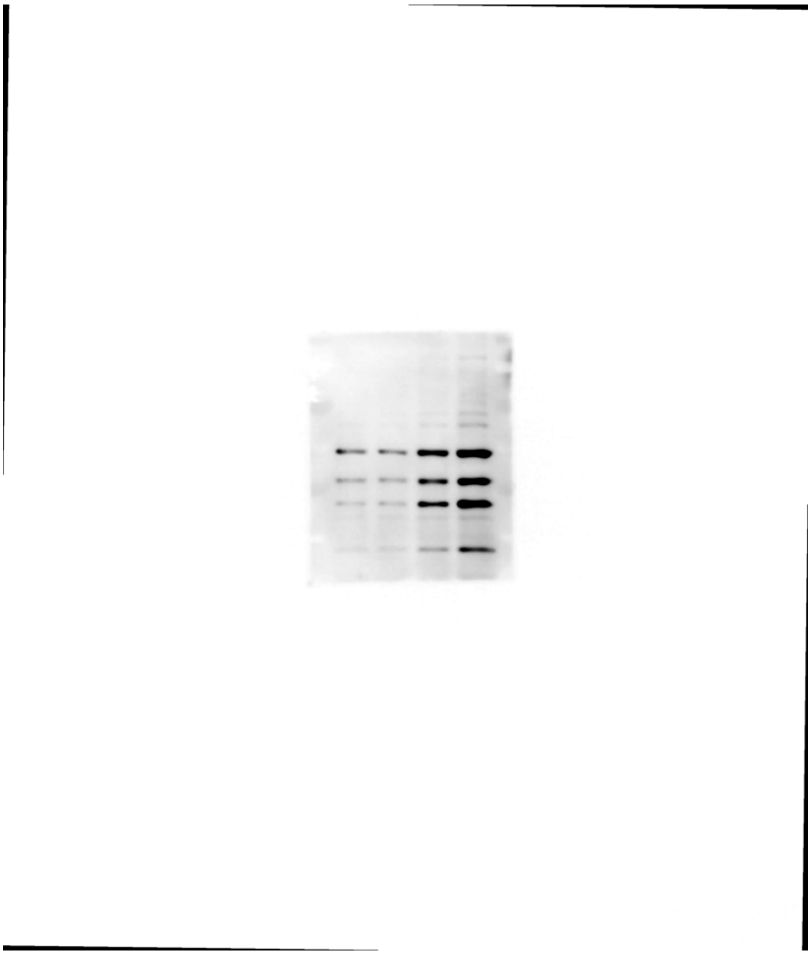

GAPDH (from lane 1 to lane 4)

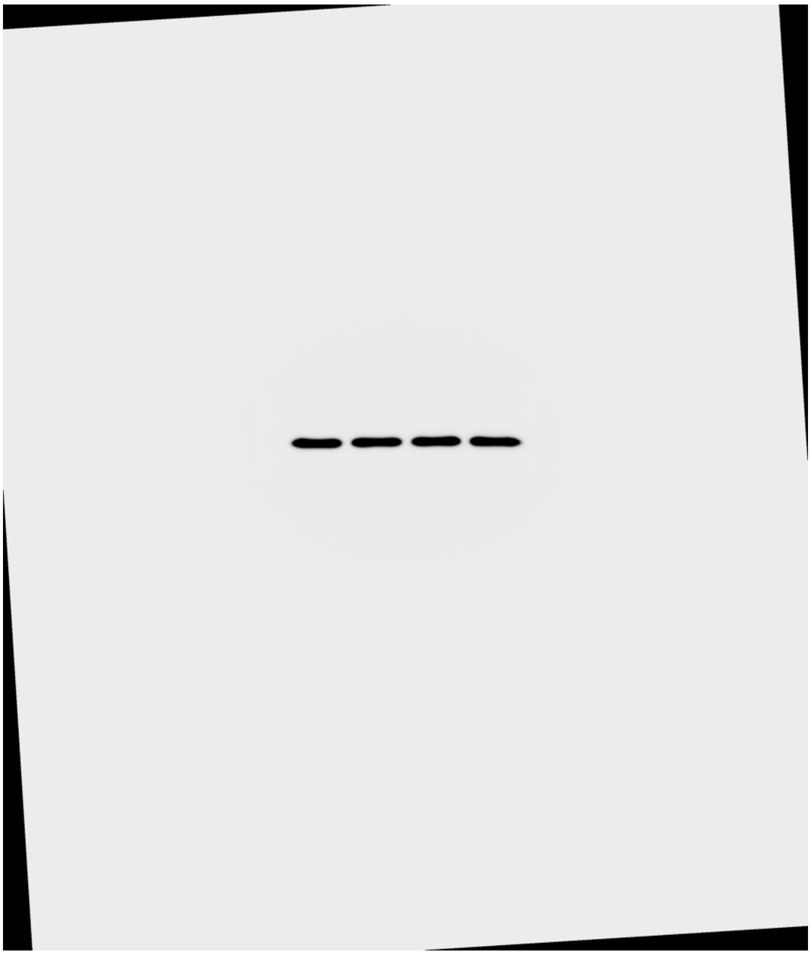

Full unedited gel for Figure 5A

PPFIA4 (from lane 1 to lane 8)

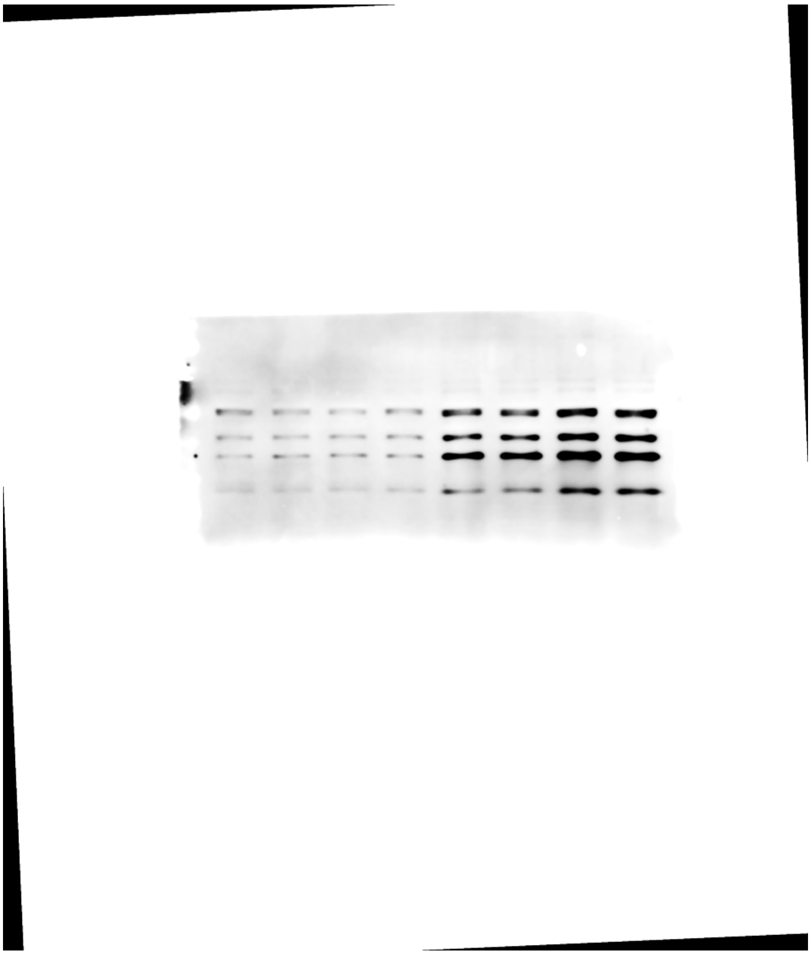

GAPDH (from lane 1 to lane 8)

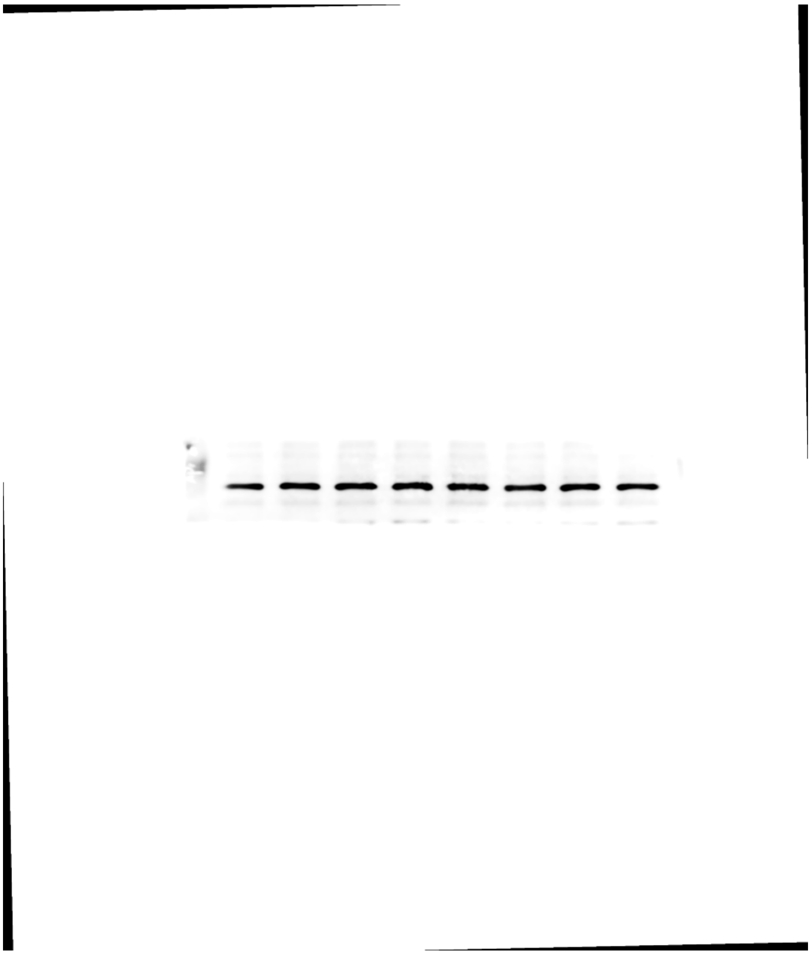

Full unedited gel for Figure 5D(up)

PPFIA4 (from lane 1 to lane 6)

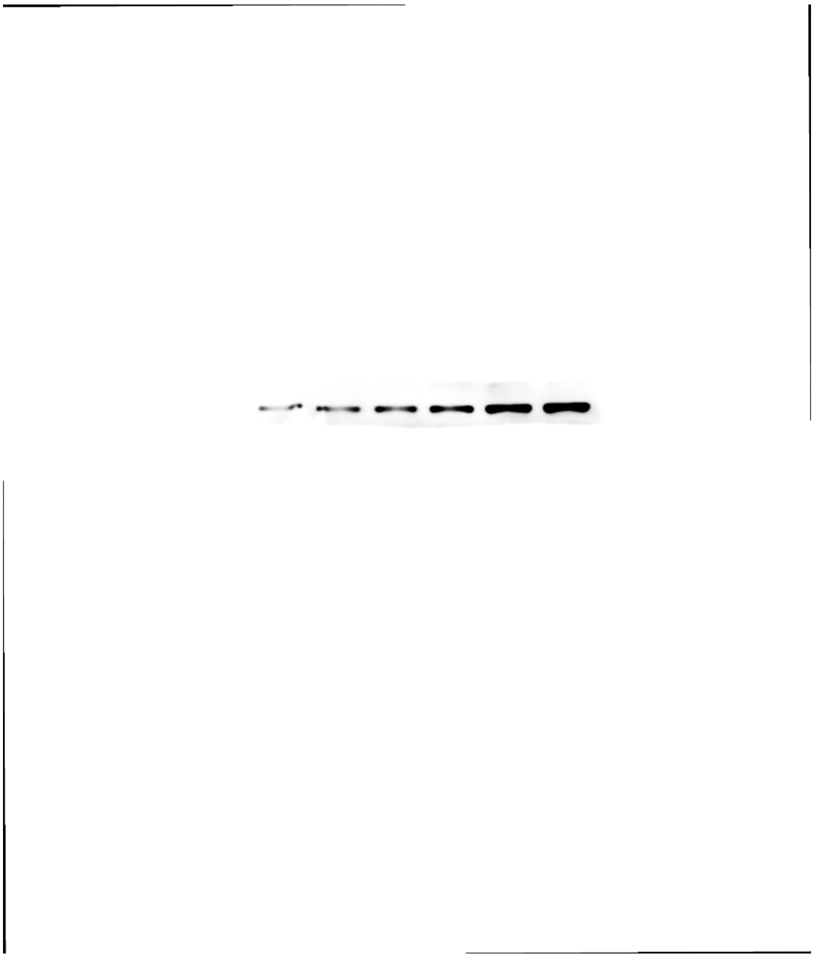

GAPDH (from lane 1 to lane 6)

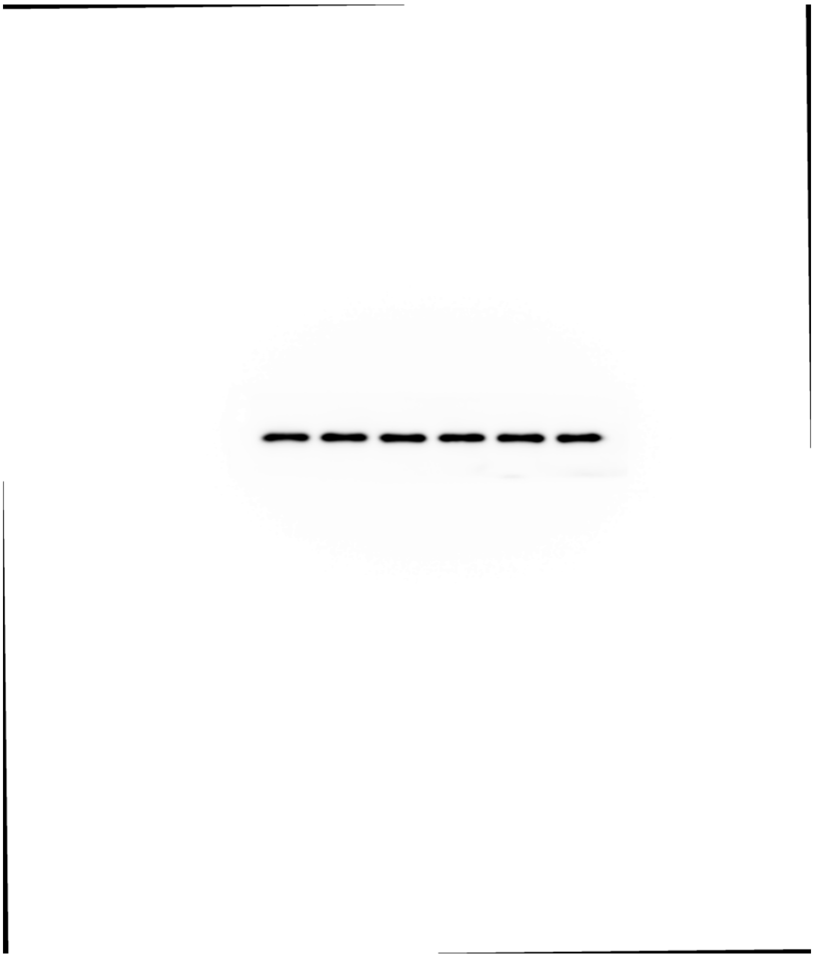

Full unedited gel for Figure 5D(down)

PPFIA4 (from lane 1 to lane 6)

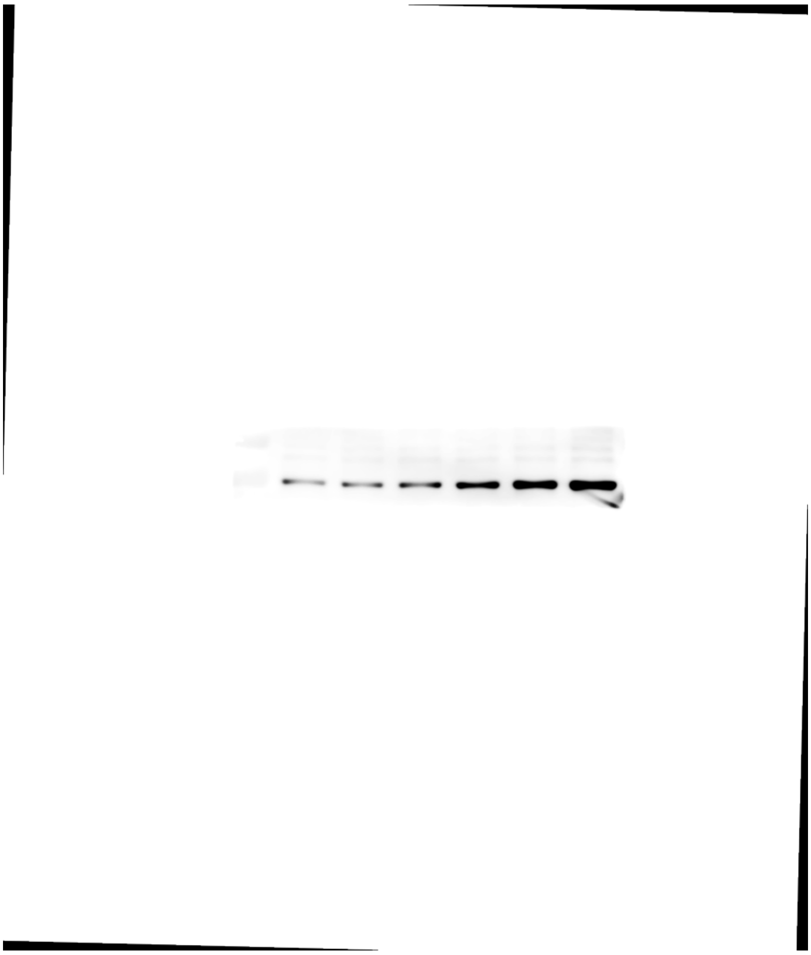

GAPDH (from lane 1 to lane 6)

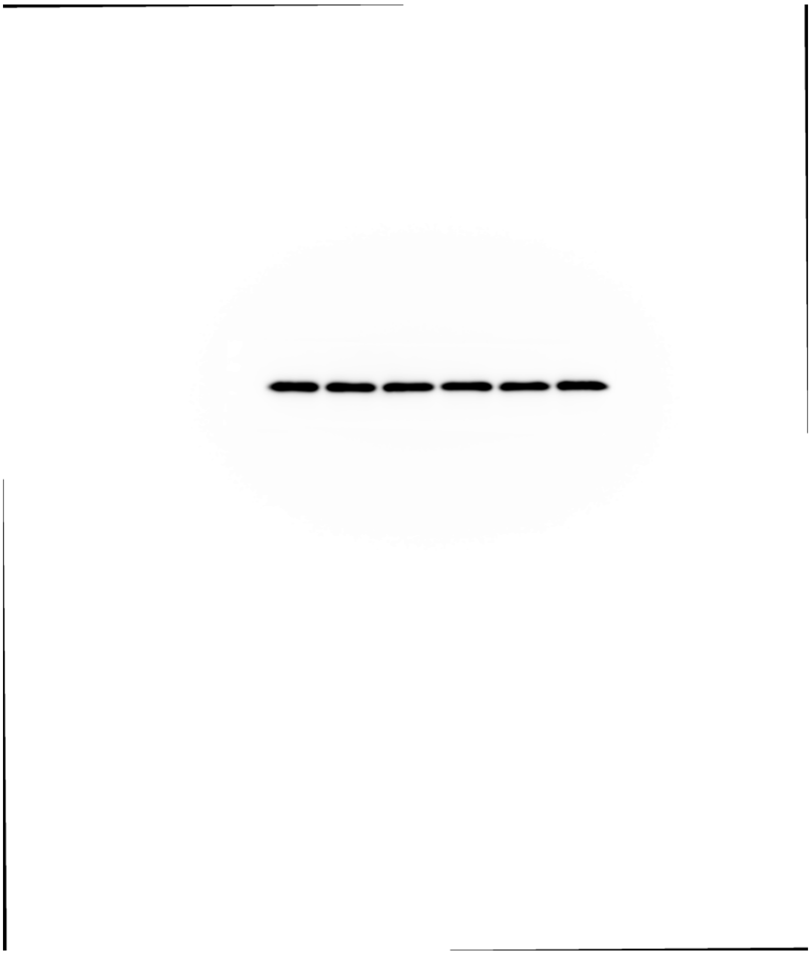

Full unedited gel for Figure 5E

PPFIA4 (from lane 1 to lane 8)

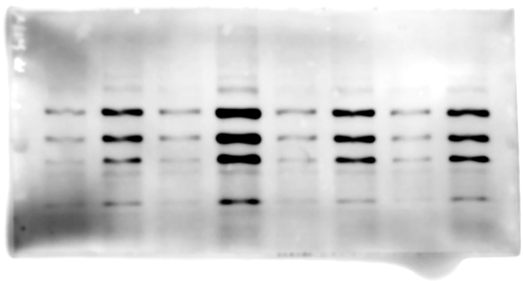

p-c-Jun (from lane 1 to lane 8)

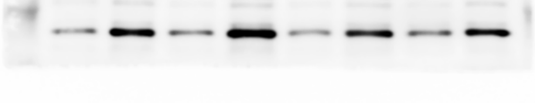

c-Jun (from lane 1 to lane 8)

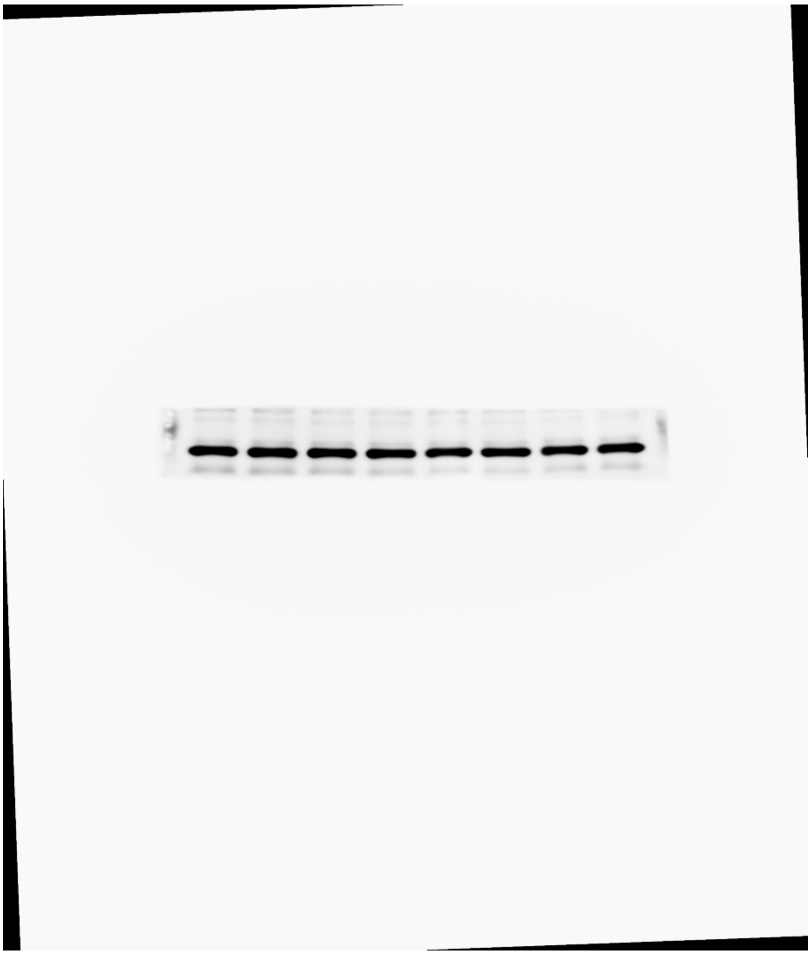

GAPDH (from lane 1 to lane 8)

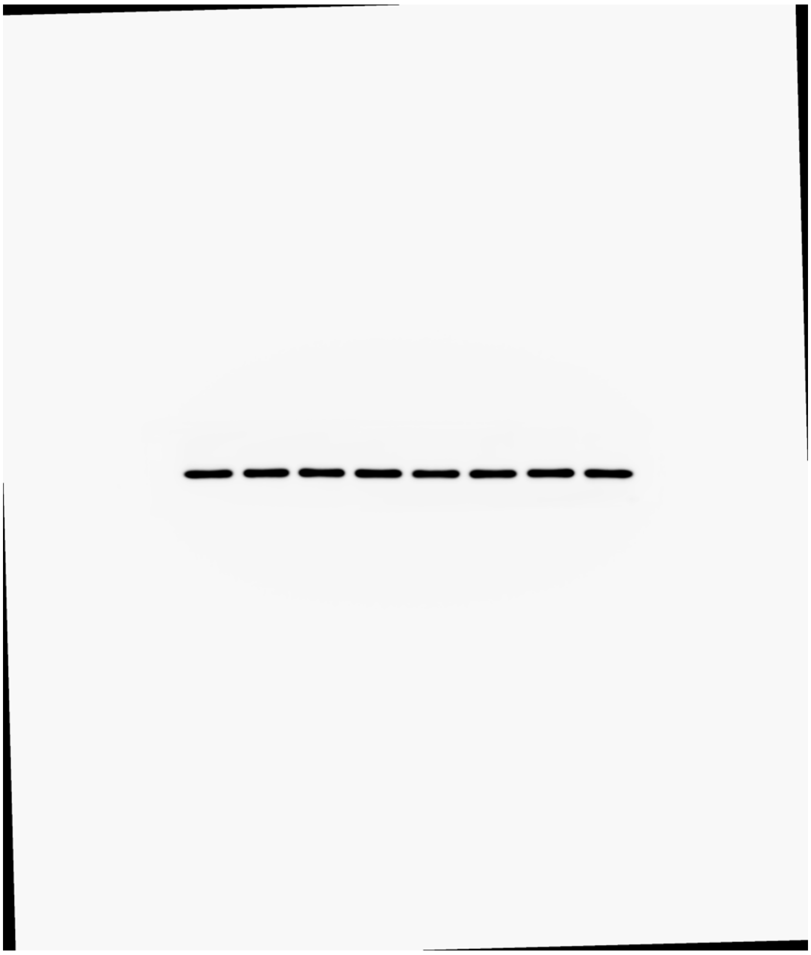

Full unedited gel for Figure 5F

PPFIA4 (from lane 1 to lane 8)

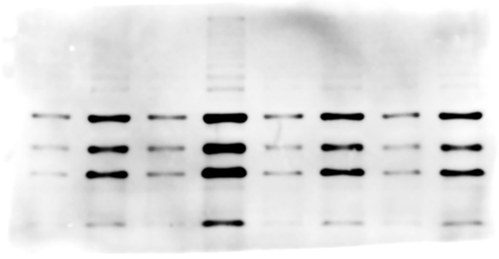

p-c-Jun (from lane 1 to lane 8)

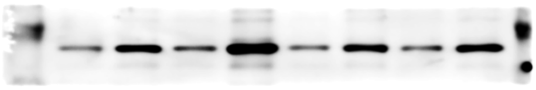

c-Jun (from lane 1 to lane 8)

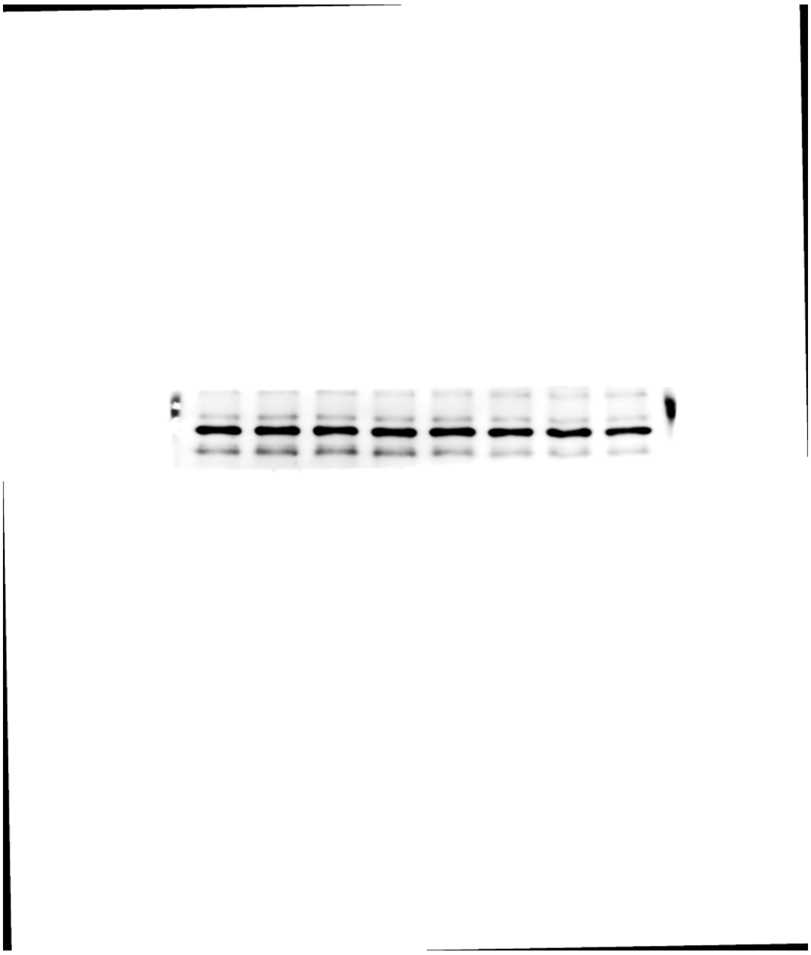

GAPDH (from lane 1 to lane 8)

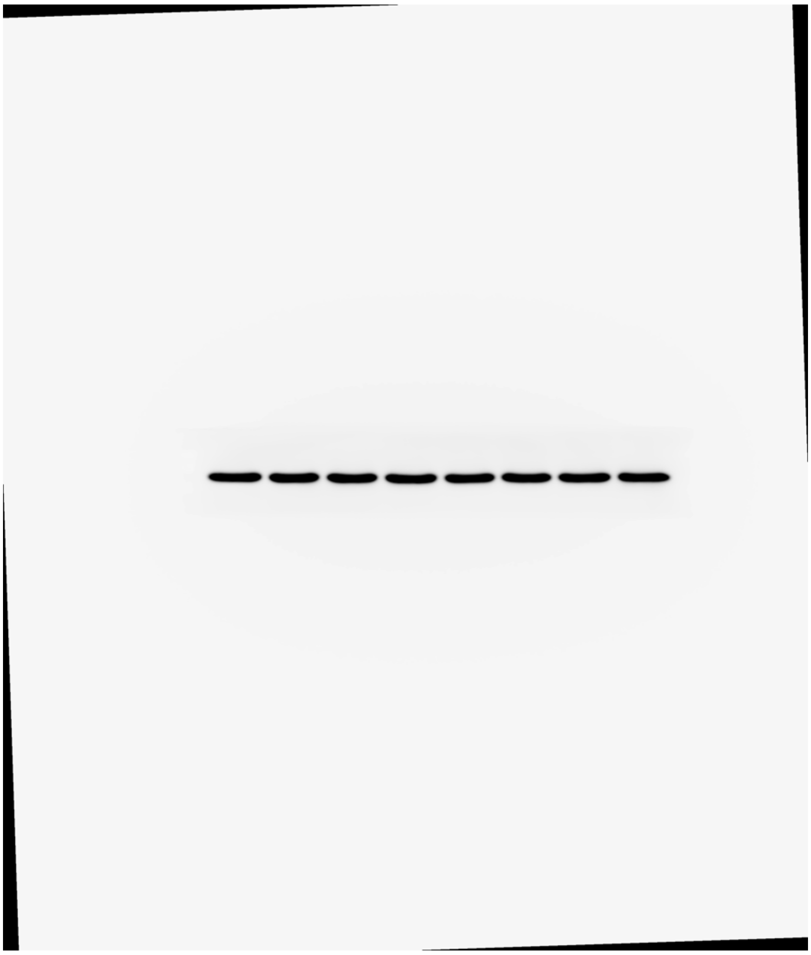

Full unedited gel for Figure 5G

PPFIA4 (from lane 1 to lane 7)

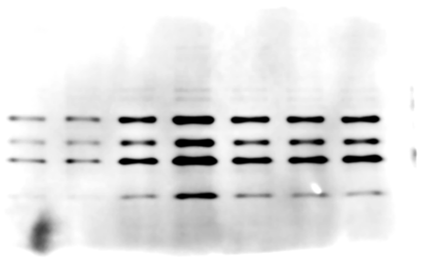

p-c-Jun (from lane 1 to lane 7)

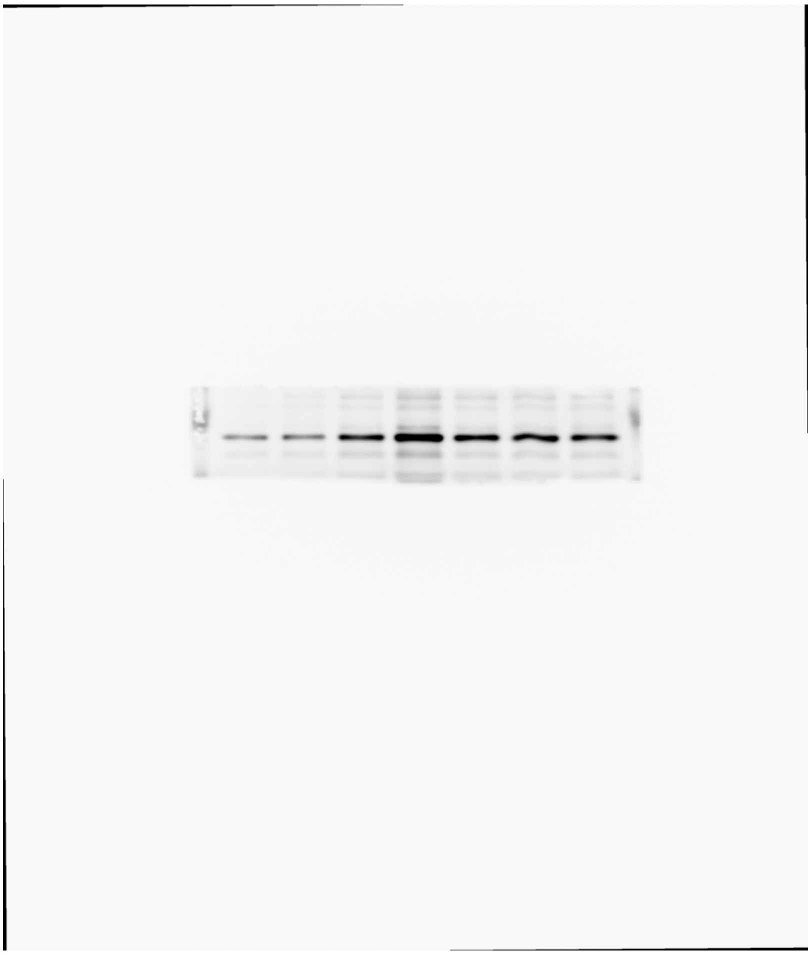

c-Jun (from lane 1 to lane 7)

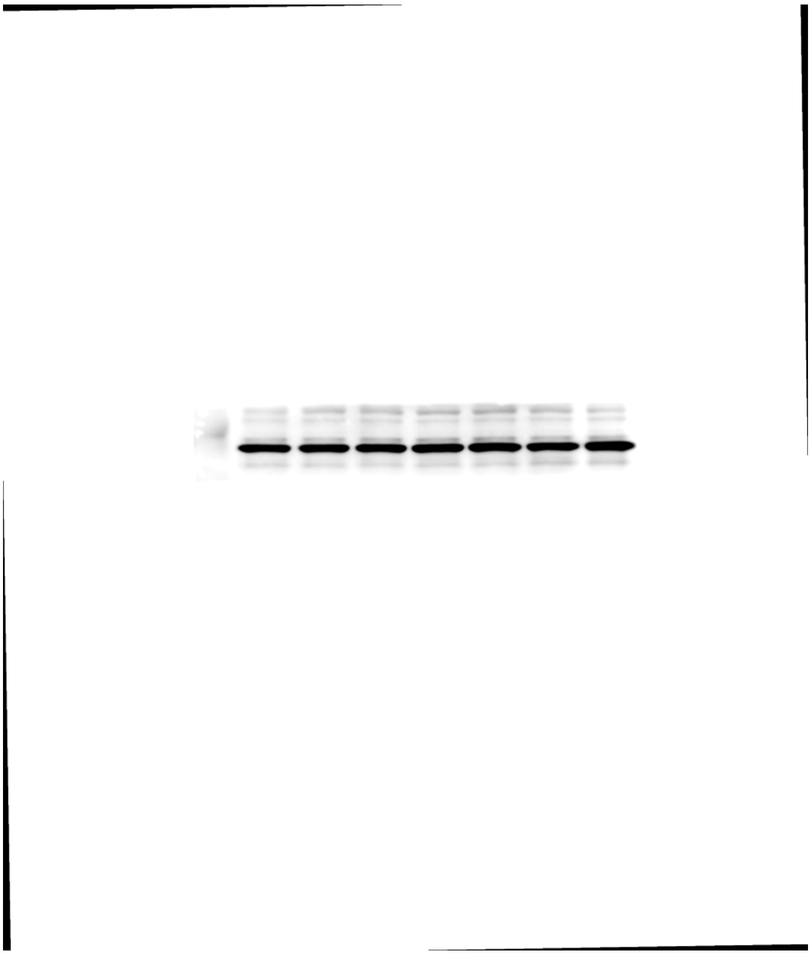

GAPDH (from lane 1 to lane 7)

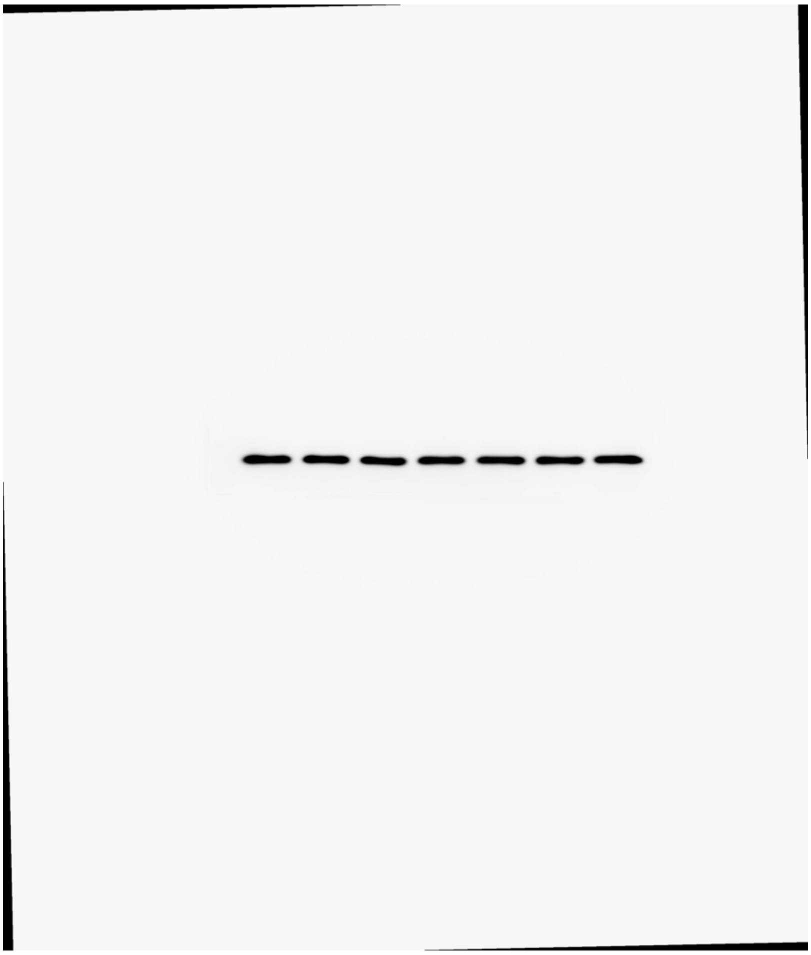

Full unedited gel for Figure 5H

PPFIA4 (from lane 1 to lane 6)

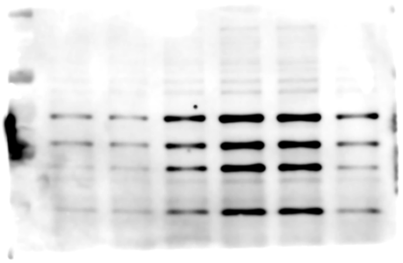

p-c-Jun (from lane 1 to lane 6)

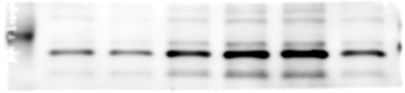

c-Jun (from lane 1 to lane 6)

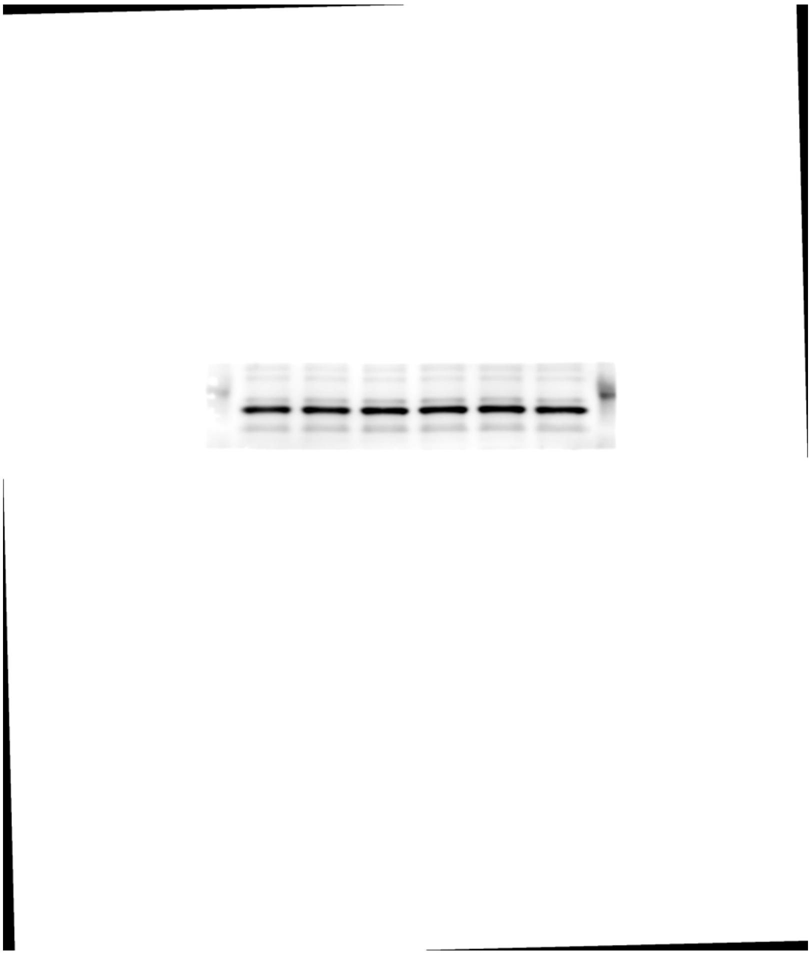

GAPDH (from lane 1 to lane 6)

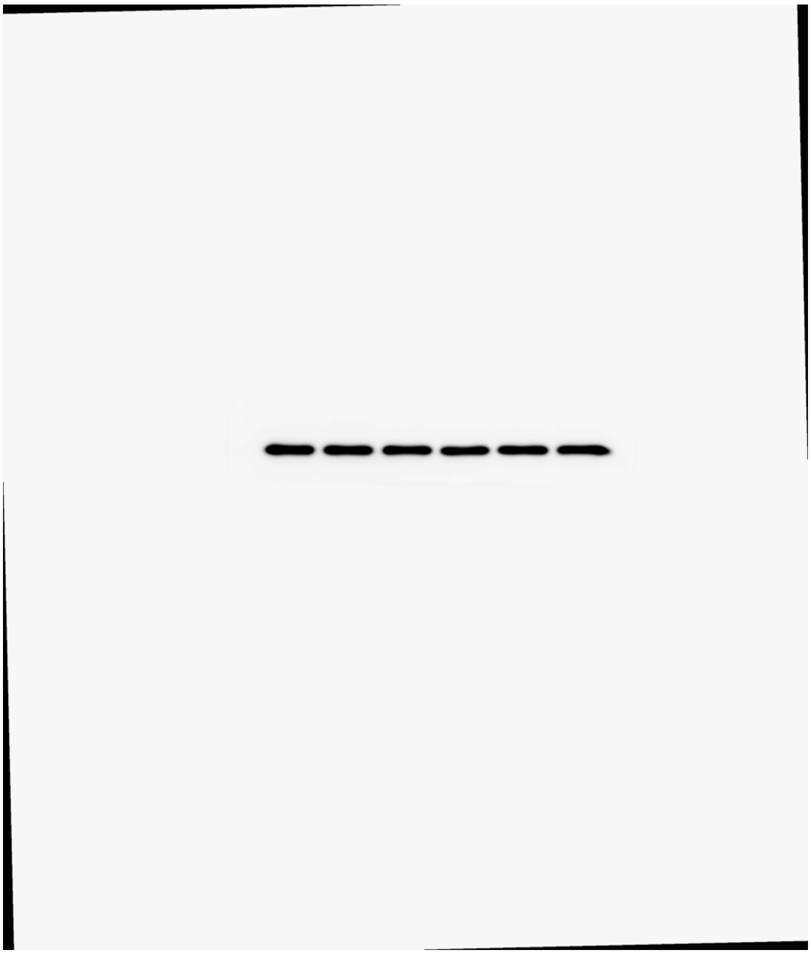

Full unedited gel for Figure 8E(up left panel)

MMP1 (from lane 1 to lane 4)

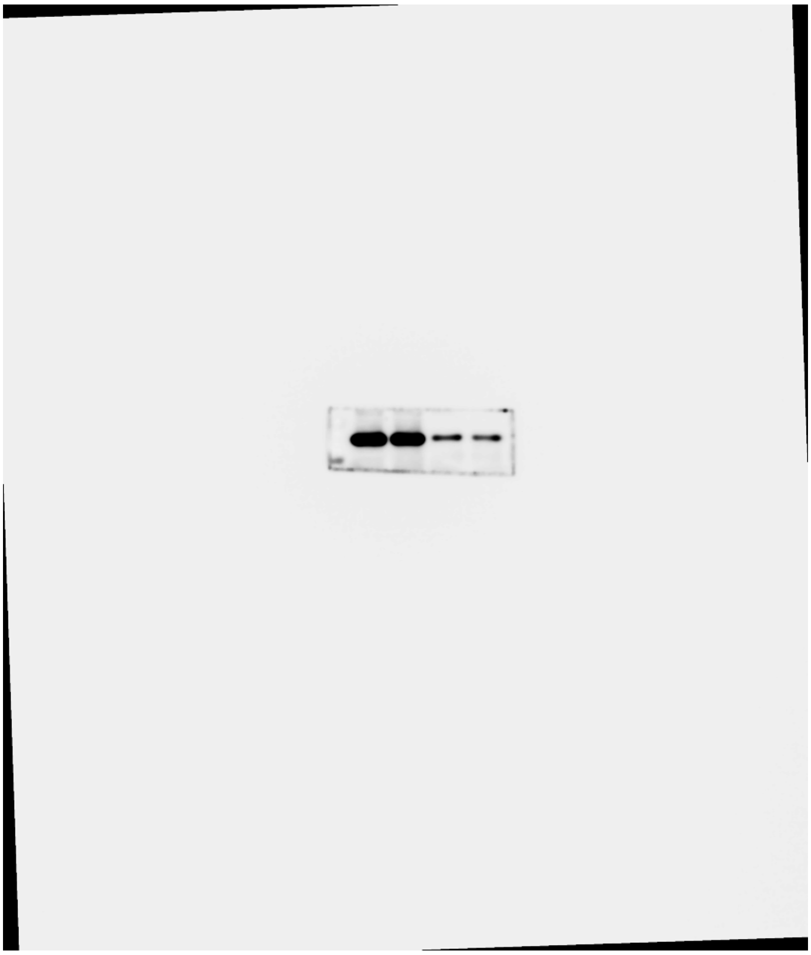

E-Cad (from lane 1 to lane 4)

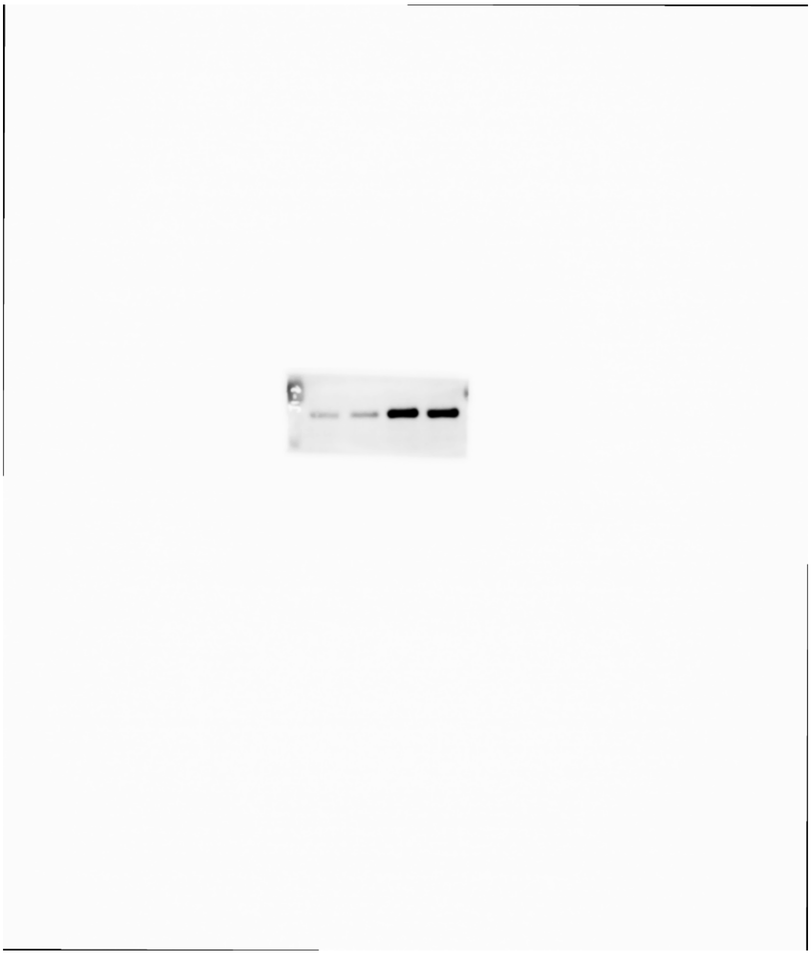

ZO-1 (from lane 1 to lane 4)

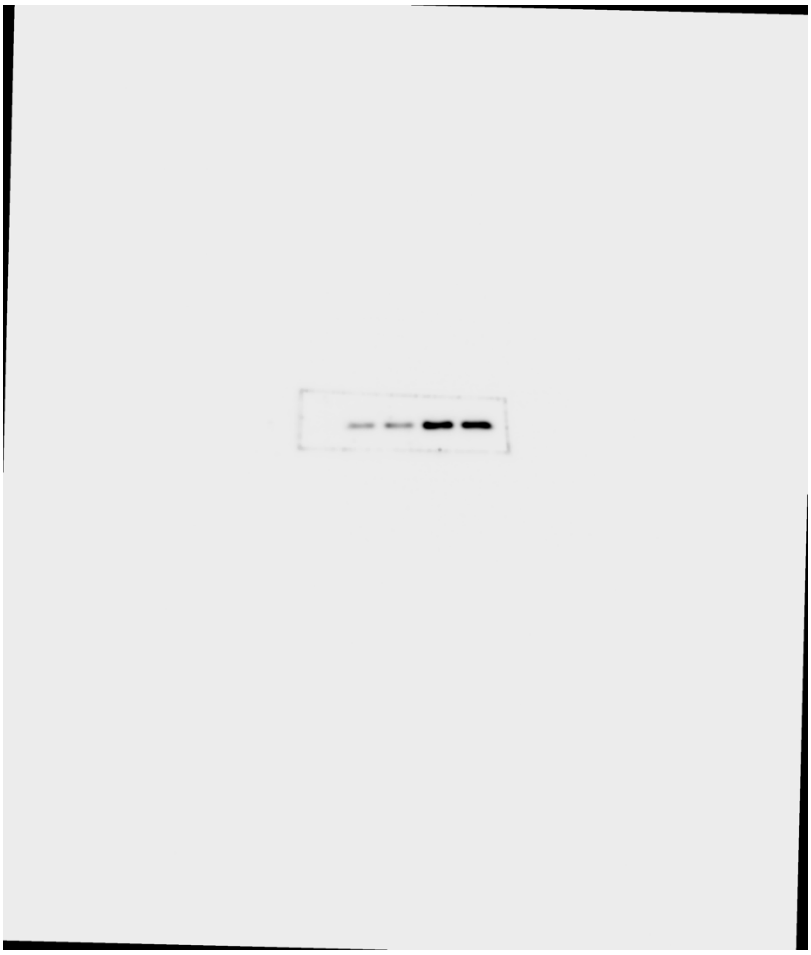

GAPDH (from lane 1 to lane 4)

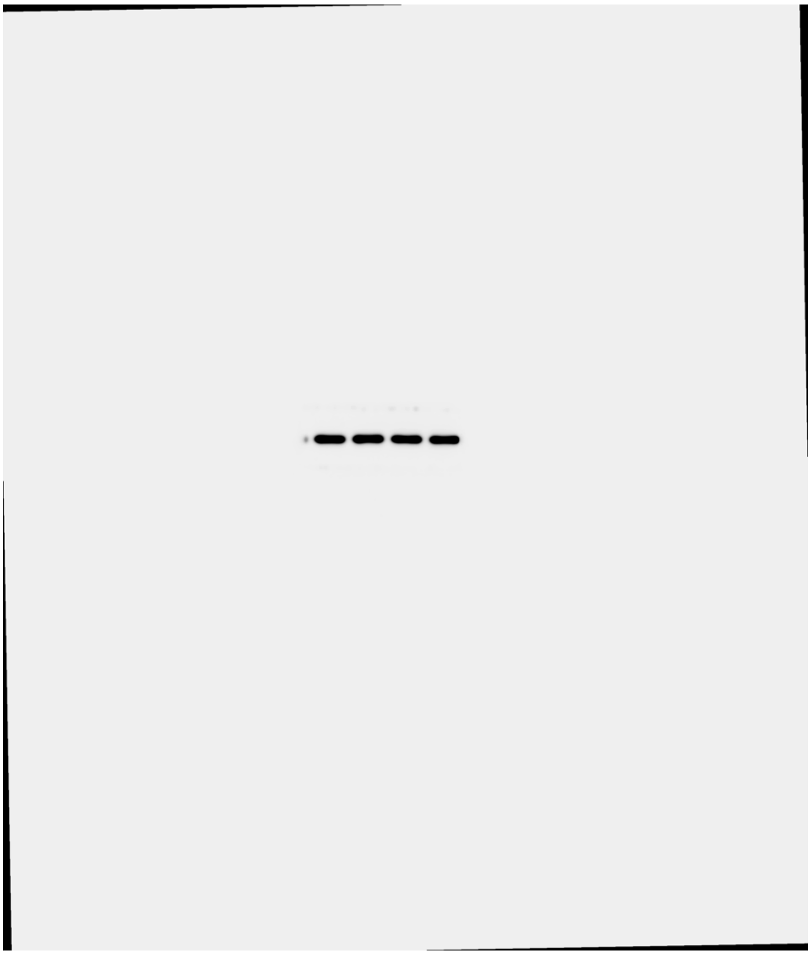

Full unedited gel for Figure 8E(up right panel)

MMP1 (from lane 1 to lane 10)

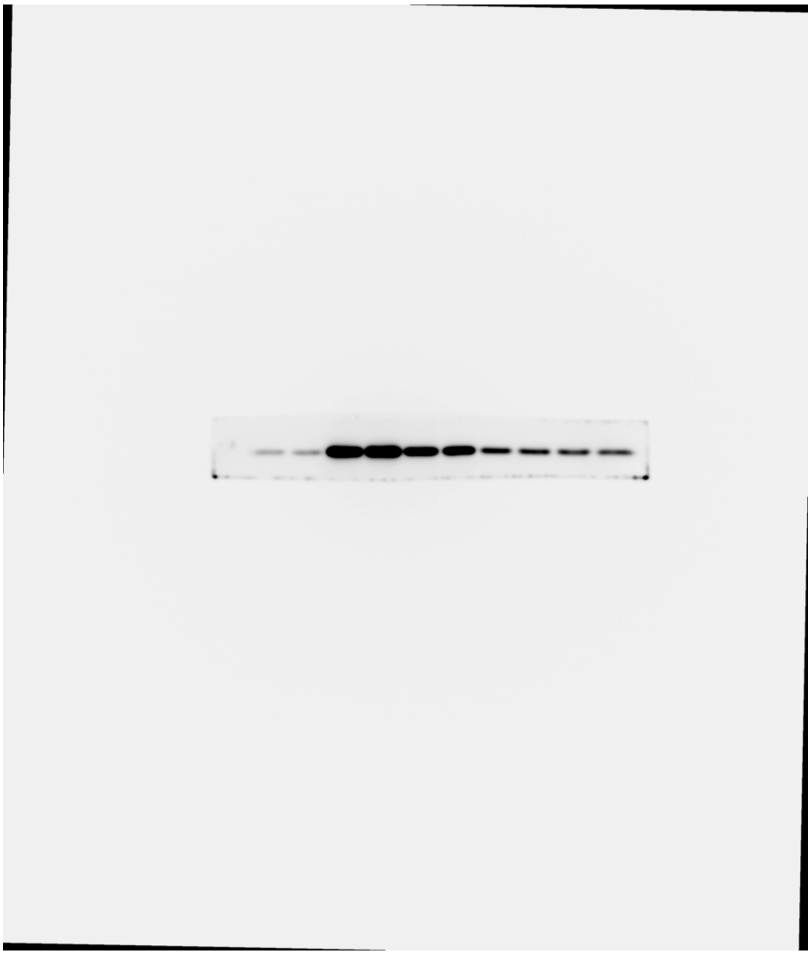

E-Cad (from lane 1 to lane 10)

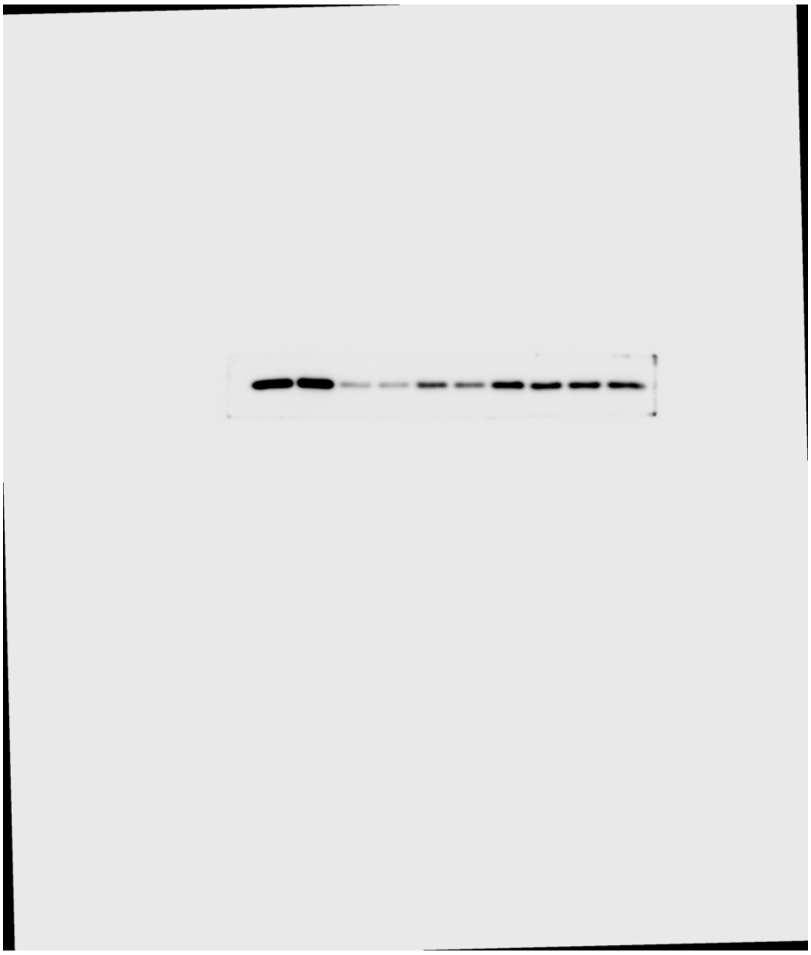

ZO-1 (from lane 1 to lane 10)

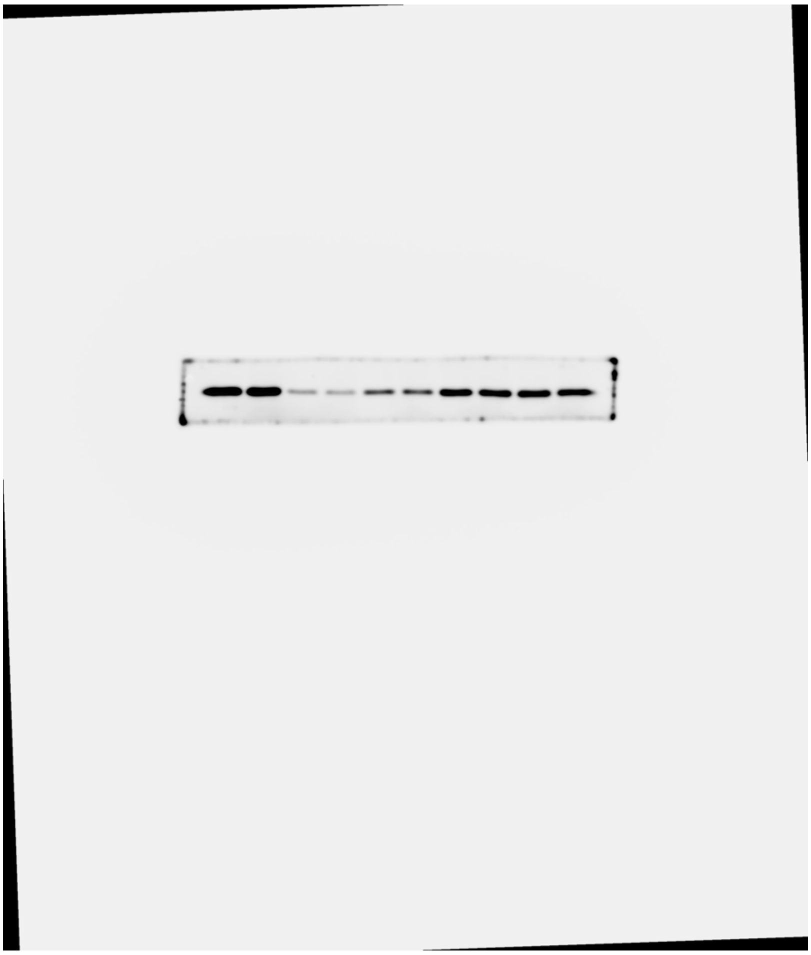

GAPDH (from lane 1 to lane 10)

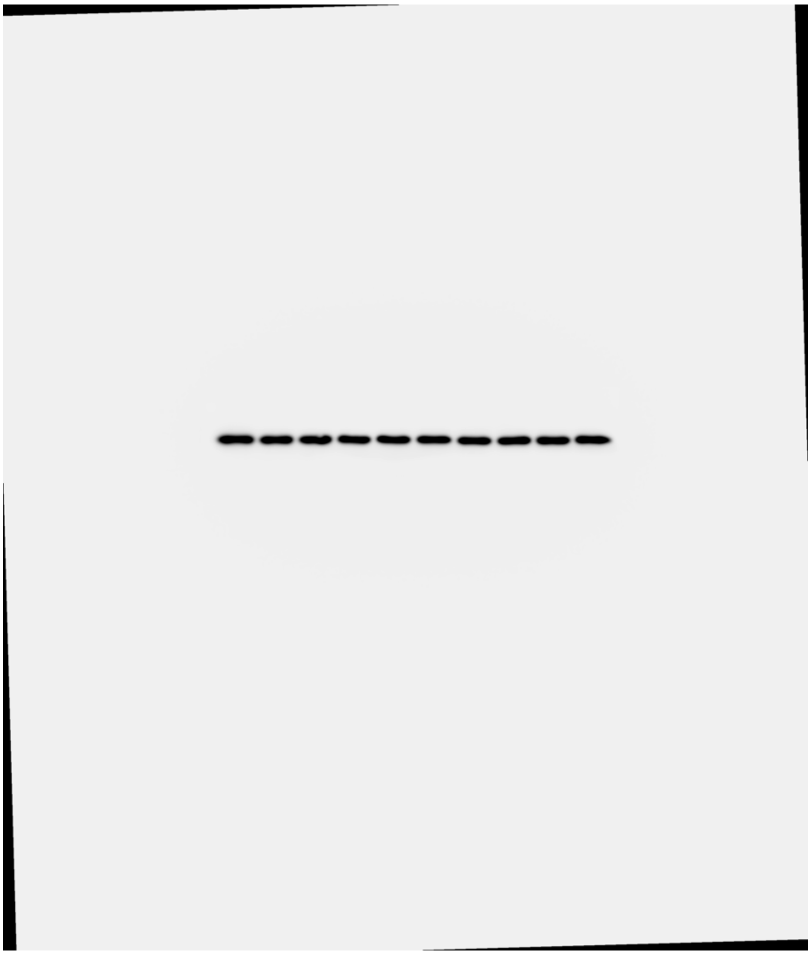

Full unedited gel for Figure 8E(down left panel)

MMP1 (from lane 1 to lane 4)

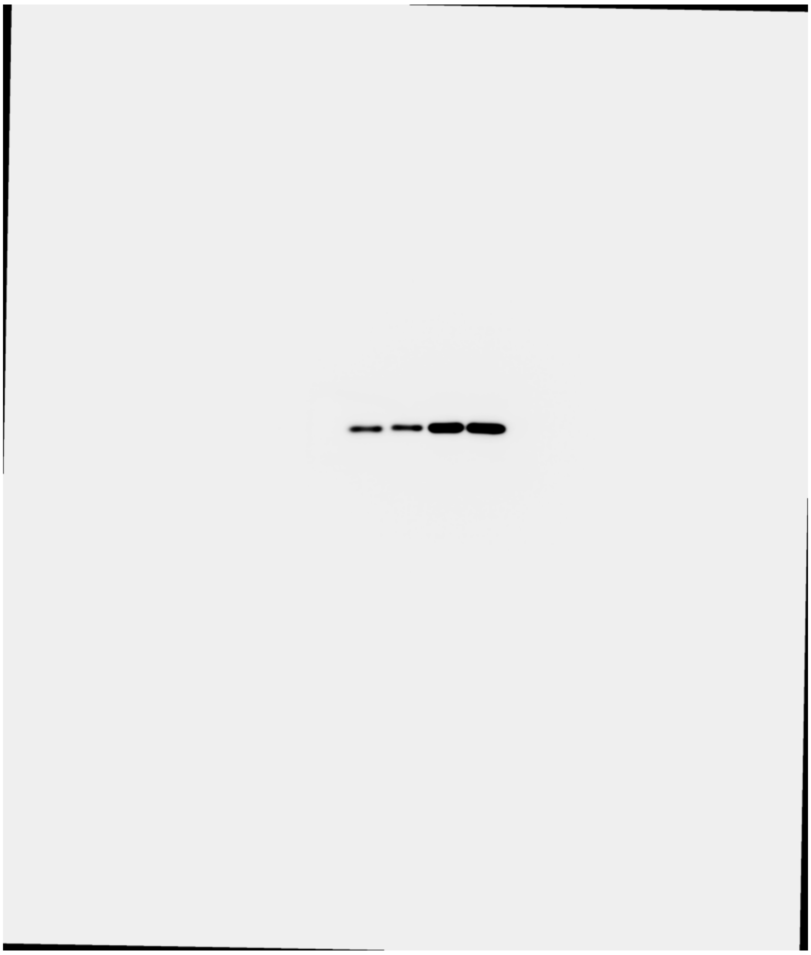

E-Cad (from lane 1 to lane 4)

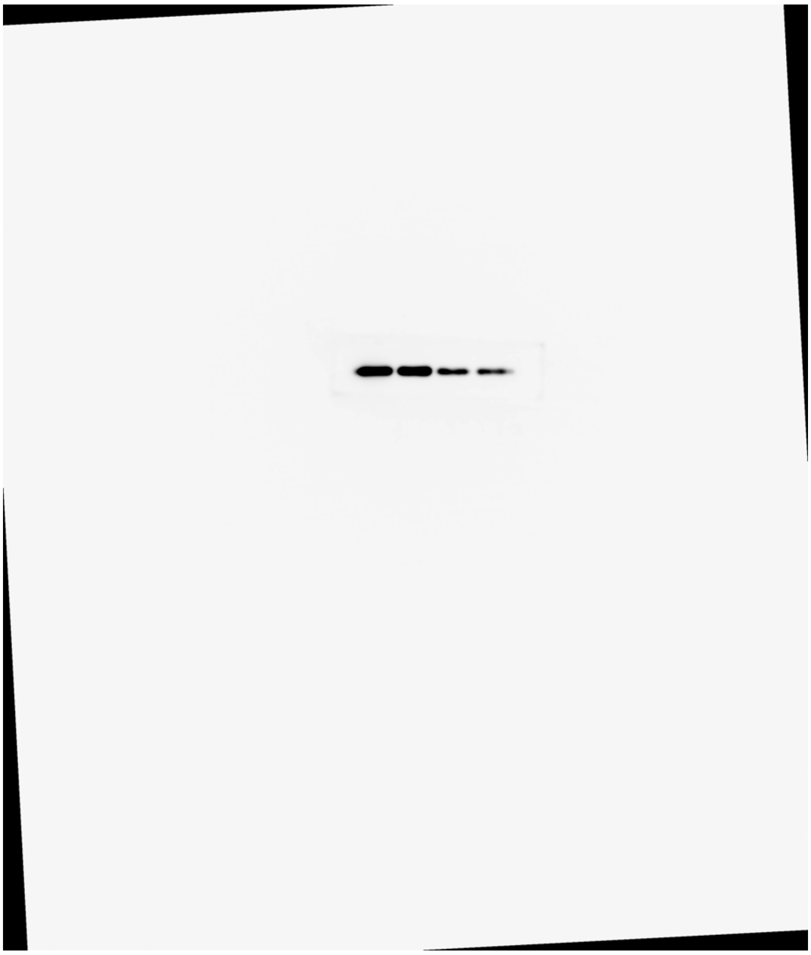

ZO-1 (from lane 1 to lane 4)

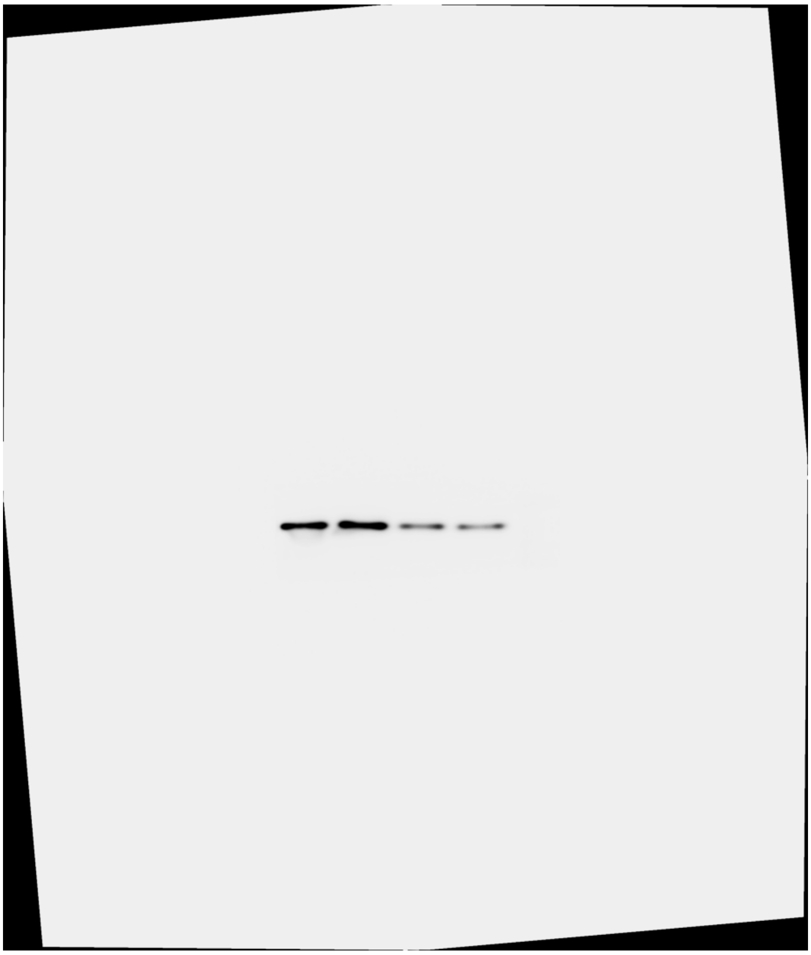

GAPDH (from lane 1 to lane 4)

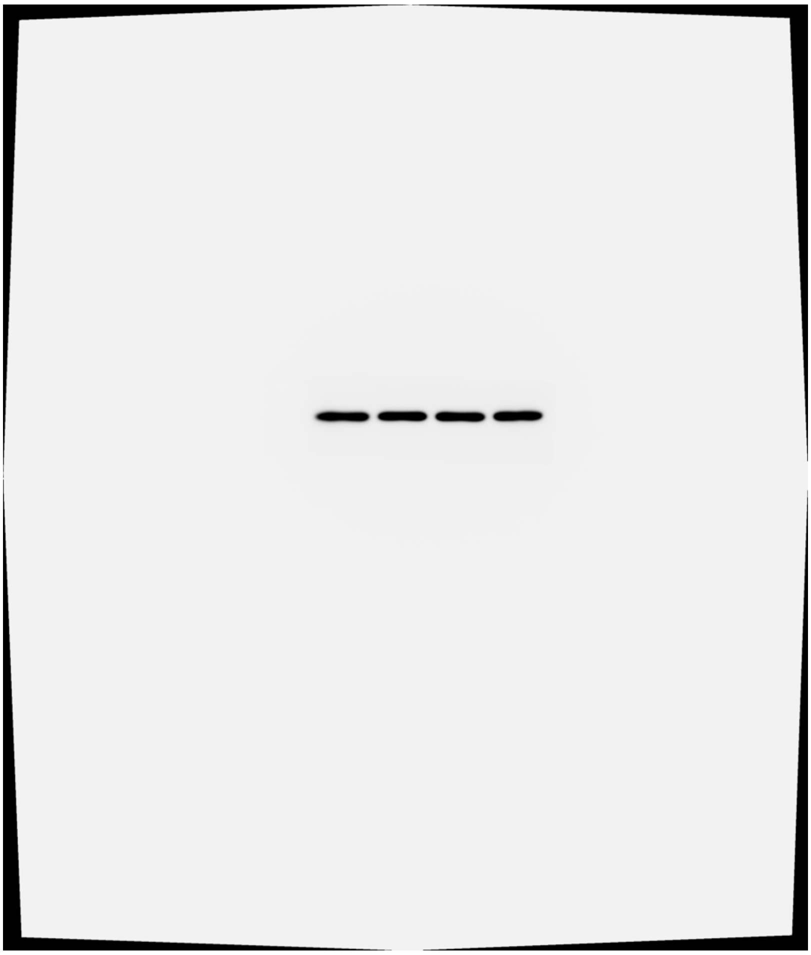

Full unedited gel for Figure 8E(down middle panel)

MMP1 (from lane 1 to lane 8)

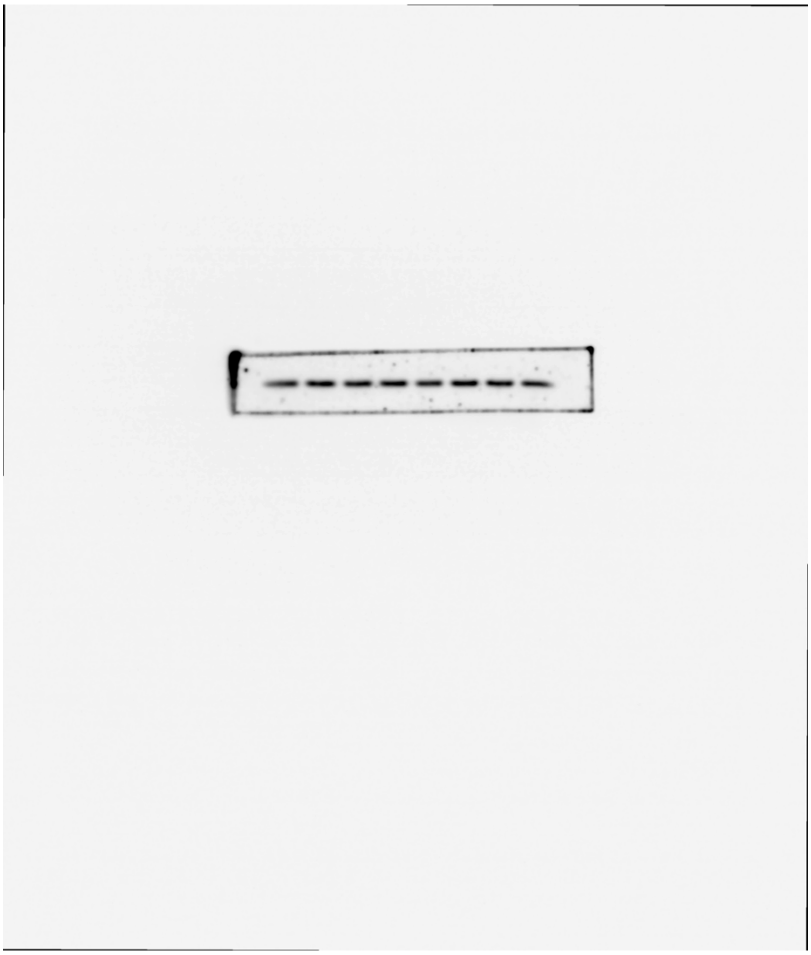

E-Cad (from lane 1 to lane 8)

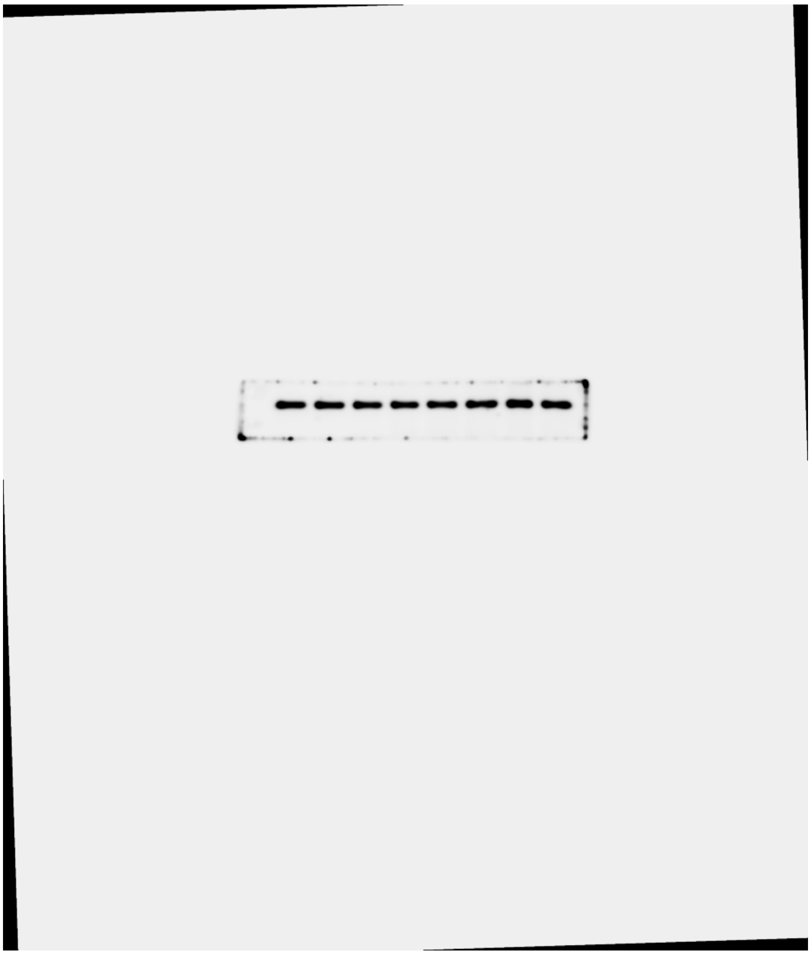

ZO-1 (from lane 1 to lane 8)

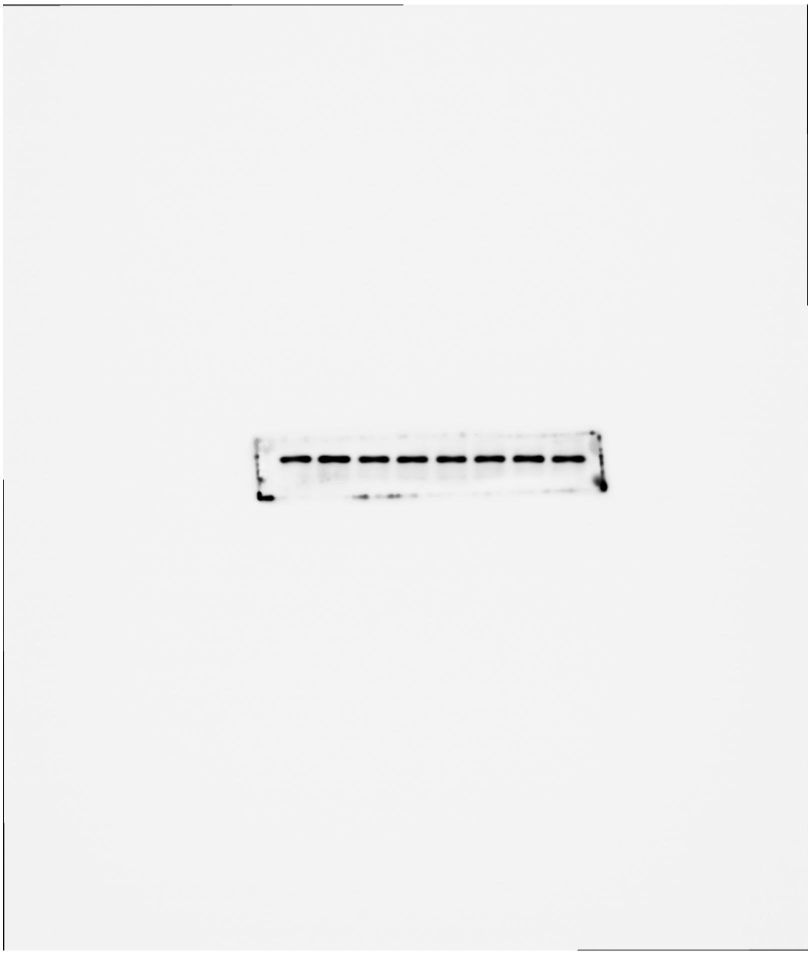

GAPDH (from lane 1 to lane 8)

Full unedited gel for Figure 8E(down right panel)
MMP1 (from lane 1 to lane 8)

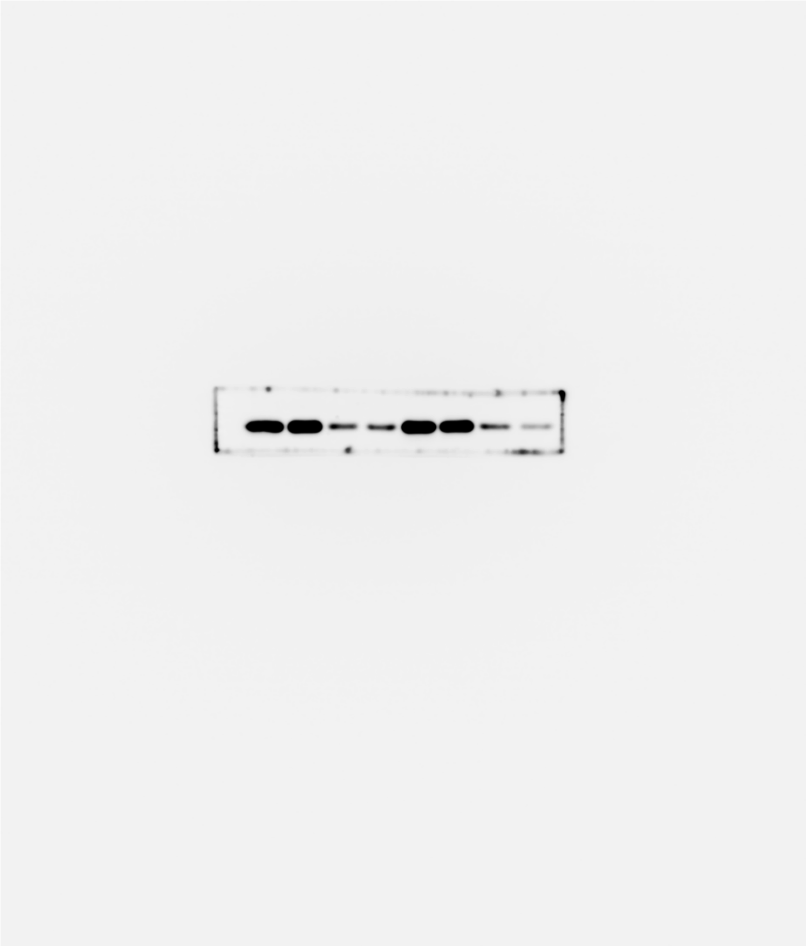

E-Cad (from lane 1 to lane 8)

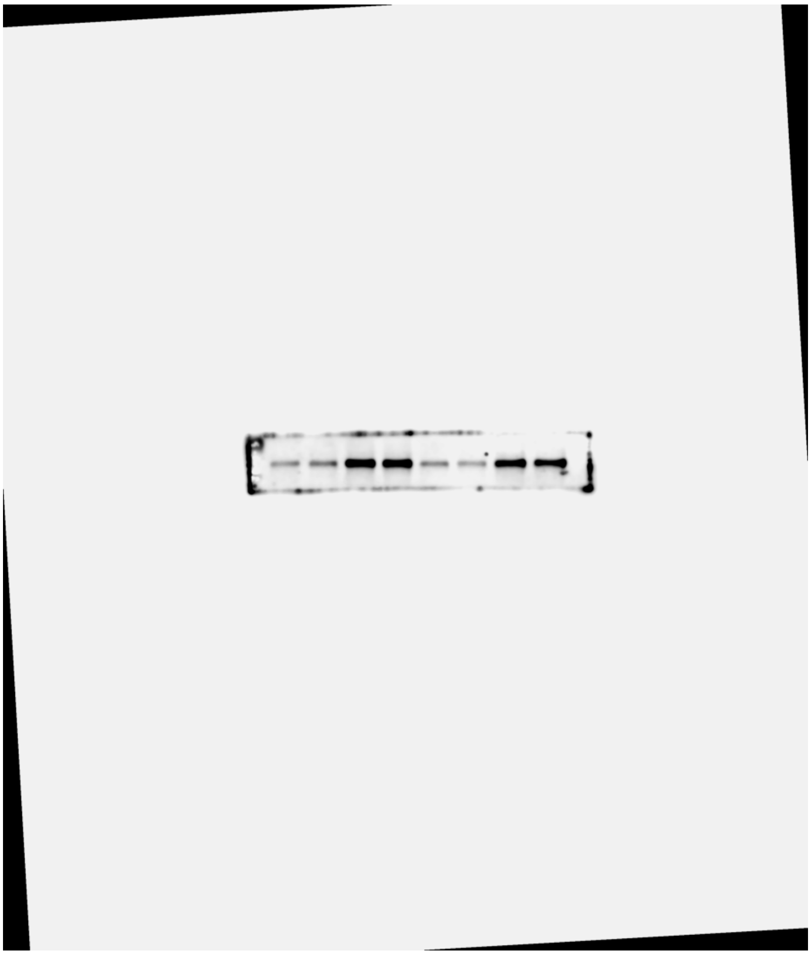

ZO-1 (from lane 1 to lane 8)

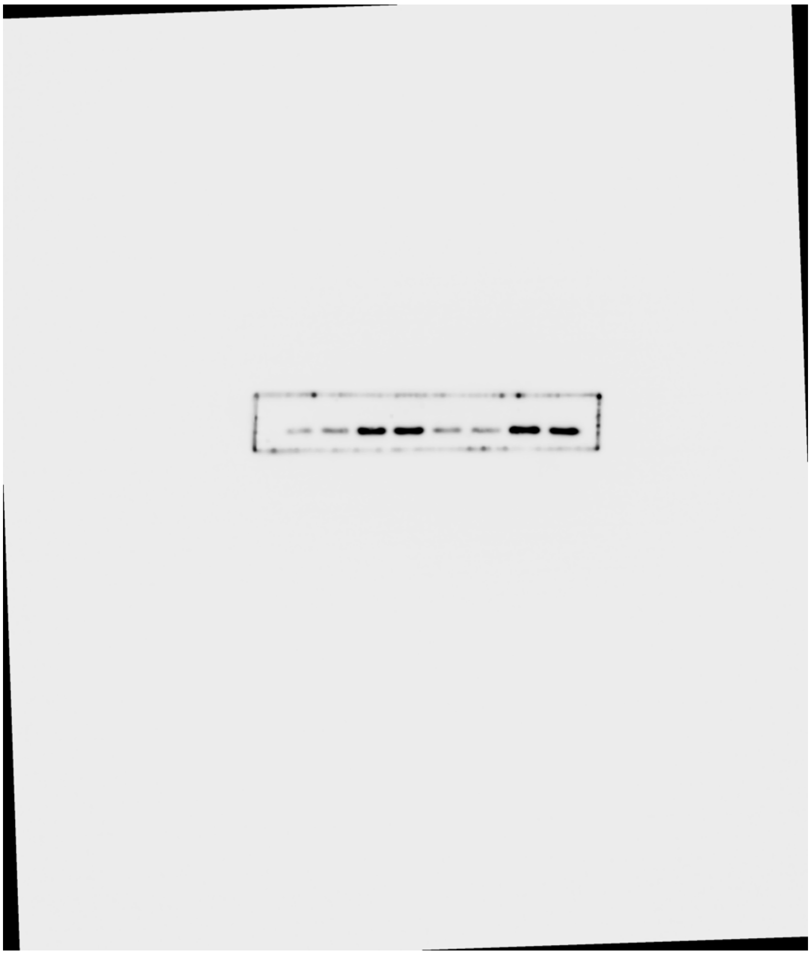

GAPDH (from lane 1 to lane 8)

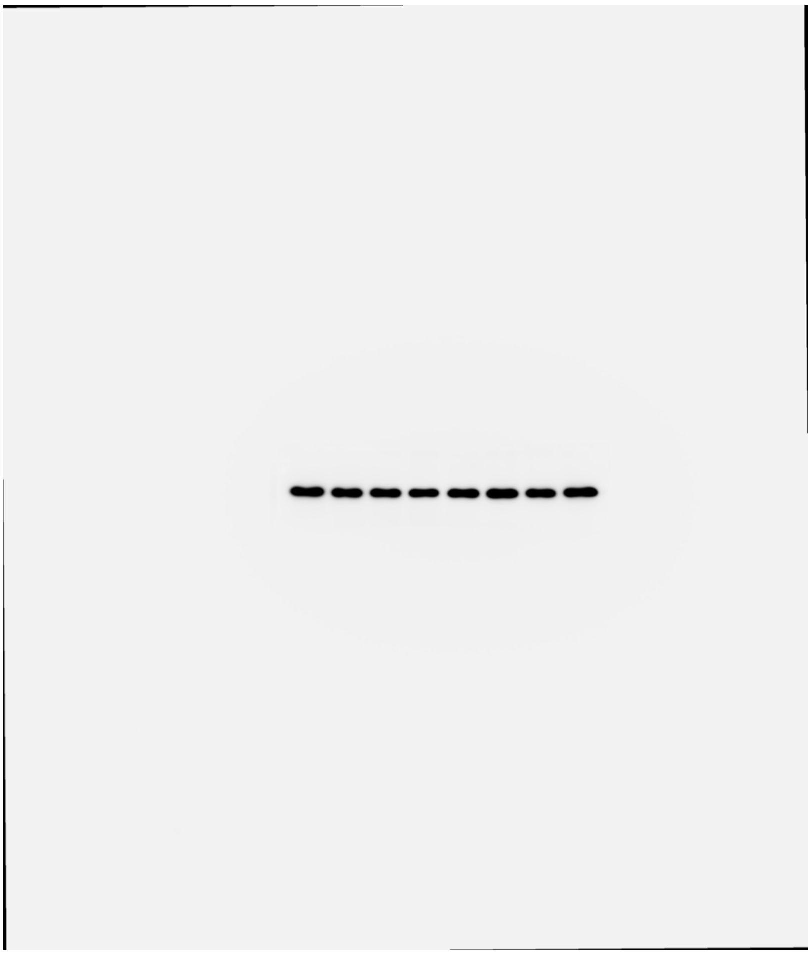

Full unedited gel for Figure 8F(left)

E-Cad (from lane 1 to lane 8)

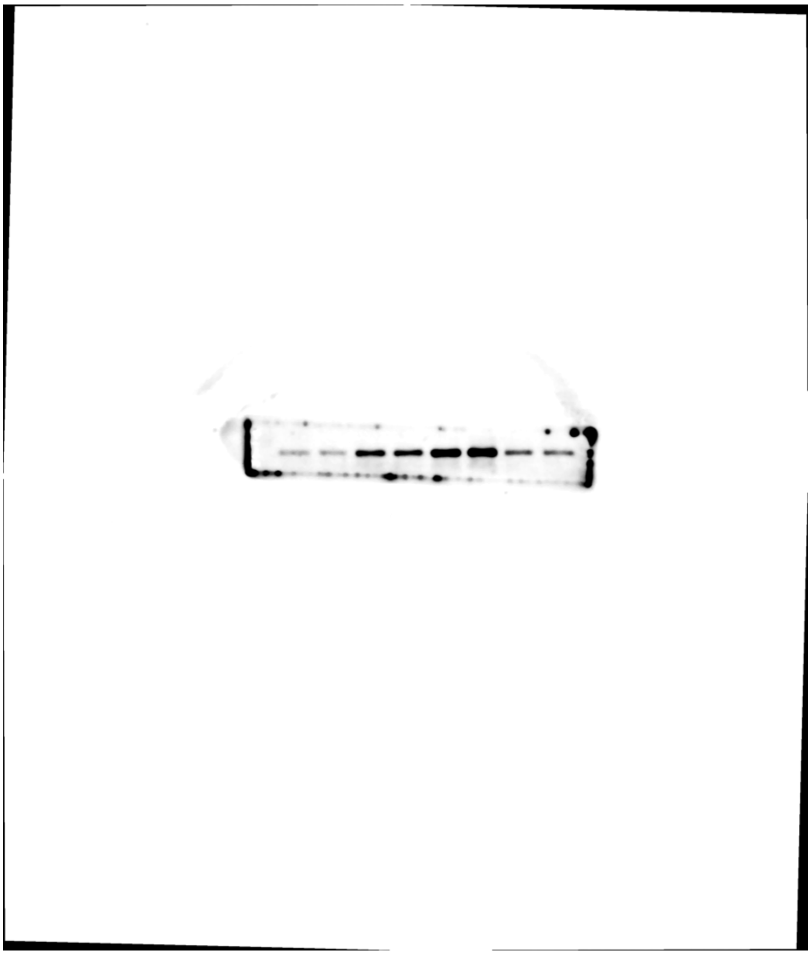

ZO-1 (from lane 1 to lane 8)

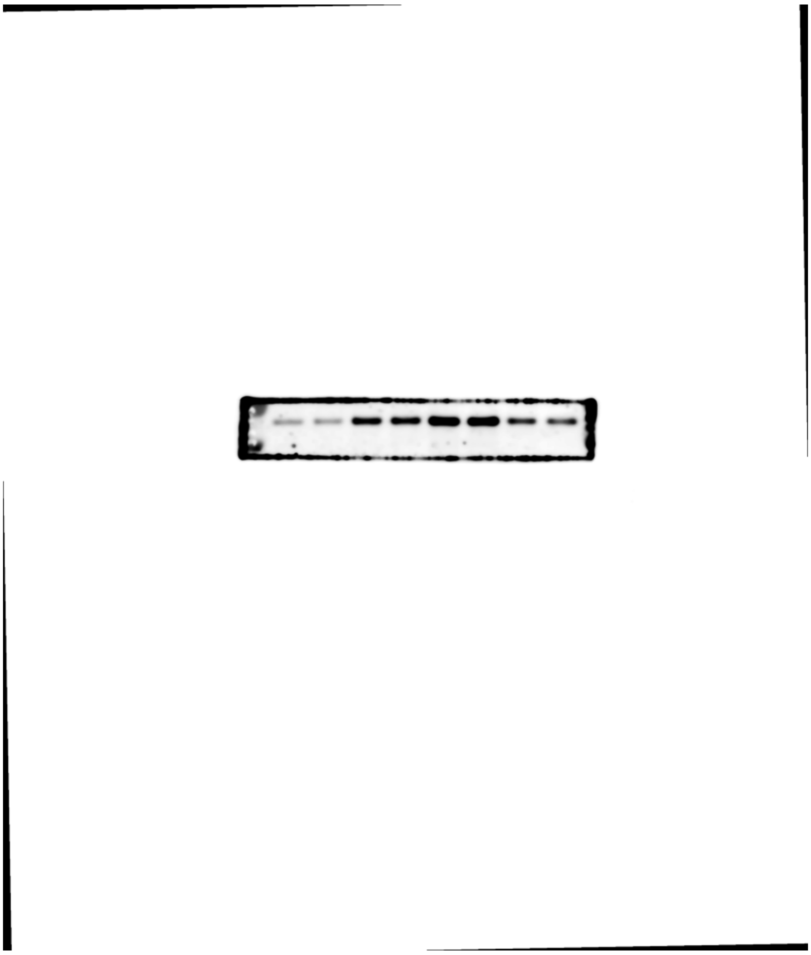

GAPDH (from lane 1 to lane 8)

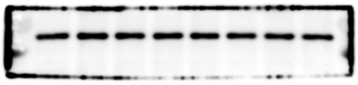

Full unedited gel for Figure 8F(right)

E-Cad (from lane 1 to lane 8)

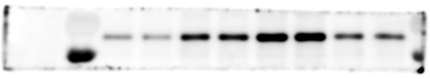

ZO-1 (from lane 1 to lane 8)

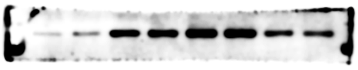

GAPDH (from lane 1 to lane 8)

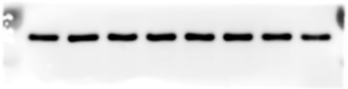

Full unedited gel for Figure 9A(left)

PPFIA4 (from lane 1 to lane 2)

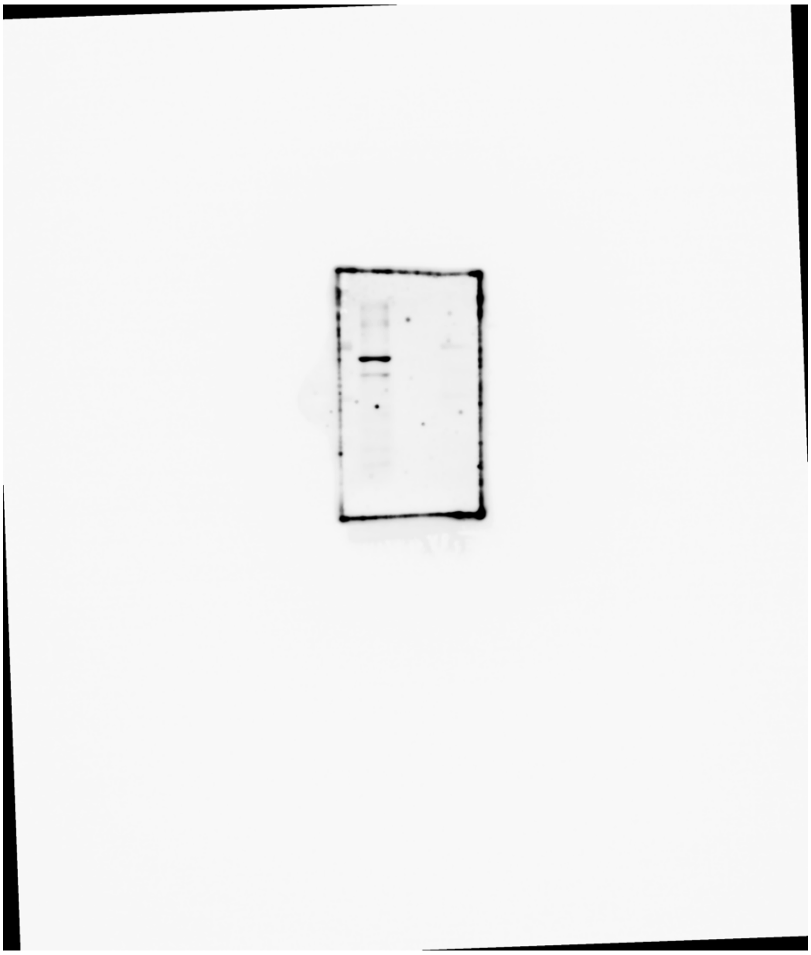

MMP1 (from lane 1 to lane 2)

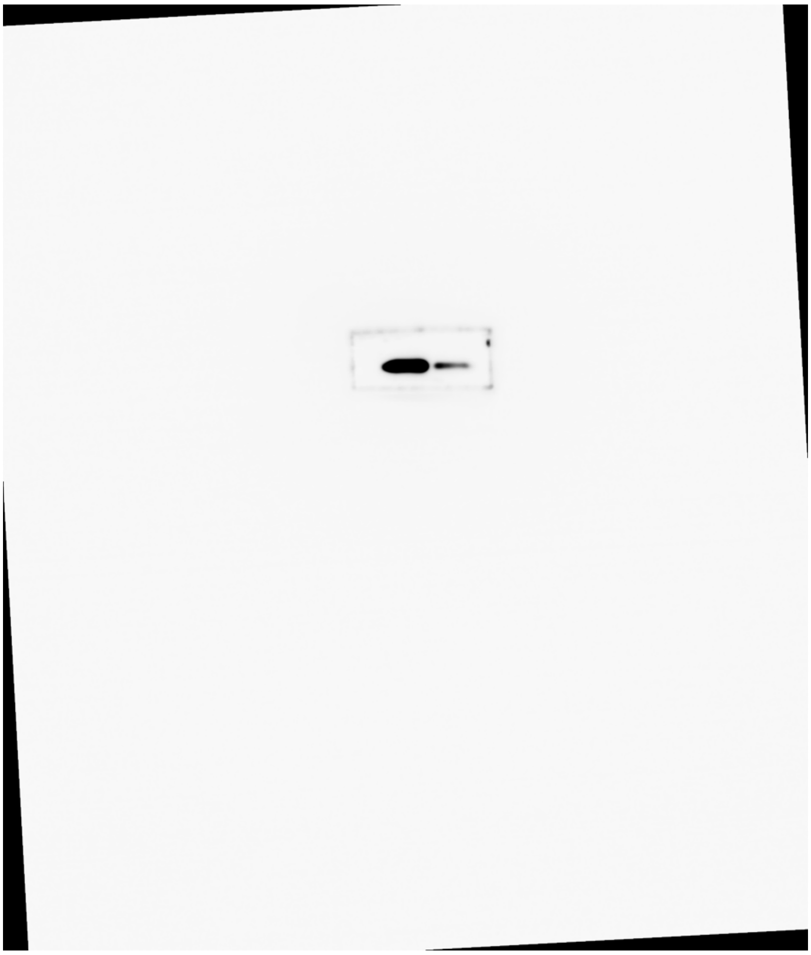

E-Cad (from lane 1 to lane 2)

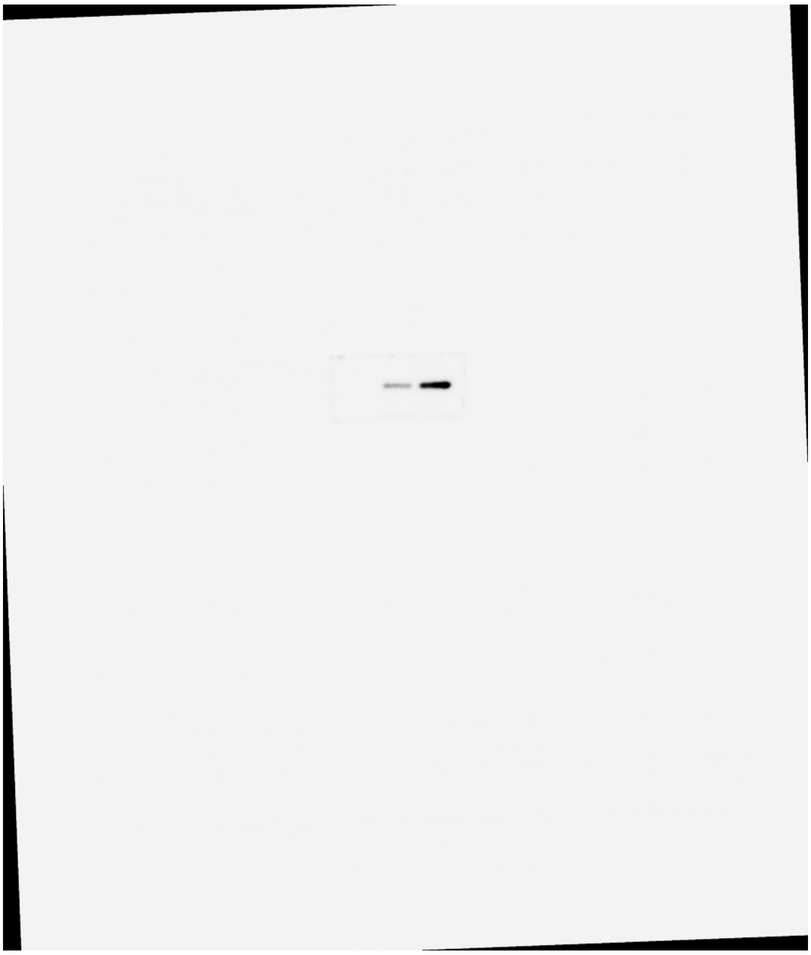

ZO-1 (from lane 1 to lane 2)

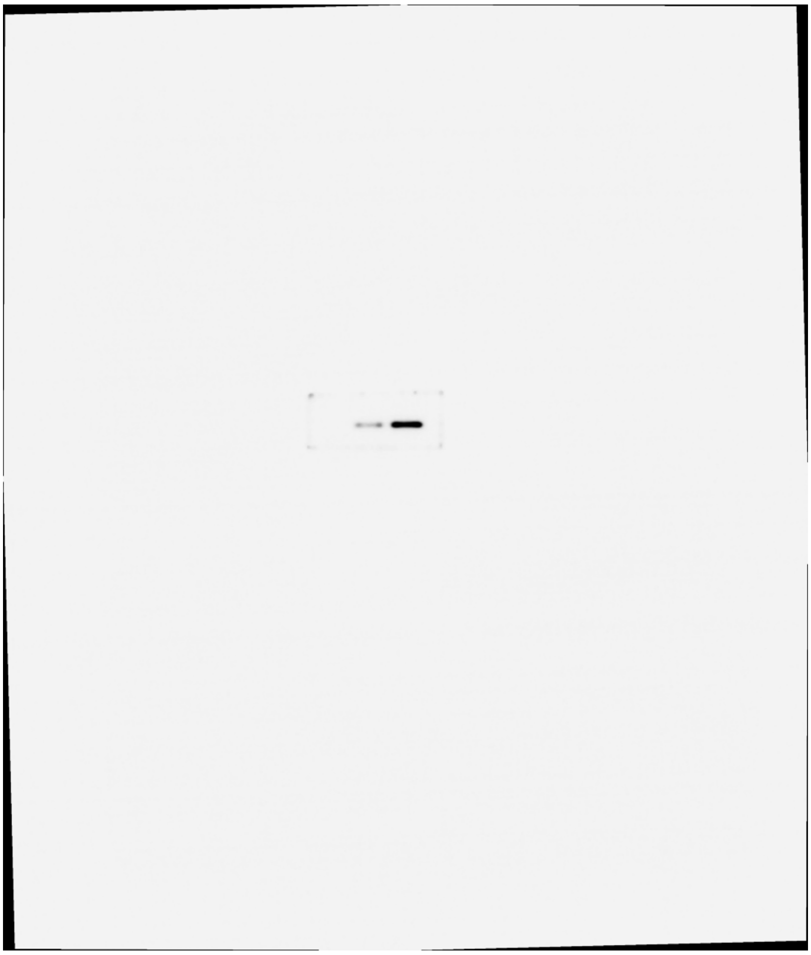

GAPDH (from lane 1 to lane 2)

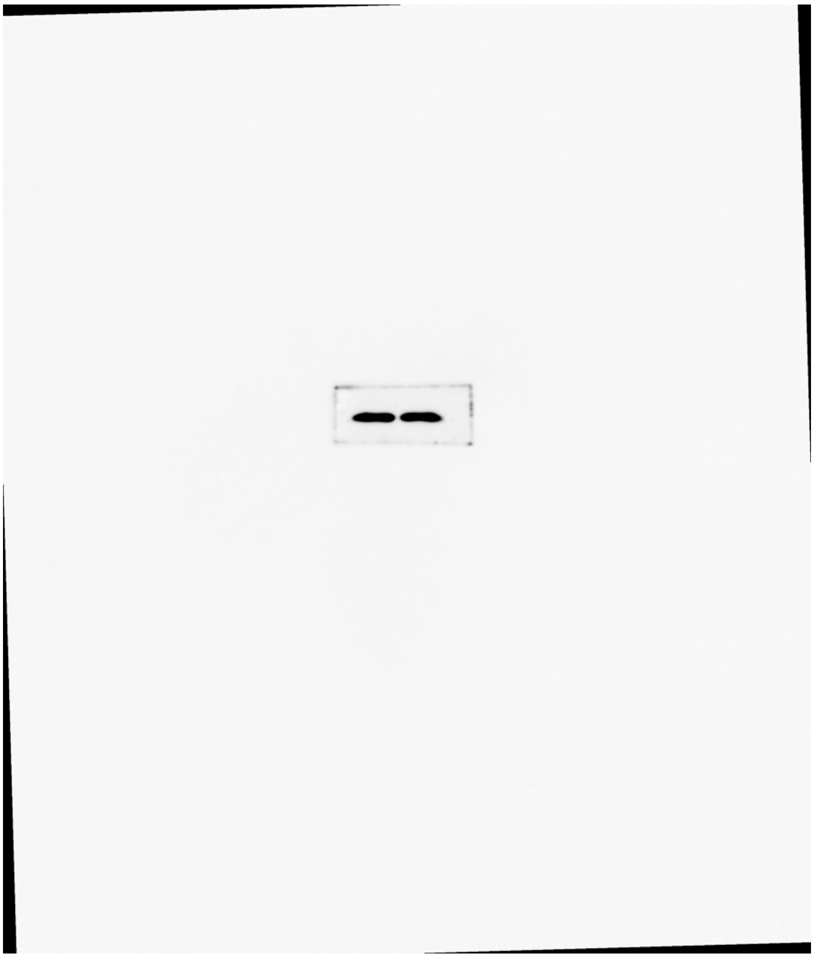

Full unedited gel for Figure 9A(right)

PPFIA4 (from lane 1 to lane 2)

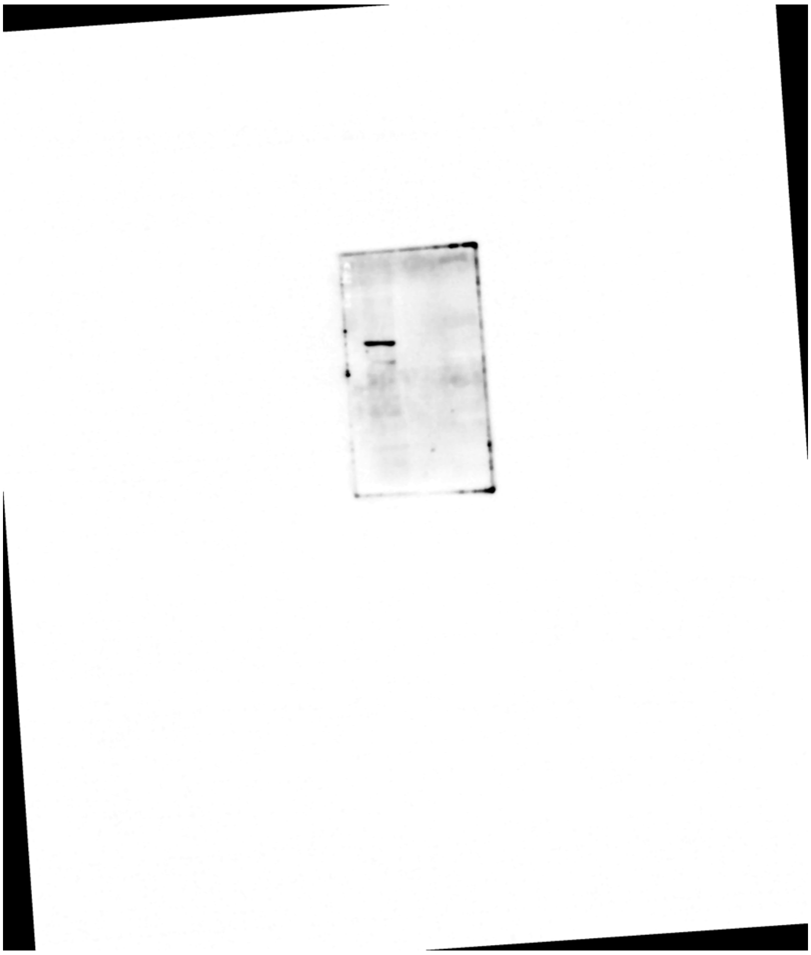

MMP1 (from lane 1 to lane 2)

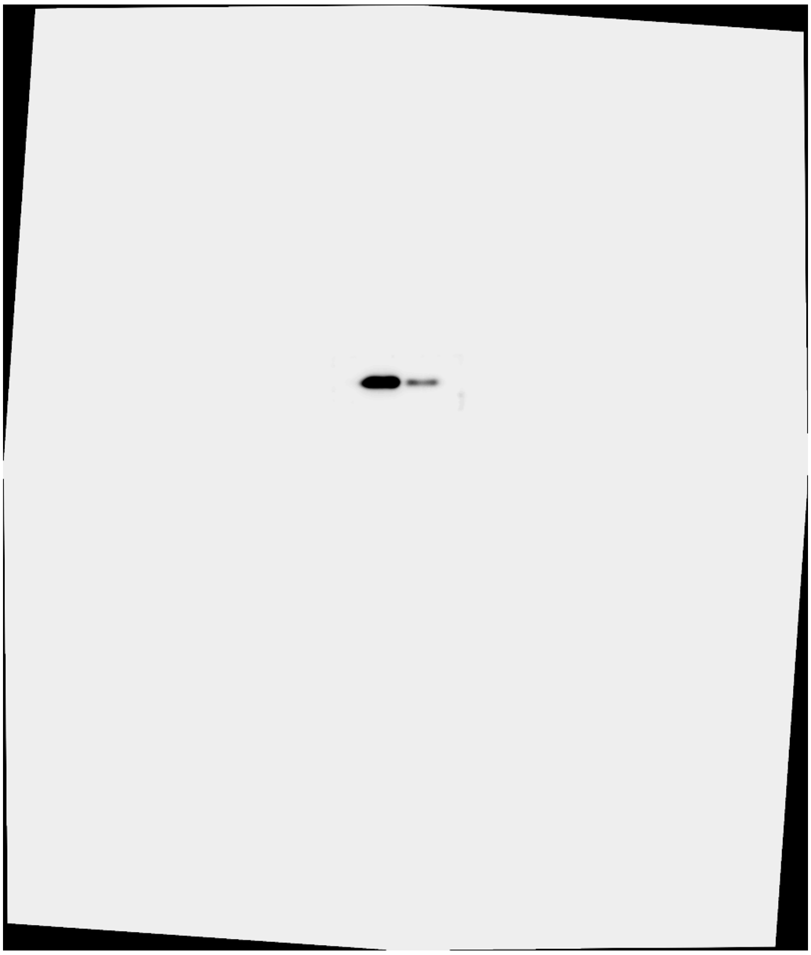

E-Cad (from lane 1 to lane 2)

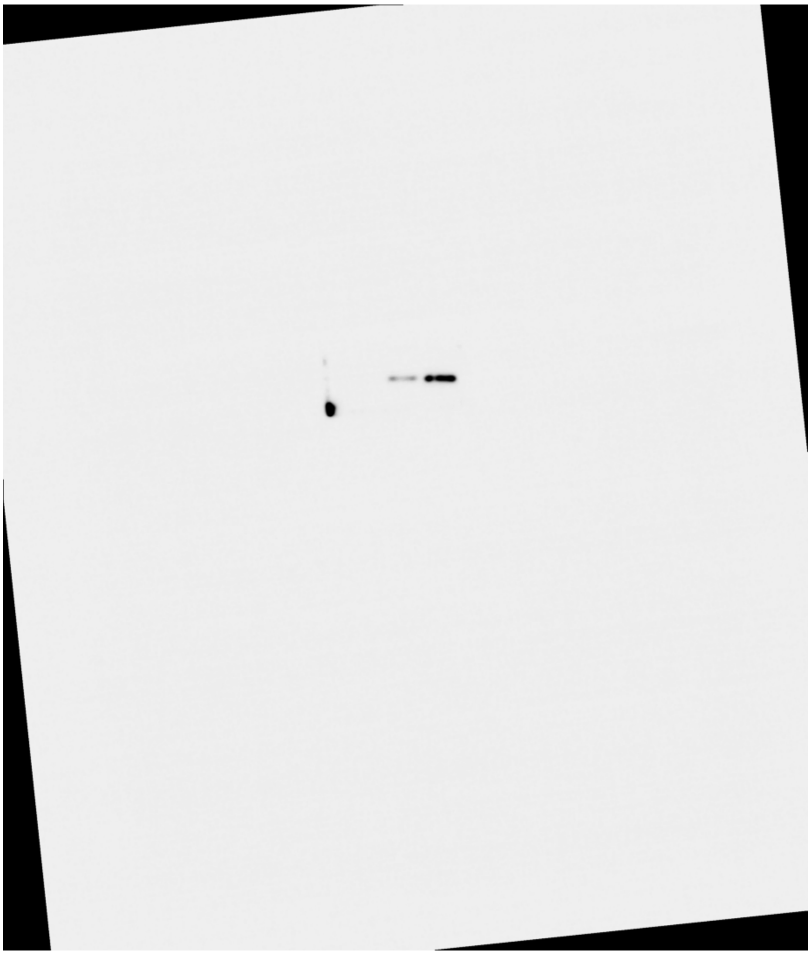

ZO-1 (from lane 1 to lane 2)

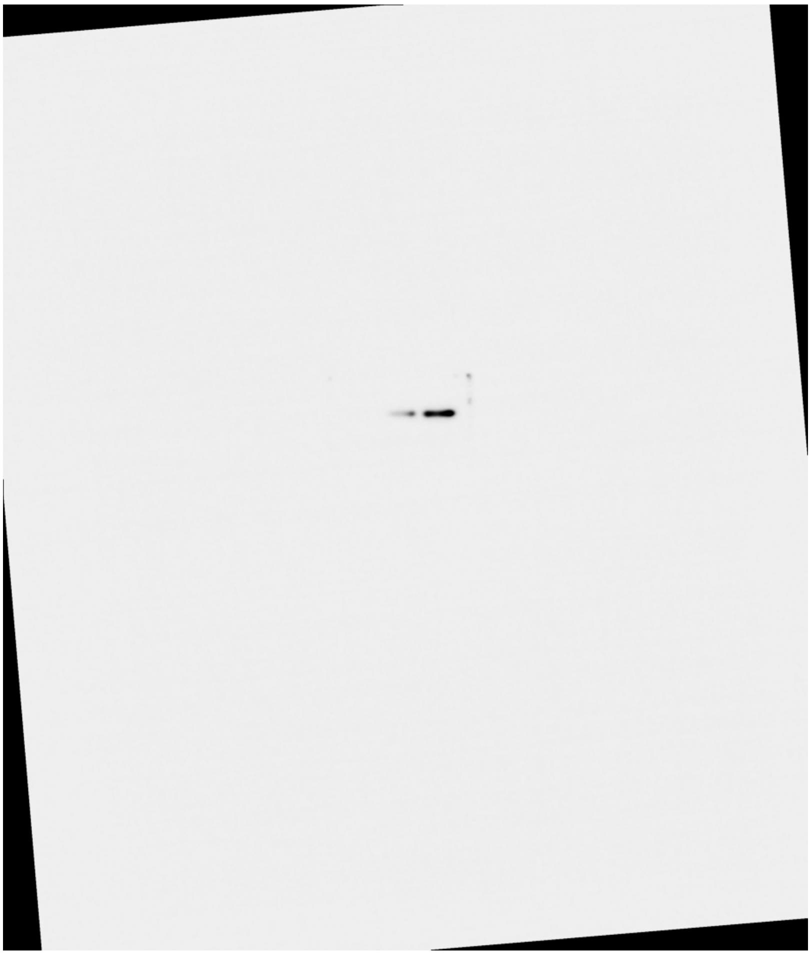

GAPDH (from lane 1 to lane 2)

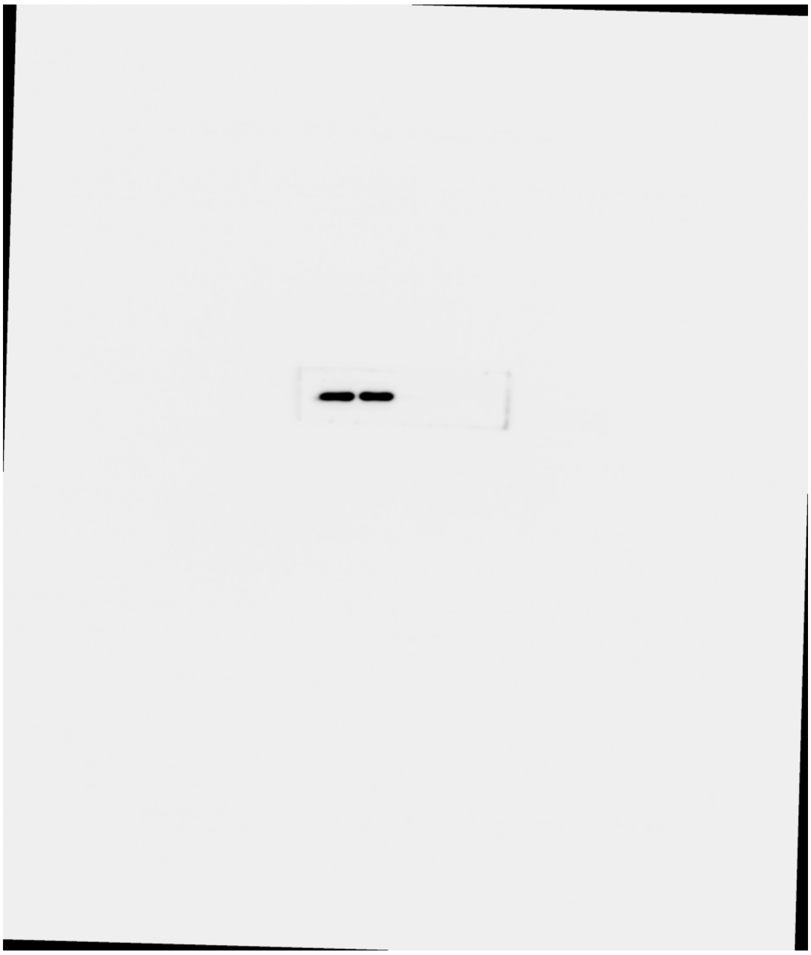

Full unedited gel for Figure 9B

E-Cad (from lane 1 to lane 4)

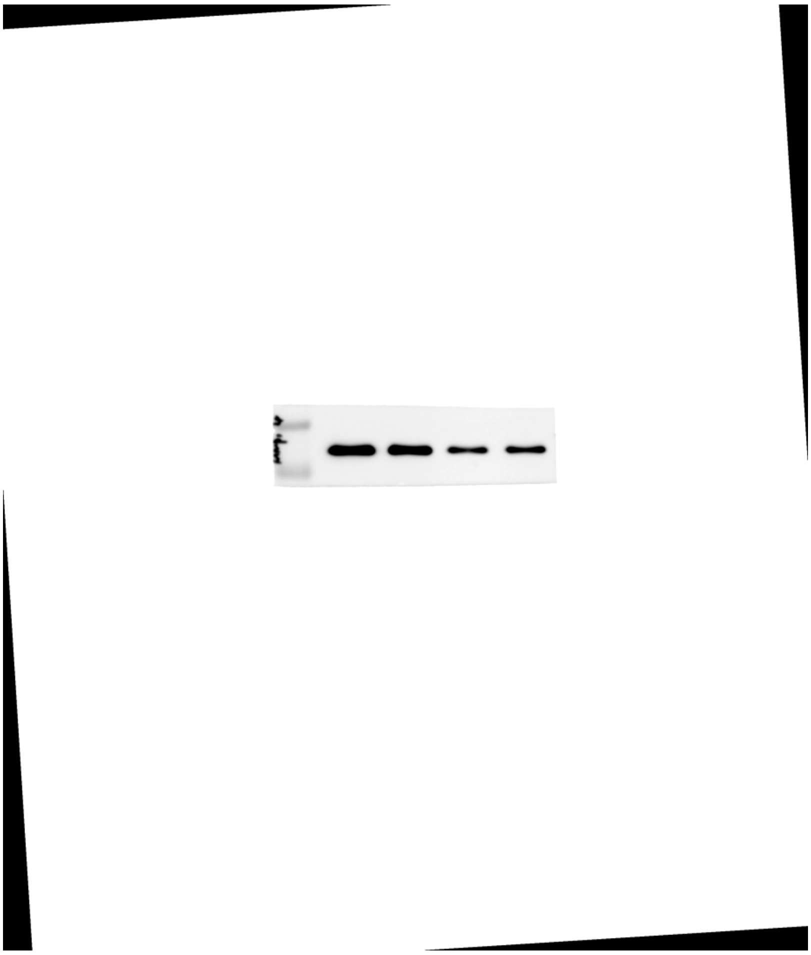

ZO-1 (from lane 1 to lane 4)

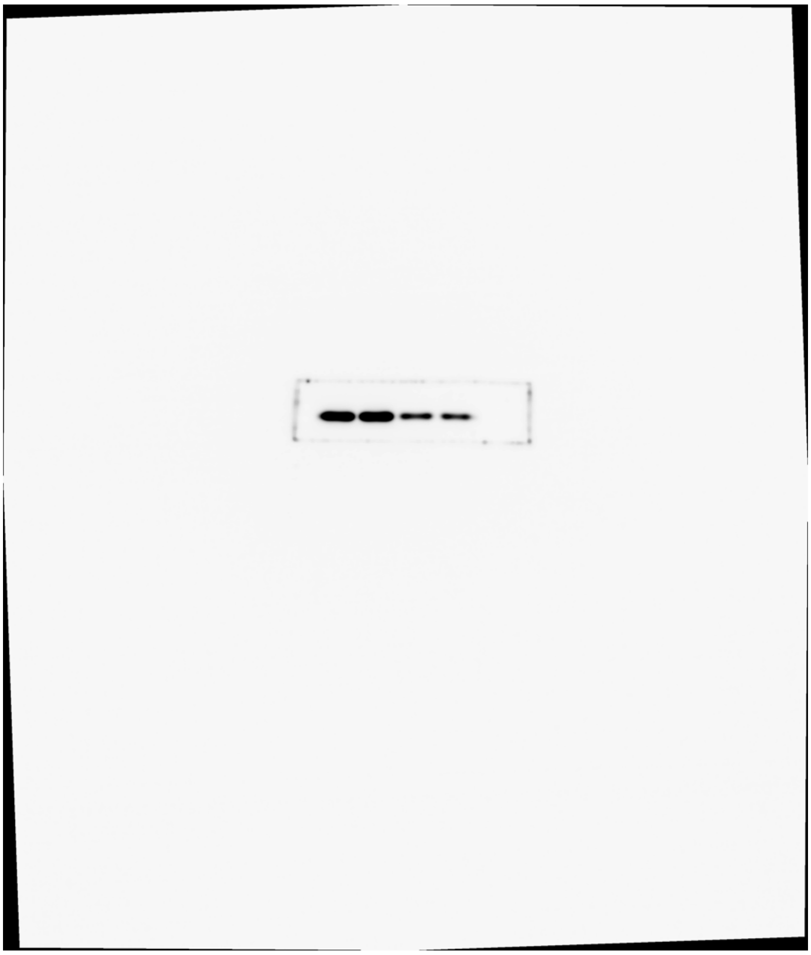

GAPDH (from lane 1 to lane 4)

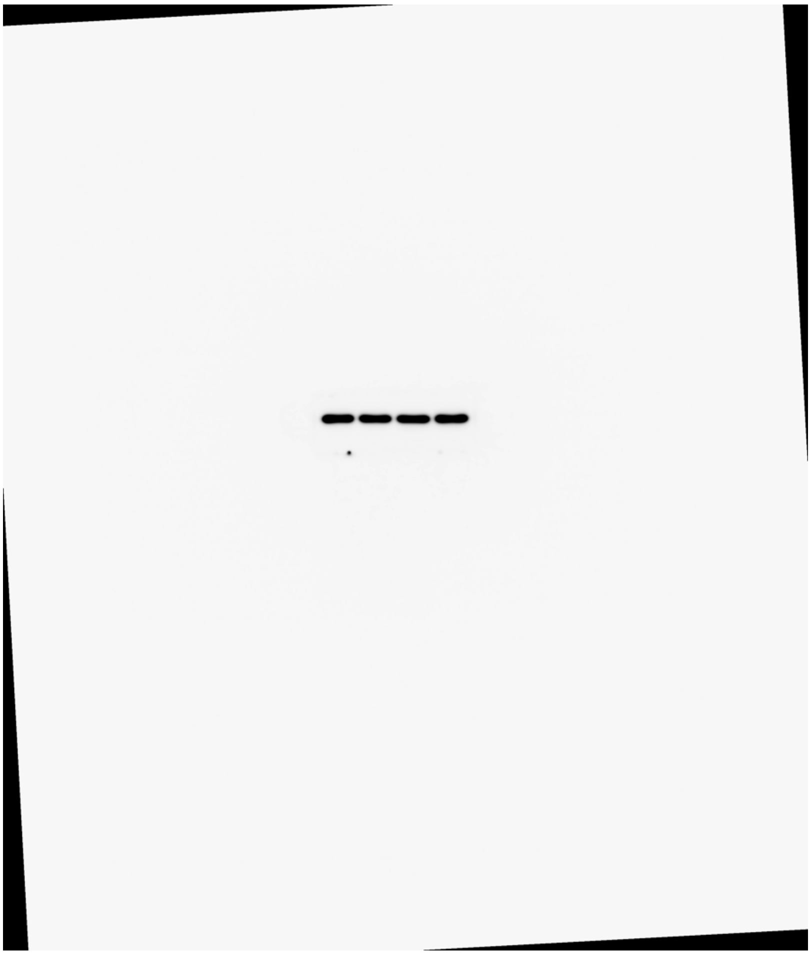

Full unedited gel for Figure 9D

PPFIA4 (from lane 1 to lane 8)

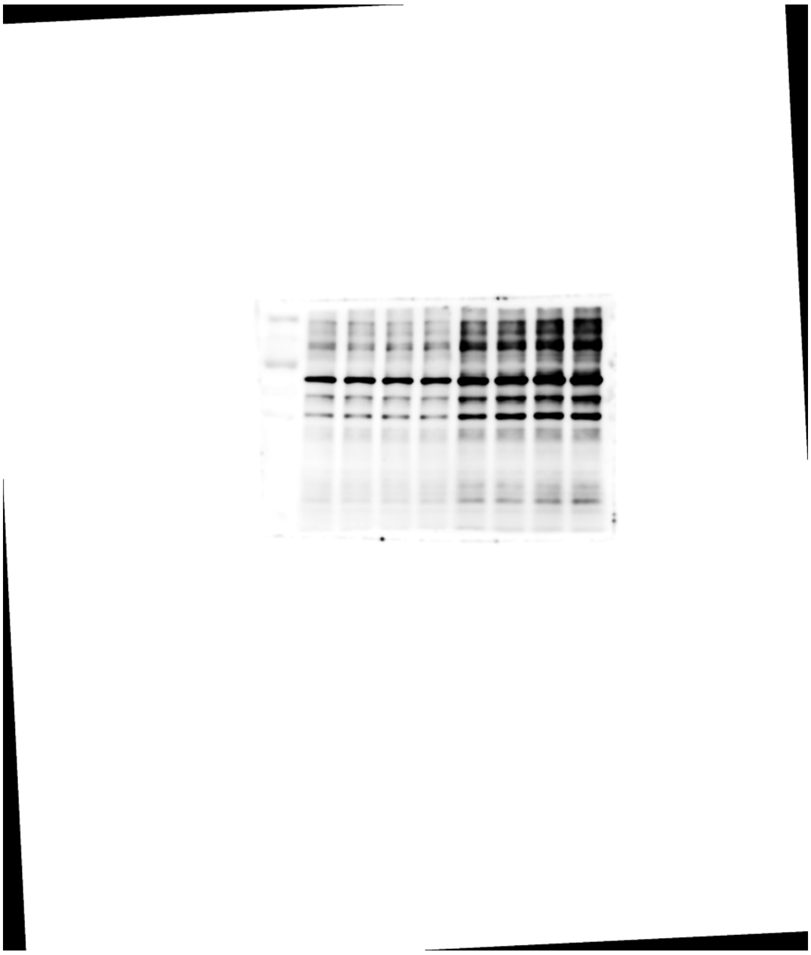

MMP1 (from lane 1 to lane 8)

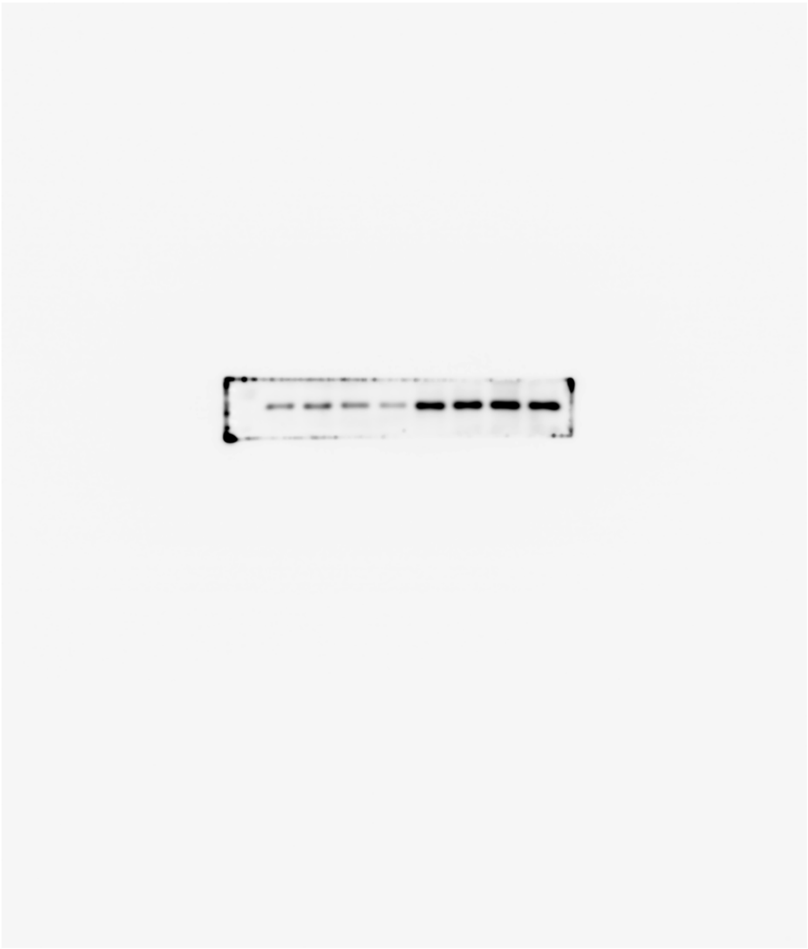

E-Cad (from lane 1 to lane 8)

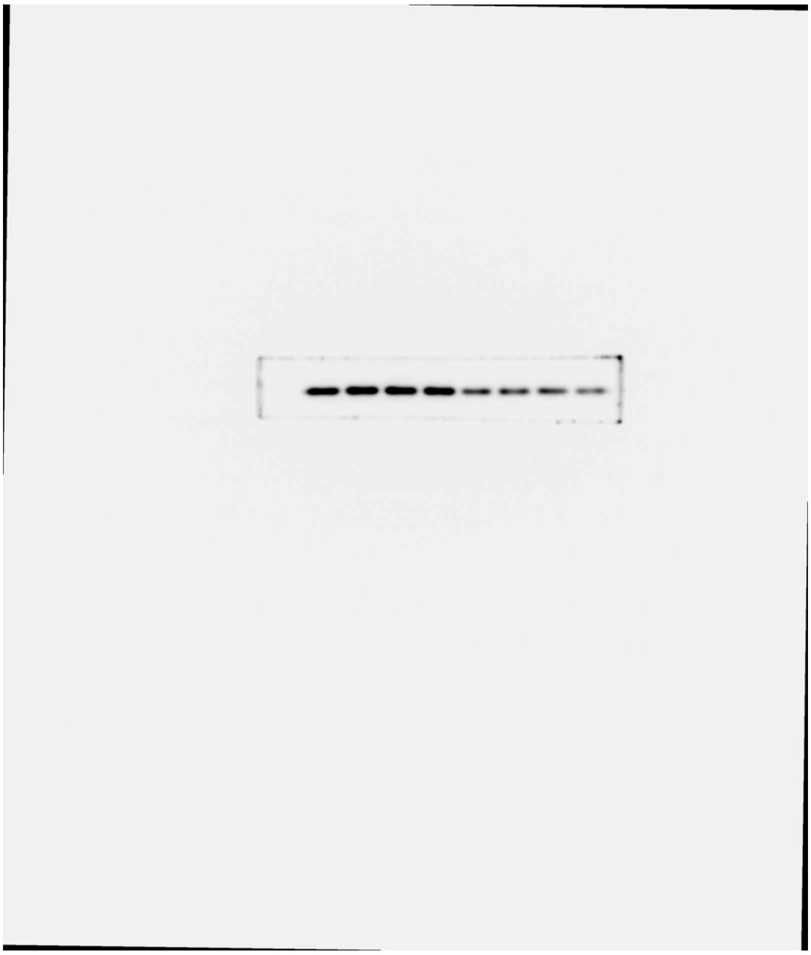

ZO-1 (from lane 1 to lane 8)

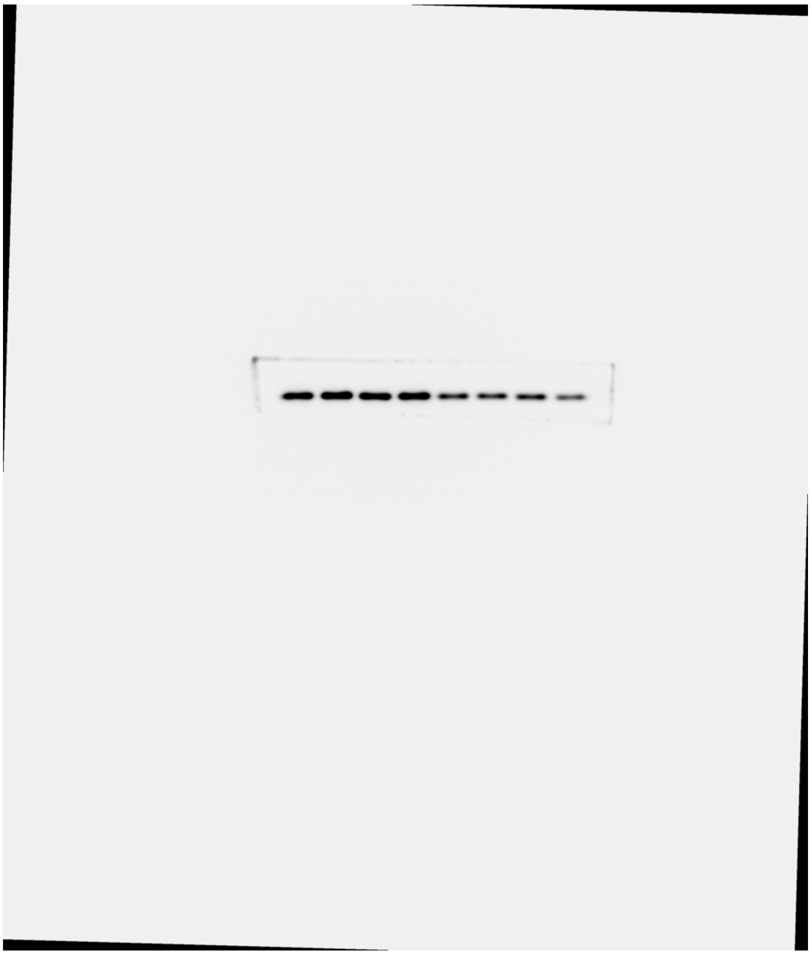

GAPDH (from lane 1 to lane 8)

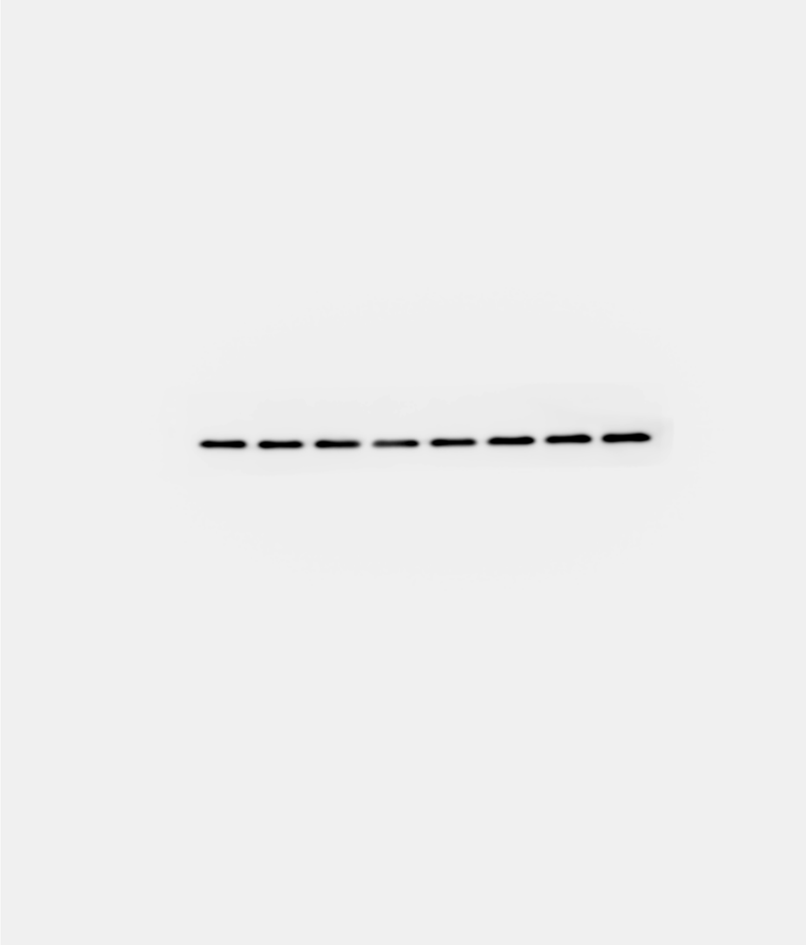

Full unedited gel for Figure 10B(left)
IB: CASK (from lane 3 to lane 4)

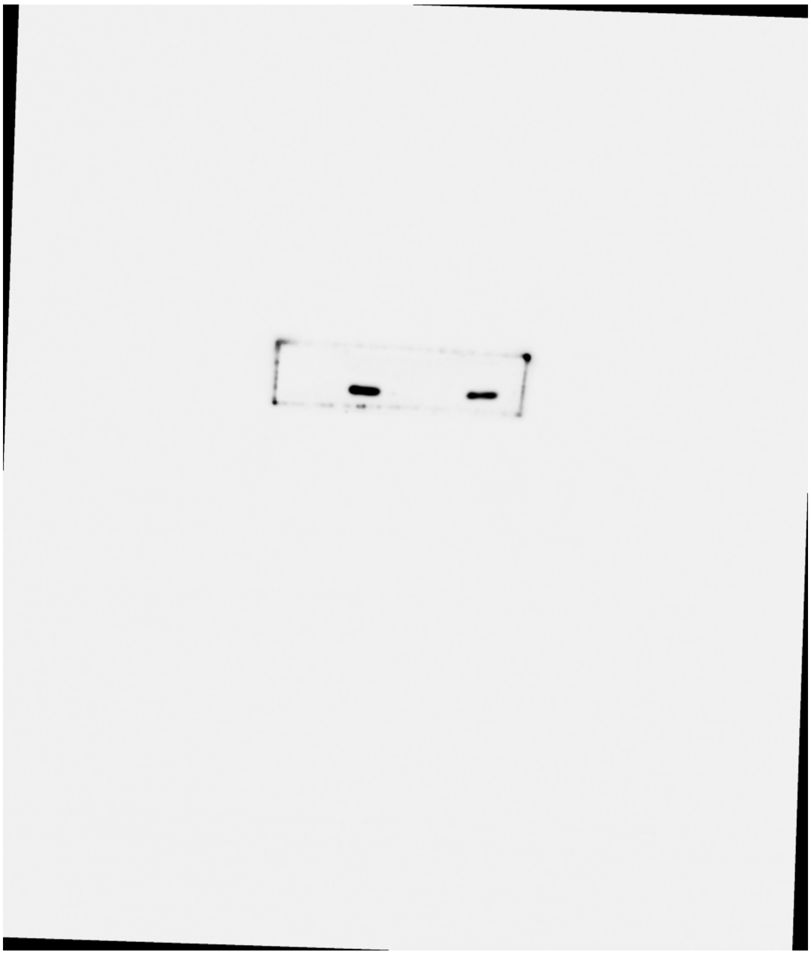

IB: PPFIA4-Flag (from lane 1 to lane 2)

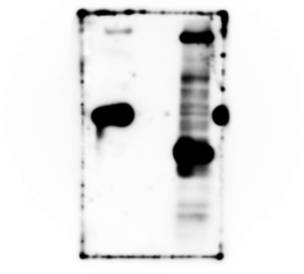

Input: CASK (from lane 3 to lane 4)

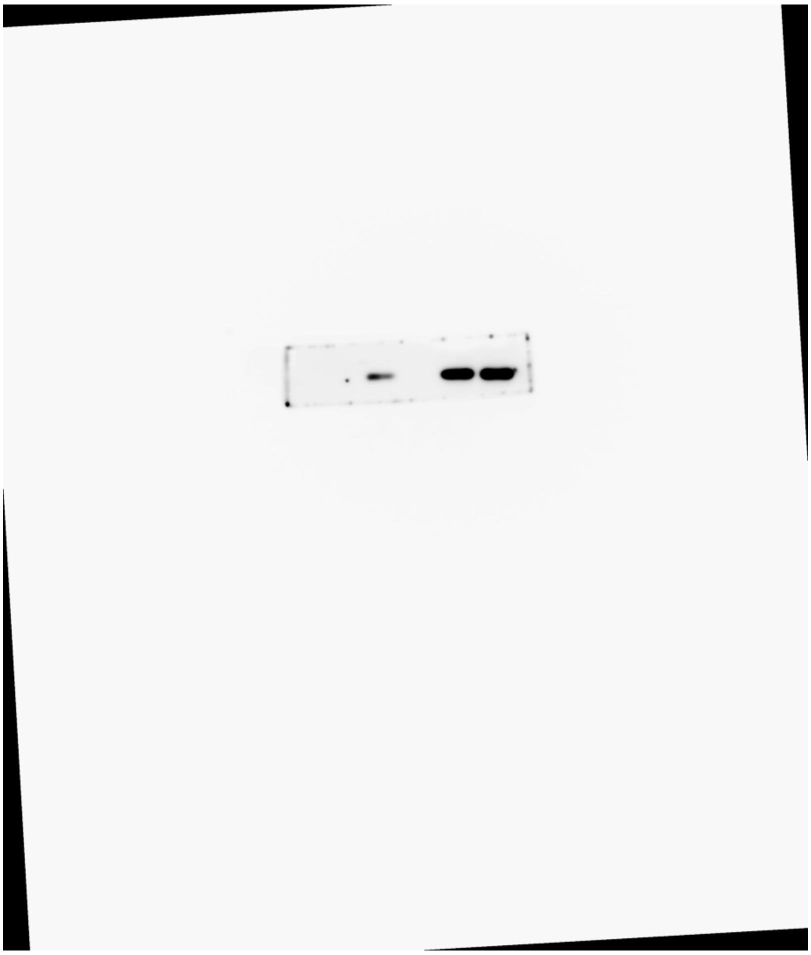

Input: PPFIA4-Flag (from lane 1 to lane 2)

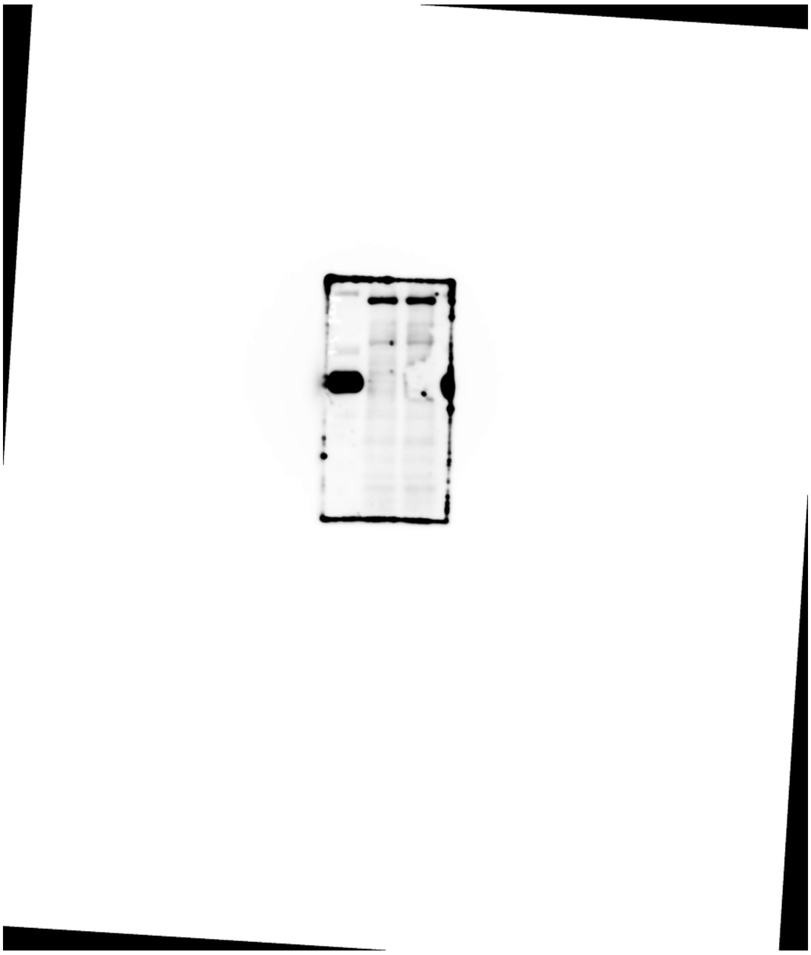

Full unedited gel for Figure 10B(right)

IB: PPFIA4-Flag (from lane 1 to lane 2)

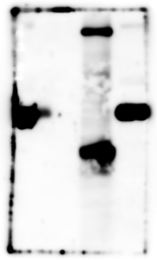

IB: CASK (from lane 1 to lane 2)

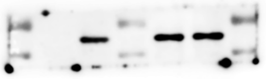

Input: PPFIA4-Flag (from lane 1 to lane 2)

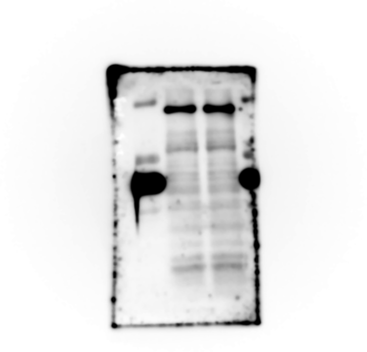

Input: CASK (from lane 3 to lane 4)

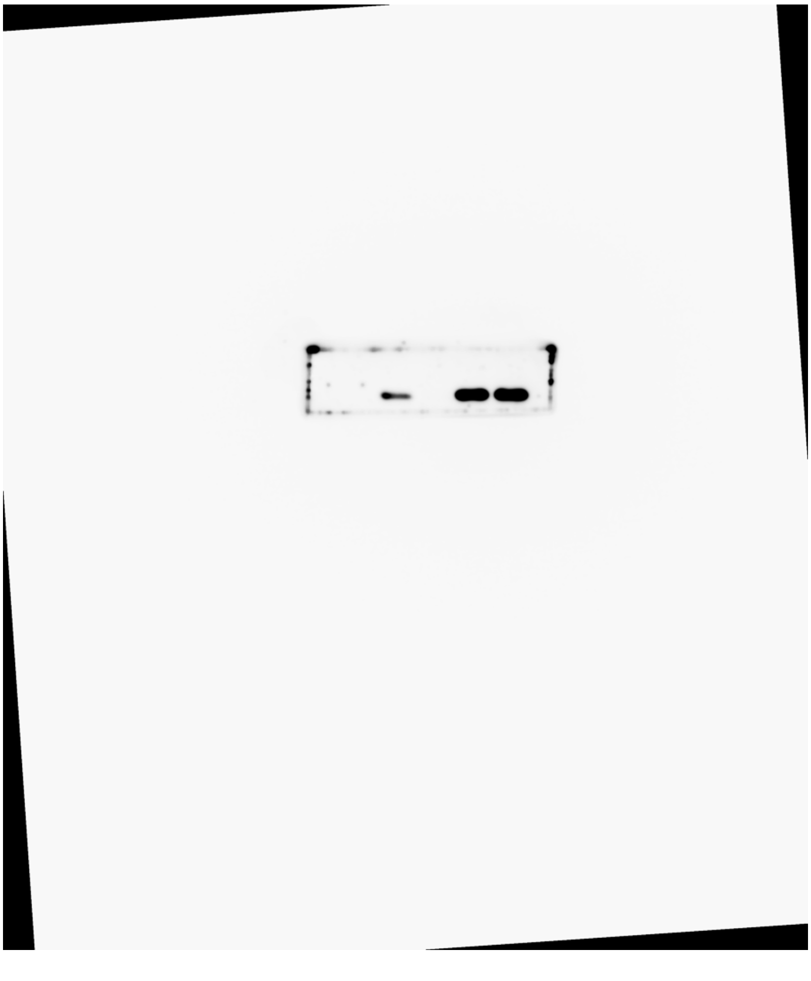

Full unedited gel for Figure 10E(up)
CASK (from lane 1 to lane 7)

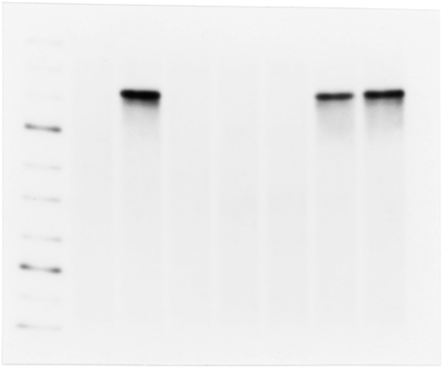

Flag (from lane 1 to lane 7)

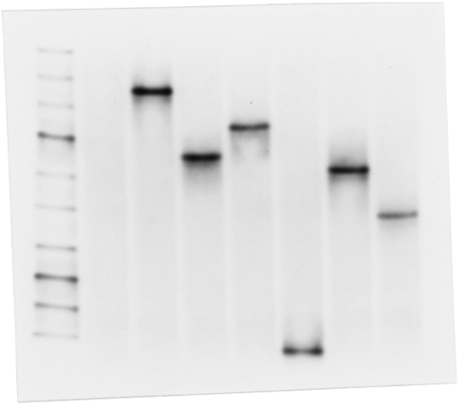

Full unedited gel for Figure 10E(down)
CASK (from lane 1 to lane 7)

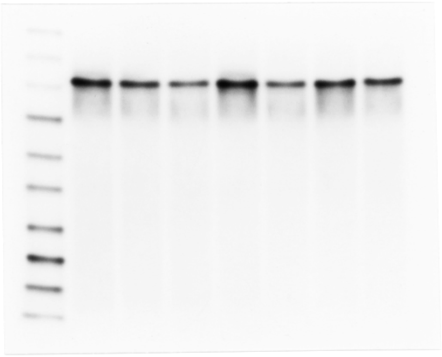

Flag (from lane 1 to lane 7)

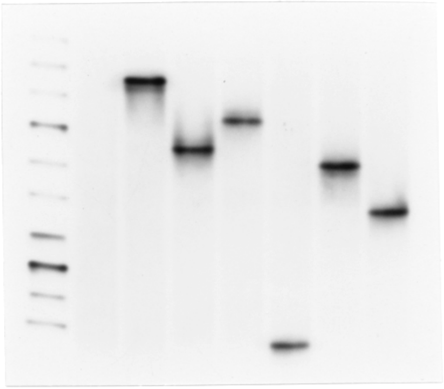

Full unedited gel for Figure 10F(up)
CASK (from lane 1 to lane 7)

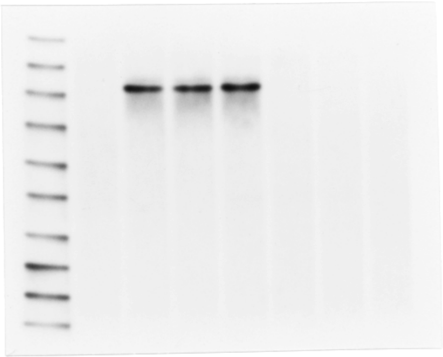

Flag (from lane 1 to lane 7)

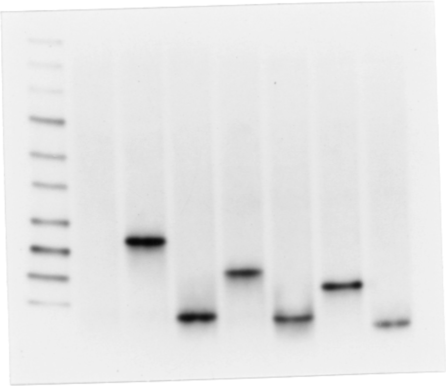

Full unedited gel for Figure 10F(down)
CASK (from lane 1 to lane 7)

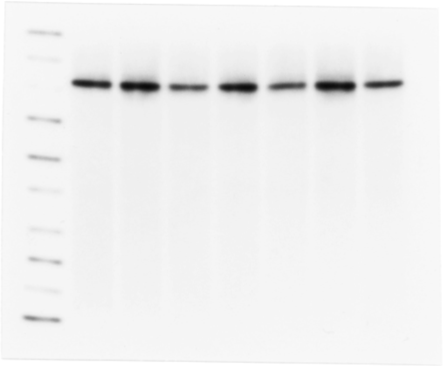

Flag (from lane 1 to lane 7)

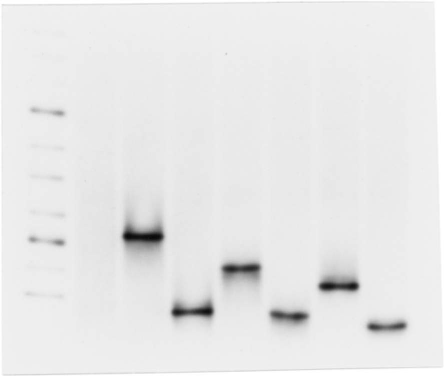

Full unedited gel for Figure 11A (left up)
PPFIA4 (from lane 1 to lane 7)

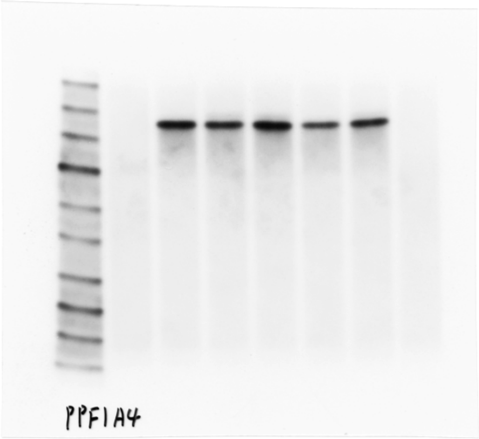


HA (from lane 1 to lane 7)

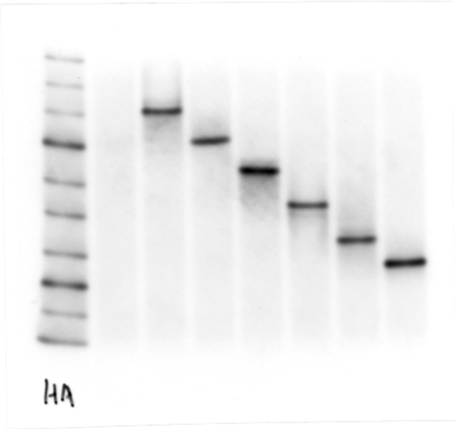

Full unedited gel for Figure 11A(left down)
PPFIA4 (from lane 1 to lane 7)

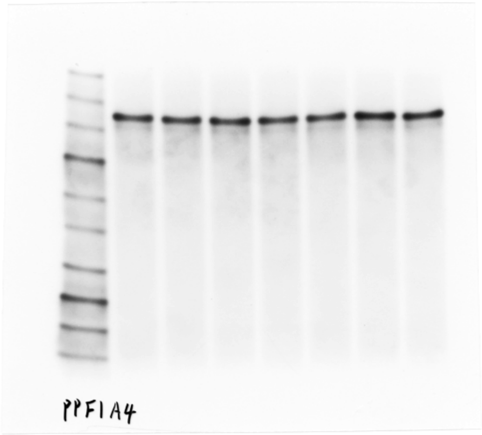


HA (from lane 1 to lane 7)

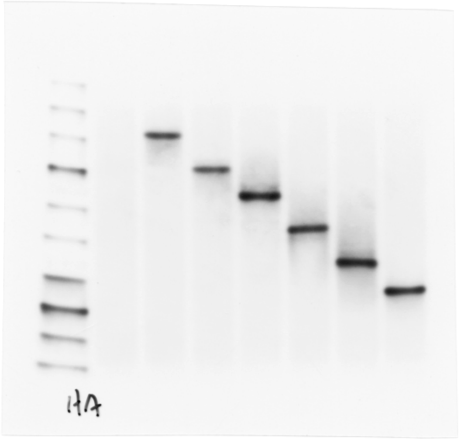

Full unedited gel for Figure 11A(right up)
PPFIA4 (from lane 1 to lane 7)

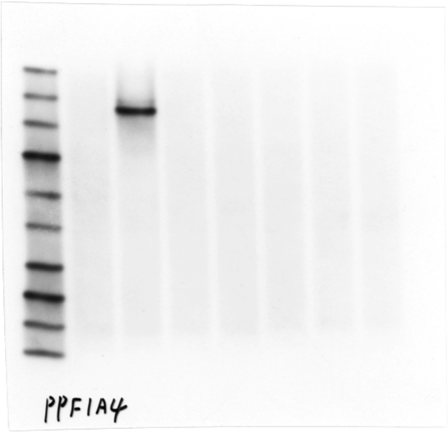


HA (from lane 1 to lane 7)

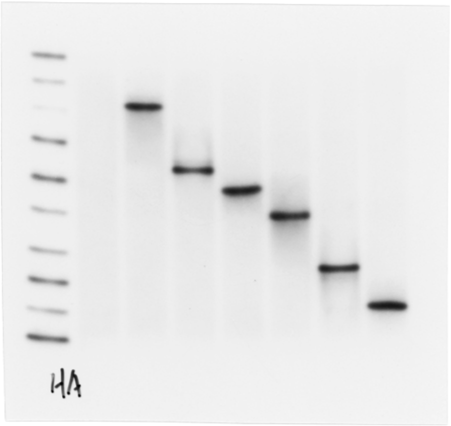

Full unedited gel for Figure 11A(right down)
PPFIA4 (from lane 1 to lane 7)

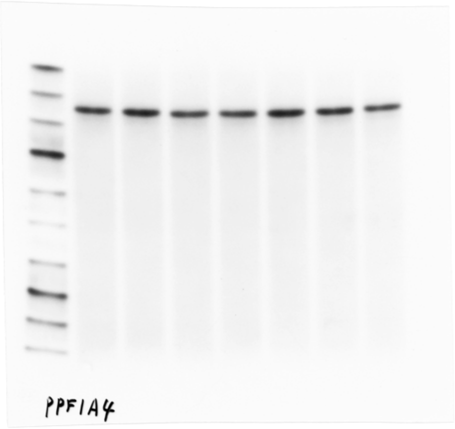


HA (from lane 1 to lane 7)

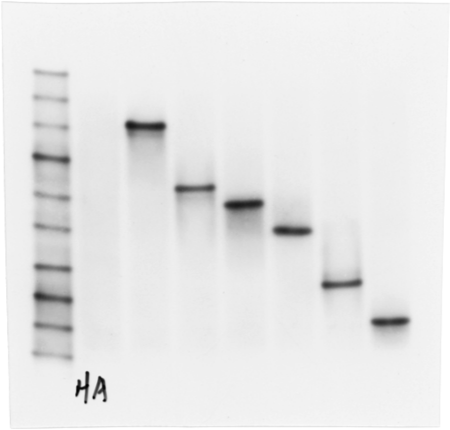

Full unedited gel for Figure 11B(left)

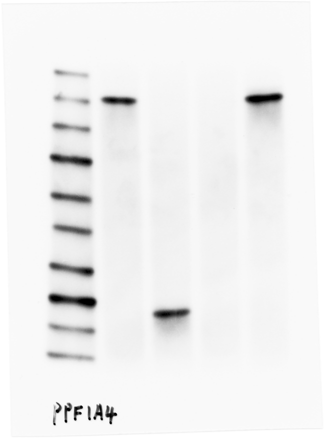

Full unedited gel for Figure 11B(right)

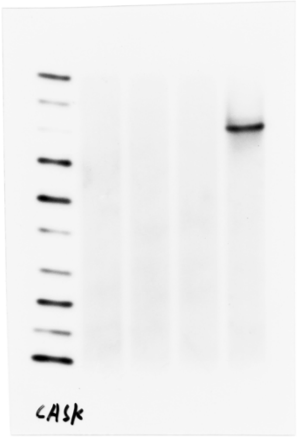

Full unedited gel for Figure 12C(left up)

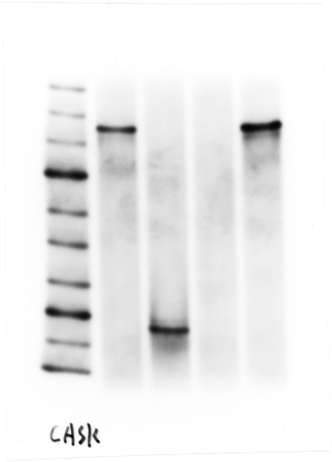

Full unedited gel for Figure 12C(left down)

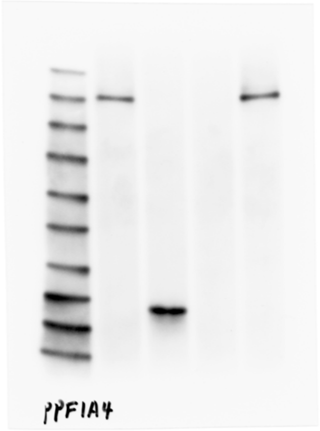

Full unedited gel for Figure 12C(right up)

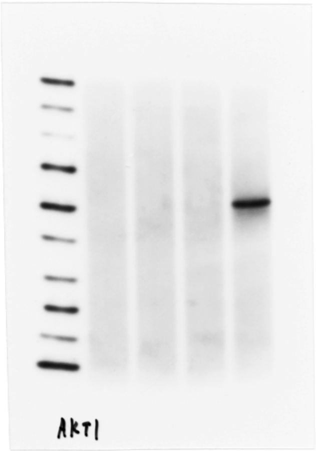

Full unedited gel for Figure 12C(right down)

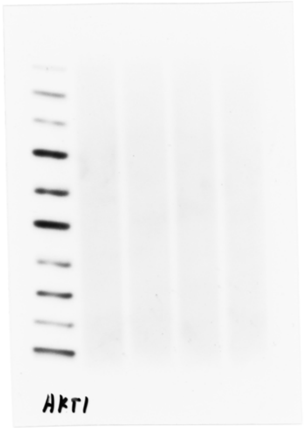

Full unedited gel for Figure 12D(left)

IB: AKT1 (from lane 3 to lane 4)

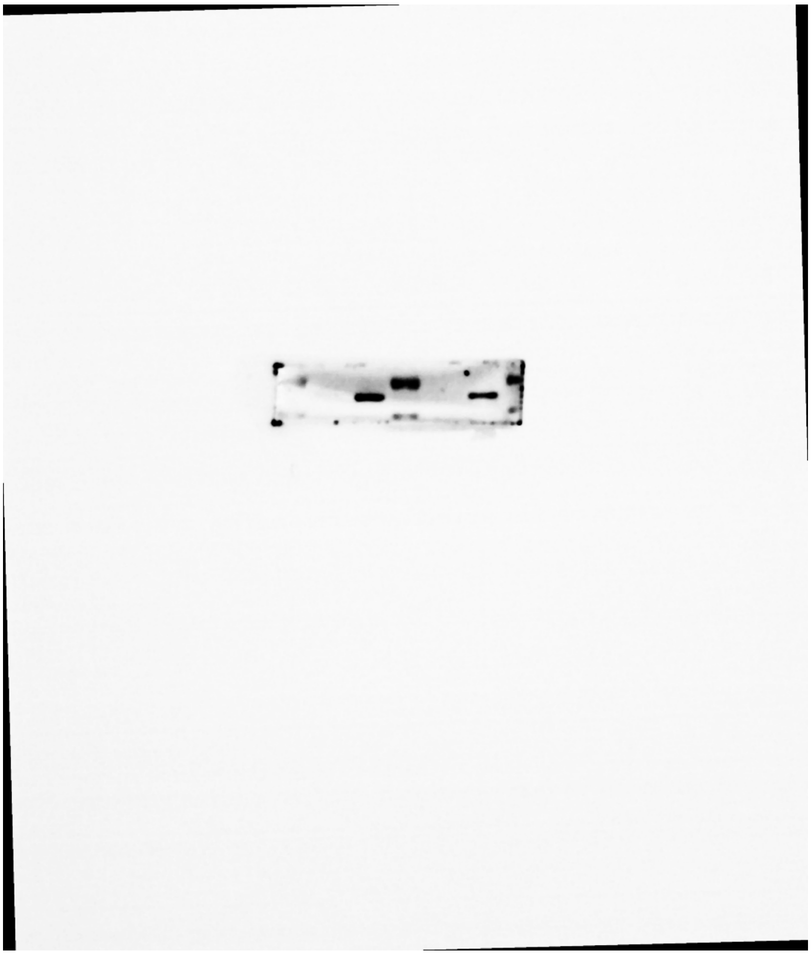

IB: PPFIA4-Flag (from lane 1 to lane 2)

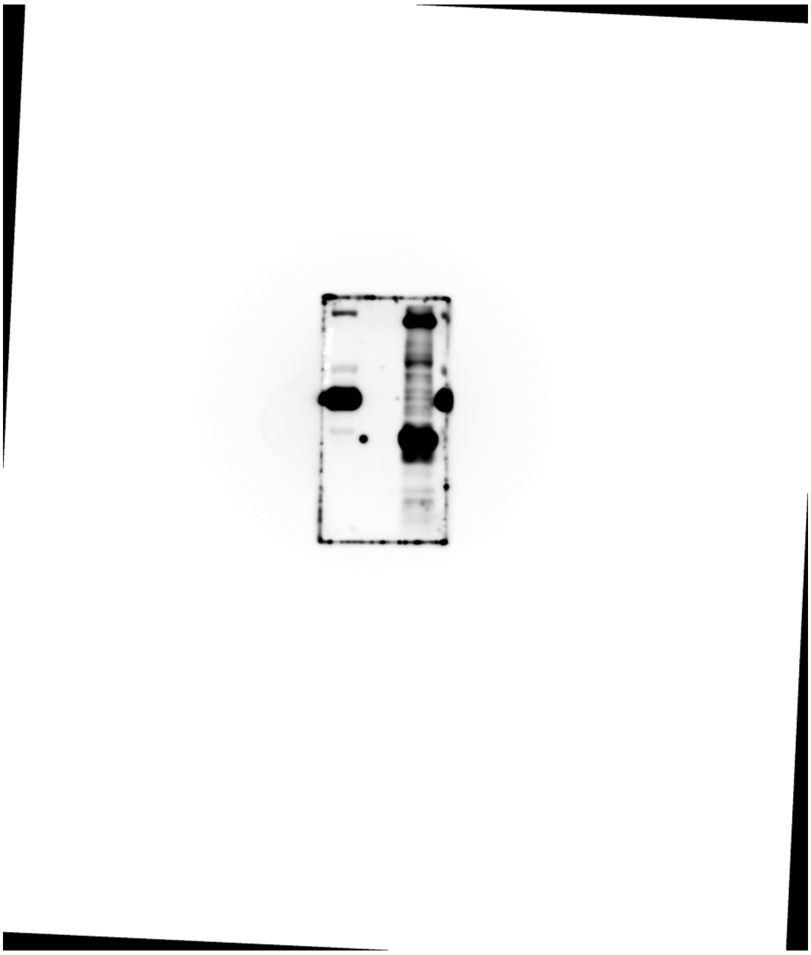

Input: AKT1 (from lane 3 to lane 4)

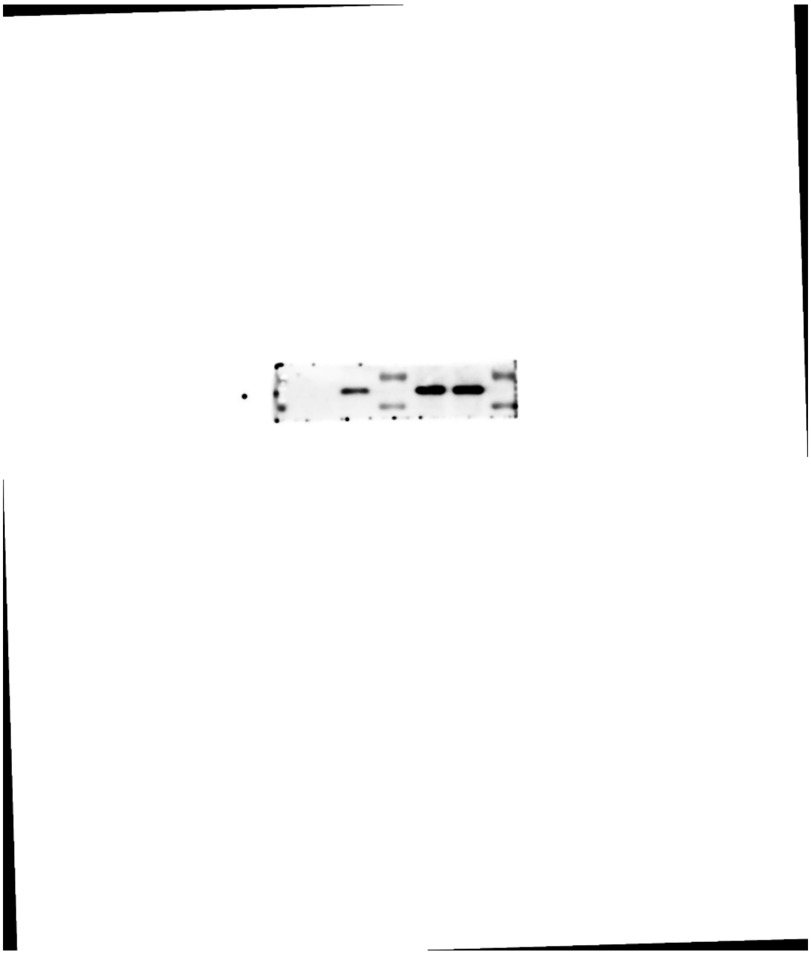

Input: PPFIA4-Flag (from lane 1 to lane 2)

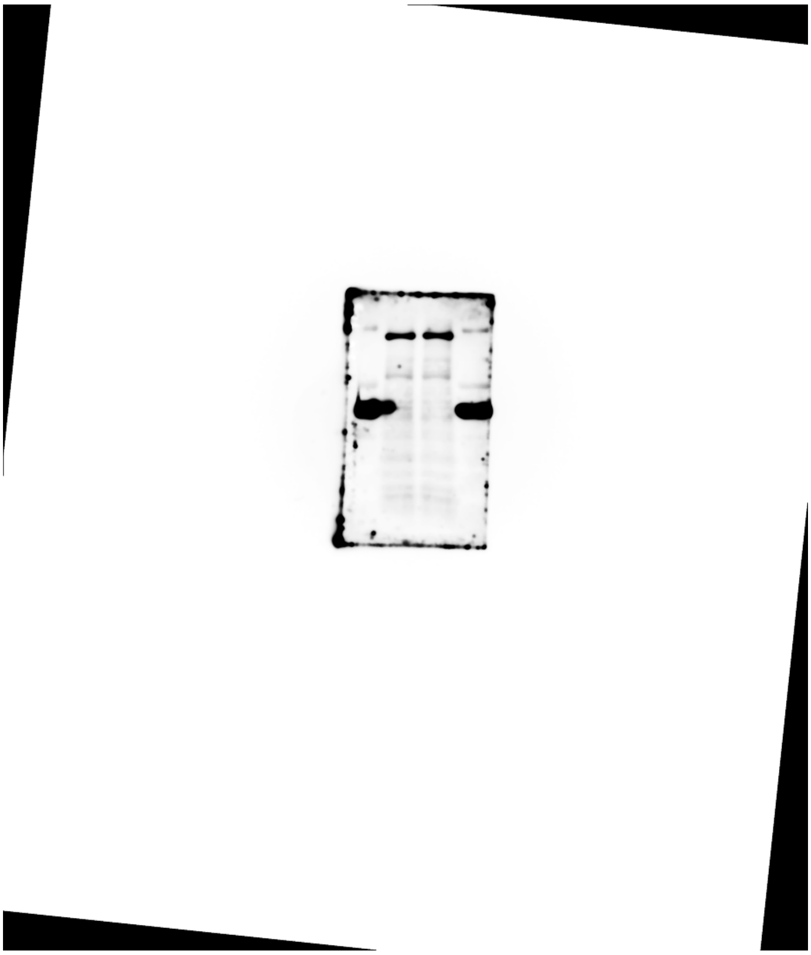

Full unedited gel for Figure 12D(right)

IB: AKT1 (from lane 3 to lane 4)

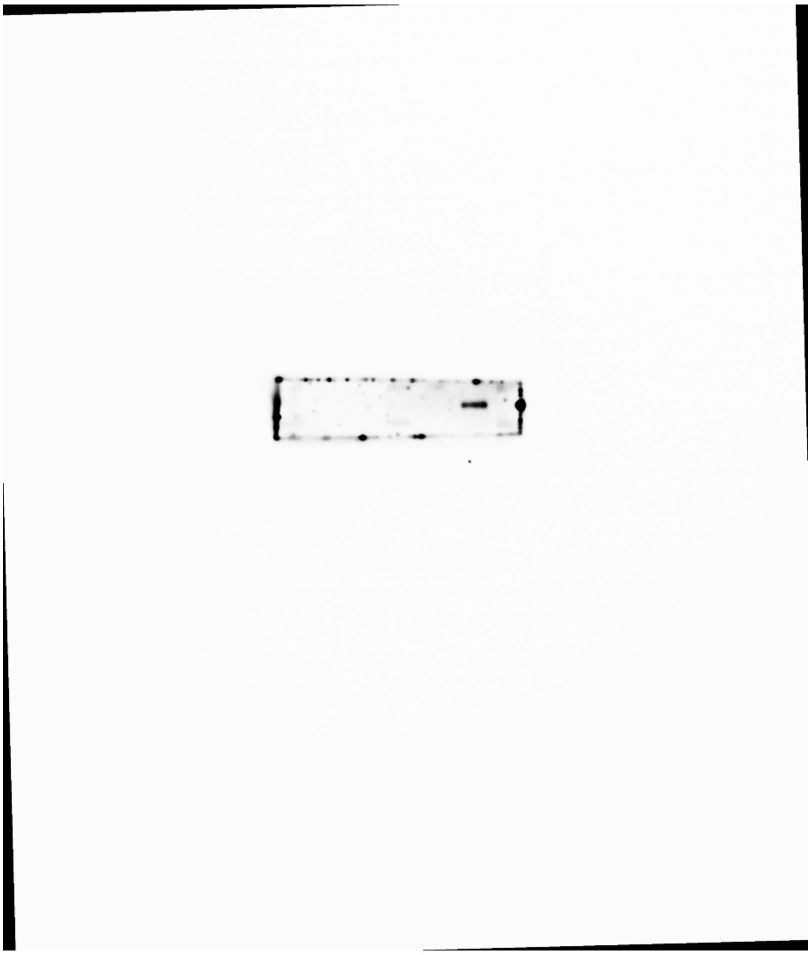

IB: CASK (from lane 1 to lane 2)

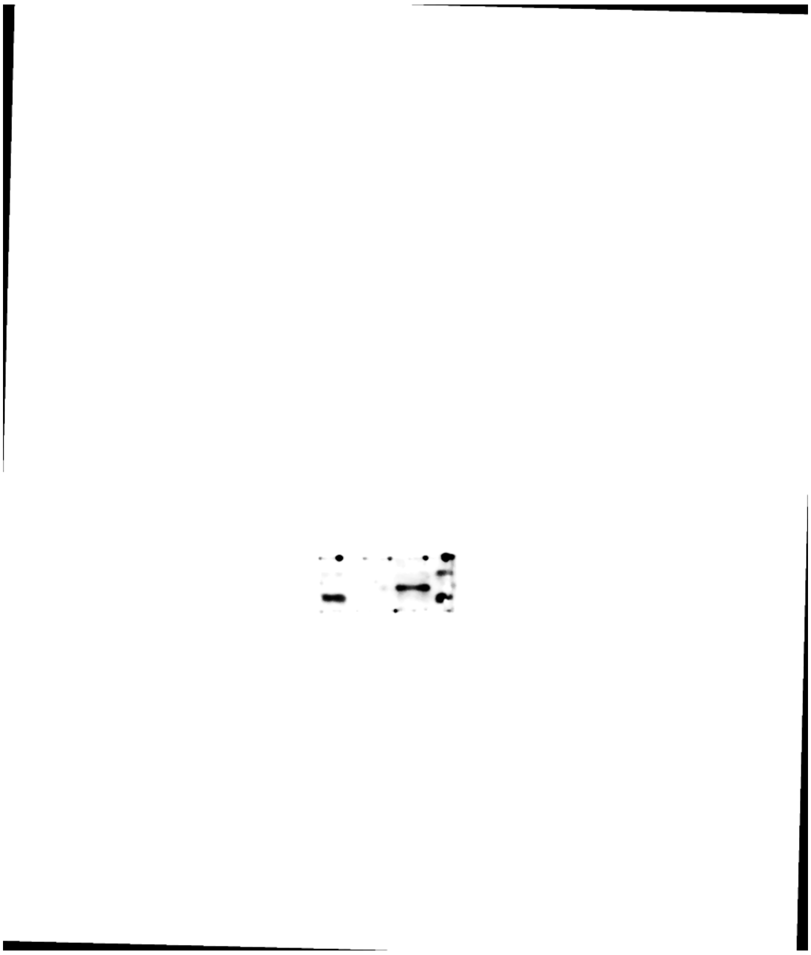

Input: AKT1 (from lane 3 to lane 4)

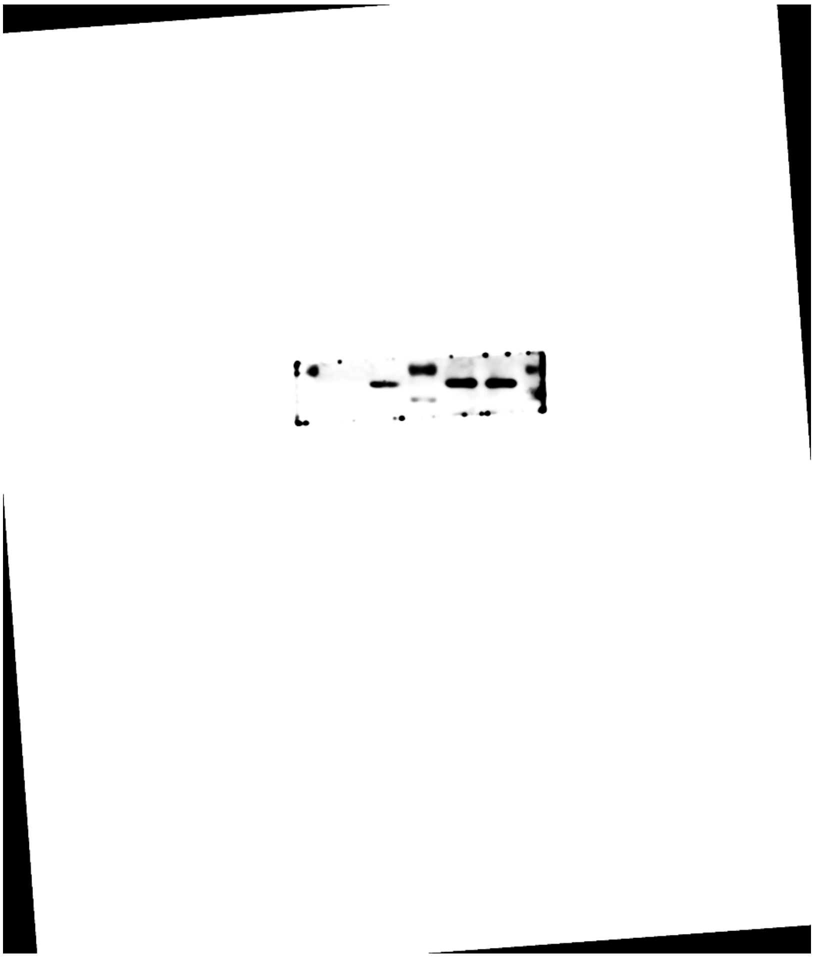

Input: CASK (from lane 1 to lane 2)

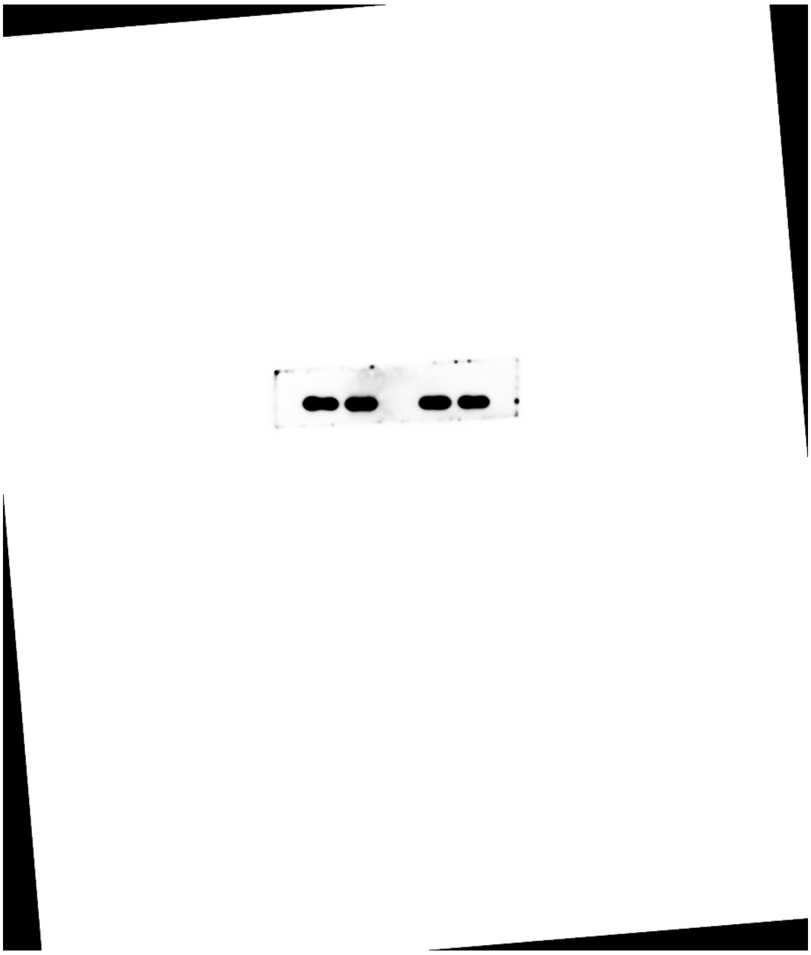

Full unedited gel for Figure 12G(left)

IB: p-AKT1(Thr308) (from lane 1 to lane 2)

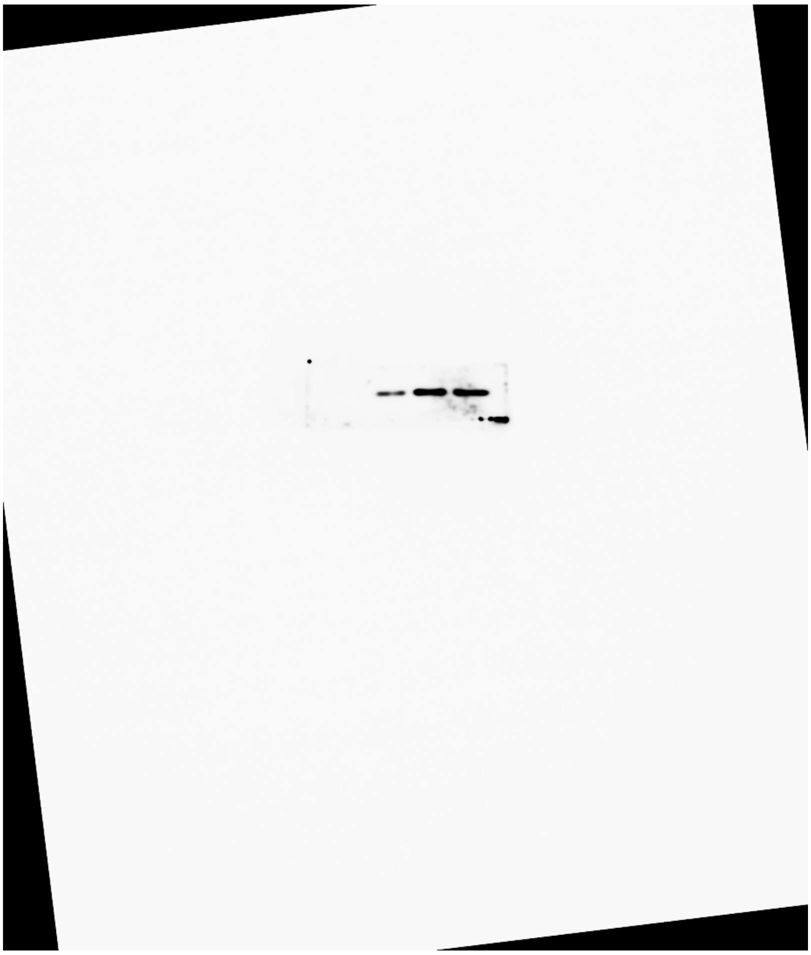

IB: PPFIA4-Flag (from lane 1 to lane 2)

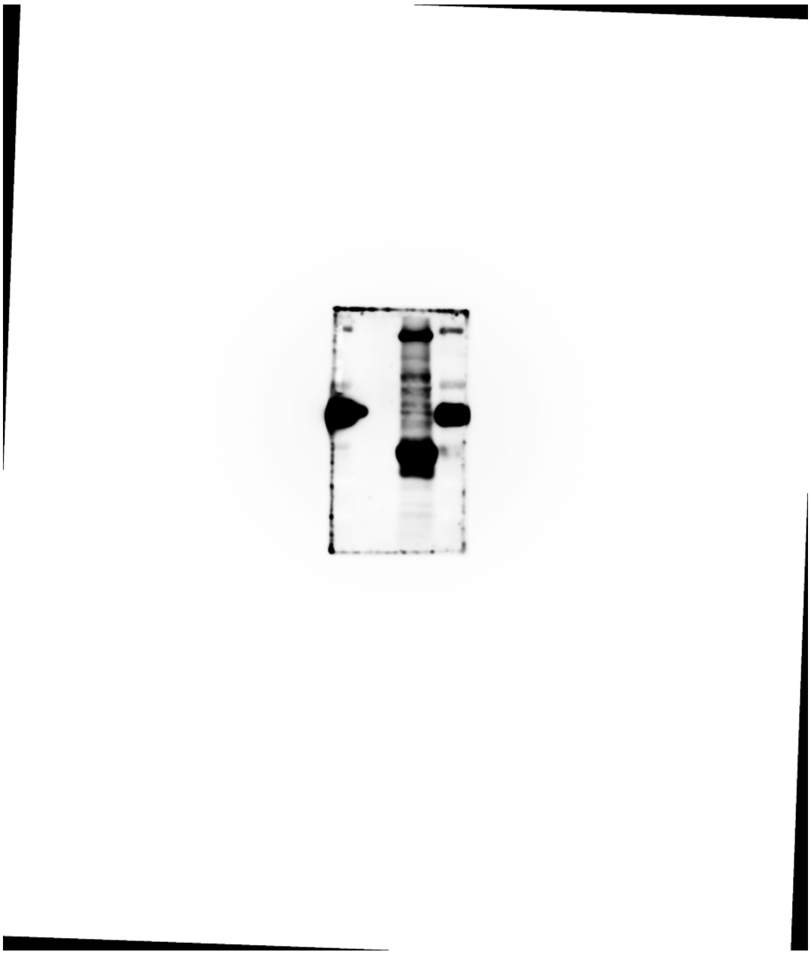

Input: p-AKT1(Thr308) (from lane 3 to lane 4)

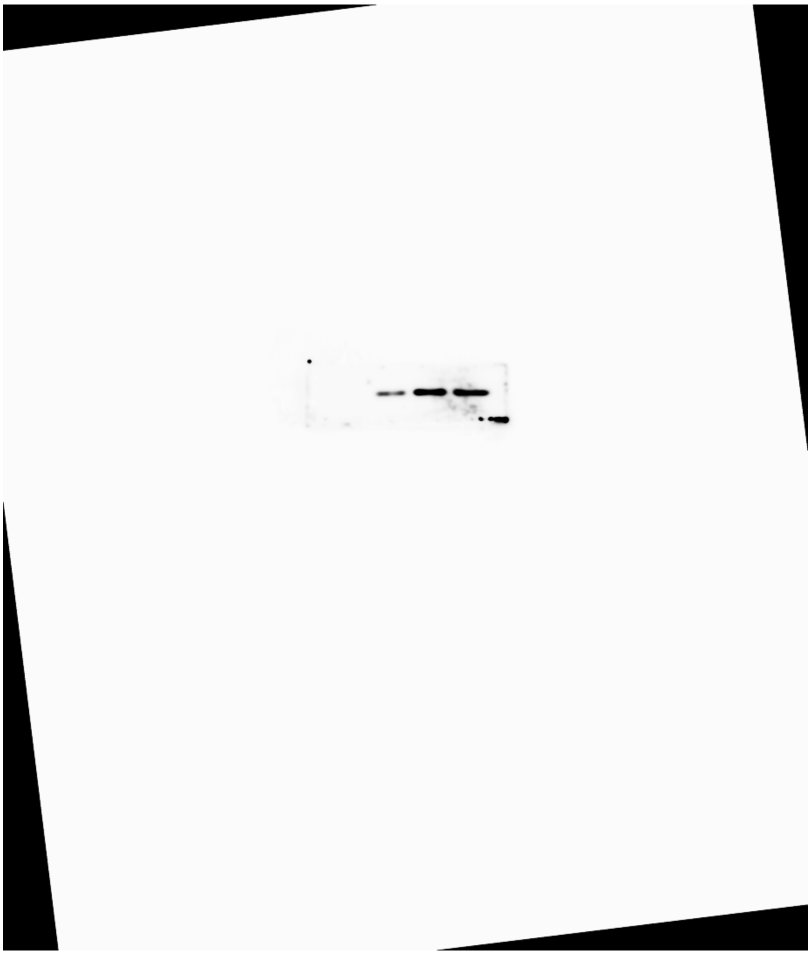

Input: PPFIA4-Flag (from lane 1 to lane 2)

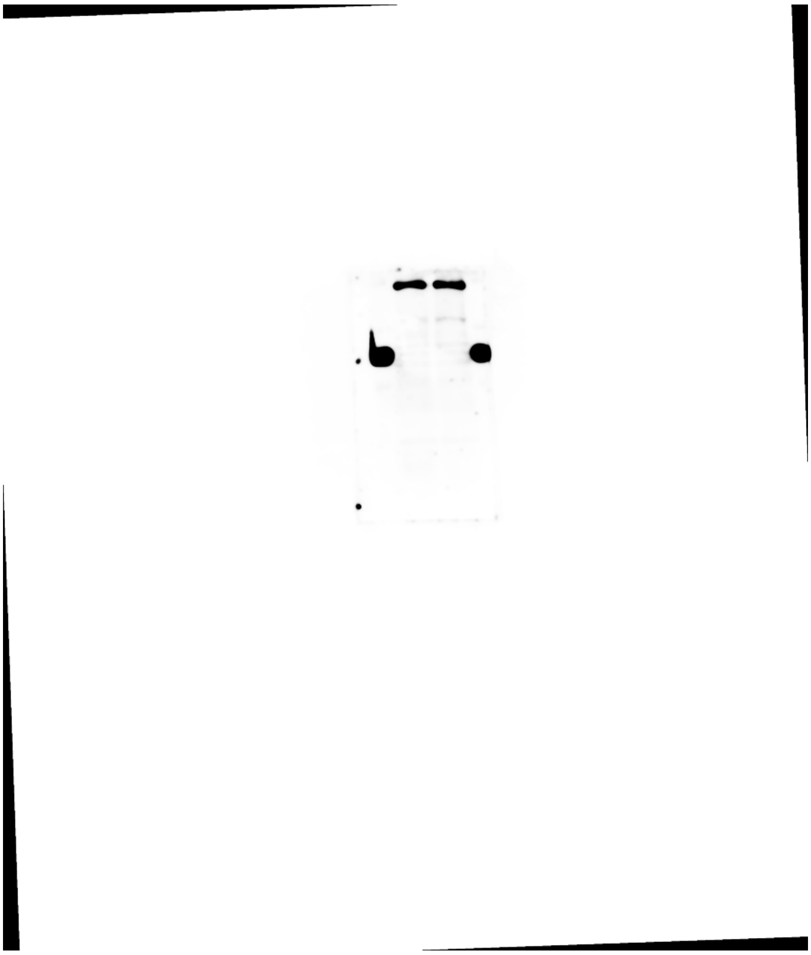

Full unedited gel for Figure 12G(right)

IB: p-AKT1(Thr308) (from lane 1 to lane 2)

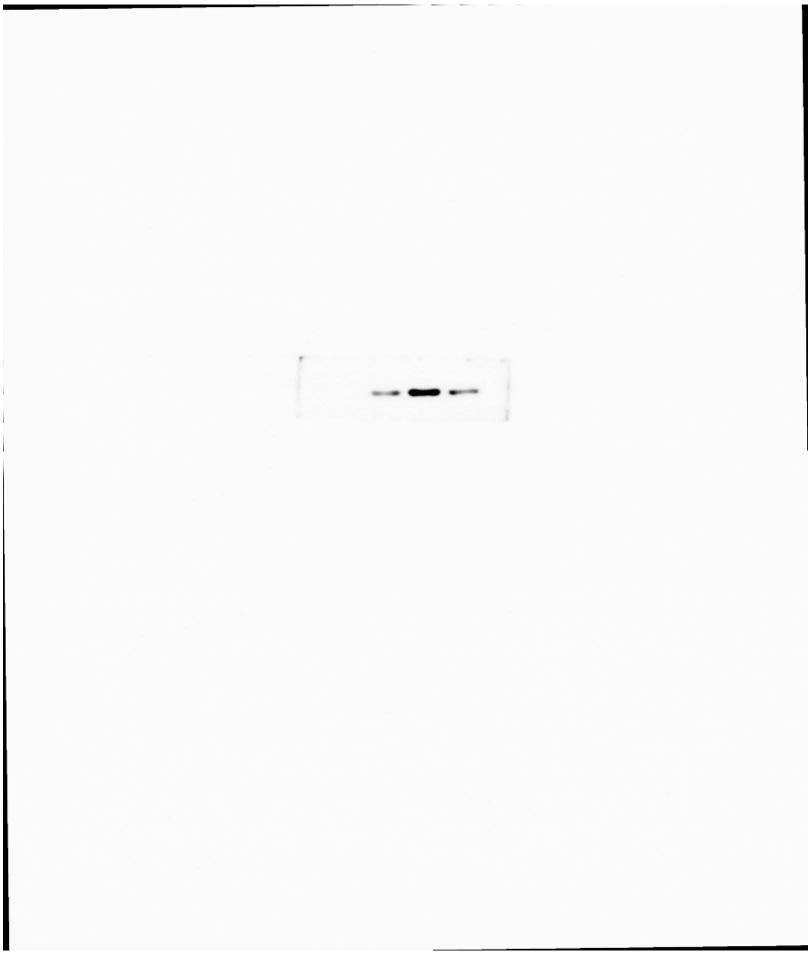

IB: CASK (from lane 3 to lane 4)

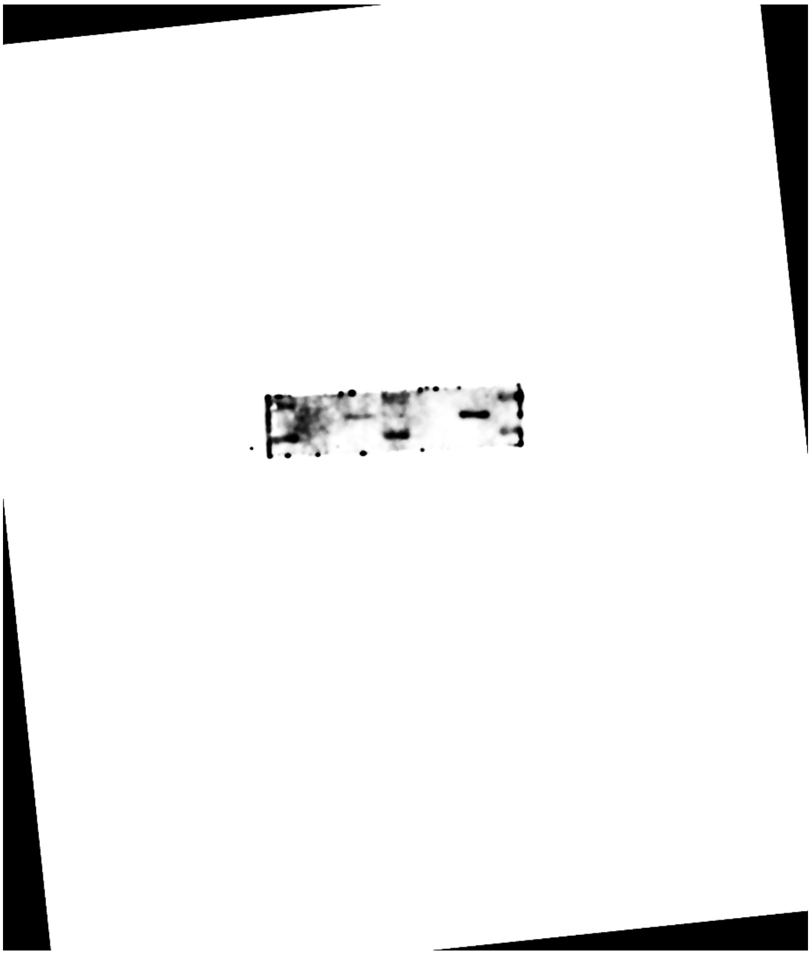

Input: p-AKT1(Thr308) (from lane 1 to lane 2)

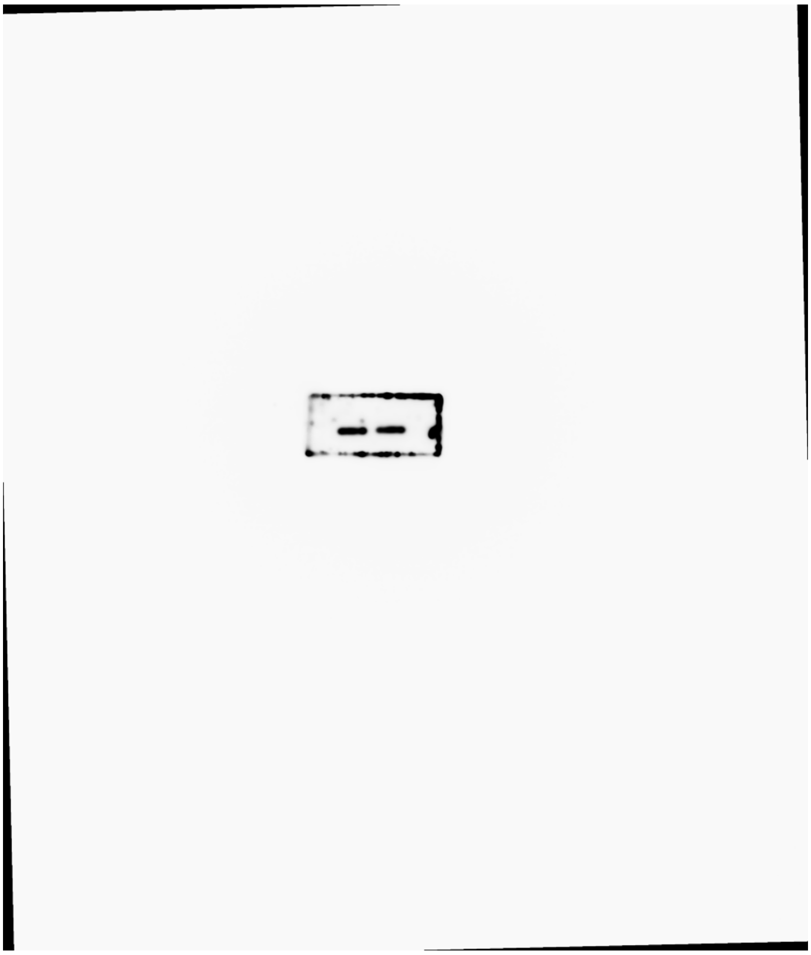

Input: CASK (from lane 3 to lane 4)

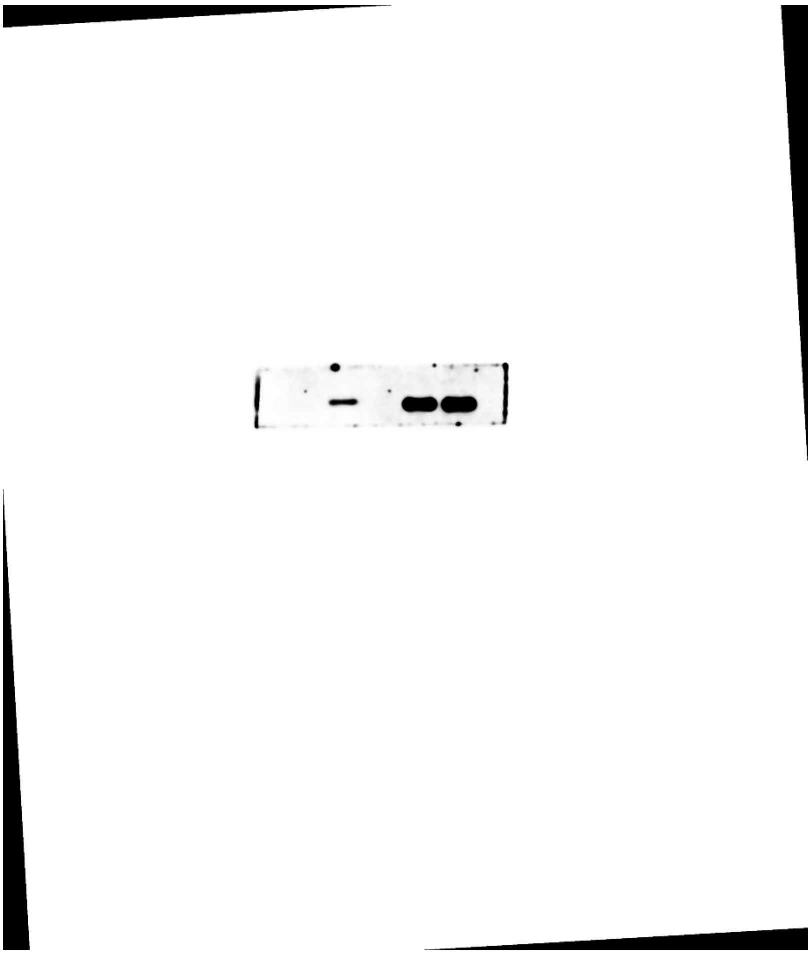

Full unedited gel for Figure 12H(left)
IB: p-AKT1(Ser473) (from lane 1 to lane 2)

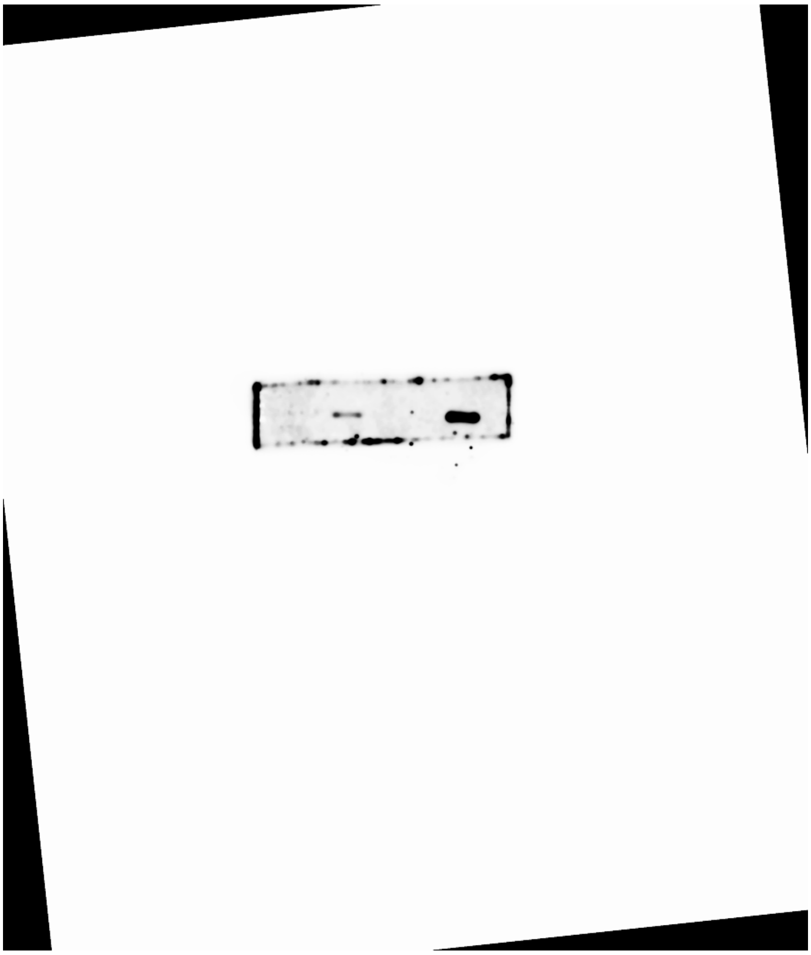

IB: PPFIA4-Flag (from lane 1 to lane 2)

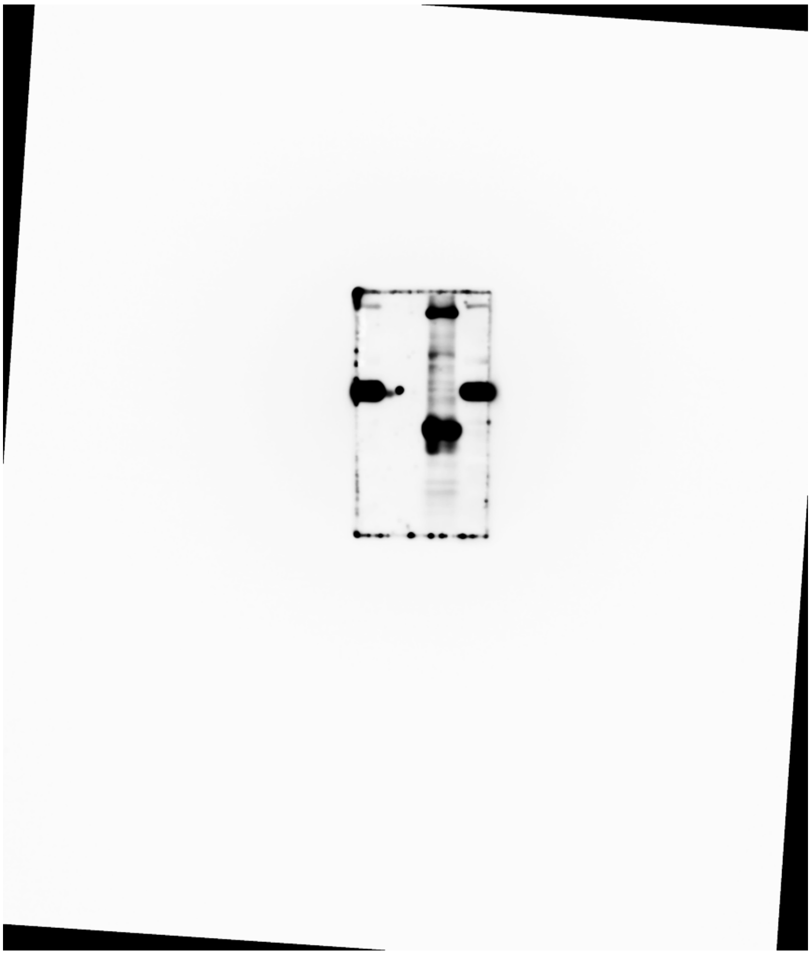

Input: p-AKT1(Ser473) (from lane 3 to lane 4)

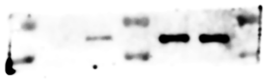

Input: PPFIA4-Flag (from lane 1 to lane 2)

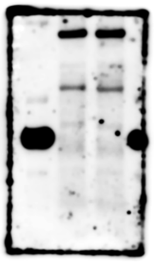

Full unedited gel for Figure 12H(right)

IB: p-AKT1(Ser473) (from lane 1 to lane 2)

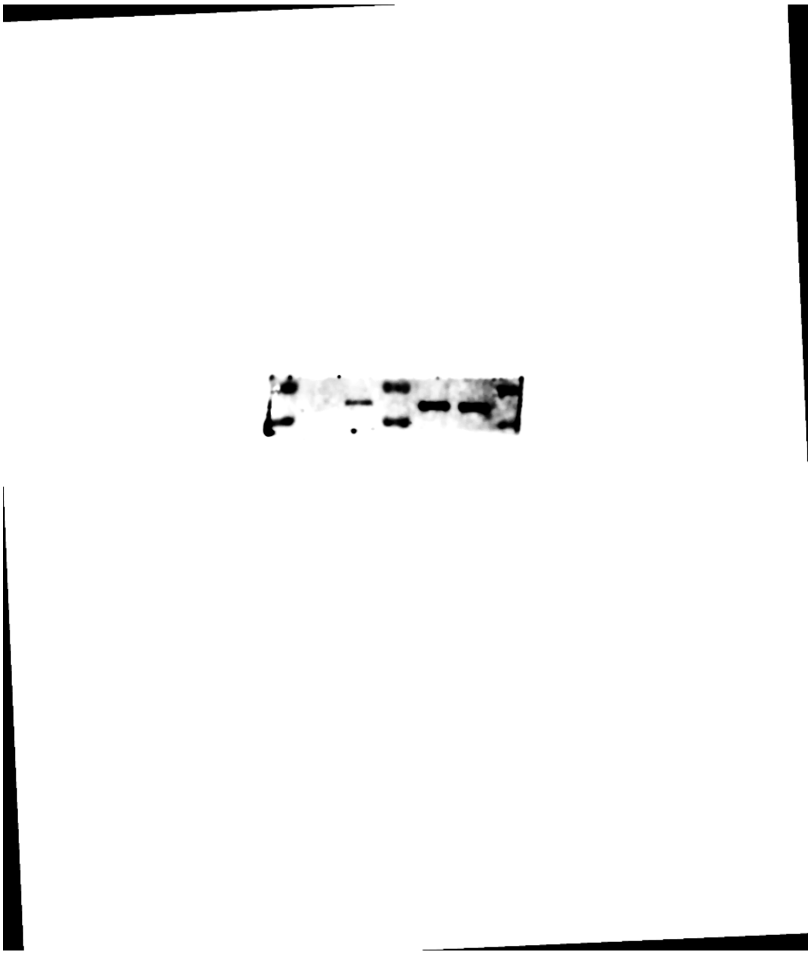

IB: CASK (from lane 1 to lane 2)

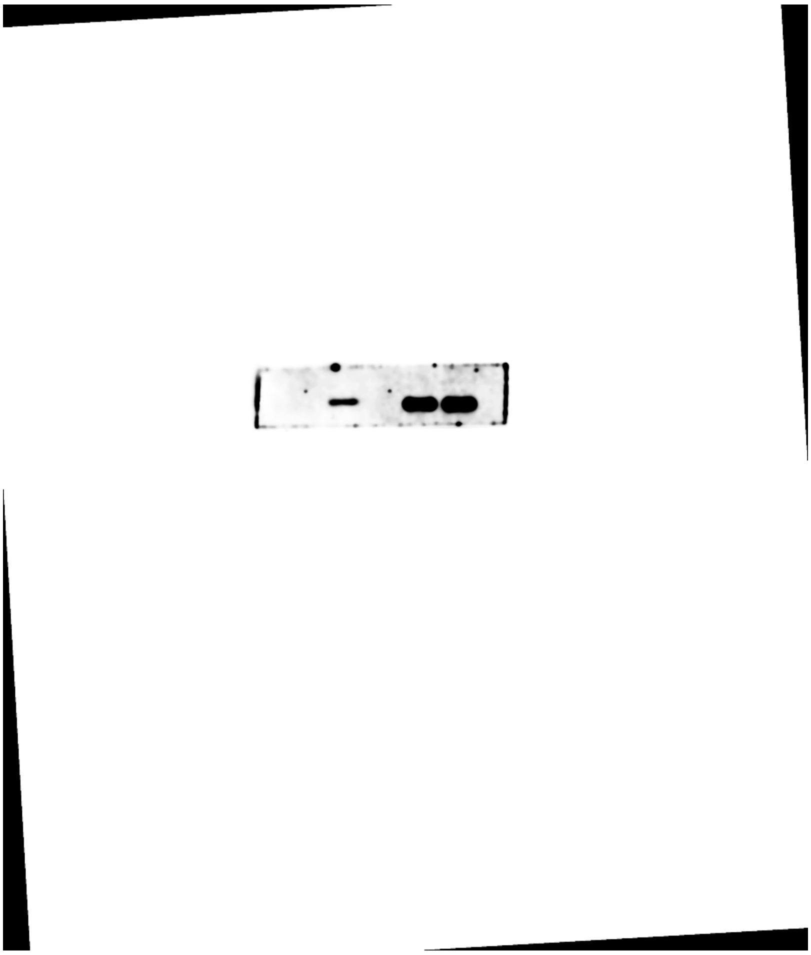

Input: p-AKT1(Ser473) (from lane 3 to lane 4)

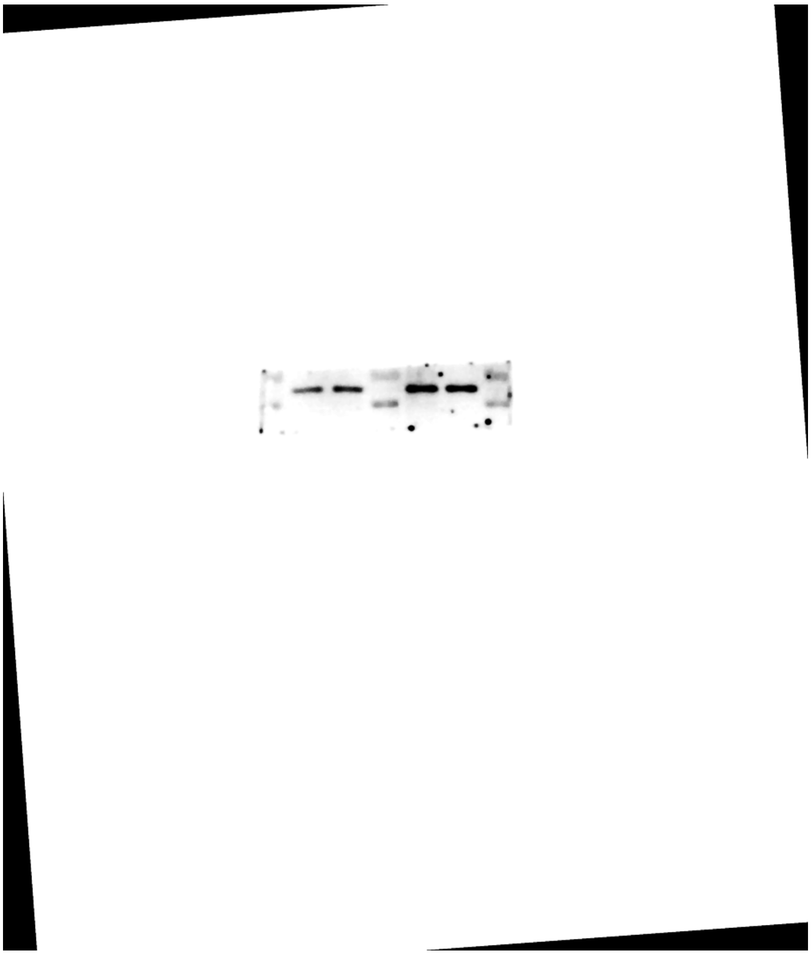

Input: CASK (from lane 3 to lane 4)

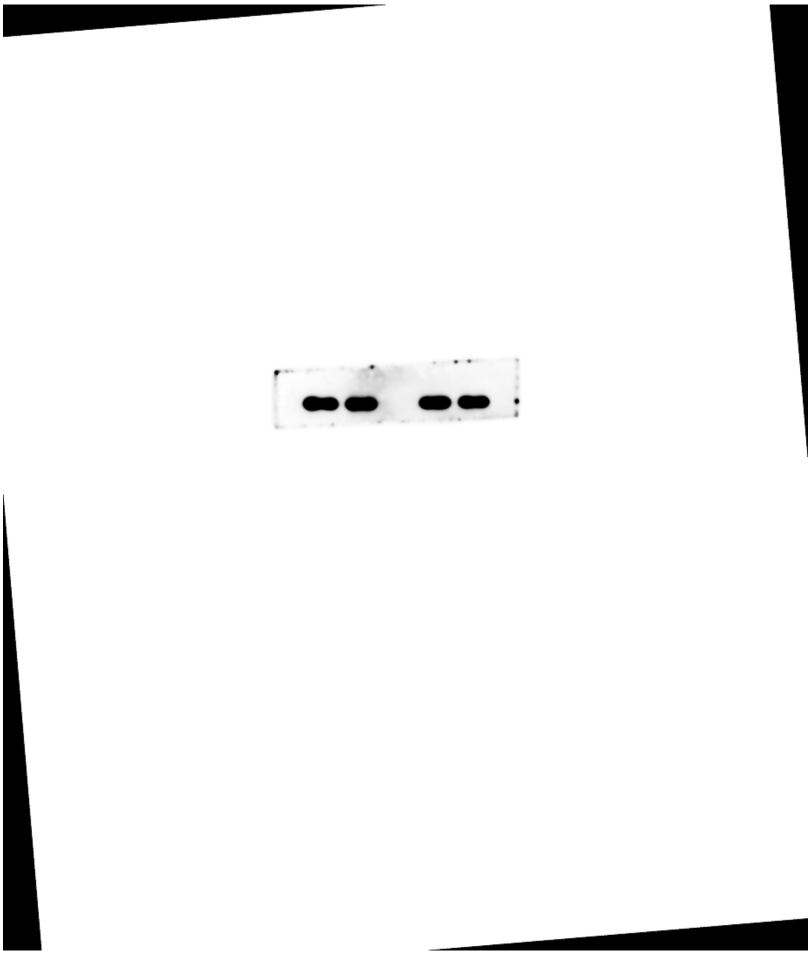

Full unedited gel for Figure 13A

PPFIA4 (from lane 1 to lane 4)

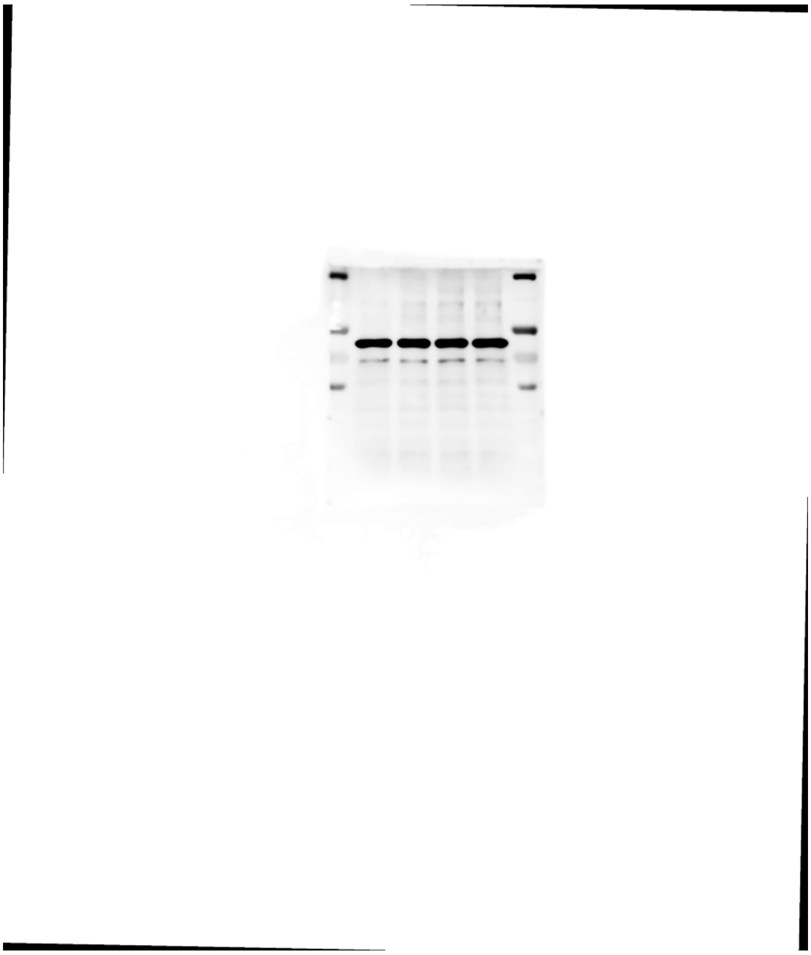

CASK (from lane 1 to lane 4)

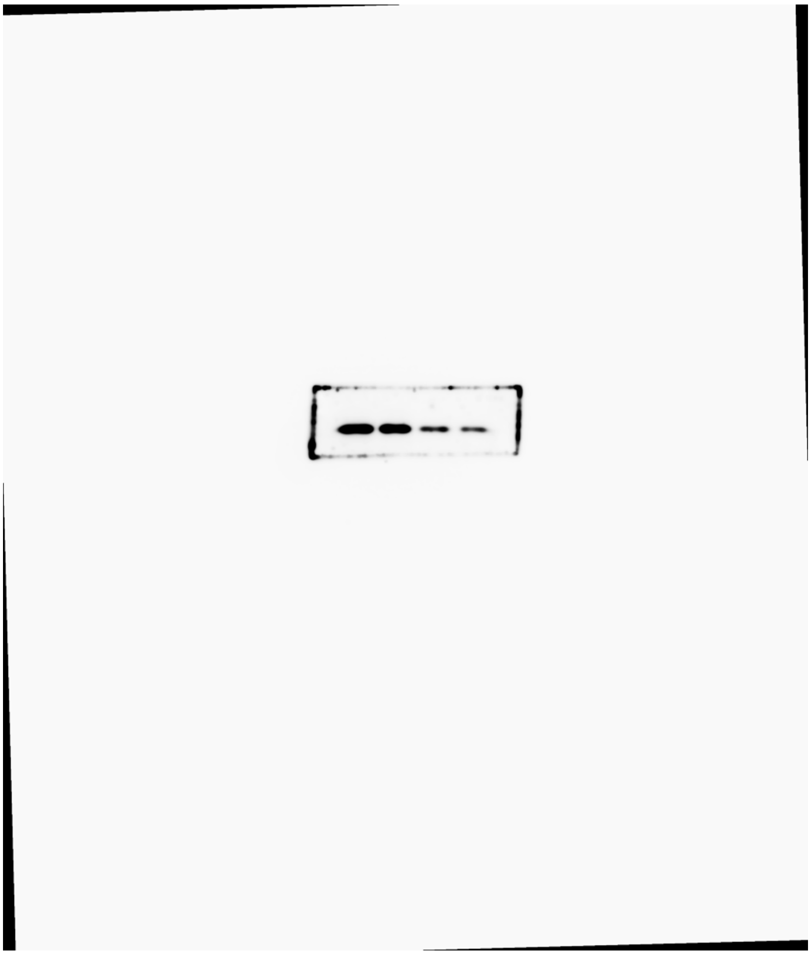

AKT1 (from lane 1 to lane 4)

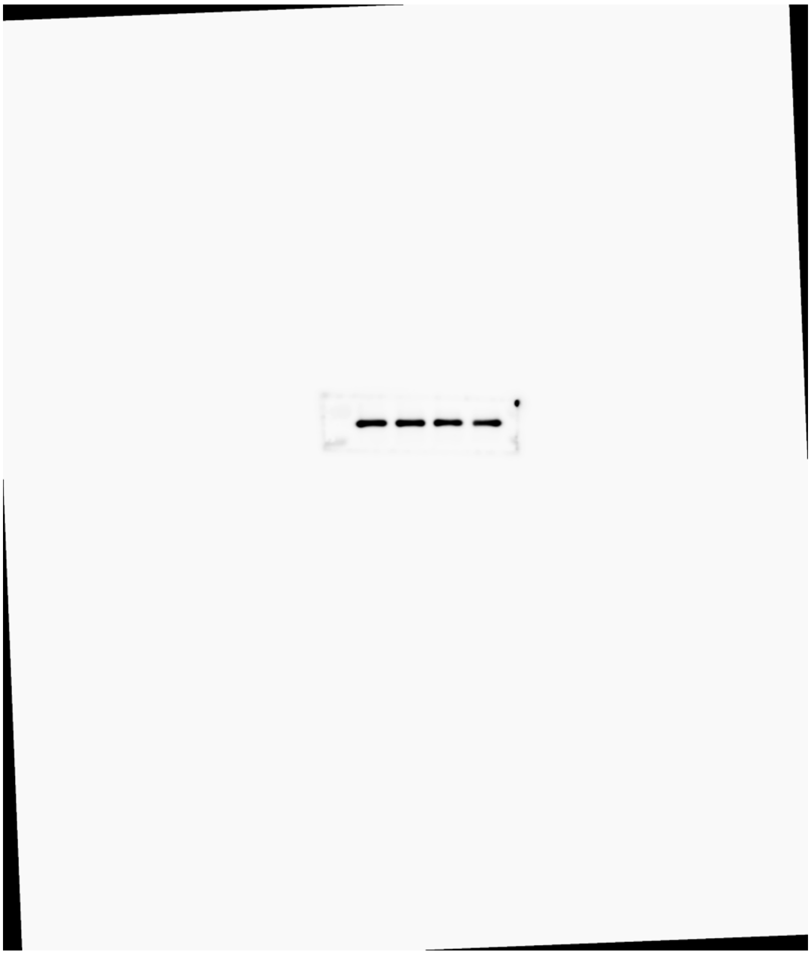

p-AKT1(Thr308) (from lane 1 to lane 4)

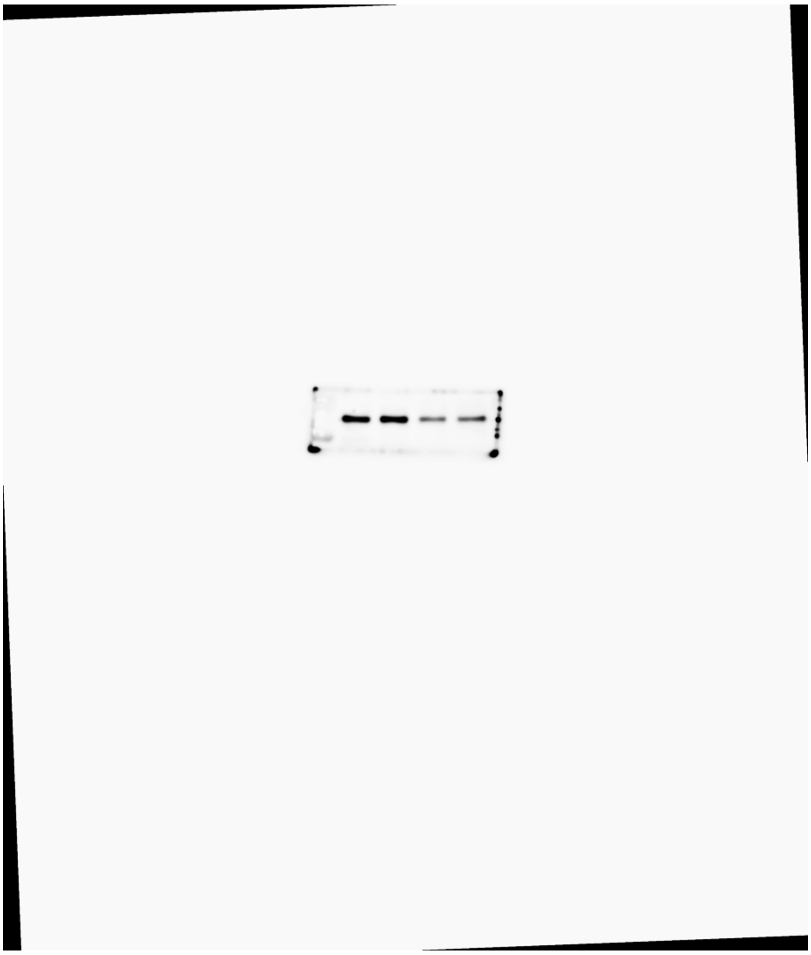

p-AKT1(Ser473) (from lane 1 to lane 4)



GAPDH (from lane 1 to lane 4)

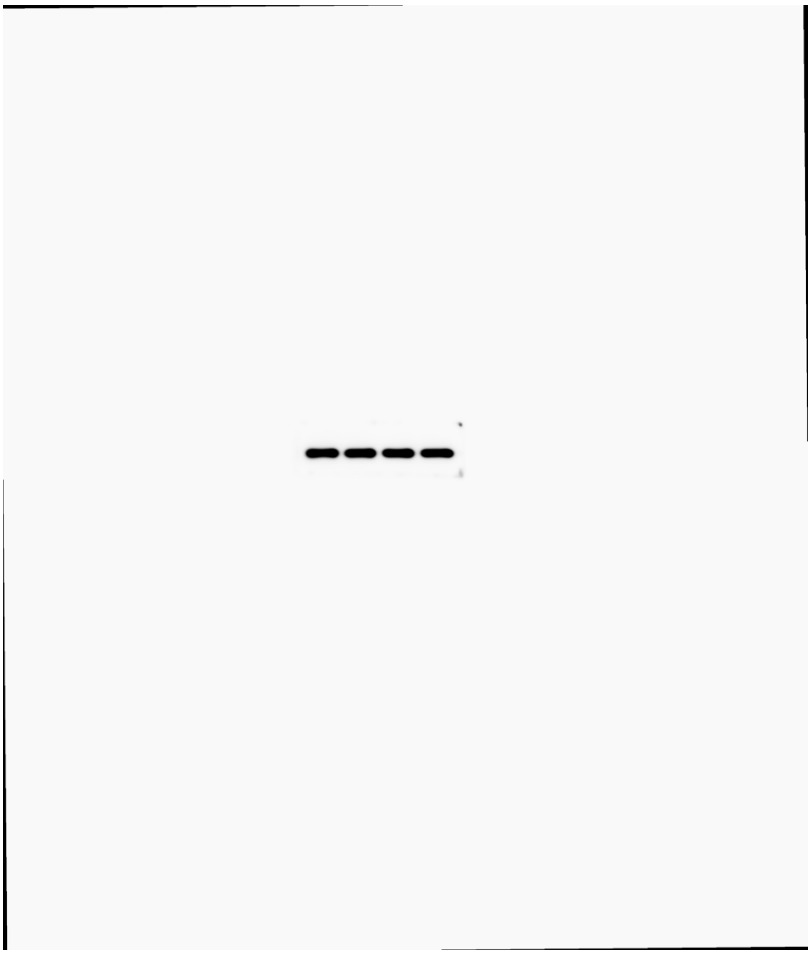

Full unedited gel for Figure 13D(left panel)

PPFIA4 (from lane 1 to lane 4)

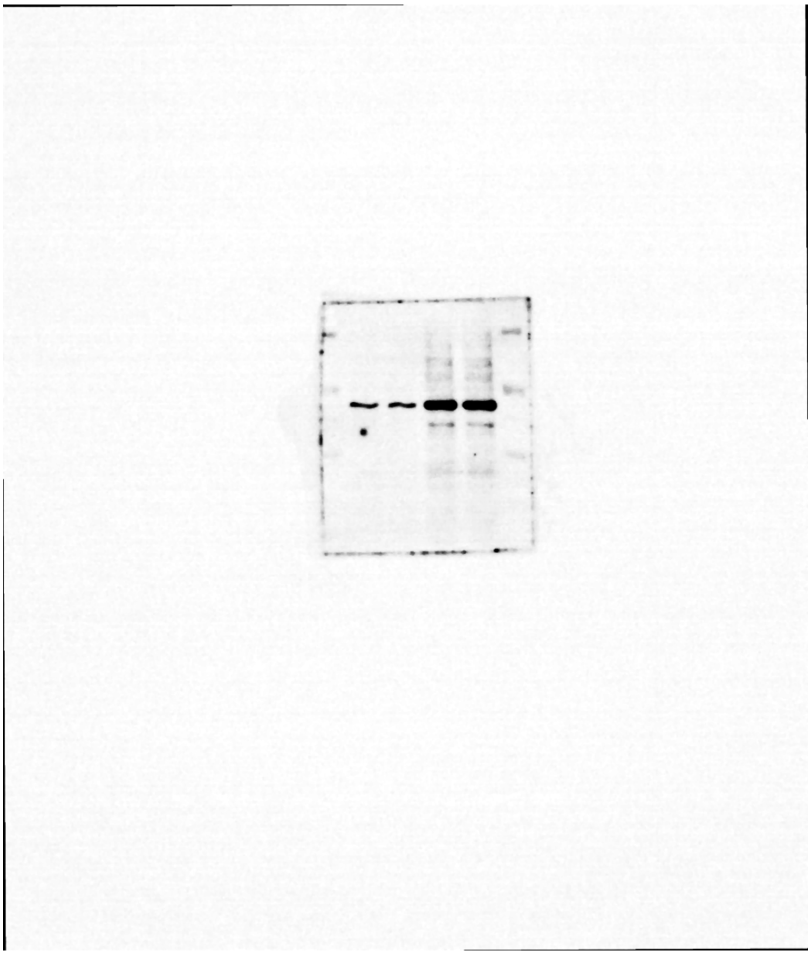

CASK (from lane 1 to lane 4)

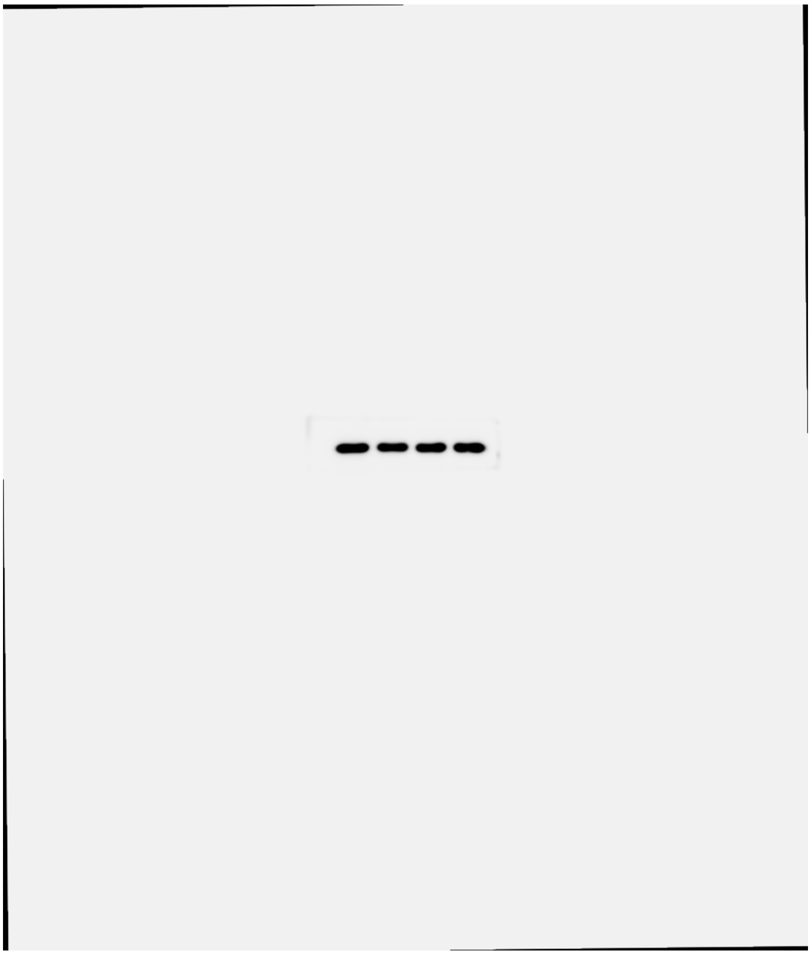

AKT1 (from lane 1 to lane 4)

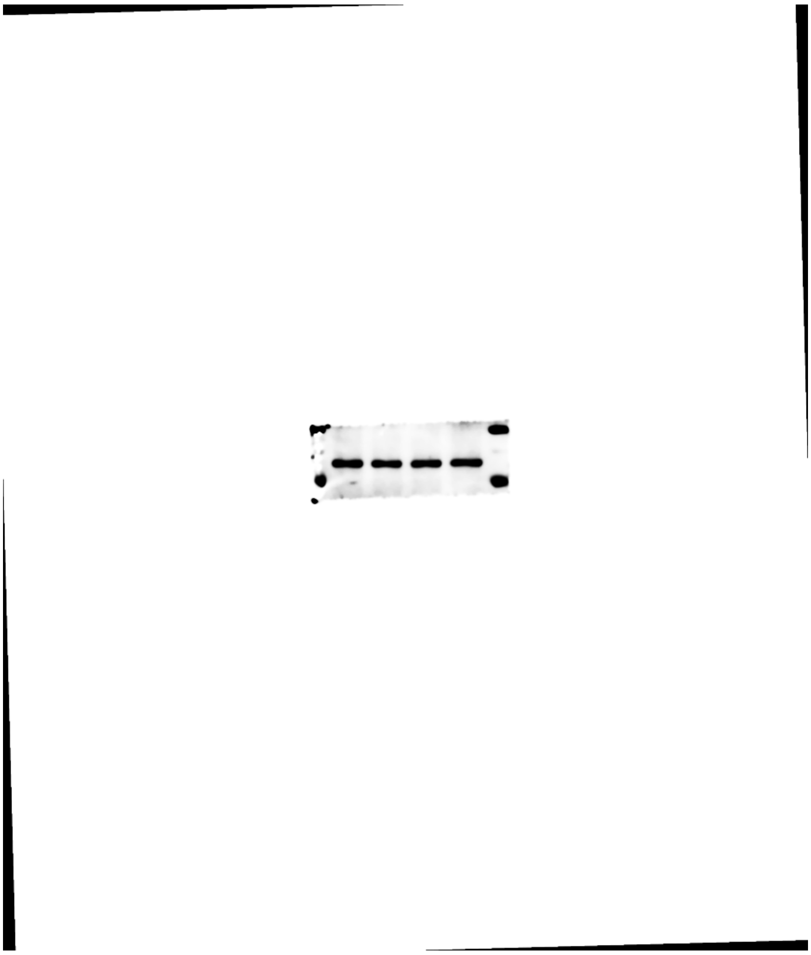

p-AKT1(Thr308) (from lane 1 to lane 4)

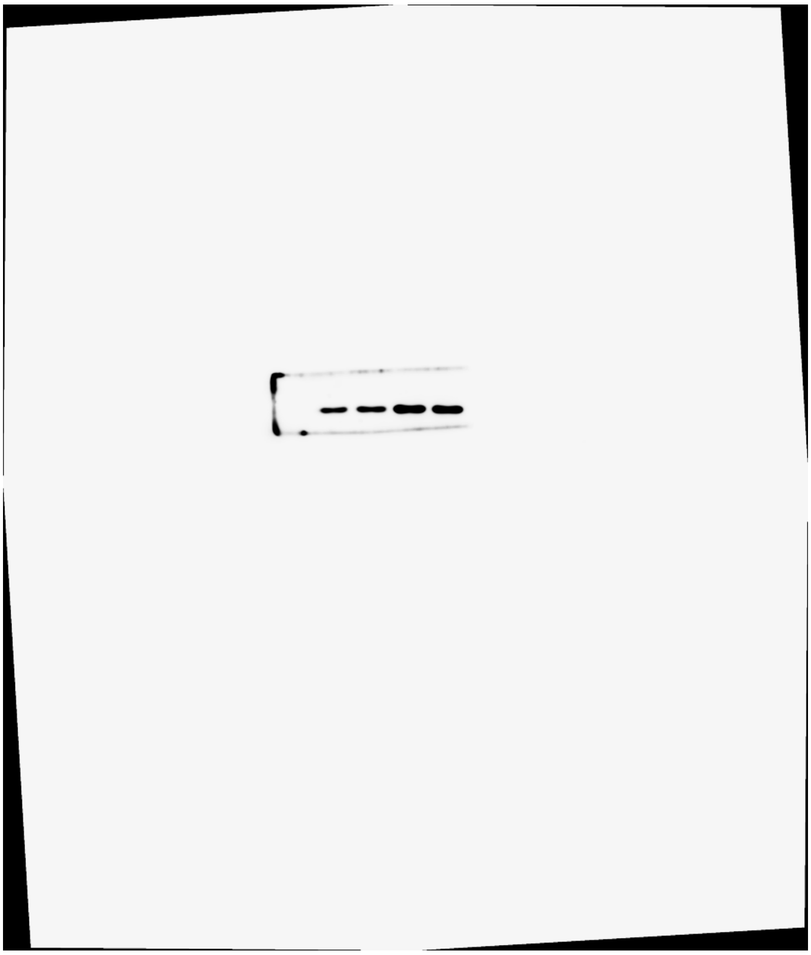

p-AKT1(Ser473) (from lane 1 to lane 4)

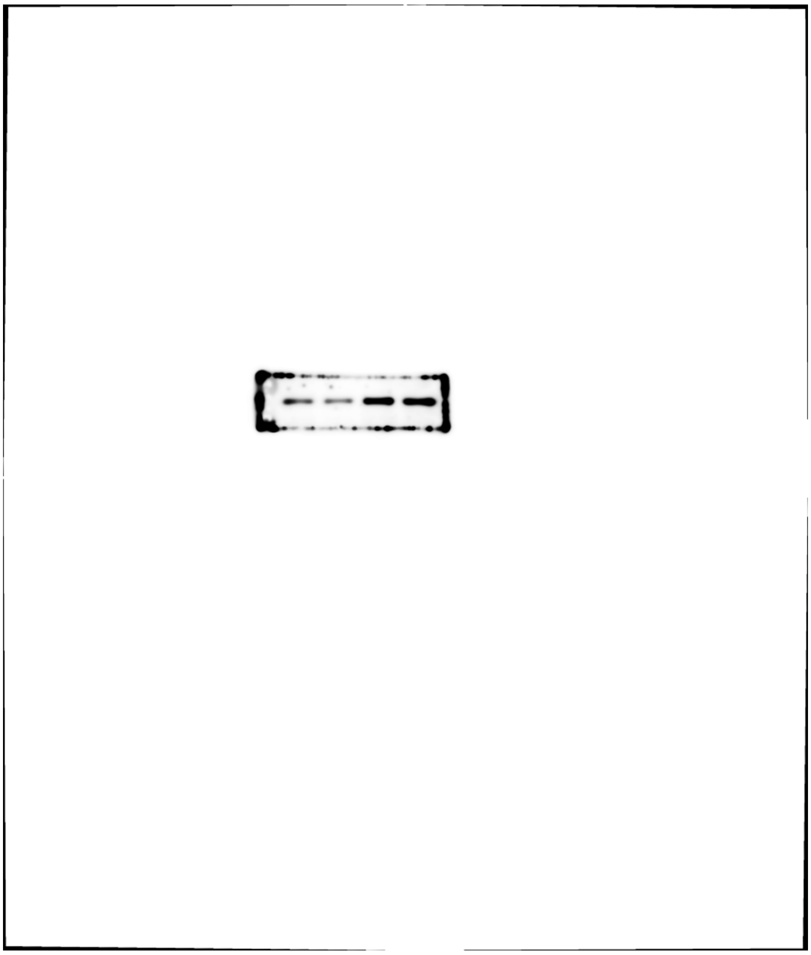

p65 (from lane 1 to lane 4)

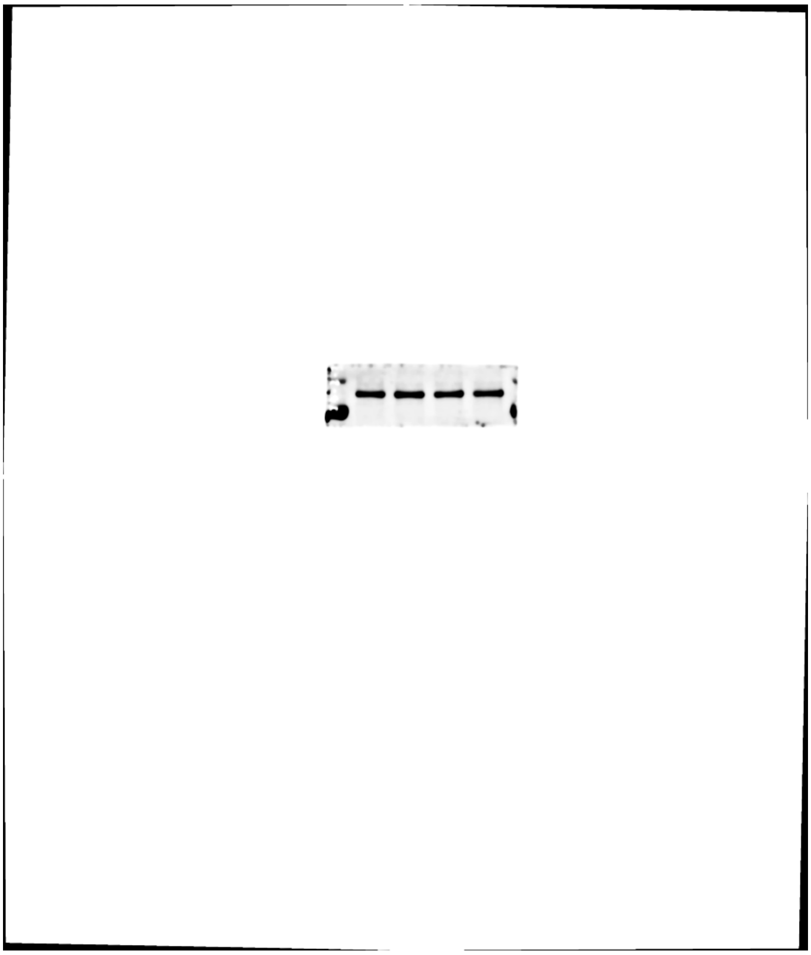

p-p65 (from lane 1 to lane 4)

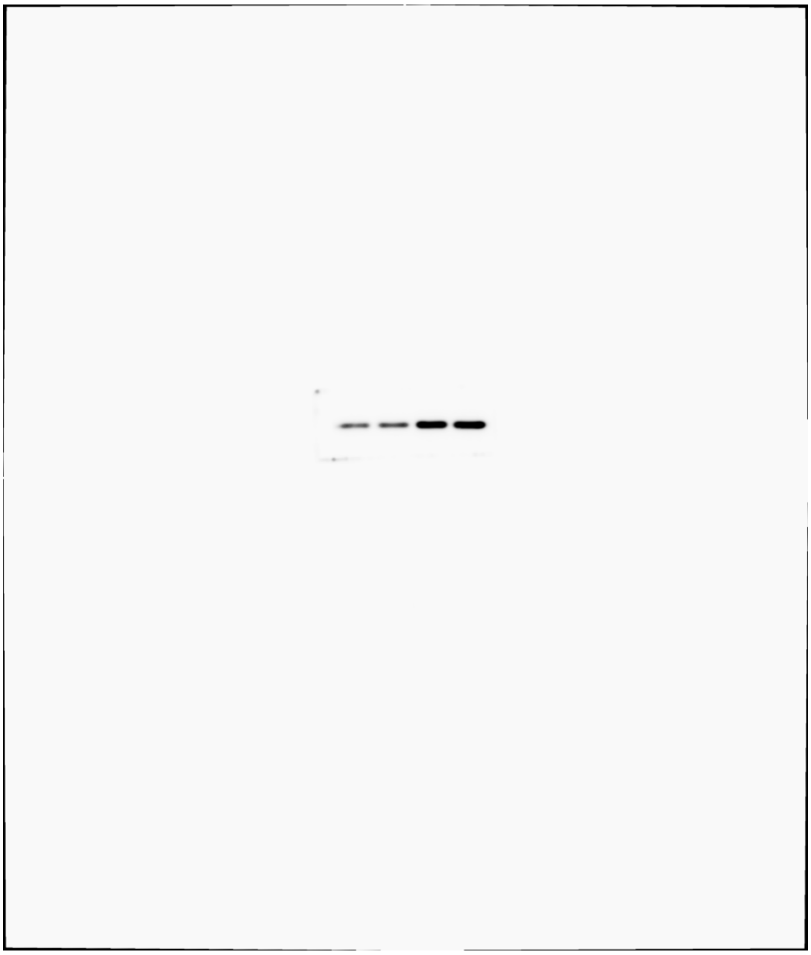

GAPDH (from lane 1 to lane 4)

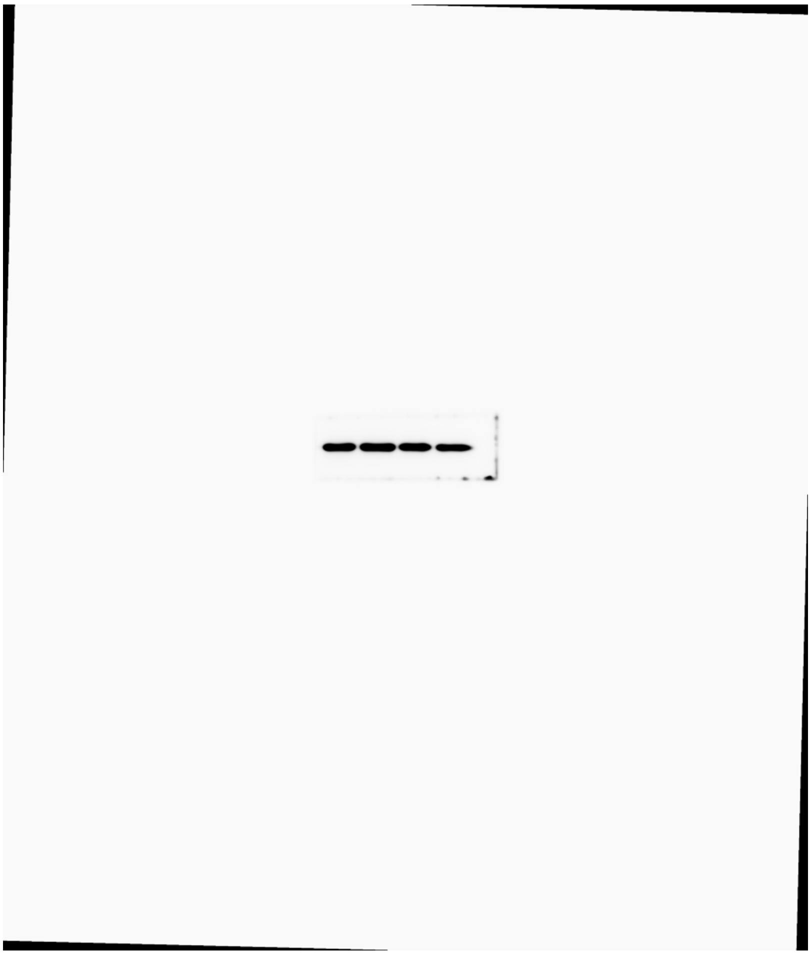

Full unedited gel for Figure 13D(middle panel)

PPFIA4 (from lane 1 to lane 4)

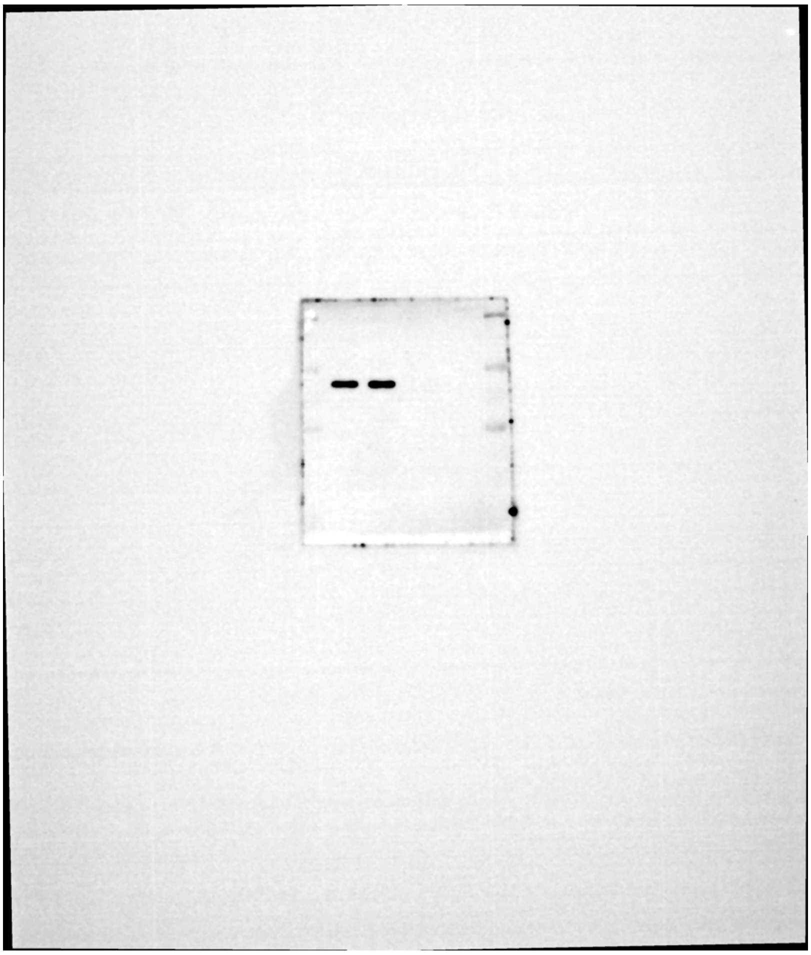

CASK (from lane 1 to lane 4)

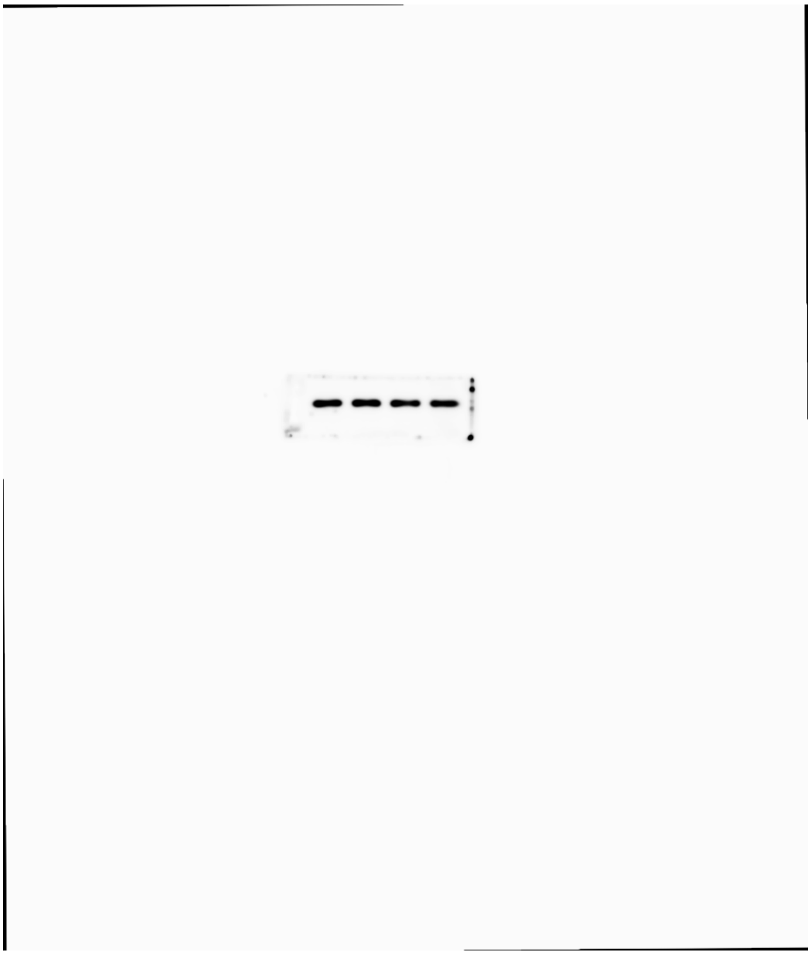

AKT1 (from lane 1 to lane 4)

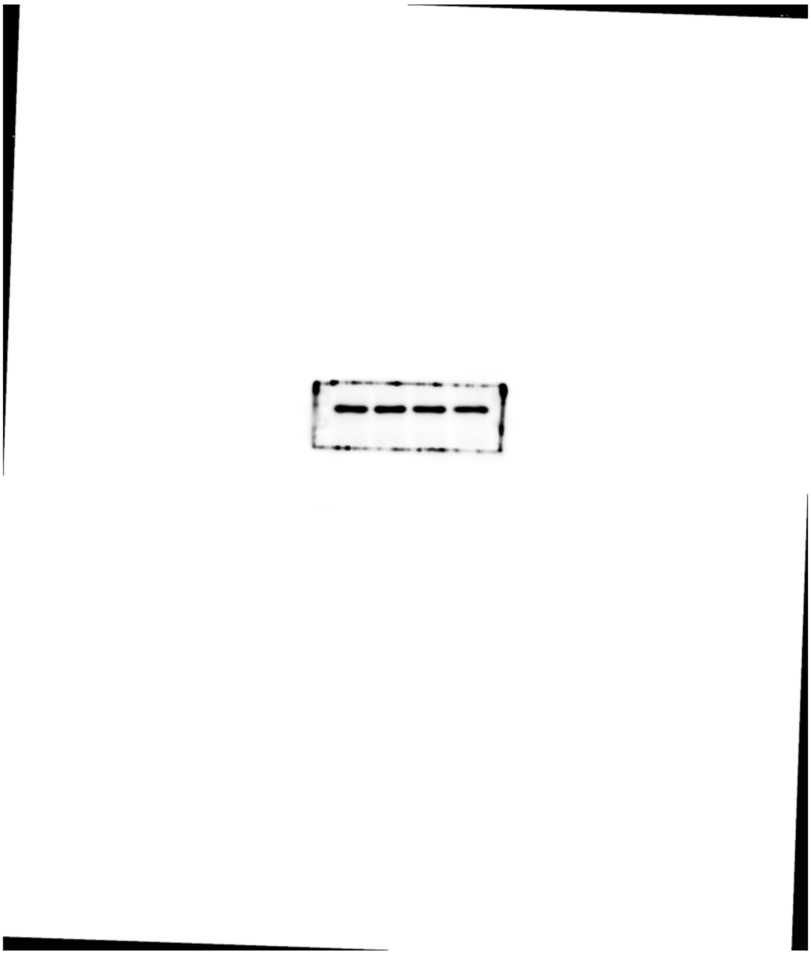

p-AKT1(Thr308) (from lane 1 to lane 4)

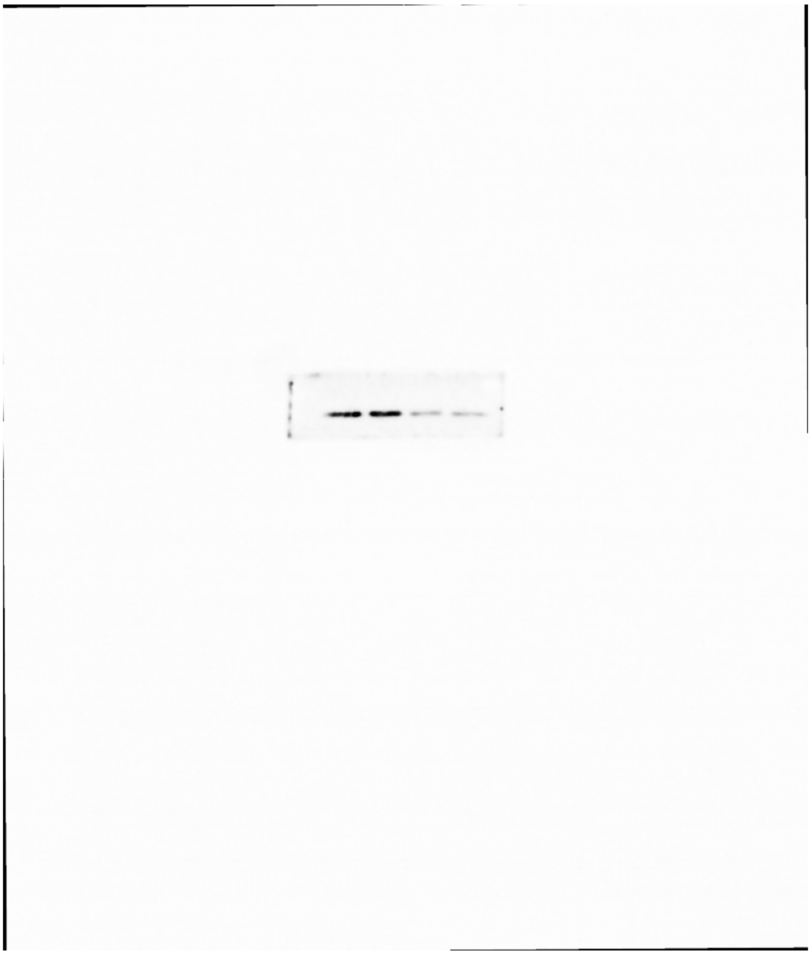

p-AKT1(Ser473) (from lane 1 to lane 4)

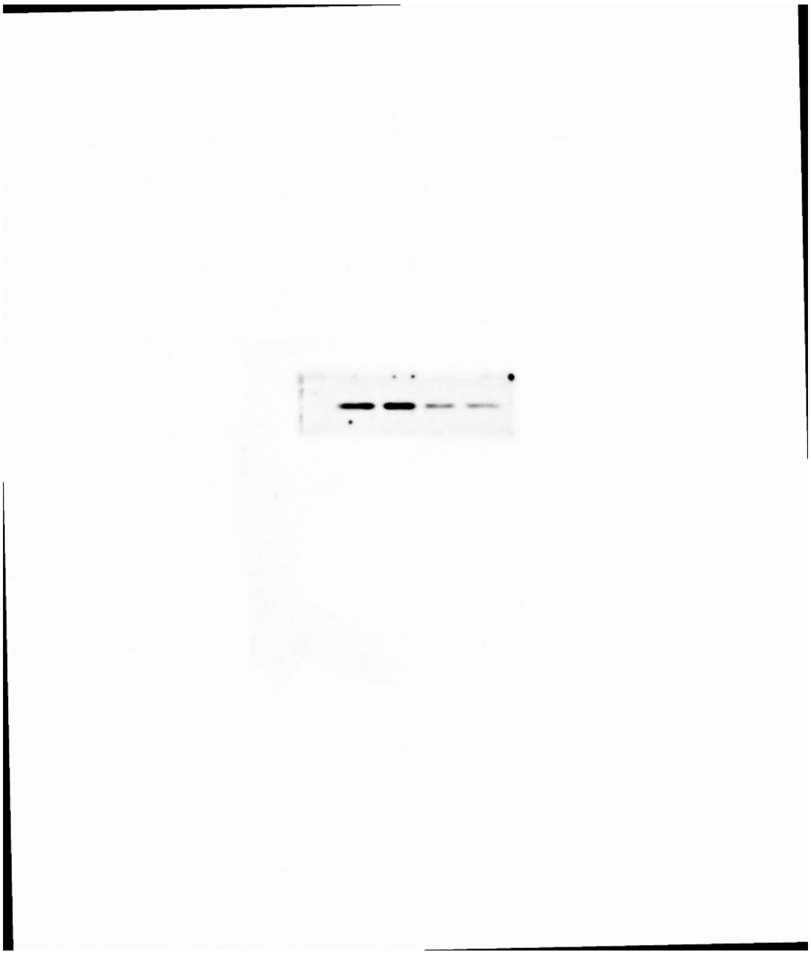

p65 (from lane 1 to lane 4)

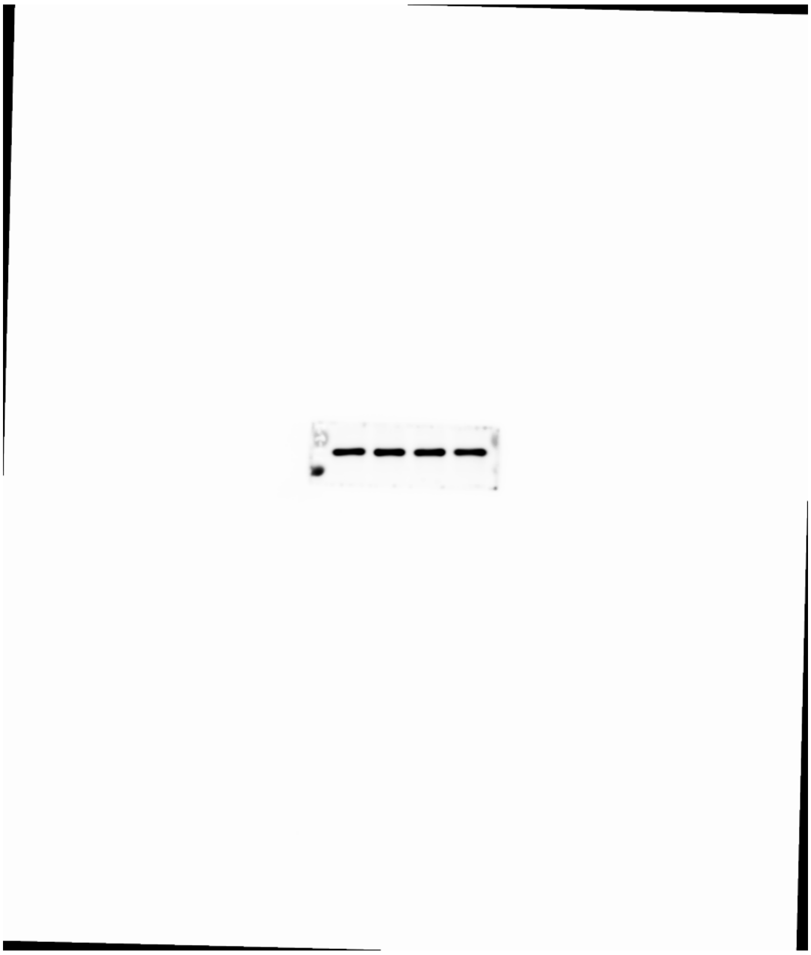

p-p65 (from lane 1 to lane 4)

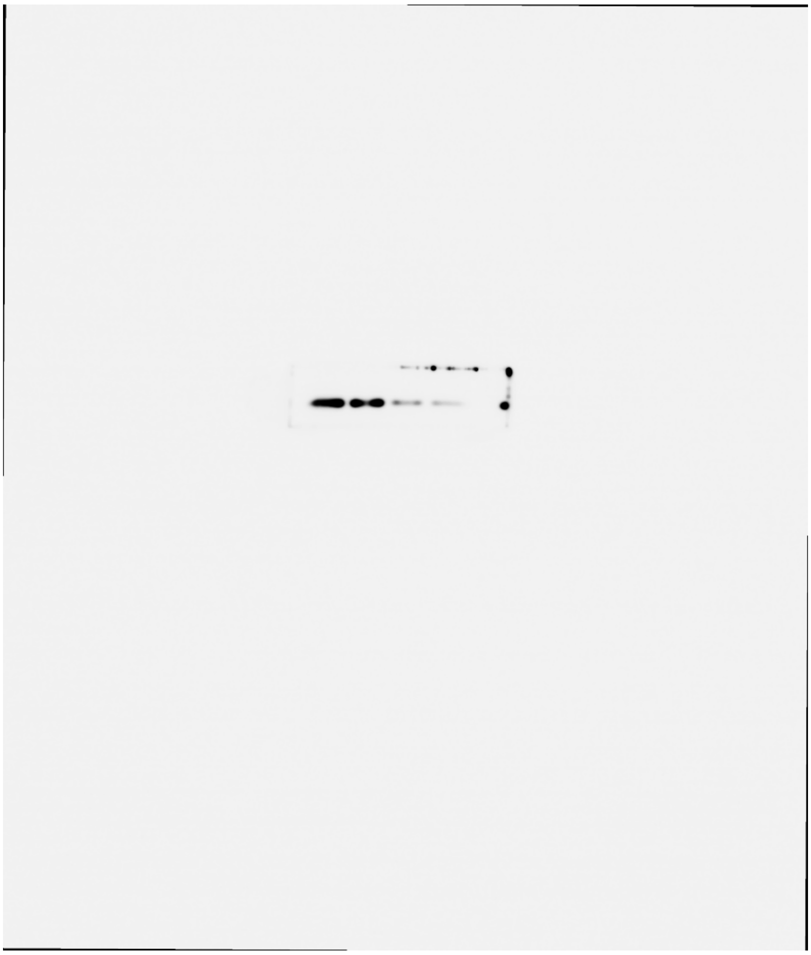

GAPDH (from lane 1 to lane 4)

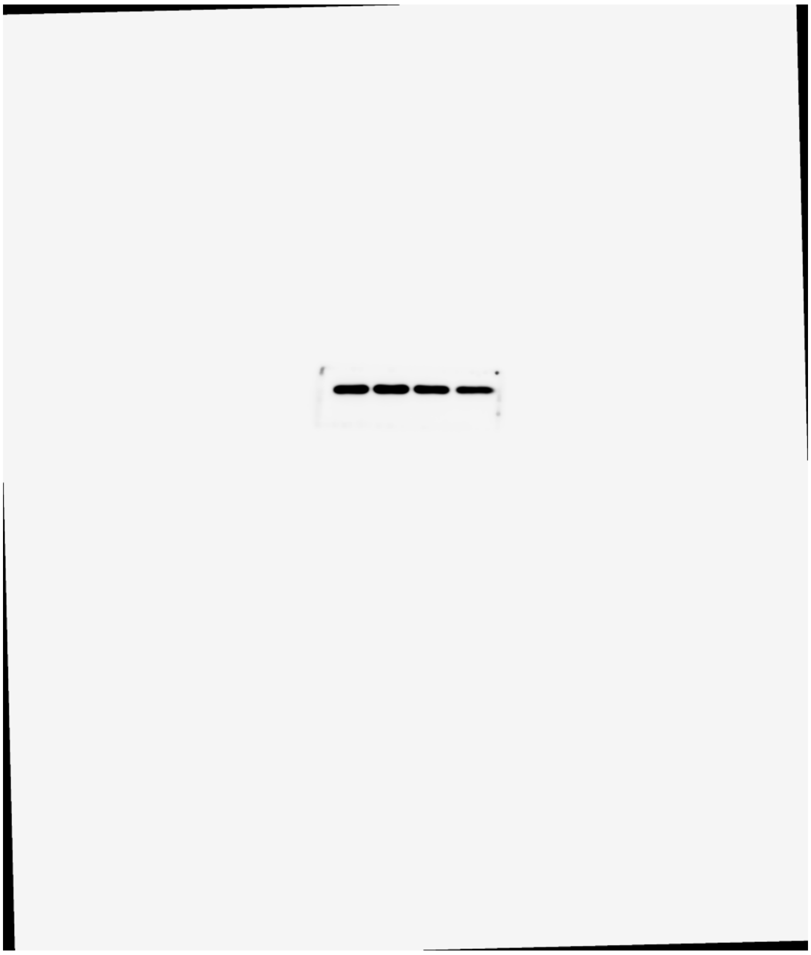

Full unedited gel for Figure 13D(right panel)

PPFIA4 (from lane 1 to lane 4)

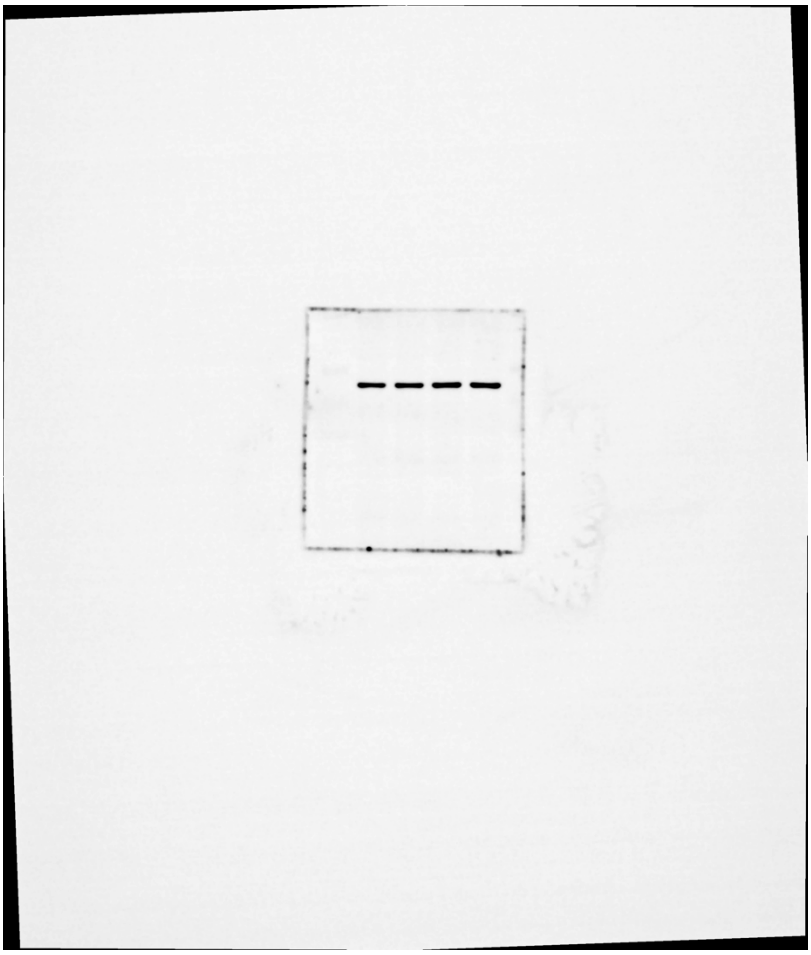

CASK (from lane 1 to lane 4)

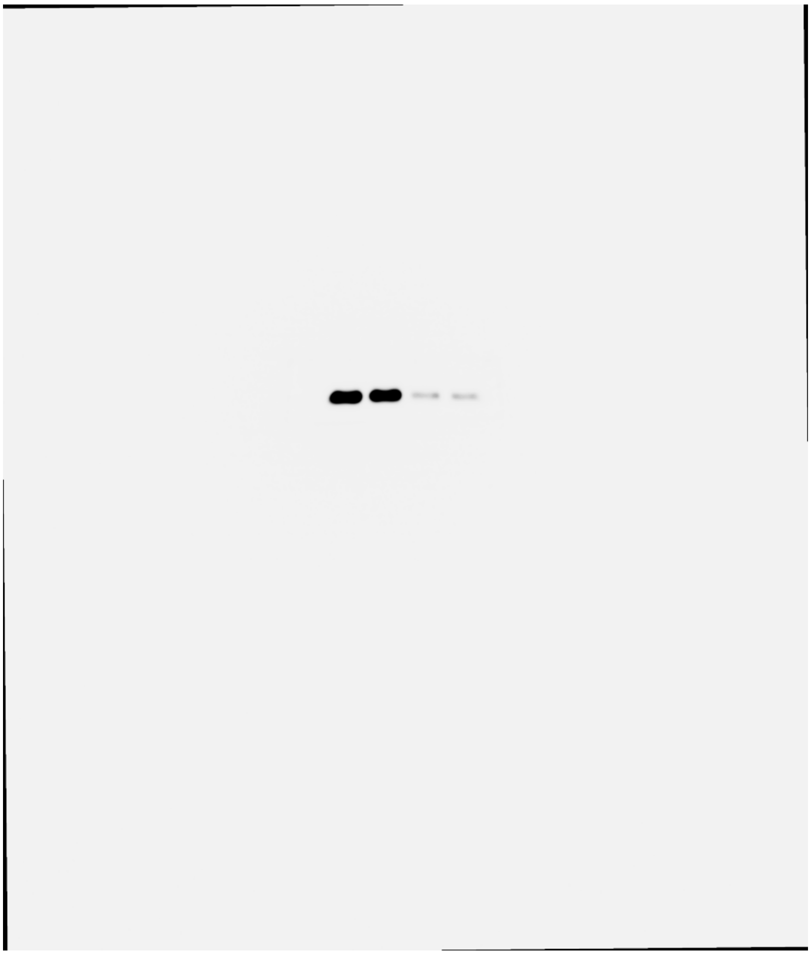

AKT1 (from lane 1 to lane 4)

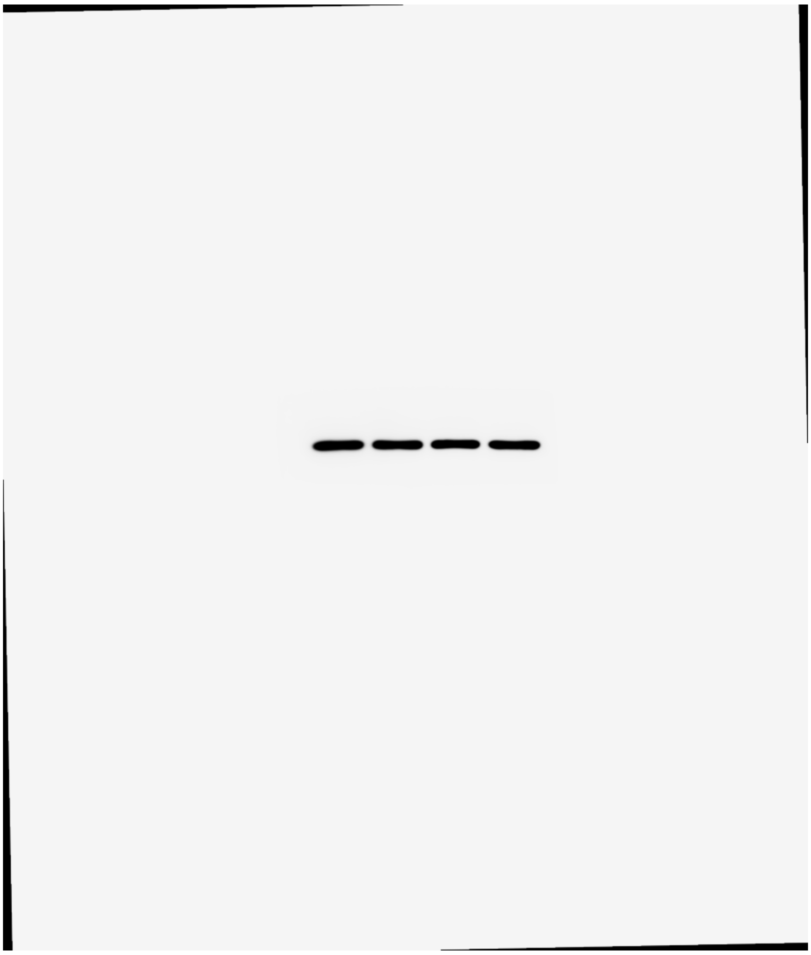

p-AKT1(Thr308) (from lane 1 to lane 4)

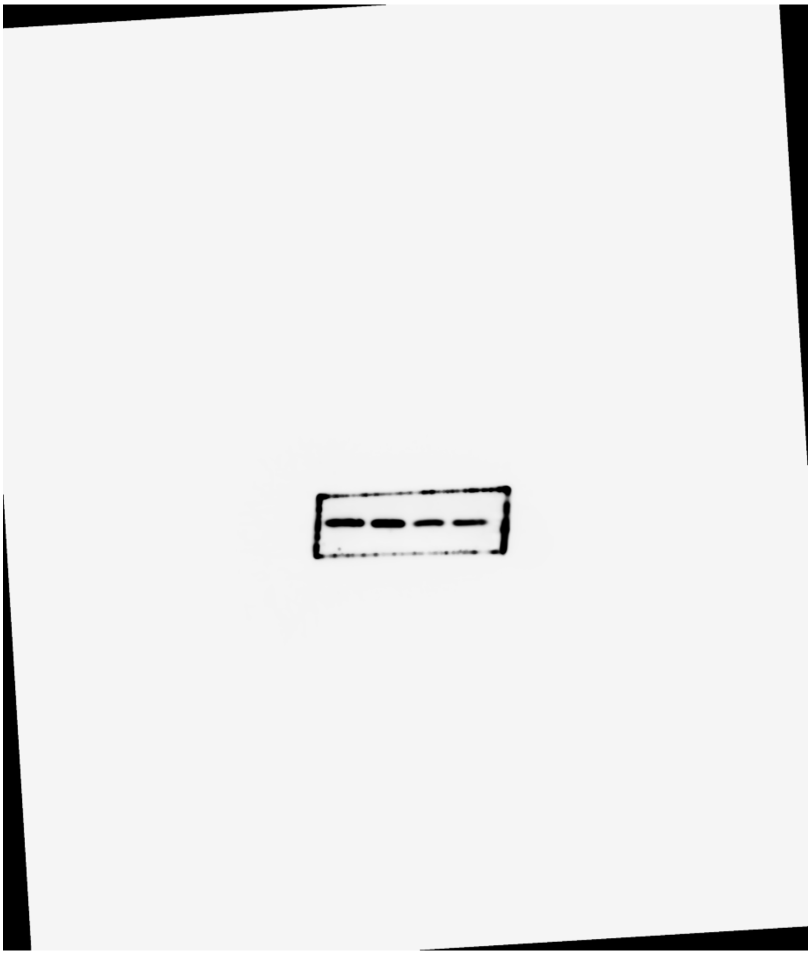

p-AKT1(Ser473) (from lane 1 to lane 4)

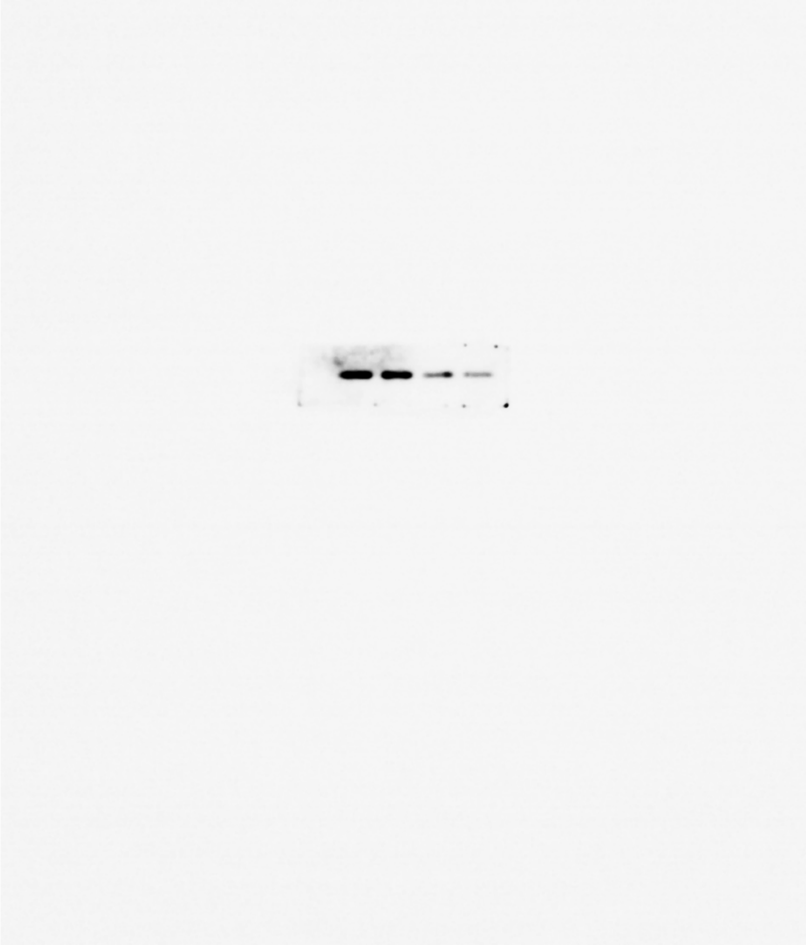

p65 (from lane 1 to lane 4)

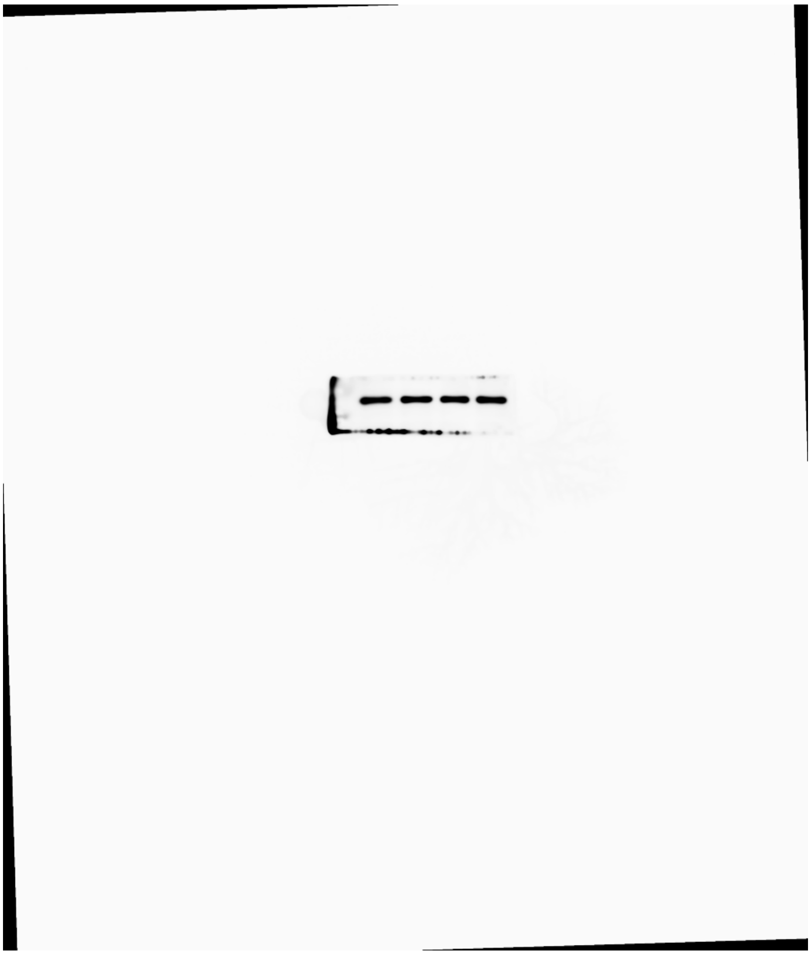

p-p65 (from lane 1 to lane 4)

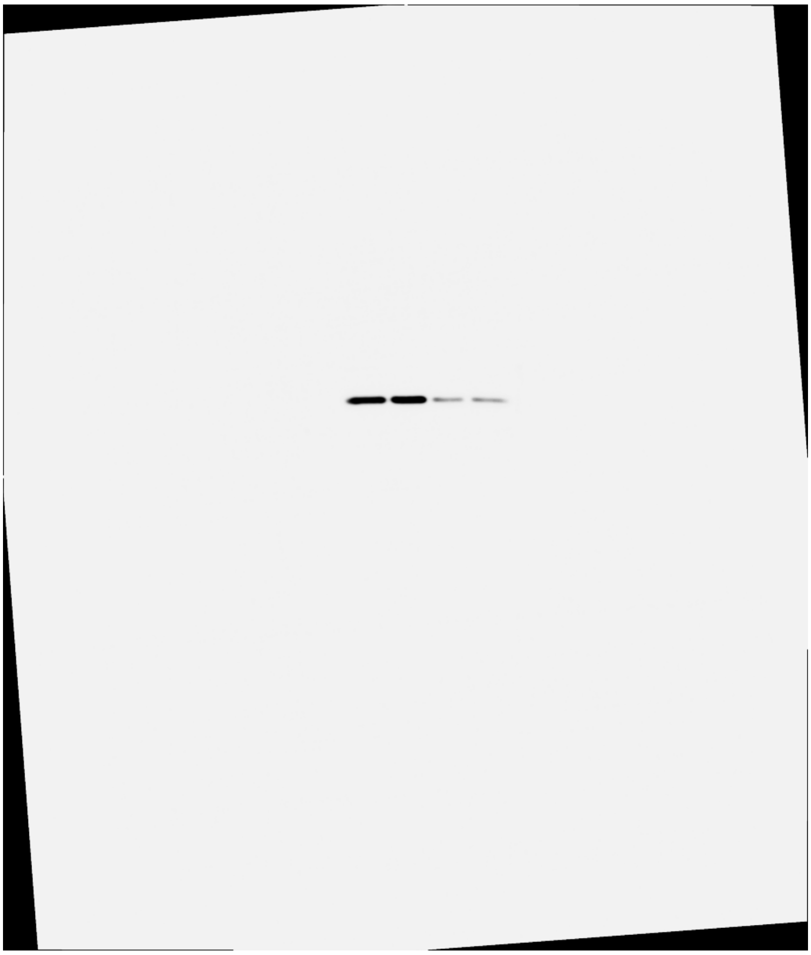

GAPDH (from lane 1 to lane 4)

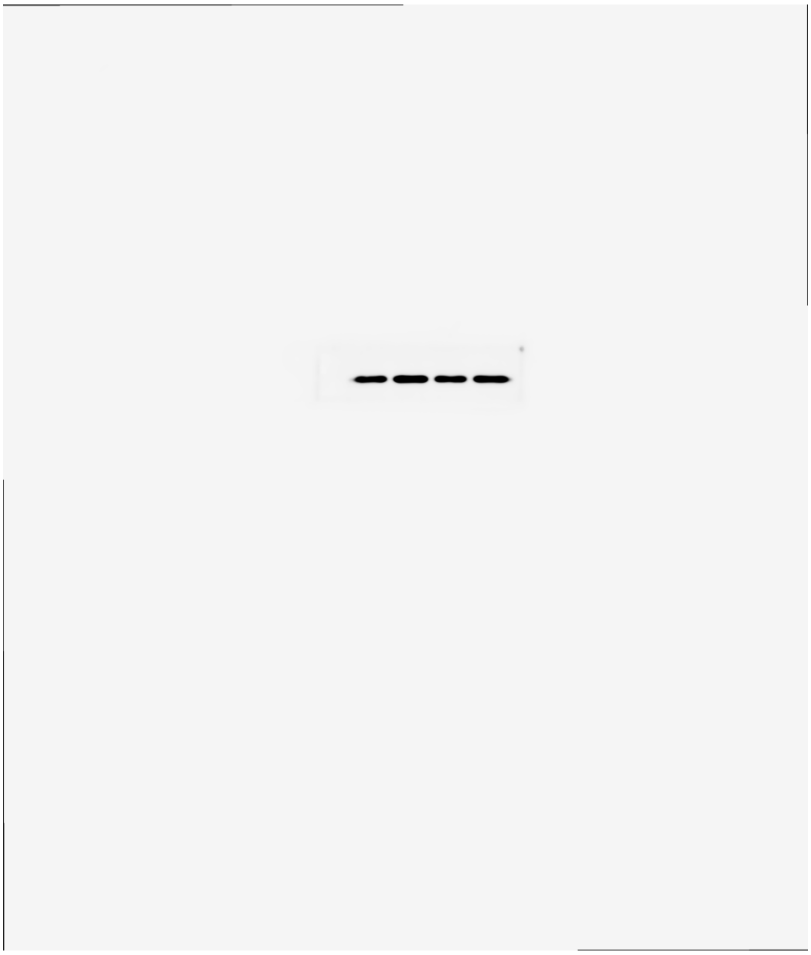

Full unedited gel for Figure 14B

PPFIA4 (from lane 1 to lane 4)

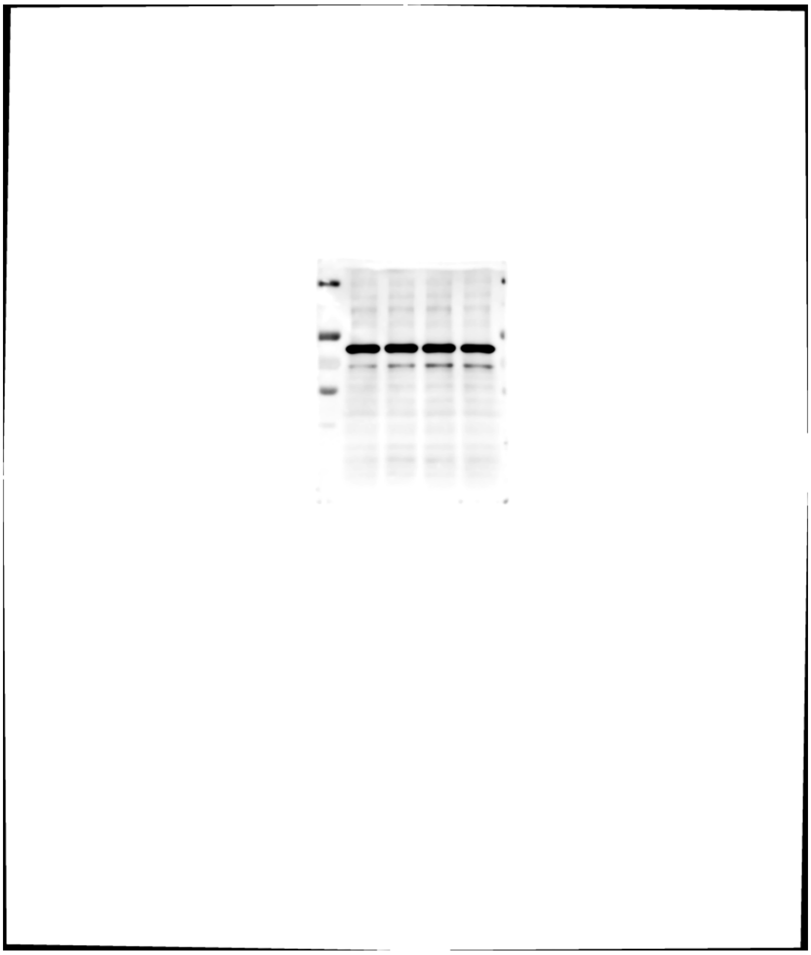

CASK (from lane 1 to lane 4)

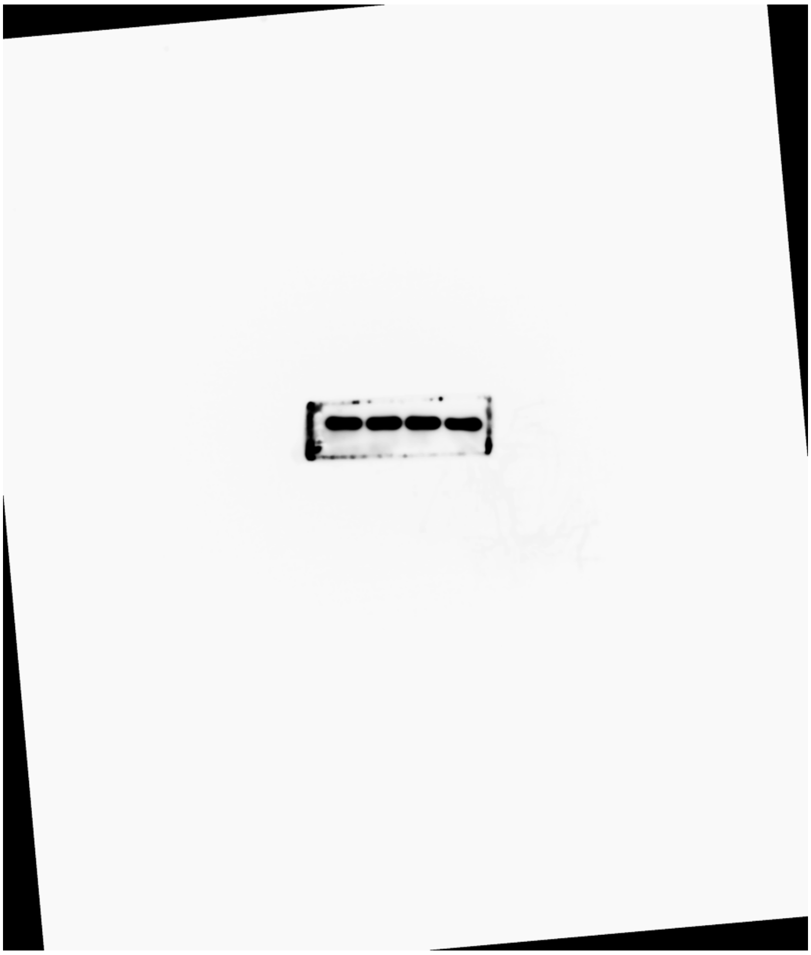

AKT1 (from lane 1 to lane 4)

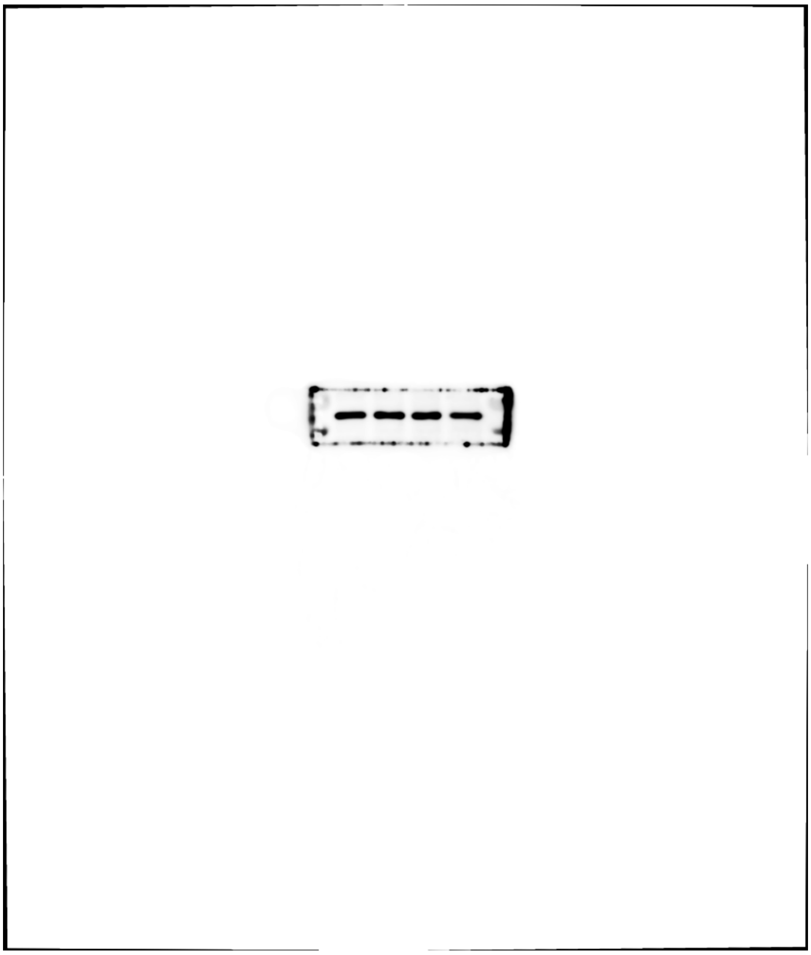

p-AKT1(Thr308) (from lane 1 to lane 4)

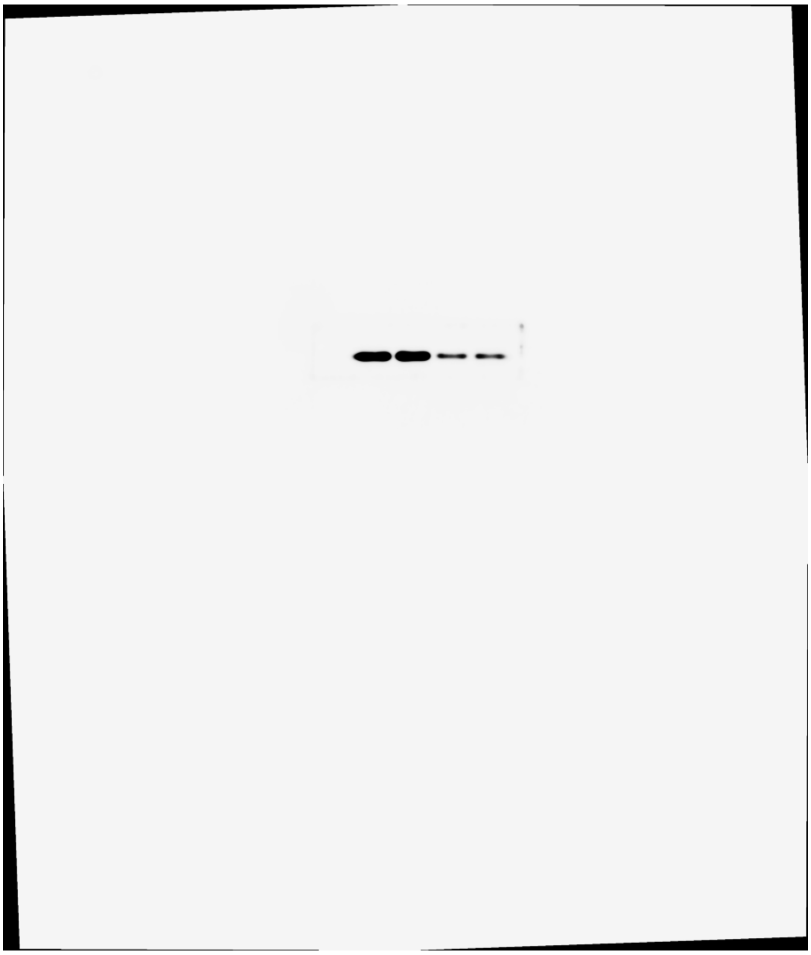

p-AKT1(Ser473) (from lane 1 to lane 4)

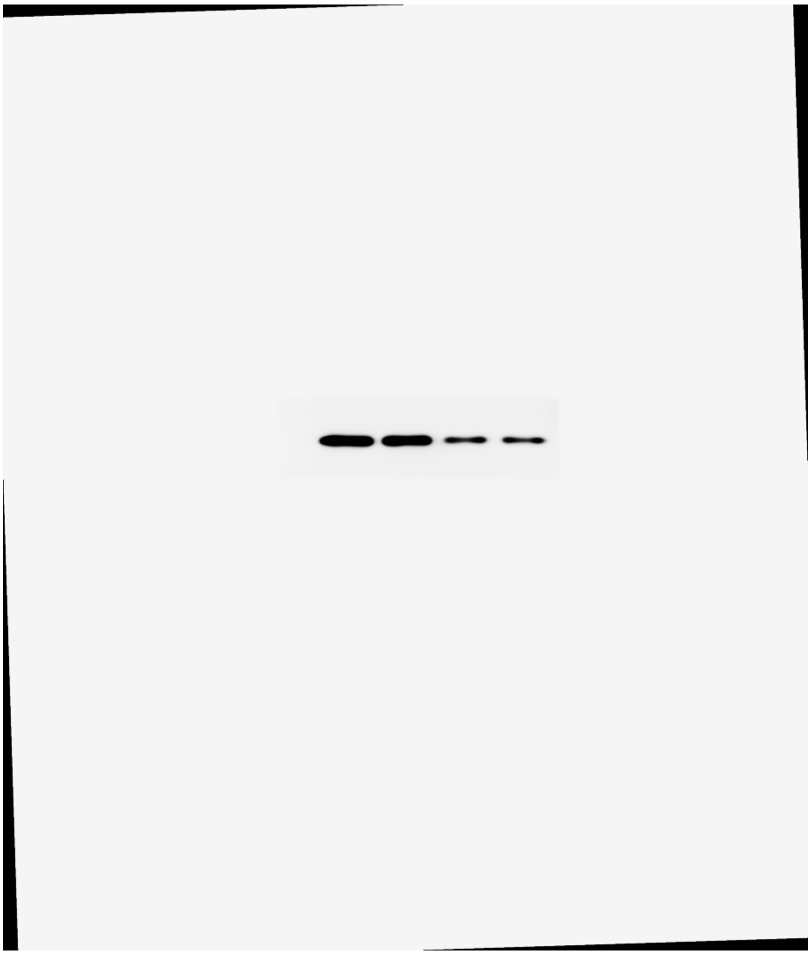

p65 (from lane 1 to lane 4)

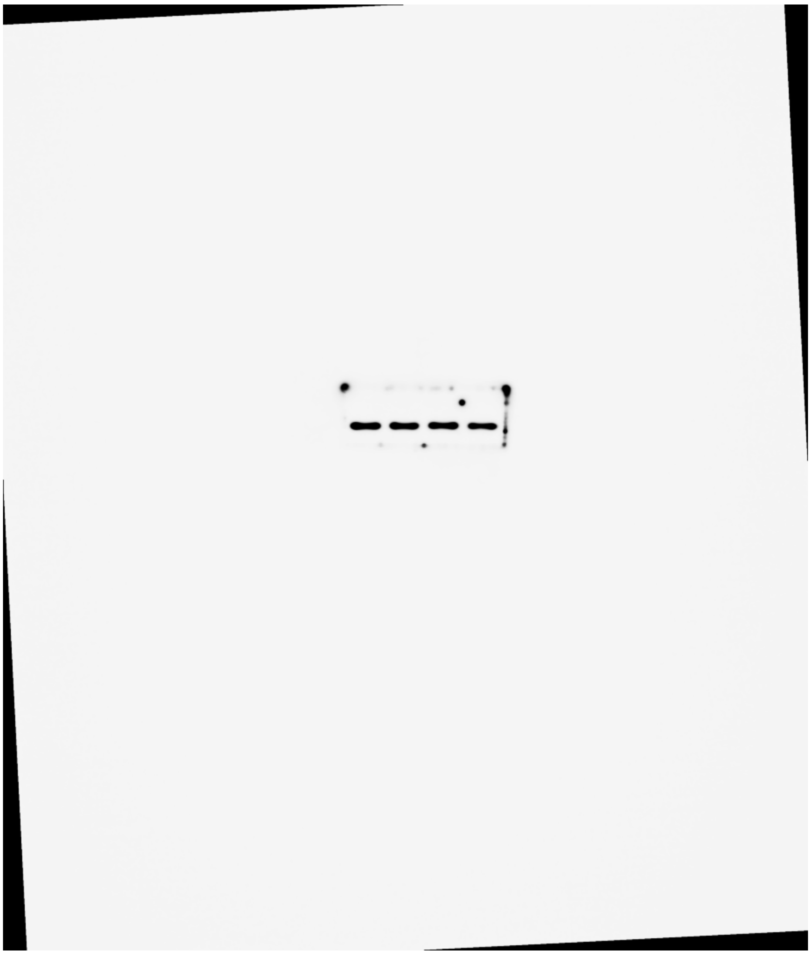

p-p65 (from lane 1 to lane 4)

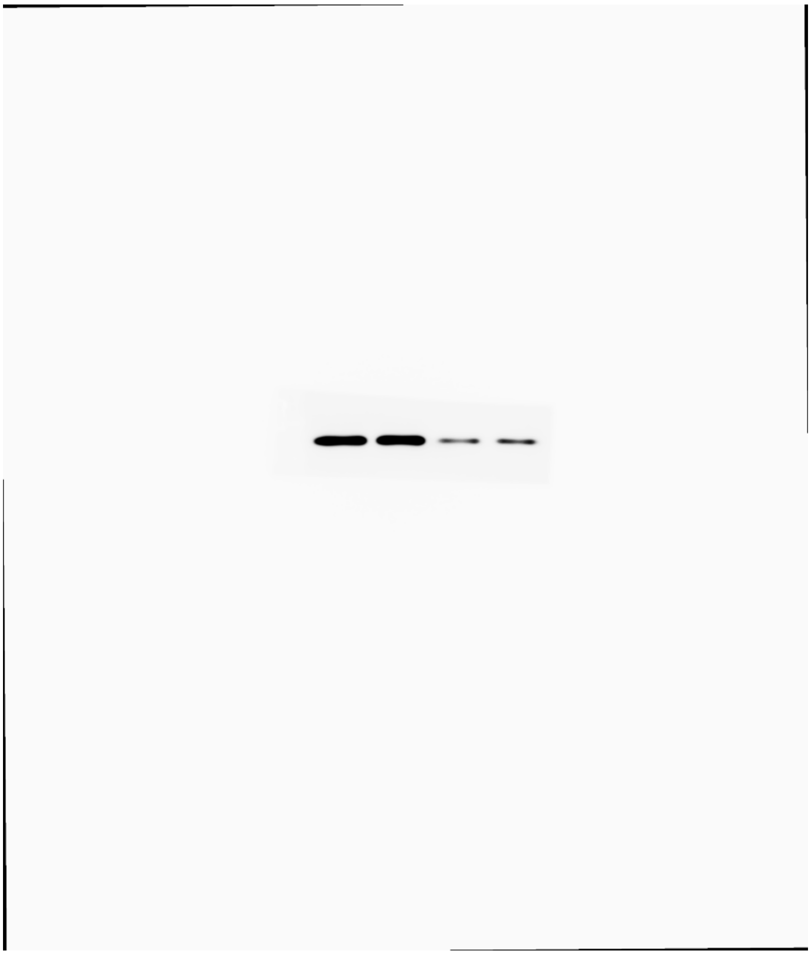

GAPDH (from lane 1 to lane 4)

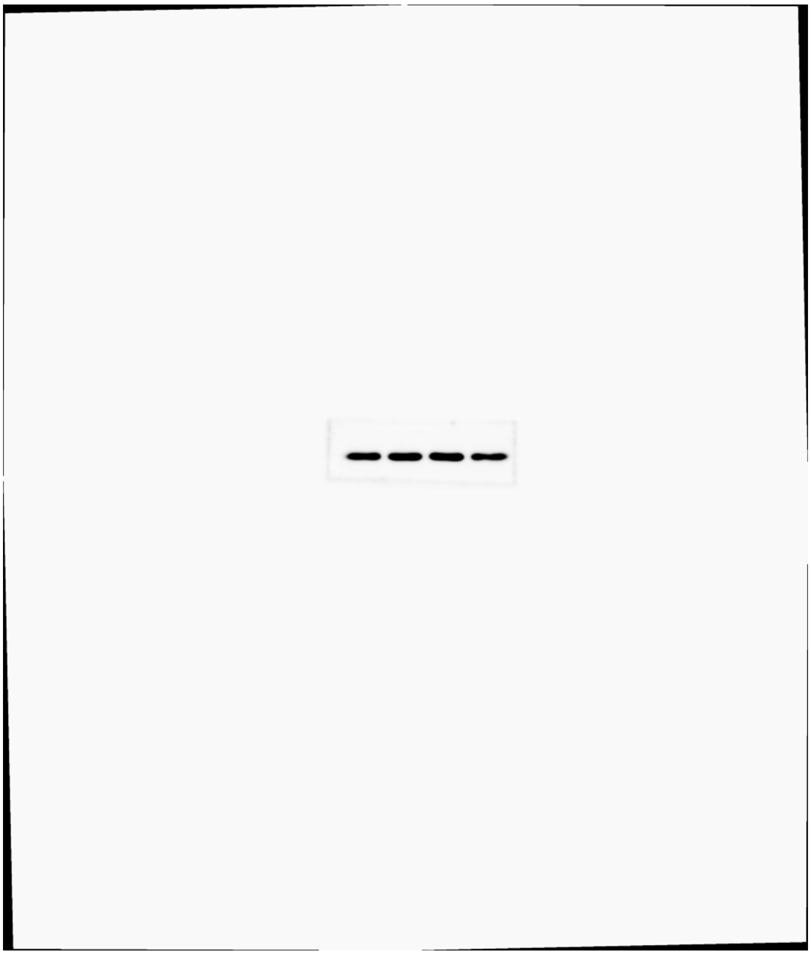

Full unedited gel for Figure 14C

PPFIA4 (from lane 1 to lane 6)

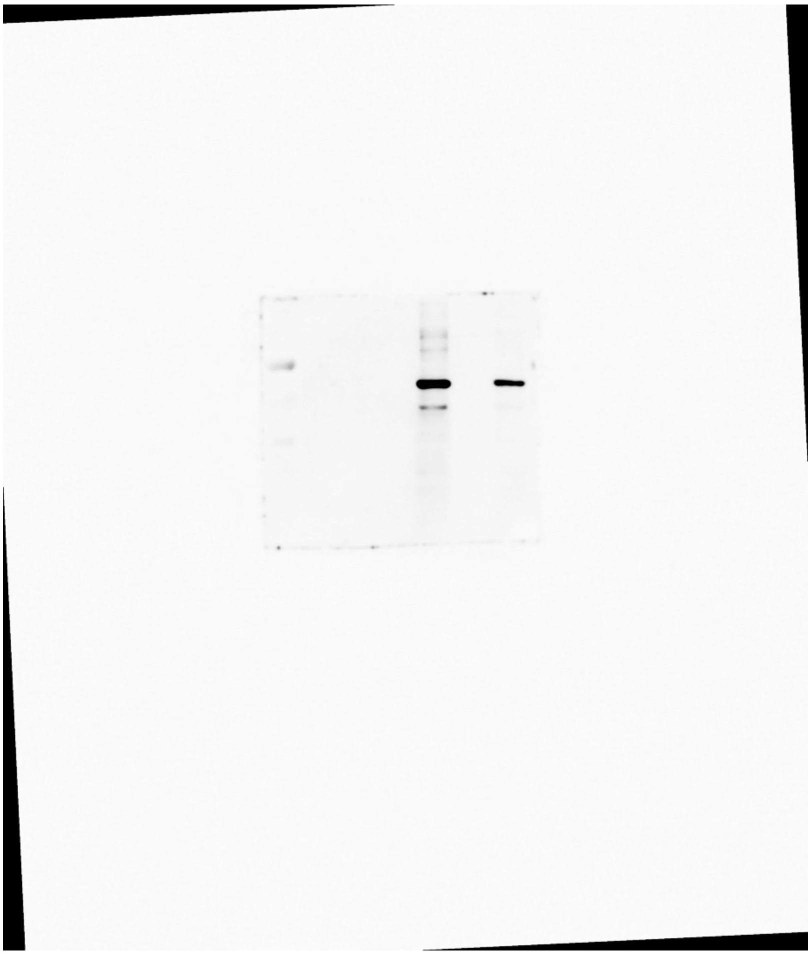

CASK (from lane 1 to lane 6)

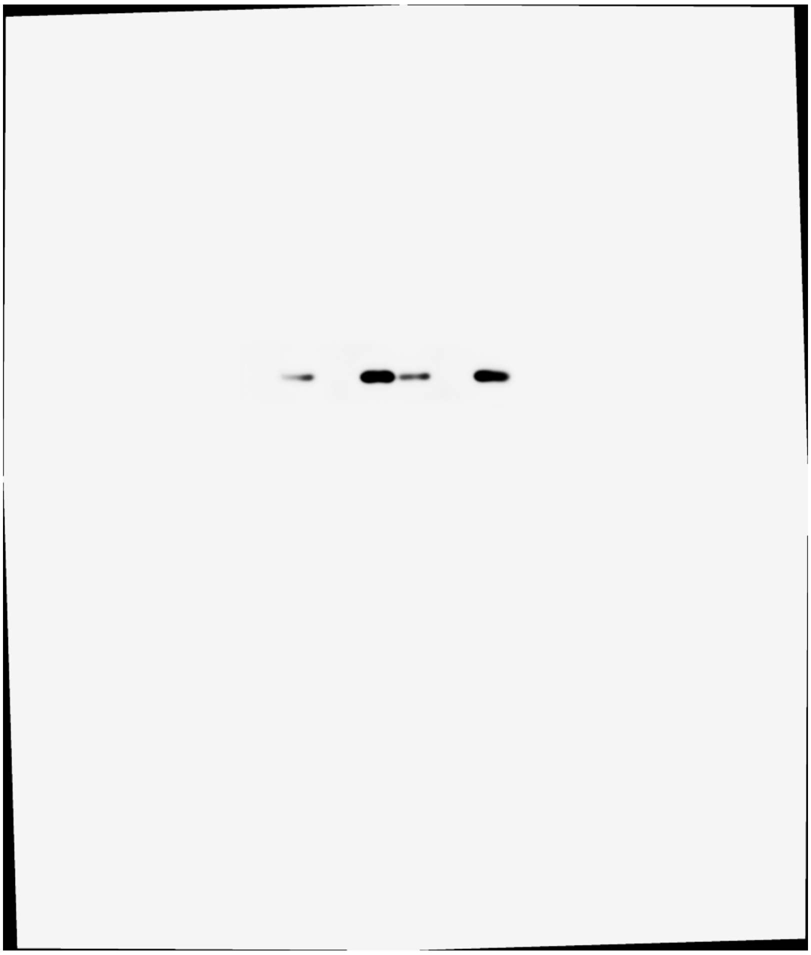

AKT1 (from lane 1 to lane 6)

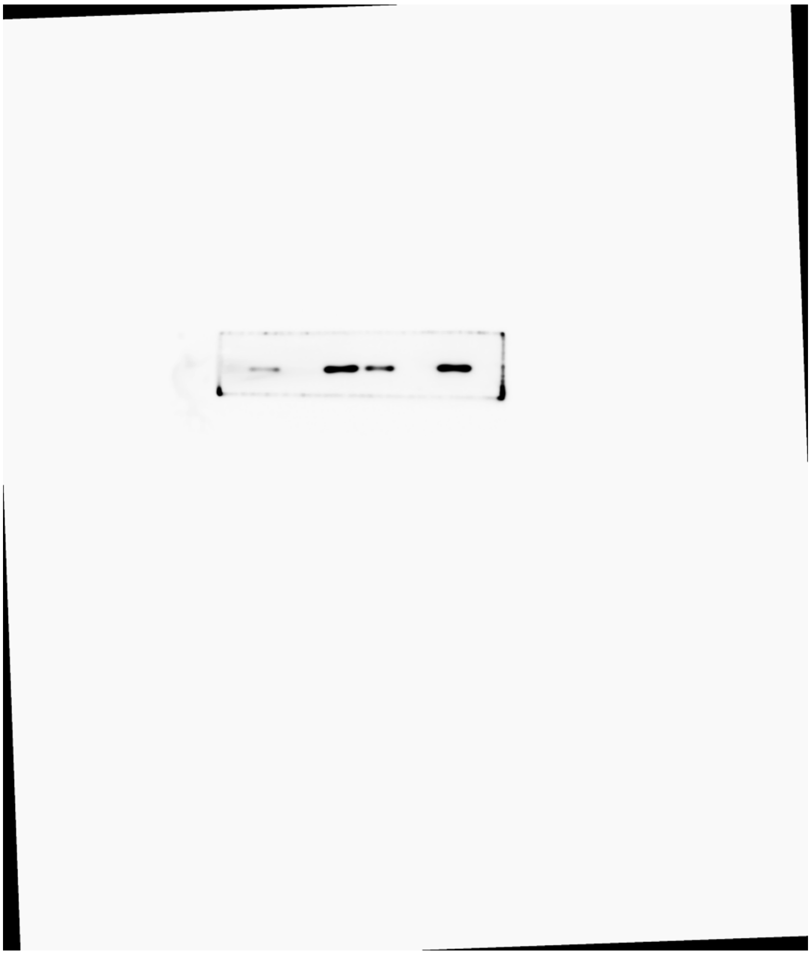

p-AKT1(Thr308) (from lane 1 to lane 6)

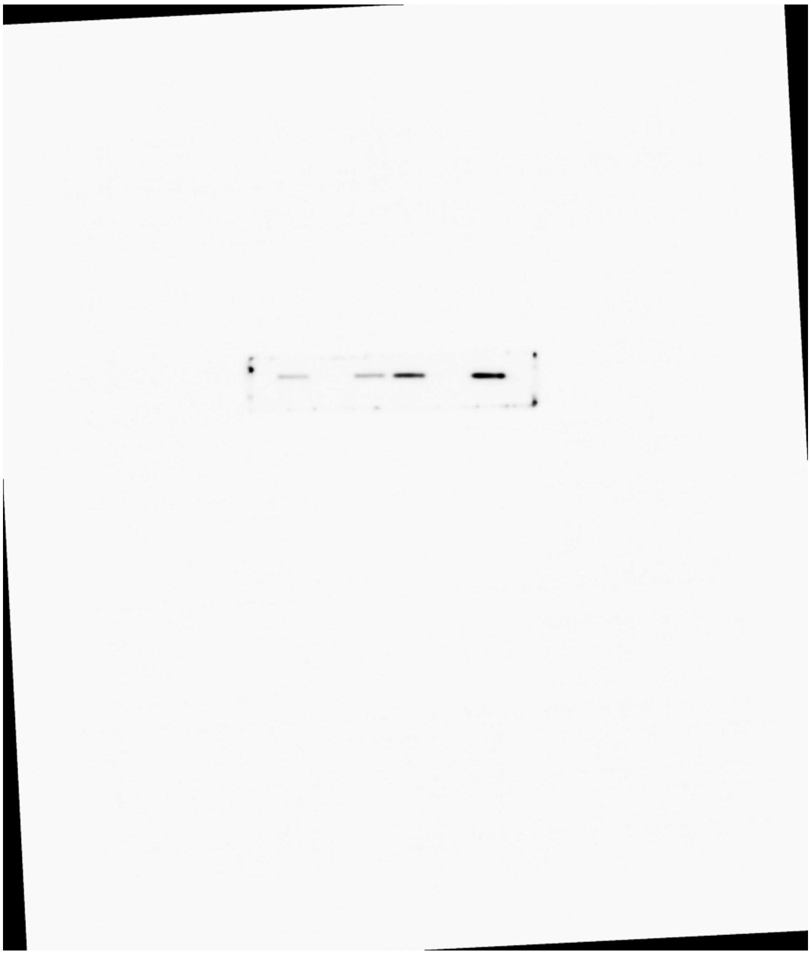

p-AKT1(Ser473) (from lane 1 to lane 6)

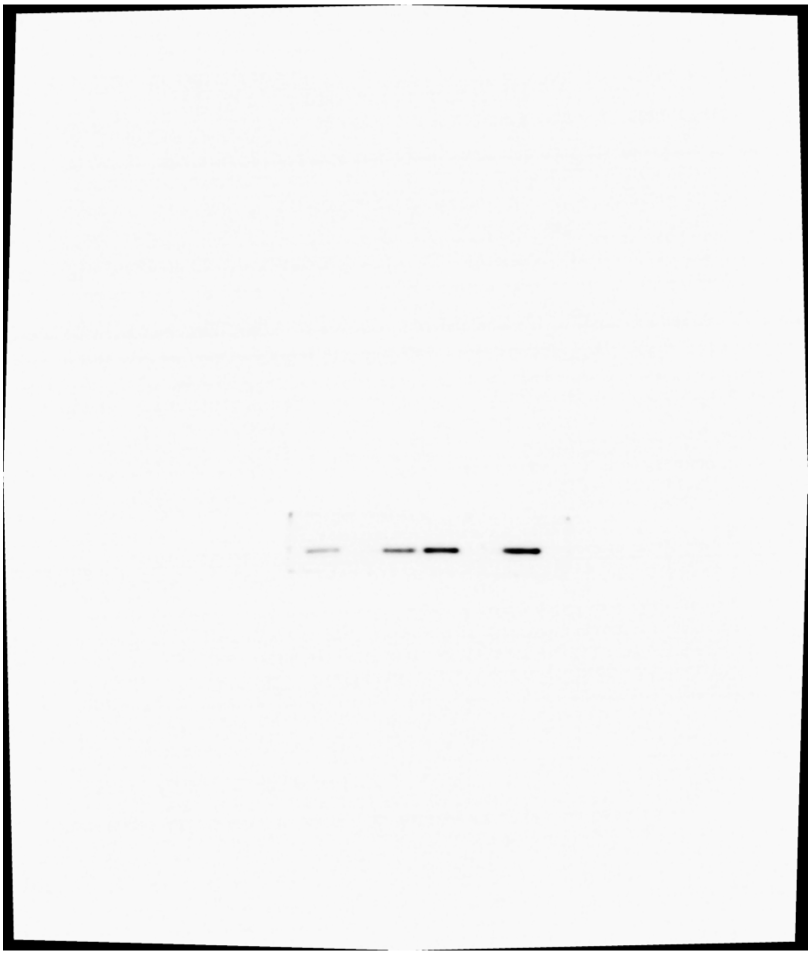

GAPDH (from lane 1 to lane 6)

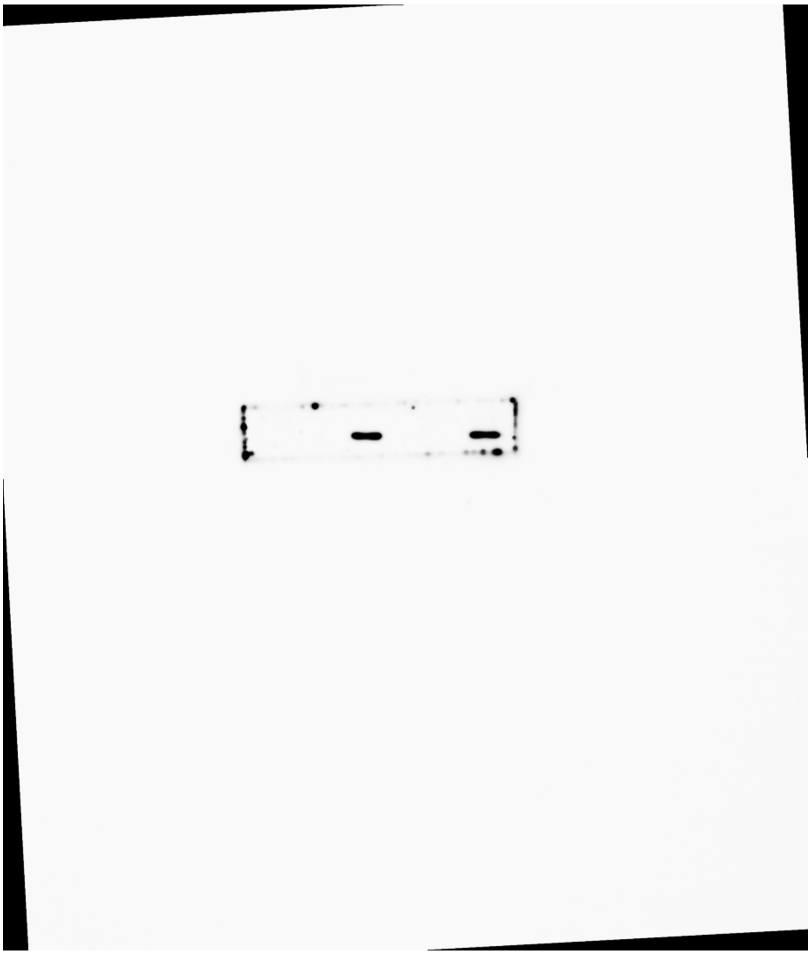

Full unedited gel for Figure 14D

PPFIA4 (from lane 1 to lane 6)

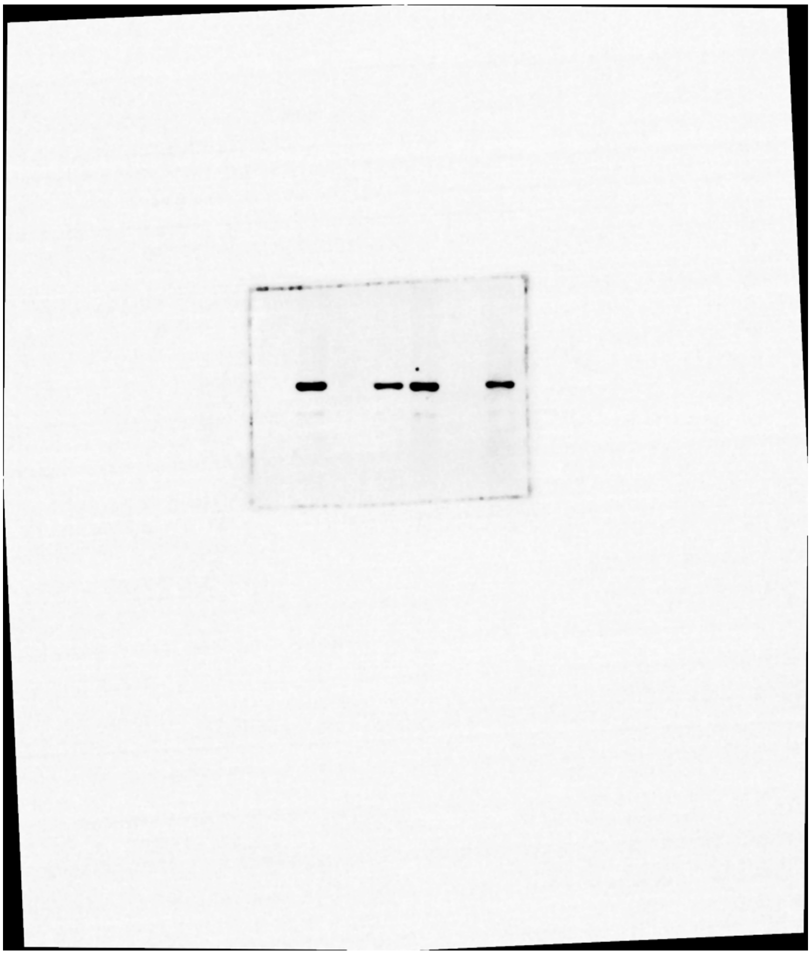

CASK (from lane 1 to lane 6)

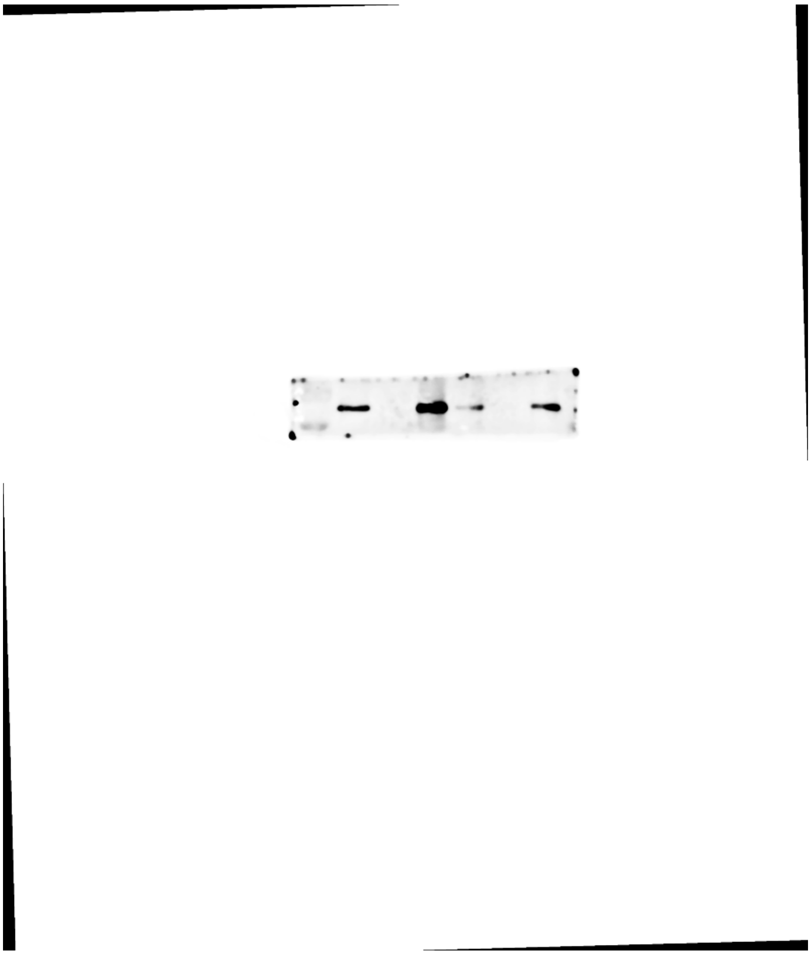

AKT1 (from lane 1 to lane 6)

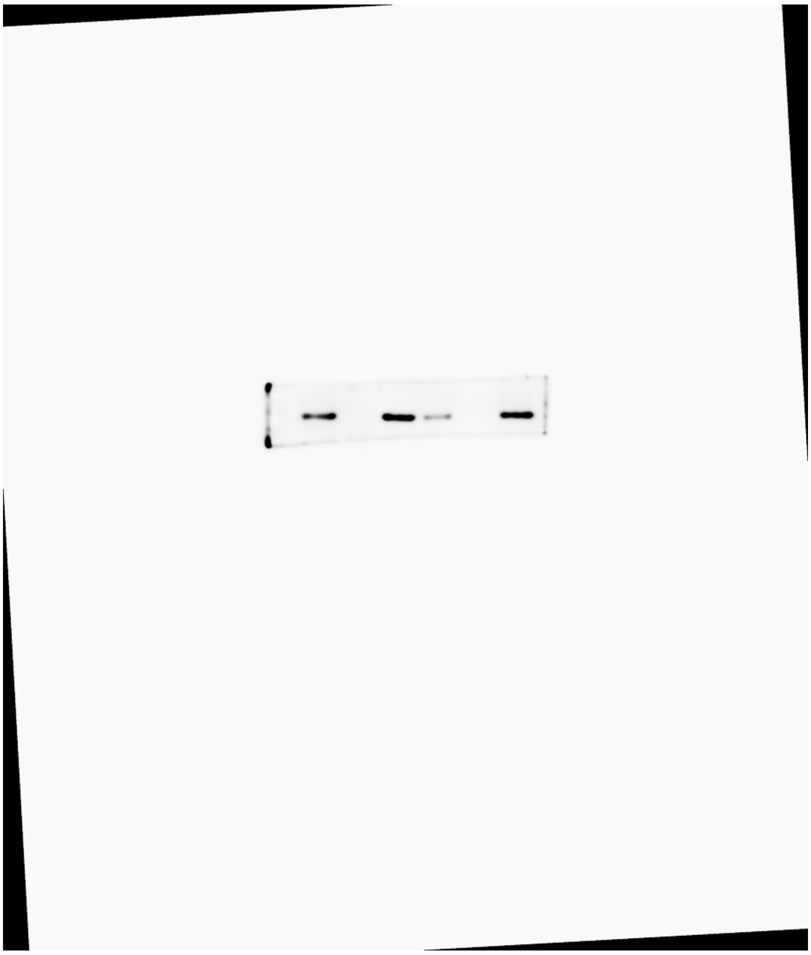

p-AKT1(Thr308) (from lane 1 to lane 6)

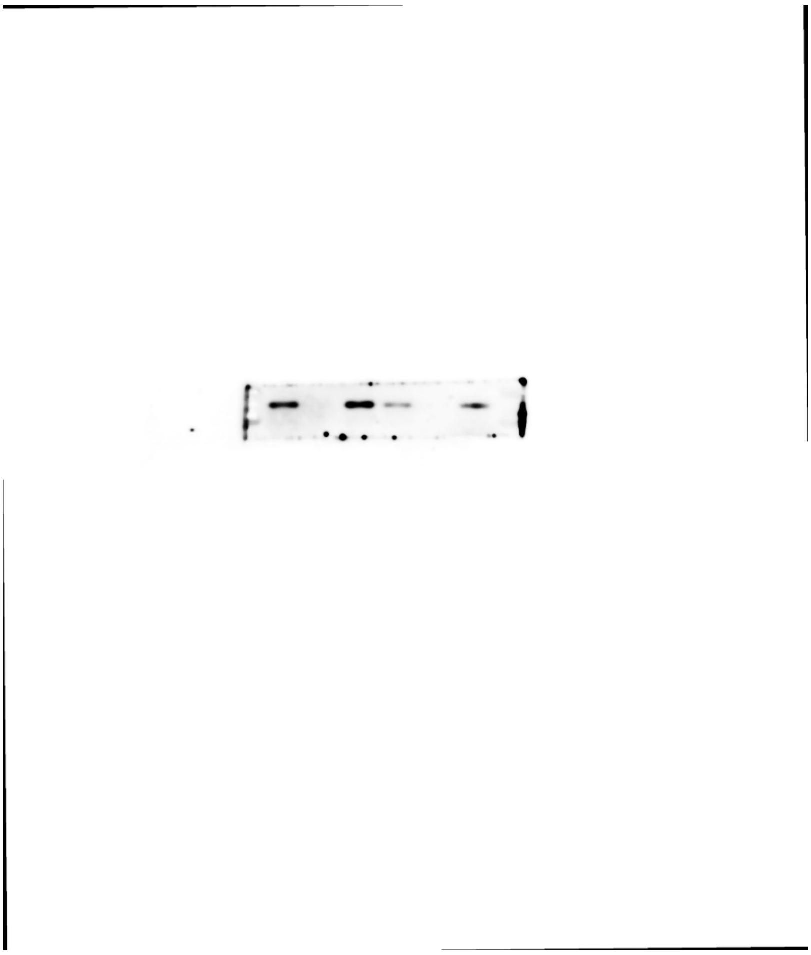

p-AKT1(Ser473) (from lane 1 to lane 6)

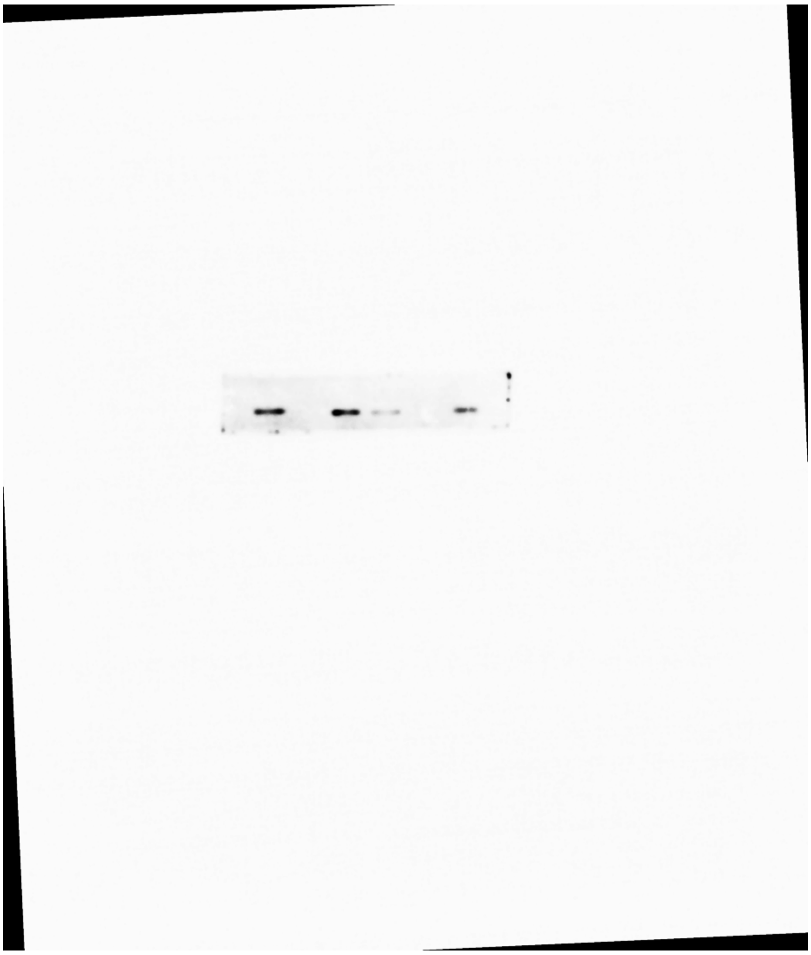

GAPDH (from lane 1 to lane 6)

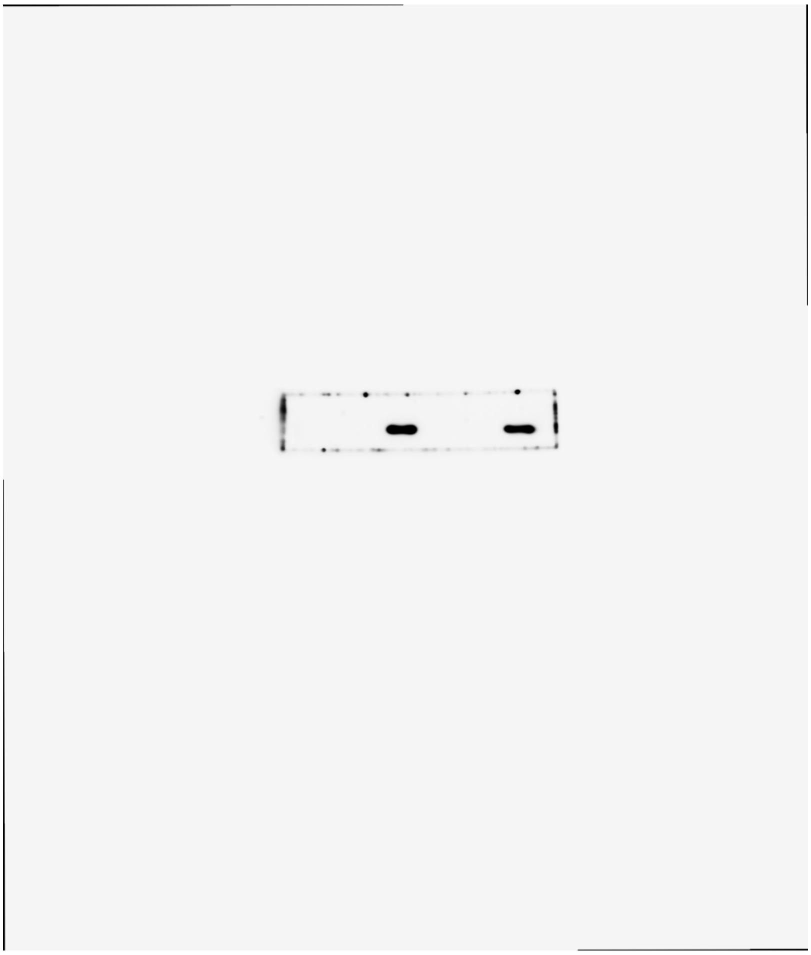

Full unedited gel for Figure 14E
PPFIA4 (from lane 1 to lane 6)

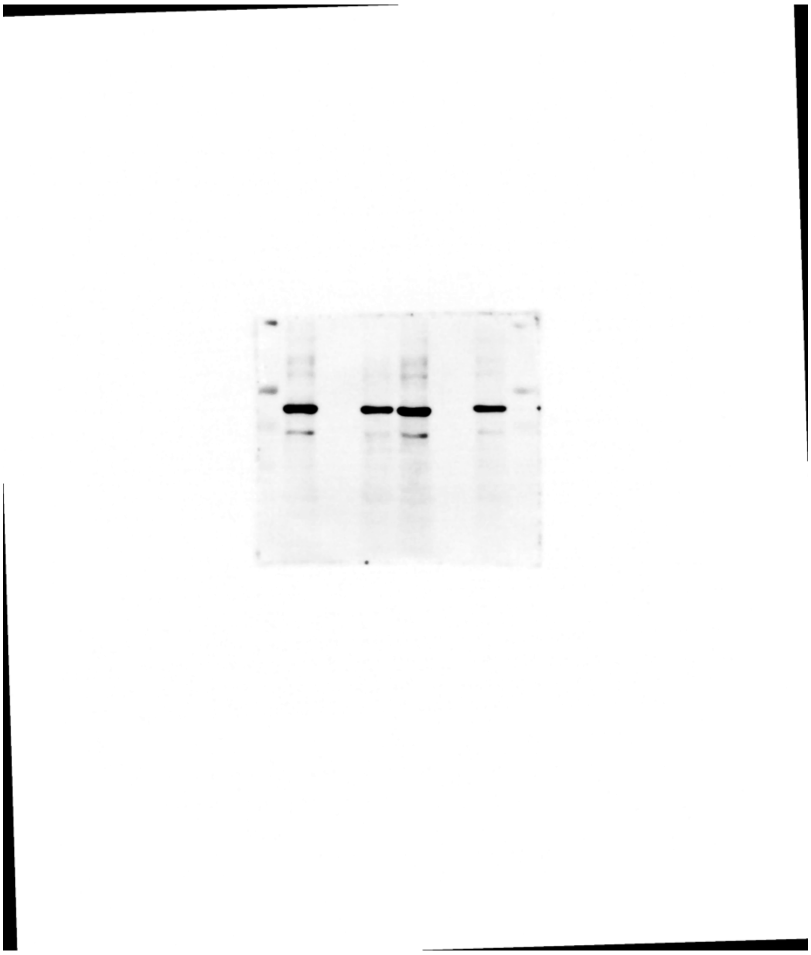

CASK (from lane 1 to lane 6)

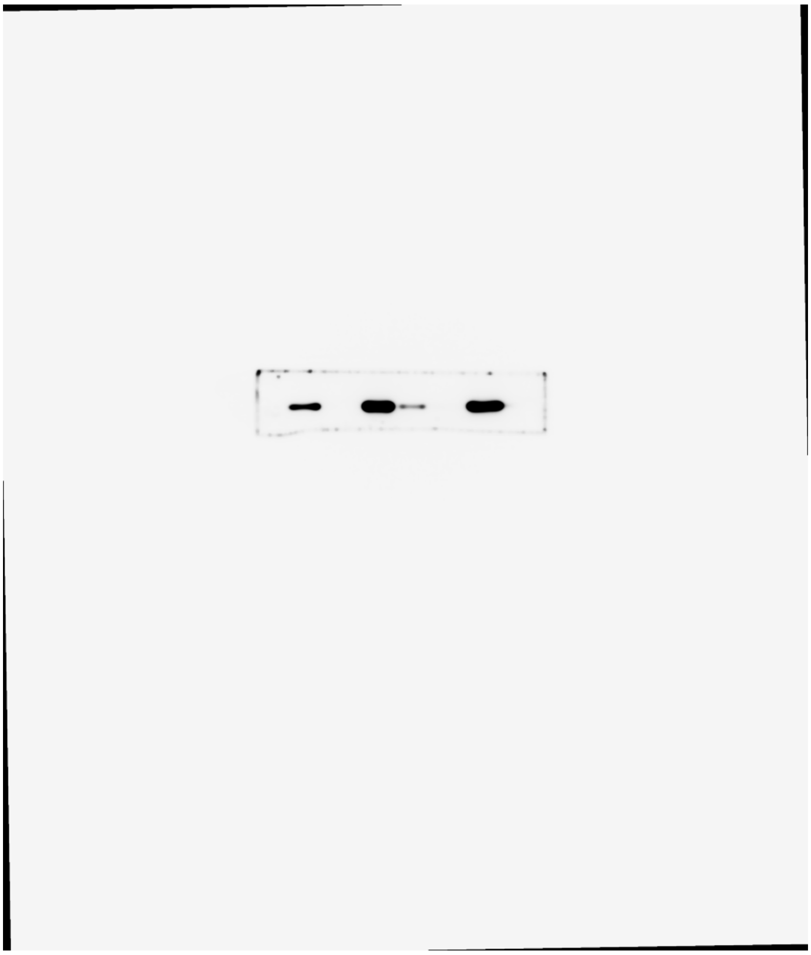

AKT1 (from lane 1 to lane 6)

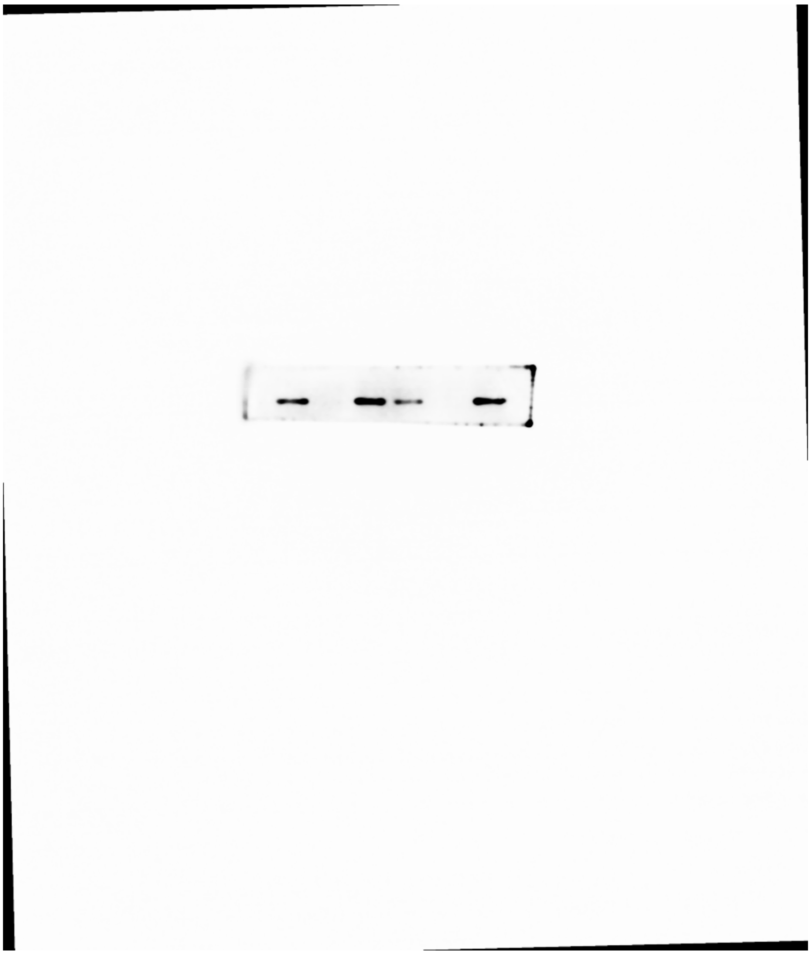

p-AKT1(Thr308) (from lane 1 to lane 6)

p-AKT1(Ser473) (from lane 1 to lane 6)

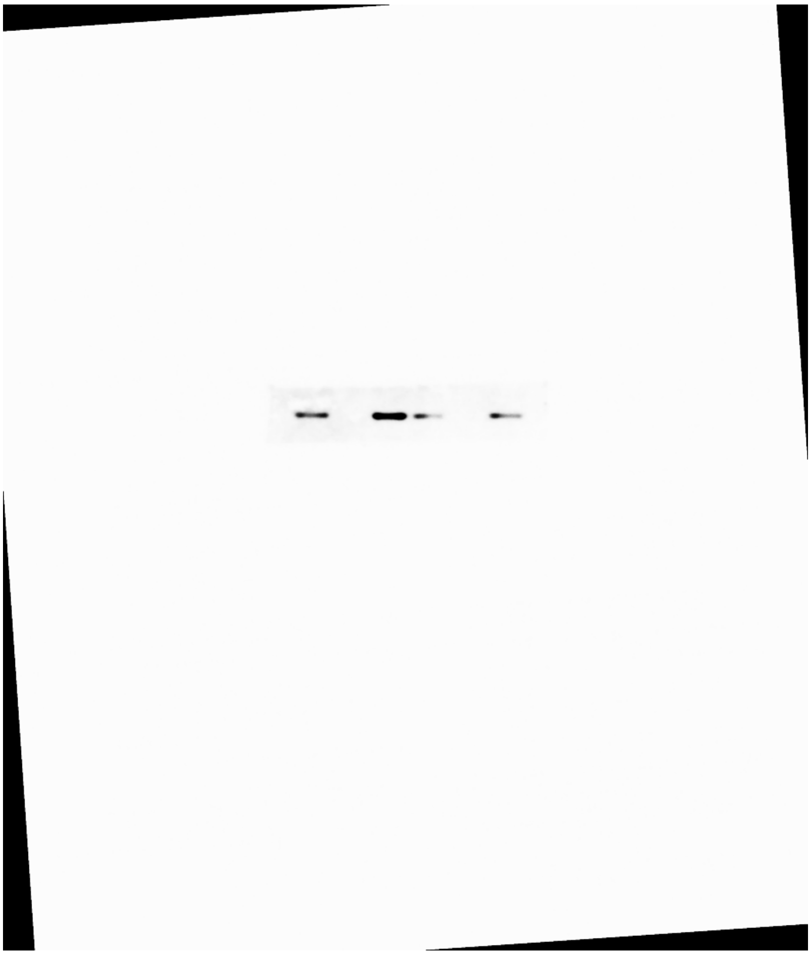

GAPDH (from lane 1 to lane 6)

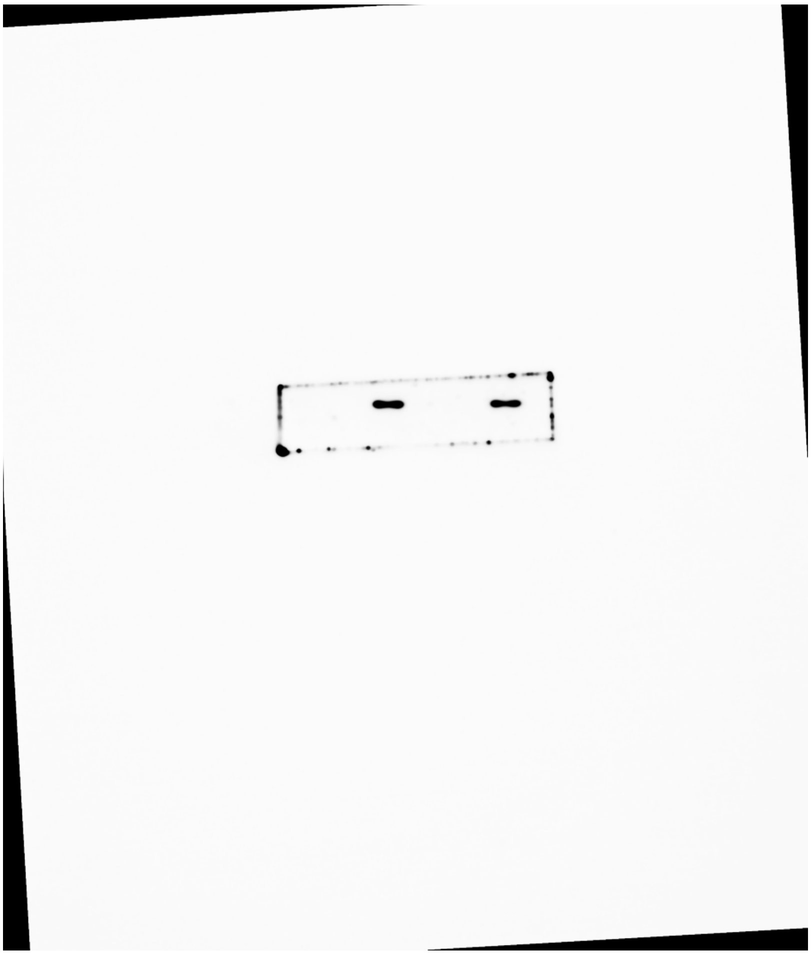

Full unedited gel for Figure 15C

PPFIA4 (from lane 1 to lane 4)

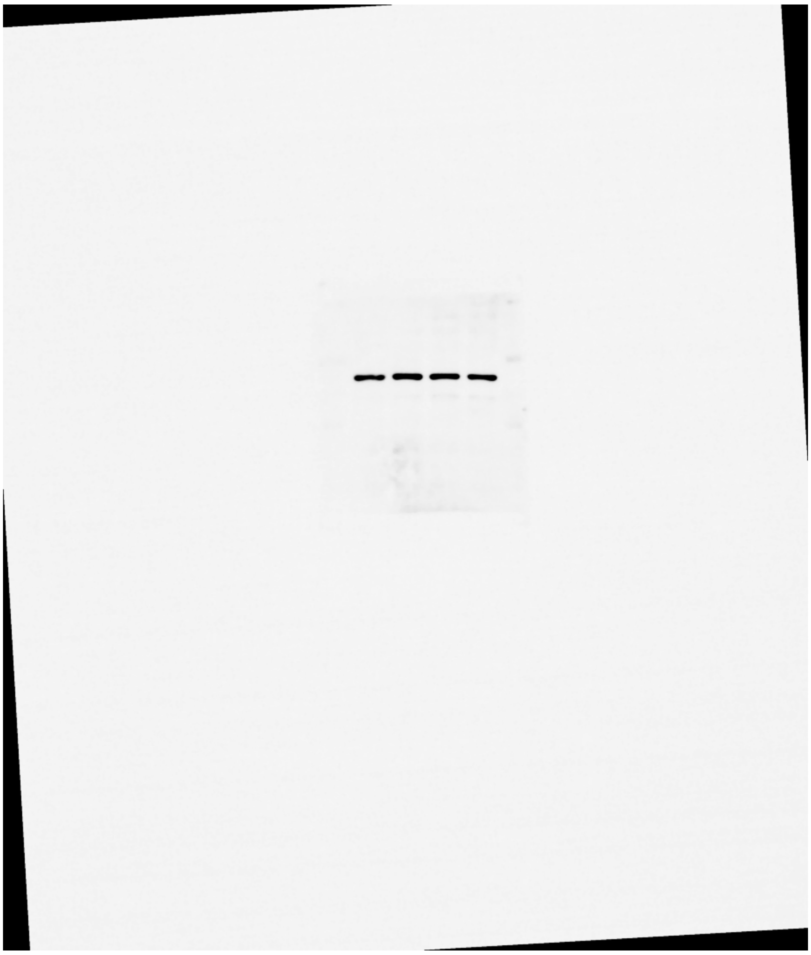

CASK (from lane 1 to lane 4)

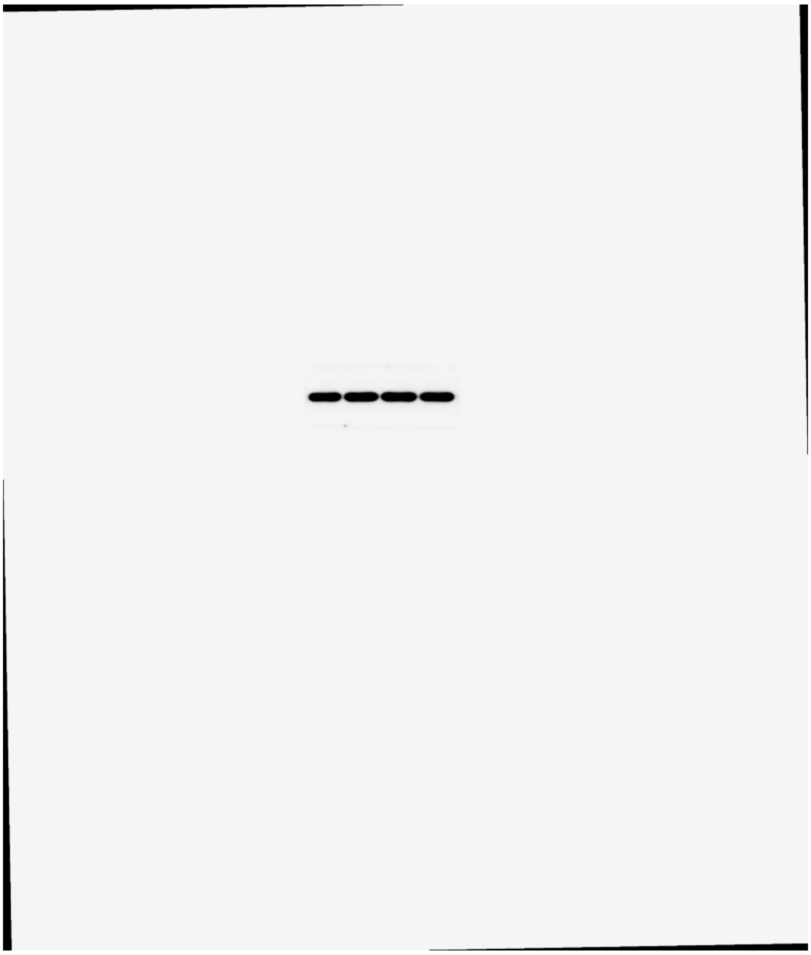

AKT1 (from lane 1 to lane 4)

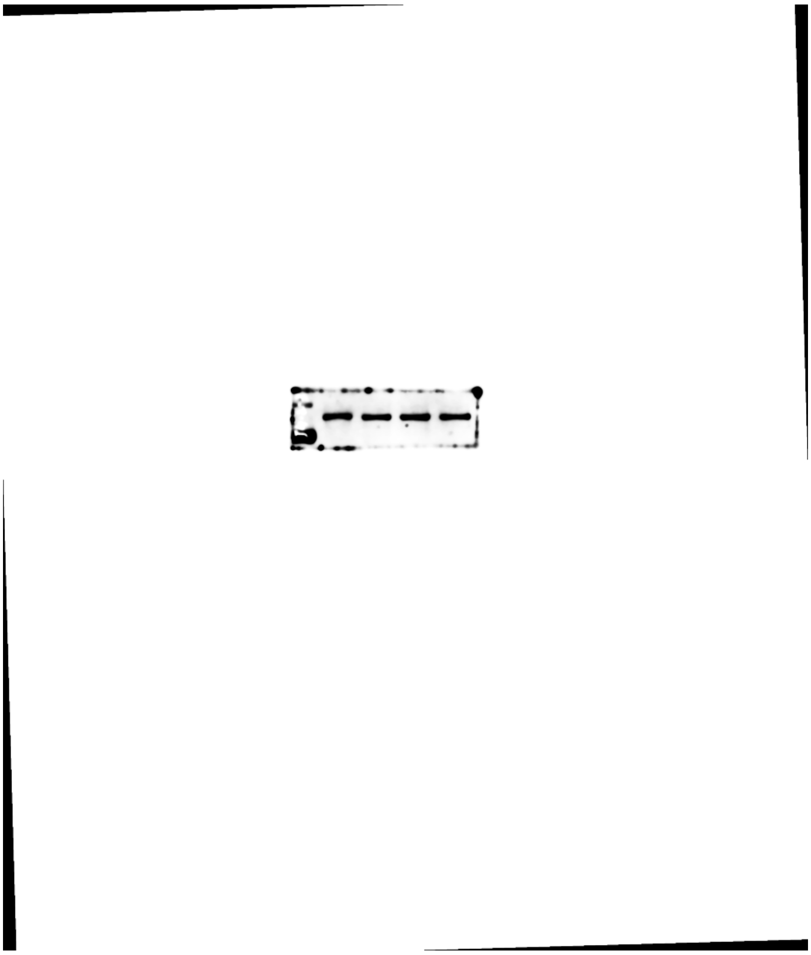

p-AKT1(Thr308) (from lane 1 to lane 4)

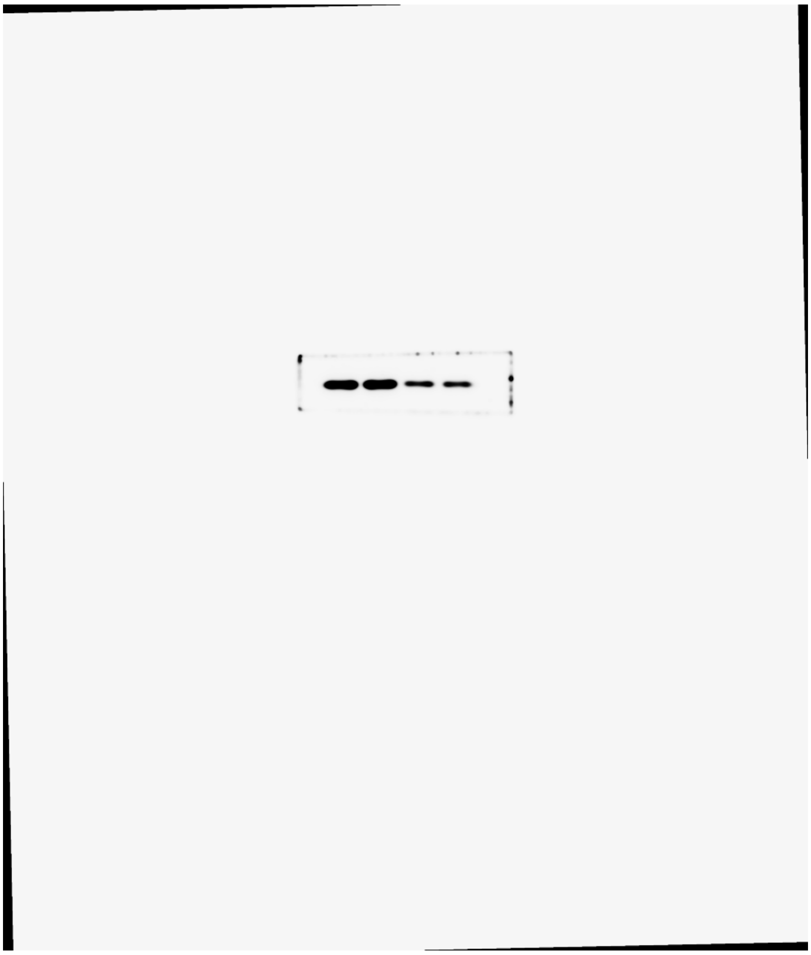

p-AKT1(Ser473) (from lane 1 to lane 4)

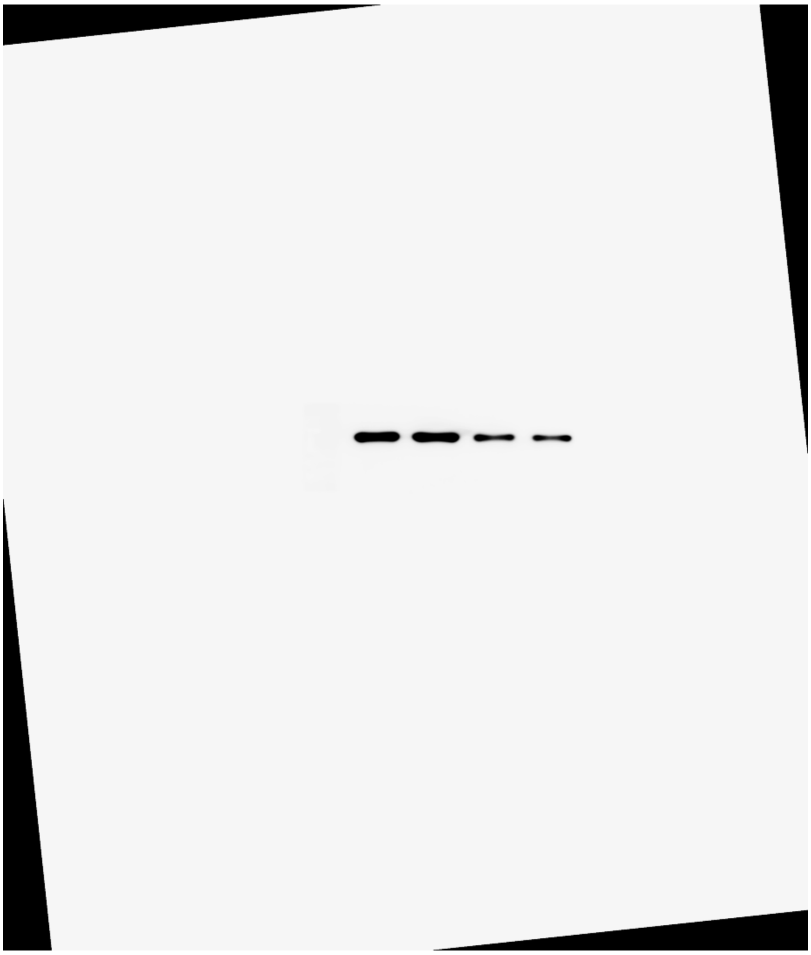

p65 (from lane 1 to lane 4)

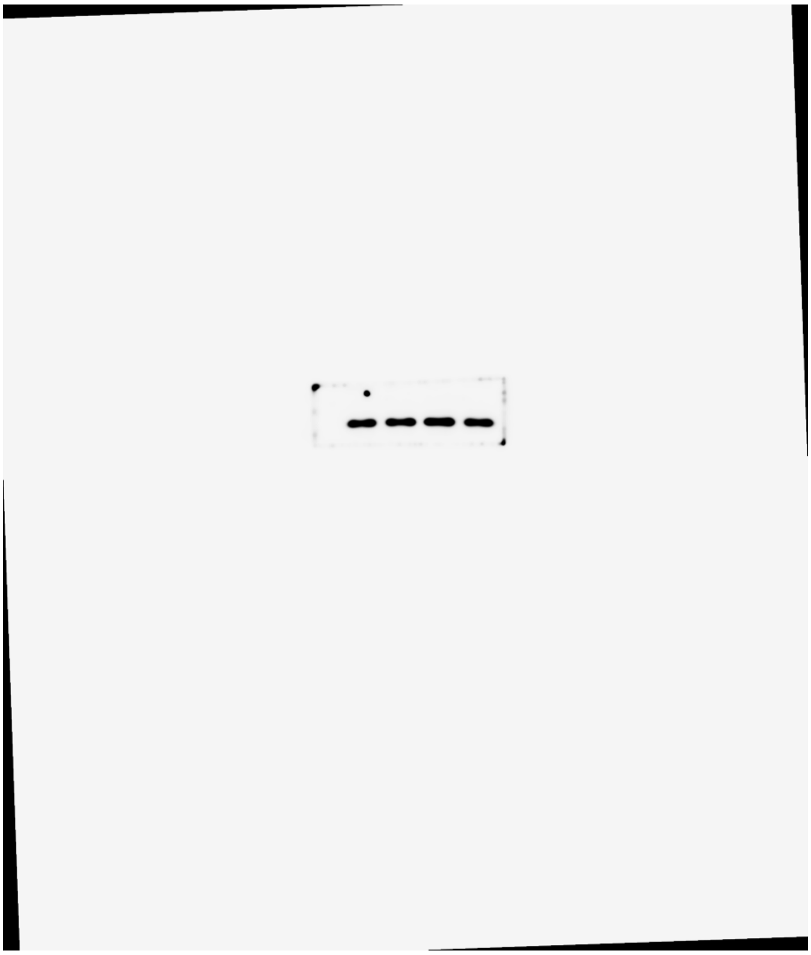

p-p65 (from lane 1 to lane 4)

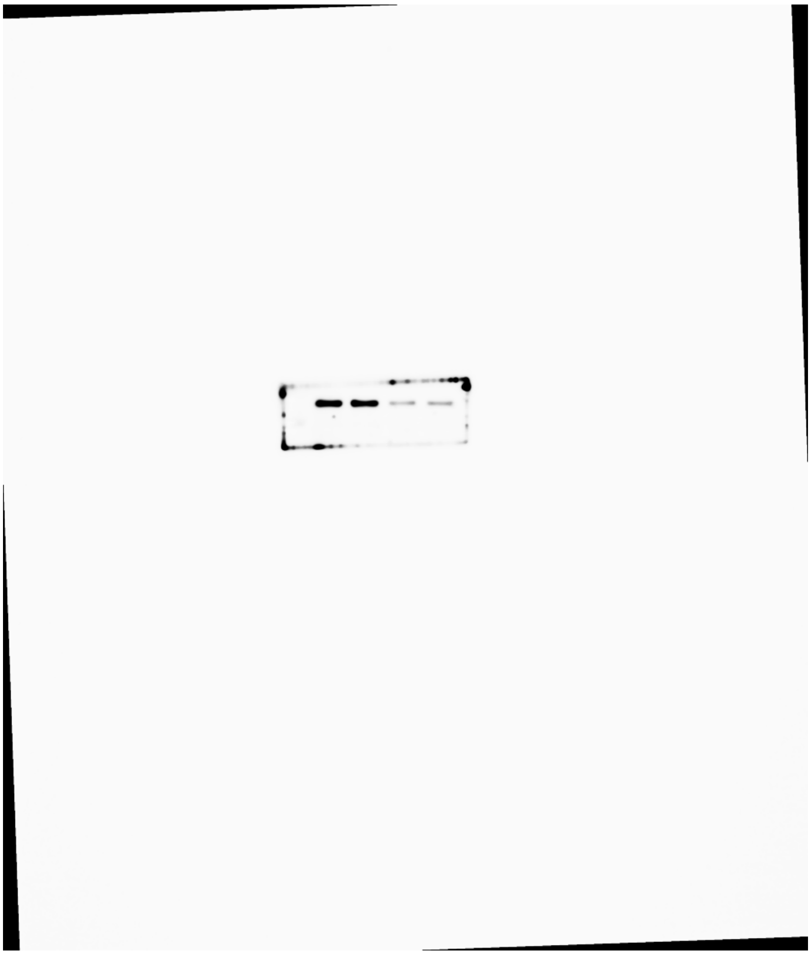

MMP1 (from lane 1 to lane 4)

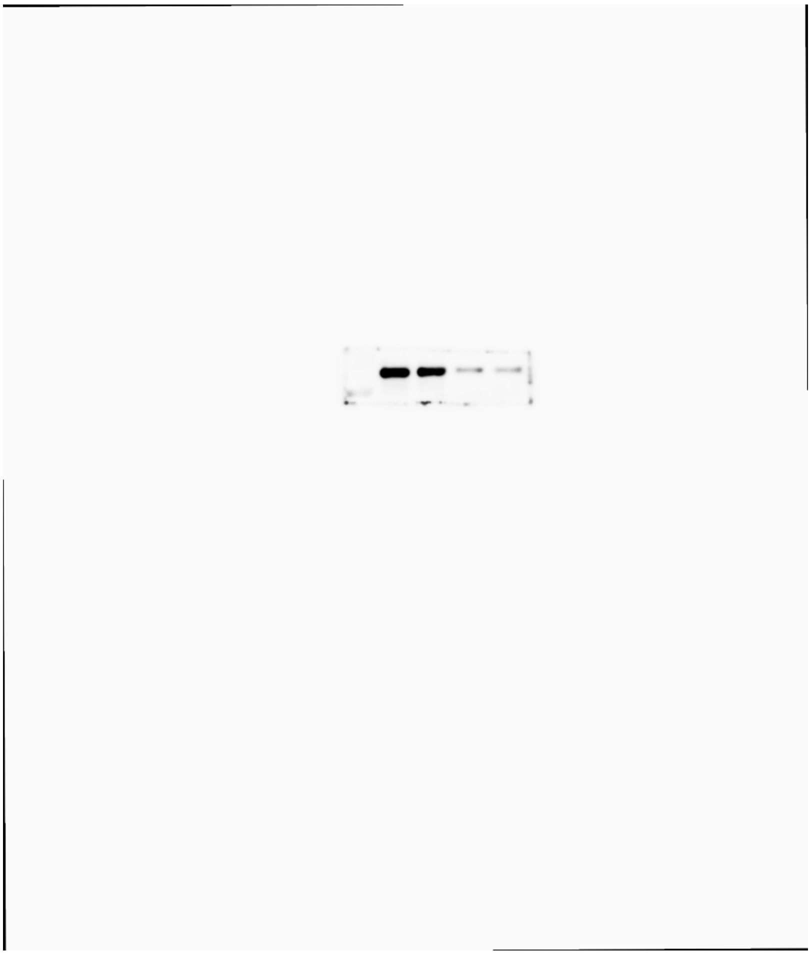

GAPDH (from lane 1 to lane 4)

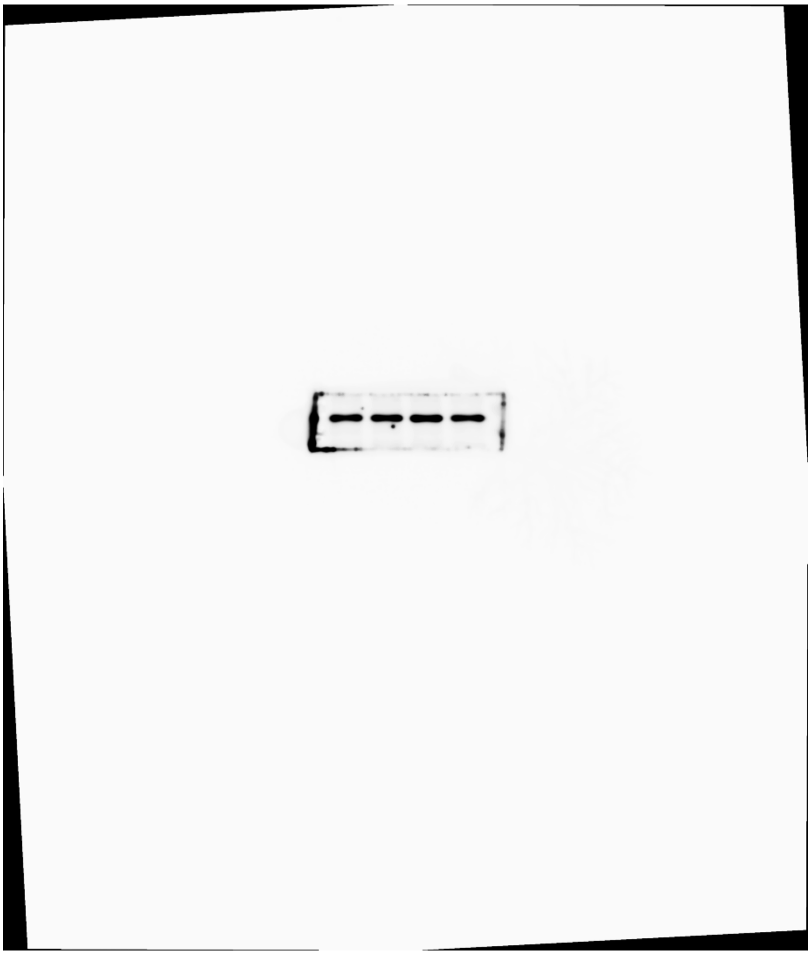

Full unedited gel for Figure 15D
PPFIA4 (from lane 1 to lane 4)

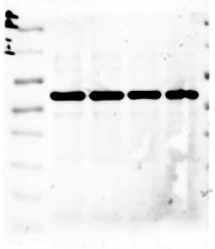

CASK (from lane 1 to lane 4)

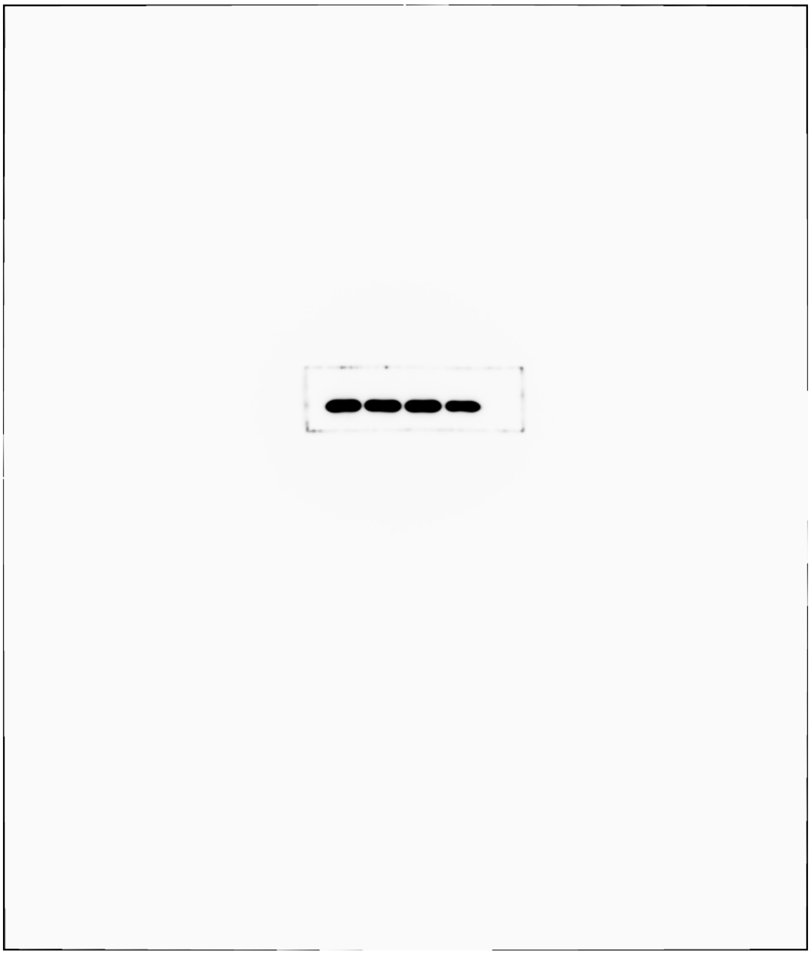

AKT1 (from lane 1 to lane 4)

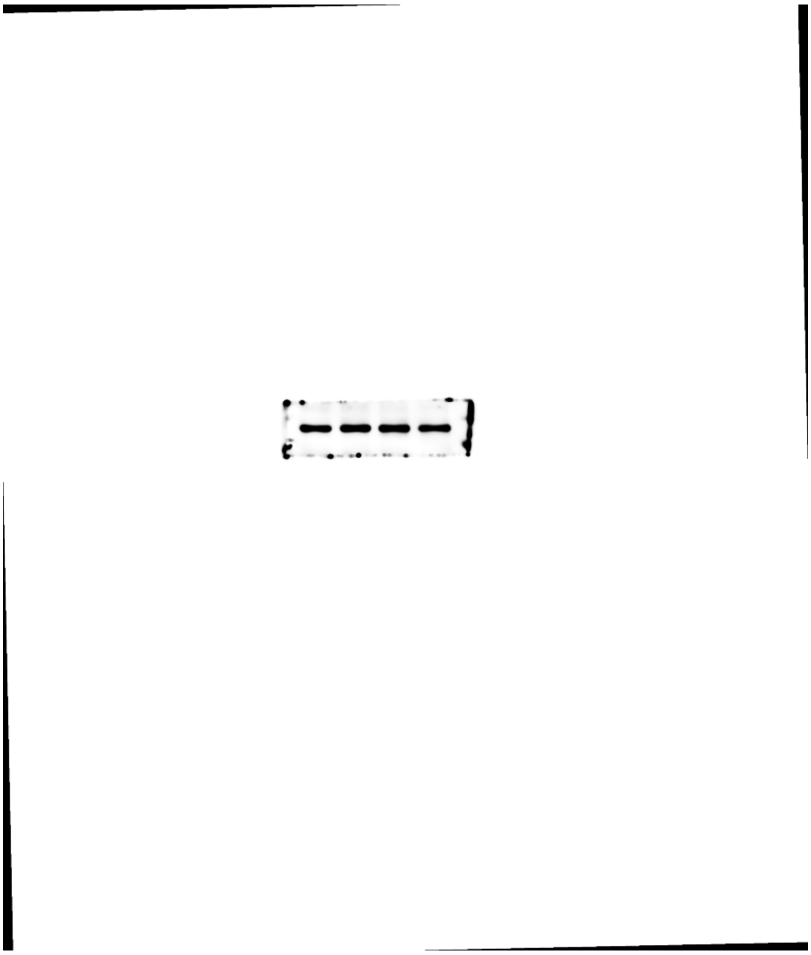

p-AKT1(Thr308) (from lane 1 to lane 4)

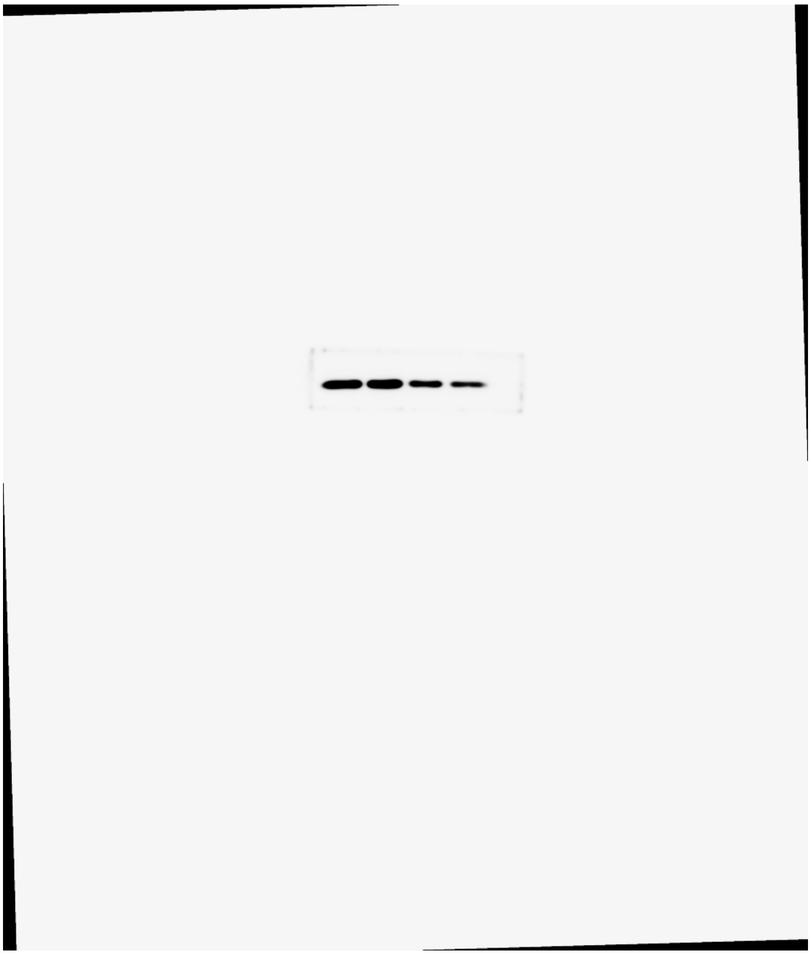

p-AKT1(Ser473) (from lane 1 to lane 4)

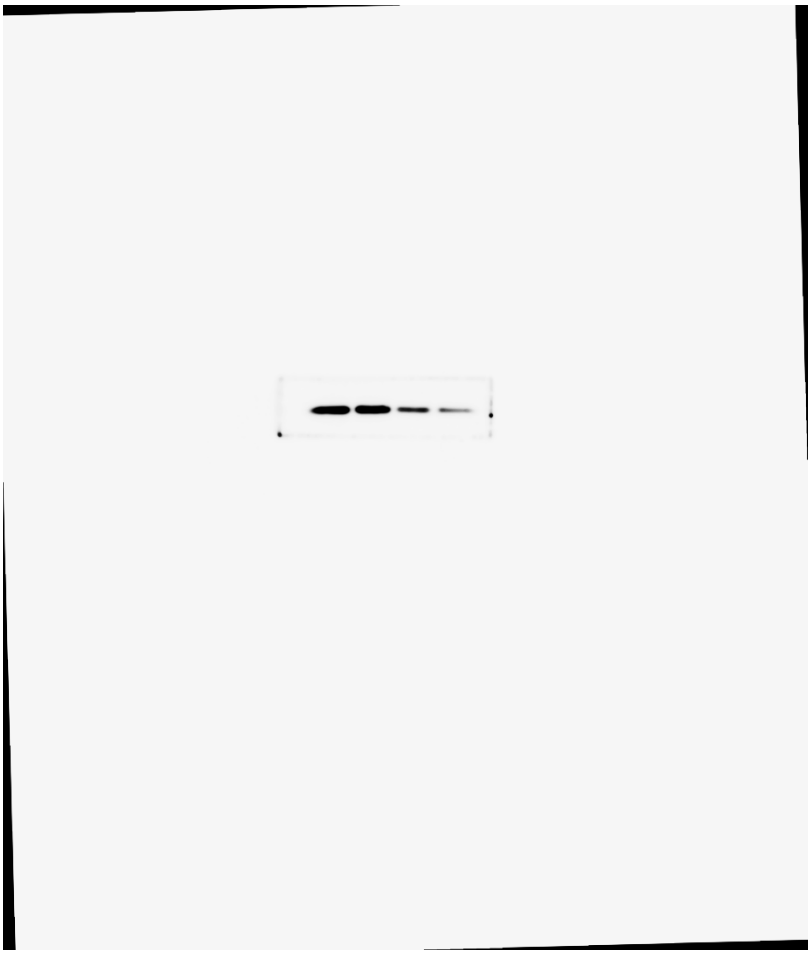

p65 (from lane 1 to lane 4)

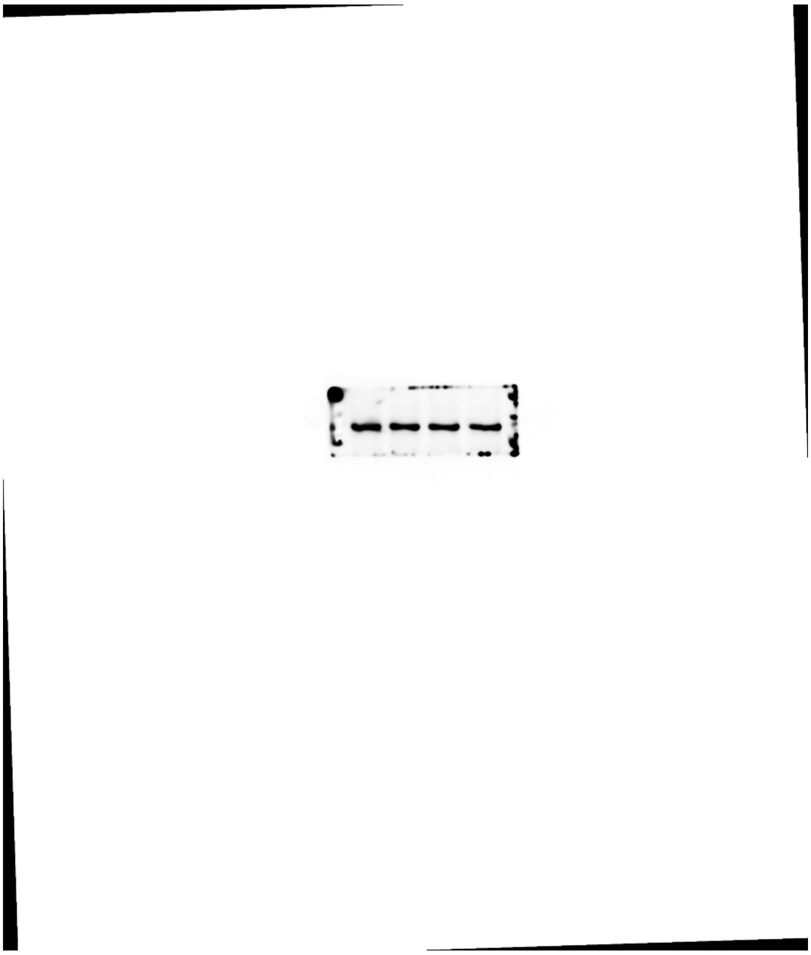

p-p65 (from lane 1 to lane 4)

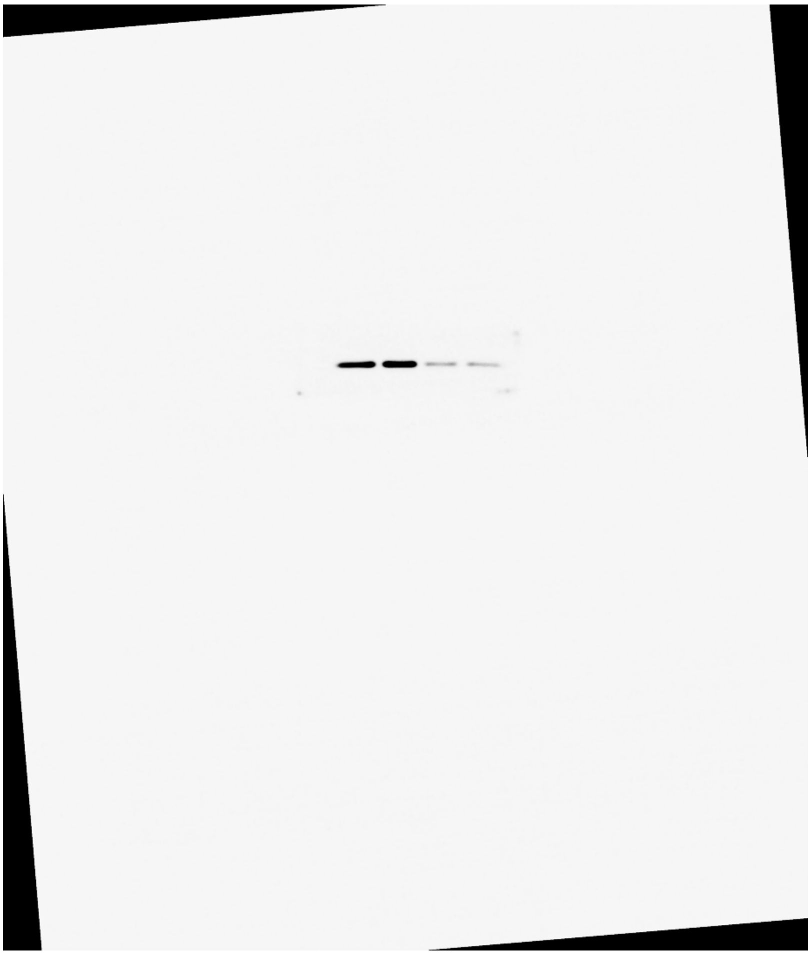

MMP1 (from lane 1 to lane 4)

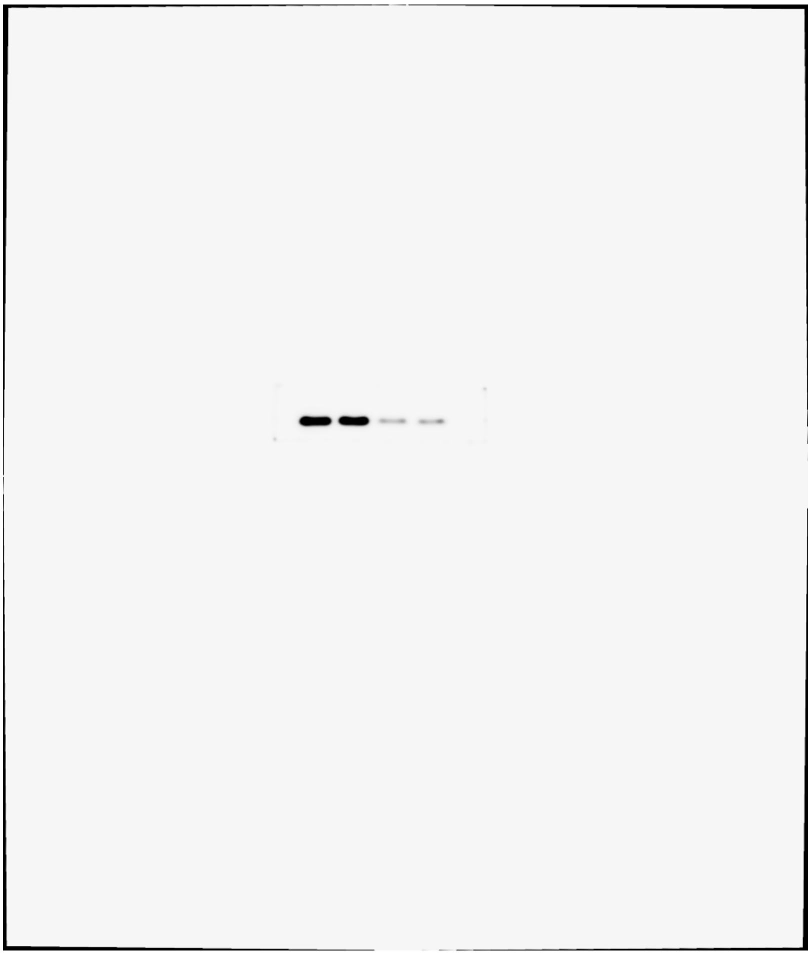

GAPDH (from lane 1 to lane 4)

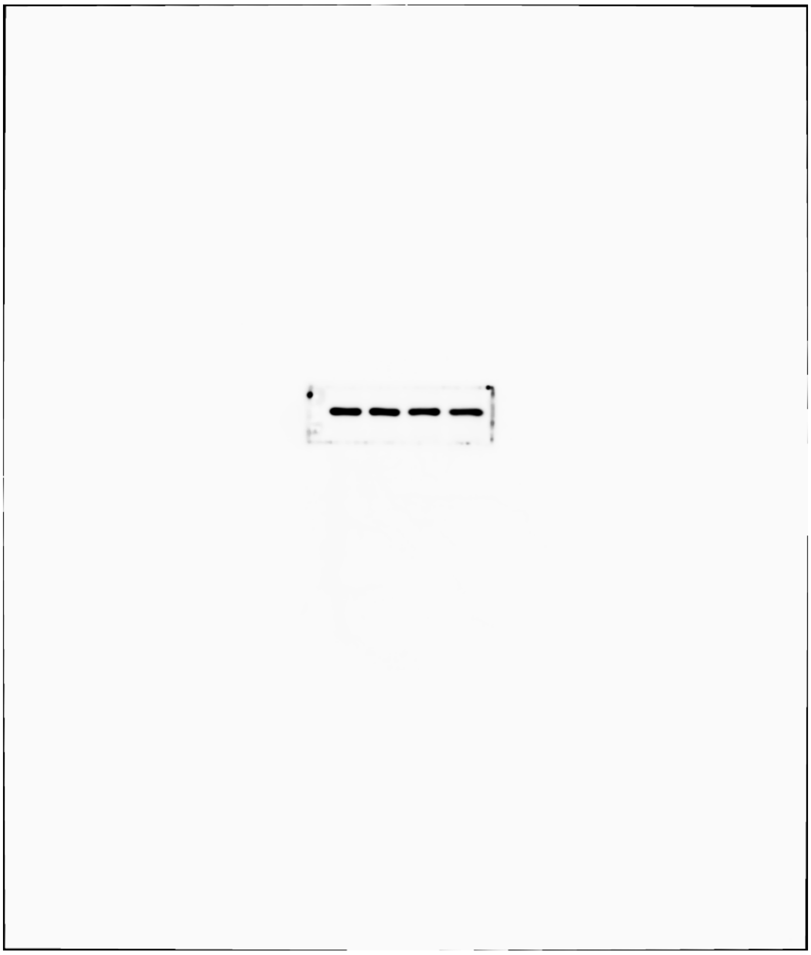

Full unedited gel for Supplemental Figure 1C

PPFIA4 (from lane 1 to lane 3)

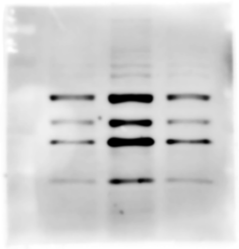

GAPDH (from lane 1 to lane 3)

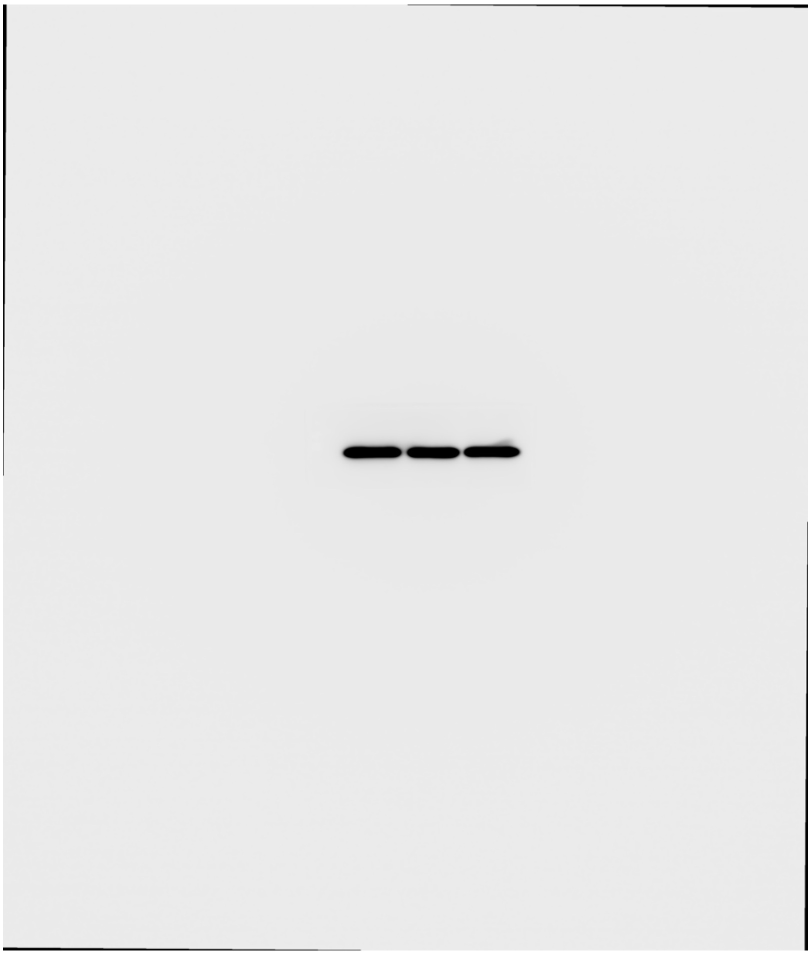

Full unedited gel for Supplemental Figure 1D(left)

PPFIA4 (from lane 1 to lane 6)

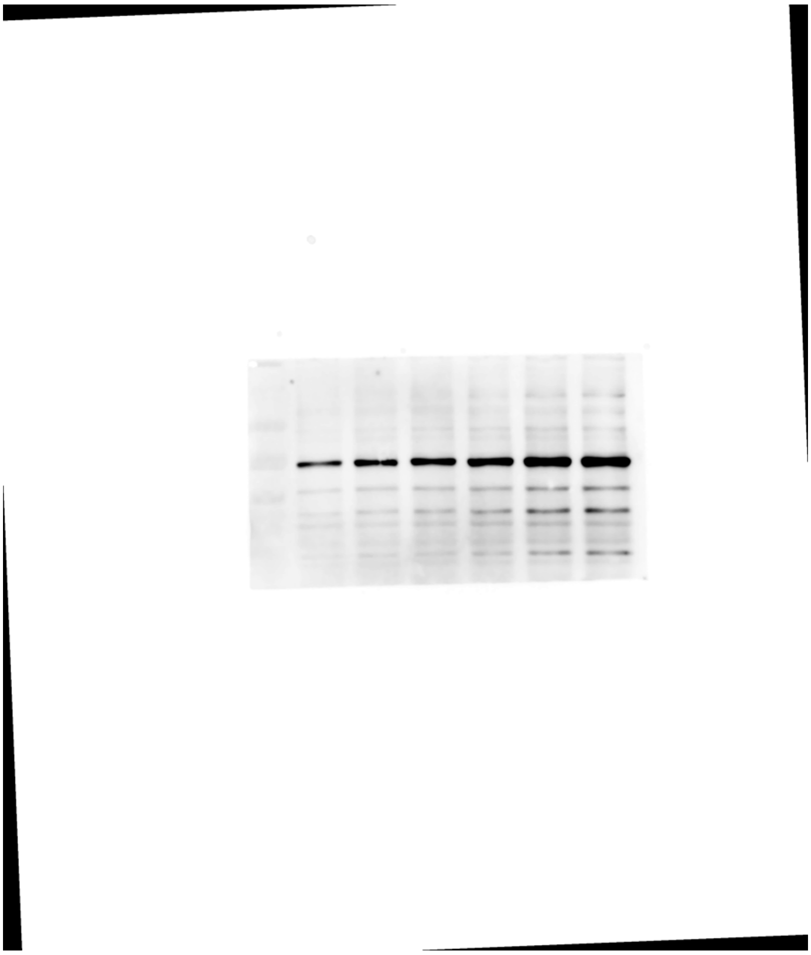

GAPDH (from lane 1 to lane 6)

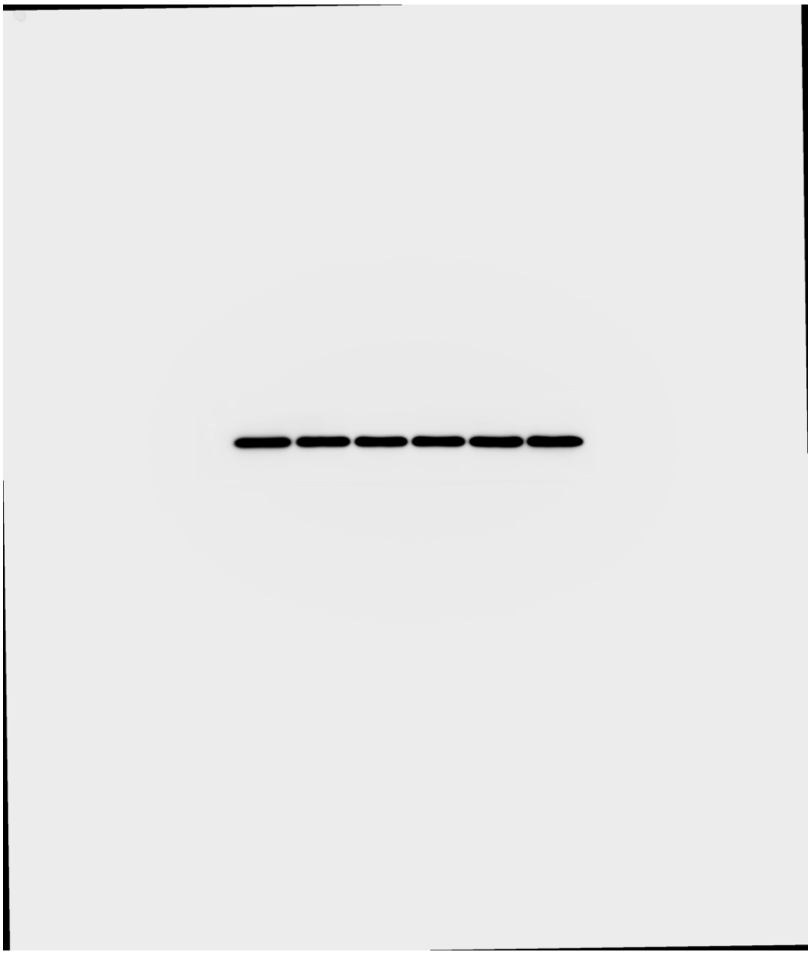

Full unedited gel for Supplemental Figure 1D(right)
PPFIA4 (from lane 1 to lane 6)

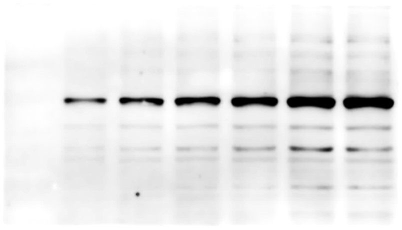

GAPDH (from lane 1 to lane 6)

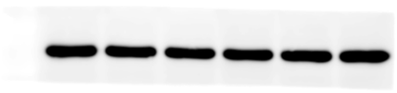

Full unedited gel for Supplemental Figure 1E(left)
PPFIA4 (from lane 1 to lane 6)

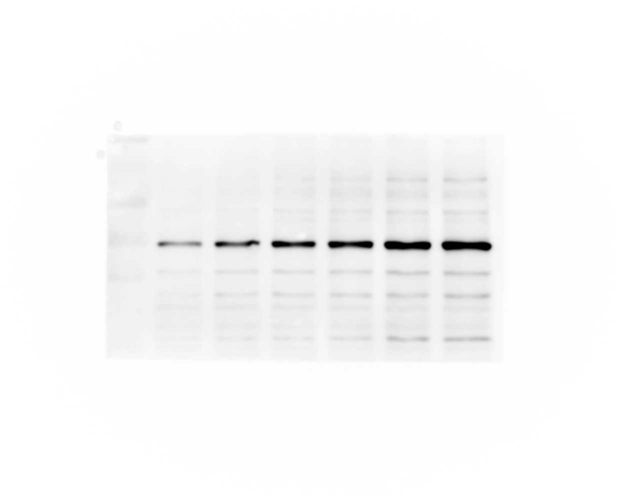

GAPDH (from lane 1 to lane 6)

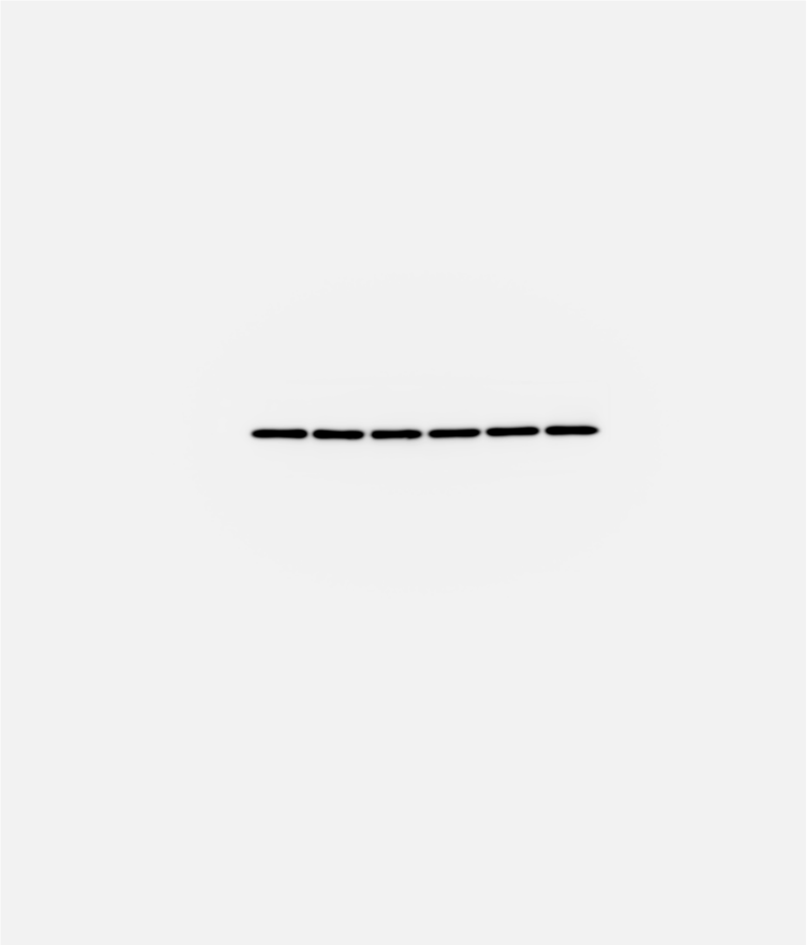

Full unedited gel for Supplemental Figure 1E(right)

PPFIA4 (from lane 1 to lane 6)

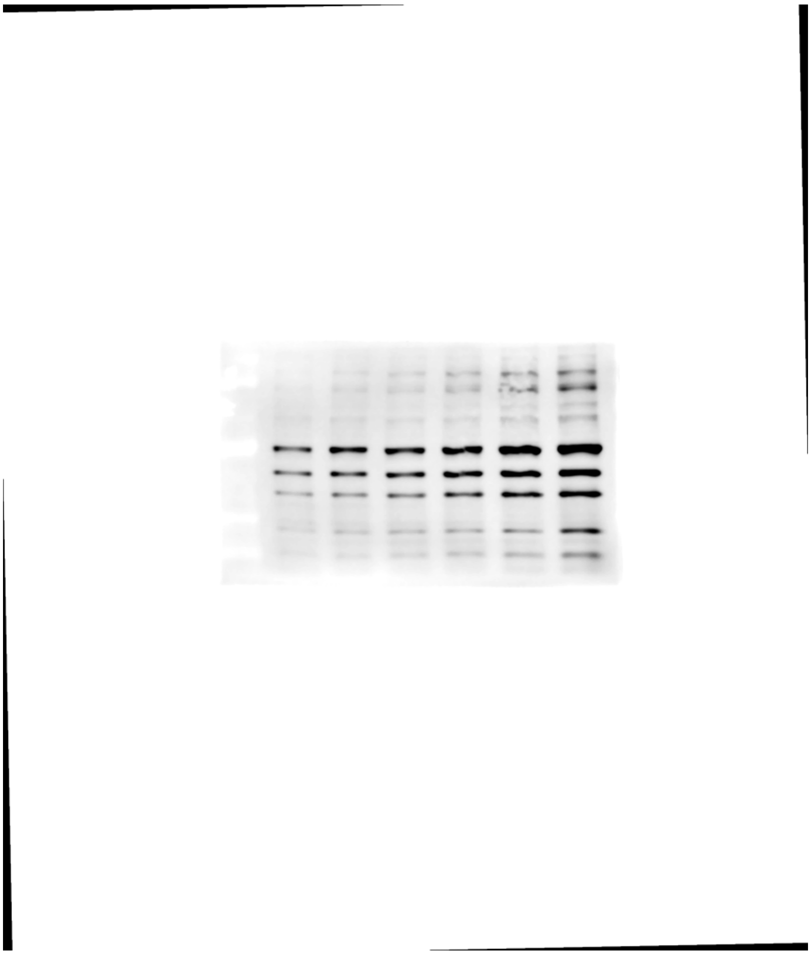

GAPDH (from lane 1 to lane 6)

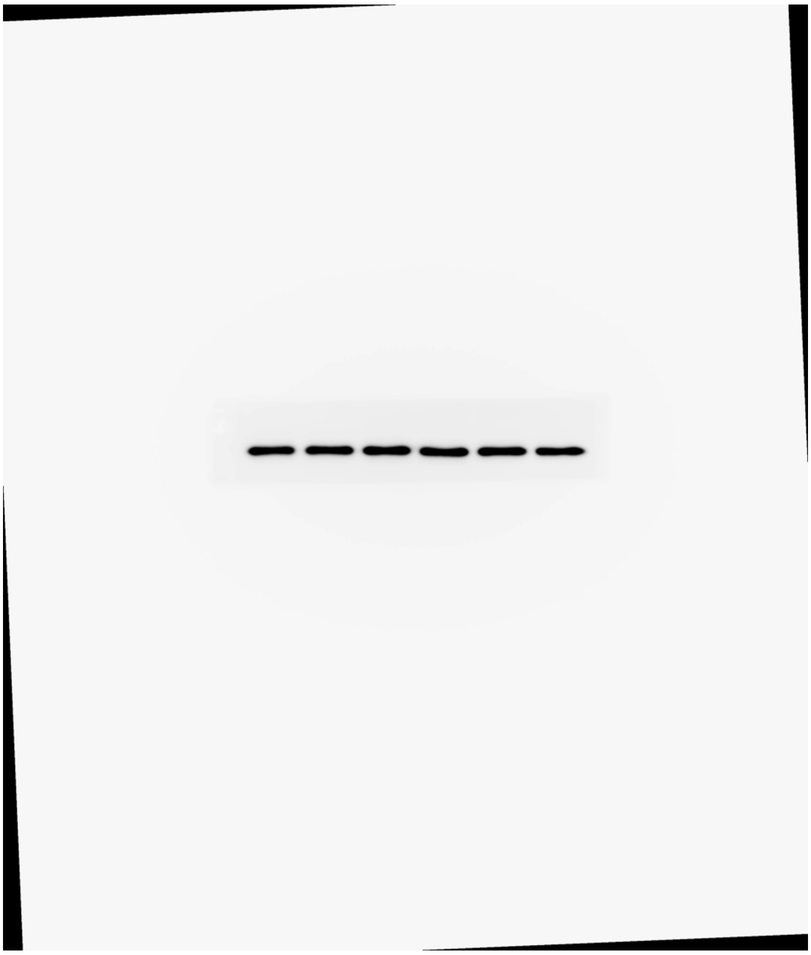

Full unedited gel for Supplemental Figure 2A

PPFIA4 (from lane 1 to lane 3)

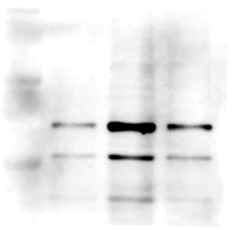

GAPDH (from lane 1 to lane 3)

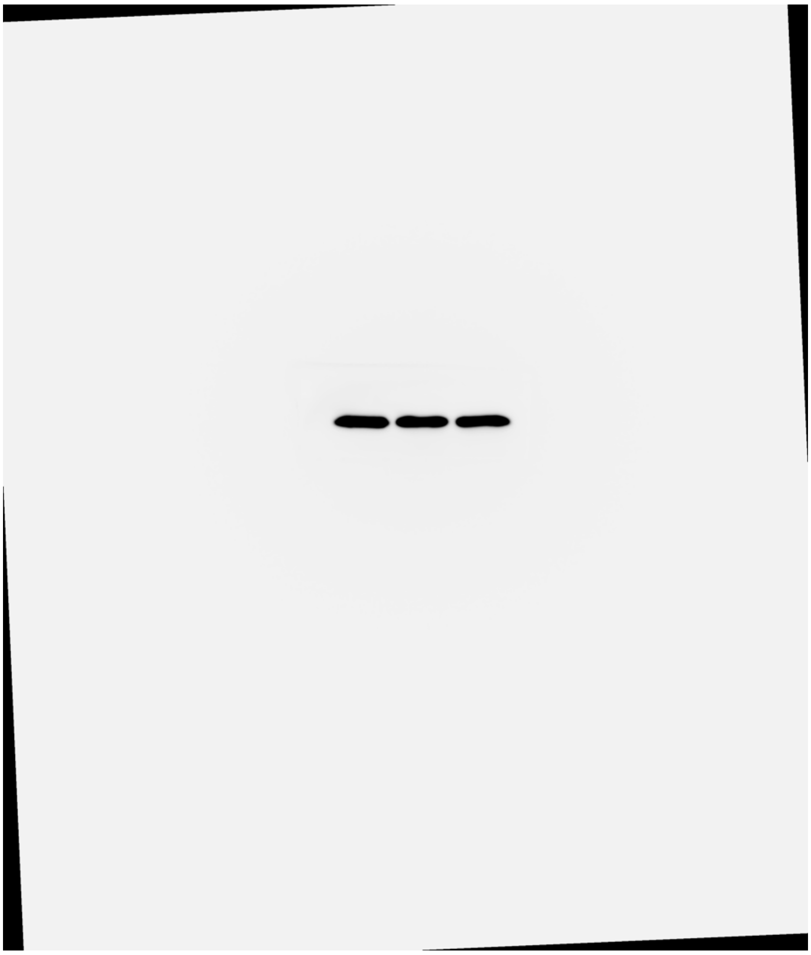

Full unedited gel for Supplemental Figure 2B

PPFIA4 (from lane 1 to lane 3)

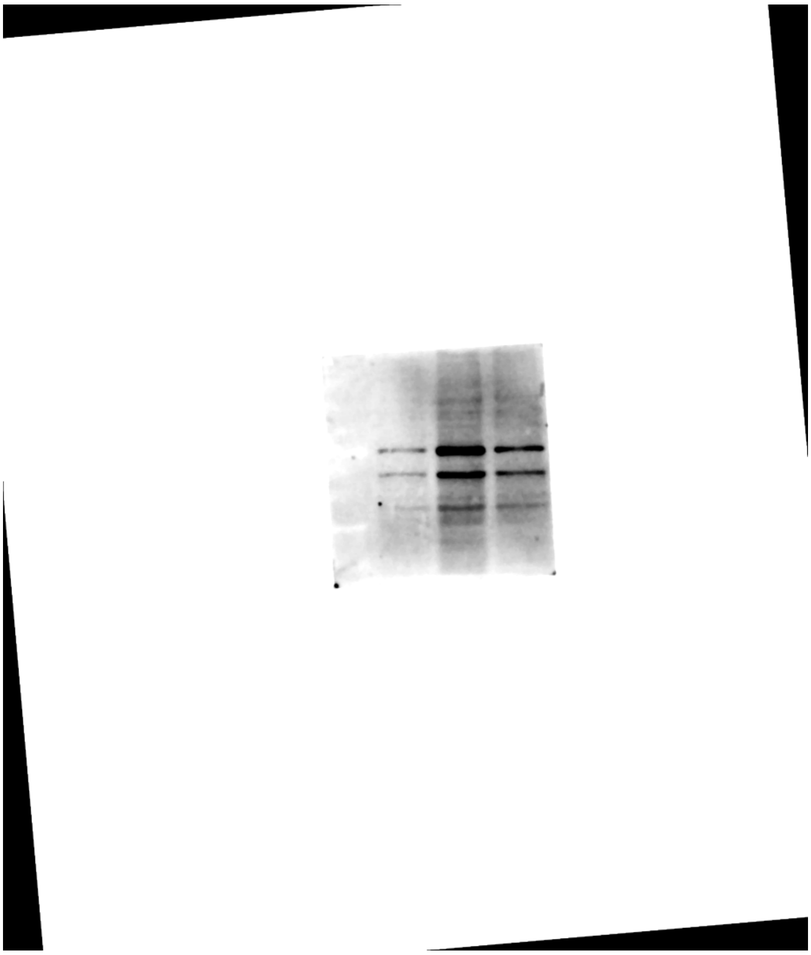

GAPDH (from lane 1 to lane 3)

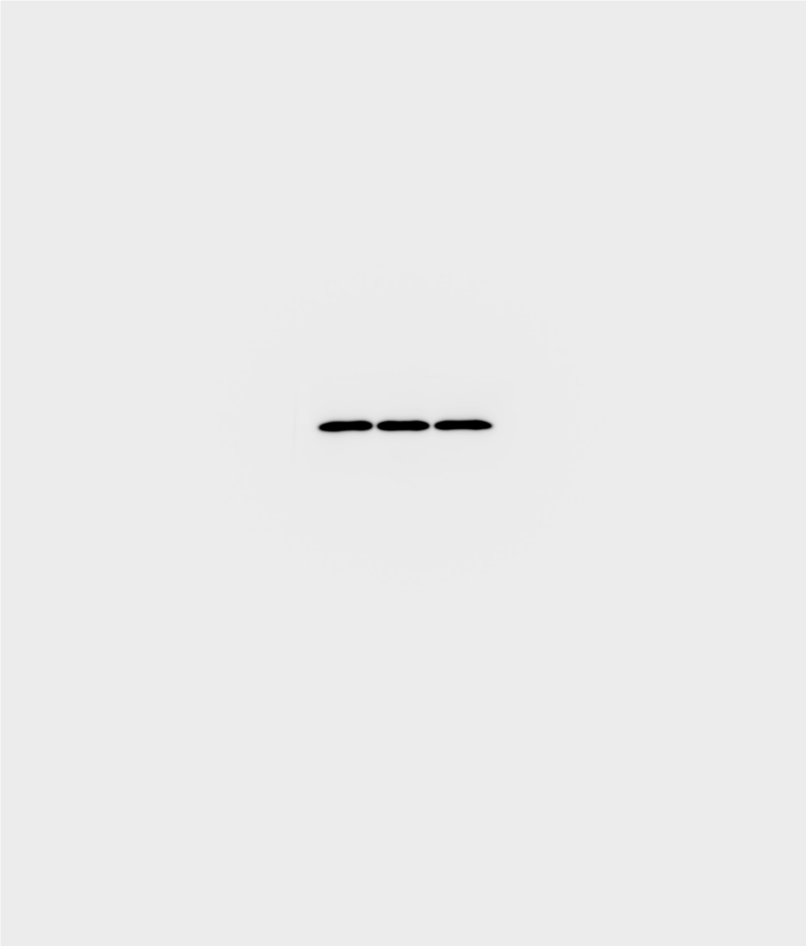

Full unedited gel for Supplemental Figure 2C

PPFIA4 (from lane 1 to lane 3)

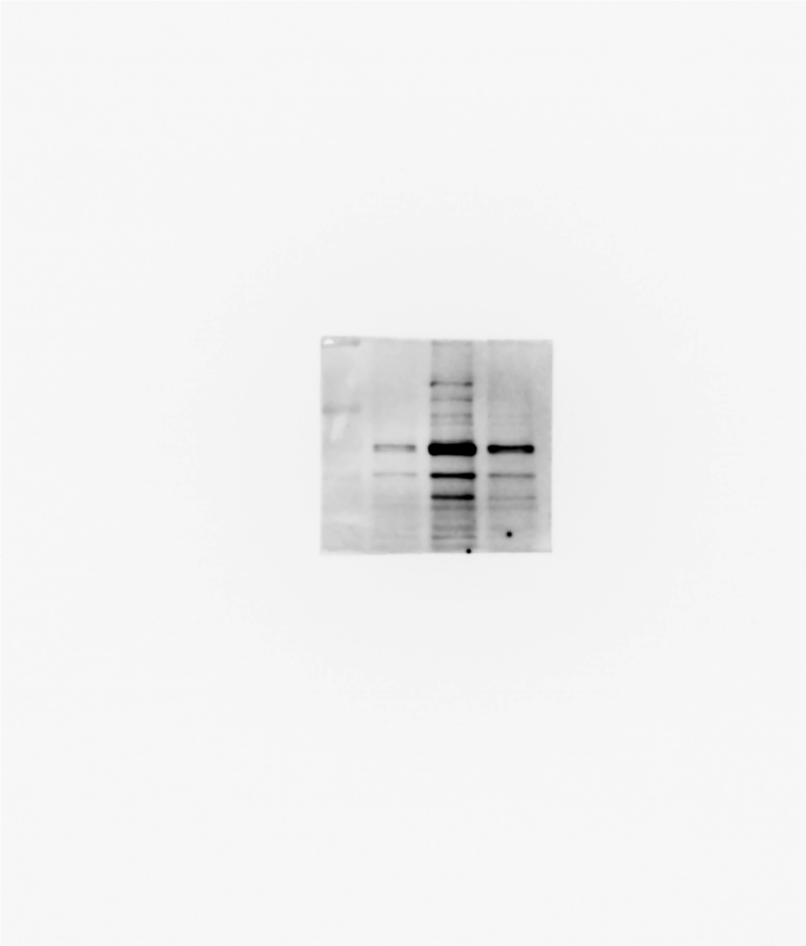

GAPDH (from lane 1 to lane 3)

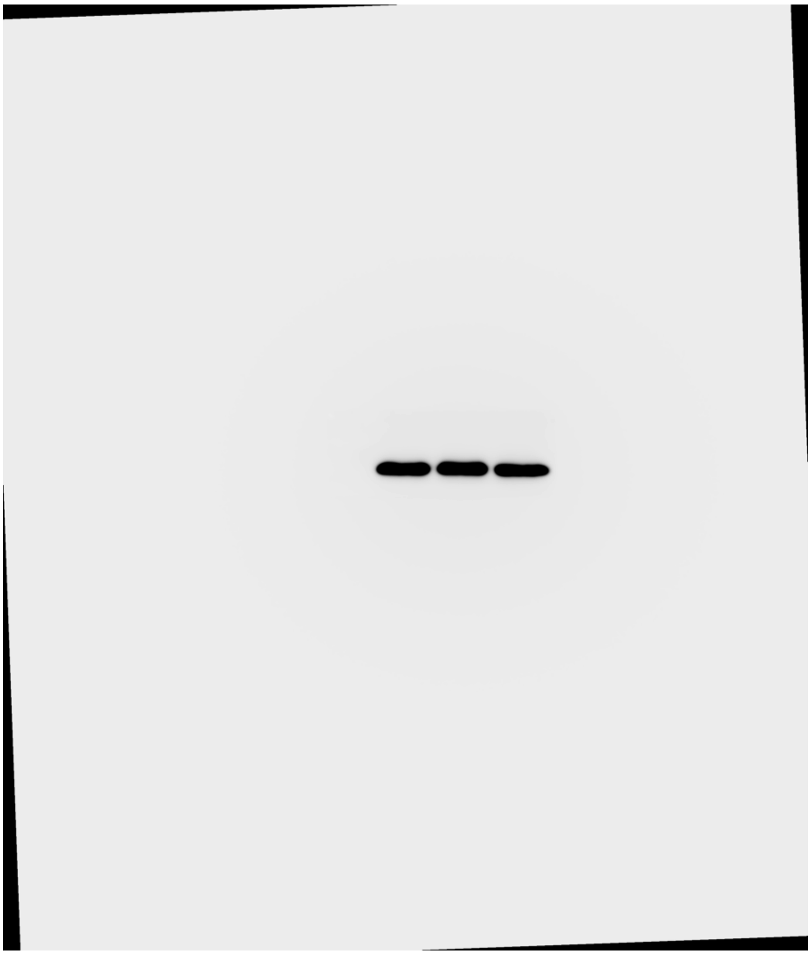

Full unedited gel for Supplemental Figure 2D

PPFIA4 (from lane 1 to lane 3)

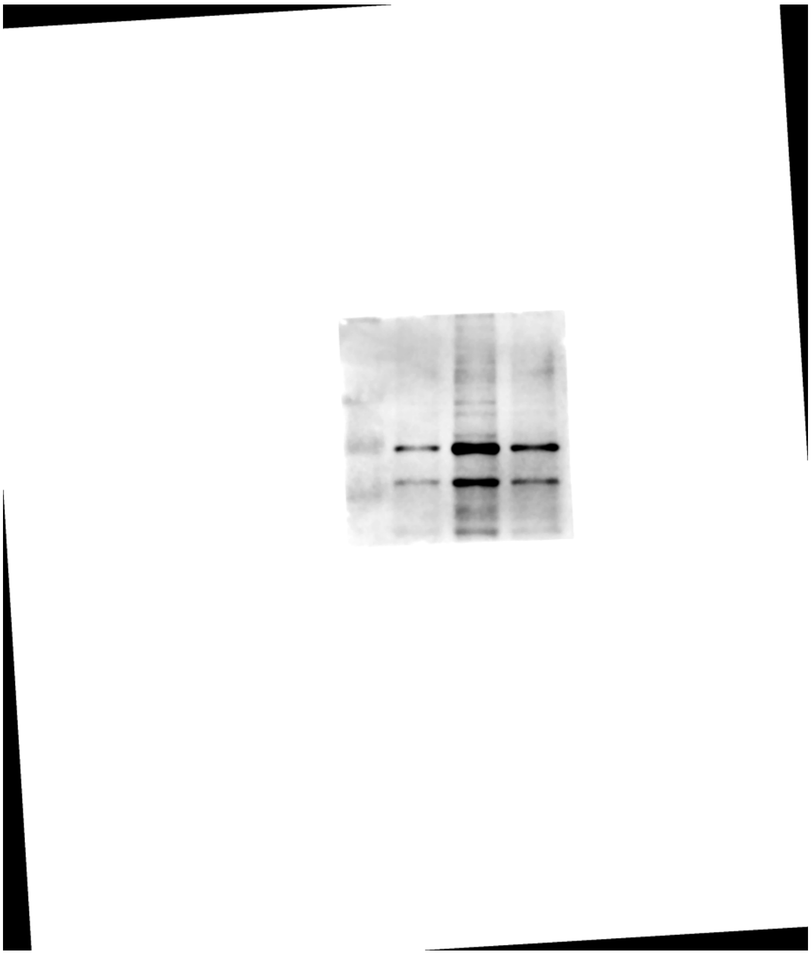

GAPDH (from lane 1 to lane 3)

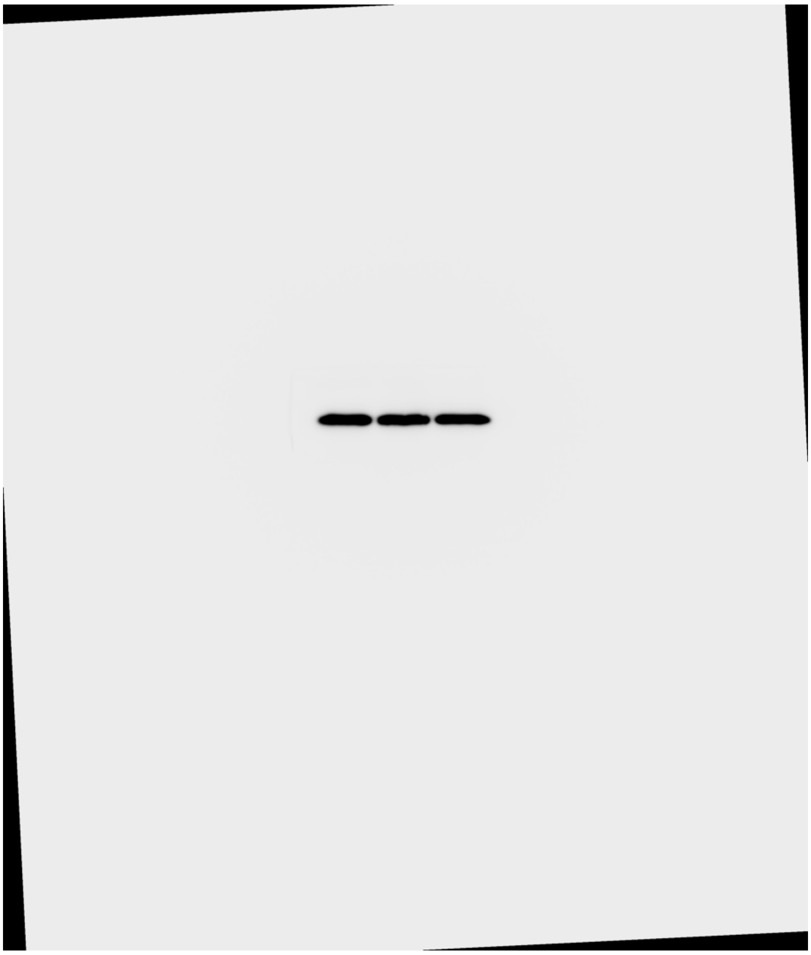

Full unedited gel for Supplemental Figure 2E

PPFIA4 (from lane 1 to lane 3)

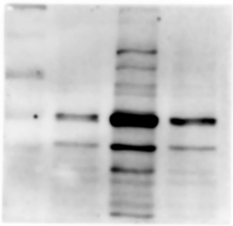

GAPDH (from lane 1 to lane 3)

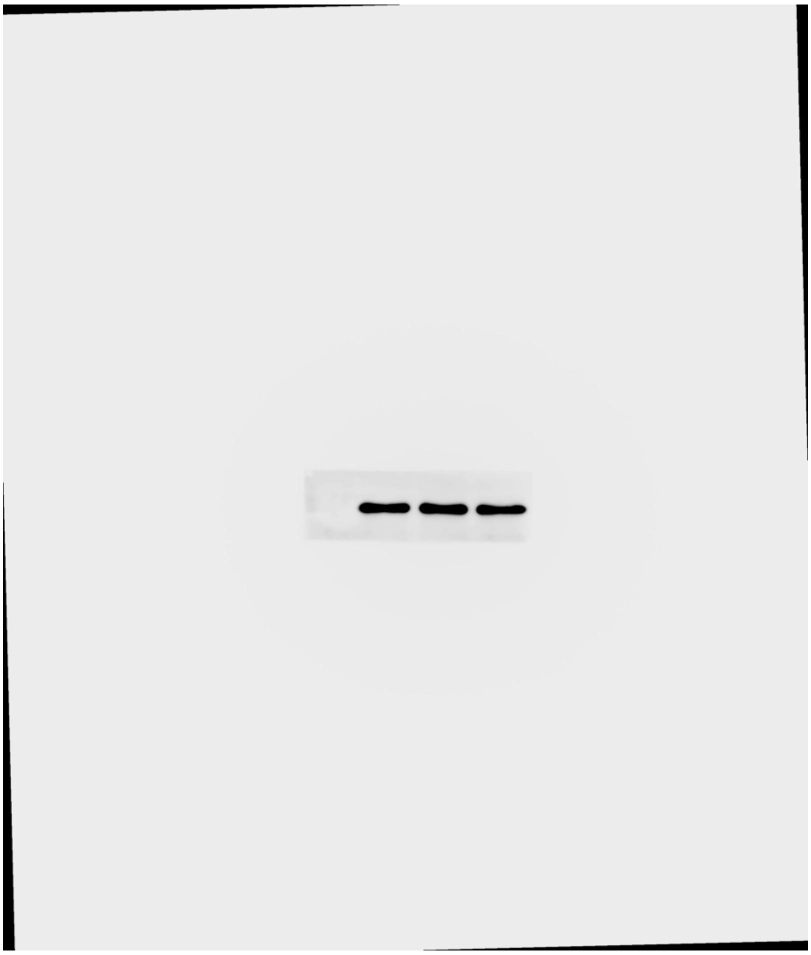

Full unedited gel for Supplemental Figure 2G(left)

PPFIA4 (from lane 1 to lane 9)

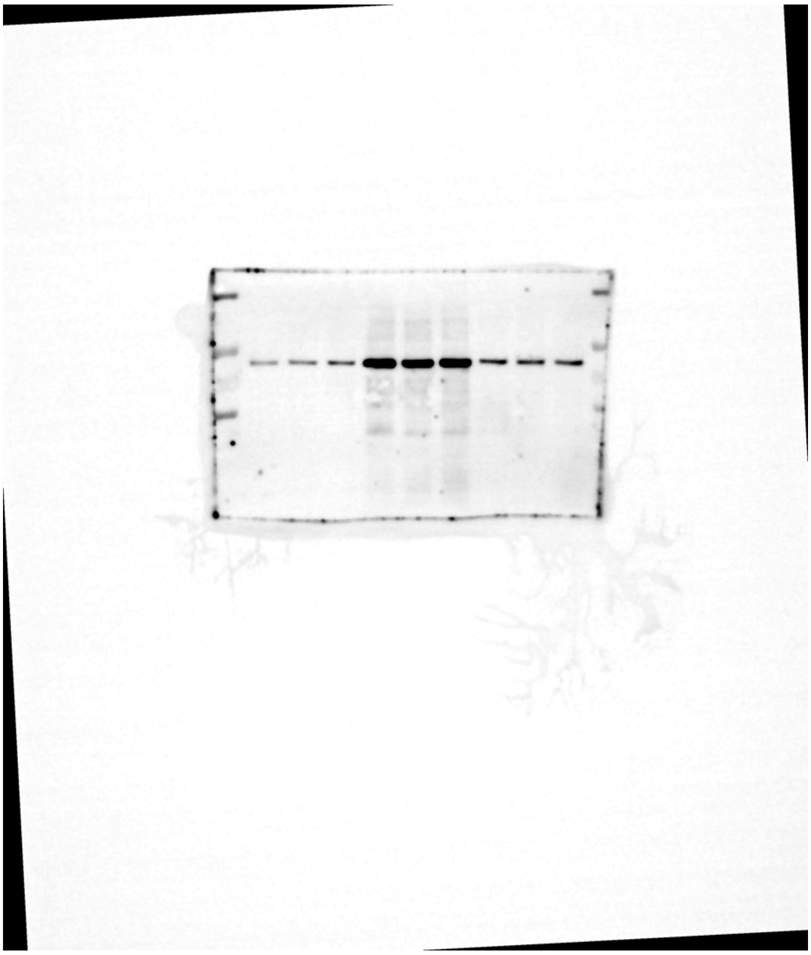

GAPDH (from lane 1 to lane 9)

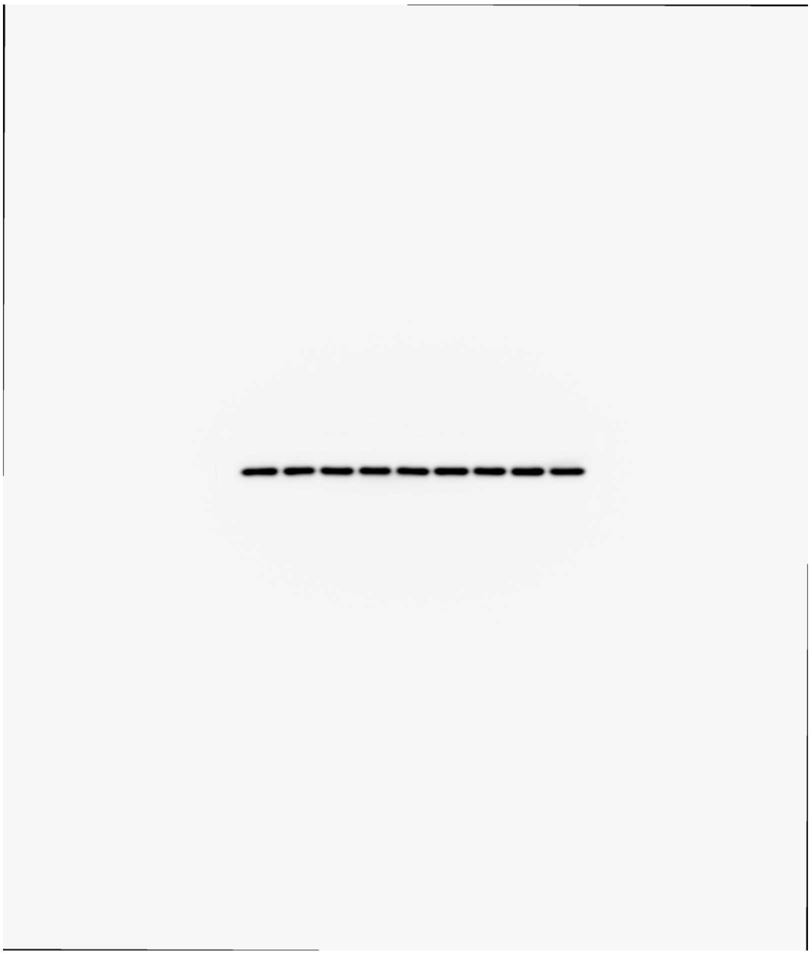

Full unedited gel for Supplemental Figure 2G(right)

PPFIA4 (from lane 1 to lane 9)

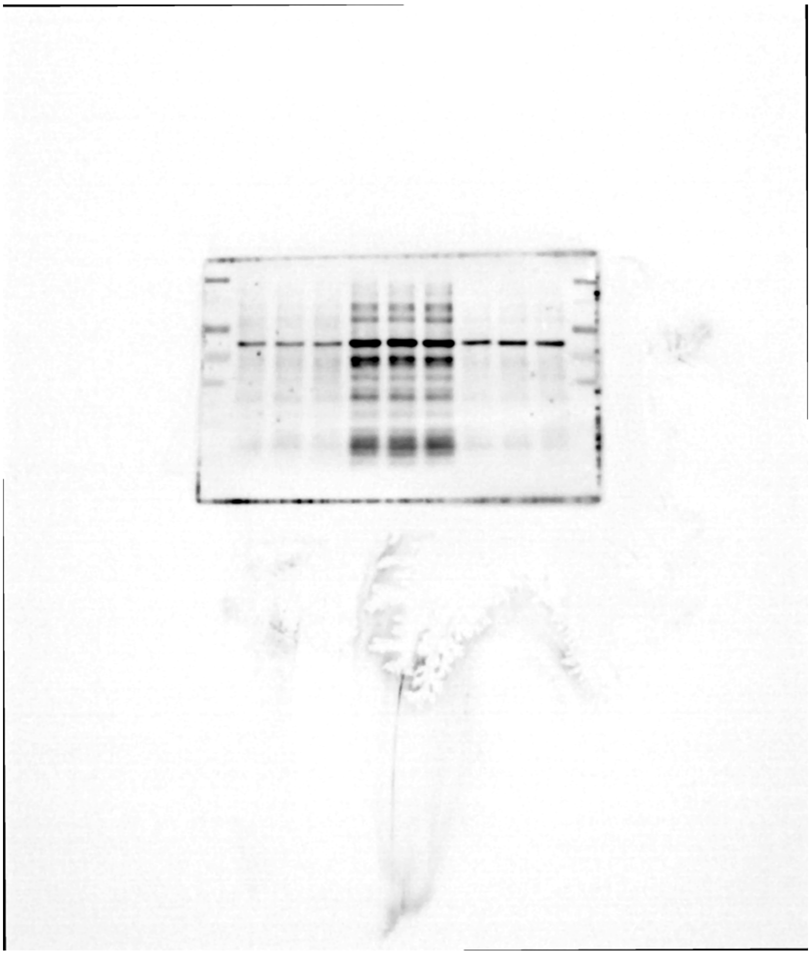

GAPDH (from lane 1 to lane 9)

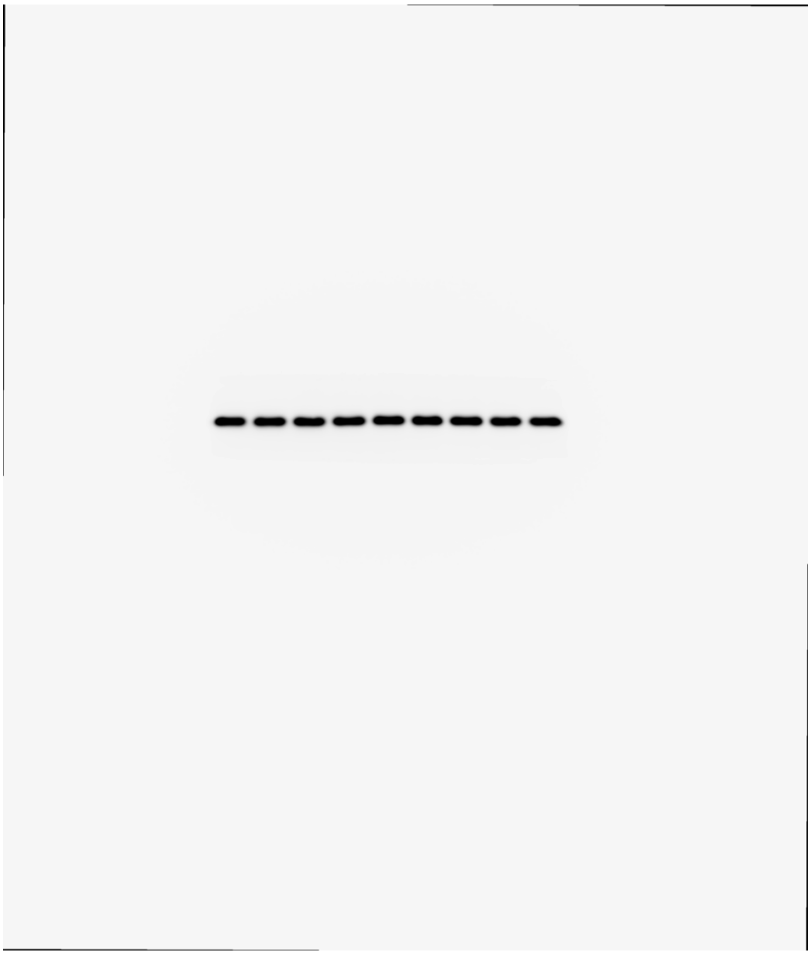

Full unedited gel for Supplemental Figure 2N
PPFIA4 (from lane 1 to lane 3)

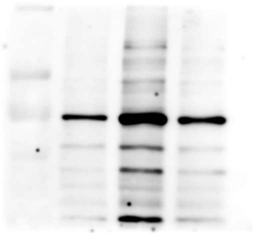

GAPDH (from lane 1 to lane 3)

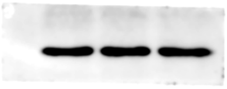

Full unedited gel for Supplemental Figure 3E

PPFIA4 (from lane 1 to lane 7)

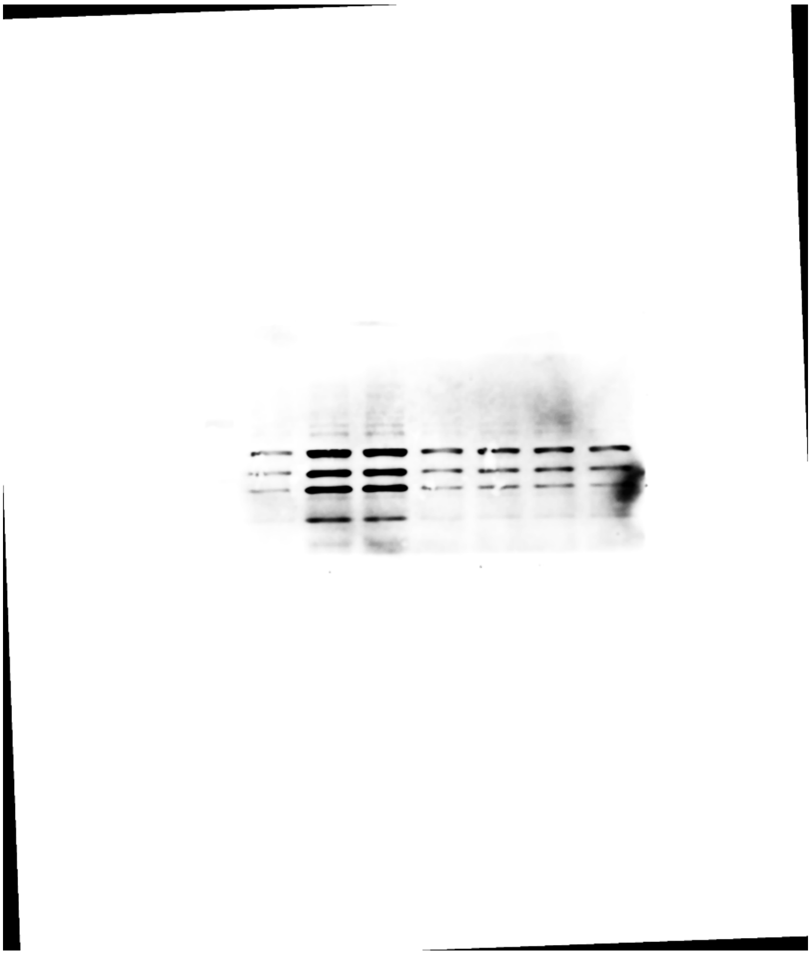

p-c-Jun (from lane 1 to lane 7)

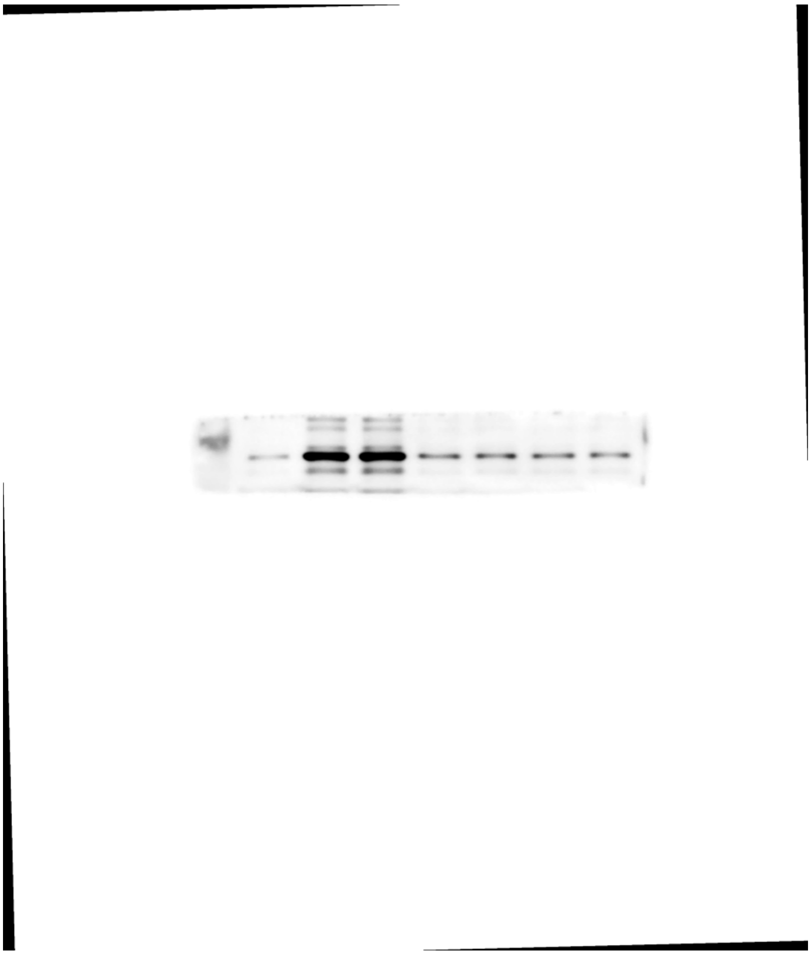

c-Jun (from lane 1 to lane 7)

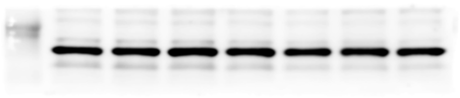

GAPDH (from lane 1 to lane 7)

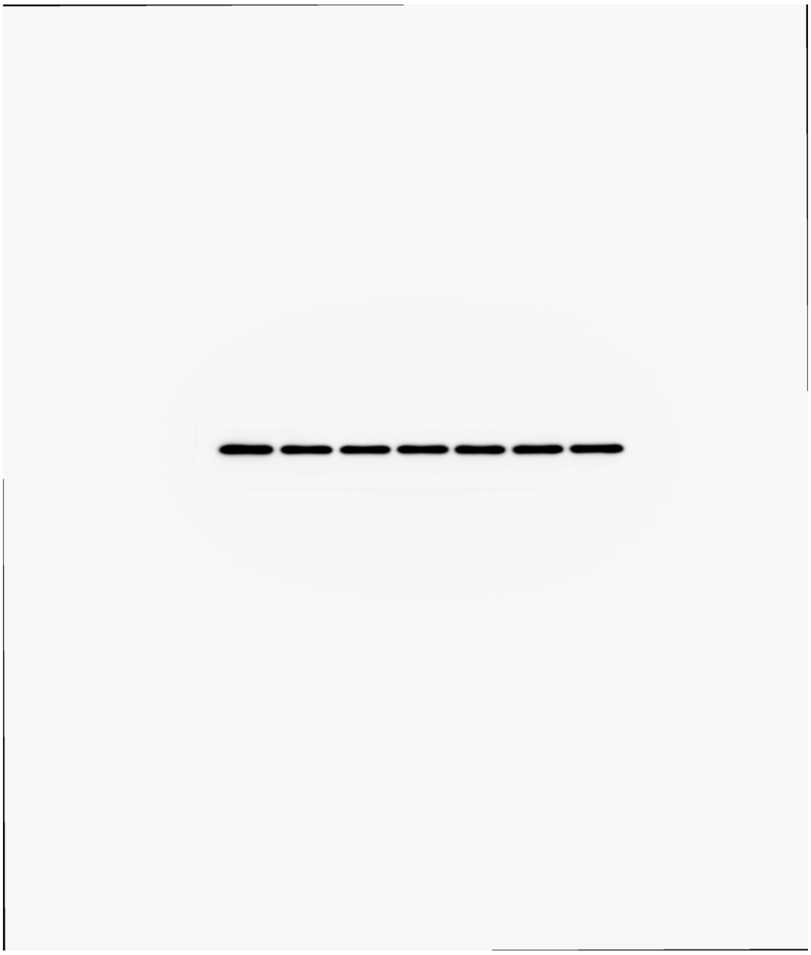

Full unedited gel for Supplemental Figure 3F(left)

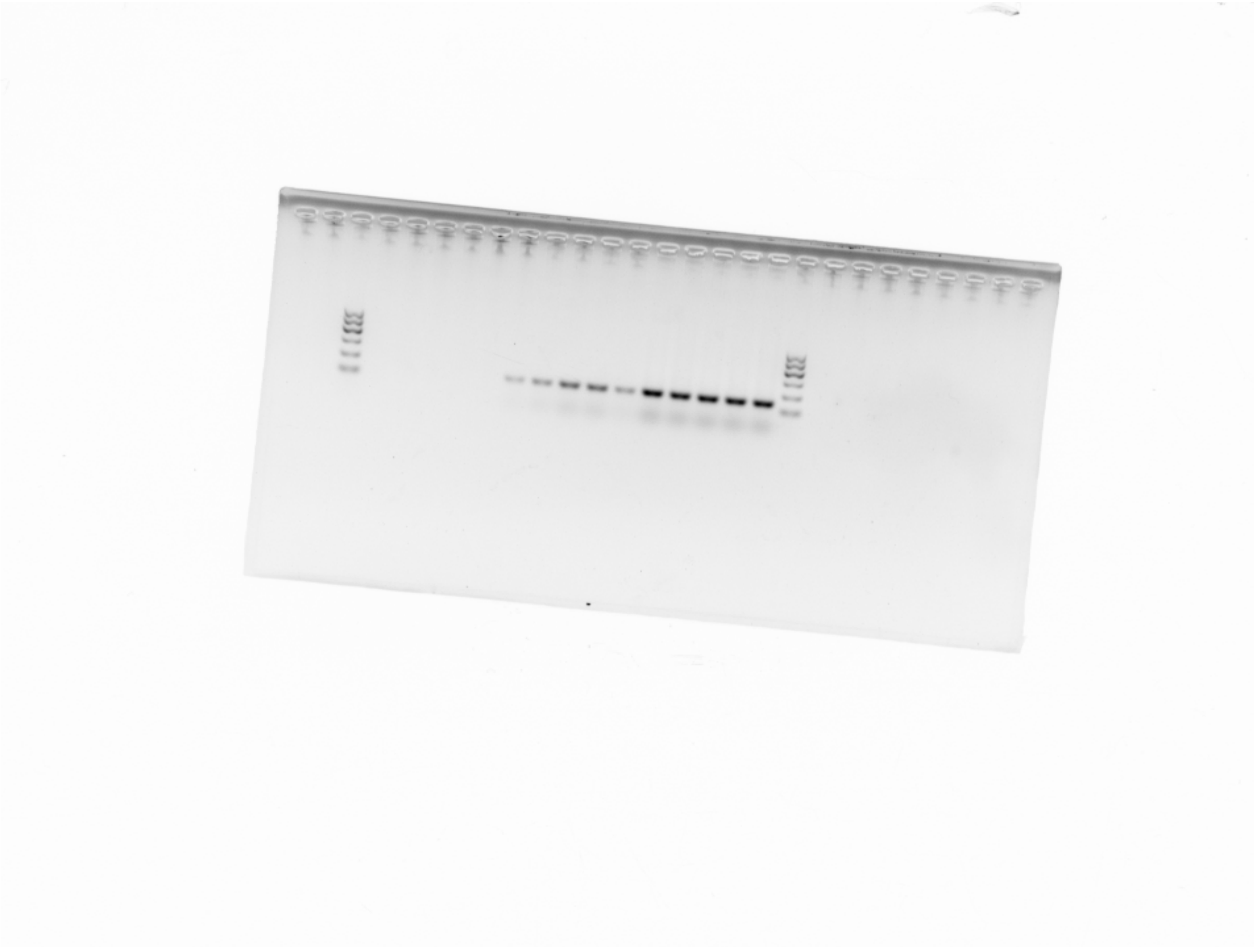

Full unedited gel for Supplemental Figure 3F(right)

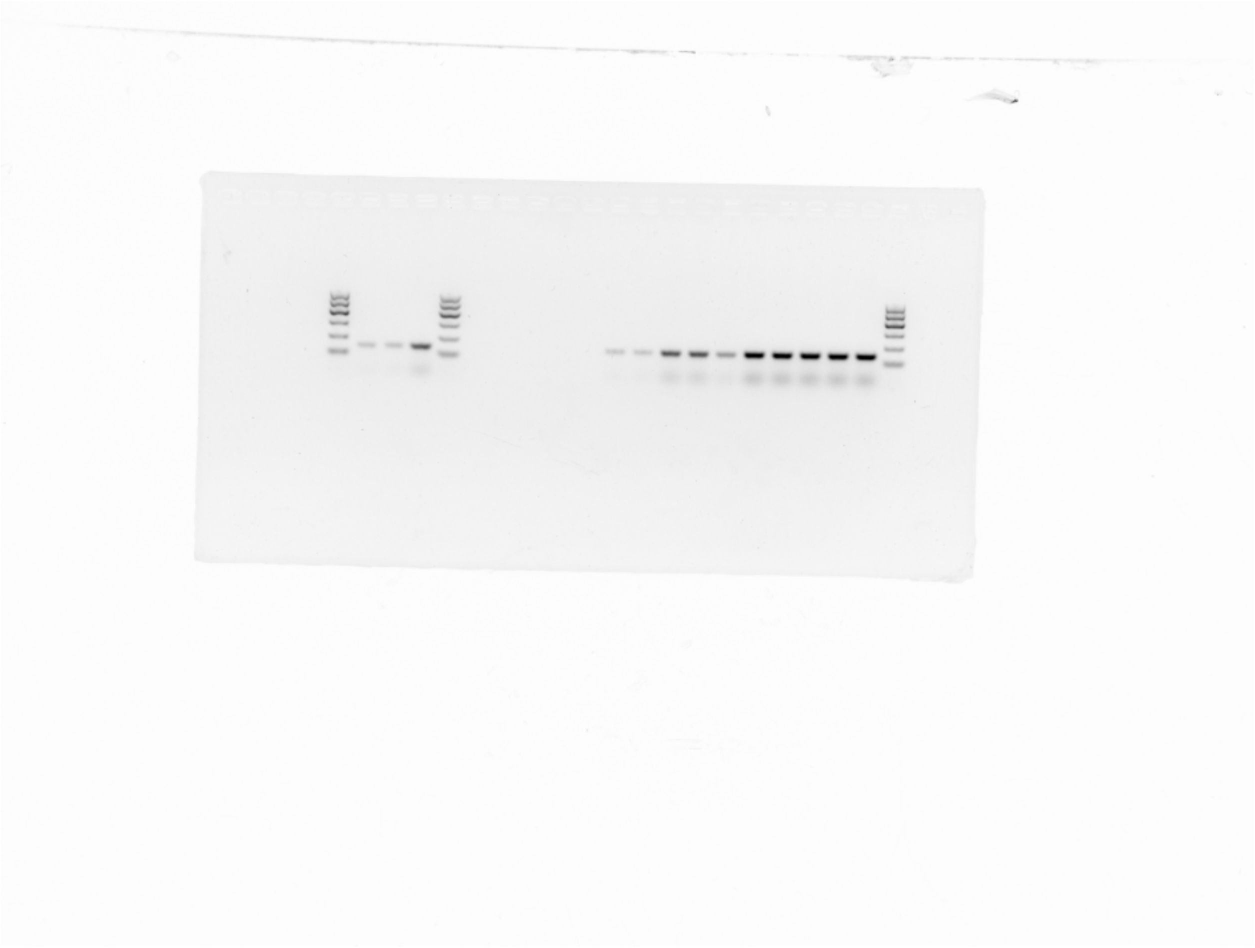

Full unedited gel for Supplemental Figure 4B

PPFIA4 (from lane 1 to lane 4)

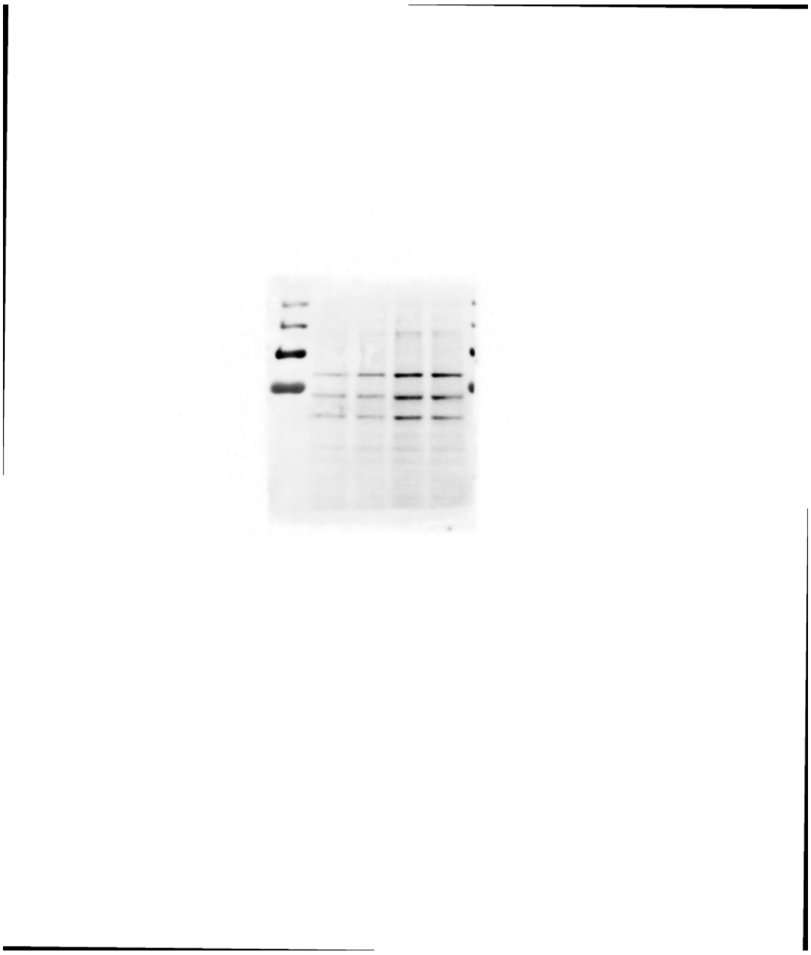

GAPDH (from lane 1 to lane 4)

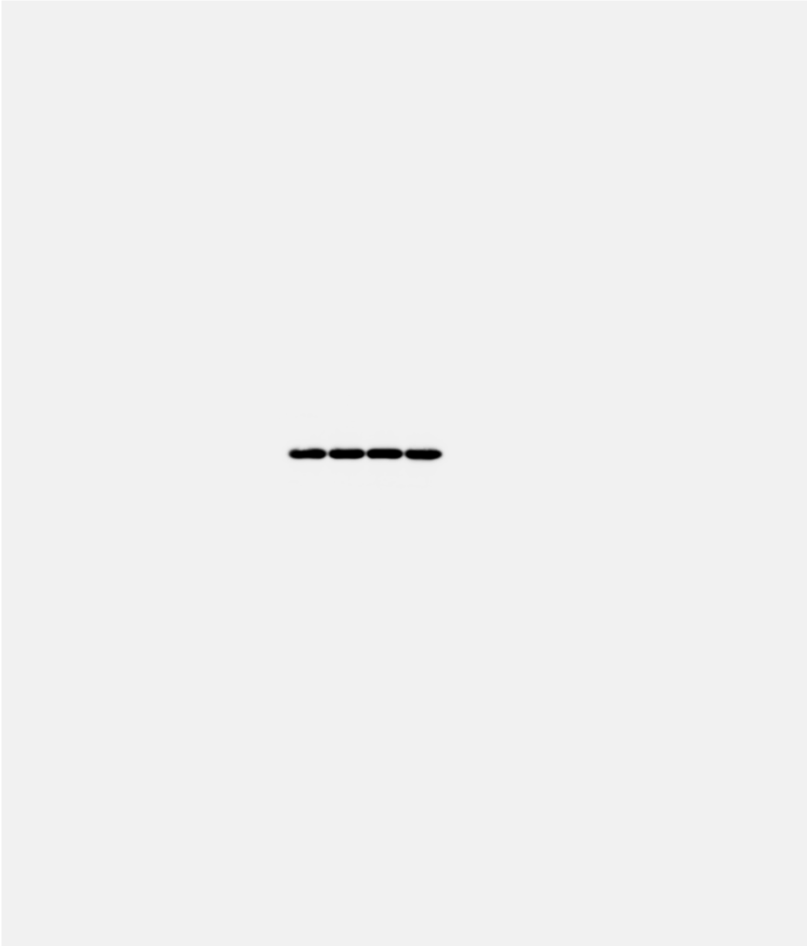

Full unedited gel for Supplemental Figure 4F

PPFIA4 (from lane 1 to lane 8)

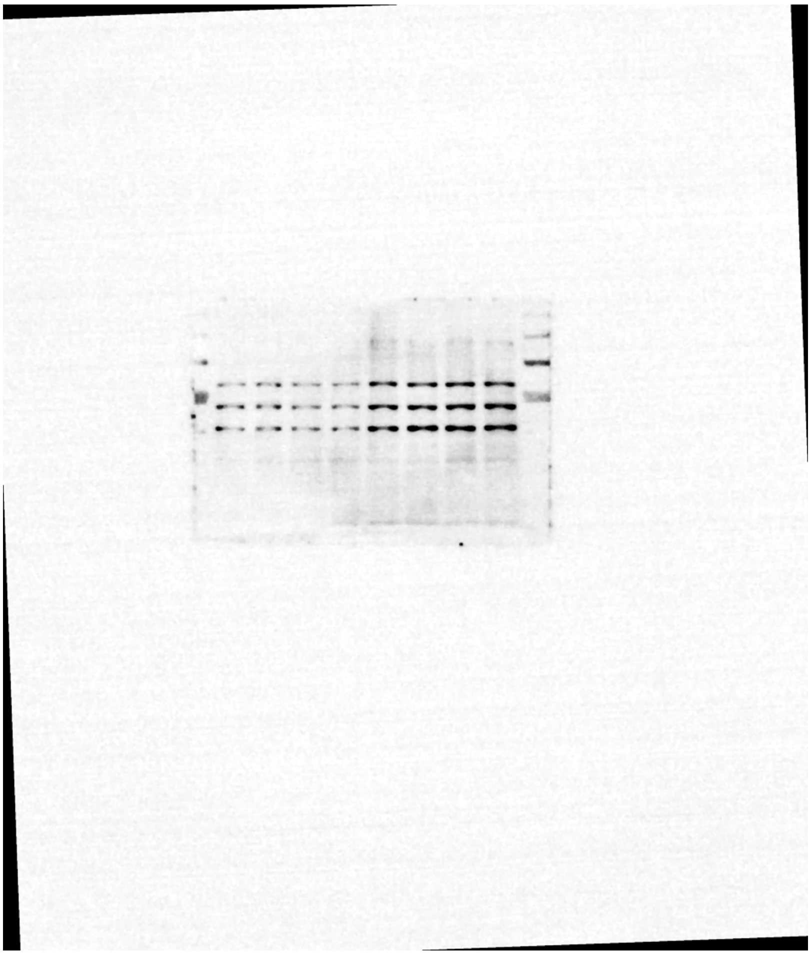

GAPDH (from lane 1 to lane 8)

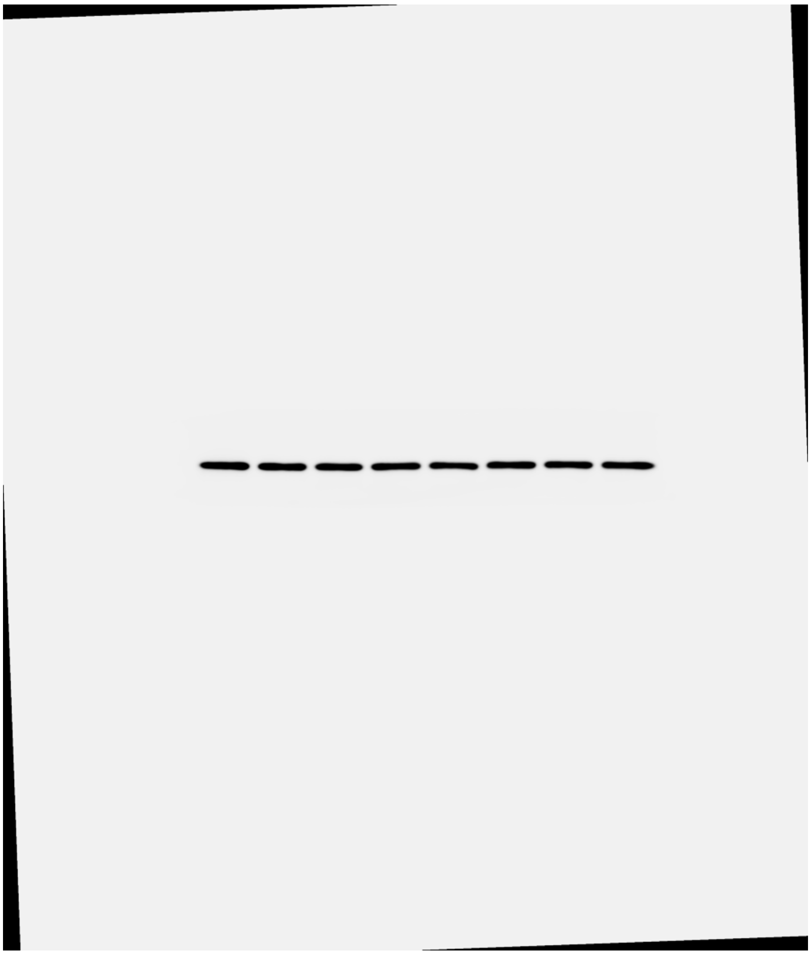

Full unedited gel for Supplemental Figure 5B(up)

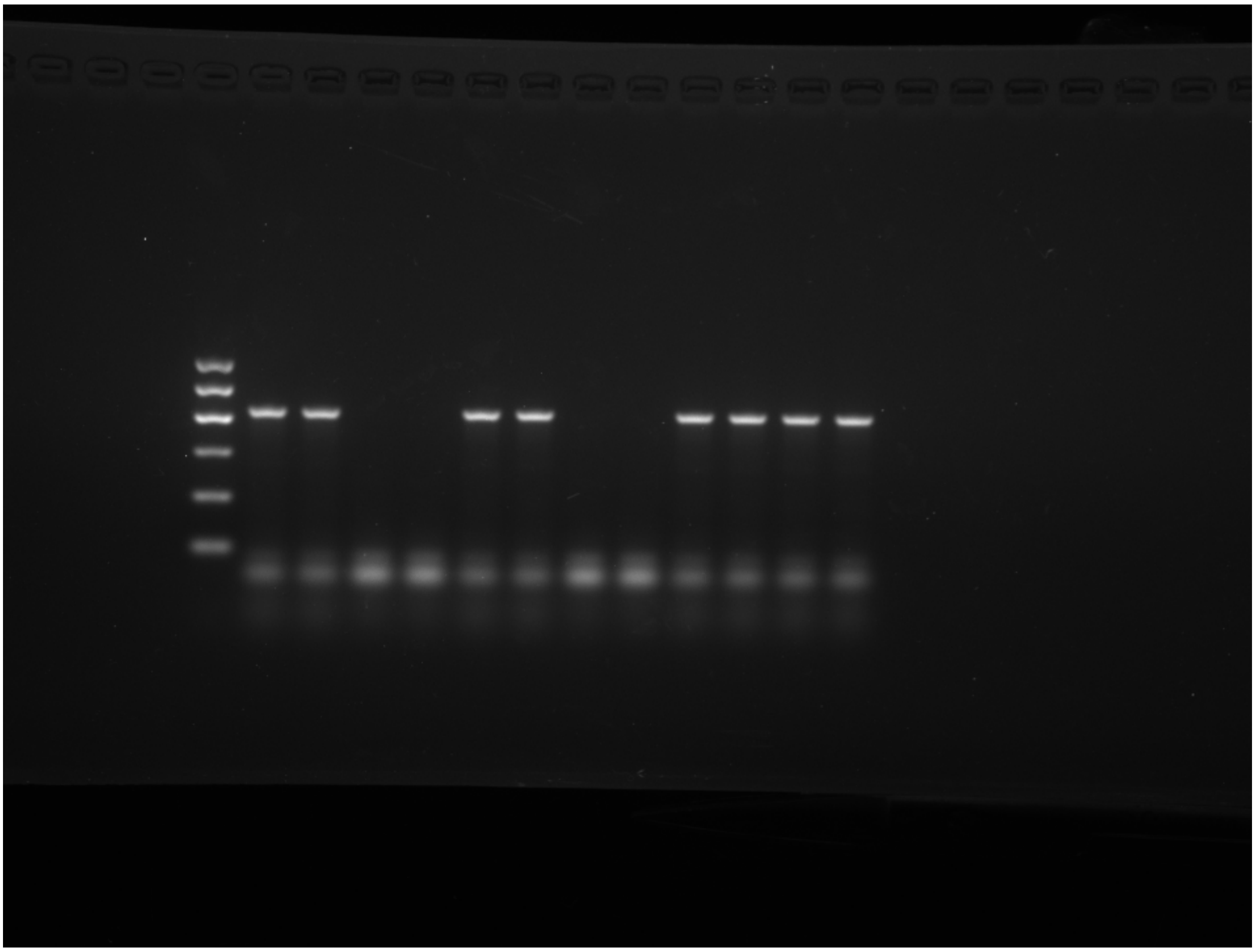

Full unedited gel for Supplemental Figure 5B(down)

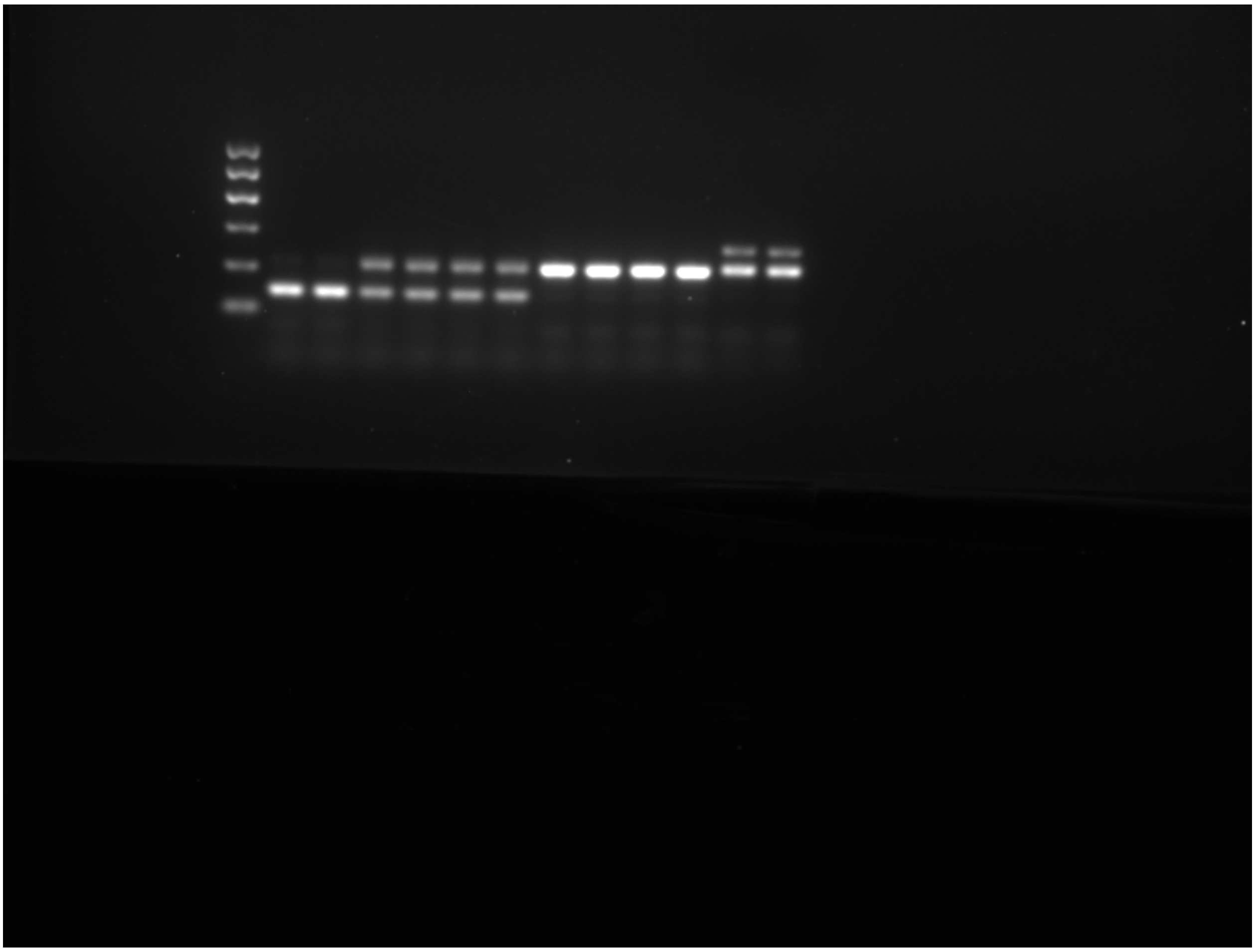

Full unedited gel for Supplemental Figure 11N(up)

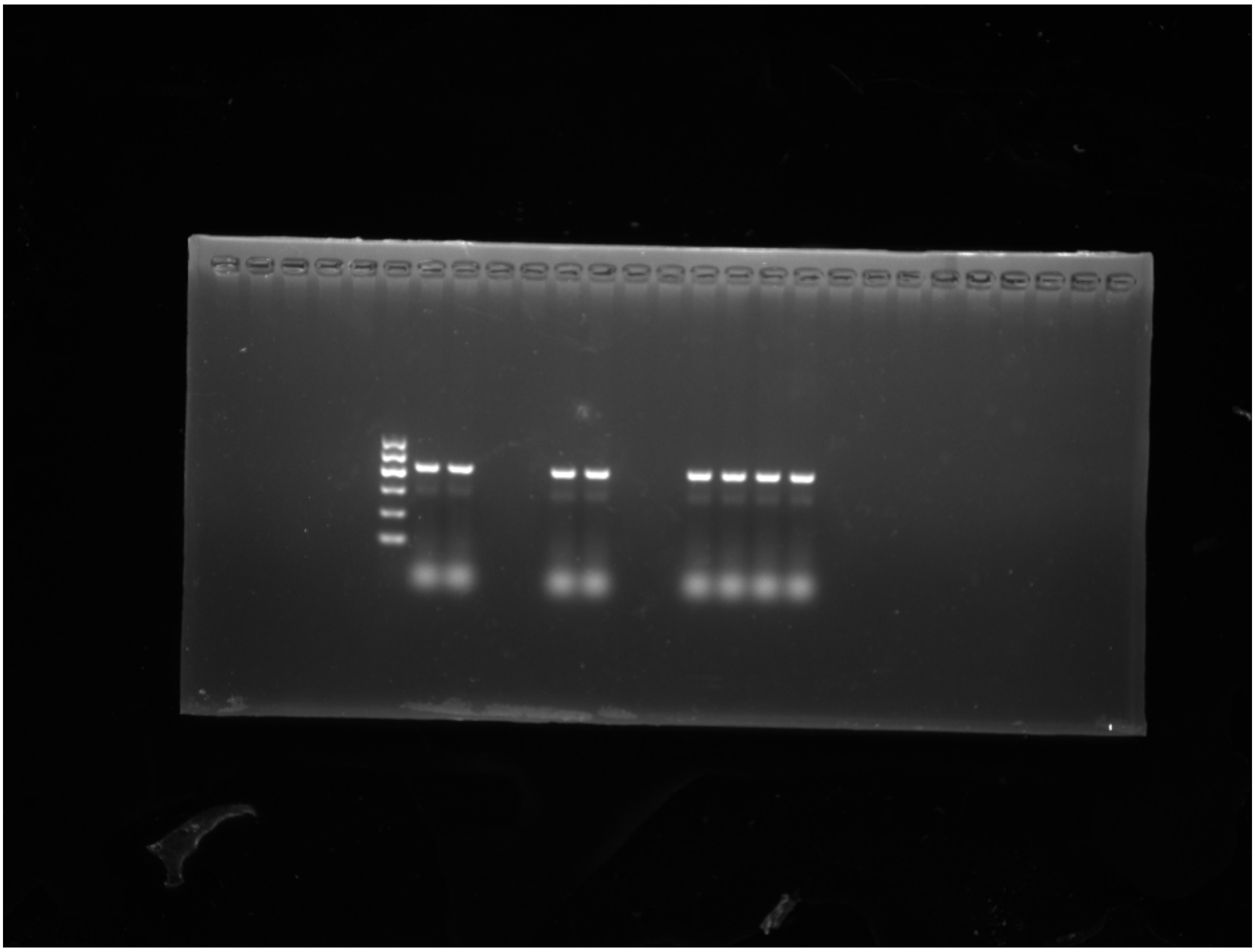

Full unedited gel for Supplemental Figure 11N(down)

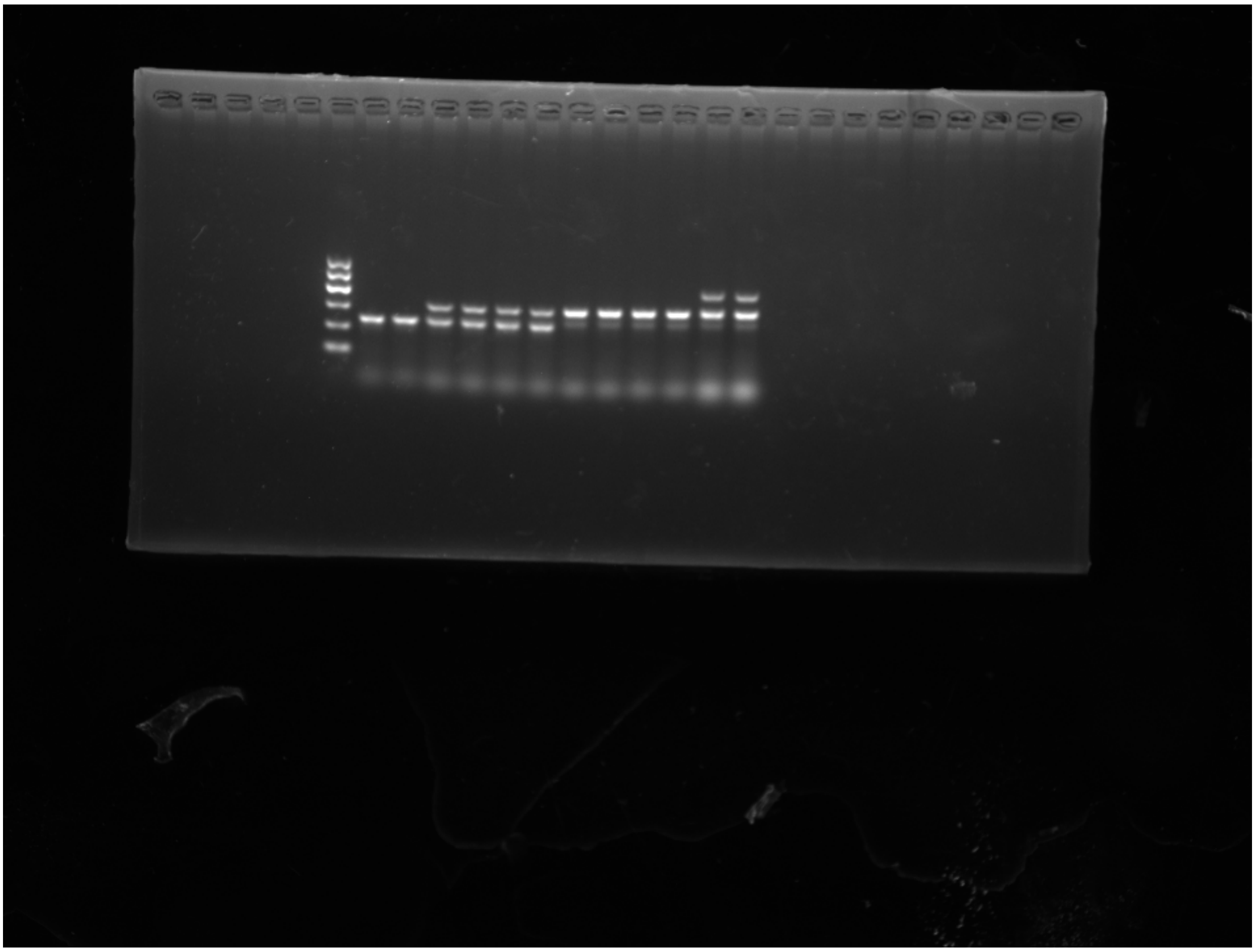

Full unedited gel for Supplemental Figure 13A

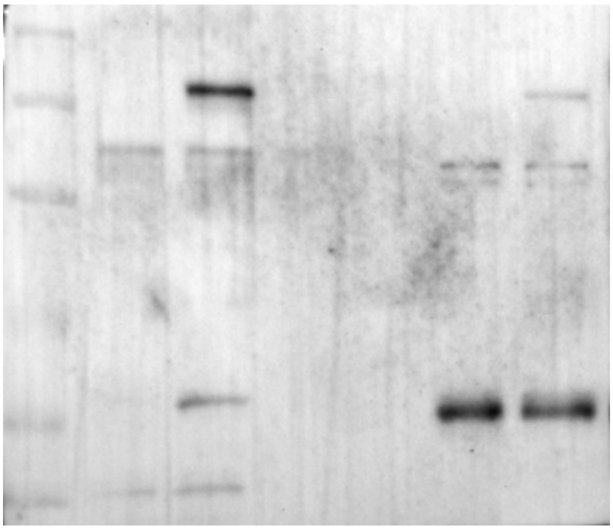

Full unedited gel for Supplemental Figure 20G(left)

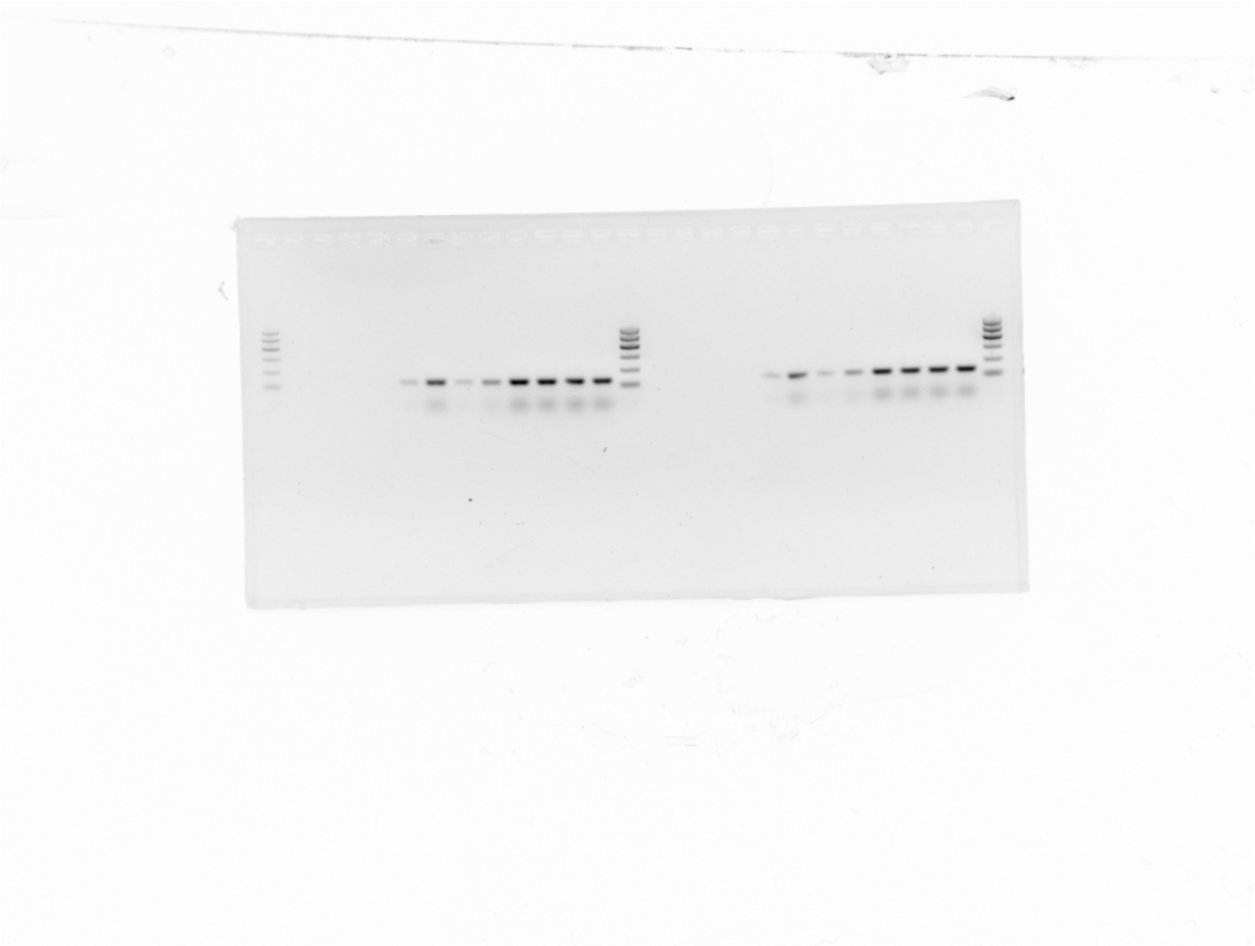

Full unedited gel for Supplemental Figure 20G(right)

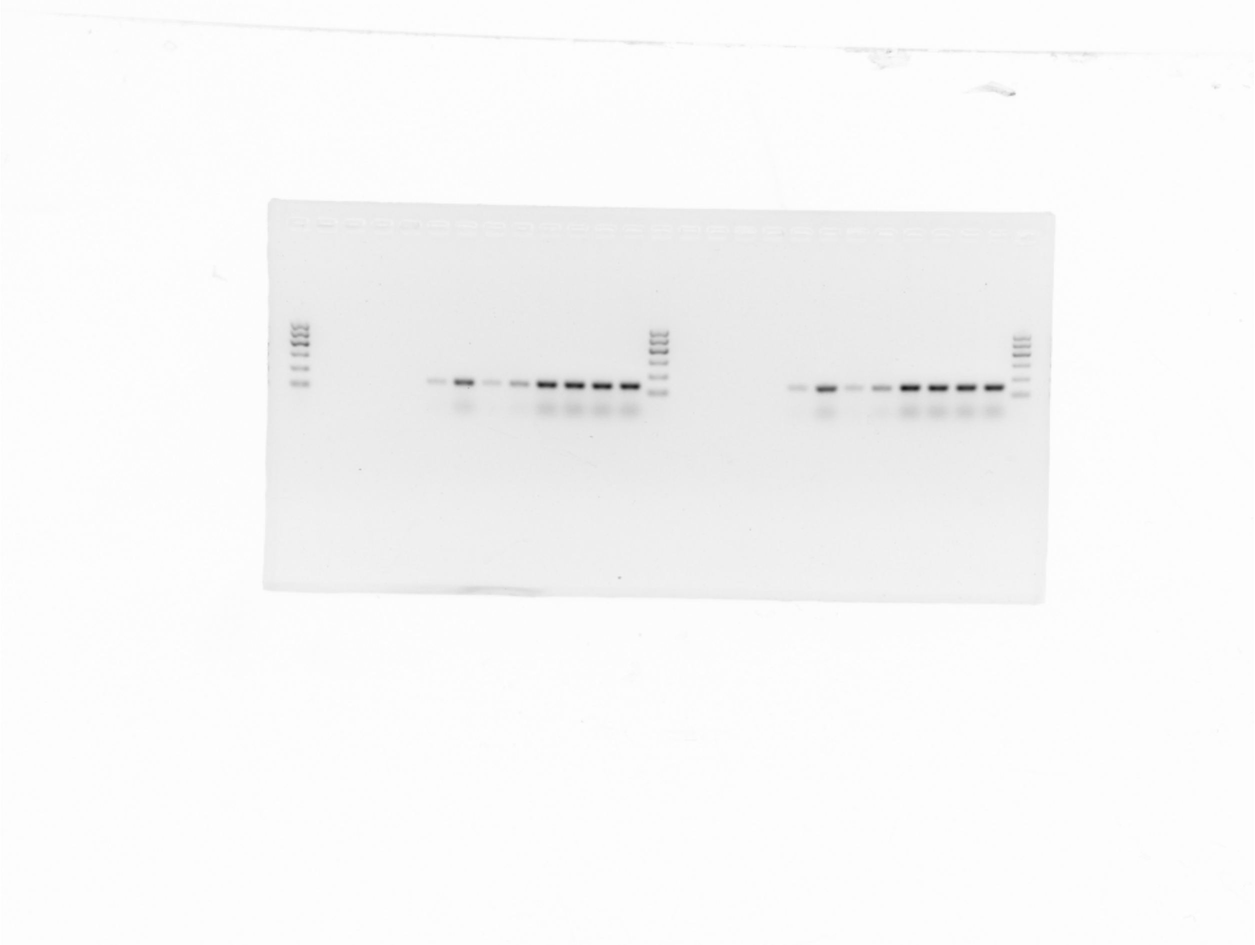

Full unedited gel for Supplemental Figure 23B

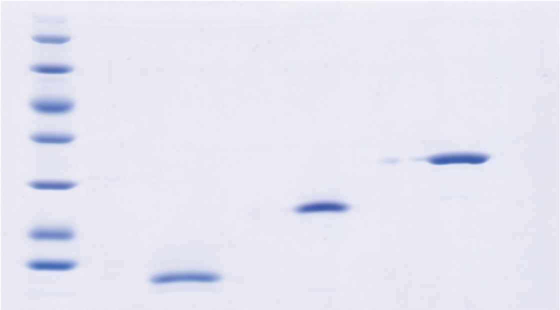

Full unedited gel for Supplemental Figure 24A

PPFIA4 (from lane 1 to lane 4)

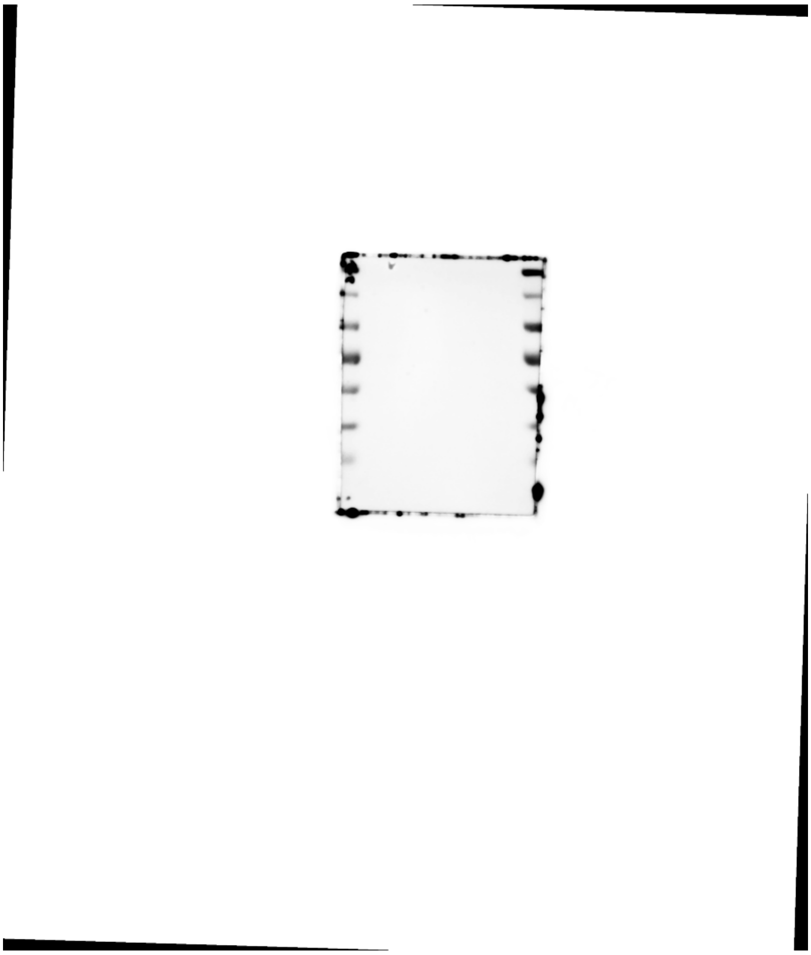

CASK (from lane 1 to lane 4)

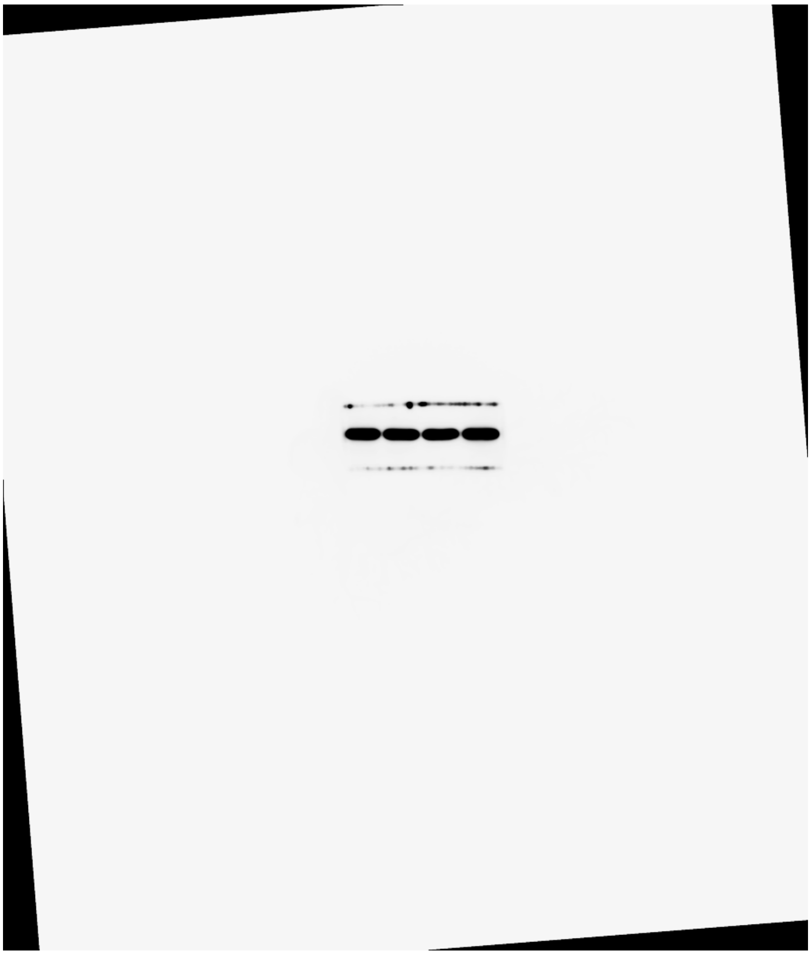

AKT1 (from lane 1 to lane 4)

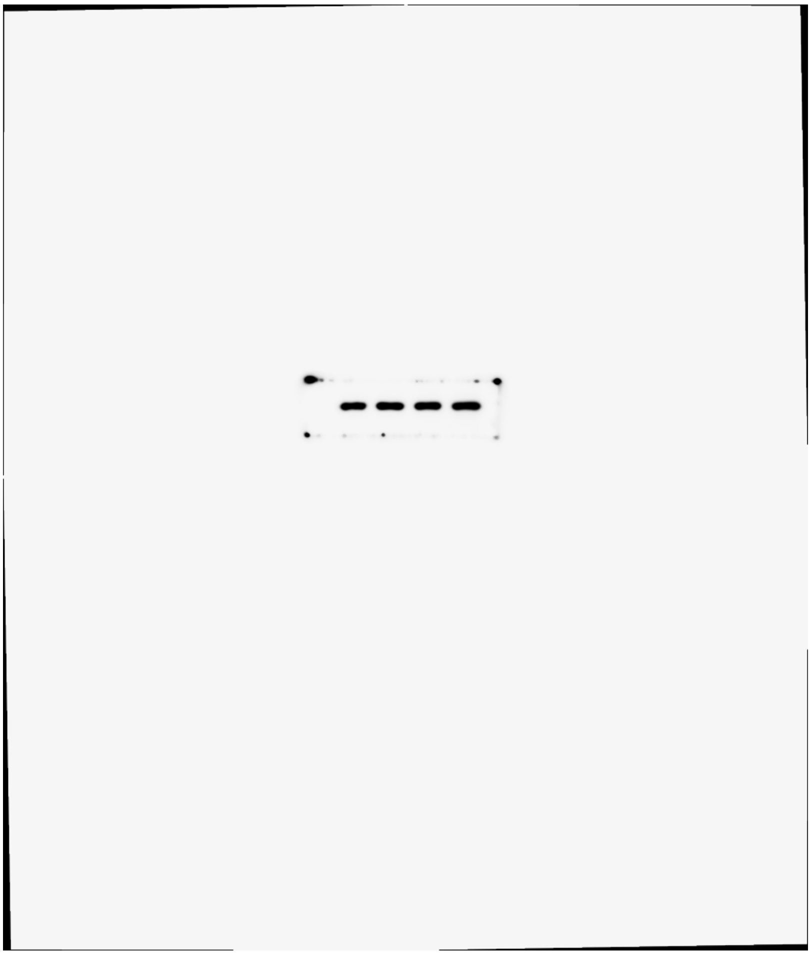

p-AKT1(Thr308) (from lane 1 to lane 4)

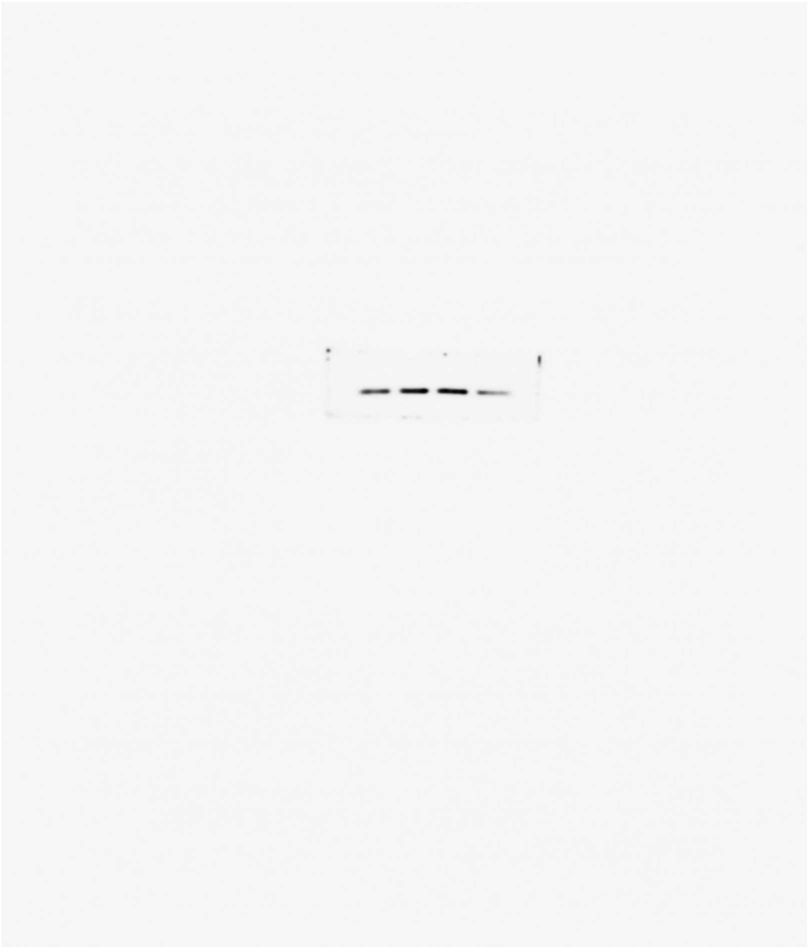

p-AKT1(Ser473) (from lane 1 to lane 4)

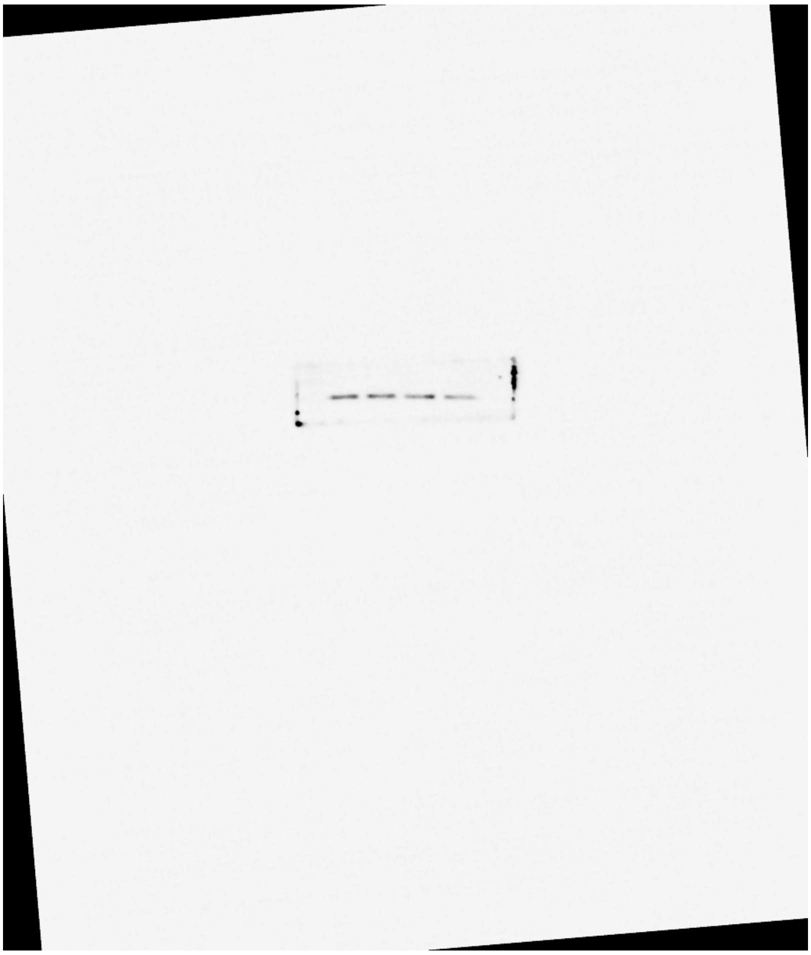

p65 (from lane 1 to lane 4)

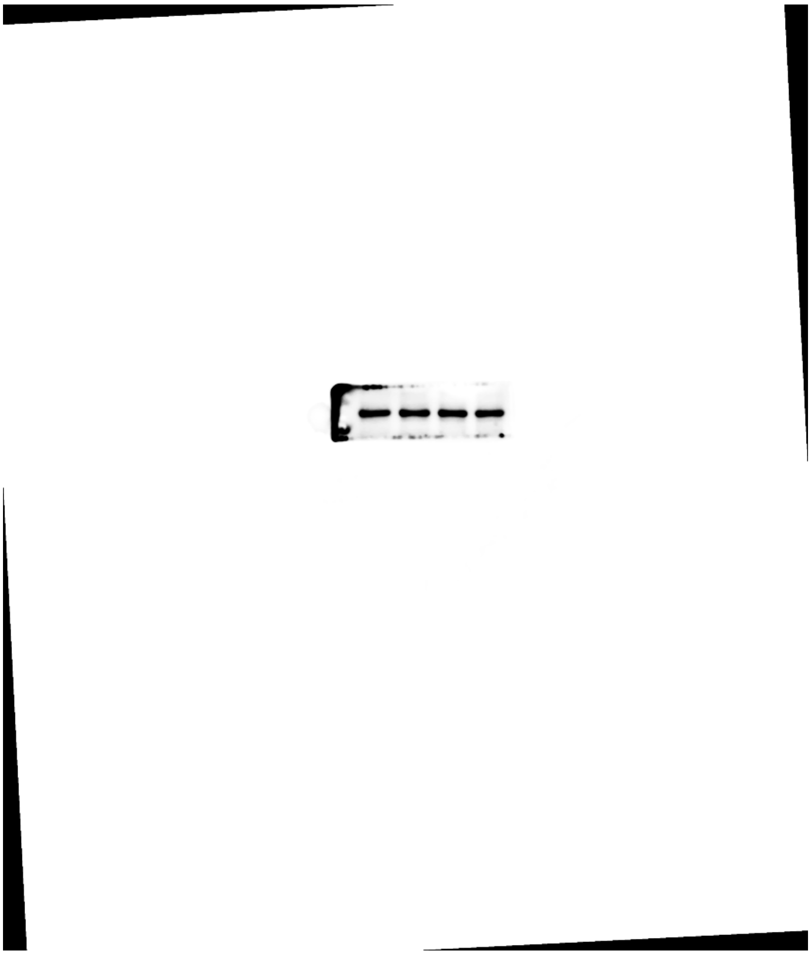

p-p65 (from lane 1 to lane 4)

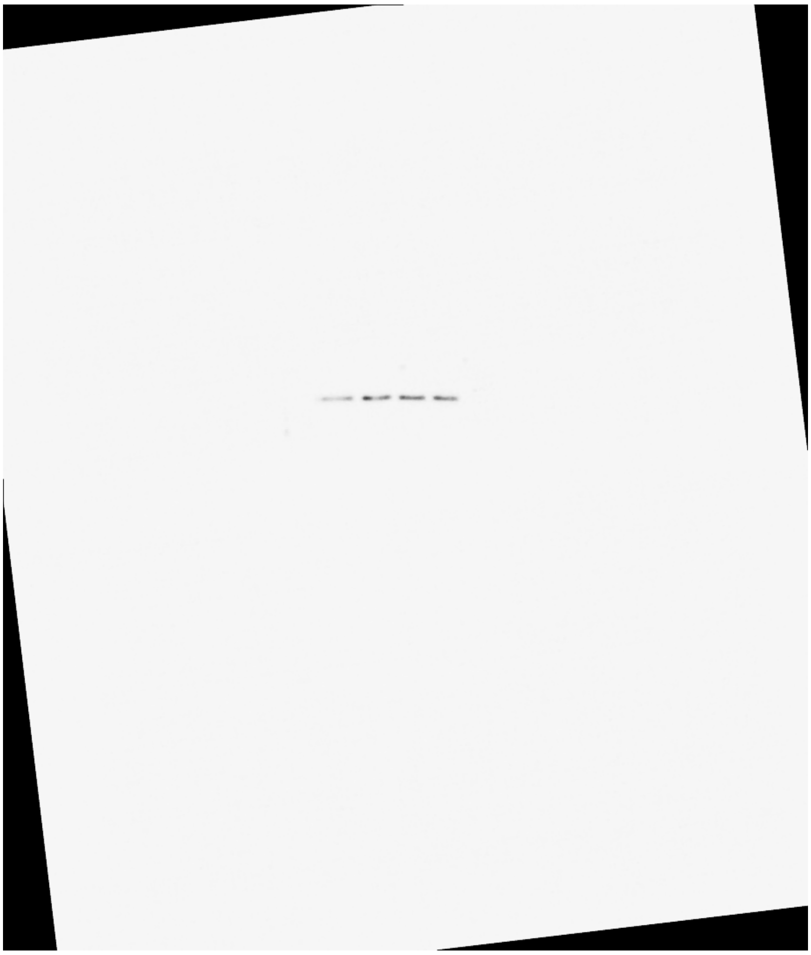

MMP1 (from lane 1 to lane 4)

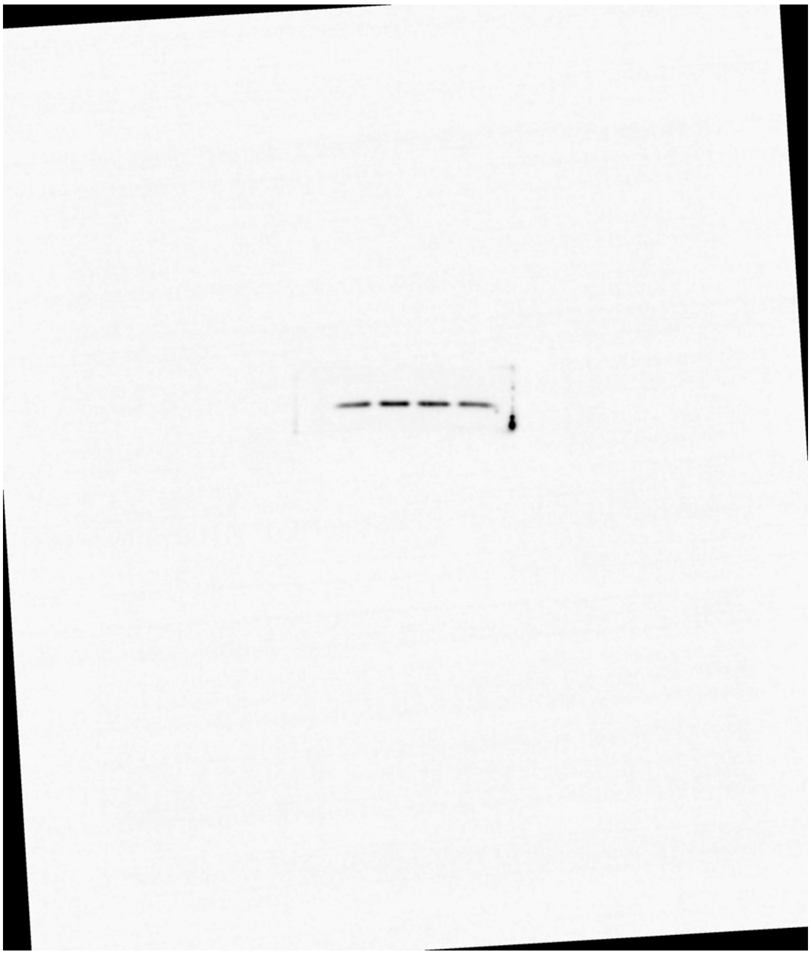

GAPDH (from lane 1 to lane 4)

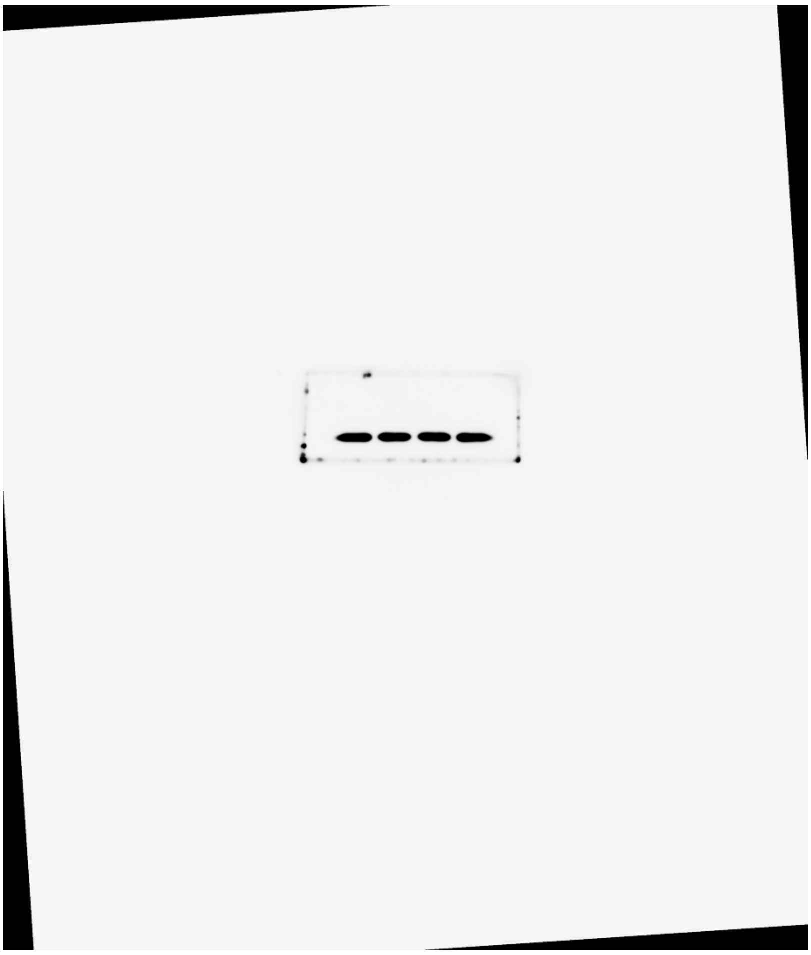

Full unedited gel for Supplemental Figure 24B

PPFIA4 (from lane 1 to lane 4)

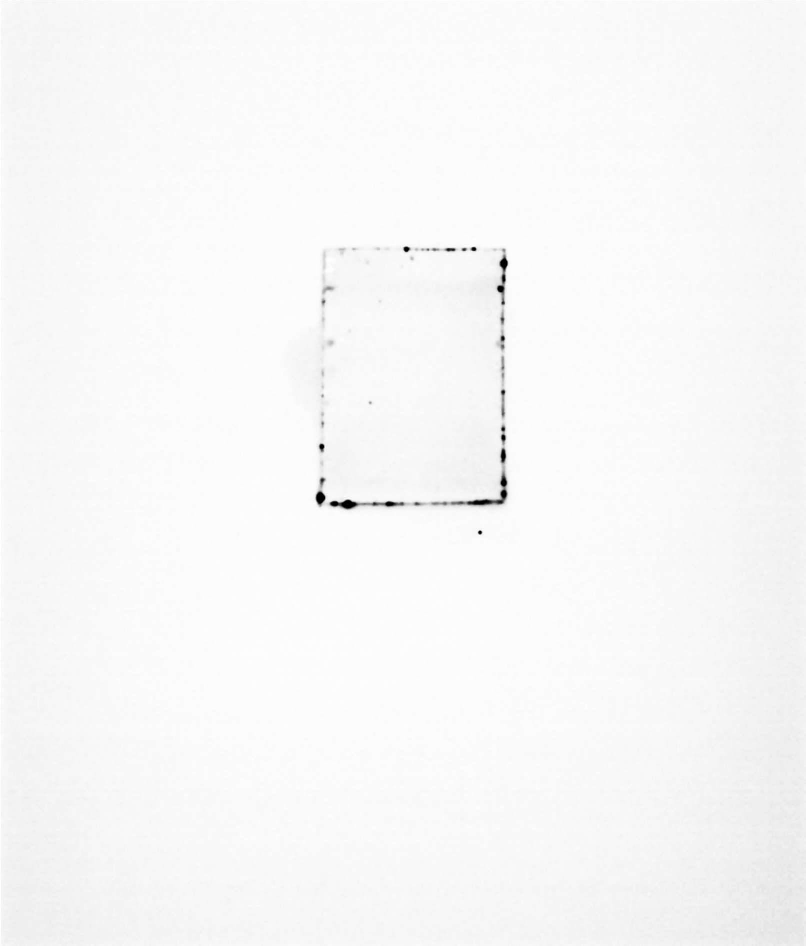

CASK (from lane 1 to lane 4)

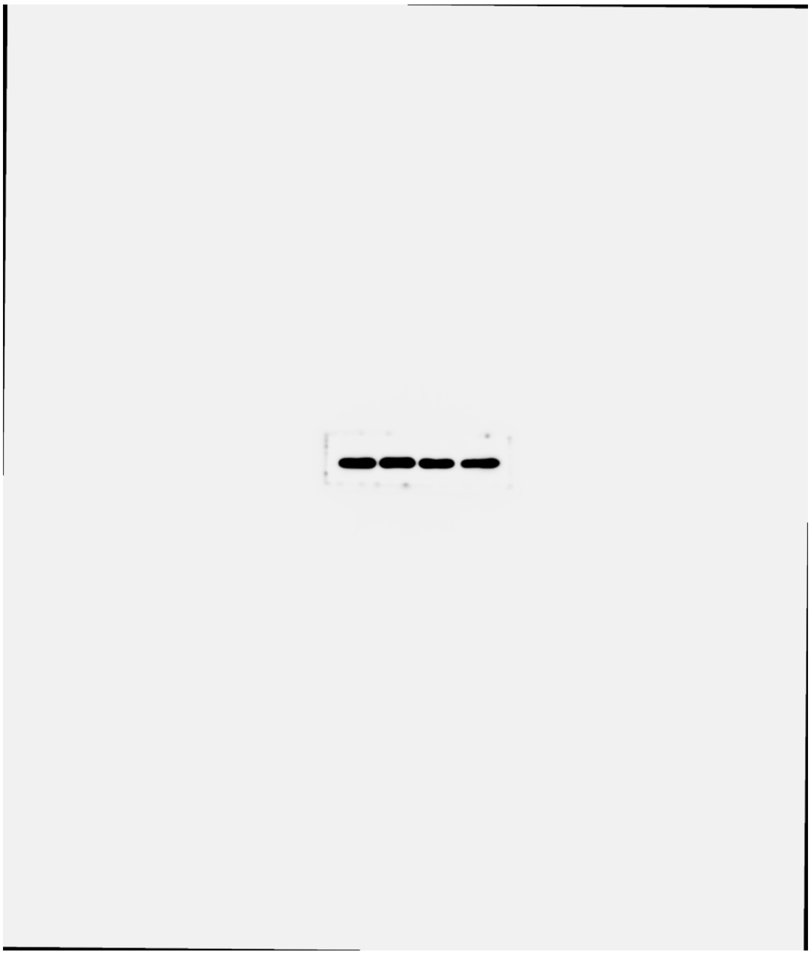

AKT1 (from lane 1 to lane 4)

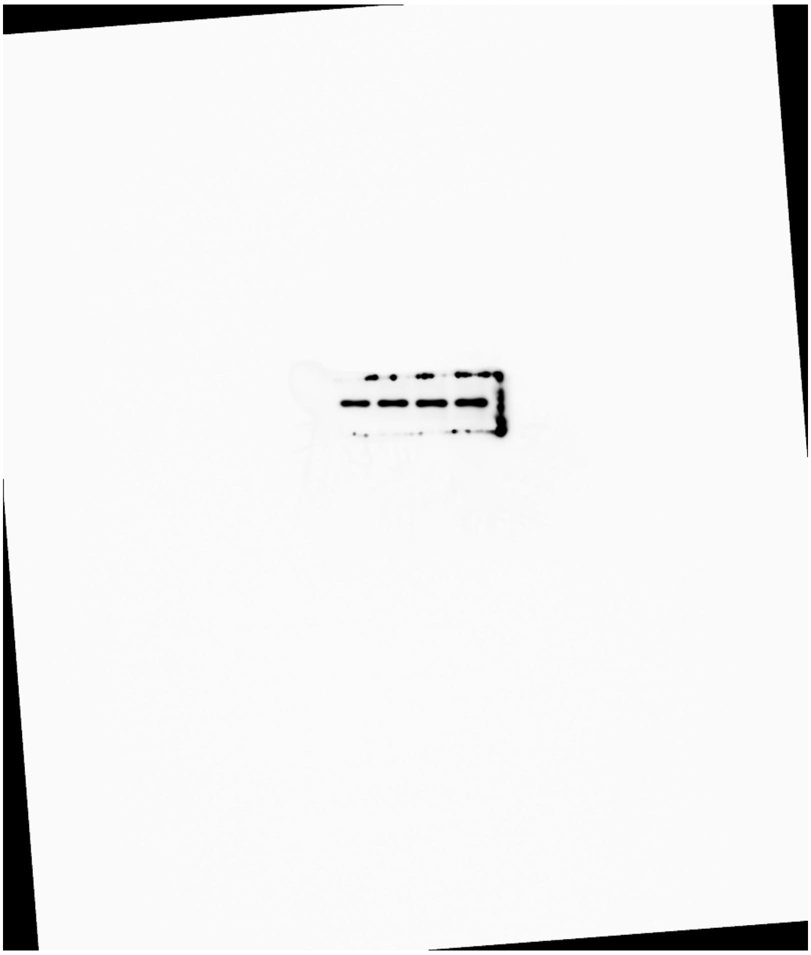

p-AKT1(Thr308) (from lane 1 to lane 4)

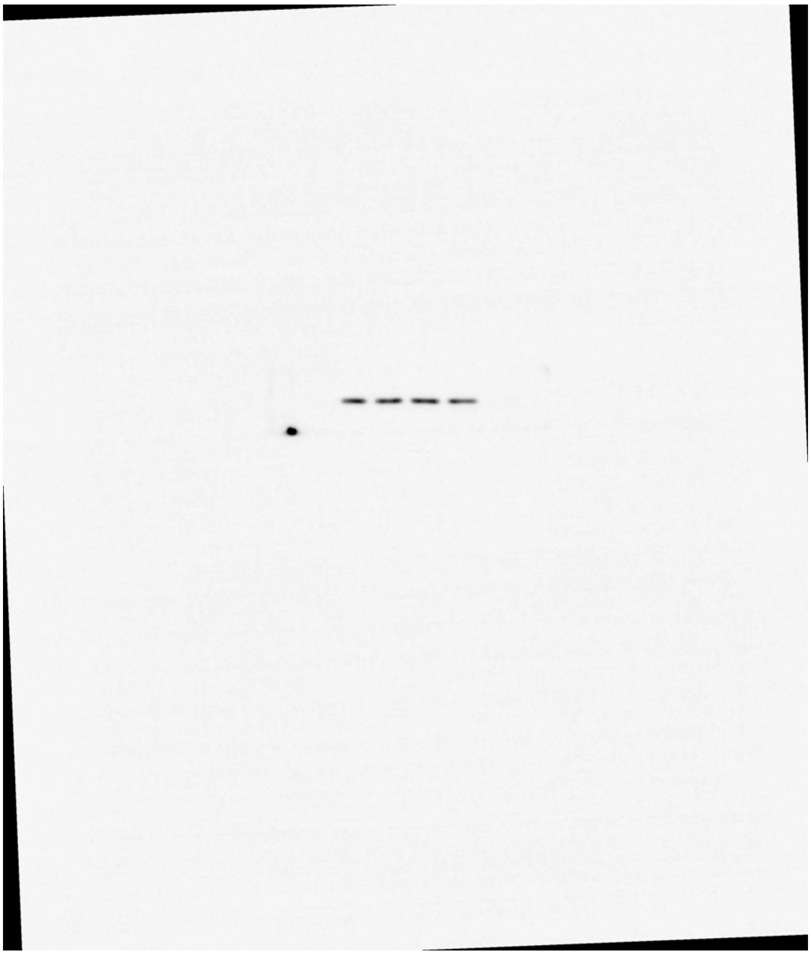

p-AKT1(Ser473) (from lane 1 to lane 4)

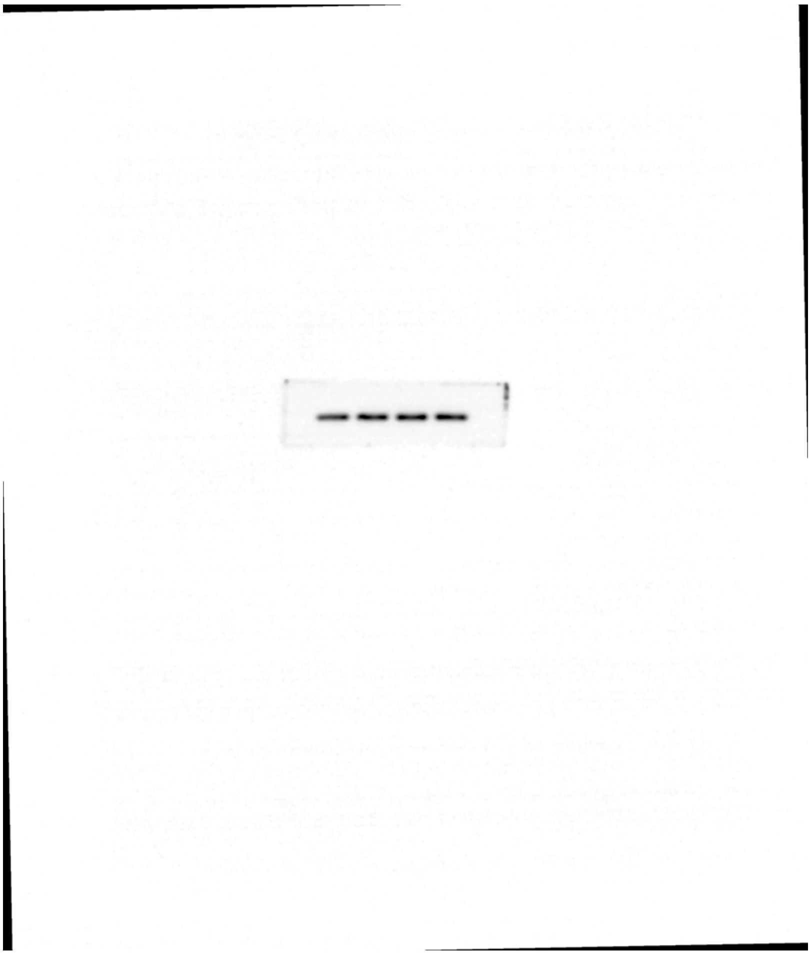

p65 (from lane 1 to lane 4)

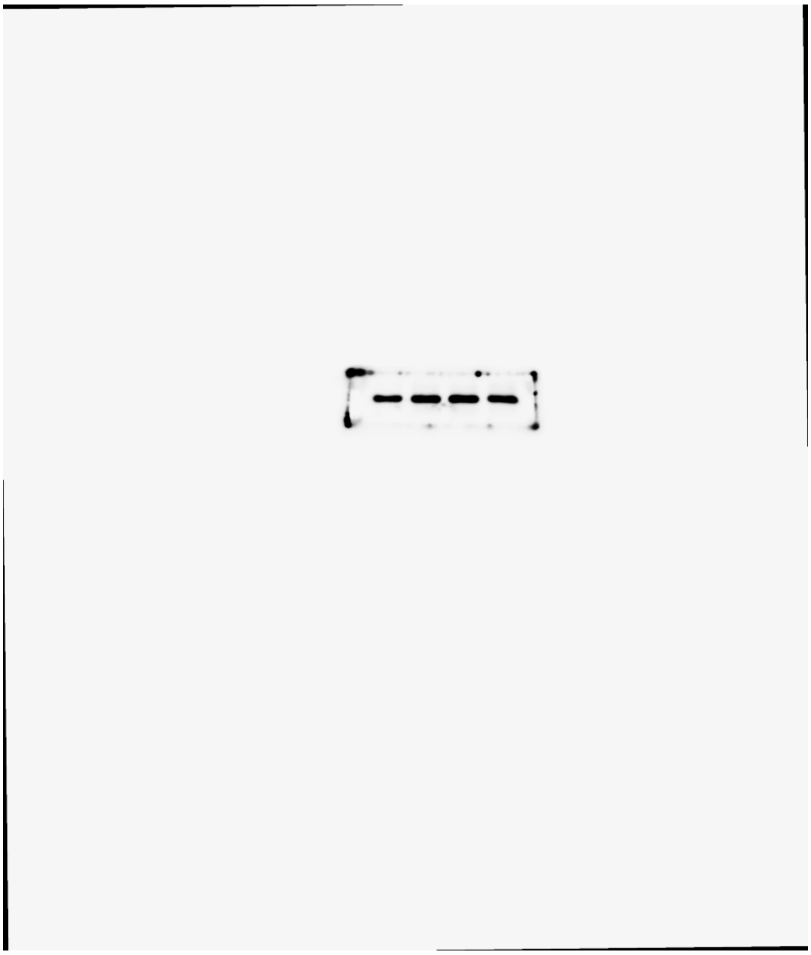

p-p65 (from lane 1 to lane 4)

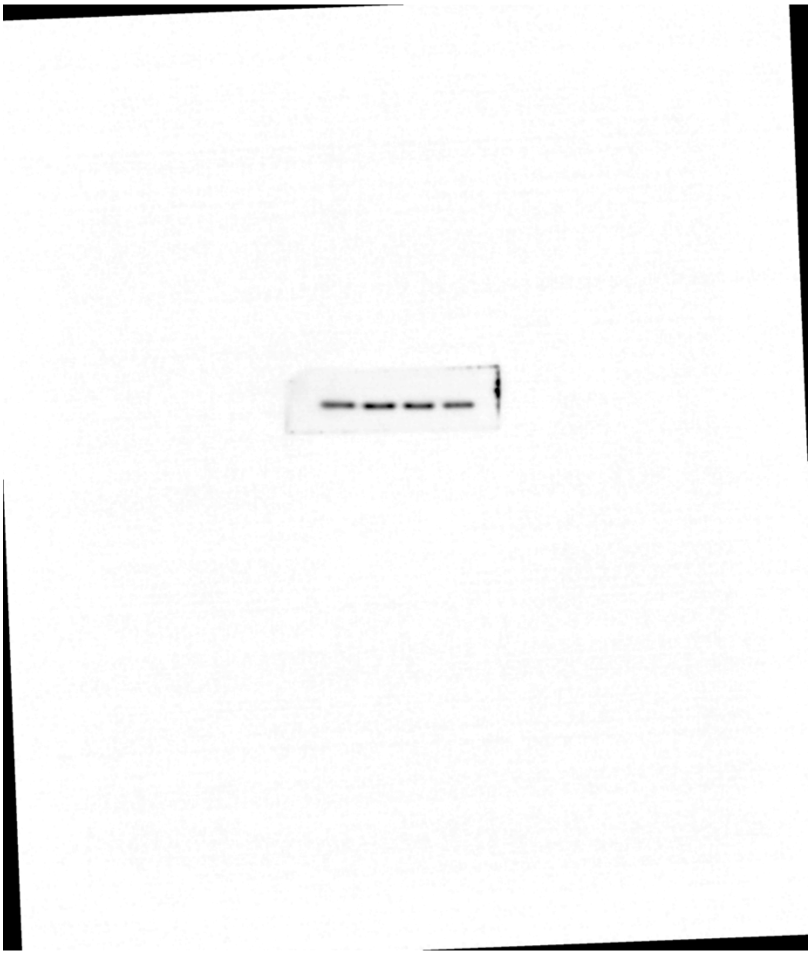

MMP1 (from lane 1 to lane 4)

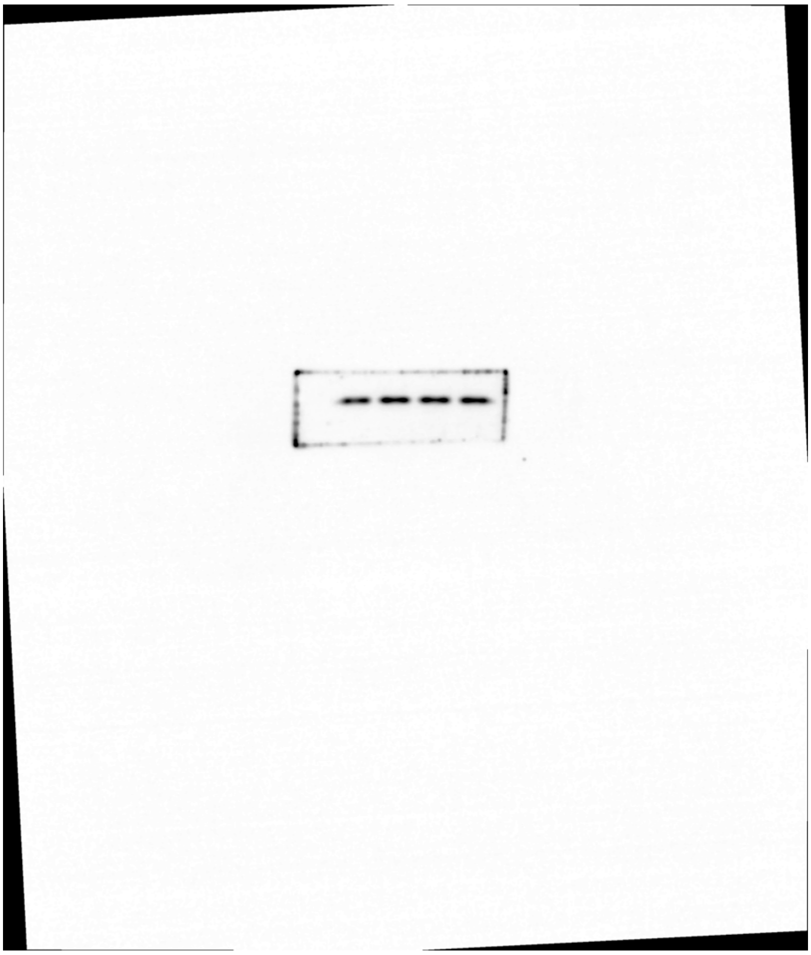

GAPDH (from lane 1 to lane 4)

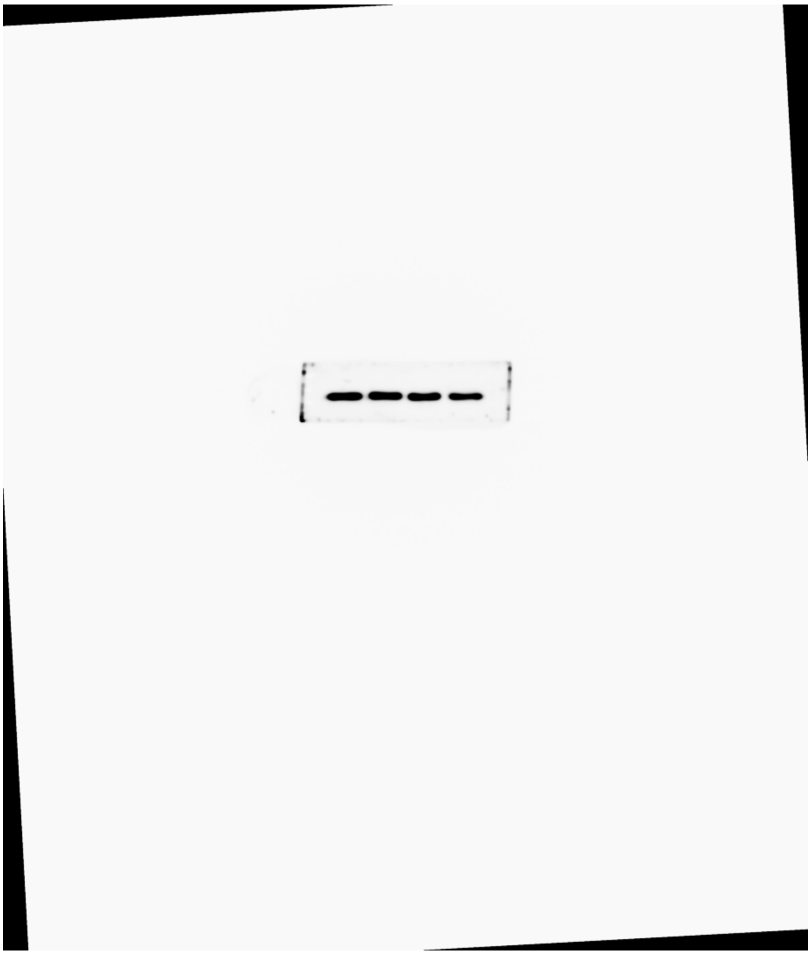

Full unedited gel for Supplemental Figure 24C

p-IRE1α (from lane 1 to lane 4)



IRE1α (from lane 1 to lane 4)

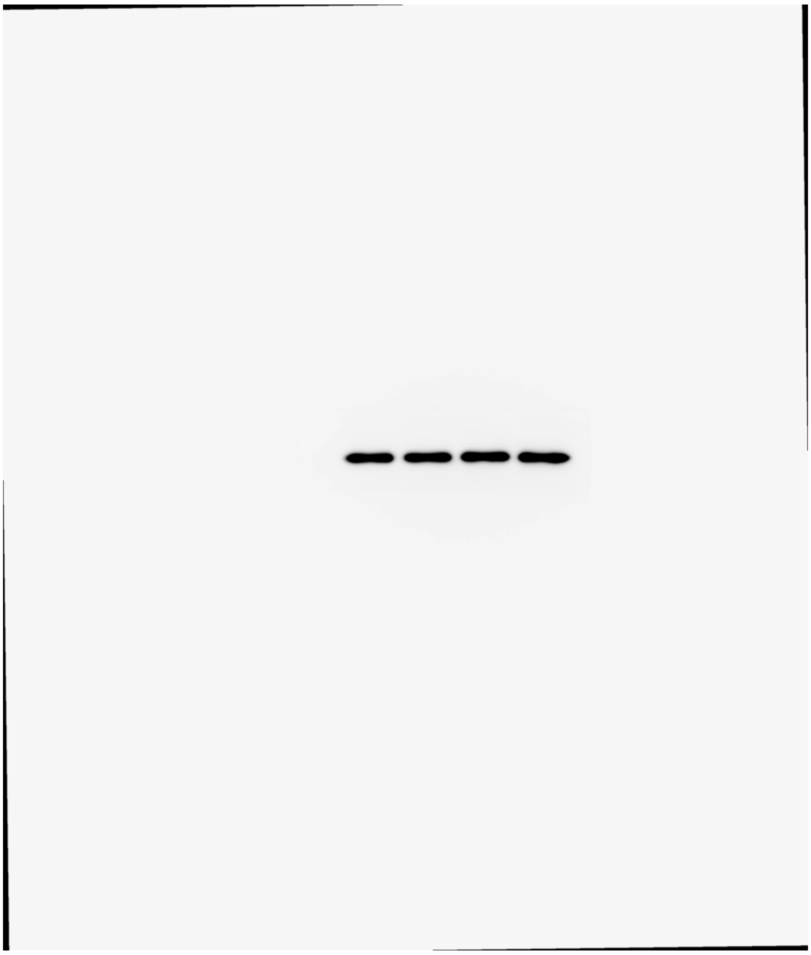

GAPDH (from lane 1 to lane 4)

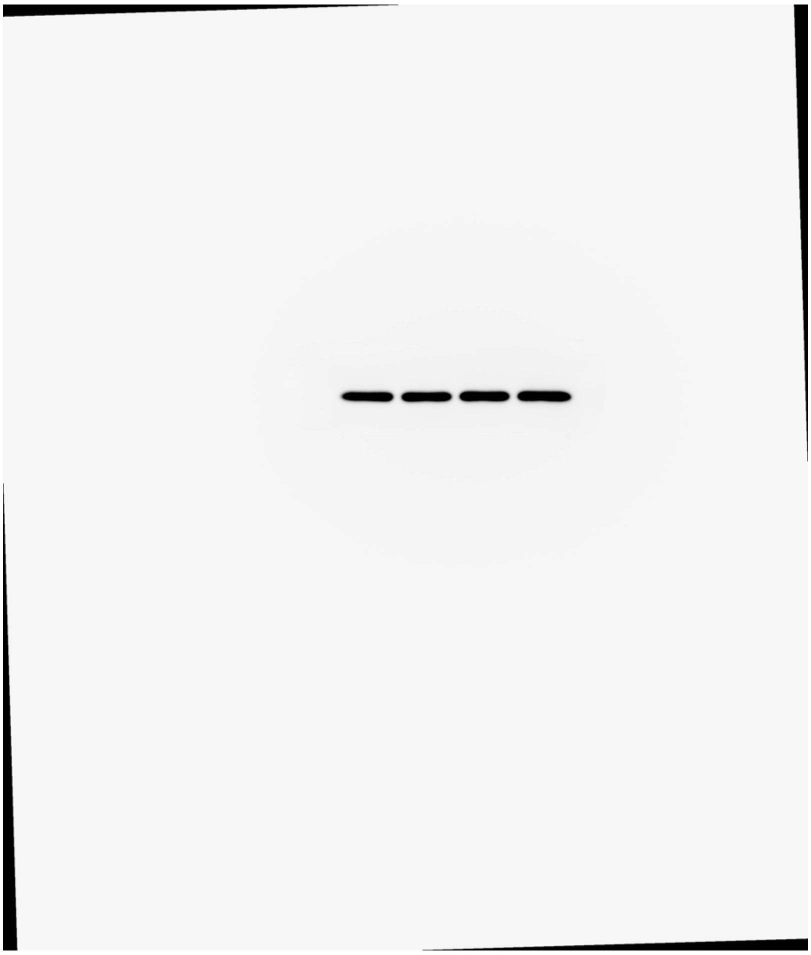

Full unedited gel for Supplemental Figure 24D

p-IRE1α (from lane 1 to lane 4)

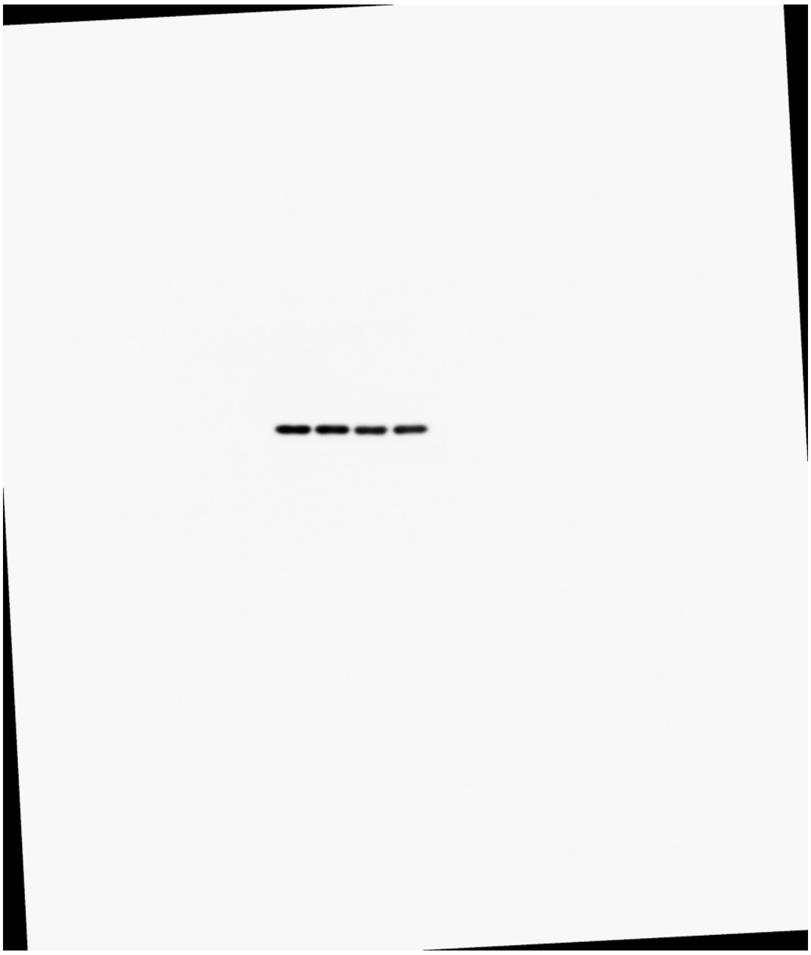

IRE1α (from lane 1 to lane 4)

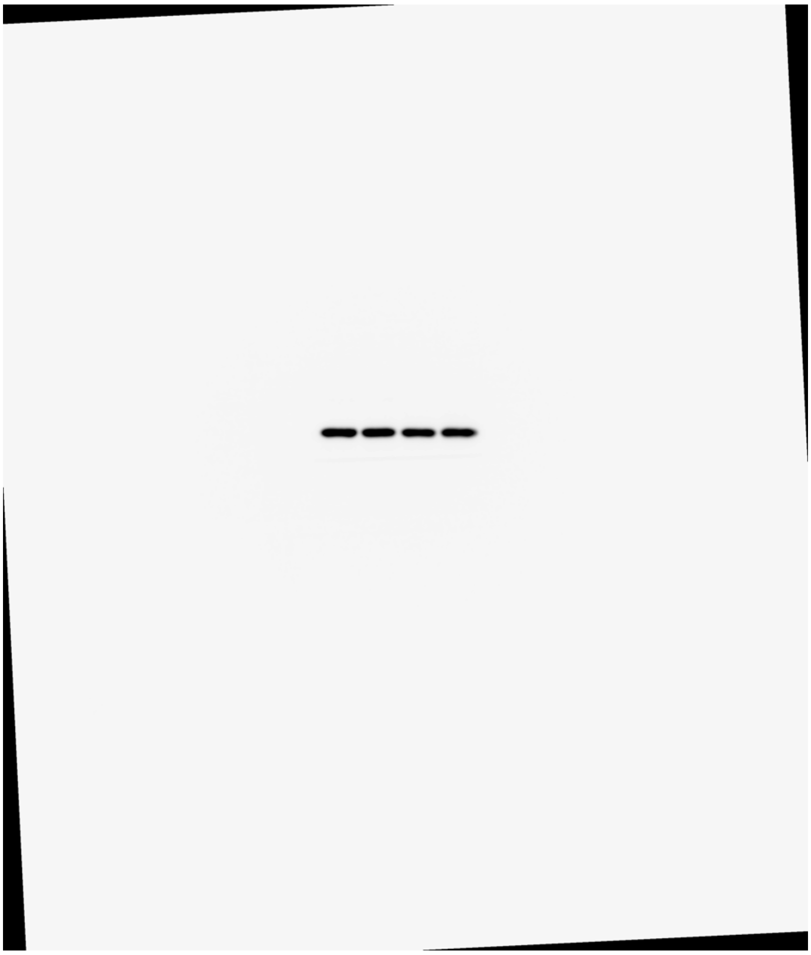

GAPDH (from lane 1 to lane 4)

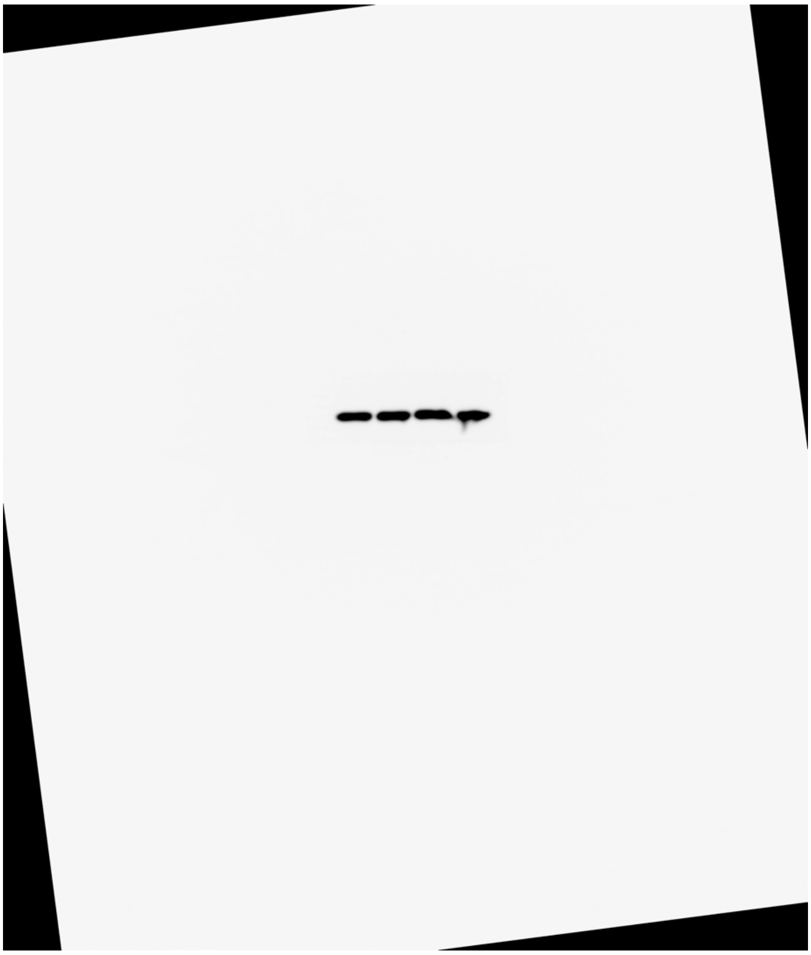

Supplement: Unedited blot and gel images [file jci-136-193848-s243.pdf]
