# Supplementary material for: Helicobacter pylori–induced PPFIA4 orchestrates immune network–promoting gastritis and gastric bacterial colonization
Source: J Clin Invest. 2026 May 1;136(9):e193848. doi: 10.1172/JCI193848 (PMC13132368; doi:10.1172/JCI193848)
Supplement: Supplementary file 3 [file jci-136-193848-s016.pdf]

Supplemental Methods

Patients and specimens

The gastric biopsy specimens and blood were collected from 50 uninfected donors, 131 *H. pylori*-infected patients and 32 gastric ulcer patients who underwent upper esophagogastroduodenoscopy for dyspeptic symptoms at XinQiao Hospital (Supplemental Table 1). *H. pylori* infection was determined by $[^{14}C]$ urea breath test and rapid urease test of biopsy specimens taken from the antrum, and subsequently confirmed by real-time PCR for 16s rDNA and serology test for specific anti-*H. pylori* antibodies (Abs). For isolation of human primary gastric epithelial cells (GECs), fresh non-tumor gastric tissues (at least 5-cm distant from the tumor site) were obtained from gastric cancer patients who underwent surgical resection and were determined as *H. pylori*-negative individuals as above described at Southwest Hospital. None of these patients had received chemotherapy or radiotherapy before sampling. Individuals with atrophic gastritis, hypochlorhydria, antibiotics treatment, autoimmune disease, infectious diseases and multi-primary cancer were excluded.

Antibodies and other reagents (Supplemental Table 2)

Mice

C57BL/6 *Ppfia4*$^{-/-}$ mice, C57BL/6 *Ppfia4*$^{flox/flox}$ mice, C57BL/6 *Cxcl3*$^{flox/flox}$ mice and C57BL/6 *Gif-Cre* mice were generated (Cyagen Biosciences, China). C57BL/6 *Il33*$^{-/-}$ mice and C57BL/6 *St2*$^{-/-}$ mice were kindly provided by Dr. Hong Zhou (Anhui Medical University, China). *Il33*$^{-/-}$*Ppfia4*$^{-/-}$ mice and their littermate control (wild-type, WT) mice were generated by cross-breeding *Il33*$^{-/-}$ and *Ppfia4*$^{-/-}$ mice. C57BL/6 *Il17a*$^{-/-}$ mice and C57BL/6 *Ifng*$^{-/-}$ mice were kindly provided by Dr. Richard A. Flavell (Yale University, USA). C57BL/6 *Il22*$^{-/-}$ mice were kindly provided by Dr. Wenjun Ouyang (Genentech, USA). C57BL/6 *Cxcr2*$^{-/-}$ mice were obtained from the Jackson Laboratory (Bar Harbor, USA). *Ppfia4* GEC-specific knockout mice (*Gif-Cre;Ppfia4*$^{flox/flox}$, now called *Ppfia4*$^{\Delta GEC}$) were generated by crossing *Ppfia4*$^{flox/flox}$ mice with *Gif-Cre* mice; *Ppfia4*$^{flox/flox}$ littermates were used as control. *Cxcl3* GEC-specific knockout mice (*Gif-Cre;Cxcl3*$^{flox/flox}$, now called *Cxcl3*$^{\Delta GEC}$) were generated by crossing *Cxcl3*$^{flox/flox}$ mice with *Gif-Cre* mice; *Cxcl3*$^{flox/flox}$ littermates were used as control. Only female mice were used in all experiments except for the chimeric experiments, in which male mice were also used, and were free of Abs specific for pathogenic murine viruses; negative for pathogenic bacteria, including *Helicobacter* spp., and parasites; and were maintained under specific pathogen free conditions in a barrier sustained facility and provided with sterile food and water (1).

Bacteria culture and infection of mice with bacteria

H. pylori NCTC 11637 (*cagA* positive) (*H. pylori*) and *cagA*-knockout mutant *H. pylori* NCTC 11637 (CagA- *H. pylori*) (kindly provided by Dr. Sasakawa (2, 3)) were grown in brain-heart infusion plates containing 10% rabbit blood at 37°C under microaerophilic conditions. The antibiotic-resistant *H. pylori* was developed as previously described (4), *H. pylori* NCTC 11637 was inoculated onto Columbia agar plates, and subsequently was stimulated with clarithromycin (CLA)/levofloxacin (LEV)/metronidazole (MET) to induce antibiotic resistance. For infecting mouse, bacteria were propagated in Brucella broth with 5 % fetal bovine serum (FBS) with gentle shaking at 37°C under microaerobic conditions. After culture for 1 d, live bacteria were collected and adjusted to 10^9 CFU/ml. The number of bacteria was determined by measuring the optical density at 600 nm (1 OD_{600} = 1×10^9 CFU/ml). The mice were fasted overnight and orogastrically inoculated twice at a 1 d interval with 3×10^8 CFU bacteria. *H. pylori* infection status and *H. pylori*-induced gastritis in murine experiments were confirmed using real-time PCR of *H. pylori* 16s rDNA, bacterial reisolation and quantitative culture, urease biopsy assays, Warthin-Starry staining and immunohistochemical staining for *H. pylori*, and evaluation of inflammation by haematoxylin and eosin (H&E) staining.

Generation of bone marrow (BM) chimera mice

The following BM chimeric mice were created: male *Il33*$^{-/-}$ BM→female *Ppfia4*$^{-/-}$ mice, and male *Ppfia4*$^{-/-}$ BM→female *Il33*$^{-/-}$ mice; or male WT BM→female *Il33*$^{-/-}$*Ppfia4*$^{-/-}$ mice, male *Il33*$^{-/-}$ BM→female *Il33*$^{-/-}$*Ppfia4*$^{-/-}$ mice, male *Ppfia4*$^{-/-}$ BM→female *Il33*$^{-/-}$*Ppfia4*$^{-/-}$ mice, and male *Il33*$^{-/-}$*Ppfia4*$^{-/-}$ BM→female *Il33*$^{-/-}$*Ppfia4*$^{-/-}$ mice; or male WT BM→female WT mice, male WT BM→female *Ppfia4*$^{-/-}$ mice, male *Ppfia4*$^{-/-}$ BM→female WT mice, and male *Ppfia4*$^{-/-}$ BM→female *Ppfia4*$^{-/-}$ mice. BM cells were collected from the femurs and tibia of donor mice by aspiration and flushing, and were suspended in PBS at the concentration of 5×10^7/ml. The BM in recipient mice was ablated with lethal irradiation (8 Gy). Then, the animals received intravenously 1.5×10^7 BM cells from donor mice in a volume of 300 μl sterile PBS under anaesthesia. Thereafter, the transplanted BM was allowed to regenerate for 8 weeks before subsequent experimental procedures. To verify successful engraftment and reconstitution of the BM in the host mice, genomic DNA was isolated from tail tissues of each chimera mouse 8 weeks after BM transplantation. Quantitative PCR was performed to detect the *Sry* gene present in the Y chromosome (primers seen in Supplemental Table 3) and mouse *β2-microglobulin* gene as an internal control. The chimeric rates were calculated on the assumption that the ratio of the *Sry* to

β2-microglobulin gene was 100% in female recipient mice. We confirmed that the chimeric rates were consistently higher than 90%. After BM reconstitution was confirmed, mice were infected with bacteria as described above.

Chemokine/MMP/Antibody/Kira6 administration

One day after infection with *H. pylori* as described above, WT mice were injected intraperitoneally with 25 μg of recombinant mouse CXCL3, or anti-mouse CXCL3, anti-mouse CXCR2 Abs or their control IgG (100 μg) and repeated every week until the mice were sacrificed 15 weeks p.i.. One day after infection with *H. pylori* as described above, WT mice or *Ppfia4*$^{flox/flox}$ littermates were injected intraperitoneally with anti-mouse MMP1 Abs or control IgG (100 μg), and *Ppfia4*$^{-/-}$ mice or *Ppfia4*$^{\Delta GEC}$ mice were injected intraperitoneally with recombinant mouse MMP1 (25 μg) or PBS control and repeated every week until the mice were sacrificed 15 weeks p.i.. One day after infection with *H. pylori* as described above, *Ppfia4*$^{\Delta GEC}$ mice and *Ppfia4*$^{flox/flox}$ littermates were injected intraperitoneally with kira6 (5 mg/kg) or vehicle control and repeated every week until the mice were sacrificed 15 weeks p.i.. Twelve weeks after infection with *H. pylori* as described above, *Ppfia4*$^{\Delta GEC}$ mice and *Ppfia4*$^{flox/flox}$ littermates were injected intraperitoneally with kira6 (5 mg/kg) or vehicle control and repeated every week until the mice were sacrificed 18 weeks p.i..

T cell adoptive transfer

One day before infection with *H. pylori*, *Ppfia4*$^{\Delta GEC}$ mice and *Ppfia4*$^{flox/flox}$ littermates were injected intravenously (1×10^6 cells/mouse) with purified spleen $CD4^+$ T cells (StemCell Technologies) from uninfected WT mice, or *H. pylori*-infected WT mice, *Il17a*$^{-/-}$ mice, *Il22*$^{-/-}$ mice or *Ifng*$^{-/-}$ mice (15 weeks p.i.). Then the recipient mice were infected with bacteria as described above and sacrificed for bacteria colonization evaluation 15 weeks p.i..

Evaluation of bacteria colonization by measuring *H. pylori*-specific 16s rDNA

The mice were sacrificed at the indicated times. The stomach was cut open from the greater curvature and half of the tissue was cut into four parts for RNA extraction, DNA extraction, protein extraction and tissue fixation, respectively. DNA of the biopsy specimens were extracted with QIAamp DNA Mini Kit. As previously described (5), *H. pylori* colonization was quantified by measuring *H. pylori*-specific 16s rDNA using specific primer and probe (Supplemental Table 3) by the TaqMan method. The amount of mouse β2-microglobulin

DNA in the same specimen was used to normalize the data. According to a previous study (6), the density of *H. pylori* was shown as the number of bacterial genomes per nanogram of host genomic DNA (7). Another half of stomach was used for isolation of single cells as described below. The isolated single cells were collected, sorted and analyzed by flow cytometry staining.

Evaluation of bacteria colonization by bacterial reisolation and quantitative culture

For assessment of *H. pylori* colonization by quantitative culture, weighed stomachs mechanically homogenized in Brucella broth, and homogenates were plated on brain-heart infusion plates containing 10% rabbit blood in serial dilutions. The plates were incubated for 5 days, and colonies were counted to determine the number of CFU per gram of stomach tissue.

Evaluation of inflammation

Mice were sacrificed at the indicated times. The greater curvature of the stomach was cut to perform H&E staining. The intensity of inflammation was evaluated independently by two pathologists according to previous established criteria (8, 9). Inflammatory scores for immune cell infiltration in the lamina propria of gastric mucosa were graded as follows: 1, mild multifocal (scattered clumps of two or three cells); 2, mild widespread (widespread scattering of cells across most of the region) or moderate multifocal (larger clumps of cells seen in a few fields per region); 3, mild widespread and moderate multifocal or severe multifocal (large infiltration of cells across whole width of mucosa); 4, moderate widespread; 5, moderate widespread and severe multifocal; 6, severe widespread.

Isolation of single cells from tissues

Fresh tissues were washed three times with Hank's solution containing 1% FBS, cut into small pieces, collected in RPMI 1640 containing 1 mg/ml collagenase IV and 10 mg/ml DNase I, and then mechanically dissociated by using the gentle MACS Dissociator (Miltenyi Biotec). Dissociated cells were further incubated for 0.5-1 h at 37°C under continuous rotation. The cell suspensions were then filtered through a 70-μm cell strainer (BD Labware).

Gastric organoid cultures

For mouse gastric organoid, gastric tissue single-cell suspensions isolated from uninfected mice were mixed

in Matrigel and cultured in 24-well plates. As previously described (10), the mixture was coagulated in a 37°C incubator for 10 minutes before adding into the before adding culture medium containing recombinant mouse growth factors (Wnt3a (100 ng/ml), Noggin (100 ng/ml), R-Spindin-1 (1 μg /ml), EGF (50 ng/ml), FGF-10 (100 ng/ml), Gastrin I (10 nM)) and various value-added components (1×N-2 Supplement, 10% GlutaMAX Supplement to Advanced DMEM/F12). Special notes were to supplement the culture medium with Y-27632 (10 mM) in the first two days of cultivation to avoid loss of nest cell apoptosis. The culture medium was changed once every 3-5 days, and the passage of organoids was performed in a 1:4 ratio depending on the growth situation after 2 weeks. For human gastric organoid, gastric non-tumor (at least 5 cm distant from the gastric cancer site) tissues were obtained from *H. pylori*-uninfected gastric cancer donors who underwent surgical resections. As previously described (11), the fresh gastric mucosa layer of the collected tissues was separated and cut into small pieces (2 mm^3) and then digested with collagenase and hyaluronidase for 1 h at 37°C. The supernatant was filtered with a 100 μm cell filter and resuspended in appropriate pre-cooled Matrigel, and was added to the center of each well of 12-well plates until the Matrigel was completely solidified. Then the pre-prepared medium containing various growth factors (Biogenous) was added. The culture medium was changed once every 3-5 days, and the passage of organoids was performed in a 1:4 ratio depending on the growth situation after 2 weeks.

Cell/tissue/organoid culture and stimulation

Primary GECs were purified from gastric tissue single-cell suspensions from uninfected donors or mice with a MACS column purification system using anti-human or mouse CD326 magnetic beads (Miltenyi Biotec). The sorted primary GECs were used only when their viability was determined >90% and their purity was determined >95%. The cells were cultured in complete RPMI 1640 medium supplemented with 10% FBS in a humidified environment containing 5% CO_2 at 37 °C. Human GEC lines (AGS cells, GES-1 cells, HGC-27 cells and SGC-7901 cells) were obtained from American Type Culture Collection (ATCC, Manassas, VA, USA). Human GEC lines were infected with *H. pylori* or CagA- *H. pylori* at a multiplicity of infection (MOI) of 100 for 24 h. Human and mouse primary GECs, primary gastric mucosa tissues and gastric organoids were infected with *H. pylori* or CagA- *H. pylori* (MOI=100) for 24 h, or infected with *H. pylori* (MOI=100) in the presence or absence of IL-33 (100 ng/ml) for 24 h. AGS cells were also infected with *H. pylori* or CagA- *H. pylori* (MOI=100) in the presence or absence of IL-33 (100 ng/ml) for 24 h, or infected with *H. pylori* (MOI=100) in the presence or absence of IL-33 (50, 100, 200 ng/ml) for 24 h, or infected with *H. pylori* (MOI=100) in the presence or

absence of IL-1β, IL-2, IL-3, IL-4, IL-6, IL-9, IL-10, IL-12, IL-17A, IL-17F, IL-22, IL-23, IL-33, TNF-α, TGF-β, IFN-γ, G-CSF, M-CSF or GM-CSF (100 ng/ml) for 24 h, or infected with *H. pylori* (MOI=100) and/or IL-33 (100 ng/ml) in the presence or absence of neutralizing Abs against IL-33 (20 μg/ml) and/or ST2 (20 μg/ml) for 24 h. Additionally, AGS cells were pre-treated with *ST2* siRNA (si*ST2*) or non-specific control siRNA (siNC) (40 nM) for 24 h, then were infected with *H. pylori* (MOI=100) in the presence or absence of IL-33 (100 ng/ml) for 24 h; primary mouse GECs from WT mice and *St2*$^{-/-}$ mice were infected with *H. pylori* (MOI=100) in the presence or absence of IL-33 (100 ng/ml) for 24 h. AGS cells, primary GECs or primary gastric mucosa tissues were also infected with *H. pylori* at different MOI (24 h) or at the indicated time points (MOI=100). In some cases, AGS cells were transfected with plasmids *cagA*-pcDNA3.1 or pcDNA3.1 (control vector) by using lipofectamine 2000 according to the manufacturer's protocols for 48 h. For inhibition experiments, AGS cells were pre-treated with PP2 or T-5224 (20 μM) for 2 h. For PPFIA4 function experiments, *PPFIA4* knockout AGS cells were generated with *PPFIA4* sgRNA (sg*PPFIA4*) or non-specific control sgRNA (sgNC) by Sangon Biotech (Shanghai, China). For CASK function experiments, AGS cells were pre-treated with *CASK* siRNA (si*CASK*) or siNC (40 nM) for 24 h. For transwell assays, AGS cells were added to the lower chamber, and *H. pylori* (MOI=100) were placed into the lower or the upper chambers of transwells (0.4-μm pore) and then incubated for 24 h. AGSs were also treated with MMP1 (1 μg/ml) for 24 h. After co-culture, the cells were collected for real-time PCR and western blot, and the culture supernatants were harvested for ELISA.

Chemotaxis assay

Human (CD45$^+$CD11b$^+$CD15$^+$CD66b$^+$HLA-DR$^-$) or mouse (defined as described in Supplemental Figure 8A) G-MDSCs from blood of *H. pylori*-infected donors or *H. pylori*-infected WT mice (15 weeks p.i.) were sorted by fluorescence activating cell sorter (FACS) (FACSAria III; BD Biosciences). sg*PPFIA4* or sgNC-modified AGS cells were stimulated with *H. pylori* or CagA- *H. pylori* (MOI=100) for 24 h. The culture supernatants were collected and used as source of chemoattractants in a human G-MDSC chemotaxis assay. In another set of experiments, mouse primary GECs from WT mice and *Ppfia4*$^{-/-}$ mice were purified from gastric tissue single-cell suspensions with a MACS column purification system using anti-mouse CD326 magnetic beads (Miltenyi Biotec), and then stimulated with *H. pylori* or CagA- *H. pylori* (MOI=100) for 24 h. The culture supernatants were collected and used as source of chemoattractants in a mouse G-MDSC chemotaxis assay. In a chemotaxis assay, sorted cells (1×10^5) were transferred into the upper chambers of transwells (5-μm pore). CXCL3 (100 ng/ml) and culture supernatants from various cultures were placed in the lower chambers.

After 6 h culture, migration was quantified by counting cells in the lower chamber and cells adhering to the bottom of the membrane. In some cases, blocking Abs for CXCL3 (20 μg/ml) or control IgG (20 μg/ml) were added into culture supernatants, and blocking Abs for CXCR2 (20 μg/ml) or control IgG (20 μg/ml) were added into cell suspensions and incubated for 2 h before chemotaxis assay.

In vitro T-cell culture system

Purified mouse spleen $CD4^+$ T cells from uninfected or *H. pylori*-infected WT mice (15 weeks p.i.) were labeled with carboxyfluorescein succinimidyl ester (CFSE) and co-cultured (1×10^5 cells/well) with FACS-sorted gastric G-MDSCs from *H. pylori*-infected WT mice (15 weeks p.i.) at 2:1 ratio in 200 μl RPMI 1640 medium containing recombinant mouse IL-2 (20 IU/ml), anti-CD3 (2 μg/ml), and anti-CD28 (1 μg/ml) Abs. After a 5-d incubation, cells were collected and analyzed by intracellular cytokine staining, and the culture supernatants were harvested for ELISA.

Luciferase reporter assay

Promoter constructs containing the region from -2000 to 1000 of the *PPFIA4* gene were amplified from human genomic DNA by PCR. The amplified full-length or fragments were cloned into the pGL3-basic vector respectively by Sangon Biotech (Shanghai, China). The constructs containing the region of genes of transcription factors were amplified from human genomic DNA by PCR. The amplified fragments were cloned into the pcDNA3.1-basic vector respectively by Sangon Biotech (Shanghai, China). For luciferase reporter assay, cells were seeded in 24-well plates, and were transfected when reaching approximately 80% confluence with the constructed luciferase reporter vector. Lipofectamine 2000 was used to transfect AGS cells according to the manufacturer's protocols. Luciferase activity was measured to assess promoter activity after *H. pylori* or CagA- *H. pylori* infection (MOI=100) (pre-treated with or without T-5224) for 24 h, or after *cagA*-pcDNA3.1 plasmid transfection (pre-treated with or without T-5224) for 48 h by the Dual-Luciferase Reporter assay following the manufacturer's protocol. Luciferase activity was normalized to Renilla luciferase activity.

Electrophoretic mobility shift assay (EMSA)

The AP1 binding site of *PPFIA4* gene was predicted by using the PROMO tool V.8.3 of TRANSFAC (Beverly, MA, USA). The sequences were extended by 13 bases at the upstream and downstream of the biding sites,

and then labeled by biotin at the 5' end and synthesized by Sangon Biotech (Shanghai, China) as EMSA detection probes (Supplemental Table 5). The AP1 protein was purchased from Abcam. The probe without biotin labeled was regarded as competitor probe. The EMSA were performed using LightShift Chemiluminescent EMSA Kit (Thermo Scientific) according to the manufacturer's instructions.

Chromatin Immunoprecipitation (ChIP)

AGS cells were infected with *H. pylori* or CagA- *H. pylori* (MOI=100) (pre-treated with or without T-5224) for 24 h, or transfected with *cagA*-pcDNA3.1 (pre-treated with or without T-5224) for 48 h. AGS cells stably expressing PPFIA4-Flag were established by Sangon Biotech (Shanghai, China) and then cultured (pre-treated with or without BAY 11-7082). The cells were then treated at room temperature for 10 min with 1% formaldehyde in cell culture media. Glycine (11 % in media) solution was then gently mixed in at room temperature for 5 min to terminate cross-linking. The cells were washed twice with ice-cold PBS and palleted at 3000g for 5 min. Membrane Extraction Buffer containing protease/phosphatase inhibitors was then added to each palleted sample. The cell lysates were pulse-sonicated on ice; supernatants containing the digested chromatin were collected into two tubes for input and immunoprecipitation. Anti-c-Jun Abs, anti-p65 Abs or corresponding control IgG were added and IP reactions conducted overnight at 4°C with agitation. ChIP grade protein A/G magnetic beads were then added to each IP reaction. Two hours later beads were collected, washed, bounded IP materials eluted with 5 M NaCl containing 20 μg/ml Proteinase K. The cross-linking was reversed by heating up to 65°C for 1.5 h and DNA was purified. Purified DNA samples were analyzed by PCR with designed primers (Supplemental Table 6).

Generation of AGS cells expressing the Flag-tagged PPFIA4 (PPFIA4-Flag)

The full length of human PPFIA4 (NM_001304331.2), with the Flag tag just behind the start codon, was chemically synthesized and inserted into the pLVX-CMV7-EGFP-Puro lentivirus vector at the two restriction sites XhoI and BamHI. The above reconstructed plasmids were transiently transfected into the AGS cells, and immunoprecipitation (IP) and western blotting were applied to confirm its over-expression. Briefly, the adherent cells were lysed by pre-cold IP dilution buffer (20 mM Tris-HCl, 2 mM EDTA, 1% Triton-X100, 150 mM NaCl) supplemented with PMSF and Protein inhibitor complex (PIC) on ice. The cellular debris was got rid of by centrifugation at 12000 rpm at 4°C for 10 min and the supernatant was maintained for the subsequent IP. In detail, the total cell lysis was equally divided into two parts which were incubated with mouse anti-Flag

monoclonal Ab, or mouse IgG isotype respectively. After gently agitated at 4°C overnight, pre-treated Pierce™ Protein G Magnetic beads were added into the lysate samples respectively and agitated at 4°C for 4 h. Next, the immunoprecipitated protein complexes were enriched using a magnetic separator, collected in Elution buffer (10% SDS, 0.5M EDTA, 1M Tris-HCl) and validated by western blotting.

Mass spectrometry (MS) analysis

The samples were sent to Sangon Biotech (Shanghai, China) for MS analysis. In brief, the sample was digested in-gel, then was analyzed by on-line nanospray liquid chromatography-tandem mass spectrometry (LC-MS/MS) on Q Exactive™ HF mass spectrometer (Thermo Fisher Scientific, USA) coupled to an EASY-nanoLC 1000 system (Thermo Fisher Scientific, USA). The peptides (3 μl) were loaded (analytical column: Acclaim PepMap C18, 75 μm x 25 cm) and separated with a 60 min gradient. The column flow rate was maintained at 300 nl/min with the column temperature controlled at 40°C. The electrospray voltage of 2 kV versus the inlet of the mass spectrometer was used. The mass spectrometer was run under data dependent acquisition mode, and automatically switched between MS and MS/MS mode. Tandem mass spectra were processed by PEAKS Studio version X+ (Bioinformatics Solutions Inc., Waterloo, Canada). PEAKS DB was set up to search the uniprot_homo_sapiens_201907 database (ver 201907, 20414 entries) assuming trypsin as the digestion enzyme.

Immunoprecipitation (IP) assay

Immunoprecipitation assay was performed using a Pierce™ Classic Magnetic IP/Co-IP Kit following the manufacturer's protocol. Firstly, whole-cell extracts of AGS cells expressing PPFIA4-Flag were lysed in IP Lysis/Wash Buffer in the presence of protease inhibitor; after centrifugation for 10 min at 13000 g, supernatants were collected and incubated with anti-Flag Abs, anti-CASK Abs or corresponding control IgG. Secondly, AGS cells were transfected with constructs containing the different region of PPFIA4-Flag or constructs containing the different region of CASK-HA for 24 h, whole-cell lysates and supernatants of these cells were collected as described above, and then incubated with anti-Flag Abs or with anti-HA Abs. Thirdly, sg*PPFIA4*-modified AGS cells were transfected with *PPFIA4*-Flag-pLVX or NC-Flag-pLVX for 24 h, whole-cell lysates and supernatants of these cells were collected as described above, and then incubated with anti-CASK Abs or corresponding control IgG. Fourthly, sg*PPFIA4*-modified AGS cells were transfected with si*CASK* or siNC (40 nM) for 24 h, then were transfected with *PPFIA4*-Flag-pLVX or NC-Flag-pLVX for 24 h,

whole-cell extracts of these cells were lysed and supernatants were collected as described above, and then incubated with anti-Flag Abs or corresponding control IgG. Fifthly, AGS cells expressing PPFIA4-Flag were pre-treated with kira6 (100 nM) or vehicle for 2 h, whole-cell lysates and supernatants of these cells were collected as described above, and then incubated with anti-Flag Abs or corresponding control IgG. After overnight incubation, the protein A/G magnetic beads (washed three times with IP Lysis/Wash Buffer) were incubated with total cell extracts with gentle shaking for 1 h at room temperature. Then, the beads were washed three times with IP Lysis/Wash Buffer and resuspended in 50 μl of 1% (w/v) SDS sample buffer and boiled at 97°C for 10 min. The proteins were separated by SDS-PAGE (10% SDS) and transferred to a PVDF membrane for western blot analysis.

Proximity ligation assay

Duolink in situ proximity ligation assay detection kit (Sigma-Aldrich) was used to detect physical proximity between PPFIA4 and CASK, between PPFIA4 and AKT1, between CASK and AKT1, between PPFIA4 and p-AKT1(Thr308), between PPFIA4 and p-AKT1(Ser473), between CASK and p-AKT1(Thr308), or between CASK and p-AKT1(Ser473), following the guidelines from the manufacturer as previously descripted (12). In brief, cells were cultured in 8-well chamber slides following fixation with 4% paraformaldehyde buffer, permeabilization with 0.5% Triton X-100 buffer, blocking with goat serum, and incubation overnight at 4°C with 2 primary Abs (mouse anti-human PPFIA4 and rabbit anti-human CASK/rabbit anti-HA, mouse anti-human CASK and rabbit anti-human PPFIA4/rabbit anti-Flag, mouse anti-human PPFIA4 and rabbit anti-human AKT1, mouse anti-human CASK and rabbit anti-human AKT1, mouse anti-human PPFIA4 and rabbit anti-human p-AKT1(Thr308), mouse anti-human PPFIA4 and rabbit anti-human p-AKT1(Ser473), mouse anti-human CASK and rabbit anti-human p-AKT1(Thr308), or mouse anti-human CASK and rabbit anti-human p-AKT1(Ser473)), derived from different host species. After rinsing away non-specific binding Abs, we performed the following steps according to the supplier’s protocol, including hybridizations, ligations, detection, and slide sealing steps, and then imaged by a confocal fluorescence microscope (LSM 900, Zeiss).

GST-pull down assay

GST tag was purchased from Genecreate. The coding sequences of *PPFIA4*, *CASK* and *AKT1* were inserted into pGEX-4T-1 plasmids, fused with GST and HA tags, respectively. These recombinant plasmids were introduced into *E. coli*. Protein expression was induced by the addition of isopropyl β-D-thiogalactoside (IPTG)

followed by incubation. Cells were subsequently lysed and sonicated, followed by centrifugation to collect supernatants. GST-PPFIA4 and GST-CASK fusion proteins were prepared and purified using GST magnetic beads (Beyotime) following the manufacturer's instructions. GST-PPFIA4 protein was mixed with HA-CASK or HA-AKT1 protein respectively. GST-CASK protein was mixed with HA-AKT1 protein. Following incubation and washing, the proteins were separated via SDS-PAGE, and analyzed by western blotting.

Surface plasmon resonance (SPR) assay

SPR analysis was conducted using a Biacore 8K instrument (Biacore AB, GE Healthcare) equipped with CM5 sensor chips (Cytiva, product number 29149603). All solutions were filtered through 0.22 μm Millipore membranes to ensure purity. Target proteins were immobilized on the sensor chip surface via amine coupling following standard protocols. Specifically, a 1:1 (v/v) mixture of 100 mM NHS and 400 mM EDC was injected to activate the chip matrix for 10 minutes at a flow rate of 10 μL/min. Subsequently, the target proteins (SAM1, SAM2-3 and SAM1-3) were diluted to 100 μg/mL in 10 mM acetate buffer (pH 5.5) and injected onto the activated chip surface for immobilization over 5 minutes at the same flow rate. Following immobilization, 1 M ethanolamine was injected for 10 minutes at 10 μL/min to block any remaining active carboxyl groups on the chip surface, thereby preventing non-specific binding. After protein immobilization, the test compounds were serially diluted in HBS buffer (10 mM HEPES, pH 7.4, 0.137 M NaCl, 3.4 mM EDTA, 0.05% P20) across a concentration range from 100 μM to 0.1 μM. These dilutions were injected over the immobilized proteins with an association phase of 180 seconds and a dissociation phase of 180 seconds at a flow rate of 30 μL/min. Upon completion of each binding cycle, the sensor chip surface was regenerated by injecting 100 mM glycine/HCl buffer (pH 2.0) for 30 seconds at a flow rate of 30 μL/min to dissociate bound compounds and restore the chip for subsequent measurements. All experiments were conducted at 25 °C to maintain consistent temperature control of the samples and buffers. Data were analyzed using Biacore Insight Evaluation Software (version 2.0.15.12933), fitting the sensor responses to a 1:1 binding model to calculate binding affinities (K_D values). This SPR protocol effectively integrates efficient target protein immobilization, specific ligand binding, and robust chip regeneration steps, ensuring the accuracy and reproducibility of SPR measurements. It is well-suited for analyzing interactions between small molecules and proteins, providing reliable insights into binding kinetics and affinities. All data were analyzed using Biacore Insight Evaluation Software (V 2.0.15.12933).

GEC permeability assay

In vitro GEC permeability assay was performed as below. Briefly, AGS cells were seeded on collagen-coated transwells (3-mm pore) and allowed to grow as a 3-day-old mature monolayer. After overnight starvation, 500 µl and 100 µl media (MMP1 (1 µg/ml) or medium control) were added for 24 h (37°C) to the bottom and top chambers, respectively. After washing, 500 µl RPMI 1640 medium and 100 µl FITC-dextran (molecular weight=4KD; 1 mg/ml) was added for 1 h (37°C) to the bottom and top chambers, respectively. Fluorescence was quantified using a SpectraMax M5 Microplate Reader (Molecular Devices) with excitation at 494 nm and emission at 520 nm.

Transepithelial electrical resistance (TEER) measurements

AGS cells were seeded in 12-well transwell plates (8.0-µm pore) considering an initial seeding of 150000 cells/well to achieve a 60-80% confluence after a 24 h incubation at 37 °C. After this incubation, AGS cells were stimulated with MMP1 (1 µg/ml) for 24 h. As previously described (13), TEER measurements were then performed using an EVOM2 Epithelial Voltohmeter with an STX2 electrode, and TEER values were further calculated.

Cell viability/proliferation assay

AGS cells expressing PPFIA4-Flag were treated with kira6 (100 nM) for 12, 24 and 48 h respectively, and the viability of these cells were measured by trypan blue, and the proliferation of these cells were measured using Cell Counting Kit-8 (CCK-8) (Solarbio, China) following the manufacturer's instructions.

Immunohistochemistry

Paraformaldehyde-fixed and paraffin-embedded samples were cut into 5 µm sections. For immunohistochemical staining, the sections were incubated with rabbit anti-human/mouse PPFIA4 followed by HRP-conjugated anti-rabbit IgG and later its substrate diaminobenzidine. All the sections were finally counterstained with haematoxylin and examined using a microscope (Nikon Eclipse 80i; Nikon).

Immunofluorescence

Gastric organoids were infected with *H. pylori* or CagA- *H. pylori* (MOI=100) for 24 h, or were infected with *H. pylori* (MOI=100) in the presence or absence of IL-33 (100 ng/ml) for 24 h. AGS cells were infected with *H.*

pylori (MOI=100) for 24 h. AGS cells were treated with MMP1 (1 μg/ml) for 24 h. AGS cells expressing PPFIA4-Flag were cultured. Paraformaldehyde-fixed tissue sections, gastric organoid sections or AGS cells were washed in PBS, blocked with 20% goat serum in PBS, and stained for PPFIA4/CASK/AKT1, PPFIA4/CASK/p-AKT1(Thr308), PPFIA4/CASK/p-AKT1(Ser473), PPFIA4/GIF, PPFIA4/ATB4B, PPFIA4/MUC5AC, PPFIA4/ChgA, PPFIA4/Dclk1, PPFIA4/Epcam, CXCL3/GIF, PPFIA4/CASK, Ly6G/MHC II, CD3, CD19, NK1.1, CD68, PPFIA4, E-cadherin or ZO-1. AGS cells were treated with MMP1 (1 μg/ml) at 4°C for 3 h. Paraformaldehyde-fixed AGS cells were washed in PBS, blocked with 20% goat serum in PBS, and stained for MMP1/E-cadherin or MMP1/ZO-1. Slides were examined with a confocal fluorescence microscope (LSM 900, Zeiss).

Real-time PCR

DNA of the biopsy specimens were extracted with QIAamp DNA Mini Kit and RNA of biopsy specimens and cultured cells were extracted with TRIzol reagent. The RNA samples were reversed transcribed into cDNA with PrimeScript^{TM} RT reagent Kit. Real-time PCR was performed on an IQ5 (Bio-Rad) with Real-time PCR Master Mix according to the manufacturer's specifications. The expression of 16s rDNA, *cagA*, *PPFIA4/Ppfia4*, *IL33/Il33*, *ST2/St2*, *CXCL3/Cxcl3*, *MMP1/Mmp1*, and genes of cytokine, chemokine, MMP, β-defensin and Reg3 was measured using the TaqMan and/or SYBR green method with the relevant primers (Supplemental Table 3). For mice, mouse *β2-microglobulin*/*β-actin* mRNA level served as a normalizer, and its level in the unstimulated/uninfected cells or stomach/cells of uninfected or WT mice served as a calibrator. For human, human *GAPDH* mRNA level served as a normalizer, and its level in the unstimulated/uninfected/NC-treated cells or stomach of uninfected donors served as a calibrator. The relative gene expression was expressed as fold change of relevant mRNA calculated by the ΔΔCt method.

Flow cytometry

Cell surface markers were stained with specific or isotype control Abs. For intracellular molecules measurements, the cells were stimulated for 5 h, using Leukocyte Activation Cocktail. Intracellular cytokine staining was performed after fixation and permeabilization, using Perm/Wash solution. Then, the cells were analyzed by multicolor flow cytometry on a FACSCanto^{TM} (BD Biosciences). Data were analyzed with Flowjo (TreeStar) or FACSDiva software (BD Biosciences).

ELISA

Isolated human and mouse gastric tissues were homogenized in 1 ml sterile Protein Extraction Reagent, and centrifuged. Tissue supernatants were collected for ELISA. Concentrations of IL-33, CXCL3, MMP1 and IFN-γ in the tissue/cell supernatants were determined using ELISA kits according to the manufacturer's instructions.

Western blot analysis

Western blots were performed on 10%-15% SDS-PAGE gel transferred PVDF membranes with equivalent amounts of cell or tissue lysate proteins for each sample. Five percent skim milk was used for blocking the PVDF membranes. Mouse PPFIA4, CASK, AKT1, p-AKT1(Thr308), p-AKT1(Ser473), p65, p-p65, MMP1, E-cadherin and ZO-1 was detected with rabbit anti-PPFIA4 Abs, rabbit anti-CASK Abs, rabbit anti-AKT1 Abs, rabbit anti-p-AKT1(Thr308) Abs, rabbit anti-p-AKT1(Ser473) Abs, rabbit anti-p65 Abs, rabbit anti-p-p65 Abs, rabbit anti-MMP1 Abs, mouse anti-E-cadherin Abs and rabbit anti-ZO-1 Abs; human PPFIA4, c-Jun, p-c-Jun, CASK, AKT1, p-AKT1(Thr308), p-AKT1(Ser473), p65, p-p65, IRE1α, p-IRE1α, MMP1, E-cadherin and ZO-1 were detected with mouse anti-PPFIA4 Abs/rabbit anti-Flag Abs, rabbit anti-c-Jun Abs, rabbit anti-p-c-Jun Abs, mouse anti-CASK/rabbit anti-HA Abs, rabbit anti-AKT1 Abs, rabbit anti-p-AKT1(Thr308) Abs, rabbit anti-p-AKT1(Ser473) Abs, rabbit anti-p65 Abs, rabbit anti-p-p65 Abs, rabbit anti-IRE1α Abs, rabbit anti-p-IRE1α Abs, rabbit anti-MMP1 Abs, mouse anti-E-cadherin Abs and rabbit anti-ZO-1 Abs, respectively. This was followed by incubation with HRP-conjugated secondary Abs. Bound proteins were visualized using Super ECL plus Western blotting Kit (Bioground, China).

Tandem mass tag combined with liquid chromatography-tandem mass spectrometry (TMT-LC-MS/MS) and quantitative phosphoproteome analysis

AGS cells stably expressing PPFIA4-Flag were cultured. Following centrifugation, the precipitates were washed with PBS and immediately frozen in liquid nitrogen. After thawing, the samples were sonicated three times on ice using a high intensity ultrasonic processor (Scientz) in lysis buffer, containing 8 M urea, 1% phosphatase inhibitor cocktail, and 1% protease inhibitor cocktail. The remaining debris was removed by centrifugation at 12000 g at 4°C for 10 min. Finally, the supernatant was collected, and the protein concentration was determined with BCA Protein Assay kit according to the manufacturer's instructions. For TMT phosphoproteome analysis, the same amount of protein from different samples were used for this experiment. The protein solution was incubated on ice for 2 h and centrifuged (4°C, 4500 g, 5 min). The

precipitate was washed three times with pre-cooled acetone. Tetraethylammonium bromide (TEAB, 200 mM) was added to resuspend the protein pellet and trypsin was added at 1:50 trypsin-to-protein mass ratio for the digestion overnight at 37°C. The digests were incubated at 37°C for 30 min with DL-Dithiothreitol (DTT, 5 mM), and then incubated at room temperature for 15 min in the dark with iodoacetamide (IAM, 11mM). Using a Strata X C18 SPE column, the tryptic peptides were desalted, vacuum-dried, reconstituted in TEAB (0.5 M) and further labeled by TMT in accordance with the manufacturer's instructions. The labeled tryptic peptides were fractionated by high pH reverse-phase HPLC using BETASIL C18 column (5 μm particles, 4.6 mm, 250×10 mm). The peptides were separated into 54 fractions with a gradient of 8% to 32% acetonitrile over 80 min. Then, the peptides were dried by vacuum centrifugation. Peptide mixtures were first incubated with IMAC microspheres suspension with vibration in loading buffer (50% acetonitrile/0.5% acetic acid). To remove the non-specifically adsorbed peptides, the IMAC microspheres were washed with 50% acetonitrile/0.5% acetic acid and 30% acetonitrile/0.1% trifluoroacetic acid, sequentially. To elute the enriched phosphopeptides, the elution buffer containing 10% NH_4OH was added and the enriched phosphopeptides were eluted with vibration. The supernatant containing phosphopeptides was collected and lyophilized for LC-MS/MS analysis. The resulting MS/MS data were processed using PD 2.4 search engine. Tandem mass spectra were searched against homo sapiens database concatenated with reverse decoy database. The identify differentially expressed phosphoproteins (DEPPs) whose CV-values were <0.2 with their corresponding fold change scores were selected for further analyses. Using the UniPort-GOA database (http://www.ebi.ac.uk/GOA/), the InterProScan soft to annotated protein's GO functional based on protein sequence alignment method. Using the Kyoto Encyclopedia of Genes and Genomes (KEGG) database was used to annotate protein pathway. Then mapping the annotation result on the KEGG pathway database using KEGG online service tools KEGG mapper. For each term, a two-tailed Fisher's exact test was employed to test the enrichment of the differentially expressed protein against all identified proteins. The pathway with a two-tailed Fisher's exact test (P<0.05) was considered significant. All differentially expressed modified protein sequence were searched against the STRING database version 11.0 for protein-protein interactions. Chord diagram and network were visualized by circlize R package and Cytoscape software.

In silico prediction

We first retrieved the structures of PPFIA4 (UniProt ID: O75335), CASK (UniProt ID: O14936) and AKT1 (UniProt ID: P31749) from the AlphaFold database. Initially, PPFIA4 and CASK were docked using HADDOCK.

The resulting protein-protein complexes were visualized, and their binding free energies were calculated using Amber software. The conformation exhibiting the strongest binding affinity was selected for subsequent molecular docking with AKT1 using HADDOCK. Additionally, alanine scanning was performed to identify key residues critical for protein-protein interactions. We then employed a molecular docking-based virtual screening approach to identify inhibitors of the PPFIA4 protein. Initially, we screened 12426 active compounds from a compound library against PPFIA4. The PPFIA4 protein sequence (UniProt ID: O75335) was retrieved from the UniProt database and its structure predicted using AlphaFold2. Protein-ligand binding pockets were identified with Protein Plus (https://proteins.plus/). The virtual screening workflow involved two stages of molecular docking. In the first stage, AutoDock Vina version 1.1.2 was utilized to dock the small molecules with the PPFIA4 protein, selecting compounds with docking scores below -12 kcal/mol for further evaluation. In the second stage, the selected compounds were re-docked using leDock software. Compounds achieving docking scores of ≤-5 kcal/mol were subjected to MMGBSA calculations using AmberTools. The top 20 compounds, based on their MMGBSA rankings, were prioritized for experimental validation.

Molecular dynamics simulation

The structure of kira6 was downloaded from the PubChem database and subjected to energy minimization using the MMFF94 force field in Chem3D, after which it was saved in mol2 format. The proteins PPFIA4, CASK, AKT1, and p65 were individually retrieved from the AlphaFold database with corresponding UniProt ID (O75335, O14936, P31749 and P19838) respectively. These receptor proteins were automatically processed, and docking configuration files were generated using the LePro module. Protein–ligand binding pockets were predicted using Protein Plus (https://proteins.plus/). The binding pocket interfaces of the receptor proteins were identified using PyMOL software, and the pocket locations in the LeDock docking configuration files were subsequently modified. After importing the Kira6 molecular structure, docking between the receptor proteins and Kira6 was performed using the LeDock module, and docking scores were calculated based on the scoring functions. We performed molecular dynamics simulations of ligand-receptor docking complexes using GROMACS (Version 2022.5). Protein topology files were generated with the AMBER99SB-ILDN force field, while ligand topology files were prepared using the ACPYPE script based on the Amber force field. The simulations were conducted in a cubic box solvated with TIP3P water molecules under periodic boundary conditions, and the system was neutralized with NaCl counterions. Prior to molecular dynamics, energy minimization was executed using the steepest descent algorithm to reduce the system's energy, employing

both Coulomb and van der Waals interaction cutoffs of 1.4 nm. Equilibration was achieved through 100 ps simulations in the NVT and NPT ensembles. Bond constraints were enforced using the LINCS algorithm, and long-range electrostatic interactions were treated via the Particle Mesh Ewald (PME) method. Each system underwent a 100 ns production run at 310 K and 1.0 bar under periodic boundary conditions. Subsequent analyses, including root-mean-square deviation (RMSD), root-mean-square fluctuation (RMSF), radius of gyration (Rg), and solvent-accessible surface area (SASA), were performed using GROMACS's gmx tools. The binding affinities of the protein-ligand complexes were calculated using GROMACS's g_mmpbsa module.

Bulk RNA sequencing

Total RNA from sg*PPFIA4* or sgNC-modified AGS cells was extracted using RNeasy Micro Kits, following the manufacturer's instructions. Then, the samples were sent to Guangzhou Epibiotek Co., Ltd. (Guangzhou, China) for RNA sequencing. The concentration and quality of RNA samples were determined by the NanoDrop 2000 spectrophotometer (NanoDrop technologies, USA) and then used to construct strand-specific (first cDNA strand) RNA libraries with Epi^{TM} mini longRNA-seq kit. RNA libraries for sequencing were performed on Illumina NovaSeq 6000 platform. Subsequently, 150 bp paired-end reads were mapped to the reference human genome build GRCh38/hg38. The reads mapped the genome were calculated using HTSeq. Differential expression analysis presented here has been performed with edgeR package. Significantly differentially expressed genes (DEGs) ($|log2FC| > 1$ and $FDR < 0.05$) were defined with the DESeq2 (v1.18.1). Gene ontology (GO) and KEGG pathway enrichment analyses were conducted by clusterProfiler R package. Hierarchically clustered heatmaps and volcano plots were visualized by heatmap R package and ggplot2 R package.

Microarray experiments

Gene expression profiles of *H. pylori*-infected and uninfected AGS cells were analyzed with the human Exon 1.0 ST GeneChip (Affymetrix), strictly following the manufacturer's protocol. Microarray experiments were performed at the Genminix Informatics (China) with the microarray service certified by Affymetrix.

Thiol(SH)-Linked Alkylation for the Metabolic sequencing of RNA (SLAM-seq)

After s^4U labeling and pulse/chase labeling, total RNA from *H. pylori*-infected and uninfected AGS cells was extracted using Trizol reagent, following the manufacturer's instructions. Then, the samples were sent to

Guangzhou Epibiotek Co., Ltd. (Guangzhou, China) for SLAM-seq. Briefly, after Poly(A) RNA capture, IAA treatment and library preparation, Bioptic Qsep100 Analyzer is used for library quality inspection to check whether the library size distribution is in line with the theoretical size, and NovaSeq high-throughput sequencing platform and PE150 sequencing mode are used for sequencing. SLAM-DUNK was used to map reads, filter alignments, call variants on final alignments and use these to calculate conversion rates, counts and various statistics for 3'UTRs. Extract and summarize the count results of tcount files into a count table. Differential gene expression analysis was performed using the DESeq2 R-package. GO and KEGG analyses were performed using clusterProfiler R package (v3.6.0).

Digital RNA with pertUrbation of Genes sequencing (DRUG-seq)

Total RNA from gastric G-MDSCs and MHCII$^+$ neutrophils sorted from WT mice 15 weeks p.i. was extracted using Trizol reagent, following the manufacturer's instructions. Then, the samples were sent to Guangzhou Epibiotek Co., Ltd. (Guangzhou, China) for DRUG-seq. DRUG-seq was performed on an Illumina NovaSeq 6000 platform (Illumina, San Diego, CA, USA). Raw reads were filtered to remove the adaptor by cutadapt (v2.5; https://cutadapt.readthedocs.io/en/stable/). The sequenced reads were single-end mapped to reference genome sequences (GRCm38) using NextGenMap (v0.5.5; http://cibiv.github.io/NextGenMap/) and SAMtools (v1.10; http://samtools.sourceforge.net). The featureCounts (v1.6.3; https://subread.sourceforge.net/featureCounts.html) was used to perform quantification on the mapped sequences. The differential expression analysis was performed with the DESeq2 (v1.18.1) to define DEGs (|log2FC| >1 and P<0.05). The DEGs were used for GO and KEGG analyses.

Switching Mechanism At the 5' end of RNA Template sequencing (SMART-seq)

Total RNA from gastric G-MDSCs and MHCII$^+$ neutrophils sorted from WT mice 15 weeks p.i. was extracted using Trizol reagent. Then, the samples were sent to Guangzhou Epibiotek Co., Ltd. (Guangzhou, China) for SMART-seq. The RNA-seq transcriptome library was prepared following SMART-Seq_V4 Ultra Low Input RNA Kit according to the manufacturer's instructions (Illumina, San Diego, CA). After reverse transcription and quantified by Qubit 4.0, paired-end RNA-seq sequencing library was sequenced with the NovaSeq X plus sequencer (2×150bp read length). The raw paired end reads were trimmed and quality controlled by fastp with default parameters. Then clean reads were separately aligned to reference genome with orientation mode using HISAT2 software. The mapped reads of each sample were assembled by StringTie in a reference-based

approach. The differential expression analysis was performed with the DESeq2 (v1.18.1) to define DEGs (|log2FC| >1 and *P*<0.05). The DEGs were used for GO and KEGG analyses.

Single-cell RNA sequencing (scRNA-seq)

Mouse $CD45^+$ cells were sorted by using anti-CD45 magnetic beads (StemCell Technologies) from gastric tissue single-cell suspensions of *H. pylori*-infected *Ppfia4*$^{\Delta GEC}$ mice and *Ppfia4*$^{flox/flox}$ littermates 15 weeks p.i.. Then, the samples were sent to Majorbio Co., Ltd. (Shanghai, China) for scRNA-seq. We targeted for approximately 13000 cells recovered from each channel on 10X Genomics Chips. ScRNA-seq libraries were performed according to the standard manufacturer's protocol (Chromium Single Cell 3' v3.1), and sequencing was accomplished with an Illumina NovaSeq 6000 (Illumina, Inc, San Diego, CA) with 150-bp paired-end mode. Raw sequencing data were processed using CellRanger (version 7.1.0) with default parameters.

Analysis of scRNA-seq (Cell-type clustering and identification, Pseudotime trajectory analysis, cell communication analysis, gene module enrichment analysis and gene set enrichment analysis)

The FindVariableGenes function from Seurat R package was used to screen highly variable genes (HVGs). Then the principal component analysis (PCA) was performed based on HVGs, followed by visualization using uniform manifold approximation and projection (UMAP) or t-distributed stochastic neighbor embedding (tSNE). To detect cluster-specific expressed genes, the FindAllMarkers from Seurat R package was used to compare the differential genes of a given cluster with all other clusters. The identified marker genes were visualized through VlnPlot and FeaturePlot functions. The SingleR package was utilized to assign cell types based on a public reference dataset. This involved correlating the expression profiles of unidentified cells with those in the reference dataset, assigning the cell type from the refence dataset that exhibited the highest correlation to the unidentified cell type. Pseudotime analysis was performed with Monocle 1 to determine the dynamic differentiation trajectories of cell subpopulations and gene expression changes with gastritis. Utilizing the Monocle R package, genes exhibiting significant expression variation across cells were firstly identified, and their expression profiles were used for dimensionality reduction; subsequently, a minimum spanning tree (MST) was constructed, representing the differentiation trajectories by Destiny, and trajectory analysis algorithm DDRTree. Cellchat package was used to calculate differences in cell communication between different groups, and the AddModuleScore function was used for gene module enrichment analysis, which was derived from the Molecular Signatures Database. To compare the differences in biological states and pathways between cell

subtypes, gene set enrichment analysis (GSEA) was performed to identify whether the differential gene expression between two different cell subtypes was significantly enriched in these gene sets.

Analysis of public scRNA-seq datasets (Cell-type clustering and identification, SCENIC analysis, Protein-protein interaction network analysis)

For the analysis of the datasets of clinic samples of *H. pylori*-associated patients with gastritis, raw data of 18 patients pathologically diagnosed with gastric lesions including 6 gastritis (GS) (3 *H. pylori*-positive (*HP*+) and 3 *H. pylori*-negative (*HP*-)), 6 of intestinal metaplasia (IM) (3 *HP*+ and 3 *HP*-), and 6 of gastric cancer (GC) (3 *HP*+ and 3 *HP*-) from the Gene Expression Omnibus (GEO) (GSE249874) were downloaded. The FindVariableGenes function from Seurat R package was used to screen highly variable genes (HVGs). Then the principal component analysis (PCA) was performed based on HVGs, followed by visualization using UMAP. To detect cluster-specific expressed genes, the FindAllMarkers from Seurat R package was used to compare the differential genes of a given cluster with all other clusters. The identified marker genes were visualized through VlnPlot and FeaturePlot functions. The SingleR package was utilized to assign cell types based on a public reference dataset. This involved correlating the expression profiles of unidentified cells with those in the reference dataset, assigning the cell type from the refence dataset that exhibited the highest correlation to the unidentified cell type. For the identification of GEC subtypes, the specific embryonic development patterns of GECs were also considered. To predict the key regulons in *H. pylori*-associated gastritis, gene regulatory networks were constructed by SCENIC analysis. The analysis of transcriptional factor binding motifs was performed using RcisTarget R package. Grnboost was used to infer the co-expression network. The co-expressed modules between transcriptional factors and potential target genes, as well as the regulon activity score (RAS) of single cell. The regulon specificity score (RSS) inferred the specific correspondence of regulon to each cell. Connection specificity index (CSI) represented the association between different regulons. To predict the interaction pattern of regulons in *H. pylori*-associated gastritis, the protein-protein interaction (PPI) of regulons was constructed from the STRING12.0 database, and then was merged and visualized by Cytoscape software (version 3.7.2). We removed the protein nodes with no interactions with other proteins. Moreover, to identify which genes may be co-regulated to other genes, we conducted co-expression analysis of all genes to calculate Pearson correlation of pair-to-pair genes with the screening criteria (P<0.05). In the PPI network, the genes served as the nodes and the edges represented the associated interactions. The connectivity degree of each node, which indicates the number of interactions of the corresponding gene.

References

1. Zhuang Y, et al. A pro-inflammatory role for Th22 cells in Helicobacter pylori-associated gastritis. *Gut*. 2015;64(9):1368-1378.
2. Asahi M, et al. Helicobacter pylori CagA protein can be tyrosine phosphorylated in gastric epithelial cells. *J Exp Med*. 2000;191(4):593-602.
3. Suzuki M, et al. Helicobacter pylori CagA phosphorylation-independent function in epithelial proliferation and inflammation. *Cell Host Microbe*. 2009;5(1):23-34.
4 Gong X, et al. The crosstalk between efflux pump and resistance gene mutation in Helicobacter pylori. *Gut Microbes*. 2024;16(1):2379439.
5. Roussel Y, et al. Evaluation of DNA extraction methods from mouse stomachs for the quantification of H. pylori by real-time PCR. *J Microbiol Methods*. 2005;62(1):71-81.
6. Mikula M, et al. Quantitative detection for low levels of Helicobacter pylori infection in experimentally infected mice by real-time PCR. *J Microbiol Methods*. 52003;55(2):351-359.
7. Shi Y, et al. Helicobacter pylori-induced Th17 responses modulate Th1 cell responses, benefit bacterial growth, and contribute to pathology in mice. *J Immunol*. 2010;184(9):5121-5129.
8. Lv YP, et al. Helicobacter pylori-induced matrix metallopeptidase-10 promotes gastric bacterial colonization and gastritis. *Sci Adv*. 2019;5(4):eaau6547.
9. Ferrero RL, et al. NF-kappaB activation during acute Helicobacter pylori infection in mice. *Infect Immun*. 2008;76(2):551-561.
10. Miao ZF, et al. A Dedicated Evolutionarily Conserved Molecular Network Licenses Differentiated Cells to Return to the Cell Cycle. *Dev Cell*. 52020;55(2):178-194.e7.
11. Sebrell TA, et al. A Novel Gastric Spheroid Co-culture Model Reveals Chemokine-Dependent Recruitment of Human Dendritic Cells to the Gastric Epithelium. *Cell Mol Gastroenterol Hepatol*. 2019;8(1):157-171.e3.
12. Lu H, et al. APE1 Upregulates MMP-14 via Redox-Sensitive ARF6-Mediated Recycling to Promote Cell Invasion of Esophageal Adenocarcinoma. *Cancer Res*. 2019;79(17):4426-4438.
13. Kim YM, et al. Inhibition of AGS human gastric cancer cell invasion and proliferation by Capsosiphon fulvescens glycoprotein. *Mol Med Rep*. 2013;8(1):11-16.

Supplemental Figure 1

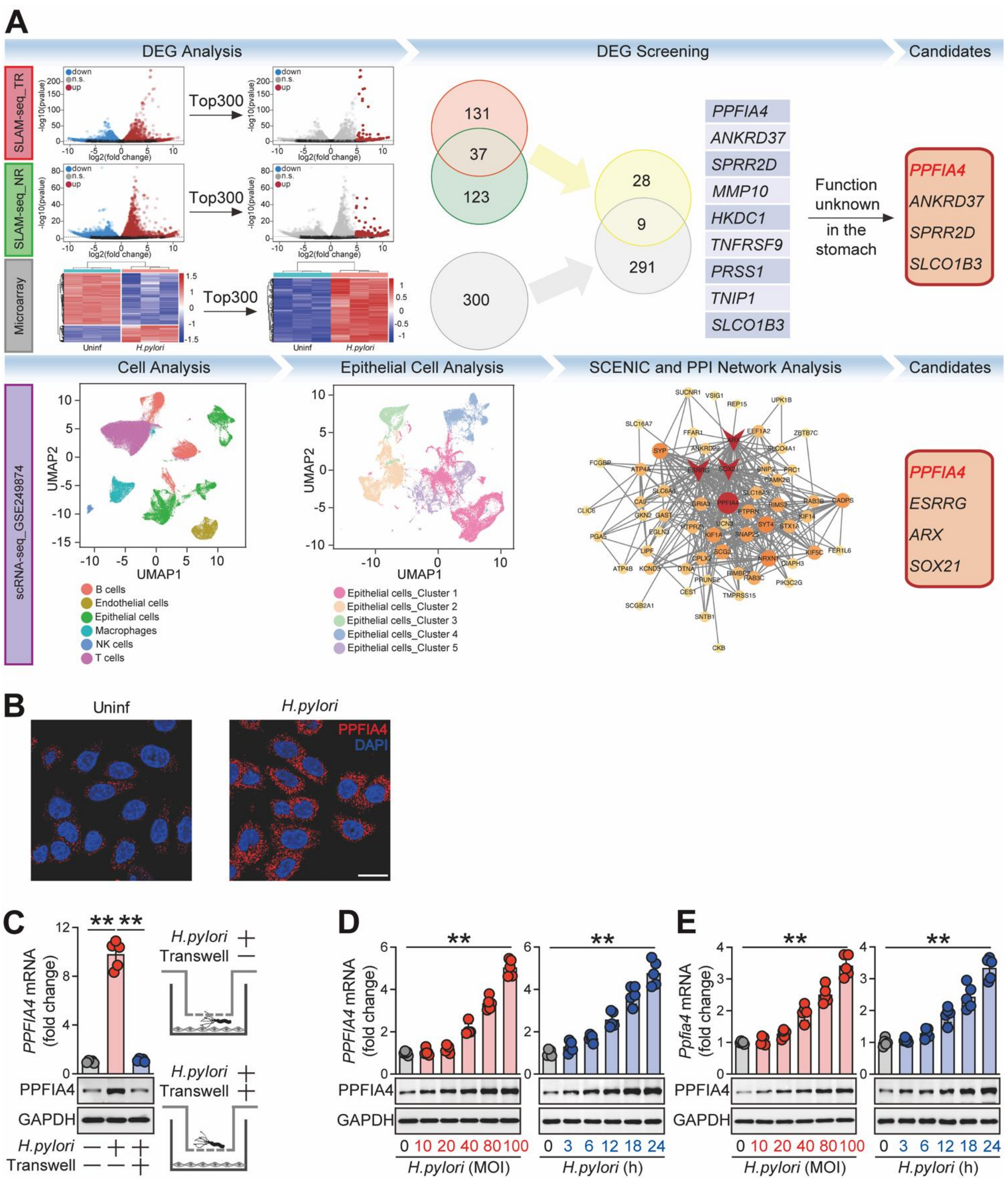

PPFIA4 is increased in gastric epithelial cells (GECs) from gastric mucosa of *H. pylori*-infected patients and mice. (A) Firstly, analyses of the SLAM-seq data of 2 cohorts (total RNA (TR), new RNA (NR)) of uninfected and *H. pylori*-infected AGS cells. VolcanoPlots of significant DEGs were shown in DEG analysis. Overlapping top 300 significantly upregulated DEGs from 2 cohorts identified 37 significantly upregulated DEGs. Overlapping these 37 significantly upregulated DEGs with top 300 significantly upregulated DEGs from microarray data of uninfected and *H. pylori*-infected AGS cells further identified 9 significantly upregulated

DEGs, and 4 significantly upregulated DEGs with unknown function in the stomach were finally identified. Secondly, analyses of scRNA-seq data from GEO (GSE249874) to identify 5 clusters of GECs. Further SCENIC analysis of these GEC clusters and further PPI analysis identified 3 key regulons that are mostly associated with *H. pylori*-associated gastritis, and *PPFIA4* that has the closest relationship with these 3 regulons. By overlapping the DEGs enriched from data of SLAM-seq and microarray as well as key genes enriched from scRNA-seq data, *PPFIA4* was identified as the only candidate. (B) Immunofluorescence showed PPFIA4 protein in uninfected and *H. pylori*-infected AGS cells (MOI=100, 24 h). Scale bars: 10 microns. (C) The induction of PPFIA4 in AGS cells infected with *H. pylori* (MOI=100, 24 h) was assessed by a transwell assay and analyzed by real-time PCR and western blot (n=5) as descried in the Methods. (D and E) *PPFIA4/Ppfia4* expression and PPFIA4 protein in human (D) and mouse (E) primary gastric mucosa from uninfected donors/mice infected with *H. pylori* at different time point (MOI=100) or with different MOI (24 h) *ex vivo* were analyzed by real-time PCR and western blot (n=5). Data are presented as mean ± SEM. Statistics: unpaired 2-tailed t test (C) and 1-way ANOVA (D-E). *P<0.05, **P<0.01 for groups connected by horizontal lines.

Supplemental Figure 2

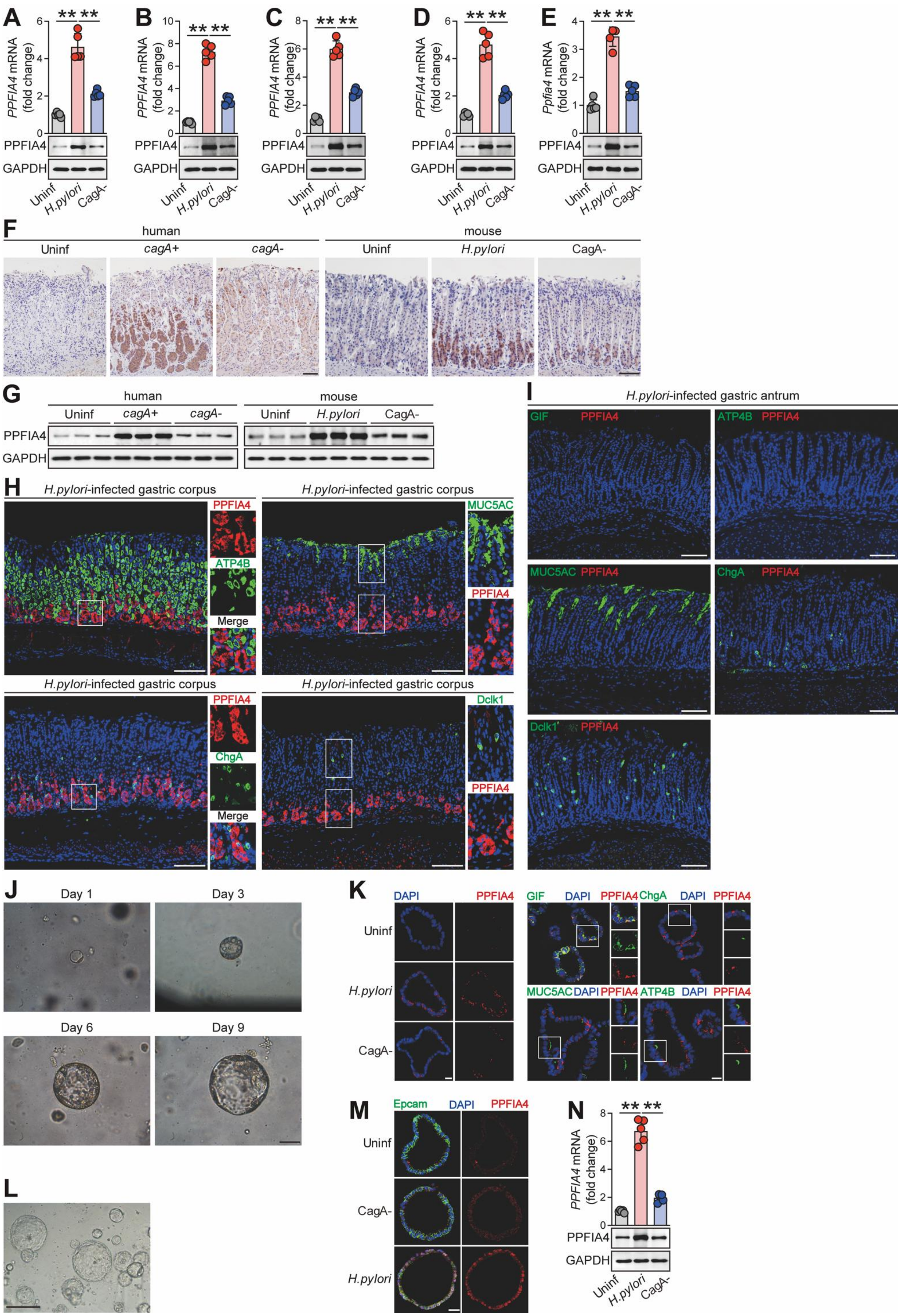

H. pylori induces gastric epithelial cells (GECs) to express PPFIA4 via CagA-AP1 pathway. (A-C) *PPFIA4* expression and PPFIA4 protein in *H. pylori*-infected, CagA- *H. pylori*-infected, and uninfected GES-1 cells (A), HGC-27 cells (B) and SGC-7901 cells (C) (MOI=100, 24 h) were analyzed by real-time PCR and western blot (n=5). (D and E) *PPFIA4/Ppfia4* expression and PPFIA4 protein in *H. pylori*-infected, CagA- *H. pylori*-infected, and uninfected human (D) and mouse (E) primary gastric mucosa from uninfected donors/mice (MOI=100, 24 h) were analyzed by real-time PCR and western blot (n=5). (F and G) PPFIA4 protein in gastric mucosa of *cagA*-positive *H. pylori*-infected, *cagA*-negative *H. pylori*-infected, and uninfected donors or in gastric mucosa of *H. pylori*-infected, CagA- *H. pylori*-infected, and uninfected mice 15 weeks p.i. was analyzed by immunohistochemical staining (F) and western blot (G). Scale bars: 100 microns. (H) Immunofluorescence showed PPFIA4 expression/ATP4B$^+$ cells, PPFIA4 expression/MUC5AC$^+$ cells, PPFIA4 expression/ChgA$^+$ cells and PPFIA4 expression/Dclk1$^+$ cells in gastric corpus of *H. pylori*-infected mice 15 weeks p.i.. Scale bars: 100 microns. (I) Immunofluorescence showed PPFIA4 expression/GIF$^+$ cells, PPFIA4 expression/ATP4B$^+$ cells PPFIA4 expression/MUC5AC$^+$ cells, PPFIA4 expression/ChgA$^+$ cells and PPFIA4 expression/Dclk1$^+$ cells in gastric antrum of *H. pylori*-infected mice 15 weeks p.i.. Scale bars: 100 microns. (J) Representative example of a growing mouse gastric organoid. Scale bars: 50 microns. (K) Immunofluorescence showed PPFIA4 protein in *H. pylori*-infected, CagA- *H. pylori*-infected, and uninfected mouse gastric organoids (MOI=100, 24 h), and PPFIA4-expressing GIF$^+$ cells, PPFIA4 expression/ATP4B$^+$ cells, PPFIA4 expression/MUC5AC$^+$ cells and PPFIA4 expression/ChgA$^+$ cells in *H. pylori*-infected mouse gastric organoids (MOI=100, 24 h). Scale bars: 10 microns. (L) Representative morphology of human gastric organoids. Scale bars: 200 microns. (M) Immunofluorescence showed PPFIA4 protein in *H. pylori*-infected, CagA- *H. pylori*-infected, and uninfected human gastric organoids (MOI=100, 24 h). (N) *PPFIA4* expression and PPFIA4 protein in *H. pylori*-infected, CagA- *H. pylori*-infected, and uninfected human gastric organoids (MOI=100, 24 h) were analyzed by real-time PCR and western blot (n=5). Data are presented as mean ± SEM. Statistics: unpaired 2-tailed t test (A-E and N). **P*<0.05, ***P*<0.01 for groups connected by horizontal lines.

Supplemental Figure 3

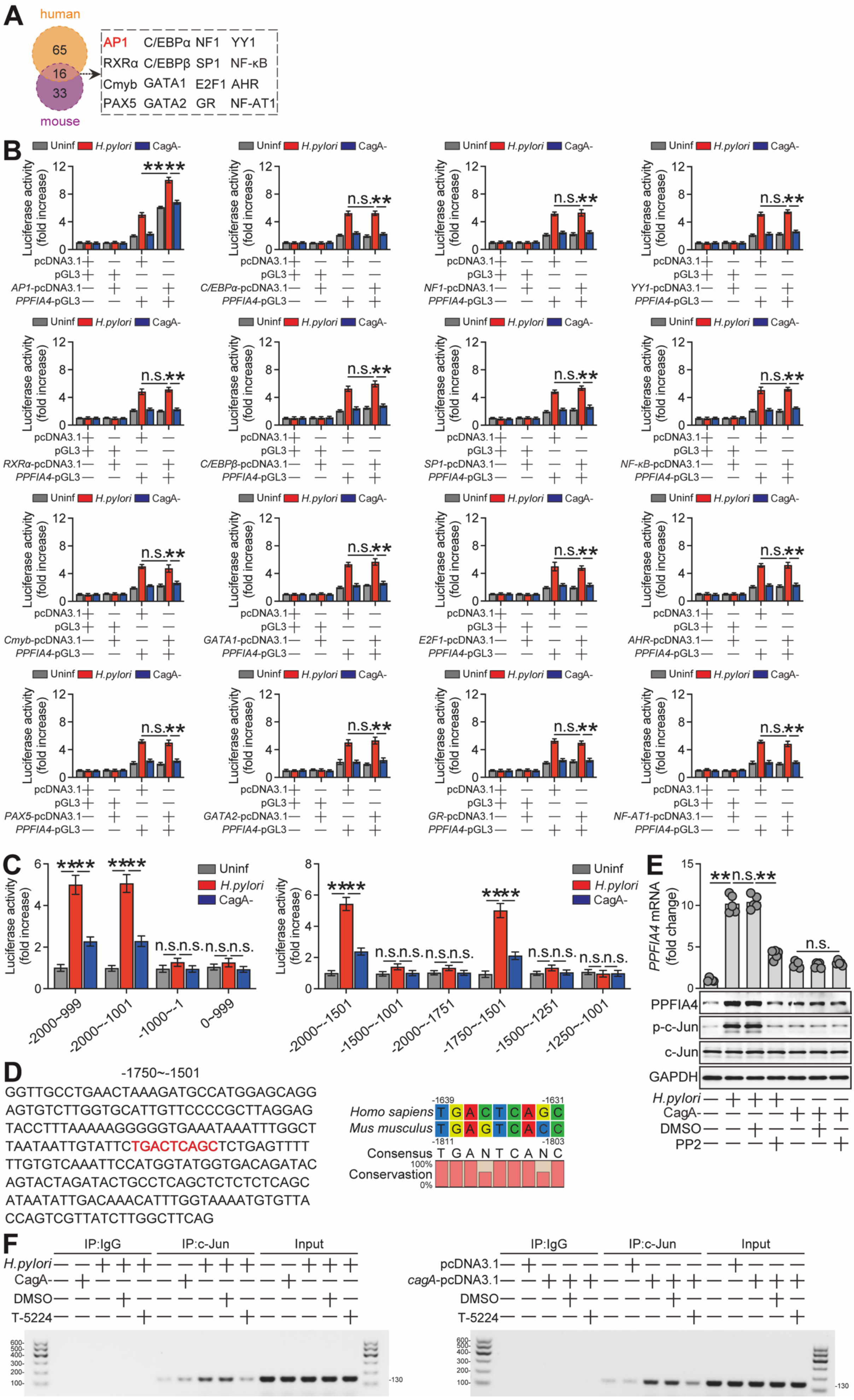

H. pylori induces gastric epithelial cells (GECs) to express PPFIA4 via CagA-AP1 pathway. (A) The transcription factors were predicted by the PROMO Web site using a 3000-base pair conserved segment of *PPFIA4/Ppfia4* promoter. (B) AGS cells were co-transfected with luciferase reporter constructs containing the *PPFIA4*-luc promoter or pGL3 and/or constructs expressing transcription factors or pcDNA3.1 for 24 h. Luciferase activity was measured to assess *PPFIA4* promoter activity after *H. pylori* or CagA- *H. pylori* infection (MOI=100) for 24 h (n=3). (C) AGS were transfected with luciferase reporter constructs containing the *PPFIA4*-luc promoter for 24 h. Luciferase activity was measured to assess *PPFIA4* promoter activity after *H. pylori* or CagA- *H. pylori* infection (MOI=100) for 24 h (n=3). (D) Sequences of the AP1 binding sites (red) in *PPFIA4* promoter (-1750/-1501). A conserved sequence of AP1 binding site of *PPFIA4* promoter with the highest scores predicted by the PROMO Web site in human and mice. (E) AGS cells were pre-treated with or without PP2 then infected with *H. pylori* or CagA- *H. pylori* (MOI=100) for 24 h. *PPFIA4* expression and PPFIA4, c-Jun and p-c-Jun proteins were analyzed by real-time PCR and western blot (n=5). (F) Representative data of ChIP assay in AGS cells infected with *H. pylori* (pre-treated with or without T-5224) or CagA- *H. pylori*, or in AGS cells transfected with plasmids *cagA*-pcDNA3.1 (pre-treated with or without T-5224) or pcDNA3.1, followed by regular PCR with primers designed for AP1 binding site of *PPFIA4* promoter region. Data are presented as mean ± SEM. Statistics: unpaired 2-tailed t test (B-C and E). **P*<0.05, ***P*<0.01, n.s. *P*>0.05 for groups connected by horizontal lines.

Supplemental Figure 4

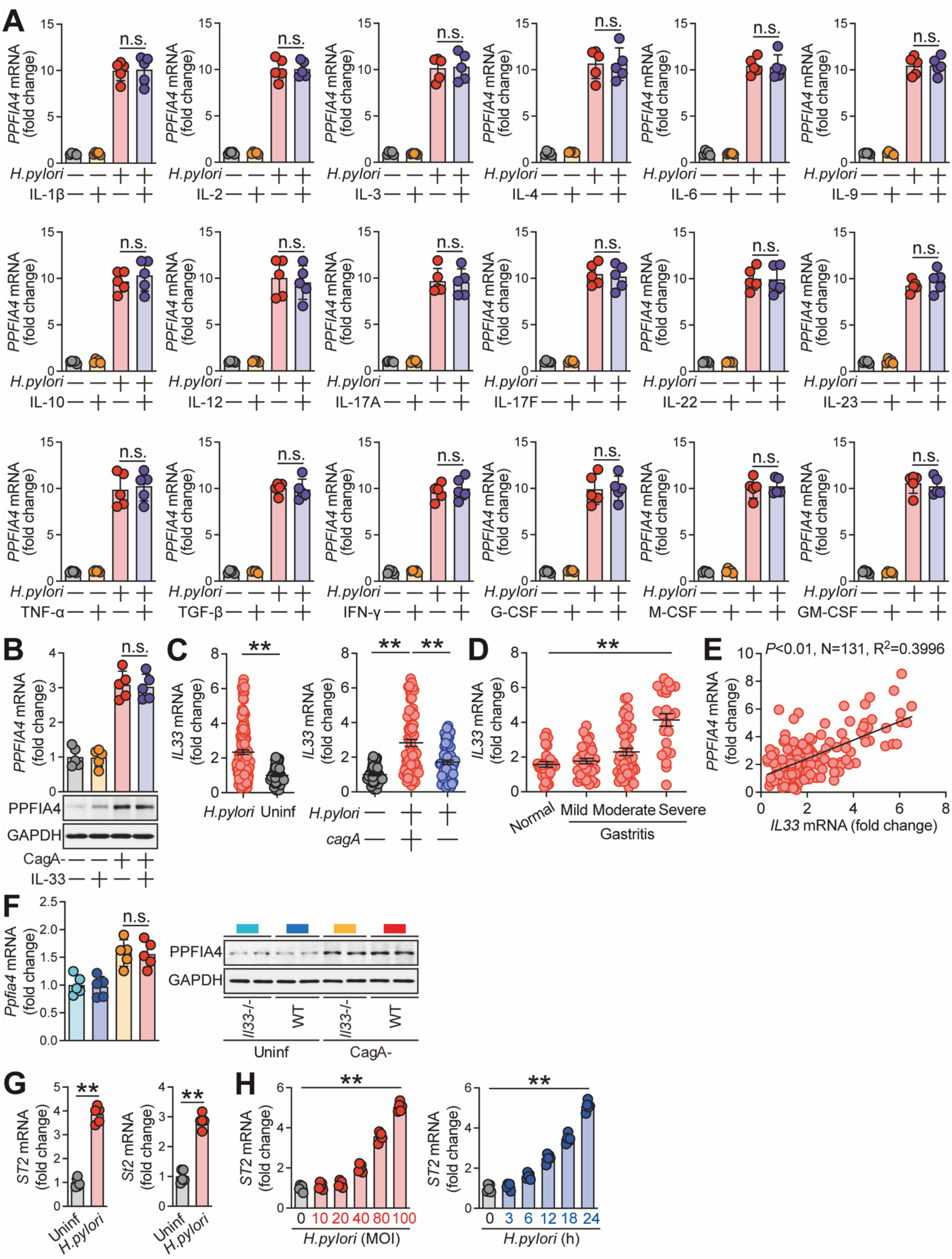

H. pylori and IL-33 induce PPFIA4 synergistically. (A) *PPFIA4* expression in AGS cells infected with *H. pylori* (MOI=100) in the presence or absence of IL-β, IL-2, IL-3, IL-4, IL-6, IL-9, IL-10, IL-12, IL-17A, IL-17F, IL-22,

IL-23, TNF-α, TGF-β, IFN-γ, G-CSF, M-CSF or GM-CSF (100 ng/ml) (24 h) was analyzed by real-time PCR (n=5). (B) *PPFIA4* expression and PPFIA4 protein in AGS cells infected with CagA- *H. pylori* (MOI=100) in the presence or absence of IL-33 (100 ng/ml) (24 h) were analyzed by real-time PCR and western blot (n=5). (C) *IL33* expression in gastric mucosa of *H. pylori*-infected (n=131) and uninfected donors (n=50), or in gastric mucosa of *cagA*-positive *H. pylori*-infected (n=74), *cagA*-negative *H. pylori*-infected (n=57) and uninfected donors (n=50) was compared. (D) *IL33* expression in gastric mucosa of *H. pylori*-infected patients with mild (n=34), moderate (n=45), severe inflammation (n=24), and with normal gastric histopathology (n=28) was compared. (E) The correlation between *PPFIA4* expression and *IL33* expression in gastric mucosa of *H. pylori*-infected patients was analyzed. (F) *Ppfia4* expression and PPFIA4 protein in gastric mucosa of CagA- *H. pylori*-infected WT and $Il33^{-/-}$ mice 15 weeks p.i. were compared (n=5). (G) *ST2*/*St2* expression in *H. pylori*-infected and uninfected human/mouse primary gastric epithelial cells (GECs) (MOI=100, 24 h) was analyzed by real-time PCR (n=5). (H) *ST2* expression in *H. pylori*-infected AGS cells at different time point (MOI=100) or with different MOI (24 h) was analyzed by real-time PCR (n=5). Data are presented as mean ± SEM. Statistics: unpaired 2-tailed t test (A-C and F-G), 1-way ANOVA test (D and H) and 2-tailed Pearson correlation test (E). $^*P<0.05$, $^{**}P<0.01$, n.s. $P>0.05$ for groups connected by horizontal lines.

Supplemental Figure 5

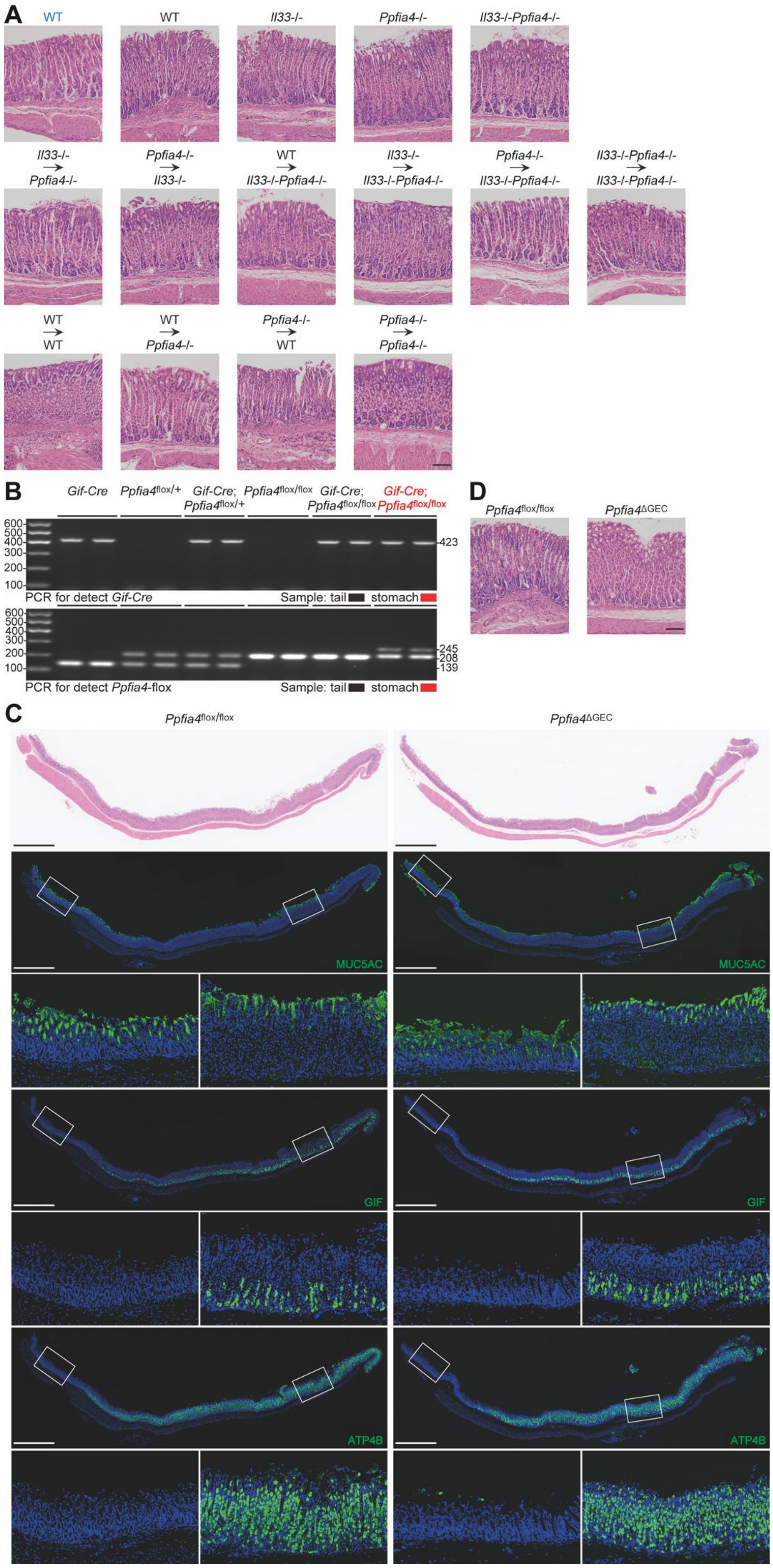

PPFIA4 increases inflammation in gastric mucosa during *H. pylori* infection. (A) Representative H&E staining images showing inflammation in gastric mucosa of uninfected WT mice (blue), *H. pylori*-infected WT, *Il33*$^{-/-}$, *Ppfia4*$^{-/-}$ and *Il33*$^{-/-}$*Ppfia4*$^{-/-}$ mice, or in gastric mucosa of *H. pylori*-infected BM chimera mice 15 weeks p.i.. Scale bars: 100 microns. (B) PCR genotype analysis of pups generated by crossing *Ppfia4*$^{flox/flox}$ mice with *Gif-Cre* mice to obtain *Ppfia4*$^{\Delta GEC}$ mice (*Gif-Cre*;*Ppfia4*$^{flox/flox}$) using specific primers (Supplemental Table 7). (C) Representative H&E staining images showing stomach tissue histology of *Ppfia4*$^{\Delta GEC}$ mice and *Ppfia4*$^{flox/flox}$ littermates. Scale bars: 1000 microns. Immunofluorescence showed MUC5AC^{+} cells, GIF^{+} cells and ATP4B^{+} cells in gastric mucosa of *Ppfia4*$^{\Delta GEC}$ mice and *Ppfia4*$^{flox/flox}$ littermates. Scale bars: 1000 microns. (D) Representative H&E staining images showing inflammation in gastric mucosa of *H. pylori*-infected *Ppfia4*$^{\Delta GEC}$ mice and *Ppfia4*$^{flox/flox}$ littermates 15 weeks p.i.. Scale bars: 100 microns.

Supplemental Figure 6

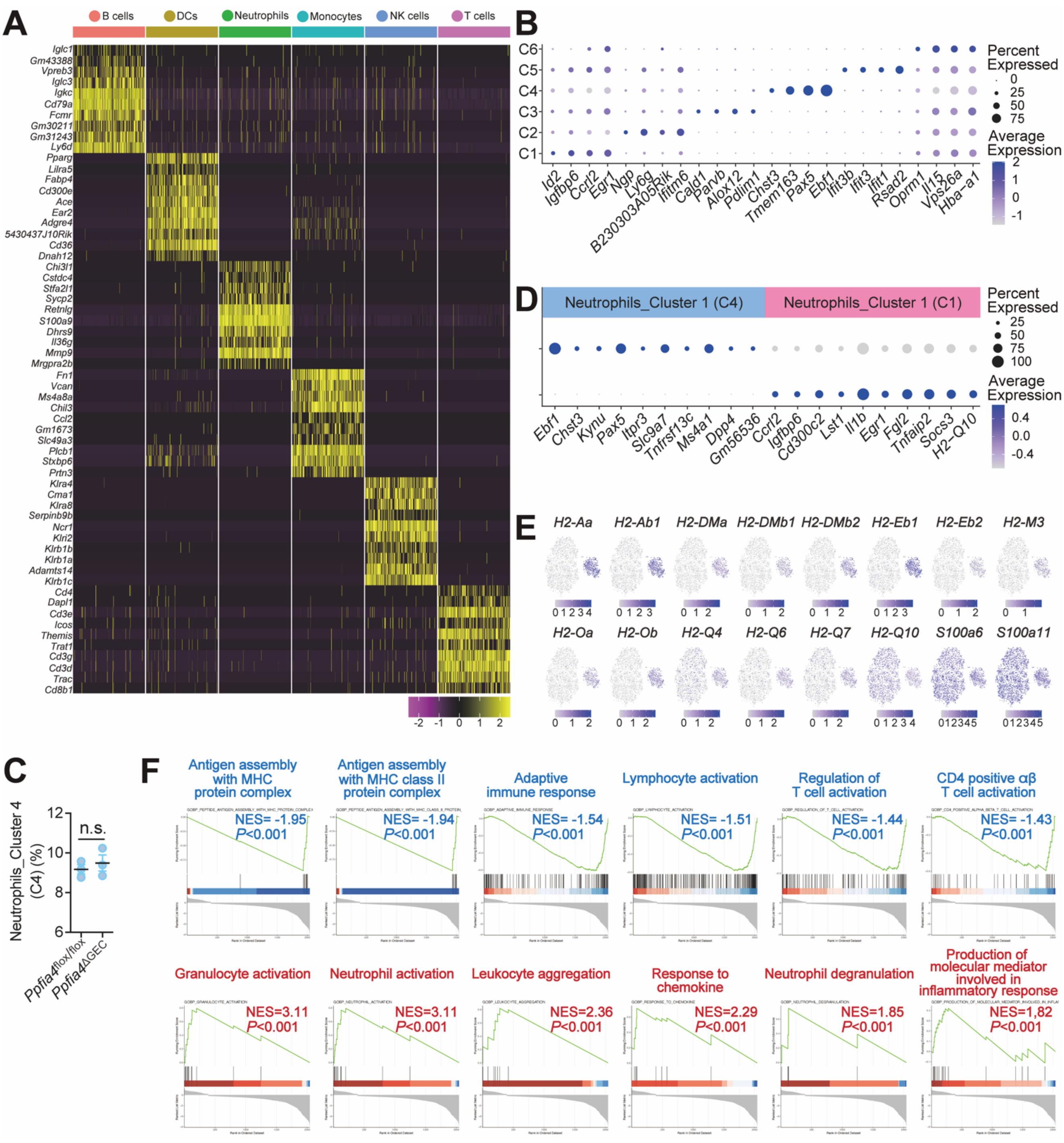

PPFIA4 promotes gastric G-MDSC accumulation during *H. pylori* infection. (A) Heatmaps showing the annotation and color codes for immune cell types in gastric mucosa of *H. pylori*-infected *Ppfia4*$^{\Delta GEC}$ mice and *Ppfia4*$^{flox/flox}$ littermates 15 weeks p.i.. (B) Dot plots showing the selected gene expression in neutrophil clusters (Neutrophils_Cluster 1 (C1), Neutrophils_Cluster 2 (C2), Neutrophils_Cluster 3 (C3), Neutrophils_Cluster 4 (C4), Neutrophils_Cluster 5 (C5), Neutrophils_Cluster 6 (C6)) and percentage of these cells expressing genes in neutrophil populations. (C) Statistical analysis of C4 level in gastric mucosa of *H. pylori*-infected *Ppfia4*$^{\Delta GEC}$ mice and *Ppfia4*$^{flox/flox}$ littermates 15 weeks p.i. (n=3). (D) Dot plots showing the selected gene expression

between C1 and C4 and percentage of these cells expressing genes in neutrophil populations. (E) T-distributed stochastic neighbor embedding (tSNE) plots showing the expression of the indicated genes in 2 neutrophil clusters (C1, C4). (F) Gene set enrichment analysis of enriched pathways in C1 vs C4. Data are presented as mean ± SEM. Statistics: unpaired 2-tailed t test (C). n.s. $P>0.05$ for groups connected by horizontal lines.

Supplemental Figure 7

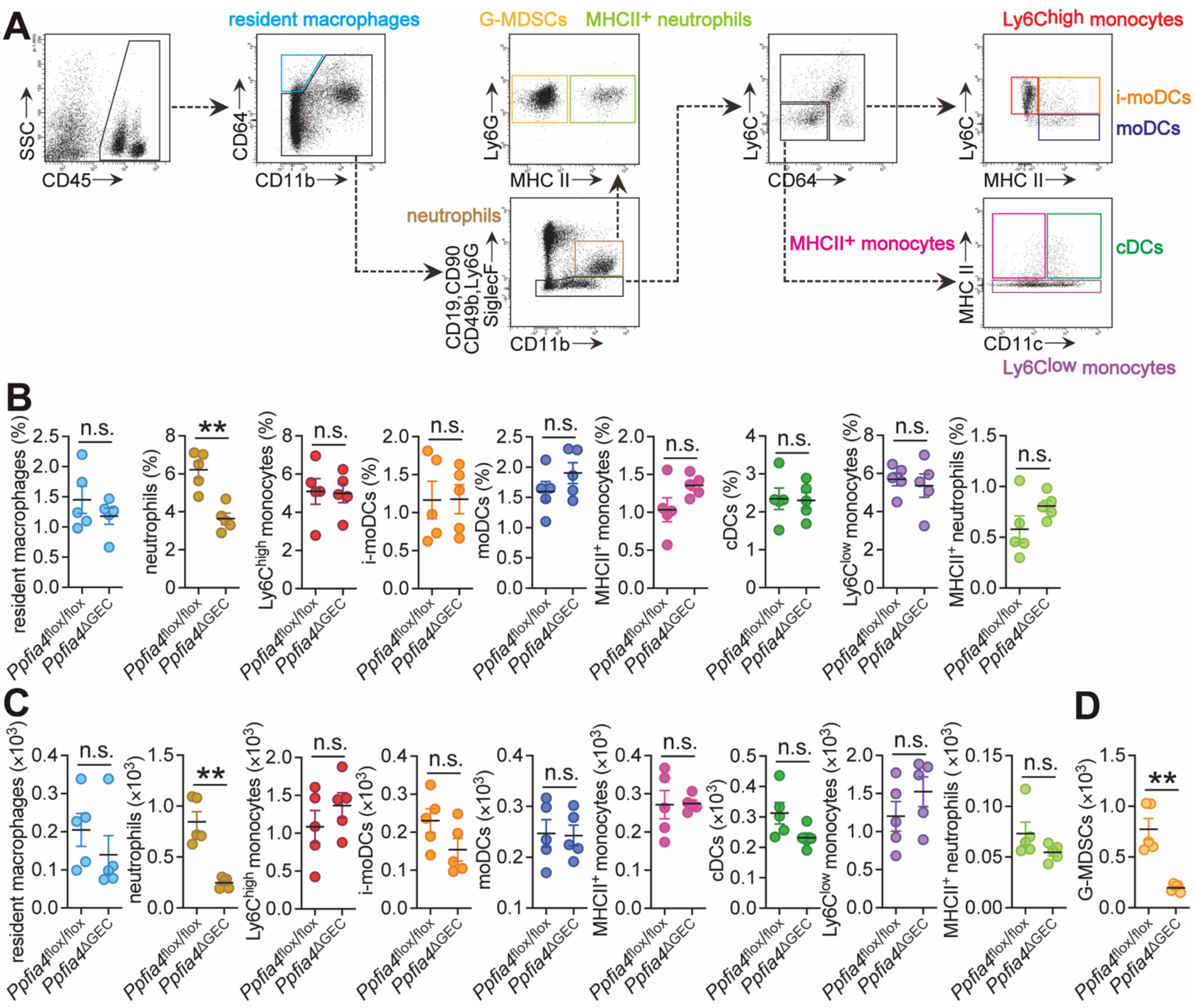

PPFIA4 promotes gastric G-MDSC accumulation during *H. pylori* infection. (A) Characterization by flow cytometry of tissue-resident macrophages, MHCII^{+} neutrophils, granulocytic myeloid-derived suppressor cells (G-MDSCs), Ly6C^{low} monocytes, Ly6C^{high} monocytes, MHCII^{+} monocytes, monocyte-derived DCs (moDCs), immature moDCs (i-moDCs) and conventional DCs (cDCs) in the mouse stomach. (B and C) The levels of tissue-resident macrophages, MHCII^{+} neutrophils, Ly6C^{low} monocytes, Ly6C^{high} monocytes, MHCII^{+} monocytes, moDCs, i-moDCs and cDCs in gastric mucosa of *H. pylori*-infected *Ppfia4*$^{\Delta GEC}$ mice and *Ppfia4*$^{flox/flox}$ littermates 15 weeks p.i. were compared (n=5). Results are expressed as the percentage of tissue-resident macrophages, MHCII^{+} neutrophils, Ly6C^{low} monocytes, Ly6C^{high} monocytes, MHCII^{+} monocytes, moDCs, i-moDCs and cDCs among all CD45^{+} cells (B) or the number of tissue-resident macrophages, MHCII^{+} neutrophils, Ly6C^{low} monocytes, Ly6C^{high} monocytes, MHCII^{+} monocytes, moDCs, i-moDCs and cDCs per million total cells (C). (D) The levels of G-MDSCs in gastric mucosa of *H.*

pylori-infected *Ppfia4*$^{\Delta GEC}$ mice and *Ppfia4*$^{flox/flox}$ littermates 15 weeks p.i. were compared (n=5). Results are expressed as the number of G-MDSCs per million total cells. Data are presented as mean ± SEM. Statistics: unpaired 2-tailed t test (B-D). ***P*<0.01, n.s. *P*>0.05 for groups connected by horizontal lines.

Supplemental Figure 8

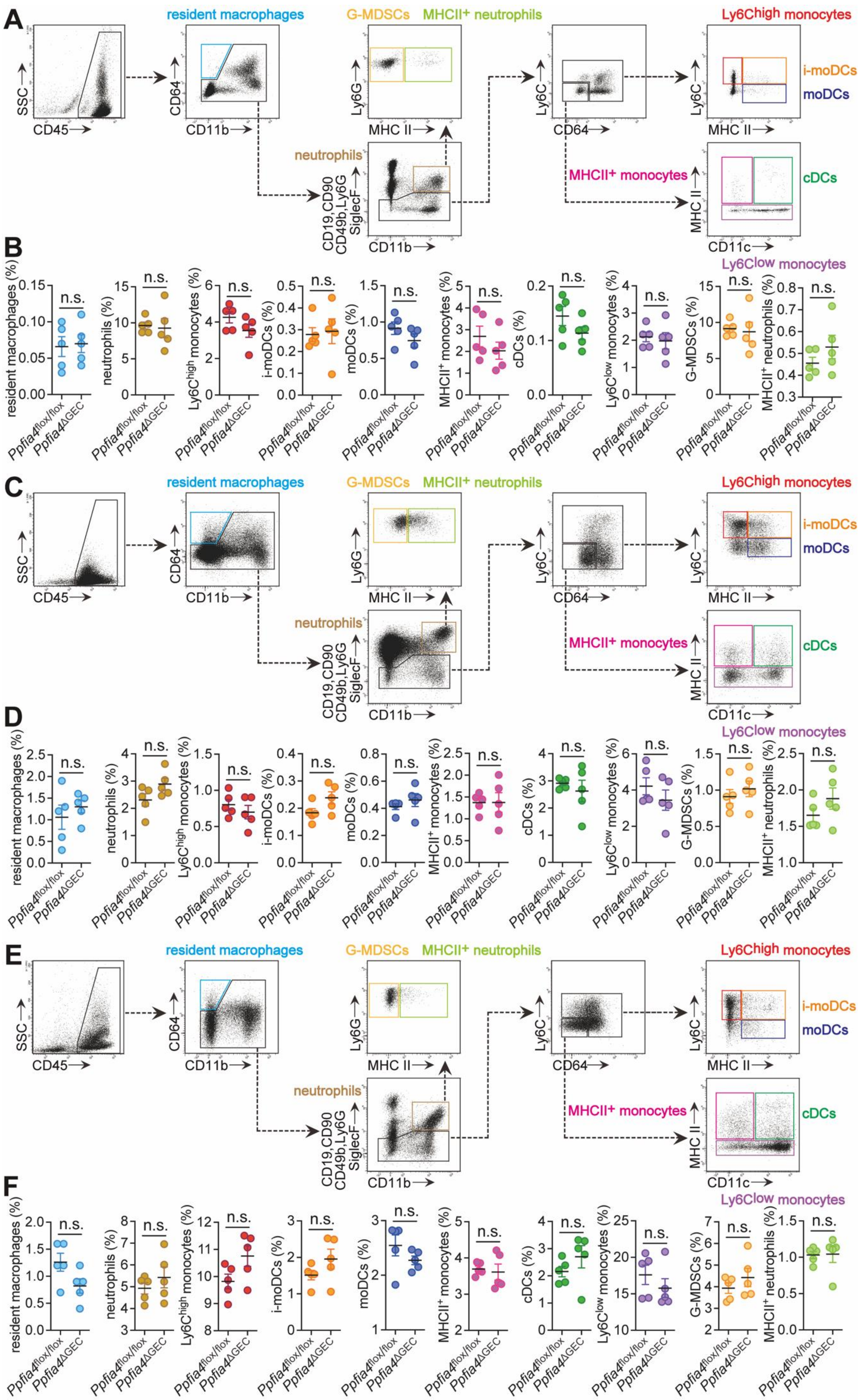

PPFIA4 has no effect on the accumulation of myeloid cells in blood, bone marrow and spleen during *H. pylori* infection. (A, C, E) Characterization by flow cytometry of tissue-resident macrophages, MHCII^{+} neutrophils, granulocytic myeloid-derived suppressor cells (G-MDSCs), Ly6C^{low} monocytes, Ly6C^{high} monocytes, MHCII^{+} monocytes, monocyte-derived DCs (moDCs), immature moDCs (i-moDCs) and conventional DCs (cDCs) in the mouse blood (A), bone marrow (C) and spleen (E). (B, D, F) The levels of tissue-resident macrophages, MHCII^{+} neutrophils, G-MDSCs, Ly6C^{low} monocytes, Ly6C^{high} monocytes, MHCII^{+} monocytes, moDCs, i-moDCs and cDCs in blood (B), bone marrow (D) and spleen (F) of *H. pylori*-infected *Ppfia4*$^{\Delta GEC}$ mice and *Ppfia4*$^{flox/flox}$ littermates 15 weeks p.i. were compared (n=5). Results are expressed as the percentage of tissue-resident macrophages, MHCII^{+} neutrophils, G-MDSCs, Ly6C^{low} monocytes, Ly6C^{high} monocytes, MHCII^{+} monocytes, moDCs, i-moDCs and cDCs among all CD45^{+} cells. Data are presented as mean ± SEM. Statistics: unpaired 2-tailed t test (B, D and F). n.s. *P*>0.05 for groups connected by horizontal lines.

Supplemental Figure 9

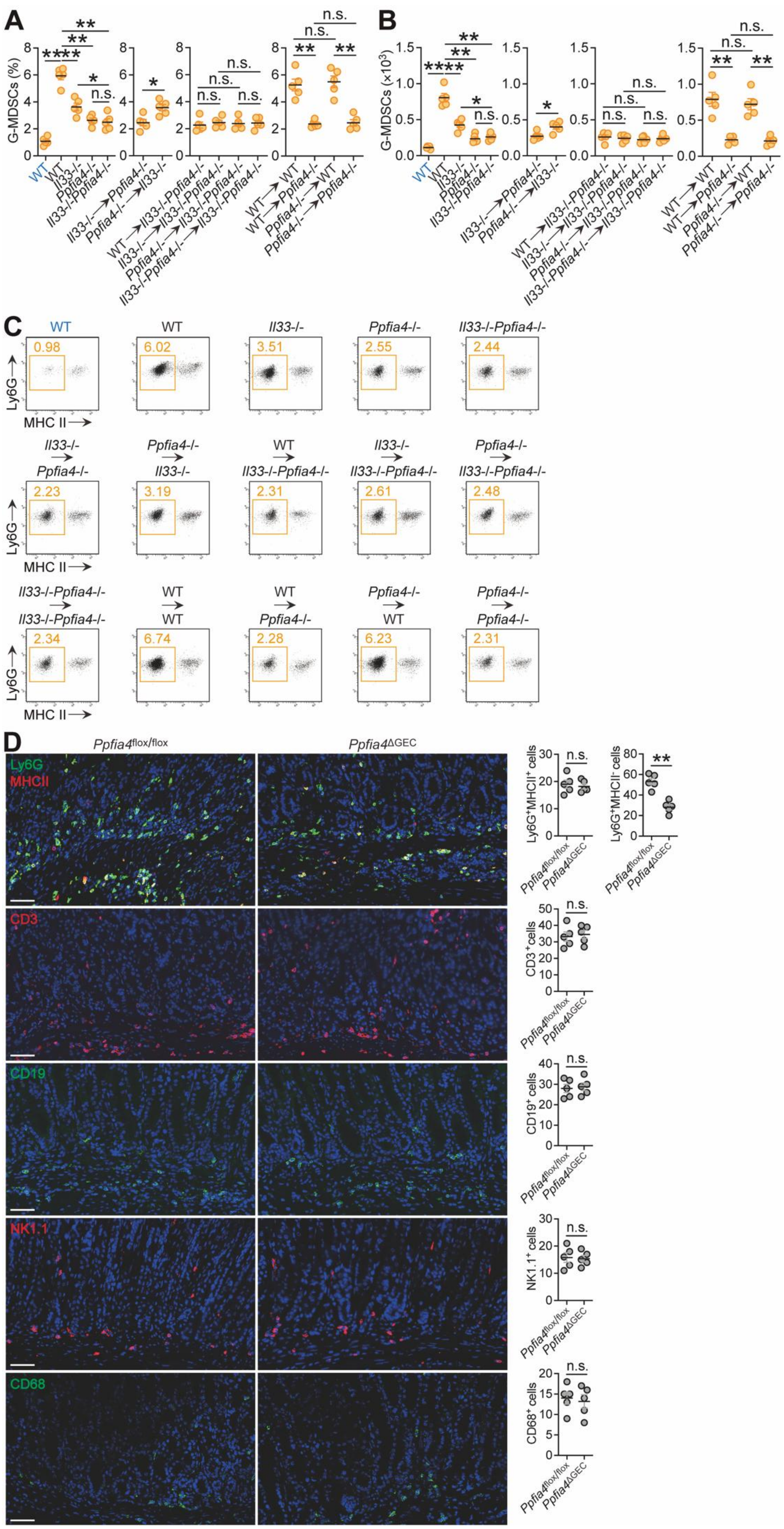

PPFIA4 promotes gastric G-MDSC accumulation during *H. pylori* infection. (A and B) The levels of G-MDSCs in gastric mucosa of uninfected WT mice (blue), *H. pylori*-infected WT, *Il33*$^{-/-}$, *Ppfia4*$^{-/-}$ and *Il33*$^{-/-}$*Ppfia4*$^{-/-}$ mice, or in gastric mucosa of *H. pylori*-infected BM chimera mice 15 weeks p.i. were compared (n=5). Results are expressed as the percentage of G-MDSCs among all CD45$^+$ cells (A) or the number of G-MDSCs per million total cells (B). (C) Representative dot plots of G-MDSCs in gastric mucosa of uninfected WT mice (blue), *H. pylori*-infected WT, *Il33*$^{-/-}$, *Ppfia4*$^{-/-}$ and *Il33*$^{-/-}$*Ppfia4*$^{-/-}$ mice, or in gastric mucosa of *H. pylori*-infected BM chimera mice 15 weeks p.i.. Results are expressed as the percentage of G-MDSCs among all CD45$^+$ cells. (D) Immunofluorescence showed immune cell (including LyG$^+$MHCII$^+$ neutrophils, LyG$^+$MHCII$^-$ neutrophils, CD3$^+$ T cells, CD19$^+$ B cells, NK1.1$^+$ NK cells, CD68$^+$ macrophages) infiltration in gastric mucosa of *H. pylori*-infected *Ppfia4*$^{\Delta GEC}$ mice and *Ppfia4*$^{flox/flox}$ littermates 15 weeks p.i.. Scale bars: 50 microns. Quantitation of various immune cells per ×40 field in gastric mucosa of *H. pylori*-infected *Ppfia4*$^{\Delta GEC}$ mice and *Ppfia4*$^{flox/flox}$ littermates 15 weeks p.i. (n=5). Data are presented as mean ± SEM. Statistics: unpaired 2-tailed t test (A, B and D). **P*<0.05, ***P*<0.01, n.s. *P*>0.05 for groups connected by horizontal lines.

1 Supplemental Figure 10

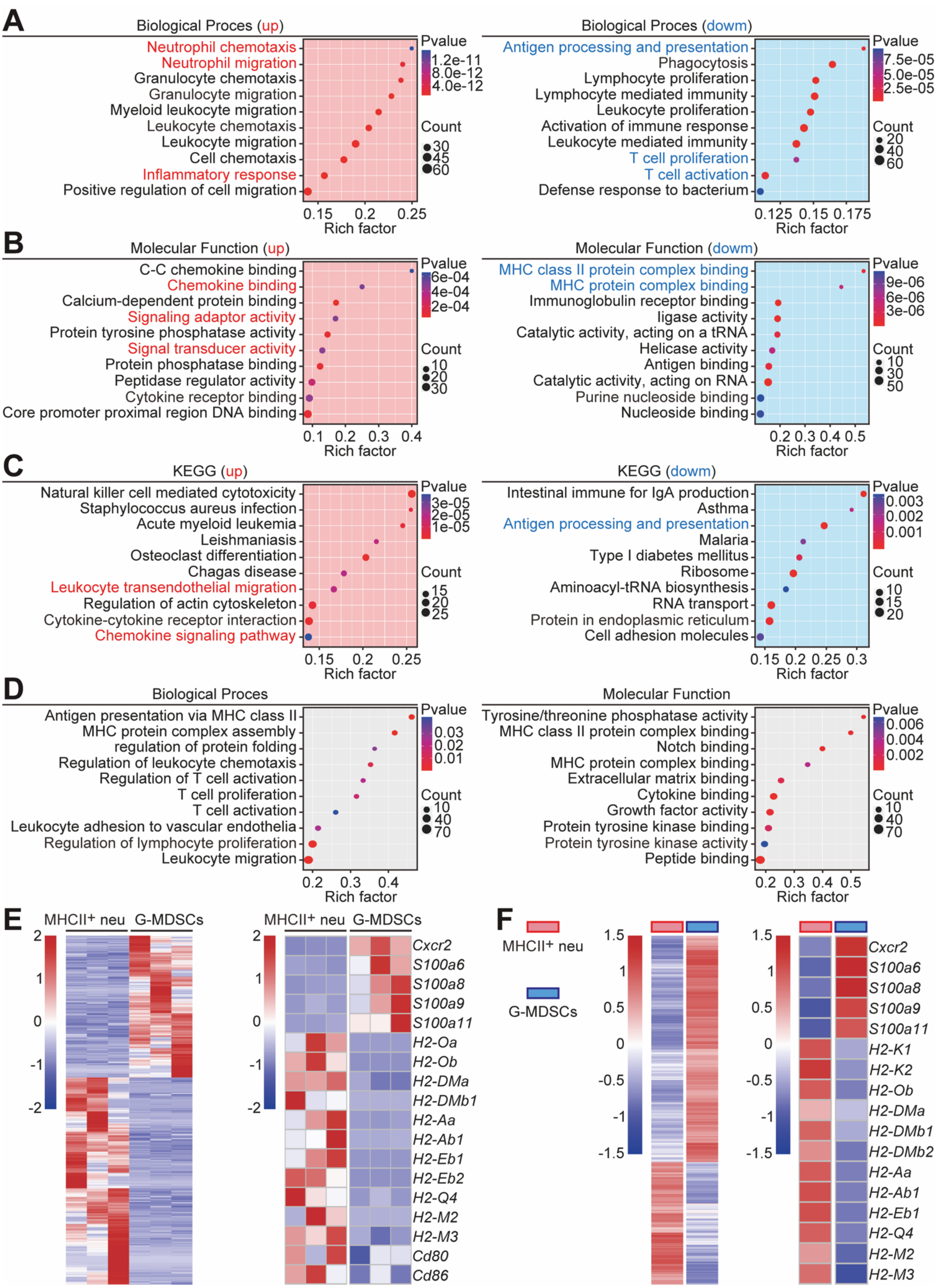

2

3 PPFIA4 promotes gastric G-MDSC accumulation during *H. pylori* infection. (A-C) Compared to MHCII⁺

neutrophils, significantly changed genes in G-MDSCs in gastric mucosa of *H. pylori*-infected WT mice 15 weeks p.i. were clustered with gene ontology (GO) analysis by Digital RNA with pertUrbation of Genes sequencing (DRUG-seq), and the significant upregulated or downregulated top 10 GO terms of “Biological Process” (A) and “Molecular Function” (B) as well as the significant upregulated or downregulated top 10 KEGG pathways (C) were shown. (D) Compared to MHCII$^+$ neutrophils, significantly changed genes in G-MDSCs in gastric mucosa of *H. pylori*-infected WT mice 15 weeks p.i. were clustered with GO analysis by Switching Mechanism At the 5' end of RNA Template sequencing (SMART-seq), and the significant changed top 10 GO terms of “Biological Process” and “Molecular Function” were shown. (E) Heatmaps showing the expression of all the genes as well as the indicated genes in MHCII$^+$ neutrophils and G-MDSCs in gastric mucosa of *H. pylori*-infected WT mice 15 weeks p.i. by DRUG-seq (n=3). (F) Heatmaps showing the expression of all the genes as well as the indicated genes in MHCII$^+$ neutrophils and G-MDSCs in gastric mucosa of *H. pylori*-infected WT mice 15 weeks p.i. by SMART-seq.

Supplemental Figure 11

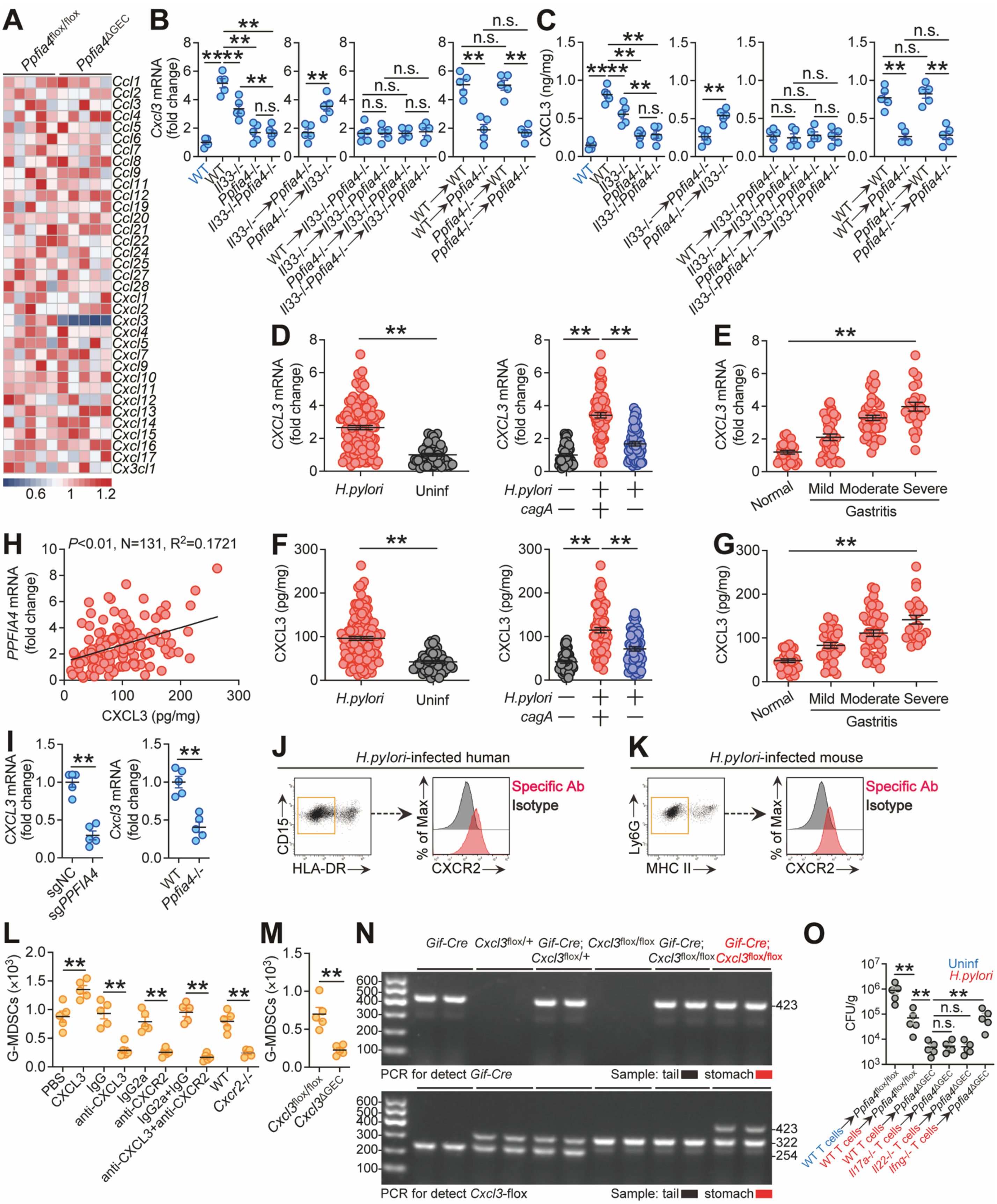

PPFIA4 increases gastritis by promoting G-MDSC accumulation via CXCL3 during *H. pylori* infection. (A) The expression of chemokine family members in gastric mucosa of *H. pylori*-infected *Ppfia4*$^{\Delta GEC}$ mice and *Ppfia4*$^{flox/flox}$ littermates 15 weeks p.i. was analyzed by real-time PCR (n=5). (B and C) *Cxcl3* expression (B)

and CXCL3 protein (C) in gastric mucosa of uninfected WT mice (blue), *H. pylori*-infected WT, *Il33*$^{-/-}$, *Ppfia4*$^{-/-}$ and *Il33*$^{-/-}$*Ppfia4*$^{-/-}$ mice, or in gastric mucosa of *H. pylori*-infected BM chimera mice 15 weeks p.i. were compared (n=5). (D and F) *CXCL3* expression (D) and CXCL3 protein (F) in gastric mucosa of *H. pylori*-infected (n=131) and uninfected donors (n=50), or in gastric mucosa of *cagA*-positive *H. pylori*-infected (n=74), *cagA*-negative *H. pylori*-infected (n=57) and uninfected donors (n=50) were compared. (E and G) *CXCL3* expression (E) and CXCL3 protein (G) in gastric mucosa of *H. pylori*-infected patients with mild (n=34), moderate (n=45), severe inflammation (n=24), and with normal gastric histopathology (n=28) were compared. (H) The correlation between *PPFIA4* expression and CXCL3 protein in gastric mucosa of *H. pylori*-infected patients was analyzed. (I) sg*PPFIA4*-modified or non-specific control sgRNA (sgNC)-modified AGS cells, and primary gastric epithelial cells (GECs) from uninfected WT and *Ppfia4*$^{-/-}$ mice were stimulated with *H. pylori* (MOI=100) for 24 h. *CXCL3*/*Cxcl3* expression was analyzed by real-time PCR (n=5). (J) CXCR2 expression on G-MDSCs in blood of *H. pylori*-infected patients. (K) CXCR2 expression on G-MDSCs in blood of *H. pylori*-infected WT mice 15 weeks p.i.. (L and M) The G-MDSC level in gastric mucosa of *H. pylori*-infected WT mice injected with CXCL3 or PBS control, or anti-CXCL3 Abs, anti-CXCR2 Abs and/or control IgG, or in gastric mucosa of *H. pylori*-infected WT and *Cxcr2*$^{-/-}$ mice 15 weeks p.i. (L), or in gastric mucosa of *H. pylori*-infected *Cxcl3*$^{\Delta GEC}$ mice and *Cxcl3*$^{flox/flox}$ littermates 15 weeks p.i. (M) was compared (n=5). Results are expressed as the number of G-MDSCs per million total cells. (N) PCR genotype analysis of pups generated by crossing *Cxcl3*$^{flox/flox}$ mice with *Gif-Cre* mice to obtain *Cxcl3*$^{\Delta GEC}$ mice (*Gif-Cre*;*Cxcl3*$^{flox/flox}$) using specific primers (Supplemental Table 7). (O) The bacteria colonization in gastric mucosa of *H. pylori*-infected *Ppfia4*$^{\Delta GEC}$ mice and *Ppfia4*$^{flox/flox}$ littermates adoptively transferred with spleen CD4^{+} T cells from uninfected or *H. pylori*-infected WT, *Il17a*$^{-/-}$, *Il22*$^{-/-}$ or *Ifng*$^{-/-}$ mice (15 weeks p.i.) was compared 15 weeks p.i. (n=5). The bacteria colonization is shown as CFU per gram of stomach tissue by bacterial reisolation and quantitative culture. Data are presented as mean ± SEM. Statistics: unpaired 2-tailed t test (B-D, F, I, L, M and O), 1-way ANOVA test (E and G) and 2-tailed Pearson correlation test (H). *$P<0.05$, **$P<0.01$, n.s. $P>0.05$ for groups connected by horizontal lines.

Supplemental Figure 12

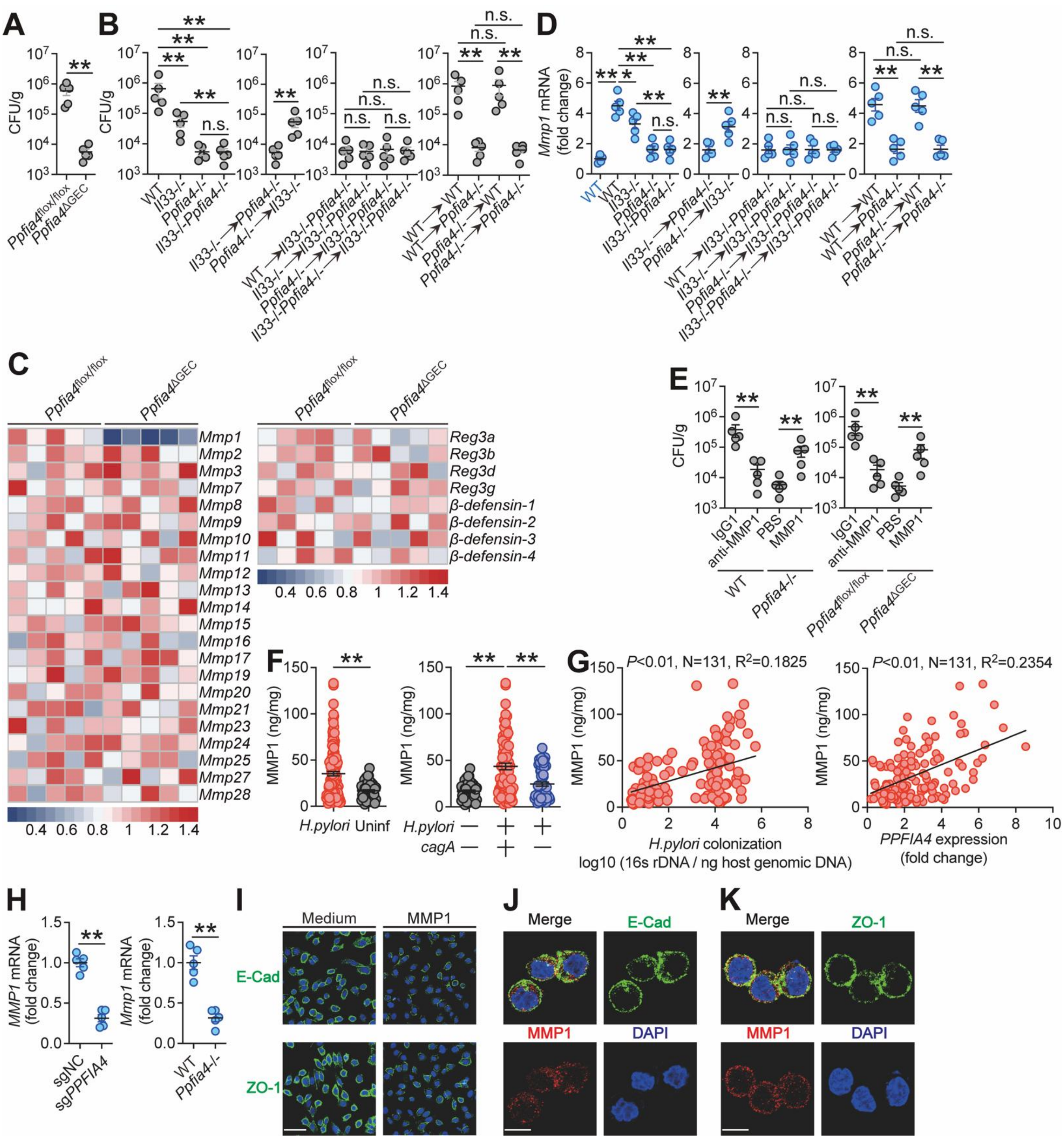

PPFIA4 promotes MMP1 expression leading to increased bacterial burden and gastric mucosal damage during *H. pylori* infection. (A and B) The bacteria colonization in gastric mucosa of *H. pylori*-infected *Ppfia4*$^{\Delta GEC}$ mice and *Ppfia4*$^{flox/flox}$ littermates (A), or in gastric mucosa of *H. pylori*-infected WT, *Il33*$^{-/-}$, *Ppfia4*$^{-/-}$ and *Il33*$^{-/-}$*Ppfia4*$^{-/-}$ mice and BM chimera mice (B) 15 weeks p.i. was analyzed (n=5). The bacteria colonization is shown as CFU per gram of stomach tissue by bacterial reisolation and quantitative culture. (C) The expressions of *Mmps*, *β-defensins* and *Reg3s* in gastric mucosa of *H. pylori*-infected *Ppfia4*$^{\Delta GEC}$ mice and

Ppfia4$^{flox/flox}$ littermates 15 weeks p.i. were analyzed by real-time PCR (n=5). (D) *Mmp1* expression in gastric mucosa of uninfected WT mice (blue), *H. pylori*-infected WT, *Il33*$^{-/-}$, *Ppfia4*$^{-/-}$ and *Il33*$^{-/-}$*Ppfia4*$^{-/-}$ mice, or in gastric mucosa of *H. pylori*-infected BM chimera mice 15 weeks p.i. was compared (n=5). (E) The bacteria colonization in gastric mucosa of *H. pylori*-infected WT mice or *Ppfia4*$^{flox/flox}$ littermates injected with anti-MMP1 Abs or control IgG, or in gastric mucosa of *H. pylori*-infected *Ppfia4*$^{-/-}$ mice or *Ppfia4*$^{\Delta GEC}$ mice injected with MMP1 or PBS control 15 weeks p.i. were analyzed (n=5). The bacteria colonization is shown as CFU per gram of stomach tissue by bacterial reisolation and quantitative culture. (F) MMP1 protein in gastric mucosa of *H. pylori*-infected (n=131) and uninfected donors (n=50), or in gastric mucosa of *cagA*-positive *H. pylori*-infected (n=74), *cagA*-negative *H. pylori*-infected (n=57) and uninfected donors (n=50) was compared. (G) The correlation between MMP1 protein and *H. pylori* colonization or between MMP1 protein and *PPFIA4* expression in gastric mucosa of *H. pylori*-infected patients was analyzed. (H) sg*PPFIA4*-modified or non-specific control sgRNA (sgNC)-modified AGS cells, and primary gastric epithelial cells (GECs) from uninfected WT and *Ppfia4*$^{-/-}$ mice were stimulated with *H. pylori* (MOI=100) for 24 h. *MMP1*/*Mmp1* expression was analyzed by real-time PCR (n=5). (I) Immunofluorescence showed E-cadherin and ZO-1 proteins in AGS treated with MMP1 (1 μg/ml, 24 h). Scale bars: 50 microns. (J and K) Immunofluorescence showed MMP1/E-cadherin binding (J) and MMP1/ZO-1 binding (K) on AGS cells treated with MMP1 (1 μg/ml, 3 h, 4°C). Scale bars: 10 microns. Data are presented as mean ± SEM. Statistics: unpaired 2-tailed t test (A, B, D-F, and H) and 2-tailed Pearson correlation test (G). **P*<0.05, ***P*<0.01, n.s. *P*>0.05 for groups connected by horizontal lines.

Supplemental Figure 13

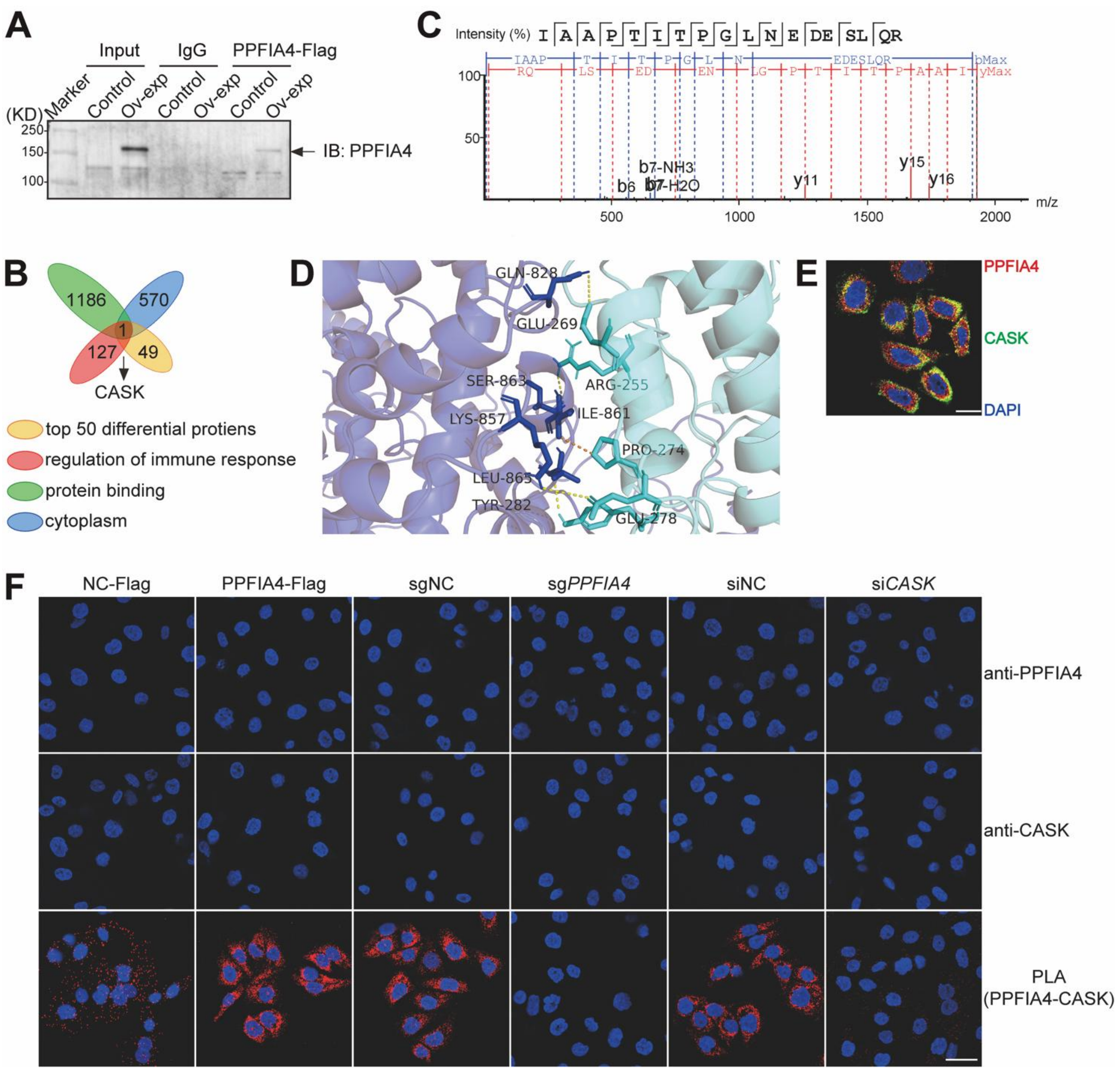

PPFIA4 binds to CASK during *H. pylori* infection. (A) AGS cells expressing the Flag-tagged PPFIA4 (PPFIA4-Flag) were lysed and immunoprecipitated (IP) with IgG (control) or anti-Flag Abs. The IP samples were subjected to western blot before mass spectrometry analysis. (B) The members of the 3 gene ontology (GO) terms in “Cellular Component”, “Biological Process”, “Molecular Function” respectively and “top 50 differential proteins” had overlapped by mass spectrometry analysis of AGS cells expressing PPFIA4-Flag IP with anti-Flag Abs. CASK was the core member by overlapping. (C) Mass spectrometry profile of the PPFIA4 interacting partner CASK. (D) The optimally predicted protein-protein complex obtained from HADDOCK’s easy interface: the key active residues involved in the interaction between PPFIA4 and CASK. (E) Immunofluorescence showed the PPFIA4/CASK co-localization in AGS cells expressing PPFIA4-Flag. Scale bars: 10 microns. (F) AGS cells expressing PPFIA4-Flag or non-specific control (NC)-Flag were cultured.

sg*PPFIA4*-modified or non-specific control sgRNA (sgNC)-modified AGS cells, and *CASK* siRNA (si*CASK*) or non-specific control siRNA (siNC) pre-treated AGS cells were stimulated with *H. pylori* (MOI=100) for 24 h. Proximity ligation assay in AGS cells was performed by using anti-PPFIA4 and anti-CASK Abs. Single Ab only was used as negative controls. Red dots represent close relationship between the 2 proteins. Scale bars: 30 microns.

Supplemental Figure 14

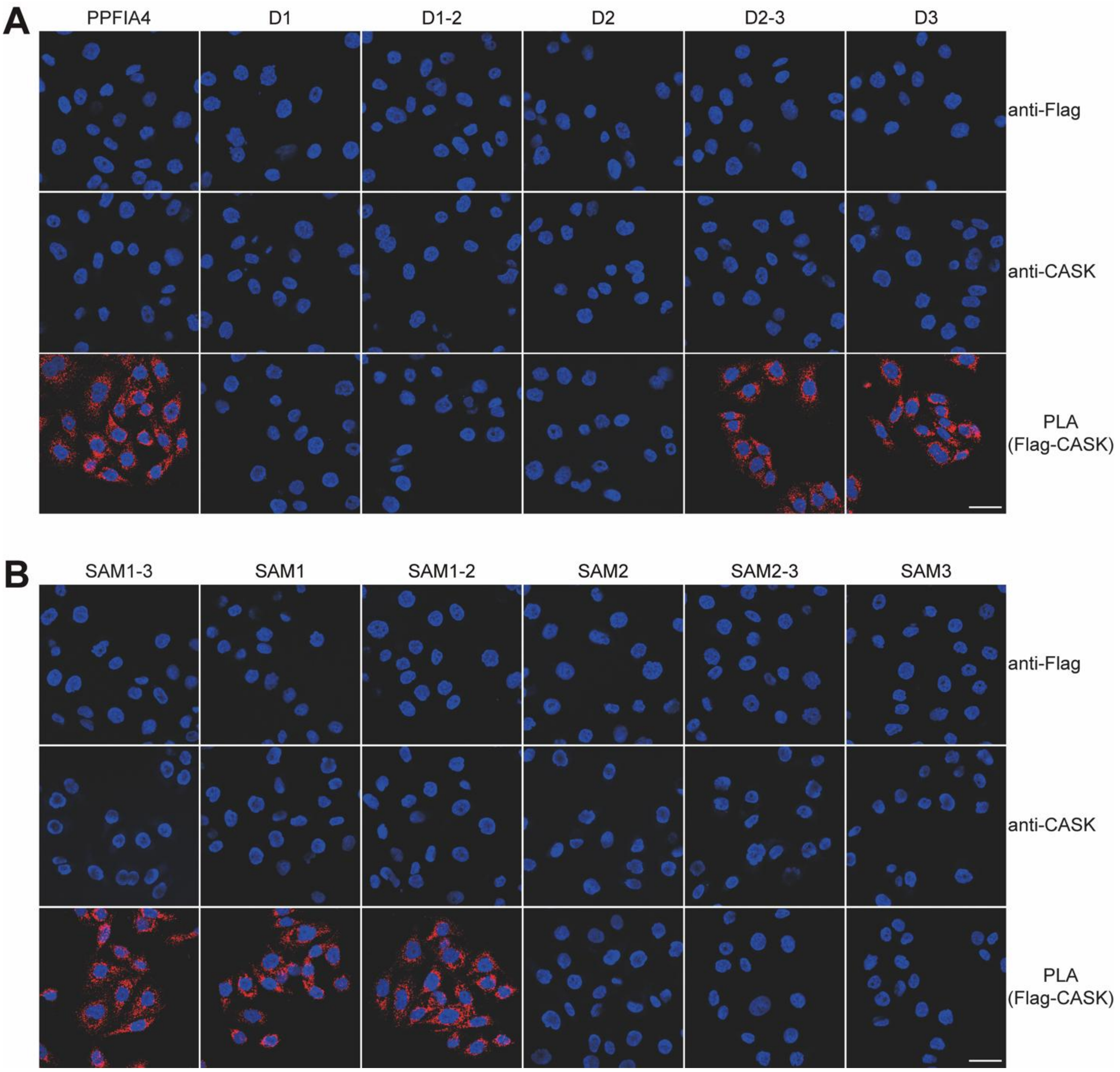

PPFIA4 domain SAM1 binds to domains of CamK to the first L27 of CASK during *H. pylori* infection. (A) Flag-tagged PPFIA4, D1, D1-2, D2, D2-3 and D3 were individually over-expressed in AGS cells. Proximity ligation assay in AGS cells was performed by using anti-Flag and anti-CASK Abs. Single Ab only was used as negative controls. Red dots represent close relationship between the 2 proteins. Scale bars: 30 microns. (B) Flag-tagged SAM1-3, SAM1, SAM1-2, SAM2, SAM2-3 and SAM3 were individually over-expressed in AGS cells. Proximity ligation assay in AGS cells was performed by using anti-Flag and anti-CASK Abs. Single Ab only was used as negative controls. Red dots represent close relationship between the 2 proteins. Scale bars: 30 microns.

Supplemental Figure 15

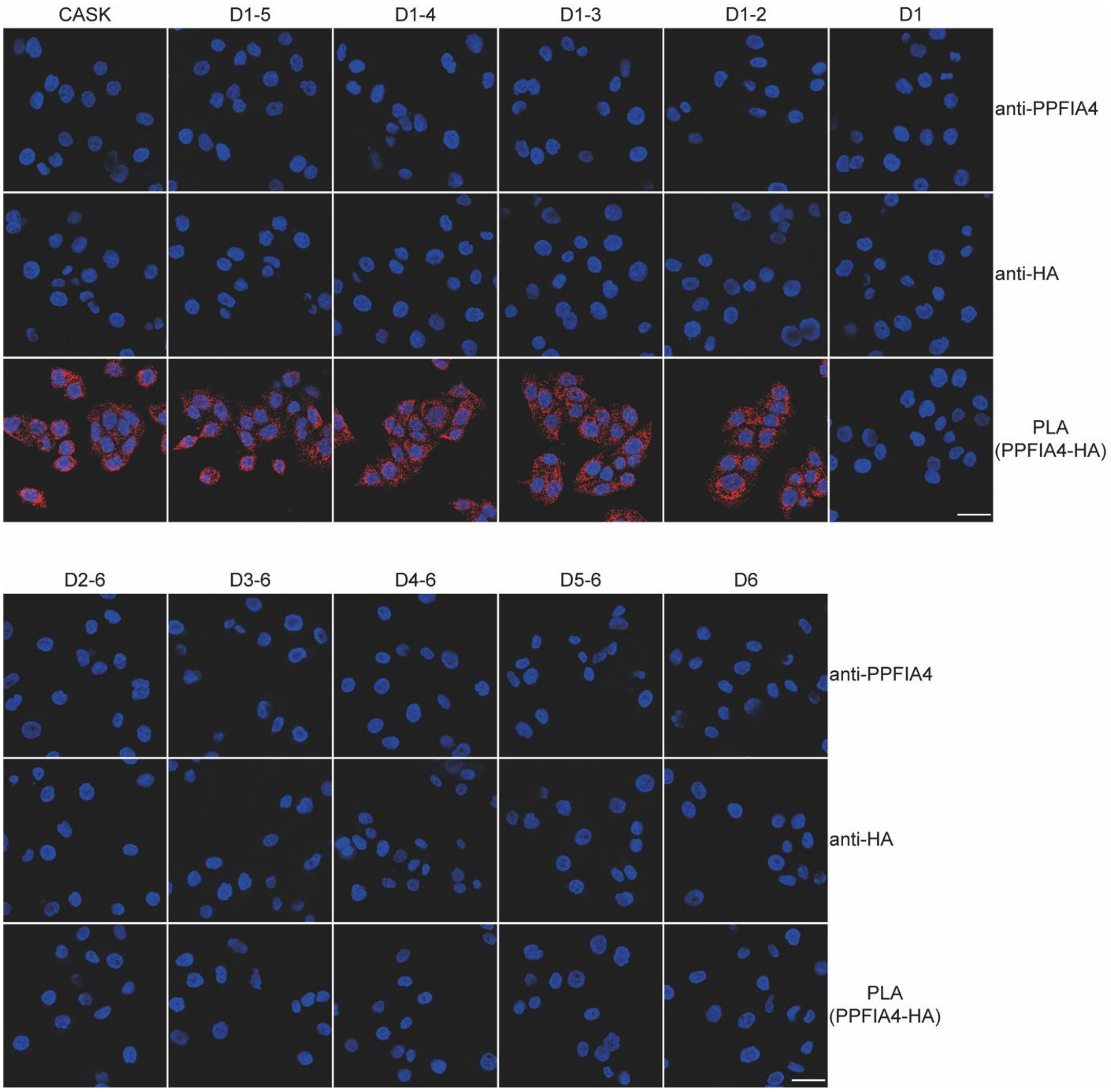

PPFIA4 domain SAM1 binds to domains of CamK to the first L27 of CASK during *H. pylori* infection. HA-tagged CASK, D1, D1-2, D1-3, D1-4, D1-5, D6, D5-6, D4-6, D3-6 and D2-6 were individually over-expressed in AGS cells. Proximity ligation assay in AGS cells was performed by using anti-HA and anti-PPFIA4 Abs. Single Ab only was used as negative controls. Red dots represent close relationship between the 2 proteins. Scale bars: 30 microns.

Supplemental Figure 16

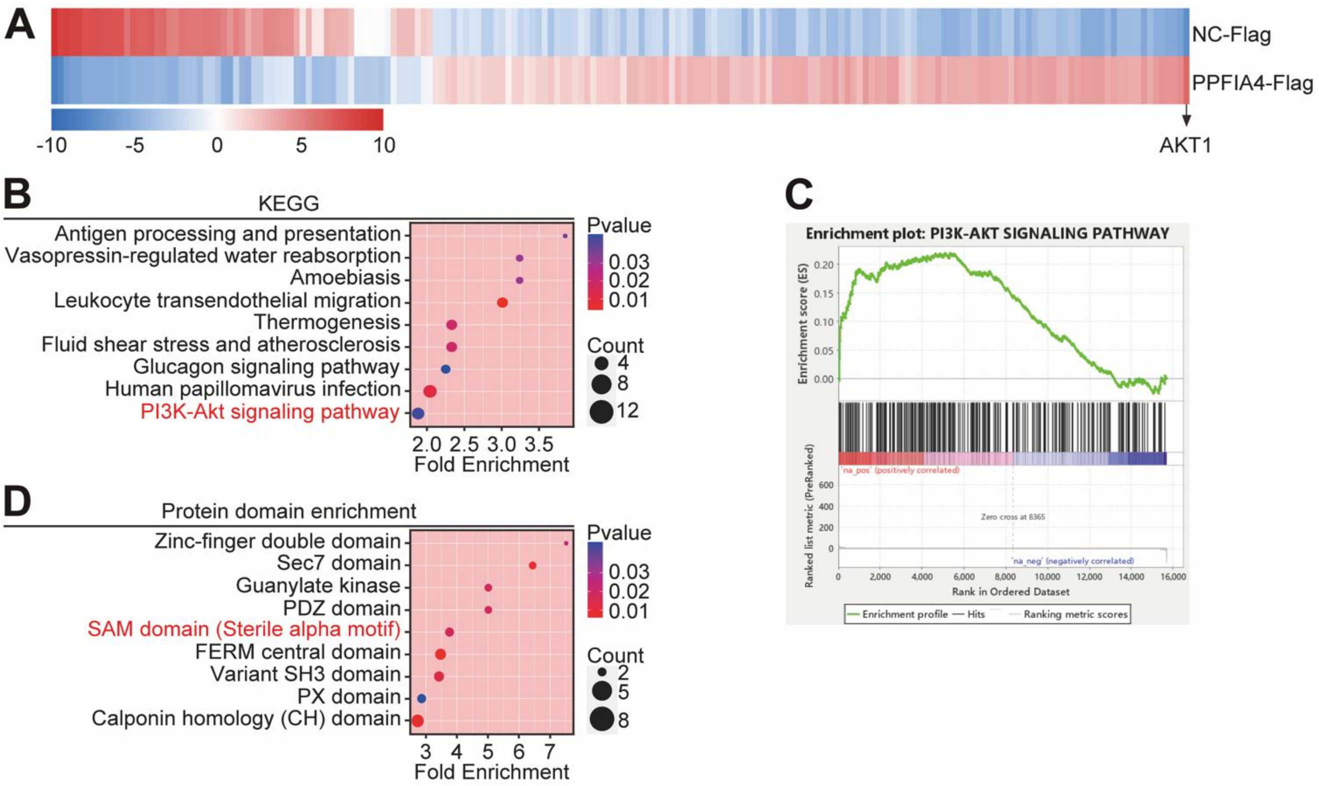

PPFIA4/CASK promotes NF-κB phosphorylation via interaction with and activation of AKT1 during *H. pylori* infection. (A) Heatmaps showing the differential expression of all the kinases in AGS cells expressing non-specific control (NC)-Flag and AGS cells expressing the Flag-tagged PPFIA4 (PPFIA4-Flag). (B and D) Compared to AGS cells expressing NC-Flag, the significantly changed phosphoproteins in AGS cells expressing PPFIA4-Flag were clustered with gene ontology (GO) analysis, and the top 9 KEGG pathways (B) as well as the top 9 GO terms of “Protein domain enrichment” (D) were shown. (C) Compared to AGS cells expressing NC-Flag, gene set enrichment analysis (GSEA) of the significantly changed phosphoproteins in AGS cells expressing PPFIA4-Flag identified the pathway “PI3K-AKT signaling pathway” by the significance levels (P<0.05).

Supplemental Figure 17

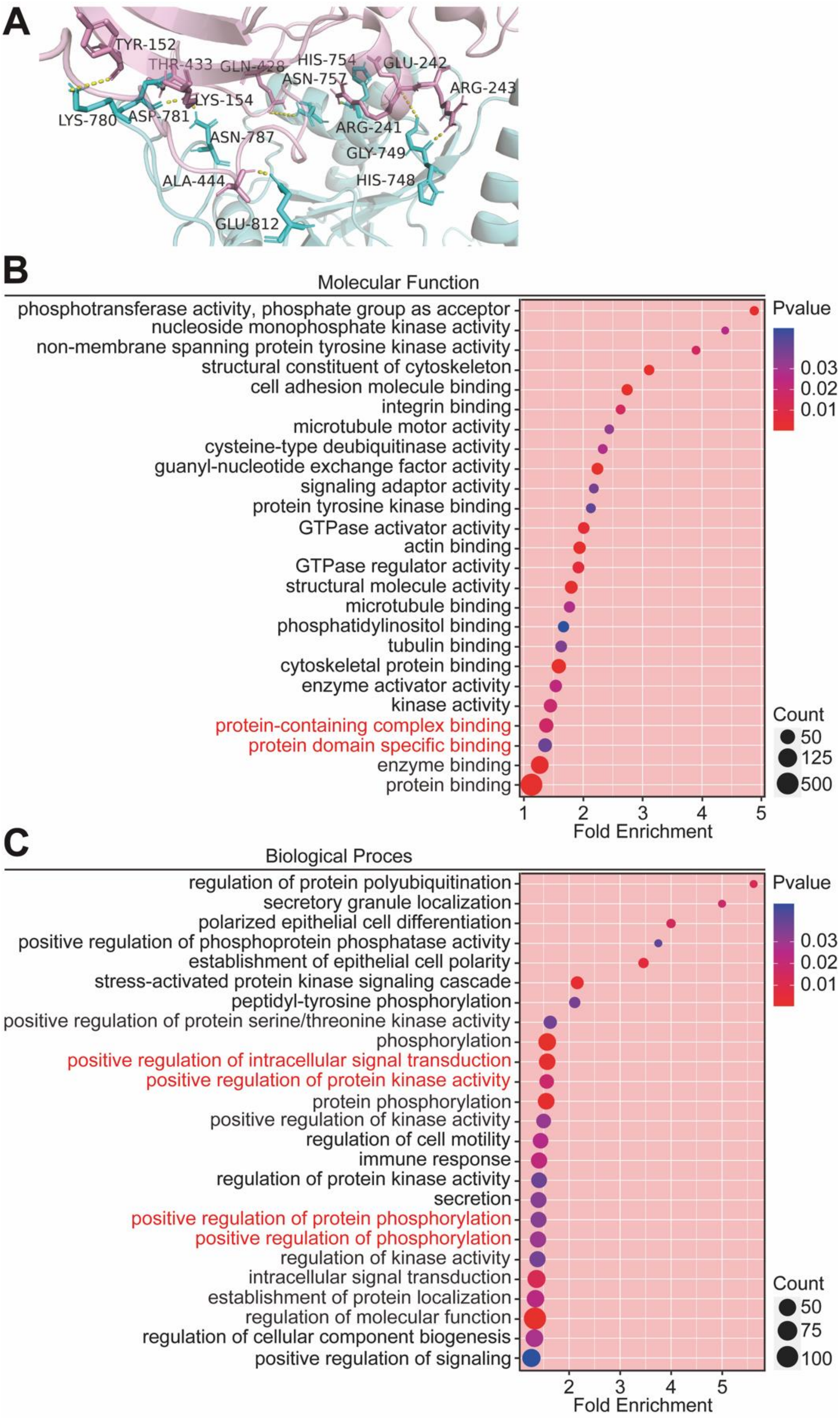

PPFIA4/CASK promotes NF-κB phosphorylation via interaction with and activation of AKT1 during *H. pylori* infection. (A) The optimally predicted protein-protein complex obtained from HADDOCK's easy interface: the key active residues involved in the interaction between CASK and AKT1. (B and C) Compared to AGS cells expressing NC-Flag, the significantly changed phosphoproteins in AGS cells expressing PPFIA4-Flag were clustered with gene ontology (GO) analysis, and the top 25 GO terms of "Molecular Function" (B) and

“Biological Process” (C) were shown.

Supplemental Figure 18

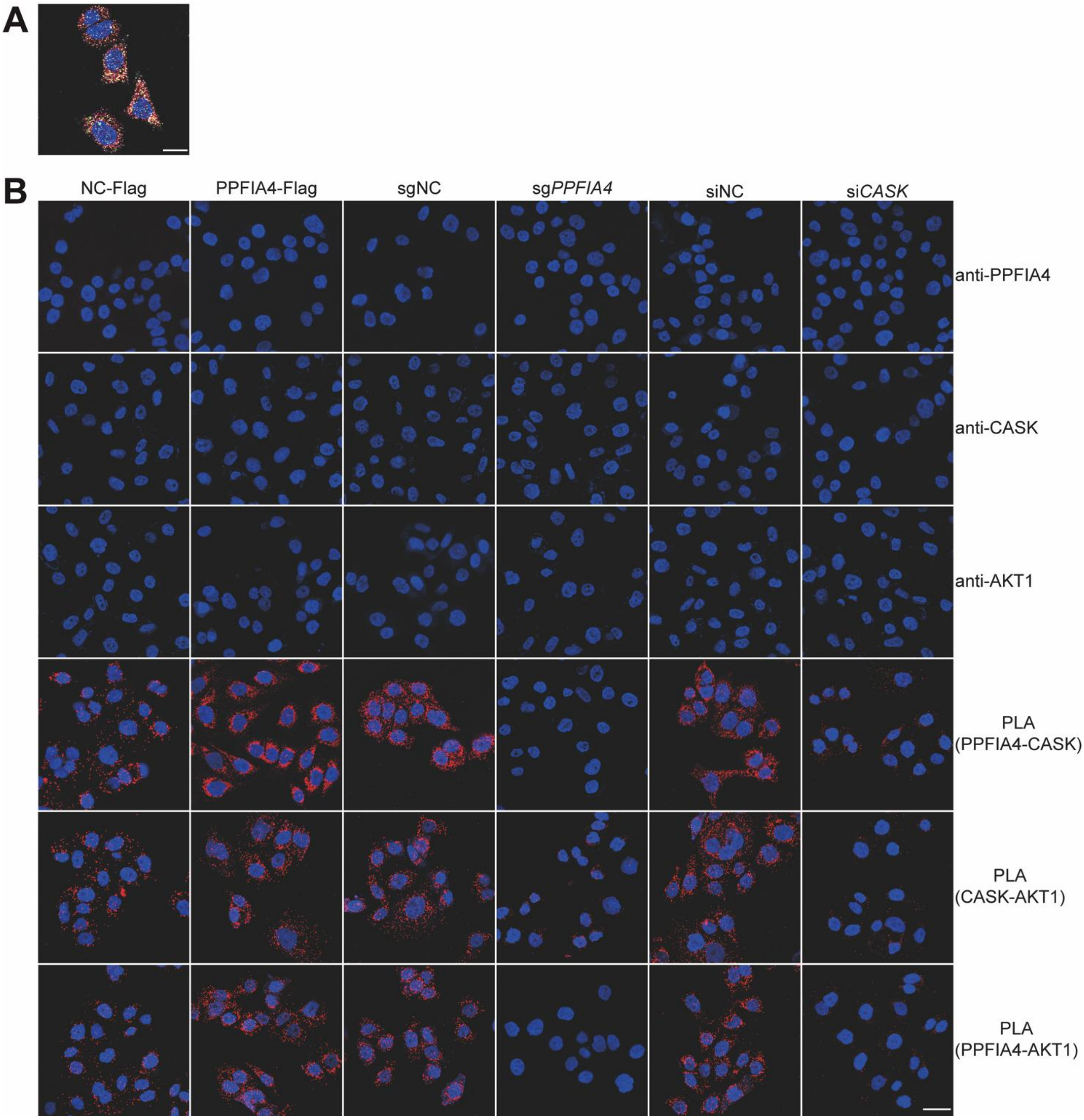

PPFIA4/CASK promotes NF-κB phosphorylation via interaction with and activation of AKT1 during *H. pylori* infection. (A) Immunofluorescence showed the PPFIA4/CASK/AKT1 co-localization in AGS cells expressing PPFIA4-Flag. Scale bars: 10 microns. (B) AGS cells expressing PPFIA4-Flag or non-specific control (NC)-Flag were cultured. sg*PPFIA4*-modified or non-specific control sgRNA (sgNC)-modified AGS cells, and *CASK* siRNA (si*CASK*) or non-specific control siRNA (siNC) pre-treated AGS cells were stimulated with *H. pylori* (MOI=100) for 24 h. Proximity ligation assay in AGS cells was performed by using anti-PPFIA4, anti-CASK and anti-AKT1 Abs. Single Ab only was used as negative controls. Red dots represent close relationship between the 2 proteins. Scale bars: 30 microns.

Supplemental Figure 19

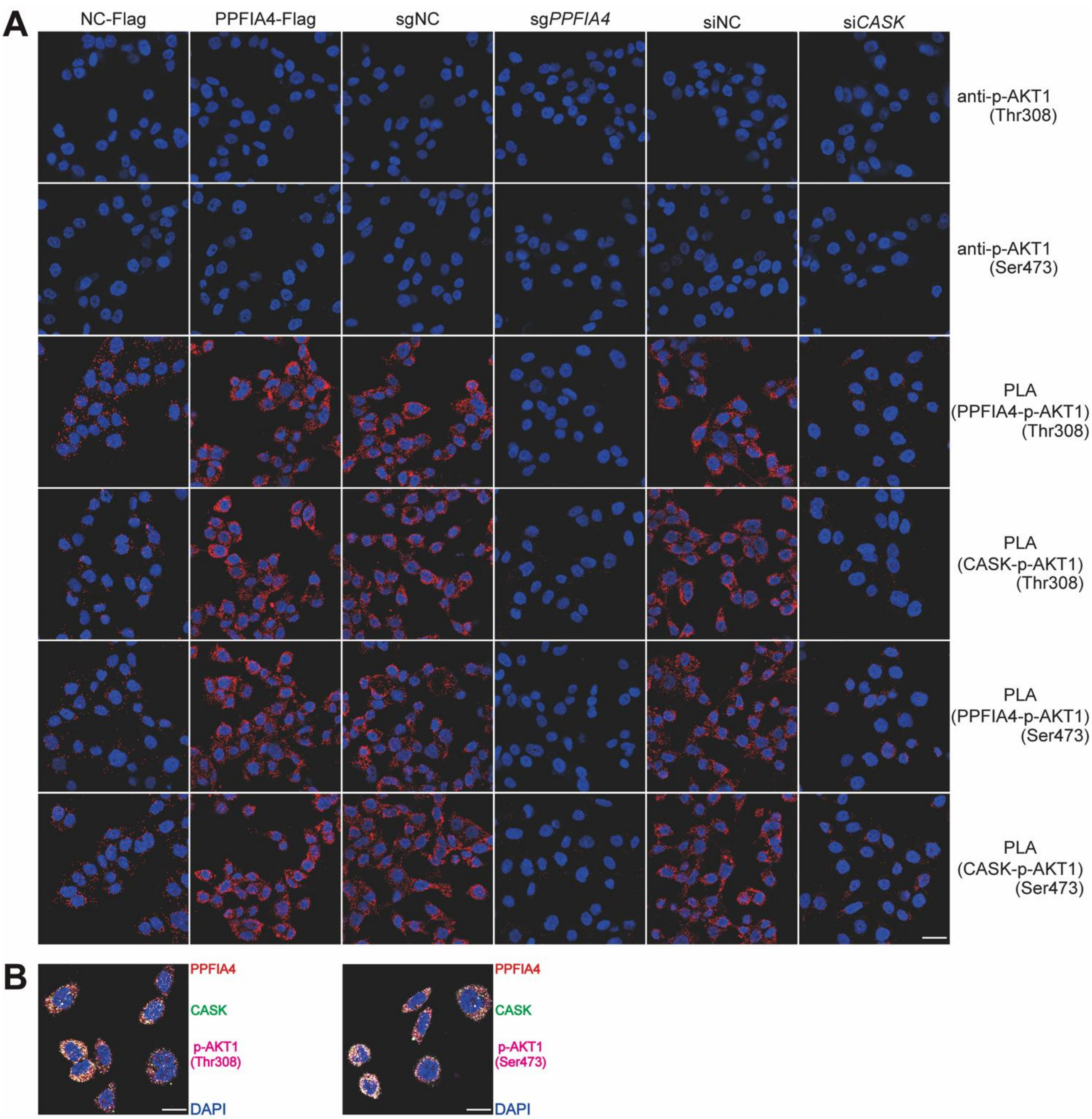

PPFIA4/CASK promotes NF-κB phosphorylation via interaction with and activation of AKT1 during *H. pylori* infection. (A) AGS cells expressing PPFIA4-Flag or non-specific control (NC)-Flag were cultured. sg*PPFIA4*-modified or non-specific control sgRNA (sgNC)-modified AGS cells, and *CASK* siRNA (si*CASK*) or non-specific control siRNA (siNC) pre-treated AGS cells were stimulated with *H. pylori* (MOI=100) for 24 h. Proximity ligation assay in AGS cells was performed by using anti-PPFIA4, anti-CASK, anti-AKT1(Thr308) and anti-AKT1(Ser473) Abs. Single Ab only was used as negative controls. Red dots represent close relationship between the 2 proteins. Scale bars: 30 microns. (B) Immunofluorescence showed the PPFIA4/CASK/p-AKT1(Thr308) or PPFIA4/CASK/p-AKT1(Ser473) co-localization in AGS cells expressing

PPFIA4-Flag. Scale bars: 10 microns.

Supplemental Figure 20

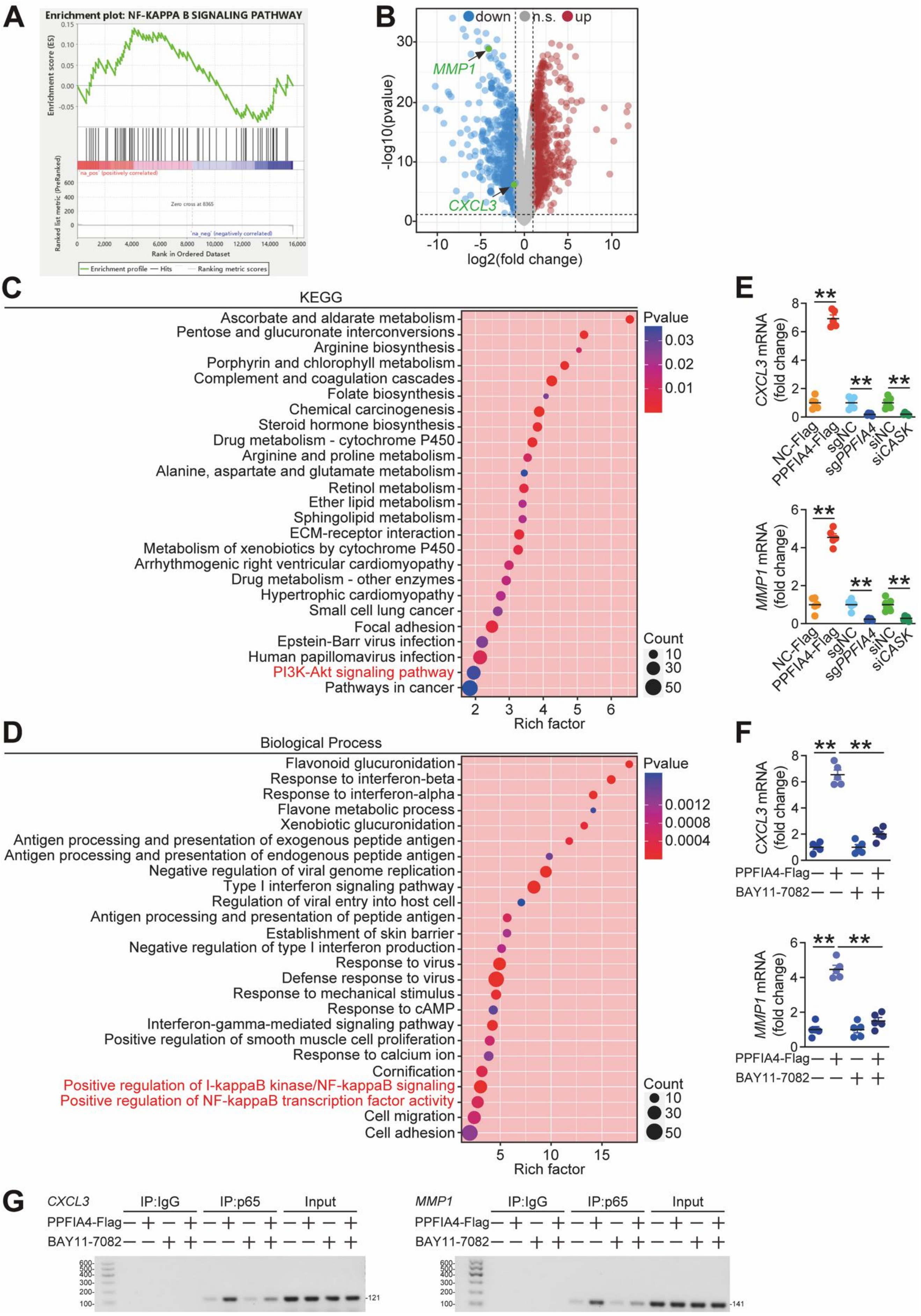

PPFIA4/CASK promotes NF-κB phosphorylation via interaction with and activation of AKT1 during *H. pylori* infection. (A) Compared to AGS cells expressing NC-Flag, gene set enrichment analysis (GSEA) of the significantly changed phosphoproteins in AGS cells expressing PPFIA4-Flag identified the pathway “NF-κB signaling pathway” by the significance levels (P<0.05). (B) Compared to non-specific control sgRNA (sgNC)-modified AGS cells, volcanoplot showed differential expression genes (DEGs) in sg*PPFIA4*-modified AGS cells by RNA-seq (n=3). (C and D) Compared to sgNC-modified AGS cells, the significantly DEGs in sg*PPFIA4*-modified AGS cells were clustered with gene ontology (GO) analysis, and the top 25 GO terms of “Biological Process” (C) as well as the top 25 KEGG pathways (D) were shown. (E) AGS cells expressing PPFIA4-Flag or NC-Flag were cultured. sg*PPFIA4*-modified or sgNC-modified AGS cells, and *CASK* siRNA (si*CASK*) or non-specific control siRNA (siNC) pre-treated AGS cells were stimulated with *H. pylori* (MOI=100) for 24 h. The expressions of *CXCL3* and *MMP1* were analyzed by real-time PCR (n=5). (F) AGS cells expressing PPFIA4-Flag or NC-Flag were pre-treated with or without BAY 11-7082 and cultured. The expressions of *CXCL3* and *MMP1* were analyzed by real-time PCR (n=5). (G) Representative data of ChIP assay in AGS cells expressing PPFIA4-Flag (pre-treated with or without BAY 11-7082), followed by regular PCR with primers designed for NF-κB binding sites of *CXCL3* and *MMP1* promoter region. Data are presented as mean ± SEM. Statistics: unpaired 2-tailed t test (E and F). **P<0.01 for groups connected by horizontal lines.

Supplemental Figure 21

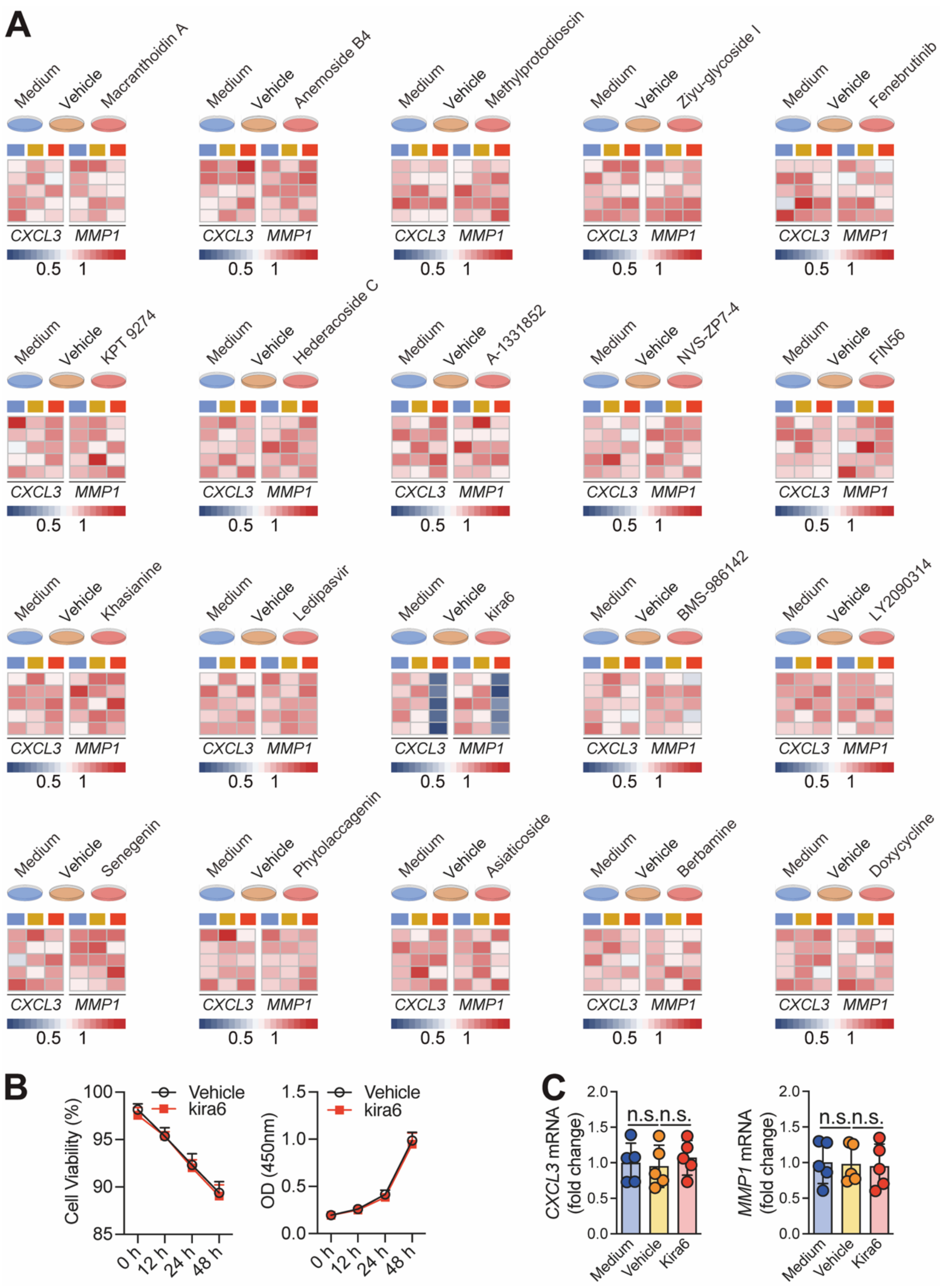

Kira6 is a novel molecular inhibitor of PPFIA4 to ameliorate *H. pylori* persistence and *H. pylori*-induced gastritis. (A) AGS cells expressing PPFIA4-Flag were treated with respective compound (100 nM) or vehicle for 2 h. The expressions of *CXCL3* and *MMP1* were analyzed by real-time PCR (n=5). (B) AGS cells expressing PPFIA4-Flag were treated with kira6 (100 nM) or vehicle for 12, 24 and 48 h. The viability and the proliferation of cells were analyzed by trypan blue and Cell Counting Kit-8 (CCK-8) (n=5). (C) sgNC-modified AGS cells were treated with kira6 (100 nM) or vehicle for 2 h. The expressions of *CXCL3* and *MMP1* were

analyzed by real-time PCR (n=5). Data are presented as mean ± SEM. Statistics: 2-way ANOVA test (B) and unpaired 2-tailed t test (C). n.s. $P>0.05$ for groups connected by horizontal lines.

Supplemental Figure 22

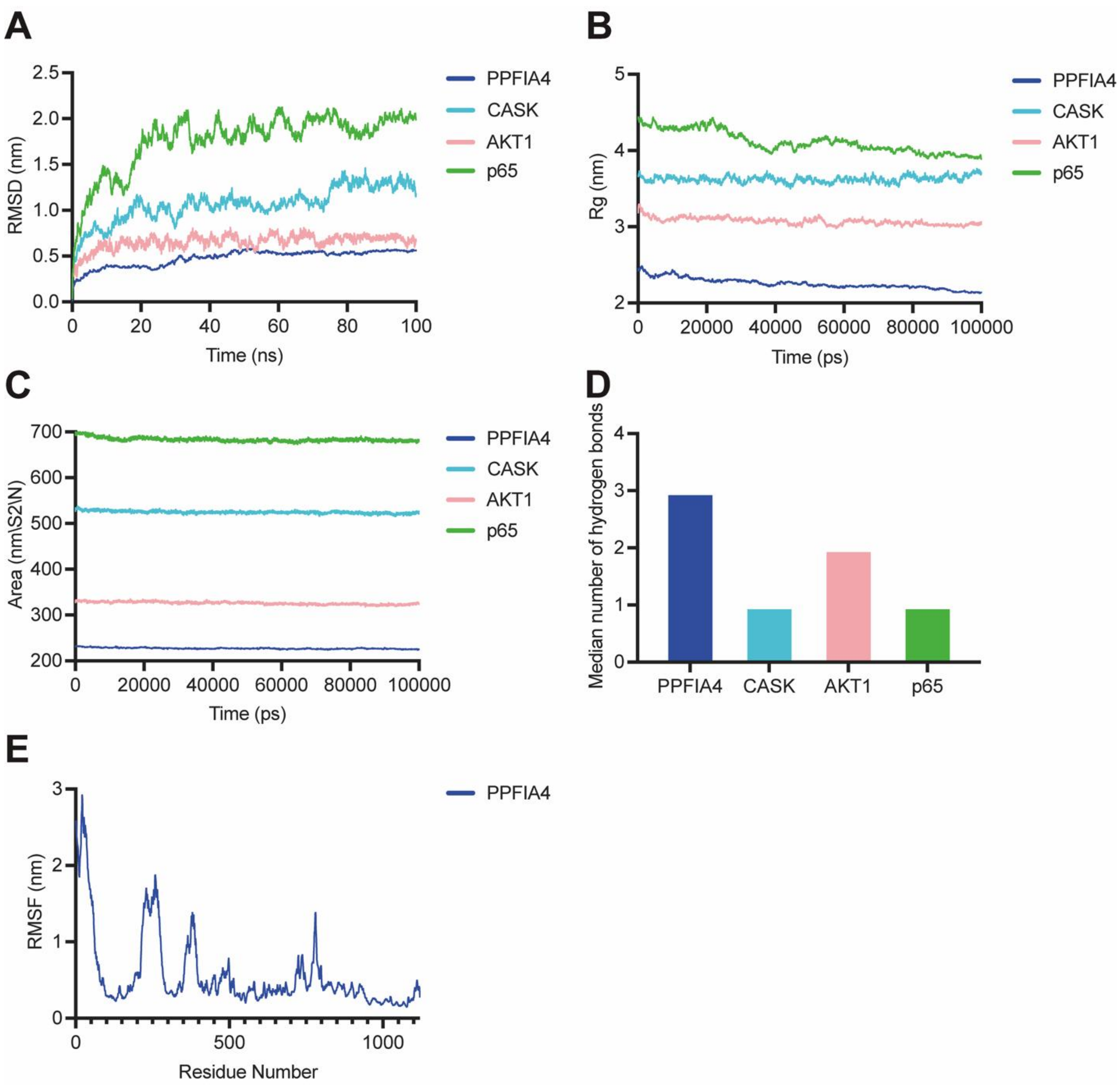

Kira6 is a novel molecular inhibitor of PPFIA4 to ameliorate *H. pylori* persistence and *H. pylori*-induced gastritis. Molecular dynamics simulation of kira6 in complex with PPFIA4, CASK, AKT1 and p65 was analyzed using the root-mean-square deviation (RMSD) (A), the radius of gyration (Rg) (B), the solvent-accessible surface area (SASA) (C), the hydrogen bonds (D) and the root-mean-square fluctuation (RMSF) (E).

Supplemental Figure 23

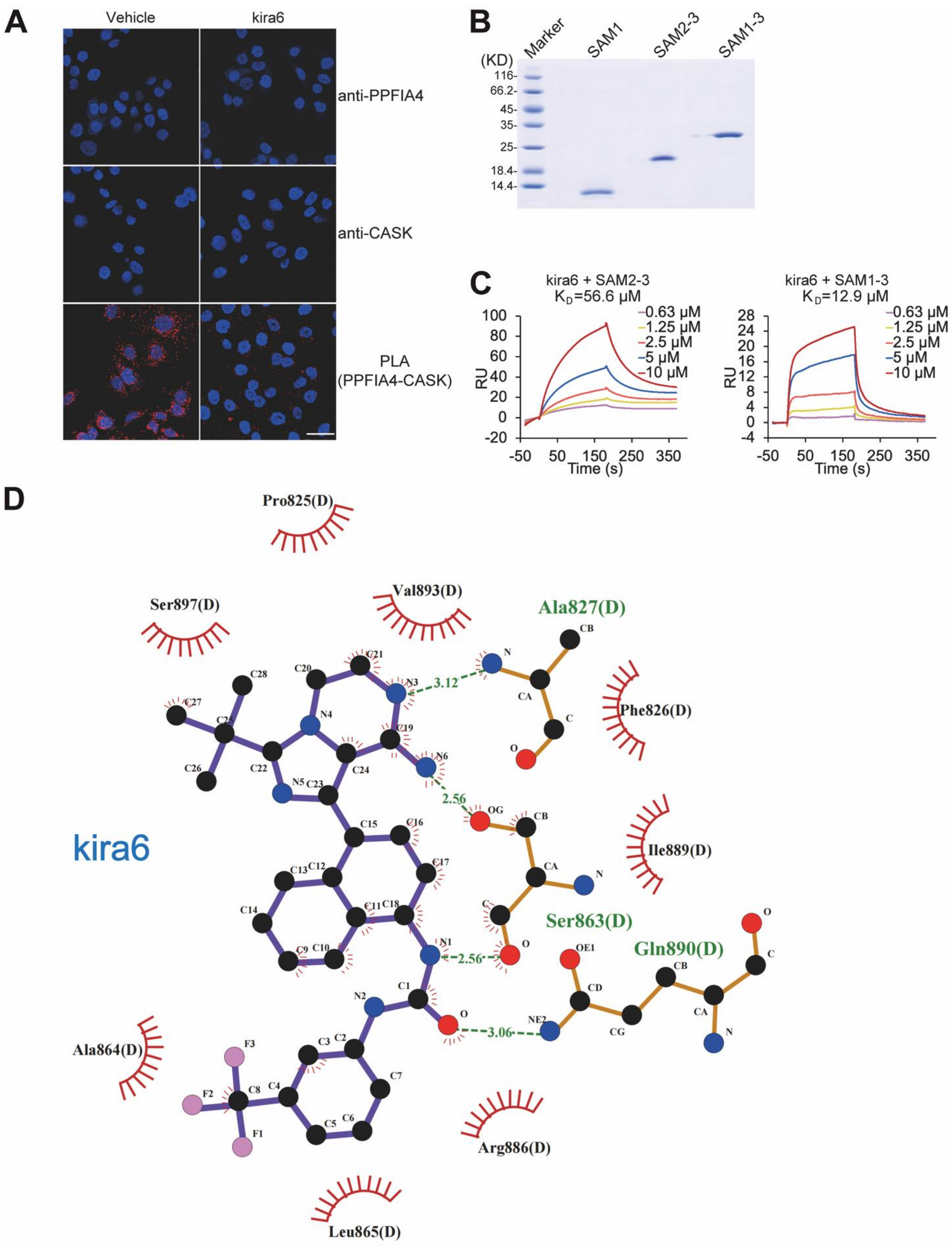

Kira6 is a novel molecular inhibitor of PPFIA4 to ameliorate *H. pylori* persistence and *H. pylori*-induced gastritis. (A) AGS cells expressing PPFIA4-Flag were treated with kira6 (100 nM) or vehicle for 2 h. Proximity ligation assay in AGS cells was performed by using anti-PPFIA4 and anti-CASK Abs. Single Ab only was used as negative controls. Red dots represent close relationship between the 2 proteins. Scale bars: 30 microns. (B)

The proteins of the domains of SAM1, SAM2-3 and SAM1-3 of PPFIA4 were shown. (C) SPR experiments measuring the binding ability of the domains of SAM2-3 and SAM1-3 of PPFIA4 with kira6. Colored curves represent kira6 concentration with serial dilutions of 1:2. RU, response unit. (D) The schematic illustration for the binding between PPFIA4 and kira6.

Supplemental Figure 24

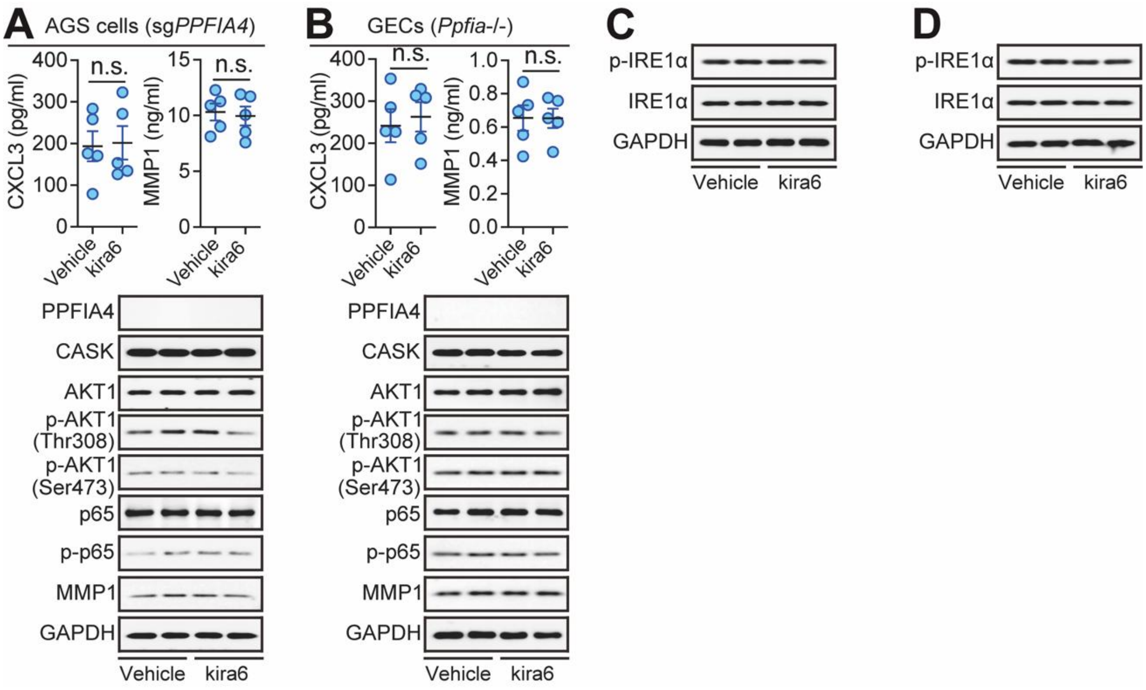

Kira6 is a novel molecular inhibitor of PPFIA4 to ameliorate *H. pylori* persistence and *H. pylori*-induced gastritis. (A and B) sg*PPFIA4*-modified AGS cells (A) and primary gastric epithelial cells (GECs) from uninfected *Ppfia4*$^{-/-}$ mice (B) were treated with kira6 (100 nM) or vehicle for 2 h, then were stimulated with *H. pylori* (MOI=100) for 24 h. PPFIA4, CASK, AKT1, p-AKT1(Thr308), p-AKT1(Ser473), p65, p-p65 and MMP1 proteins were analyzed by western blot. CXCL3 and MMP1 productions were analyzed by ELISA (n=5). (C and D) sgNC-modified AGS cells (C) and primary GECs from uninfected WT mice (D) were treated with kira6 (100 nM) or vehicle for 2 h, then were stimulated with *H. pylori* (MOI=100) for 24 h. IRE1α and p-IRE1α proteins were analyzed by western blot. Data are presented as mean ± SEM. Statistics: unpaired 2-tailed t test (A and B). n.s. $P > 0.05$ for groups connected by horizontal lines.

Supplemental Figure 25

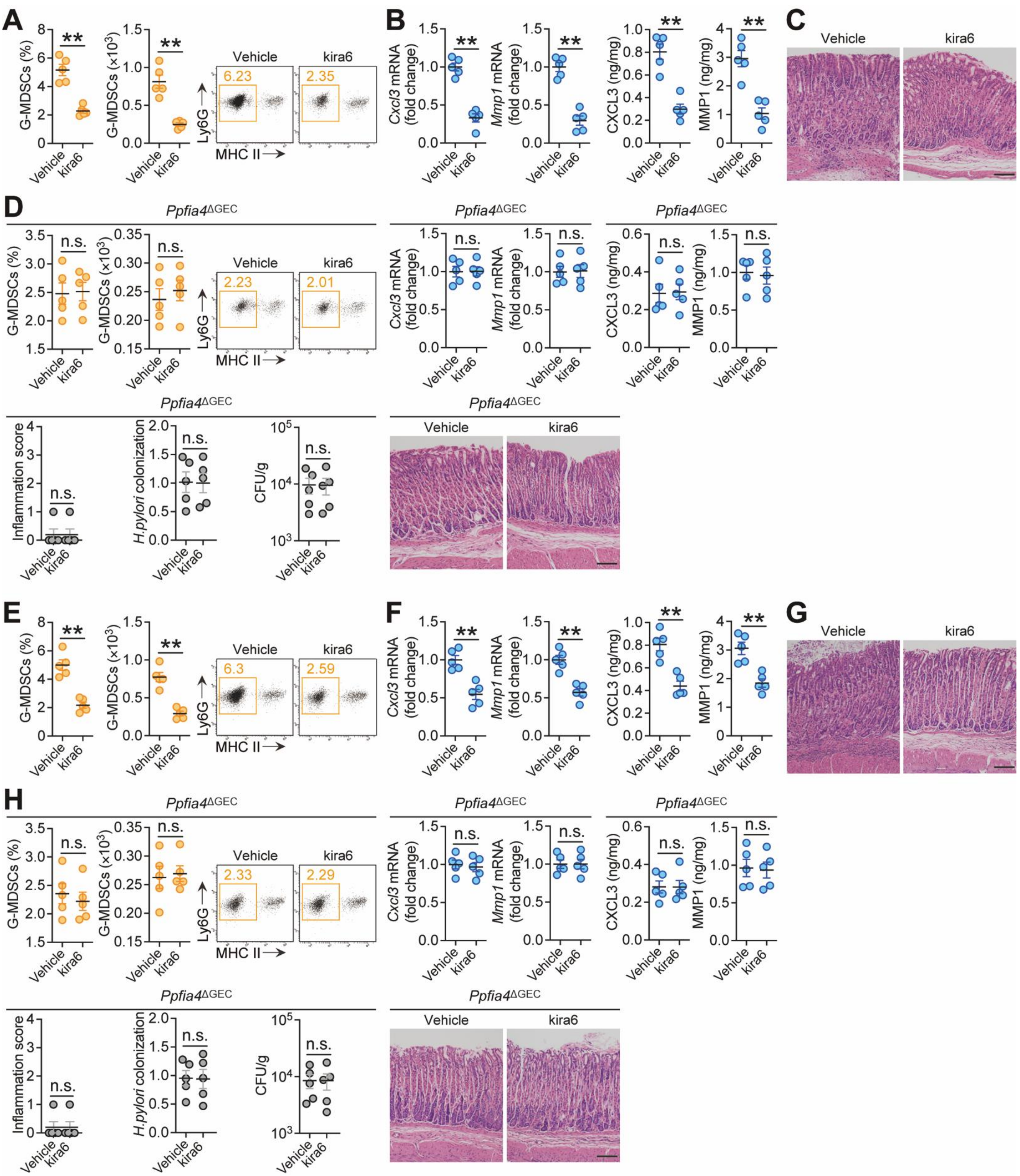

Kira6 is a novel molecular inhibitor of PPFIA4 to ameliorate *H. pylori* persistence and *H. pylori*-induced gastritis. (A and B) The levels of G-MDSCs (A), *Cxcl3*, *Mmp1*, CXCL3 and MMP1 (B) in gastric mucosa of *H. pylori*-infected *Ppfia4*$^{flox/flox}$ littermates injected with kira6 or vehicle 15 weeks p.i. were shown (n=5). (C) Representative H&E staining images showing inflammation in gastric mucosa of *H. pylori*-infected *Ppfia4*$^{flox/flox}$

littermates injected with kira6 or vehicle 15 weeks p.i.. Scale bars: 100 microns. (D) The levels of G-MDSCs, *Cxcl3*, *Mmp1*, CXCL3, MMP1, bacteria colonization and inflammation in gastric mucosa of *H. pylori*-infected *Ppfia4*$^{\Delta GEC}$ mice injected with kira6 or vehicle 15 weeks p.i. were shown (n=5). Representative H&E staining images showing inflammation in gastric mucosa of *H. pylori*-infected *Ppfia4*$^{\Delta GEC}$ mice injected with kira6 or vehicle 15 weeks p.i.. Scale bars: 100 microns. The bacteria colonization is shown as log10(the number of bacterial genomes per nanogram of host genomic DNA) by measuring *H. pylori*-specific 16s rDNA or as CFU per gram of stomach tissue by bacterial reisolation and quantitative culture. (E and F) The levels of G-MDSCs (E), *Cxcl3*, *Mmp1*, CXCL3 and MMP1 (F) in gastric mucosa of *H. pylori*-infected *Ppfia4*$^{flox/flox}$ littermates injected with kira6 or vehicle 18 weeks p.i. were shown (n=5). (G) Representative H&E staining images showing inflammation in gastric mucosa of *H. pylori*-infected *Ppfia4*$^{flox/flox}$ littermates injected with kira6 or vehicle 18 weeks p.i.. Scale bars: 100 microns. (H) The levels of G-MDSCs, *Cxcl3*, *Mmp1*, CXCL3, MMP1, bacteria colonization and inflammation in gastric mucosa of *H. pylori*-infected *Ppfia4*$^{\Delta GEC}$ mice injected with kira6 or vehicle 18 weeks p.i. were shown (n=5). Representative H&E staining images showing inflammation in gastric mucosa of *H. pylori*-infected *Ppfia4*$^{\Delta GEC}$ mice injected with kira6 or vehicle 18 weeks p.i.. Scale bars: 100 microns. The bacteria colonization is shown as log10(the number of bacterial genomes per nanogram of host genomic DNA) by measuring *H. pylori*-specific 16s rDNA or as CFU per gram of stomach tissue by bacterial reisolation and quantitative culture. Data are presented as mean ± SEM. Statistics: The P Value of inflammation score represent Mann-Whitney U test (D and H), all other P values represent unpaired 2-tailed t test (A, B, D-F and H). **P*<0.05, ***P*<0.01, n.s. *P*>0.05 for groups connected by horizontal lines.

Supplemental Figure 26

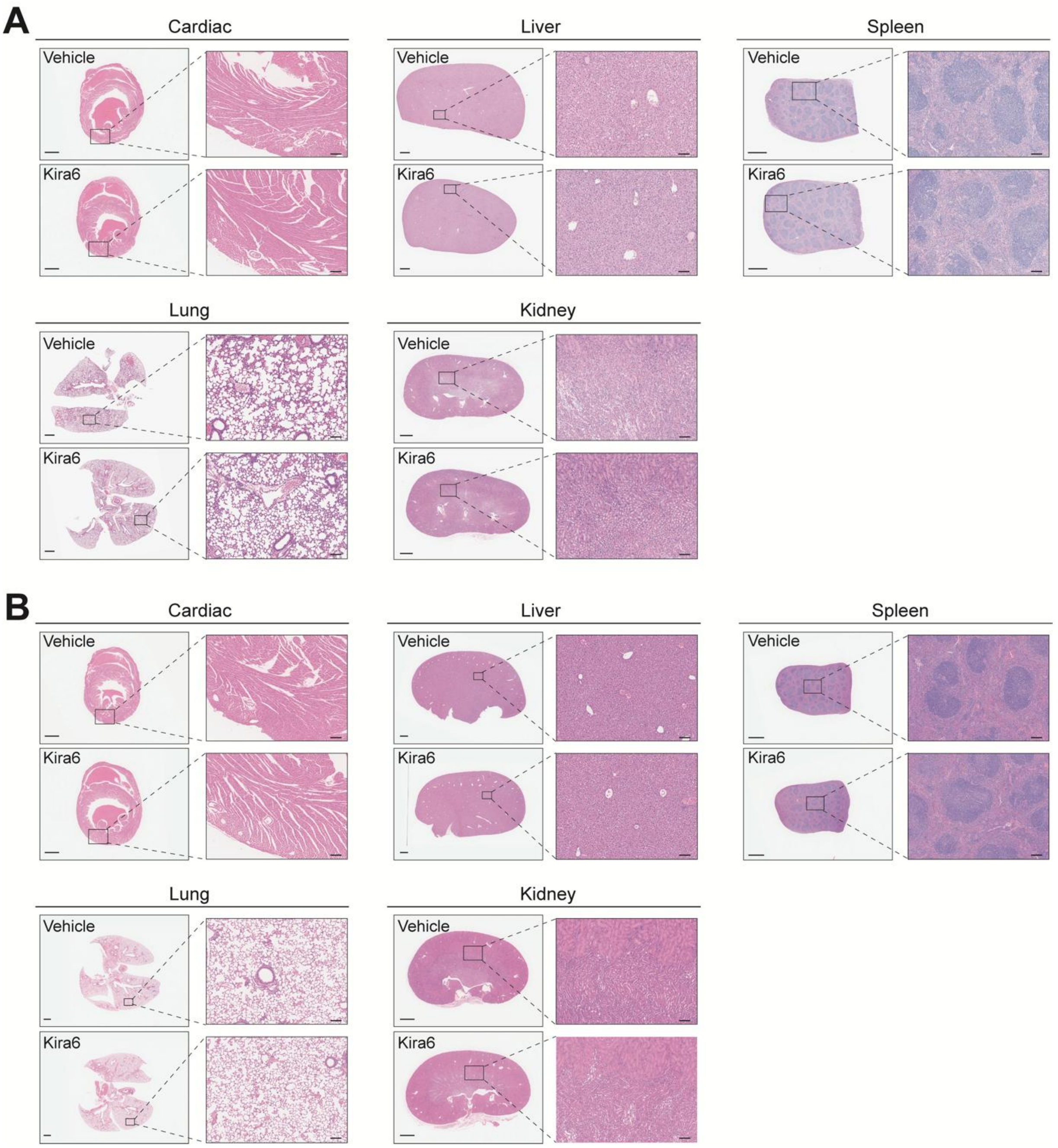

Kira6 is a novel molecular inhibitor of PPFIA4 to ameliorate *H. pylori* persistence and *H. pylori*-induced gastritis. (A) Representative H&E staining images showing tissue histology of *Ppfia4*$^{\Delta GEC}$ mice and *Ppfia4*$^{flox/flox}$ littermates injected with kira6 or vehicle 15 weeks p.i.. Scale bars: 1000 microns (left panel in each tissue images) or 100 microns (right panel in each tissue images). (B) Representative H&E staining images showing tissue histology of *Ppfia4*$^{\Delta GEC}$ mice and *Ppfia4*$^{flox/flox}$ littermates injected with kira6 or vehicle 18 weeks p.i.. Scale bars: 1000 microns (left panel in each tissue images) or 100 microns (right panel in each tissue images).

Supplemental Figure 27

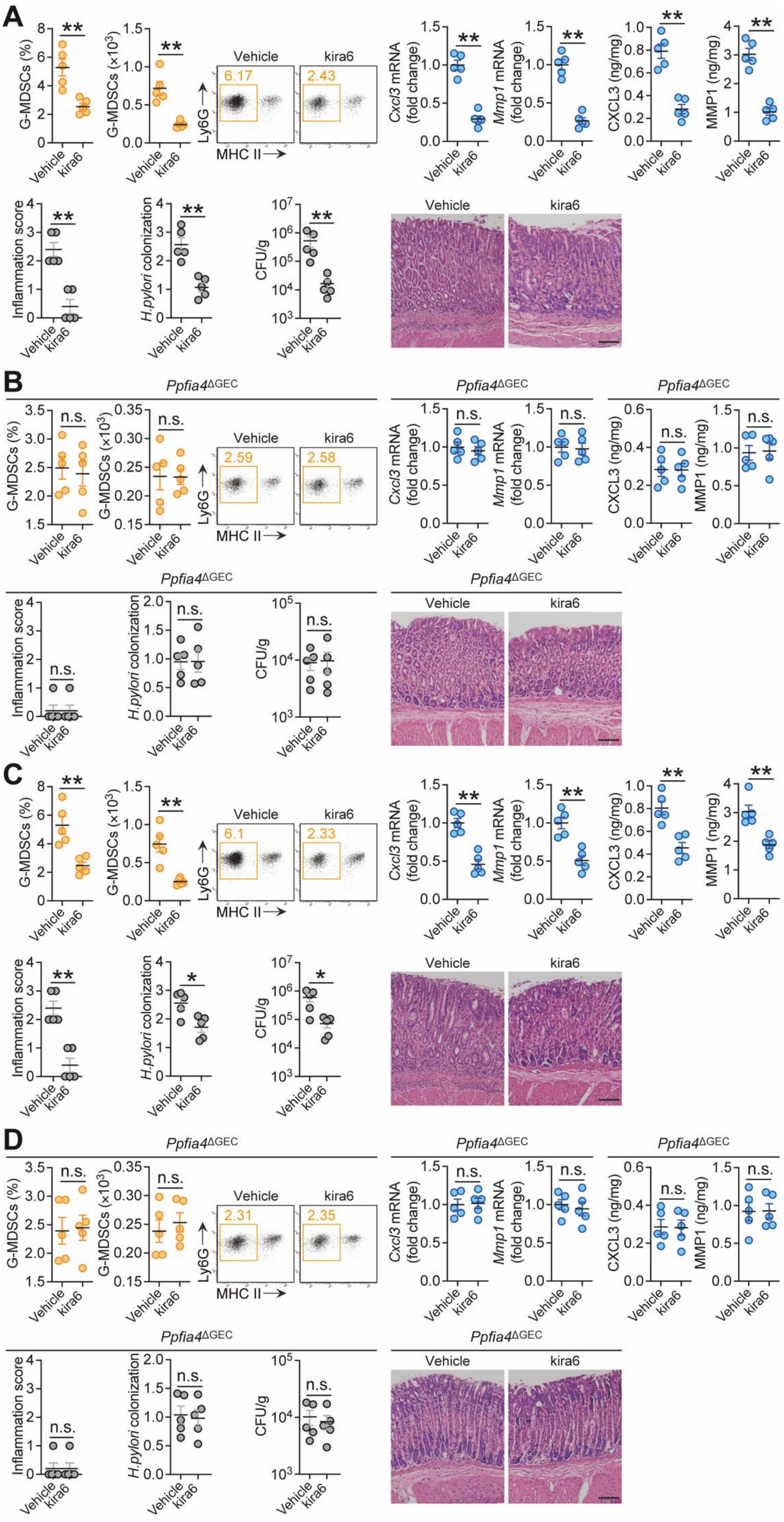

Kira6 is a novel molecular inhibitor of PPFIA4 to ameliorate *H. pylori* persistence and *H. pylori*-induced gastritis. The levels of G-MDSCs, *Cxcl3*, *Mmp1*, CXCL3, MMP1, bacteria colonization and inflammation in gastric mucosa of antibiotic-resistant *H. pylori*-infected *Ppfia4*$^{flox/flox}$ littermates injected with kira6 or vehicle 15 weeks p.i. (A), in gastric mucosa of antibiotic-resistant *H. pylori*-infected *Ppfia4*$^{\Delta GEC}$ mice injected with kira6 or vehicle 15 weeks p.i. (B), in gastric mucosa of antibiotic-resistant *H. pylori*-infected *Ppfia4*$^{flox/flox}$ littermates injected with kira6 or vehicle 18 weeks p.i. (C), or in gastric mucosa of antibiotic-resistant *H. pylori*-infected *Ppfia4*$^{\Delta GEC}$ mice injected with kira6 or vehicle 18 weeks p.i. (D) were shown (n=5). Representative H&E staining images showing inflammation in gastric mucosa. Scale bars: 100 microns. The bacteria colonization is shown as log10(the number of bacterial genomes per nanogram of host genomic DNA) by measuring *H. pylori*-specific 16s rDNA or as CFU per gram of stomach tissue by bacterial reisolation and quantitative culture. Data are presented as mean ± SEM. Statistics: The P Value of inflammation score represent Mann-Whitney U test (A-D), all other P values represent unpaired 2-tailed t test (A-D). **P*<0.05, ***P*<0.01, n.s. *P*>0.05 for groups connected by horizontal lines.

1 Supplemental Figure 28

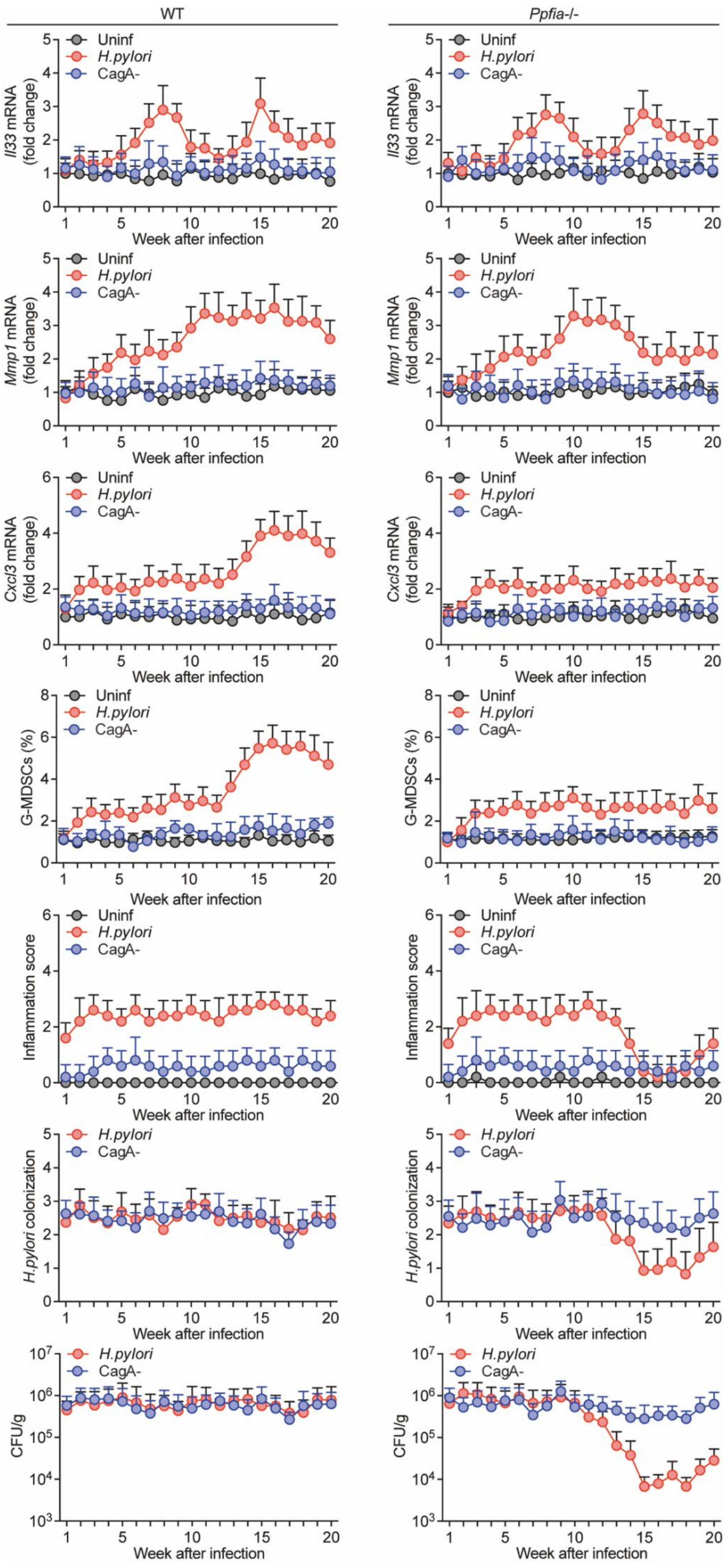

2

Dynamic changes of *Il33* expression, *Mmp1* expression, *Cxcl3* expression, G-MDSC levels, histological scores of inflammation and bacteria colonization in gastric mucosa of *H. pylori*-infected, CagA- *H. pylori*-infected and uninfected WT mice and *Ppfia4*$^{-/-}$ mice (n=5 per group per time point). The bacteria colonization is shown as log10(the number of bacterial genomes per nanogram of host genomic DNA) by measuring *H. pylori*-specific 16s rDNA or as CFU per gram of stomach tissue by bacterial reisolation and quantitative culture. Only the temporal trends in the three groups are visualized, statistical significance was not evaluated.

Supplemental Figure 29

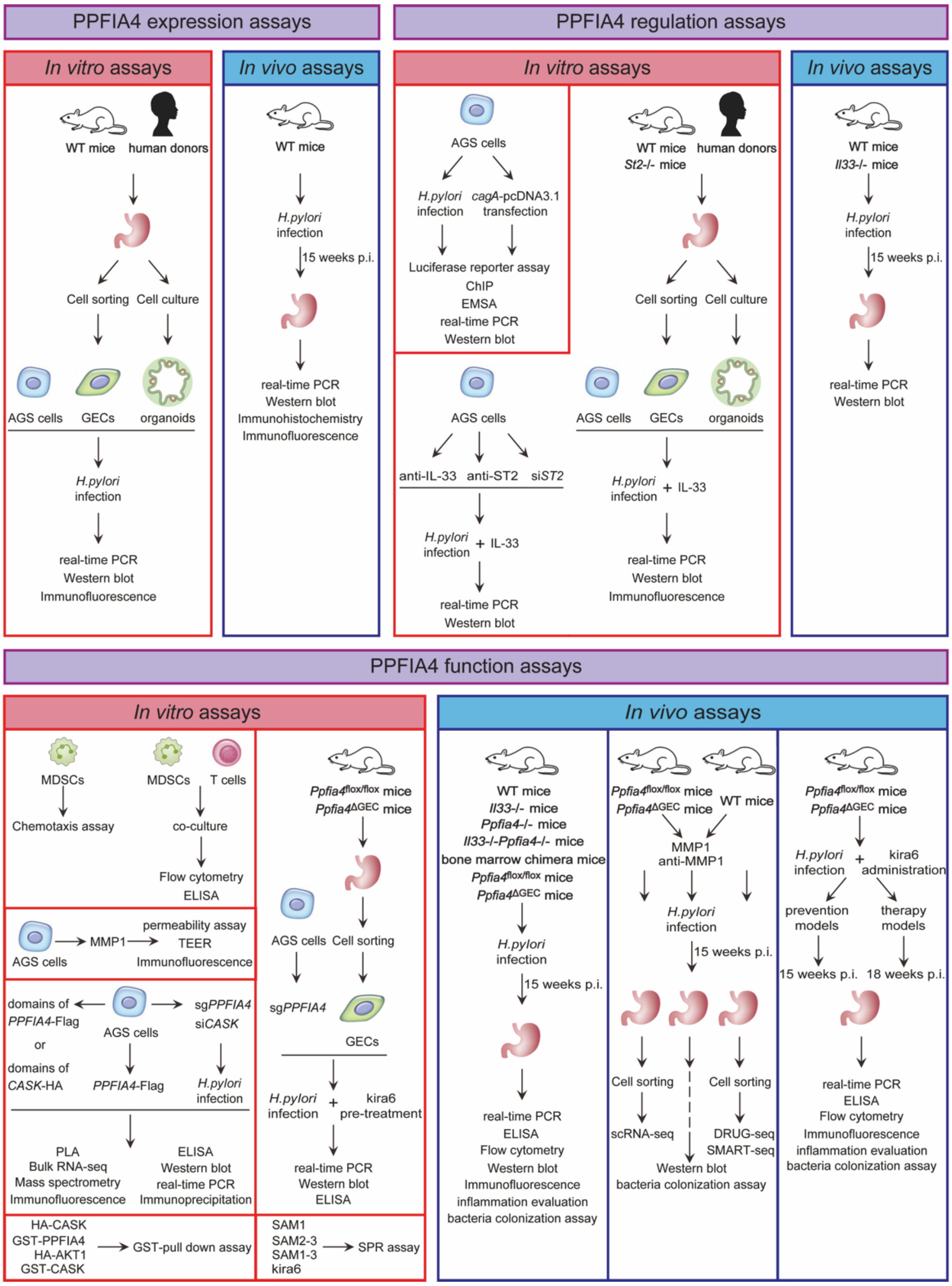

A schematic summary of experimental workflows.

Supplemental Table. 1. Clinical characteristics of patients

Variables	*H. pylori*-infected	Uninfected	Gastric ulcer
Age (median, range)	(50 year, 16-86 years)	(51 year, 23-71 years)	(45 year, 28-66 years)
Sex (male/female)	72/59	31/19	20/12

Exclusion criteria were: previous treatment for *H. pylori* infection, use of inhibitors of acid secretion and/or antibiotics during the 2 months before the study, use of anticoagulant drugs in the last week, gastrointestinal malignancy, severe concomitant cardiovascular, respiratory or endocrine diseases, clinically significant renal or hepatic disease, haematological disorders, previous gastro-oesophageal surgery, history of allergy to any of the drug used in the study, pregnancy or lactation, alcohol abuse, drug addiction, severe neurological or psychiatric disorders, and long-term use of corticosteroids or anti-inflammatory drugs.

Supplemental Table. 2. Antibodies and other reagents

Antibodies and reagents	Manufacturers
Antibodies for flow cytometry	
anti-mouse ST2-APC-Cy7(567901)	BD Pharmingen
anti-mouse CD45-BV421(563890)	BD Pharmingen
anti-mouse CD11b-PE-Cy7(552850)	BD Pharmingen
anti-mouse CD64-APC(139306)	Biolegend
anti-mouse CD19-PE(557399)	BD Pharmingen
anti-mouse CD90.2-PE(553005)	BD Pharmingen
anti-mouse CD49b-PE(108908)	Biolegend
anti-mouse Ly6G-PE(551461)	BD Pharmingen
anti-mouse SiglecF-PE(552126)	BD Pharmingen
anti-mouse MHC II-FITC(107605)	Biolegend
anti-mouse Ly6C-PerCP-Cy5.5(551461)	BD Pharmingen
anti-mouse CD11c-APC-Cy7(117324)	Biolegend
anti-mouse CXCR2-APC-Cy7(149314)	Biolegend
anti-mouse CD4-PE(116005)	Biolegend
anti-mouse IFN-γ-APC(505810)	Biolegend
anti-human ST2-PE(569315)	BD Pharmingen
anti-human CD45-PE-Cy7(368532)	Biolegend
anti-human CD11b-PerCP-Cy5.5(301328)	Biolegend
anti-human CD15-APC-Cy7(323048)	Biolegend
anti-human CD66b-FITC(305104)	Biolegend
anti-human HLA-DR-Alexa Fluor® 700(560743)	BD Pharmingen
anti-human CXCR2-BV605(744197)	BD Pharmingen
Antibodies for immunohistochemical staining	
rabbit anti-human/mouse PPFIA4(NBP2-31560, 1:500)	NOVUS
horseradish peroxidase anti-rabbit IgG(ZB-2301)	Zhongshan Biotechnology
Antibodies for immunofluorescence	
rabbit anti-human/mouse PPFIA4(NBP2-31560, 1:500)	NOVUS
mouse anti-human CASK(MA5-36120, 1:100)	Thermo Fisher Scientific
rabbit anti-human AKT1(75692S, 1:500)	Cell Signaling Technology
rabbit anti-human p-AKT1(Thr308)(710122, 1:500)	Thermo Fisher Scientific
rabbit anti-human p-AKT1(Ser473)(9018S, 1:500)	Cell Signaling Technology
rabbit anti-human EpCAM(ab71916, 1:500)	Abcam
rabbit anti-human E-cadherin(3195S, 1:1000)	Cell Signaling Technology
rabbit anti-human ZO-1(ab96587, 1:500)	Abcam
rabbit anti-human MMP1(ab137332, 1:500)	Abcam
mouse anti-human/mouse GIF(sc-514524, 1:500)	Santa Cruz Biotechnology

mouse anti-human/mouse ATP4B(sc-374094, 1:500)	Santa Cruz Biotechnology
mouse anti-human/mouse MUC5AC(MA5-12178, 1:500)	Thermo Fisher Scientific
mouse anti-human/mouse ChgA(sc-393941, 1:500)	Santa Cruz Biotechnology
rabbit anti-human/mouse Dclk1(ab31704, 1:200)	Abcam
rabbit anti-mouse CXCL3(PA5-103136, 1:100)	Thermo Fisher Scientific
rabbit anti-mouse CASK(PA1544, 1:2000)	Thermo Fisher Scientific
rabbit anti-human/mouse CD3(ab5690, 1:500)	Abcam
mouse anti-human/mouse CD19(sc-373897, 1:1000)	Santa Cruz Biotechnology
mouse anti-human/mouse NK1.1(MA1-70100, 1:250)	Thermo Fisher Scientific
mouse anti-human/mouse CD68(sc-20060, 1:200)	Santa Cruz Biotechnology
rabbit anti-mouse Ly6G(6O10Y1, 1:200)	Thermo Fisher Scientific
rabbit anti-mouse MHC II(sc-32247, 1:200)	Santa Cruz Biotechnology
goat anti-rabbit antibody, AF Plus 555(A32732, 1:1000)	Thermo Fisher Scientific
goat anti-rabbit antibody, AF Plus 488(A32731, 1:1000)	Thermo Fisher Scientific
goat anti-mouse antibody, AF Plus 488(A32723, 1:1000)	Thermo Fisher Scientific
goat anti-mouse antibody, AF Plus 555 (A-21424, 1:1000)	Thermo Fisher Scientific
goat anti-rabbit antibody, AF Plus 647(A32733, 1:1000)	Thermo Fisher Scientific
Antibodies for neutralizing and blocking	
anti-human IL-33 (Goat IgG)(AF3625)	R&D Systems
anti-human ST2 (Goat IgG)(AF523)	R&D Systems
Normal Goat IgG Control(AB-108-C)	R&D Systems
anti-human CXCL3(Mouse IgG1)(MAB276)	R&D Systems
Mouse IgG1 Isotype Control(MAB002)	R&D Systems
anti-human CXCR2(Mouse IgG2a)(MAB331)	R&D Systems
Mouse IgG2a Isotype Control(MAB003)	R&D Systems
anti-mouse CXCL3(Sheep IgG)(AF5568)	R&D Systems
Normal Sheep IgG Control(5-001-A)	R&D Systems
anti-mouse CXCR2(Rat IgG2a)(MAB2164)	R&D Systems
Rat IgG2a Isotype Control(MAB006)	R&D Systems
anti-mouse MMP1 (Mouse IgG1)(NBP2-22123)	NOVUS
Antibodies for western blot	
rabbit anti-human/mouse PPFIA4(NBP2-31560, 1:1000)	NOVUS
rabbit anti-human/mouse PPFIA4(PA5-62787, 1:1000)	Thermo Fisher Scientific
rabbit anti-human/mouse PPFIA4(HPA053419, 1:1000)	Sigma-Aldrich
rabbit anti-human/mouse c-Jun(9165S, 1:1000)	Cell Signaling Technology
rabbit anti-human/mouse p-c-Jun(3270S, 1:1000)	Cell Signaling Technology
mouse anti-human CASK(MA5-36120, 1:2000)	Thermo Fisher Scientific
rabbit anti-human/mouse AKT1(75692S, 1:1000)	Cell Signaling Technology
rabbit anti-human/mouse p-AKT1(Thr308)(710122, 1:1000)	Thermo Fisher Scientific

rabbit anti-human/mouse p-AKT1(Ser473)(9018S, 1:1000)	Cell Signaling Technology
rabbit anti-human/mouse p65(8242S, 1:1000)	Cell Signaling Technology
rabbit anti-human/mouse p-p65(3033S, 1:1000)	Cell Signaling Technology
rabbit anti-human/mouse E-cadherin(3195S, 1:1000)	Cell Signaling Technology
rabbit anti-human/mouse ZO-1(ab96587, 1:1000)	Abcam
rabbit anti-human/mouse MMP1(ab137332, 1:1000)	Abcam
rabbit anti-human ST2(ab194113, 1:1000)	Abcam
rabbit anti-HA(3724S, 1:1000)	Cell Signaling Technology
rabbit anti-Flag(14793S, 1:1000)	Cell Signaling Technology
rabbit anti-mouse CASK(PA1544, 1:2000)	Thermo Fisher Scientific
rabbit anti-IRE1α(3294S, 1:1000)	Cell Signaling Technology
rabbit anti-p-IRE1α(ab308387, 1:1000)	Abcam
rabbit anti-human/mouse GAPDH(5174S)	Cell Signaling Technology
Antibodies for Chromatin Immunoprecipitation	
rabbit anti-human c-Jun(9165S, 1:50)	Cell Signaling Technology
rabbit anti-human p65(8242S, 1:100)	Cell Signaling Technology
rabbit IgG Isotype Control(ab172730)	Abcam
Antibodies for Co-Immunoprecipitation	
rabbit anti-Flag(Rabbit IgG)(14793S, 1:50)	Cell Signaling Technology
rabbit anti-HA(Rabbit IgG)(3724S, 1:50)	Cell Signaling Technology
rabbit anti-human CASK(Rabbit IgG)(8968S, 1:50)	Cell Signaling Technology
rabbit IgG Isotype Control(2729S)	Cell Signaling Technology
Antibodies for GST-pull down assay	
rabbit anti-GST(Rabbit IgG)(2622S, 1:1000)	Cell Signaling Technology
rabbit anti-HA(Rabbit IgG)(3724S, 1:1000)	Cell Signaling Technology
ELISA kits	
human CXCL3	MultiSciences
human MMP1	Thermo Fisher Scientific
human IL-33	Thermo Fisher Scientific
mouse CXCL3	Abcam
mouse MMP1	NOVUS
mouse IFN-γ	Thermo Fisher Scientific
Reagents for human gastric organoid cultures	
Human Gastric Epithelial Organoid Kit	Biogenous
Organoid Recovery Solution	Biogenous
Anti-Adherence Rinsing Solution	Biogenous
Organoid Dissociation Solution	Biogenous
Organoid Cryopreservation Medium (Serum Free)	Biogenous
Advanced DMEM/F-12(12634010)	Thermo Fisher Scientific

Matrigel® Basement Membrane Matrix(356234)	Corning
DPBS	Solarbio
Reagents for mouse gastric organoid cultures	
Murine Wnt3a(315-20)	PeproTech
Murine Noggin(250-38)	PeproTech
Murine R-Spindin-1(315-32)	PeproTech
Murine EGF(315-09)	PeproTech
Murine FGF-10(450-61)	PeproTech
Murine Gastrin I(1003377)	PeproTech
N-2 Supplement(17502048)	Thermo Fisher Scientific
Advanced DMEM/F12(12634010)	Thermo Fisher Scientific
GlutaMAX Supplement(35050061)	GIBCO
Y-27632 dihydrochloride(Y0503)	Sigma-Aldrich
Matrigel® Basement Membrane Matrix(356234)	Corning
Reagents for signaling pathway inhibition	
AP1 inhibitor T-5224	Merck Millipore
NF-κB inhibitor BAY 11-7082	Merck Millipore
CagA EPIYA motif phosphorylation inhibitor PP2	Merck Millipore
Reagents for luciferase reporter assay and ChIP	
Dual-Luciferase Reporter assay Kit	Promega
Endo-free Plasmid Mini Kit	Omega
Pierce Magnetic ChIP Kit	Thermo Fisher Scientifific
16% Formaldehyde, Methanol-Free	Cell Signaling Technology
Antibodies and Reagents for Proximity Ligation Assay	
mouse anti-human PPFIA4(WH0008497M10, 1:1000)	Merck Millipore
rabbit anti-human PPFIA4(NBP2-31560, 1:500)	NOVUS
mouse anti-human CASK(MA5-36120, 1:100)	Thermo Fisher Scientific
rabbit anti-human CASK(8968S, 1:2000)	Cell Signaling Technology
rabbit anti-HA(3724S, 1:800)	Cell Signaling Technology
rabbit anti-Flag(14793S, 1:400)	Cell Signaling Technology
rabbit anti-human AKT1(75692S, 1:500)	Cell Signaling Technology
rabbit anti-human p-AKT1(Thr308)(710122, 1:500)	Thermo Fisher Scientific
rabbit anti-human p-AKT1(Ser473)(9018S, 1:500)	Cell Signaling Technology
Donkey anti-rabbit IgG (H+L)(DUO92002-100RXN)	Sigma-Aldrich
Donkey anti-mouse IgG (H+L)(DUO92004-100RXN)	Sigma-Aldrich
Duolink® In Situ Detection Reagents Red(DUO92008-100RXN)	Sigma-Aldrich
Duolink® In Situ Wash Buffers(DUO82049-4L)	Sigma-Aldrich
Duolink® In Situ Mounting Medium with DAPI(DUO82040-5ML)	Sigma-Aldrich
μ-Slide Angiogenesis(81506)	ibidi

Bovine Serum Albumin(BSA)(15260037)	Thermo Fisher Scientific
Human CD326 microbeads	MiltenyiBiotec
Mouse CD326 microbeads	MiltenyiBiotec
CD4 microbeads	StemCell Technologies
CD45 microbeads	StemCell Technologies
Purified anti-mouse CD3 antibodies	Biolegend
Purified anti-mouse CD28 antibodies	Biolegend
Leukocyte Activation Cocktail, with BD GolgiPlug™	BD Pharmingen
Perm/Wash solution	BD Pharmingen
8-μm pore size Transwells	Corning
5-μm pore size Transwells	Corning
3-μm pore size Transwells	Corning
0.4-μm pore size Transwells	Corning
FITC-dextran	Invitrogen
Collagenase I	Sigma-Aldrich
Collagenase II	Sigma-Aldrich
Collagenase IV	Sigma-Aldrich
DNase I	Sigma-Aldrich
DMSO	Sigma-Aldrich
Trypan blue	Beyotime Biotechnology
Cell counting kit-8	Solarbio
Complete, EDTA free, EASYpack	Roche
PhosStop EASYpack	Roche
Super ECL Plus Western Blotting Substrate	Bioground
Protein Extraction Reagent	Pierce
Fetal bovine serum (FBS)	Hyclone
Penicillin/Streptomycin	Gibco
RPMI-1640	Hyclone
DMEM/F12 (1:1)	Hyclone
Red Cell Lysis Buffer	TIANGEN
BD FACS™ Lysing Solution	BD Pharmingen
TRIzol reagent	TaKaRa
Lipofectamine™ 2000 Transfection Reagent	Invitrogen
QIAamp DNA Mini Kit	QIAGEN
PrimeScript^{TM} RT reagent Kit	TaKaRa
TB Green® Premix Ex Taq™ II	TaKaRa
Premix Ex Taq™	TaKaRa
RNeasy Micro Kits	Qiagen
Recombinant human IL-33	R&D Systems

Recombinant human MMP1	R&D Systems
Recombinant human CXCL3	R&D Systems
Recombinant mouse IL-33	R&D Systems
Recombinant mouse MMP1	Chemical Book
Recombinant mouse CXCL3	R&D Systems
All other recombinant human/mouse cytokines and chemokines	PeproTech

APC-Cy7, allophycocyanin-cyanin 7; V450, BD Horizon V450; PE-Cy7, phycoerythrin-cyanin 7; APC, allophycocyanin; PE, phycoerythrin; FITC, fluorescein isothiocyanate; PerCP-Cy5.5, peridinchlorophyl protein-cyanin 5.5; BV605, Brilliant Violet™ 605; IL, interleukin.

Supplemental Table. 3. Primer and probe sequences for real-time PCR analysis

Gene	Primer or probe	Sequence 5′→3′
H. pylori 16s rDNA	forward	TTTGTTAGAGAAGATAATGACGGTATCTAAC
	reverse	CATAGGATTTCACACCTGACTGACTATC
	probe	CGTGCCAGCAGCCGCGGT
Mouse *β2-microglobulin*	forward	CCTGCAGAGTTAAGCATGCCAG
	reverse	TGCTTGATCACATGTCTCGATCC
	probe	TGGCCGAGCCCAAGACCGTCTAC
H. pylori cagA	forward	GAGTCATAATGGCATAGAACCTGAA
	reverse	TTGTGCAAGAAATTCCATGAAA
Mouse *Sry*	forward	TGGGACTGGTGACAATTGTC
	reverse	GAGTACAGGTGTGCAGCTCT
Mouse *β-actin*	forward	AGTGTGACGTTGACATCCGT
	reverse	GCAGCTCAGTAACAGTCCGC
Mouse *Ppfia4*	forward	GACATGAATCACGAGTGGATTG
	reverse	AGGCACTCCATGAAGTAACTAC
Mouse *Ccl1*	forward	CTTCCCCTGAAGTTTATCCAGT
	reverse	TCTACCTTTGTTCAGCCTGAAT
Mouse *Ccl2*	forward	TCACCTGCTGCTACTCATTCA
	reverse	CACTGTCACACTGGTCACTCC
Mouse *Ccl3*	forward	TTCTCTGTACCATGACACTCTGC
	reverse	CGTGGAATCTTCCGGCTGTAG
Mouse *Ccl4*	forward	TGTCTGCCCTCTCTCTCCTCT
	reverse	AGCAAGGACGCTTCTCAGTGA
Mouse *Ccl5*	forward	GCTGCTTTGCCTACCTCTCC
	reverse	TCGAGTGACAAACACGACTGC
Mouse *Ccl6*	forward	CCAAGACTGCCATTTCATTC
	reverse	AAGCAATGACCTTGTTCCCA
Mouse *Ccl7*	forward	ATGGAAGTCTGCGCTGAAG
	reverse	ACATGAGGTCTCCAGAGCTTT
Mouse *Ccl8*	forward	ACGCTAGCCTTCACTCCAAAA
	reverse	TTCCAGCTTTGGCTGTCTCTT
Mouse *Ccl9*	forward	TGGCATATCTGGCTTTGTCA
	reverse	ATGGCTGTAGCTCAAGATGGT
Mouse *Ccl11*	forward	TCCACAGCGCTTCTATTCCT
	reverse	GCAGTTCTTAGGCTCTGGGTT
Mouse *Ccl12*	forward	TCGAAGTCTTTGACCTCAACA

	reverse	GGGAACTTCAGGGGGAAATA
Mouse *Ccl19*	forward	ACTTGCACTTGGCTCCTGAA
	reverse	AGTCTTCCGCATCATTAGCA
Mouse *Ccl20*	forward	GCAAGCGTCTGCTCTTCCTT
	reverse	TTAGGCTGAGGAGGTTCACA
Mouse *Ccl21*	forward	GATGATGACTCTGAGCCTCCT
	reverse	TTCTGCACCCAGCCTTCCT
Mouse *Ccl22*	forward	TGGCAATTCAGACCTCTGATG
	reverse	TTGCTGGAATGGCAGAAGAA
Mouse *Ccl24*	forward	TCATCTTGCTGCACGTCCTTT
	reverse	TAAACCTCGGTGCTATTGCCA
Mouse *Ccl25*	forward	TCTCAGGACCAGAAAGGCATT
	reverse	TGGCGGAAGTAGAATCTCACA
Mouse *Ccl27*	forward	AGGCTGAGTGAGCATGATGGA
	reverse	TTGGCGTTCTAACCACCGA
Mouse *Ccl28*	forward	GCTGTGTGTGTGGCTTTTCAA
	reverse	TACCTCTGAGGCTCTCATCCA
Mouse *Cx3cl1*	forward	TGGCTTTGCTCATCCGCTATCAG
	reverse	CGTCTGTGCTGTGTCGTCTCC
Mouse *Cxcl1*	forward	ACCCAAACCGAAGTCATAG
	reverse	TTGTATAGTGTTGTCAGAAGC
Mouse *Cxcl2*	forward	GGTTGACTTCAAGAACATCCAG
	reverse	TTGAGAGTGGCTATGACTTCTG
Mouse *Cxcl3*	forward	CAGCCACACTCCAGCCTA
	reverse	CACAACAGCCCCTGTAGC
Mouse *Cxcl4*	forward	AGCGATGGAGATCTTAGCTGTGT
	reverse	CCAGGCTGGTGATGTGCTTAA
Mouse *Cxcl5*	forward	AGTCAAGAATCATTGGTTGTTAACCTT
	reverse	TCCGGAGACAATGCAATAGTCA
Mouse *Cxcl7*	forward	GGAGTTCACTGTGCTGATGTGGA
	reverse	CACAGATGAAGCAGCTGGTCAGTAA
Mouse *Cxcl9*	forward	ACAAATCCCTCAAAGACCTCAAACAG
	reverse	ATCTCCGTTCTTCAGTGTAGCAATG
Mouse *Cxcl10*	forward	TGAAAGCGTTTAGCCAAAAAAGG
	reverse	AGGGGAGTGATGGAGAGAGG
Mouse *Cxcl11*	forward	GTTTCCTGTGAGTCTGCCTTTG
	reverse	AGAGCCAGCCATCCCTACC
Mouse *Cxcl12*	forward	CCTCCAAACGCATGCTTCA

	reverse	ACTCTCCTCCCTTCCATTGCA
Mouse *Cxcl13*	forward	CAGGCCACGGTATTCTGGA
	reverse	CAGGGGGCGTAACTTGAATC
Mouse *Cxcl14*	forward	GCTTCATCAAGTGGTACAAT
	reverse	CTGGCCTGGAGTTTTTCTTTCCAT
Mouse *Cxcl15*	forward	CTAGGCATCTTCGTCCGTCC
	reverse	TTGGGCCAACAGTAGCCTTC
Mouse *Cxcl16*	forward	AAACATTTGCCTCAAGCCAGT
	reverse	GTTTCTCATTTGCCTCAGCCT
Mouse *Cxcl17*	forward	ATGAAGCTTCTAGCCTCTCCC
	reverse	CTATAAGGGCAGCGCAAAGCTTGC
Mouse *Il33*	forward	TTGACACATTGAGCATCCA
	reverse	TTGGTCTTTTCCAGAGTCG
Mouse *Mmp1*	forward	CGTGAATGGCAAGGAGATGATGG
	reverse	TCCAGTCACTTTCAGCCCAAATAAC
Mouse *Mmp2*	forward	CGACCACAACCAACTACGATGATG
	reverse	GGGCTGCCACGAGGAATAGG
Mouse *Mmp3*	forward	GACGATGATGAACGATGGACAGAG
	reverse	GCCTTGGCTGAGTGGTAGAGTC
Mouse *Mmp7*	forward	ACTTCAGACTTACCTCGGATCGTAG
	reverse	ATCTCTCCTTGCGAAGCCAATTATG
Mouse *Mmp8*	forward	ACAATCTATGGACCTTCAGACAACC
	reverse	CGGAGTGTGGTAGTAGCATCAAATC
Mouse *Mmp9*	forward	GTATCTGTATGGTCGTGGCTCTAAG
	reverse	GTGCTGTCGGCTGTGGTTC
Mouse *Mmp10*	forward	CCTGTGTTGTCTGTCTCTCCAAGA
	reverse	CGTGCTGACTGAATCAAAGGA
Mouse *Mmp11*	forward	TGCCTCTGCTGCTCCTGTTG
	reverse	GGGTGATGACGGTGACTCTCC
Mouse *Mmp12*	forward	GGACAACTCAACTCTGGCAATAATG
	reverse	CGCTTCATCCATCTTGACCTCTG
Mouse *Mmp13*	forward	AGGTGACTGGCAAACTTGA
	reverse	GGCACTCCACATCTTGGT
Mouse *Mmp14*	forward	GTCTTCAAGGAGCGATGGTTCTG
	reverse	CTCTCGTAGGCAGTATTGATGGATG
Mouse *Mmp15*	forward	GCAGATGGTGACAGCAAGGAAG
	reverse	AATACAGAGCAACAGCAGCAAGG
Mouse *Mmp16*	forward	GGACCAACAGACCGAGATAAAGAAG

	reverse	AACCAATACAAGGAGGCATAAGGC
Mouse *Mmp17*	forward	GGCGGATGCGGAGGATGTAG
	reverse	TGGAGTCAGAAGCAGCAGAGATG
Mouse *Mmp19*	forward	GGCTCCTGTCTATGCTGGCTAC
	reverse	CGGTCTCTTCCTCCTCATCTCTTG
Mouse *Mmp20*	forward	GACAATGCTGAGAAGTGGACTATGG
	reverse	GTGCTGATGGATCTGTGGAATGG
Mouse *Mmp21*	forward	CCGCAAGGAGAGGAACCAATATG
	reverse	CGCCAGCCAGTGAGGATTTG
Mouse *Mmp23*	forward	CGTTGGCGTTGCTGGAGTG
	reverse	TAGCGGCGTCTTCGGGTTAC
Mouse *Mmp24*	forward	GGAGGTAGAGCGGCGTAAGG
	reverse	CAGCACCAGGAGGCAGAGG
Mouse *Mmp25*	forward	CCTGCCCGTCTCTACTACCTTG
	reverse	GCCGTGGAAGTCTGGAGGAG
Mouse *Mmp27*	forward	CCACACACTCGGATTTCCAAGAC
	reverse	ATCTCATCATACCTCCAGCACCAG
Mouse *Mmp28*	forward	GATGGGCAATGGCGACTGTATG
	reverse	TTCTGTAGTGGACGAGGCTCTG
Mouse *β-defensin-1*	forward	GAACACGGTACACAGGCTTCC
	reverse	CCTGAATCACAGATGTCCAAG
Mouse *β-defensin-2*	forward	CTCTCTGGAGTCTGAGTGCCC
	reverse	AGGACGCCTGGCAGAAGGAGG
Mouse *β-defensin-3*	forward	TGCTGCTGTCTCCACCTGC
	reverse	AGTGTTGCCAATGCACCGAT
Mouse *β-defensin-4*	forward	ACATGCATGACCAATGGAGCC
	reverse	CATCTTGCTGGTTCTTC
Mouse *Reg3a*	forward	CTGCTCTCCTGCCTGTTGTT
	reverse	GGAGCGATAAGCCTTGTAACC
Mouse *Reg3b*	forward	AGGCTTATGGCTCCTACTGCT
	reverse	GAAGCCTCAGCGCTATTGAG
Mouse *Reg3d*	forward	CTGTCTTCTCCACGCATCAG
	reverse	CTGCTCCACTTCCATCCATT
Mouse *Reg3g*	forward	TGCCTATGGCTCCTATTGCT
	reverse	CATGGAGGACAGGAAGGAAG
Mouse *St2*	forward	ACCATTACTATCCTGTGCCATTGC
	reverse	CCAAAGCCCAAAGTCCCATTCTC
Human *PPFIA4*	forward	CTCTGCGGATGTTGTCTCCC

	reverse	ATGCTGCCACTGGTTACACG
Human *IL33*	forward	AGGTGACGGTGTTGATGGTAAGATG
	reverse	CAGAGTGTTCCTTGTTGTTGGCATG
Human *MMP1*	forward	CTGGGAGCAAACACATCTGACCTAC
	reverse	TGGAAGGCTTTCTCAATGGCATGG
Human *CXCL3*	forward	GCAGGGAATTCACCTCAAGA
	reverse	GGTGCTCCCCTTGTTCAGTA
Human *ST2*	forward	AGAATTGTCAGGCTCTTCAAGGATC
	reverse	GGTCGCCGTCACACTATAATTGG
Human *GAPDH*	forward	ACCCAGAAGACTGTGGATGG
	reverse	CAGTGAGCTTCCCGTTCAG

For the probes, a FAM fluorescent reporter is coupled to the 5' end, and a TAMRA quencher is coupled to the 3' end.

Supplemental Table. 4. siRNAs used in the present study

Name		Sequence 5′→3′
ST2 siRNA	sense	GCGAAUGUCACCAUAUAUATT
	antisense	UAUAUAUGGUGACAUUCGCTT
CASK siRNA	sense	GCUGUGUCAAGUCACAAAUTT
	antisense	AUUUGUGACUUGACACAGCTT

Supplemental Table. 5. The probes for electrophoretic mobility shift assay (EMSA)

Probe name	Sequence 5′→3′
Neg probe	Biotin- ATACTGCCTCAGCTCTCTCTCAGCATAATATTGAC
WT probe	Biotin- AATAATTGTATTCTGACTCAGCTCTGAGTTTTTTG
Cpt probe	AATAATTGTATTCTGACTCAGCTCTGAGTTTTTTG
Mut probe	Biotin- AATAATTGTATTCCAGAGTGATTCTGAGTTTTTTG

Supplemental Table. 6. Primers for Chromatin Immunoprecipitation (ChIP) PCR analysis

Gene	Primer	Sequence 5′→3′
PPFIA4	forward	AAAGCTGACATGAAGGCACG
	reverse	ACTGTCTCTGCTGGCAAGAT
CXCL3	forward	CCTACCCGTATCCGACTCCA
	reverse	TGGTTGAGACTGGAAAGCCC
MMP1	forward	TAGGCAATTTCCTGTCCAATCACAG
	reverse	ATACTCGACTGTGAGGGAATCTAGTC

Primers for ChIP analysis were designed and produced by Sangon Biotech (Shanghai, China)

Supplemental Table. 7. Primers for PCR genotype analysis

Purpose	Primer	Sequence 5′→3′	Band size
Ppfia4$^{-/-}$	forward	ACTGCACCTCAGTTTCCTTACTT	Homozygotes: 2858 bp
	reverse	ATCAGCACATCTCAGAGACAACAA	Wildtype allele: 457 bp
Ppfia4$^{flox/flox}$	forward	GTGTGTTCAAGTGAGTCATAGCAG	Homozygotes: 208 bp
	reverse	CCACTACTAAACCAGCTAGCCATT	Wildtype allele: 139 bp
Cxcl3$^{flox/flox}$	forward	AAAAGTCAGCCACACTTACCGTAG	Homozygotes: 322 bp
	reverse	TCAGAGAACACCACCAAACAAGAA	Wildtype allele: 254 bp
Gif-Cre	forward	GATCTATGGTGCCAAGGATGACTC	Homozygotes: 423 bp
	reverse	GACAGTCTGTTTTGAGAAGCCAAA	Wildtype allele: none

Supplemental Table. 8. Potential binding sites of AP1 in the *PPFIA4* promoter sequence (PROMO)

**HOMO**	Predicted sequence		Position	Dissimilarity	RE equally	RE query	**MUC**	Predicted sequence		Position	Dissimilarity	RE equally	RE query
AP1	1	TGACTCAGC	-1639;-1631	0	0.019	0.022	AP1	1	TGAGTCA	-1811;-1805	0	0.011	0.015
								2	AGAGTCA	-1124;-1118	2.286726	0.016	0.023
								3	AGAGTCA	463;469	2.286726	0.019	0.028
								4	TGACTAA	-1045;-1039	3.661628	0.021	0.037
								5	TGGGTCA	-1586;-1580	5.69868	0.040	0.042
								6	CAAGTCA	-358;-352	6.609681	0.045	0.048
								7	GCAGTCA	-1498;-1492	7.779169	0.051	0.060
								8	GCAGTCA	-377;-371	7.779169	0.055	0.071
								9	AGTGTCA	-1002;-996	9.02153	0.073	0.082
								10	TTTGTCA	-461;-455	10.396432	0.075	0.097
								11	TATGTCA	-1318;-1312	11.311839	0.088	0.102
								12	CTTGTCA	-85;-79	12.429078	0.094	0.117
								13	TGACCTC	-877;-871	12.562442	0.098	0.122
								14	GATGTCA	945;951	13.598565	0.106	0.136
								15	TGACAGC	-1646;-1640	14.513972	0.113	0.139
								16	GCTGTCA	-1615;-1609	14.513972	0.125	0.142
								17	ACTGTCA	621;627	14.513972	0.133	0.157

*Set the maximum matrix dissimilarity rate to 15%.

Supplemental Table 9. Potential binding sites of NF-κB in the *CXCL3* promoter sequence (JASPAR)

**HOMO**		Predicted sequence	Position	Score	Relative score	**MUS**		Predicted sequence	Position	Score	Relative score
NF-κB1 (MA0105.1)	1	GGGAATTTCC	-78;-69	15.048	1.0	NF-κB1 (MA0105.1)	1	GGGAATTTCC	-72;-63	15.048	1.0
	2	GGGAATTCAC	326;335	11.533	0.914		2	GGAATTTCCC	-71;-62	9.327	0.861
	3	CGGAATTCCC	492;501	10.219	0.883		3	GGGGAATTTC	-73;-64	8.594	0.843
	4	GGAATTTCCC	-77;-68	9.327	0.861						
	5	CGGGCTTTCC	-53;-44	9.001	0.853						
	6	AGGAAGTTCC	-810;-801	7.715	0.822						
RELA (MA0107.1)	1	GGGAATTTCC	-78;-69	15.269	1.0						
	2	CGGGCTTTCC	-53;-44	11.989	0.914						
	3	CGGAATTCCC	492;501	11.537	0.902						
	4	TGTAATTTCC	604;613	9.531	0.849						
	5	GGGAATTCAC	326;335	8.623	0.826						
	6	AGGAAGTTCC	-810;-801	7.754	0.803						

*Total putative sites were predicted with relative profile score threshold 80%.

Supplemental Table 10. Potential binding sites of NF-κB in the *MMP1* promoter sequence (JASPAR)

**HOMO**		Predicted sequence	Position	Score	Relative score	**MUS**		Predicted sequence	Position	Score	Relative score
NF-κB1 (MA0105.1)	1	GGCAATTTCC	-1997;-1988	10.404	0.887	NF-κB1 (MA0105.1)	1	GGAAATTCAC	-1522;-1513	7.611	0.819
	2	GAGGATTTCC	-1707;-1698	9.499	0.865						
	3	GGGACATTTC	484;493	8.963	0.852						
	4	GGGAGCTTCC	-17;-8	8.544	0.842						
RELA (MA0107.1)	1	GGCAATTTCC	-1997;-1988	11.101	0.890						
	2	GAGGATTTCC	-1707;-1698	10.598	0.877						
	3	GGGAGCTTCC	-17;-8	10.133	0.865						

*Total putative sites were predicted with relative profile score threshold 80%.

Supplemental Table. 11. Molecule compounds

Name	CAS	Docking score (Ledock)	Docking score (Vina)	MMGBSA (kcal/mol)
Macranthoidin A	140360-29-8	-10.23	-13	-42.04
Anemoside B4	129741-57-7	-9.46	-12.5	-40.32
Methylprotodioscin	54522-52-0	-8.84	-12	-32.23
Ziyu-glycoside I	35286-58-9	-8.31	-12.3	-32.17
Fenebrutinib (GDC-0853)	1434048-34-6	-8.22	-12.6	-42.69
KPT 9274 (ATG-019)	1643913-93-2	-8.03	-12.2	-29.31
Hederacoside C	14216-03-6	-7.89	-12.3	-32.47
A-1331852	1430844-80-6	-7.74	-12.5	-57.03
NVS-ZP7-4	2349367-89-9	-7.63	-12	-38.49
FIN56	1083162-61-1	-7.41	-12	-33.23
Khasianine	32449-98-2	-7.12	-13.7	-37.81
Ledipasvir (GS5885)	1256388-51-8	-7.03	-12.5	-39.90
kira6	1589527-65-0	-6.94	-12.4	-35.87
BMS-986142	1643368-58-4	-6.68	-12.6	-35.27
LY2090314	603288-22-8	-6.55	-12.5	-28.24
Senegenin	2469-34-3	-6.53	-12.4	-30.01
Phytolaccagenin	1802-12-6	-6.35	-12.6	-27.86
Asiaticoside	16830-15-2	-6.09	-12.2	-45.58
Berbamine	478-61-5	-6.07	-12	-29.34
Doxycycline	564-25-0	-6.03	-12.4	-37.75

Supplemental Table. 12. Molecular docking of kira6 with PPFIA4, CASK, AKT1 and p65

Protein	Docking score (kcal/mol)
PPFIA4	-8.20
CASK	-6.12
AKT1	-5.81
p65	-5.45

Supplemental Table. 13. Binding free energy of kira6 with PPFIA4, CASK, AKT1 and p65

Protein	ΔG (kJ/mol)
PPFIA4	-89.69±9.50
CASK	-44.20±18.11
AKT1	-39.56±8.81
p65	-34.63±6.50

Supplemental Table. 14. A glossary of abbreviations

Abbreviations	Full names
H. pylori	*Helicobacter pylori*
CagA	Cytotoxin-associated gene A
GECs	gastric epithelial cells
PPFIA4	protein tyrosine phosphatase receptor type F polypeptide interacting protein alpha 4
CASK	calcium/calmodulin-dependent serine protein kinase
SAH	single alpha helix
SAMs	sterile alpha motifs
CaMK	CaM kinase
PDZ	Postsynaptic density-95/Discs large/Zonula occludens-1
SH3	Src homology 3
GK	Guanylate kinase
IL	interleukin
WT	wild-type
Ppfia4$^{\Delta GEC}$ mice	*Ppfia4* GEC-specific knockout mice
Cxcl3$^{\Delta GEC}$ mice	*Cxcl3* GEC-specific knockout mice
Il33$^{-/-}$ mice	*Il33* knockout mice
Ppfia4$^{-/-}$ mice	*Ppfia4* knockout mice
Il33$^{-/-}$*Ppfia4*$^{-/-}$ mice	*Il33* and *Ppfia4* double-knockout mice
BM	bone marrow
p.i.	post infection
H&E	haematoxylin and eosin
AP1	activator protein-1
AKT1	AKT serine/threonine kinase 1
NF-κB	nuclear factor kappa B
IFN-γ	interferon gamma
MMP	matrix metalloproteinase
ZO-1	zonula occludens-1
G-MDSCs	granulocytic myeloid-derived suppressor cells

moDCs	monocyte-derived DCs
i-moDCs	immature moDCs
cDCs	conventional DCs
GS	gastritis
IM	intestinal metaplasia
GC	gastric cancer
DEGs	differentially expressed genes
KEGG	Kyoto Encyclopedia of Genes and Genomes
GO	Gene ontology
DRUG-seq	Digital RNA with pertUrbation of Genes sequencing
SMART-seq	Switching Mechanism At the 5' end of RNA Template sequencing
SLAM-seq	Thiol(SH)-Linked Alkylation for the Metabolic sequencing of RNA
scRNA-seq	single-cell RNA sequencing
UMAP	uniform manifold approximation and projection
tSNE	t-distributed stochastic neighbor embedding
GSEA	gene set enrichment analysis
TEER	transepithelial electrical resistance
IP	immunoprecipitation
MS	mass spectrometry
TMT-LC-MS/MS	tandem mass tag combined with liquid chromatography-tandem mass spectrometry
RMSD	solvent-accessible surface area
Rg	radius of gyration
SASA	solvent-accessible surface area
RMSF	root-mean-square fluctuation
SPR	surface plasmon resonance
KD	dissociation constants
IRE1α	inositol-requiring enzyme 1α
SPEM	spasmolytic polypeptide-expressing metaplasia
EMSA	electrophoretic mobility shift assay
ChIP	chromatin immunoprecipitation
